# Ligand-directed symmetry reduction and stereoselective assembly of *C*_3_-symmetric topologically chiral trefoil knots

**DOI:** 10.1039/d6sc05627j

**Published:** 2026-10-23

**Authors:** Zheng Cui, Junli Guo, Liyan Hao, Huiyong Wang, Jianji Wang

**Affiliations:** a Key Laboratory of Green Chemical Media and Reactions (Ministry of Education), Collaborative Innovation Center of Henan Province for Green Manufacturing of Fine Chemicals, School of Chemistry and Chemical Engineering, Henan Normal University Xinxiang Henan 453007 P. R. China cuizheng@htu.edu.cn hywang@htu.edu.cn jwang@htu.edu.cn

## Abstract

The stereoselective synthesis of low-symmetry topologically chiral molecular knots remains challenging because lowering symmetry creates competing assembly pathways. Here we report a ligand-directed strategy that couples symmetry reduction with handedness control in molecular trefoil knots. Coordination-driven self-assembly of an l-β-homoalanine-derived oriented ligand, (*S*)-L1, with three related binuclear half-sandwich Rh/Ir organometallic building blocks affords *C*_3_-symmetric Λ-handed trefoil knots Λ-Rh-1, Λ-Rh-1Br and Λ-Ir-1. The head-to-tail ligand arrangement removes the *C*_2_ symmetry elements of a *D*_3_-like framework while retaining the *C*_3_ rotational relationship among the three repeat units. The amino-acid stereocenter relays local point chirality into global topological handedness. The enantiomeric ligand (*R*)-L1 gives the mirror-related Δ-handed knots Δ-Rh-1, Δ-Rh-1Br and Δ-Ir-1. Crystallographic analysis, NMR spectroscopy, high-resolution mass spectrometry and mirror-image circular dichroism spectra collectively corroborate the trefoil-knot assignments, molecular compositions and enantiomeric relationships. Assembly from an equimolar mixture of (*S*)-L1 and (*R*)-L1 supports homochiral self-sorting into a racemic mixture of trefoil knots. An analogue of (*S*)-L1 with less differentiated coordination termini gives D-shaped metallacycles, whereas analogues with shorter amino-acid segments give access to figure-eight knots and tetranuclear metallomacrocycles. These results show that ligand directionality and point chirality can be integrated to control both the symmetry and handedness of molecular trefoil knots.

## Abstract 2

Encoding directionality and point chirality in a single ligand guides head-to-tail assembly of *C*_3_-symmetric trefoil knots, coupling symmetry reduction with control of topological handedness.

## Graphical abstract

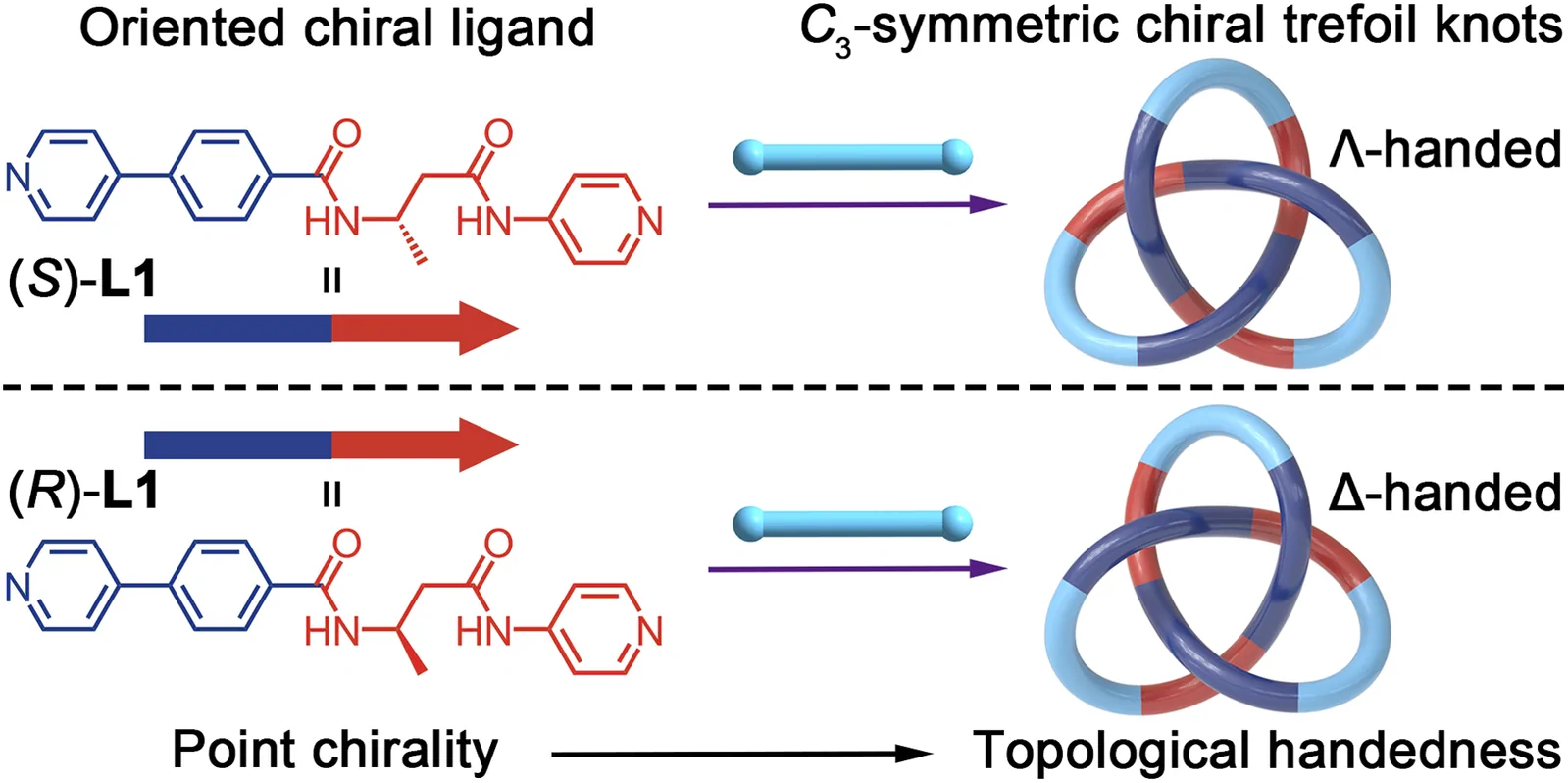

## Introduction

Molecular knots are fundamental topological architectures in which a molecular strand is entangled into a closed knotted loop.^1,2^ Beyond their aesthetic appeal, such structures impose topological constraints on molecular conformation, local environment and strand dynamics, which can influence reactivity, recognition, mechanical properties and chiroptical behavior.^3–7^ Trefoil knots, the simplest nontrivial knots, are intrinsically topologically chiral because their mirror images cannot be superimposed without cutting the strand or allowing it to pass through itself.^8–11^ Controlling the handedness, symmetry and local environment of molecular trefoil knots is therefore an important step toward connecting molecular topology with function.^12–15^

Over the past decades, molecular knots with diverse topologies, including trefoil,^16,17^ figure-eight,^18^ pentafoil^19–21^ and more complex knots,^22–25^ have been synthesized through covalent templating, metal-ion templating, dynamic covalent chemistry and coordination-driven self-assembly. Nevertheless, stereoselective access to a defined topological handedness remains demanding for topologically chiral knots because such chirality is generated globally by the entire entangled backbone rather than by a localized stereogenic element.^14,26–31^ Previous studies by Leigh, Fujita, Nitschke, Jin, Zhang and others have established strategies for controlling knot handedness.^14,22,26–32^ Integrating this stereochemical control with deliberate symmetry reduction presents a further design challenge. Many reported trefoil knots adopt high-symmetry or pseudo-high-symmetry arrangements, typically with *D*_3_-like symmetry (Fig. 1a).^26,32^ Although such symmetry can facilitate efficient assembly, reducing it provides opportunities to differentiate local environments through the arrangement of the components.^33–35^ The stereoselective synthesis of low-symmetry trefoil knots, particularly *C*_3_-symmetric topologically chiral variants, therefore remains an important synthetic target.

**Fig. 1:**
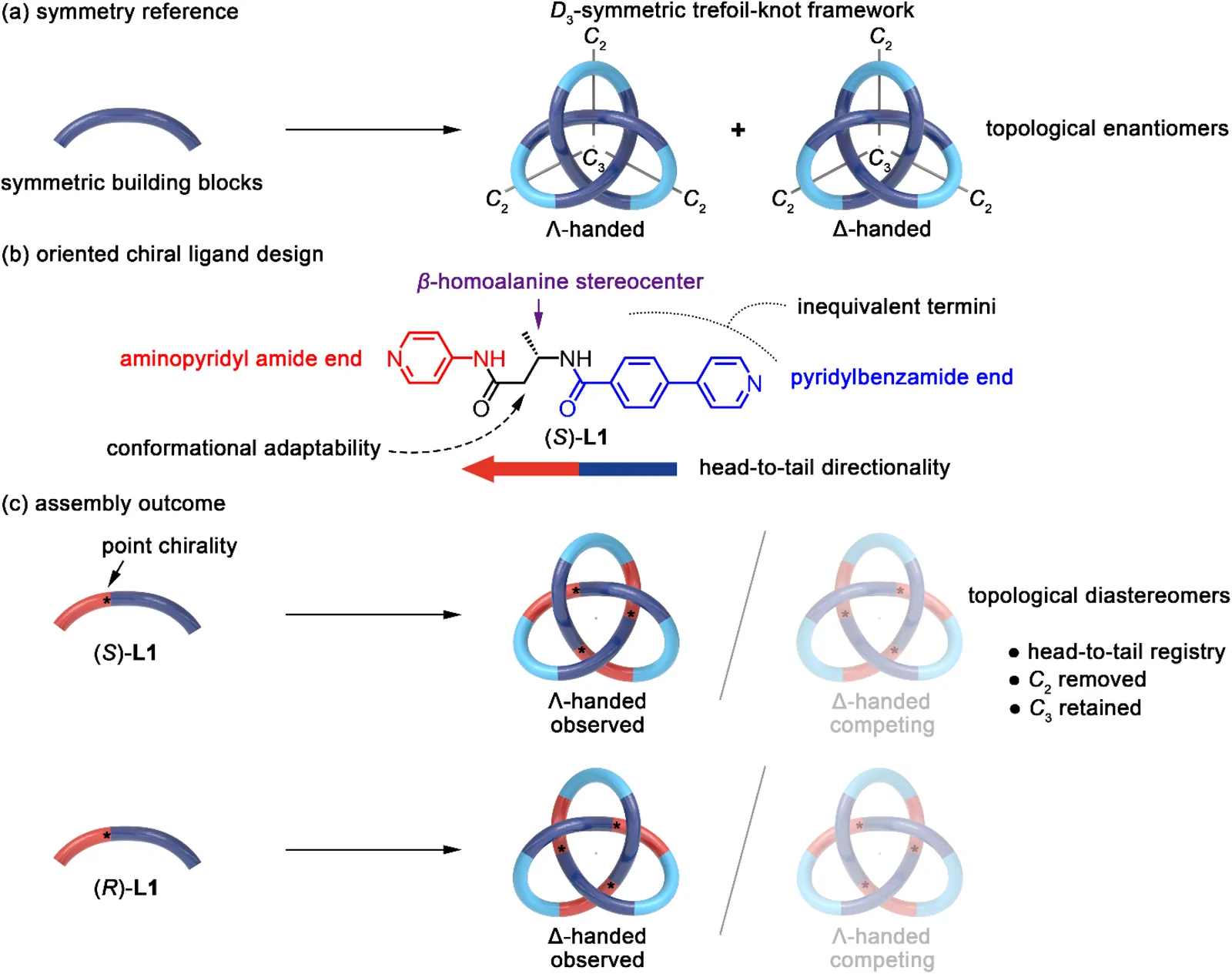
Design principle for symmetry-reduced, topologically chiral trefoil-knot assembly. (a) Schematic *D*_3_-like trefoil-knot reference containing a *C*_3_ rotational axis and three *C*_2_ rotational axes, with the Λ- and Δ-handed structures constituting a pair of topological enantiomers. (b) Design of the oriented enantiopure ligand (*S*)-L1, which combines constitutionally differentiated coordination termini with a stereogenic l-β-homoalanine-derived segment, thereby incorporating head-to-tail directionality and local stereochemical information within a single ligand. (c) For a fixed ligand configuration, the Λ- and Δ-handed *C*_3_ trefoil knots are diastereomeric. In the assemblies observed here, (*S*)-L1 gives the Λ-handed structure with a head-to-tail ligand registry, whereas inversion of the ligand configuration in (*R*)-L1 gives the mirror-related Δ-handed assembly.

A major difficulty in constructing low-symmetry knotted molecules is that symmetry reduction often increases the number of orientational and stereochemical possibilities during self-assembly.^33,36,37^ Lowering the symmetry of a building block is therefore not sufficient by itself; the reduced symmetry must be translated into a defined assembly outcome.^34,38,39^ Among possible desymmetrization strategies,^37,40–42^ we focused on oriented ligands as a way to introduce directionality into the knotted strand (Fig. 1b). For such ligands, the orientational challenge is to select a uniform head-to-tail sequence over alternatives containing head-to-head and tail-to-tail junctions. A uniform head-to-tail arrangement would preclude the *C*_2_ symmetry elements of a *D*_3_-like trefoil-knot framework while retaining the *C*_3_ rotational relationship among the three repeating units (Fig. 1c). Here, symmetry reduction refers specifically to the loss of these *C*_2_ symmetry elements. If a stereogenic unit is incorporated into such an oriented ligand, local point chirality may also be relayed into the global topology, providing a route to stereoselective formation of low-symmetry topologically chiral knots.^43–46^ For a fixed ligand configuration, the corresponding Λ- and Δ-handed knots are diastereomers (Fig. 1c). The design question is therefore whether orientational selection and handedness control can be achieved simultaneously within one ligand-directed self-assembly process.

Herein, we report a ligand-directed strategy for the stereoselective assembly of *C*_3_-symmetric topologically chiral trefoil knots (Fig. 2). The key design element is an oriented chiral bis-pyridyl ligand, (*S*)-L1, derived from l-β-homoalanine (Fig. 1b). The amino-acid segment introduces both point chirality and intrinsic N-to-C directionality, while two inequivalent pyridyl-containing termini reinforce the head-to-tail character of the ligand. In addition, the flexible amino-acid segment provides conformational adaptability, and the aromatic coordination termini provide π surfaces that favor interligand aromatic stacking within the knotted framework. Coordination-driven self-assembly of (*S*)-L1 with three related binuclear half-sandwich Rh/Ir organometallic building blocks B1(OTf)_2_–B3(OTf)_2_ affords *C*_3_-symmetric Λ-handed trefoil knots Λ-Rh-1, Λ-Rh-1Br and Λ-Ir-1. Conversely, the enantiomeric ligand (*R*)-L1 gives the mirror-related *C*_3_-symmetric Δ-handed knots Δ-Rh-1, Δ-Rh-1Br and Δ-Ir-1. Ligand-analogue studies further probe how terminal differentiation and the structure of the amino-acid segment affect the assembly outcome. Together, these results support the integration of orientational symmetry reduction and point-to-topological chirality transfer within a single ligand design.

**Fig. 2:**
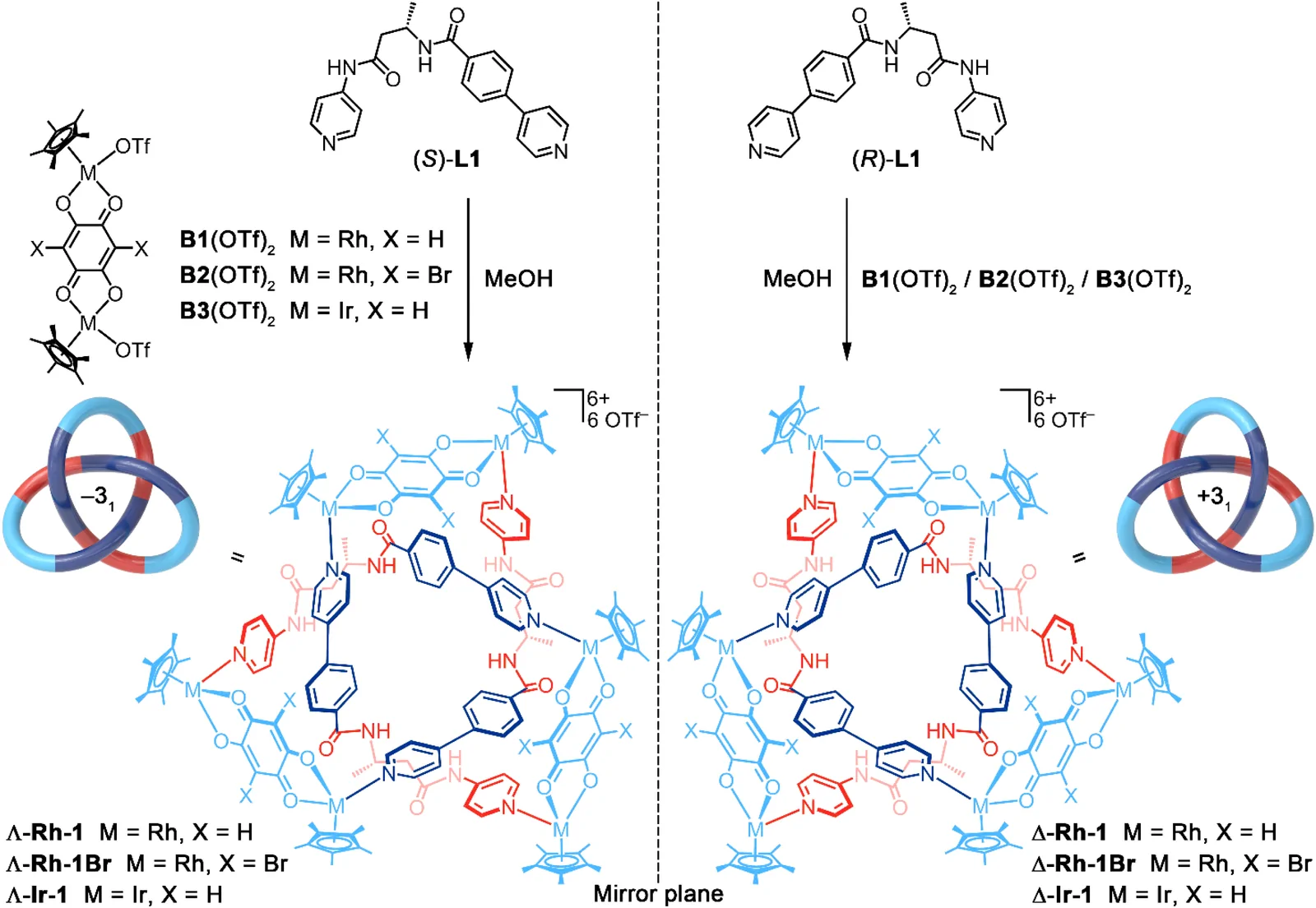
Stereoselective self-assembly of the trefoil-knot series. Coordination-driven self-assembly of (*S*)-L1 with the binuclear half-sandwich building blocks B1(OTf)_2_–B3(OTf)_2_ affords the *C*_3_-symmetric Λ-handed trefoil knots Λ-Rh-1, Λ-Rh-1Br, and Λ-Ir-1, respectively, whereas the enantiomeric ligand (*R*)-L1 gives the corresponding Δ-handed assemblies Δ-Rh-1, Δ-Rh-1Br, and Δ-Ir-1.

## Results and discussion

To realize low-symmetry topologically chiral trefoil knots, we first designed an oriented chiral bis-pyridyl ligand that integrates directionality, point chirality, conformational adaptability and aromatic π surfaces. The ligand (*S*)-L1 was prepared from l-β-homoalanine by installing two different pyridyl-containing coordination termini: a 4-aminopyridine-derived pyridyl amide terminus and a 4-(pyridin-4-yl)benzamide terminus (Fig. 1b and S1). Chiral supercritical fluid chromatography confirmed enantiomeric excesses above 99% for (*S*)-L1 and (*R*)-L1, with no peak attributable to the opposite enantiomer detected under the analytical conditions used (Fig. S200). The amino-acid segment provides intrinsic N-to-C directionality and a stereogenic center, while the two consecutive sp^3^-hybridized carbon atoms in the β-amino-acid segment introduce a flexible bending point that may facilitate the folding and intertwining required for knot formation. In addition, the aromatic coordination termini provide π surfaces that favor interligand aromatic stacking within the resulting knotted framework. Thus, this ligand was designed not only to lower symmetry through head-to-tail differentiation, but also to promote the folded conformations needed for trefoil-knot assembly.

We initially examined the coordination-driven self-assembly of (*S*)-L1 with the binuclear half-sandwich Rh building block B1(OTf)_2_ in methanol. The Rh⋯Rh separation in B1^2+^ is approximately 8.0 Å, providing space for aromatic ligand segments to interweave during assembly. Slow vapor diffusion of diethyl ether into the methanol solution afforded brown single crystals of Λ-Rh-1. Single-crystal X-ray diffraction revealed that Λ-Rh-1 consists of three oriented ligands and three binuclear Rh building blocks arranged alternately into a closed three-crossing trefoil-knot framework (Fig. 3a, S2 and S3). The structure corresponds to a Λ-handed (−3_1_) trefoil knot. In the notation 3_1_,^47^ 3 denotes the minimal crossing number and the subscript distinguishes the knot within that class; −3_1_ and +3_1_ denote the mirror-related forms shown in Fig. 2. Notably, the three oriented ligands are directionally aligned along the cyclic backbone through head-to-tail connection by the B1^2+^ units, which precludes the *C*_2_ rotational symmetry elements commonly present in higher-symmetry trefoil-knot frameworks while retaining an overall *C*_3_-symmetric architecture. Thus, Λ-Rh-1 represents a symmetry-reduced trefoil knot with a defined head-to-tail ligand sequence and topological handedness (Fig. 3b). Single-crystal structures of B1(OTf)_2_–B3(OTf)_2_ show an *anti* arrangement of the two Cp* groups within each binuclear unit, whereas these groups adopt a *syn* arrangement in the crystallographically characterized assemblies (Fig. S33 and Table S9).

**Fig. 3:**
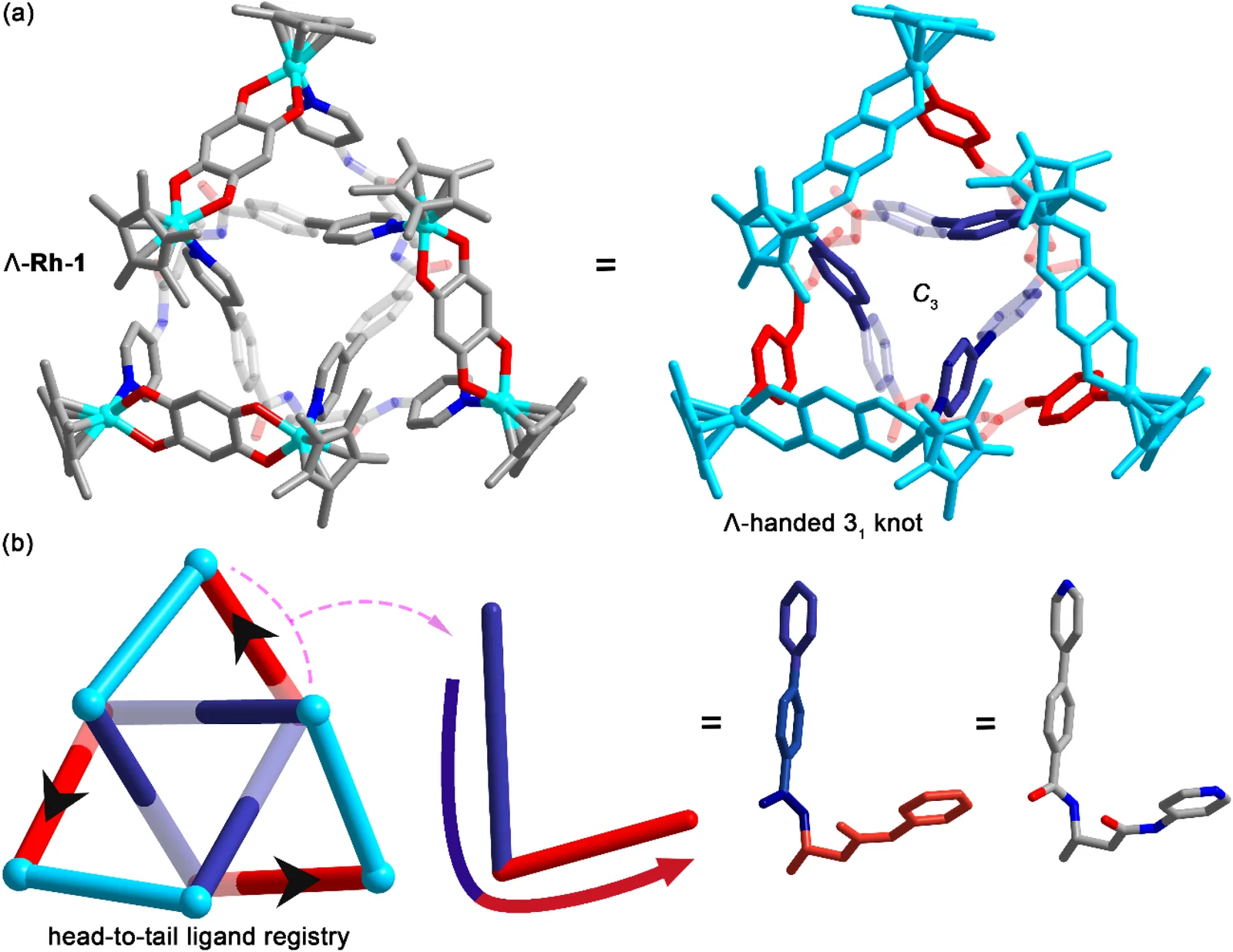
Solid-state structure and directional ligand arrangement of Λ-Rh-1. (a) Single-crystal X-ray structure of Λ-Rh-1, showing the *C*_3_-symmetric three-crossing trefoil-knot framework constructed from three (*S*)-L1 ligands and three binuclear Rh building blocks. (b) Simplified topological representation of the Λ-handed 3_1_ knot and the head-to-tail registry of the three oriented ligands within the cyclic coordination framework. The three ligand–building-block repeat units adopt a *C*_3_-symmetric arrangement with ligand directionality encoded by the head-to-tail connection. Counteranions and hydrogen atoms are omitted for clarity. Color code: C, gray; N, blue; O, red; Rh, turquoise.

A closer inspection of the crystal structure reveals how the head-to-tail oriented ligand sequence is organized within the knotted framework. The aromatic segments of adjacent ligands are brought into close proximity within the intertwined backbone, forming interligand π–π stacking interactions at the strand crossings (Fig. S5). In addition, C–H⋯π contacts around the metal–ligand junctions and hydrogen-bonding contacts involving one OTf^−^ counterion are observed within the compact folded arrangement (Fig. S6 and S7). These contacts help stabilize the folded trefoil-knot framework.

The trefoil-knot architecture of Λ-Rh-1 is retained as the predominant assembly in methanol solution, as supported by NMR spectroscopy and high-resolution electrospray ionization mass spectrometry (HR ESI-MS). In the ^1^H NMR spectrum of Λ-Rh-1 in CD_3_OD (Fig. 4a and S34), the Cp* resonances and the aromatic proton signals of the binuclear building block each appear as two sets, consistent with the *C*_3_-symmetric oriented structure observed crystallographically and its reduced symmetry relative to a *D*_3_-like framework. Each binuclear repeat unit contains two inequivalent metal/Cp* environments, whereas the three repeat units remain related by *C*_3_ symmetry. Compared with free ligand (*S*)-L1, the aromatic proton signals of the ligand in Λ-Rh-1 are generally shifted upfield (Fig. 4a), supporting their involvement in aromatic stacking interactions within the knot. The ^1^H DOSY spectrum displays one principal diffusion coefficient for the non-solvent resonances (*D* = 2.83 × 10^−10^ m^2^ s^−1^; Fig. 4b and S36), consistent with the presence of a predominant solution-state assembly. Using a modified Stokes–Einstein equation based on an oblate spheroid model,^48,49^ this diffusion coefficient gives hydrodynamic dimensions for this assembly (semimajor axis × semiminor axis: 15.7 Å × 10.7 Å; Fig. 4c and S37), which are comparable to the crystallographic dimensions of the Λ-Rh-1^6+^ cation (radius × half-height: 13.0 Å × 8.9 Å). In addition, the ^1^H–^1^H NOESY spectrum shows H_h_⋯H_a_ and H_h_⋯H_b_ correlations between the pyridylbenzamide phenyl and aminopyridyl segments of different ligands (Fig. 4d). These contacts occur at the crystallographic strand crossings, with H⋯H separations of approximately 3.93 and 3.99 Å, respectively. The H_h_⋯H_k_ correlation is likewise consistent with an interligand phenyl–pyridyl contact at a crossing, for which the crystallographic separation is approximately 3.34 Å. Further correlations involving the ligand methylene protons are shown in Fig. S38. HR ESI-MS further shows a quadruply charged ion at *m*/*z* 805.3751, assigned to [Λ-Rh-1 – 4 OTf]^4+^ (calcd *m*/*z* 805.3760; Fig. 4e and S170), with an isotope distribution matching the calculated pattern. Taken together, the ^1^H NMR spectral features, through-space correlations, and diffusion and compositional data support retention of the knotted arrangement in solution.

**Fig. 4:**
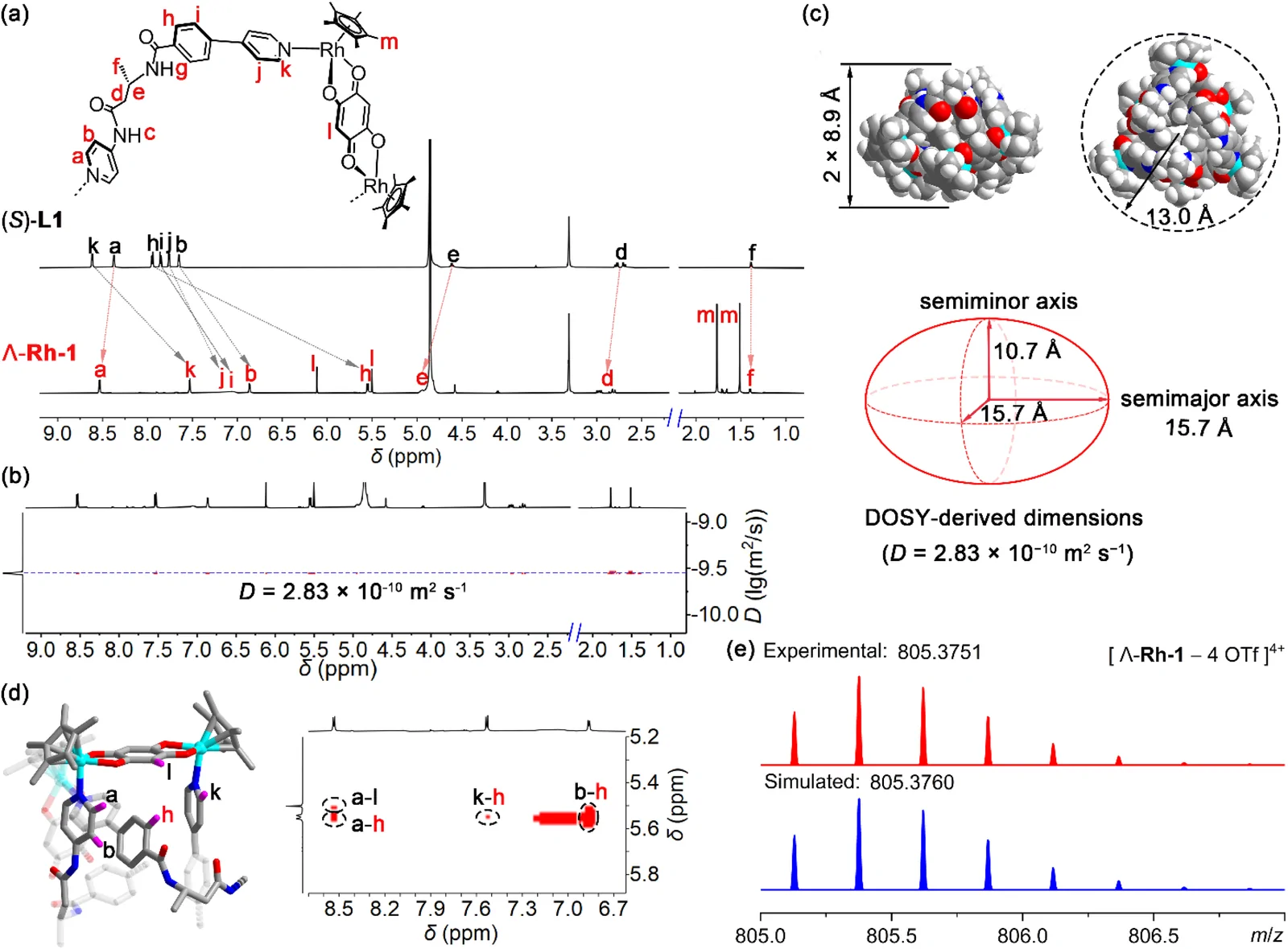
Solution-state characterization of Λ-Rh-1. (a) ^1^H NMR spectra (600 MHz) of free (*S*)-L1 and Λ-Rh-1 in CD_3_OD, showing the spectral changes accompanying coordination and self-assembly. (b) ^1^H DOSY spectrum of Λ-Rh-1, displaying one principal diffusion coefficient for the non-solvent resonances (*D* = 2.83 × 10^−10^ m^2^ s^−1^). (c) Comparison of the hydrodynamic dimensions estimated from the DOSY-derived diffusion coefficient using an oblate-spheroid model with the corresponding crystallographic dimensions of the Λ-Rh-1^6+^ cation. (d) Selected region of the ^1^H–^1^H NOESY spectrum and the corresponding through-space contacts mapped onto the crystallographic structure. (e) Experimental and calculated isotope patterns of the representative HR ESI-MS signal assigned to [Λ-Rh-1 – 4 OTf]^4+^. Counteranions and nonessential hydrogen atoms are omitted for clarity. Color code: C, gray; H, white/pink; N, blue; O, red; Rh, turquoise.

To examine the generality of this ligand-directed assembly strategy, we next replaced B1(OTf)_2_ with two related binuclear half-sandwich organometallic building blocks, B2(OTf)_2_ and B3(OTf)_2_. In B2(OTf)_2_, the tetradentate bridge of B1(OTf)_2_ is replaced by a brominated analogue, whereas B3(OTf)_2_ is the Ir analogue of B1(OTf)_2_ (Fig. 2). Under analogous self-assembly conditions, (*S*)-L1 afforded Λ-Rh-1Br and Λ-Ir-1, respectively, demonstrating that the oriented chiral ligand can direct trefoil-knot formation across related binuclear organometallic platforms.

The solution-state data of Λ-Rh-1Br are consistent with a trefoil-knot assembly analogous to Λ-Rh-1. Its ^1^H NMR spectrum closely resembles that of Λ-Rh-1, except for the absence of the bridge aromatic proton signals due to bromination of the tetradentate bridge (Fig. 5a and S59). The ^1^H DOSY spectrum displays one principal diffusion coefficient for the non-solvent resonances (*D* = 2.79 × 10^−10^ m^2^ s^−1^; Fig. S61), consistent with a well-defined solution-state assembly. Diagnostic ^1^H–^1^H NOESY correlations are observed between the pyridyl protons of the aminopyridyl amide segment and the aromatic protons of the pyridylbenzamide segment of the ligand (Fig. 5b and S62), supporting an interligand arrangement comparable to that in Λ-Rh-1. HR ESI-MS provides further compositional evidence, showing a quadruply charged ion at *m*/*z* 923.9911 corresponding to [Λ-Rh-1Br – 4 OTf]^4+^ (calcd *m*/*z* 923.9906; Fig. S175), with the experimental isotope distribution in good agreement with the calculated pattern. Attempts to crystallize the brominated assembly yielded powdery solids, without single crystals suitable for X-ray diffraction. The trefoil topology and Λ-handedness are assigned by comparison with the crystallographically characterized Λ-Rh-1 and Λ-Ir-1 and their complementary solution-state data.

**Fig. 5:**
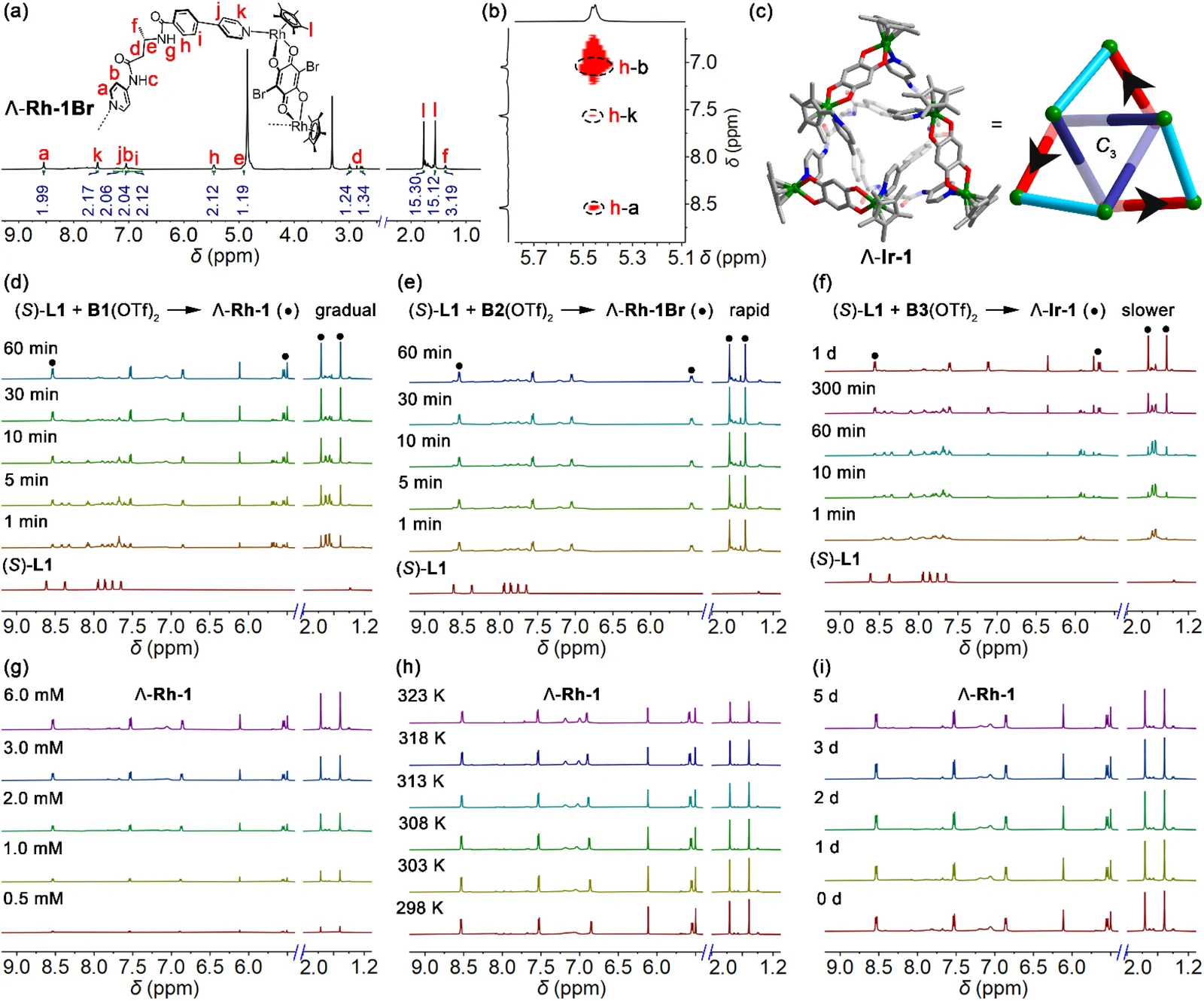
Formation and solution-state behavior of the (*S*)-L1-derived trefoil-knot assemblies. (a) ^1^H NMR spectrum (600 MHz) of Λ-Rh-1Br in CD_3_OD. (b) Selected region of the ^1^H–^1^H NOESY spectrum of Λ-Rh-1Br, showing diagnostic through-space correlations between ligand segments brought into proximity within the interlaced framework. (c) Single-crystal X-ray structure and simplified topological representation of Λ-Ir-1, confirming the corresponding *C*_3_-symmetric Λ-handed trefoil-knot architecture. (d–f) Time-dependent ^1^H NMR spectra recorded during the self-assembly of (*S*)-L1 with B1(OTf)_2_, B2(OTf)_2_, and B3(OTf)_2_, respectively. Selected resonances corresponding to the trefoil-knot assemblies are highlighted with black circles. (g) Concentration-dependent ^1^H NMR spectra of Λ-Rh-1 recorded over the concentration range of 0.5–6.0 mM. (h) Variable-temperature ^1^H NMR spectra of Λ-Rh-1 recorded from 298 to 323 K. (i) Time-dependent ^1^H NMR spectra of Λ-Rh-1 from a freshly prepared sample to 5 days. Counteranions and hydrogen atoms are omitted for clarity. Color code: C, gray; N, blue; O, red; Ir, green.

For Λ-Ir-1, single-crystal X-ray diffraction confirmed an isostructural *C*_3_-symmetric Λ-handed trefoil-knot framework (Fig. 5c), showing that replacement of the Rh centers by Ir centers preserves the head-to-tail ligand arrangement and knotted framework. The solid-state structure of Λ-Ir-1 displays a low-symmetry knotted topology and interligand aromatic stacking motif closely comparable to those of Λ-Rh-1 (Fig. S8). In CD_3_OD, the ^1^H NMR spectrum of Λ-Ir-1 shows two sets of Cp* resonances and aromatic proton signals from the binuclear building block (Fig. S82), indicating that the inequivalent environments generated by the oriented *C*_3_ framework are preserved in solution. The ^1^H DOSY spectrum displays one principal diffusion coefficient for the non-solvent resonances (Fig. S84 and S85), supporting a well-defined solution-state assembly. In addition, diagnostic ^1^H–^1^H NOESY correlations are observed between the aromatic protons of the pyridylbenzamide segment and both the aliphatic protons of the amino-acid residue and the pyridyl protons of the aminopyridyl amide segment (Fig. S86). These through-space correlations agree with the close spatial arrangement of ligand segments in the crystallographic knot. HR ESI-MS further supports the formation of Λ-Ir-1, with a quadruply charged ion observed at *m*/*z* 939.7084 for [Λ-Ir-1 – 4 OTf]^4+^ (calcd *m*/*z* 939.7115; Fig. S179), again showing good agreement between experimental and calculated isotope distributions. Together, the characterization of Λ-Rh-1, Λ-Rh-1Br and Λ-Ir-1 indicates that the oriented chiral ligand functions as a transferable design element for accessing a family of *C*_3_-symmetric Λ-handed trefoil knots.

To establish whether the knots form before isolation, we monitored the self-assembly mixtures by time-dependent ^1^H NMR spectroscopy (Fig. 5d–f, S39, S63 and S87). Signals characteristic of Λ-Rh-1 developed progressively, with formation largely complete after approximately 60 min. For Λ-Rh-1Br, conversion was largely complete within 5 min. Formation of Λ-Ir-1 was slower, requiring approximately 1 day at room temperature. Corresponding studies of Δ-Rh-1, Δ-Rh-1Br and Δ-Ir-1 showed similar time-dependent profiles (Fig. S51, S76 and S100). Thus, the characteristic knot spectra develop in the reaction mixtures before precipitation or crystallization. For Λ-Rh-1/Δ-Rh-1, the progressive growth of oppositely signed circular dichroism (CD) responses from the (*S*)-L1 and (*R*)-L1 reaction mixtures over 1–60 min further shows that chiroptical differentiation develops during assembly in solution (Fig. 6f and S205).

The persistence of the assemblies in solution was examined by concentration-, temperature- and time-dependent ^1^H NMR spectroscopy. Λ-Rh-1 retained essentially the same spectral pattern over 0.5–6.0 mM, from 298 to 323 K, and upon standing for up to 5 days (Fig. 5g–i, S40, S43 and S44). Further dilution to 0.1 mM did not appreciably alter the spectrum (Fig. S40). Λ-Ir-1 likewise showed no substantial spectral changes under the conditions examined (Fig. S88, S91 and S92). For Λ-Rh-1Br, the spectrum remained essentially unchanged over time, whereas heating and, more markedly, dilution reduced the relative intensities of the trefoil-knot resonances and increased those of additional resonances, consistent with changes in solution speciation (Fig. S64, S68 and S69). Concentration- and time-dependent ^1^H NMR studies of Δ-Rh-1, Δ-Rh-1Br and Δ-Ir-1 showed behavior similar to that of their corresponding Λ enantiomers (Fig. S52, S53, S77, S78, S101 and S102). The molar CD responses of Λ-Rh-1Br/Δ-Rh-1Br also weakened appreciably upon dilution, whereas those of Λ-Rh-1/Δ-Rh-1 and Λ-Ir-1/Δ-Ir-1 changed only slightly (Fig. S206–S208).

Consistent with the chirality-transfer design, self-assembly of the enantiomeric ligand (*R*)-L1 with B1(OTf)_2_, B2(OTf)_2_ and B3(OTf)_2_ afforded the corresponding Δ-handed trefoil knots Δ-Rh-1, Δ-Rh-1Br and Δ-Ir-1 (Fig. 2), respectively. Single-crystal X-ray diffraction confirms that Δ-Rh-1 and Δ-Ir-1 have trefoil-knot frameworks that are mirror-related to those of Λ-Rh-1 and Λ-Ir-1, respectively (Fig. 6a and b). The refined absolute structures agree with the known configurations of the ligand stereocenters; the corresponding absolute-structure parameters are listed in Tables S2 and S3. The ^1^H NMR (Fig. S48, S73 and S96), ^1^H DOSY NMR (Fig. S50, S75 and S98) and HR ESI-MS data (Fig. S172, S177 and S181) of Δ-Rh-1, Δ-Rh-1Br and Δ-Ir-1 are consistent with the formation of the corresponding trefoil-knot assemblies. Full-range ESI-MS spectra are provided for all six trefoil-knot assemblies (Fig. S169, S171, S174, S176, S178 and S180). The ^13^C NMR, HSQC and HMBC spectra further support the resonance assignments (Fig. S45–S47, S54–S56, S70–S72, S79–S81, S93–S95 and S103–S105). The CD spectra of Λ-Rh-1/Δ-Rh-1 (Fig. 6c and S202), Λ-Rh-1Br/Δ-Rh-1Br (Fig. 6d and S203) and Λ-Ir-1/Δ-Ir-1 (Fig. 6e and S204) in methanol show mirror-image Cotton effects with comparable intensities and opposite signs, corroborating the enantiomeric relationships of each Λ/Δ pair in solution. Compared with the corresponding free-ligand references on a three-ligand basis, the knots show enhanced CD responses and additional bands in the 300–600 nm region (Fig. 6c–e; see Fig. S201 for the free-ligand CD spectra). These changes show that coordination and assembly alter the chiroptical response. The absorption dissymmetry factors (*g*_abs_ = Δ*ε*/*ε*), calculated from the corresponding CD and UV-vis spectra (Fig. S193–S195 and S202–S204), are summarized in Table S1. Together with the structural evidence, these results support ligand-controlled inversion of the overall molecular stereochemistry.

**Fig. 6:**
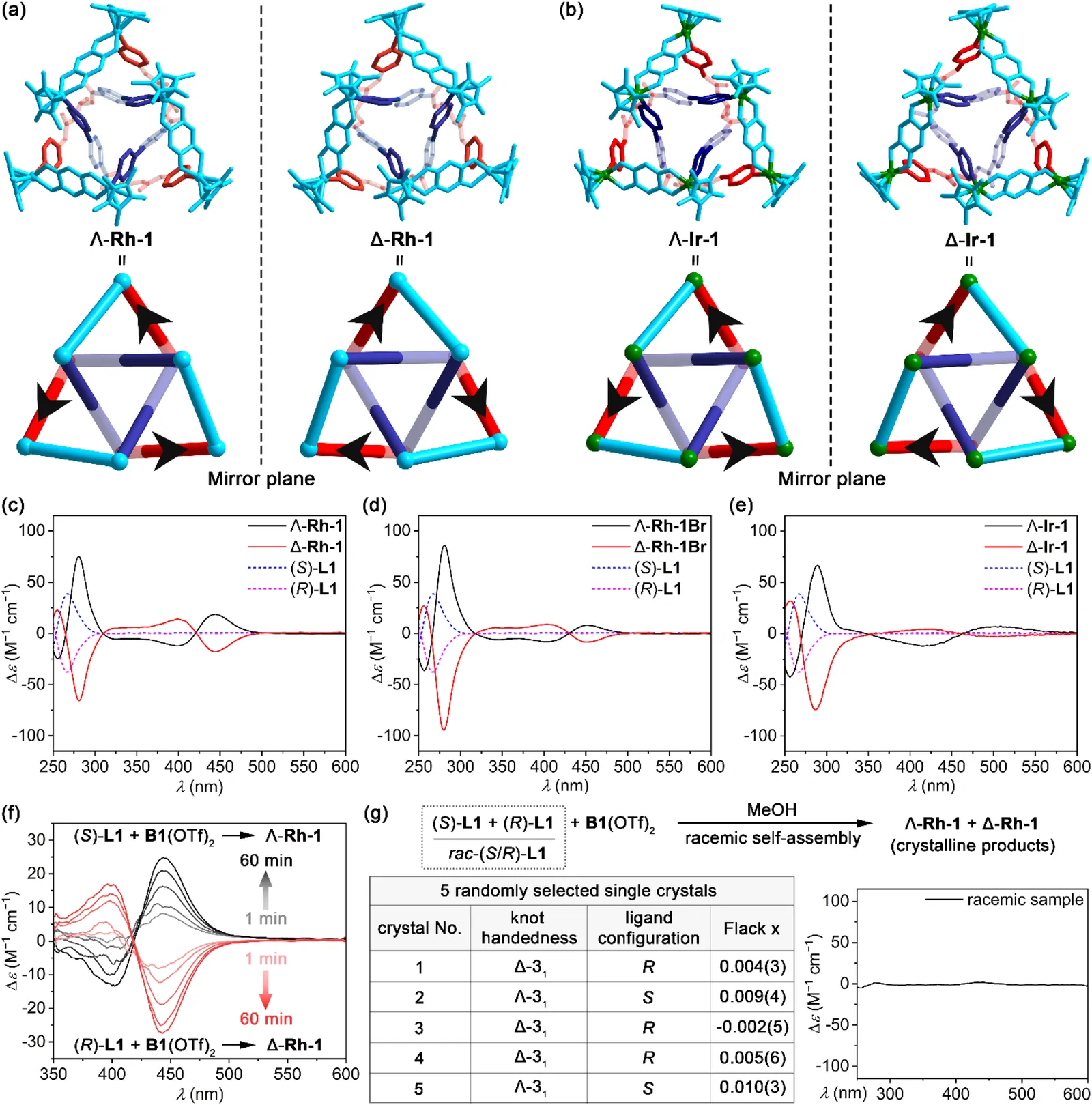
Stereochemical control and chiroptical behavior of the trefoil-knot assemblies. (a) Single-crystal structures and simplified topological representations of Λ-Rh-1 and Δ-Rh-1, showing their mirror-related trefoil-knot architectures. (b) Corresponding single-crystal structures and topological representations of Λ-Ir-1 and Δ-Ir-1. (c–e) CD spectra of Λ-Rh-1/Δ-Rh-1, Λ-Rh-1Br/Δ-Rh-1Br, and Λ-Ir-1/Δ-Ir-1, respectively, recorded in methanol at room temperature (knot concentration: 0.8 mM; optical path length: 0.1 mm). Free (*S*)-L1 and (*R*)-L1 references (2.4 mM) are plotted as 3 Δ*ε*_ligand_ to match the three ligand units in each knot. (f) Time-dependent CD spectra recorded during self-assembly of (*S*)-L1 and (*R*)-L1 with B1(OTf)_2_ from 1 to 60 min. Progressive enhancement of oppositely signed, mirror-related CD responses is observed as the two enantiomeric assemblies develop. (g) Self-assembly of *rac*-(*S*/*R*)-L1 with B1(OTf)_2_ and crystallographic analysis of five independently selected single crystals from the resulting crystalline products. Three crystals were identified as Δ-handed trefoil knots composed of (*R*)-L1, whereas two were identified as Λ-handed trefoil knots composed of (*S*)-L1; the corresponding Flack parameters are listed in the table. Each crystallographically examined knot contains ligands of a single configuration. The bulk racemic sample shows no appreciable net CD response in methanol. Counteranions and hydrogen atoms are omitted for clarity. Color code: Rh, turquoise; Ir, green.

Self-assembly with an equimolar mixture of (*S*)-L1 and (*R*)-L1 provided a further test of component selection (Fig. 6g). For the B1(OTf)_2_ system, the ^1^H NMR spectrum matched that of the enantiopure trefoil knots in solution (Fig. S57), while the bulk sample showed no appreciable net CD response in methanol (Fig. 6g and S202). Its UV-vis absorption spectrum also closely matched those of the enantiopure trefoil knots (Fig. S193). The racemic assembly was further characterized by ^1^H DOSY NMR and HR ESI-MS (Fig. S58 and S173). Five independently selected crystals were examined: three contained Δ-handed knots assembled from (*R*)-L1, and two contained Λ-handed knots assembled from (*S*)-L1. Each crystallographically examined knot contains ligands of only one configuration, with Flack parameters close to zero (Fig. 6g and Table S10). Together, the solution and crystallographic data support homochiral self-sorting to give a racemic mixture of Λ-Rh-1 and Δ-Rh-1. Thus, the ligand design supports homochiral component selection in addition to control of ligand orientation and knot handedness.

To examine how the ligand structure contributes to the assembly outcome, we compared (*S*)-L1 with four related ligands (Fig. 7). Retaining the chiral β-homoalanine-derived segment while reducing the differentiation between the coordination termini in (*S*)-L2 led to D-shaped metallacycles with B1(OTf)_2_ and B3(OTf)_2_ (Rh-2*S* and Ir-2*S*; Fig. 7b, S11 and S12). Their solution-state characterization is consistent with retention of the D-shaped assemblies (Fig. S106–S119, S182 and S183). Thus, the same amino-acid stereocenter does not by itself specify the trefoil topology. The achiral β-amino-acid analogue L3, containing a gem-dimethyl-substituted amino-acid segment, gave non-interlocked nested dimers of tetranuclear metallomacrocycles with the same two building blocks (Rh-3 and Ir-3; Fig. 7c, S13 and S16). In solution, resonances assigned to the nested dimers coexist with those of individual tetranuclear metallomacrocycles, consistent with partial dissociation of the dimers (Fig. S120, S122, S124, S128, S130, S184 and S185).

**Fig. 7:**
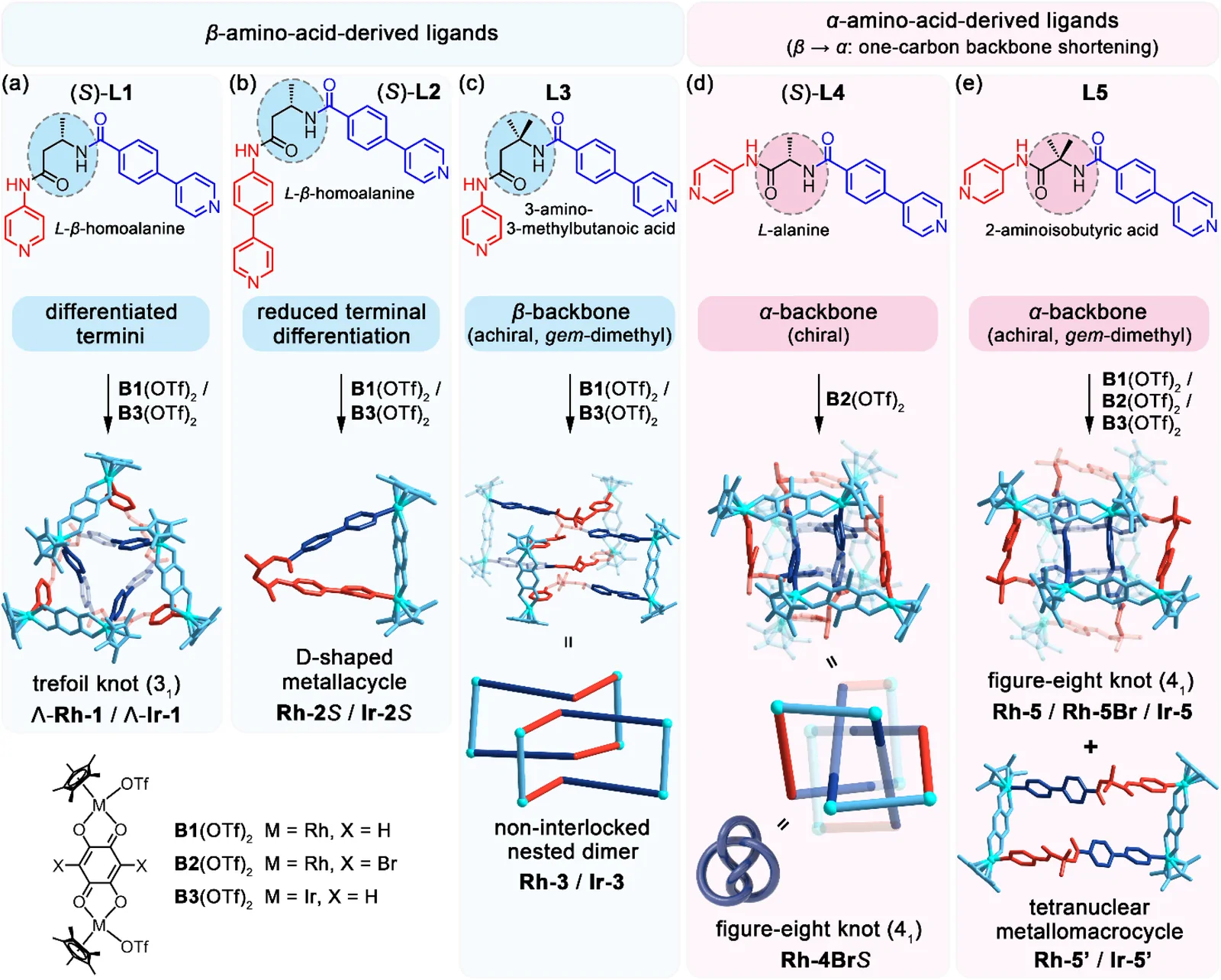
Ligand-structure-dependent assembly outcomes established by single-crystal X-ray diffraction. (a) The original chiral β-homoalanine-derived ligand (*S*)-L1, bearing constitutionally differentiated coordination termini, affords *C*_3_-symmetric Λ-handed 3_1_ knots with B1(OTf)_2_ and B3(OTf)_2_. (b) Retaining the same β-homoalanine-derived segment while reducing the differentiation between the coordination termini in (*S*)-L2 gives D-shaped metallacycles with B1(OTf)_2_ and B3(OTf)_2_. (c) The achiral β-amino-acid analogue L3, containing a 3-amino-3-methylbutanoic-acid-derived segment, affords non-interlocked nested dimers composed of two tetranuclear metallomacrocycles. (d) Incorporation of the shorter, chiral l-alanine-derived α-amino-acid segment in (*S*)-L4 gives a crystallographically characterized figure-eight knot (4_1_ knot) with B2(OTf)_2_. (e) The achiral 2-aminoisobutyric-acid-derived ligand L5 affords crystallographically characterized figure-eight knots with B1(OTf)_2_–B3(OTf)_2_ (Rh-5, Rh-5Br and Ir-5); tetranuclear metallomacrocycles were also characterized crystallographically with B1(OTf)_2_ and B3(OTf)_2_ (Rh-5′ and Ir-5′). Counteranions and hydrogen atoms are omitted for clarity. Only crystallographically characterized assemblies are shown; additional solution-state observations are provided in the SI.

Shortening the amino-acid segment by one carbon also changed the topology. The l-alanine-derived ligand (*S*)-L4 afforded a crystallographically characterized figure-eight knot (4_1_ knot), Rh-4Br*S*, with B2(OTf)_2_ (Fig. 7d and S19); 4_1_ denotes the four-crossing figure-eight knot.^47^ With the achiral α-amino-acid-derived ligand L5, figure-eight knots were characterized crystallographically for all three building blocks, whereas tetranuclear metallomacrocycles were additionally characterized only for B1(OTf)_2_ and B3(OTf)_2_ (Fig. 7e, S22, S23, S26, S29 and S30). In solution, NMR spectra reveal coexistence of knots and tetranuclear metallomacrocycles for the (*S*)-L4/B2(OTf)_2_ and all three L5-derived systems (Fig. S135–S165 and S186–S191). These results support a combined role for terminal differentiation and the geometry of the amino-acid segment in directing trefoil-knot assembly, with ligand point chirality biasing its handedness. Because the control ligands also differ in steric demand and conformational preferences, the comparisons do not isolate any one of these features as the sole determinant.

## Conclusions

In summary, we have developed a ligand-directed strategy for the stereoselective self-assembly of *C*_3_-symmetric topologically chiral trefoil knots. The key to this design is an oriented chiral bis-pyridyl ligand that combines intrinsic N-to-C directionality, inequivalent coordination termini, conformational adaptability and an amino-acid stereocenter. This ligand design enables access to symmetry-reduced trefoil-knot frameworks while simultaneously transferring local point chirality into global topological handedness. Self-assembly of (*S*)-L1 with three related binuclear half-sandwich Rh/Ir organometallic building blocks affords Λ-handed trefoil knots Λ-Rh-1, Λ-Rh-1Br and Λ-Ir-1, whereas its enantiomer (*R*)-L1 gives the corresponding Δ-handed knots Δ-Rh-1, Δ-Rh-1Br and Δ-Ir-1. Crystallographic analysis, NMR spectroscopy, HR ESI-MS and mirror-image CD spectra collectively support the knotted structural assignments and enantiomeric relationships. Comparison with the free ligands reveals enhanced CD responses and additional bands upon assembly. Time-dependent spectroscopy shows knot formation in solution before isolation. Stability studies support the persistence of Λ-Rh-1 and Λ-Ir-1 under the conditions examined, whereas Λ-Rh-1Br shows greater sensitivity to dilution and heating. Assembly from an equimolar mixture of (*S*)-L1 and (*R*)-L1 further supports homochiral self-sorting into a racemic mixture of trefoil knots. The ligand comparisons show that retaining the amino-acid stereocenter alone does not preserve the trefoil topology, supporting the combined importance of terminal differentiation and the geometry of the amino-acid segment. This work demonstrates how ligand orientation and point chirality can be integrated within a single ligand design to select both the symmetry and handedness of molecular trefoil knots.

## Author contributions

Zheng Cui: conceptualization, methodology, supervision, formal analysis, writing – original draft, writing – review & editing. Junli Guo: investigation, formal analysis, visualization, writing – original draft. Liyan Hao: investigation, formal analysis, visualization. Huiyong Wang: supervision, writing – review & editing. Jianji Wang: supervision, writing – review & editing. All authors discussed the results and approved the final version of the manuscript.

## Conflicts of interest

There are no conflicts to declare.

## Supplementary Material

SC-OLF-D6SC05627J-s001

## Data Availability

CCDC 2566605 (for Λ-Rh-1), 2566606 (for Δ-Rh-1), 2566607 (for Δ-Ir-1), 2566608 (for Λ-Ir-1), 2592947 (for Rh-2*S*), 2592948 (for Ir-2*S*), 2592949 (for Rh-3), 2592950 (for Ir-3), 2592951 (for Rh-4Br*S*), 2592952 (for Rh-5), 2592953 (for Rh-5′), 2592954 (for Rh-5Br), 2592955 (for Ir-5), 2592956 (for Ir-5′), 2592957 (for B1(OTf)_2_), 2592958 (for B2(OTf)_2_), 2592959 (for B3(OTf)_2_), 2592960 (for crystal 1 from the racemic self-assembly experiment), 2592961 (for crystal 2 from the racemic self-assembly experiment), 2592962 (for crystal 3 from the racemic self-assembly experiment), 2592963 (for crystal 4 from the racemic self-assembly experiment) and 2592964 (for crystal 5 from the racemic self-assembly experiment) contain the supplementary crystallographic data for this paper.^50*a*–*v*^ The data supporting this article have been included as part of the supplementary information (SI). Supplementary information: experimental procedures, synthetic details, molecular structures, NMR spectra, ESI-MS spectra, UV-vis and CD spectra, and X-ray crystallographic details. See DOI: https://doi.org/10.1039/d6sc05627j.
