# Supplementary material for: Ligand-directed symmetry reduction and stereoselective assembly of C3-symmetric topologically chiral trefoil knots
Source: Chem Sci. 2026 Oct 23. Online ahead of print. doi: 10.1039/d6sc05627j (PMC13716131; doi:10.1039/d6sc05627j)
Supplement: Supplementary file 1 [file SC-OLF-D6SC05627J-s001.pdf]

Supplementary Information (SI) for Chemical Science.
This journal is © The Royal Society of Chemistry

Supplementary Information

Ligand-Directed Symmetry Reduction and Stereoselective Assembly of C_3-Symmetric Topologically Chiral Trefoil Knots

Zheng Cui,* Jun-Li Guo, Li-Yan Hao, Hui-Yong Wang* and Jian-Ji Wang*

Key Laboratory of Green Chemical Media and Reactions (Ministry of Education), Collaborative Innovation Center of Henan Province for Green Manufacturing of Fine Chemicals, School of Chemistry and Chemical Engineering, Henan Normal University, Xinxiang, Henan 453007, P. R. China.

*E-mail: cuizheng@htu.edu.cn

hywang@htu.edu.cn

jwang@htu.edu.cn

Table of Contents

1. Materials and Methods S3

2. Synthesis of Ligands S4

3. Synthesis of Binuclear Building Blocks S14

4. Synthesis of Coordination Assemblies S16

5. Molecular Structures S41

6. NMR Spectra S55

7. ESI-MS Spectra S163

8. UV-Vis and CD Spectra S175

9. X-ray Crystallography Details S187

10. References S205

1. Materials and Methods

All chemical reagents were purchased from commercial companies and used as obtained unless otherwise mentioned. NMR spectra were recorded on a Bruker AVANCE NEO 400 MHz spectrometer or Bruker AVANCE III HD 600 MHz spectrometer at room temperature unless otherwise specified. Both proton and carbon chemical shifts are reported relative to the residual solvent peak (δH = 3.31 ppm for CD_3OD, δH = 2.50 ppm for DMSO-d_6, δC = 49.00 ppm for CD_3OD, δC = 39.52 ppm for DMSO-d_6). Coupling constants (J) are expressed in hertz (Hz). ESI-MS spectra were carried out on a Micro TOF II mass spectrometer or an Agilent UHPLC–QTOF system using electrospray ionization. Chiral supercritical fluid chromatography (SFC) was performed using a Waters ACQUITY UPC^2 system equipped with a photodiode array (PDA) detector. UV-Vis absorption spectra were measured on a PerkinElmer Lambda 35 UV-Vis spectrophotometer. Circular dichroism (CD) spectra were collected on a Chirascan spectrometer.

Unless otherwise specified, c denotes the molar concentration of complete trefoil-knot assemblies. The symbols c_{Rh}, c_{Ir}, and c_{ligand} denote the total molar concentrations of Rh, Ir, and ligand, respectively.

UV–vis spectra are expressed as molar absorption coefficients, $\varepsilon = A/(cl)$, and CD spectra as molar circular dichroism, $\Delta\varepsilon = \theta/(32980cl)$, where A is absorbance, θ is ellipticity in millidegrees, c is concentration in mol L^{-1}, and l is optical path length in cm. Absorption dissymmetry factors were calculated as $g_{abs} = \Delta\varepsilon/\varepsilon$.

2. Synthesis of Ligands

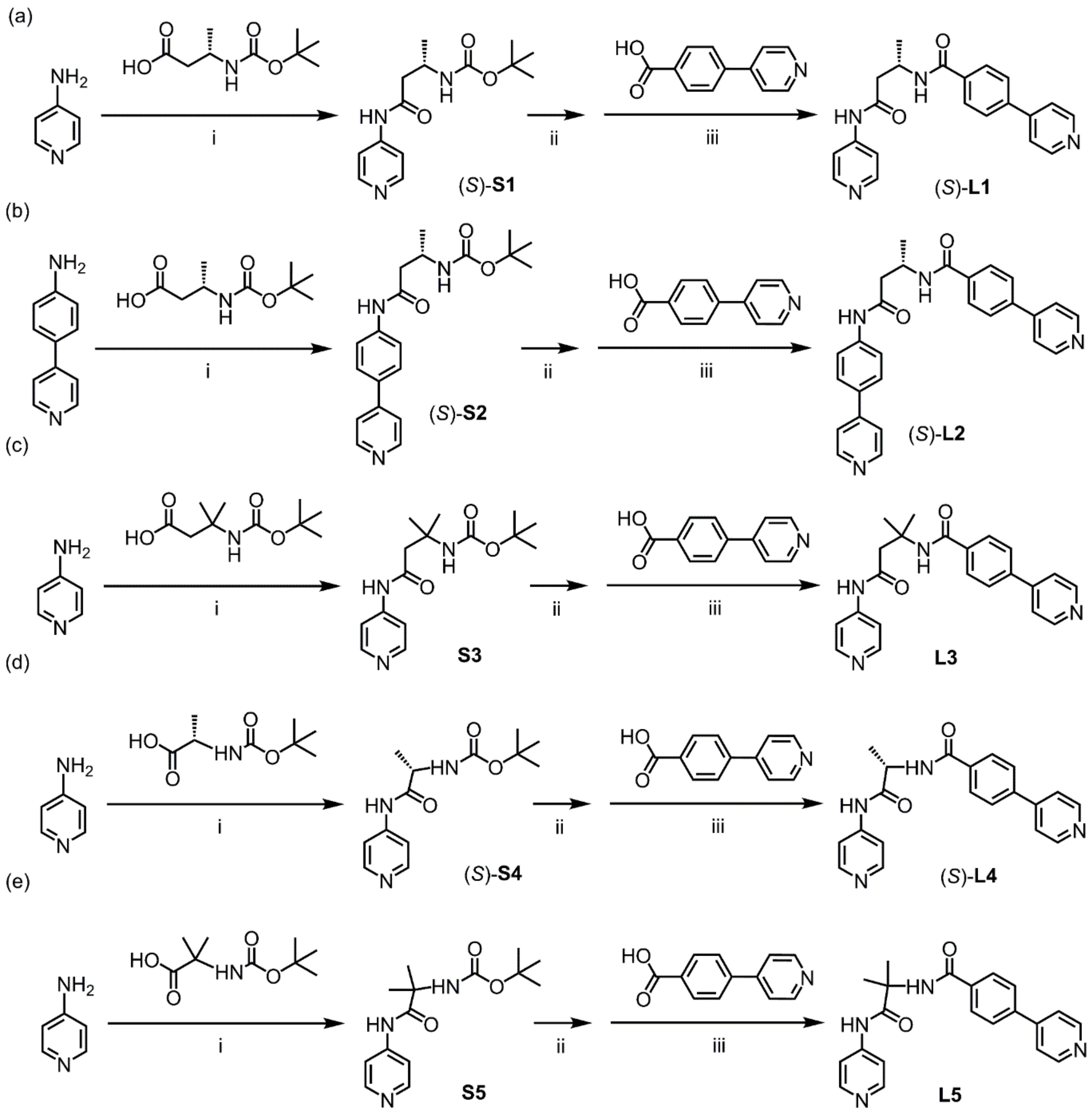

Fig. S1. Synthetic routes to (a) (*S*)-**L1**, (b) (*S*)-**L2**, (c) **L3**, (d) (*S*)-**L4**, and (e) **L5**. Reagents and conditions: (i) *N*-Methylmorpholine (NMM), isobutyl chloroformate (IBCF), in THF, 0 °C 30 min, then RT overnight; (ii) HCl in 1,4-dioxane, MeOH, RT 3 h; (iii) *N*,*N*-diisopropylethylamine (DIEA), 1-hydroxybenzotriazole (HOBt), 1-(3-dimethylaminopropyl)-3-ethylcarbodiimide hydrochloride (EDCl), in DMF, 35 °C 3 d.

Synthesis of (*S*)-S1

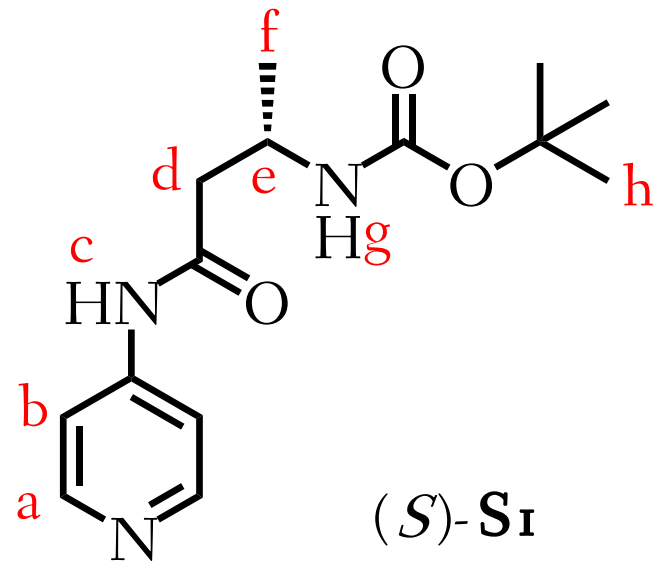

(*S*)-**S1**

The anhydrous THF (25mL) solution of Boc-*L*-β-homoalanine (6.0 mmol, 1.220g) and N-methylmorpholine (NMM, 6.0 mmol, 0.66mL) was cooled to 0 °C, and then isobutyl chloroformate (IBCF, 6.0 mmol, 0.78mL) was slowly added. The mixture was stirred at 0 °C for 30 min, and then 4-Aminopyridine (6.0 mmol, 0.570 g) was added, followed by stirring at 0 °C for 1 h and room temperature overnight. After removing the solvent by evaporation, the obtained solid was washed with an appropriate amount of $CHCl_3$ and EtOAc, and then dried under vacuum to obtain the white product (*S*)-**S1** (0.754 g, 45%). ^{1}H NMR (400 MHz, DMSO-d_6, ppm): δ = 10.26 (s, 1H, H_c), 8.45 – 8.36 (m, 2H, H_a), 7.58 – 7.50 (m, 2H, H_b), 6.84 (d, *J* = 8.2 Hz, 1H, H_g), 3.94 – 3.87 (m, 1H, H_e), 2.54 – 2.51 (m, 1H, H_d), 2.42 – 2.37 (m, 1H, H_d), 1.35 (s, 9H, H_h), 1.07 (d, *J* = 6.6 Hz, 3H, H_f).

Synthesis of (*S*)-L1

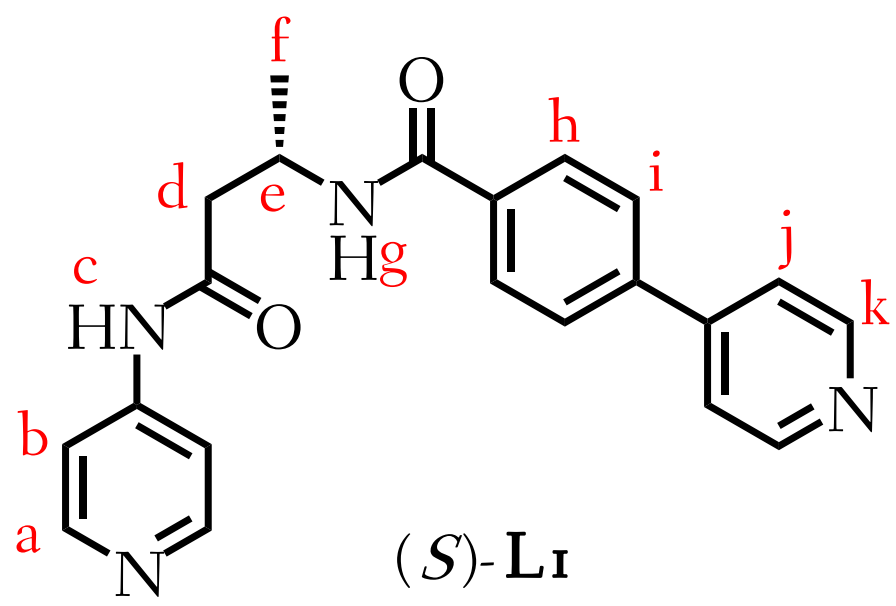

(*S*)-**L1**

Hydrogen chloride-1,4-Dioxane solution (4.0 mol L^{-1}, 3 mL) was slowly added to the dry MeOH (4 mL) solution of (*S*)-**S1** (1.0 mmol, 0.279 g). After stirring for 3 h at room temperature, the solvent was removed by evaporation. The obtained solid (de-boc (*S*)-**S1**, 1.0 mmol) was directly dissolved in anhydrous DMF

(*N*,*N*-dimethylformamide, 20 mL) after vacuum drying, and *N*,*N*-diisopropylethylamine (DIEA, 4.7 mmol, 0.81 mL), 1-hydroxybenzotriazole (HOBt, 1.72 mmol, 0.232 g), 1-(3-dimethylaminopropyl)-3-ethylcarbodiimide hydrochloride (EDCl, 1.72 mmol, 0.329 g), and 4-pyridin-4-yl-benzoic acid (1.0 mmol, 0.199 g) were added to the above solution. The mixture was stirred at 35 °C for 3 days and then evaporated to remove the solvent. The obtained orange oily product was washed with saturated aqueous solution of $NaHCO_3$, distilled water and EtOAc, and then dried under vacuum to obtain the white solid product (*S*)-**L1** (0.324 g, 90%). ESI-MS *m/z*: 361.1661 (calcd for $[(S)\text{-}\mathbf{L1} + H]^+$: 361.1659). ^1H NMR (400 MHz, DMSO-d_6, ppm): δ= 10.34 (s, 1H, H_c), 8.70 – 8.64 (m, 2H, H_k), 8.48 (d, *J* = 8.0 Hz, 1H, H_g), 8.43 – 8.37 (m, 2H, H_a), 7.96 (d, *J* = 8.5 Hz, 2H, H_h), 7.91 (d, *J* = 8.2 Hz, 2H, H_i), 7.80 – 7.74 (m, 2H, H_j), 7.59 – 7.53 (m, 2H, H_b), 4.47 (p, *J* = 6.9 Hz, 1H, H_e), 2.72 (dd, *J* = 14.6, 6.6 Hz, 1H, H_d), 2.60 (dd, *J* = 14.7, 7.3 Hz, 1H, H_d), 1.25 (d, *J* = 6.7 Hz, 3H, H_f). ^{13}C NMR (151 MHz, DMSO-d_6, ppm): δ= 170.40, 165.04, 150.34, 146.07, 145.67, 139.56, 135.18, 128.13, 126.70, 121.35, 113.18, 43.18, 42.84, 20.31.

Synthesis of (*R*)-S1

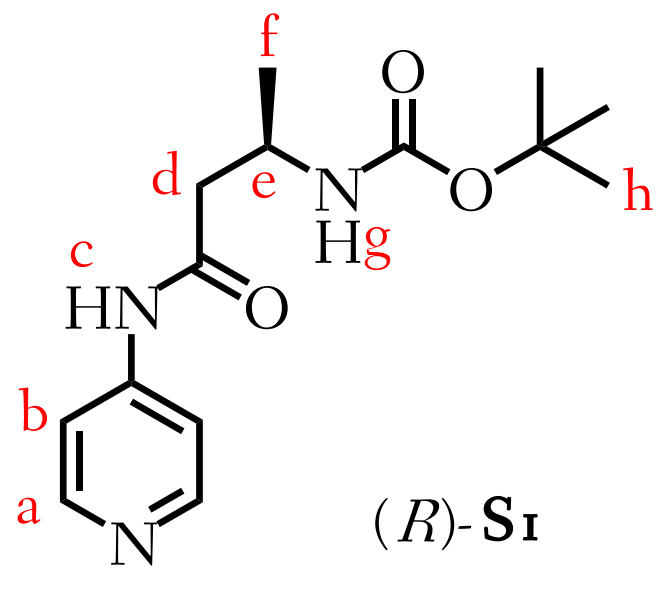

(*R*)-**S1**

(*R*)-**S1** (0.820 g, 49%) was synthesized by following the same procedure of (*S*)-**S1**, except that Boc-*L*-β-homoalanine was replaced by Boc-*D*-β-homoalanine (6.0 mmol, 1.220 g). ^1H NMR (400 MHz, DMSO-d_6, ppm): δ= 10.26 (s, 1H, H_c), 8.42 – 8.37 (m, 2H, H_a), 7.57 – 7.48 (m, 2H, H_b), 6.83 (d, *J* = 8.2 Hz, 1H, H_g), 3.94 – 3.87 (m, 1H, H_e), 2.54 – 2.51 (m, 1H, H_d), 2.42 – 2.37 (m, 1H, H_d), 1.35 (s, 9H, H_h), 1.07 (d, *J* = 6.6 Hz, 3H, H_f).

Synthesis of (*R*)-L1

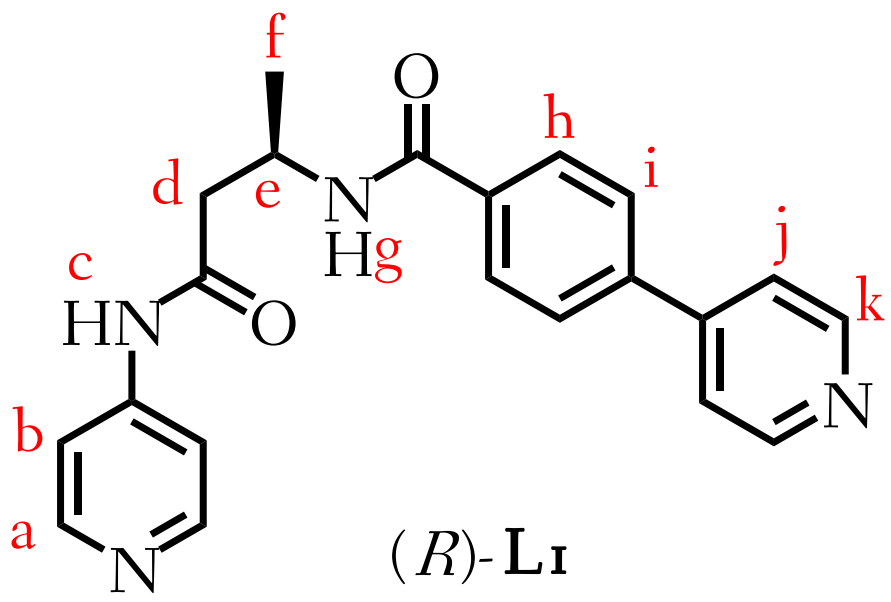

(*R*)- **L1**

(*R*)-**L1** (0.299 g, 83%) was synthesized by following the same procedure of (*S*)-**L1**, except that (*S*)-**S1** was replaced by (*R*)-**S1** (1.0 mmol, 0.279 g). ESI-MS *m/z*: 361.1663 (calcd for [(*R*)-**L1** + H]$^+$: 361.1659). ^{1}H NMR (400 MHz, DMSO-d$_6$, ppm): δ = 10.34 (s, 1H, H$_c$), 8.70 – 8.64 (m, 2H, H$_k$), 8.49 (d, *J* = 8.0 Hz, 1H, H$_g$), 8.42 – 8.37 (m, 2H, H$_a$), 7.97 (d, *J* = 8.2 Hz, 2H, H$_h$), 7.91 (d, *J* = 8.2 Hz, 2H, H$_i$), 7.80 – 7.74 (m, 2H, H$_j$), 7.59 – 7.54 (m, 2H, H$_b$), 4.47 (p, *J* = 6.9 Hz, 1H, H$_e$), 2.74 – 2.69 (m, 1H, H$_d$), 2.61 (dd, *J* = 14.6, 7.2 Hz, 1H, H$_d$), 1.25 (d, *J* = 6.7 Hz, 3H, H$_f$). ^{13}C NMR (151 MHz, DMSO-d$_6$, ppm): δ = 170.45, 165.03, 150.34, 146.07, 145.70, 139.56, 135.17, 128.14, 126.70, 121.35, 113.19, 43.17, 42.89, 20.36.

Synthesis of (*S*)-S2

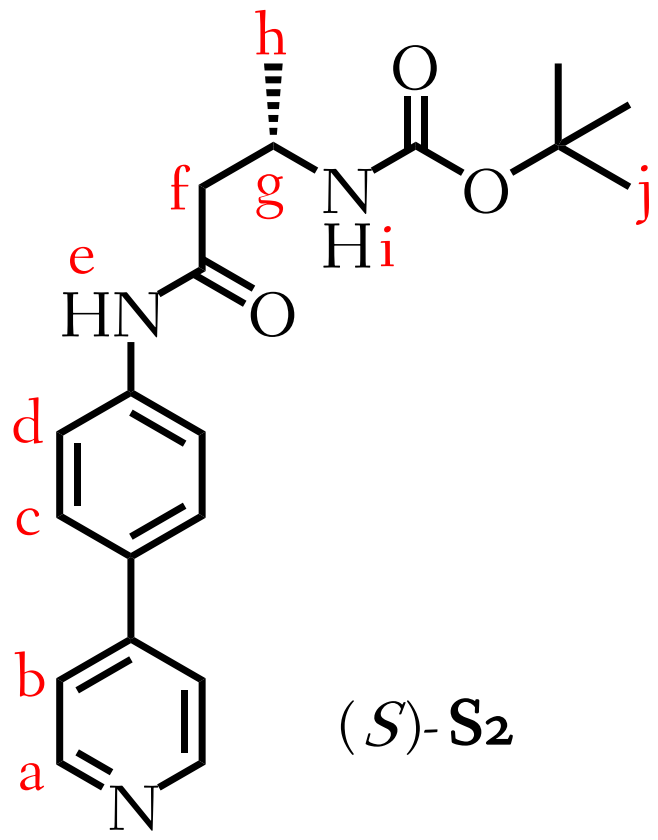

(*S*)- **S2**

(*S*)-**S2** (1.422 g, 66.7%) was synthesized by following the same procedure of (*S*)-**S1**, except that 4-Aminopyridine was replaced by 4-(pyridin-4-yl)aniline (6.0 mmol, 1.020 g). ^{1}H NMR (600 MHz, DMSO-

d$_6$, ppm): δ = 10.11 (s, 1H, H$_e$), 8.62 – 8.56 (m, 2H, H$_a$), 7.79 (d, J = 8.8 Hz, 2H, H$_c$), 7.74 (d, J = 8.8 Hz, 2H, H$_d$), 7.70 – 7.66 (m, 2H, H$_b$), 6.87 (d, J = 8.3 Hz, 1H, H$_i$), 3.97 – 3.85 (m, 1H, H$_g$), 2.54 – 2.52 (m, 1H, H$_f$), 2.40 – 2.37 (m, 1H, H$_f$), 1.37 (s, 9H, H$_j$), 1.09 (d, J = 6.6 Hz, 3H, H$_h$).

Synthesis of (*S*)-L2

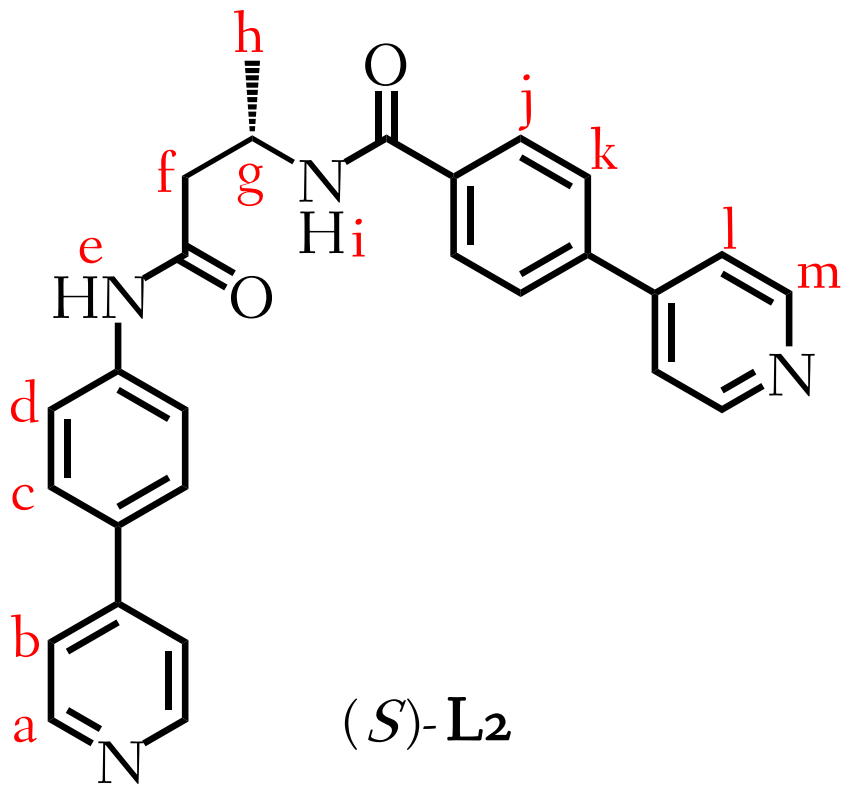

(*S*)-**L2**

(*S*)-**L2** (0.314 g, 72.6%) was synthesized by following the same procedure of (*S*)-**L1**, except that (*S*)-**S1** was replaced by (*S*)-**S2** (1.0 mmol, 0.355 g). ESI-MS *m*/*z*: 437.2003 (calcd for [(*S*)-**L2** + H]$^+$: 437.1972). ^{1}H NMR (600 MHz, DMSO-d$_6$, ppm): δ = 10.19 (s, 1H, H$_e$), 8.71 – 8.64 (m, 2H, H$_a$), 8.61 – 8.54 (m, 2H, H$_m$), 8.51 (d, J = 7.9 Hz, 1H, H$_i$), 8.04 – 7.94 (m, 2H, H$_j$), 7.92 – 7.89 (m, 2H, H$_k$), 7.80 – 7.73 (m, 6H, H$_b$, H$_c$, H$_d$), 7.69 – 7.63 (m, 2H, H$_l$), 4.54 – 4.49 (m, 1H, H$_g$), 2.72 (dd, J = 14.4, 6.6 Hz, 1H, H$_f$), 2.60 (dd, J = 14.4, 7.4 Hz, 1H, H$_f$), 1.27 (d, J = 6.6 Hz, 3H, H$_h$). ^{13}C NMR (151 MHz, DMSO-d$_6$, ppm): δ = 169.41, 164.99, 150.33, 150.17, 146.41, 146.06, 140.29, 139.55, 135.22, 131.41, 128.12, 127.20, 126.70, 121.34, 120.59, 119.54, 43.14, 43.02, 20.30.

Synthesis of S3

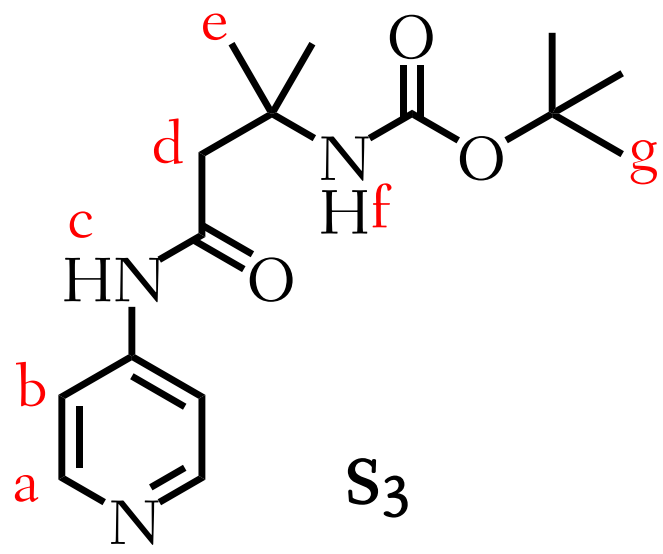

S3

S3 (1.614 g, 91.8%) was synthesized by following the same procedure of (*S*)-**S1**, except that Boc-*L*-β-homoalanine was replaced by N-Boc-3-amino-3-methylbutanoic acid (6.0 mmol, 1.303 g). ^{1}H NMR (600 MHz, DMSO-d_6, ppm): δ = 10.22 (s, 1H, H_c), 8.39 (d, *J* = 5.6 Hz, 2H, H_a), 7.56 (d, *J* = 5.6 Hz, 2H, H_b), 6.60 (s, 1H, H_f), 2.69 (s, 2H, H_d), 1.35 (s, 9H, H_g), 1.31 (s, 6H, H_e).

Synthesis of L3

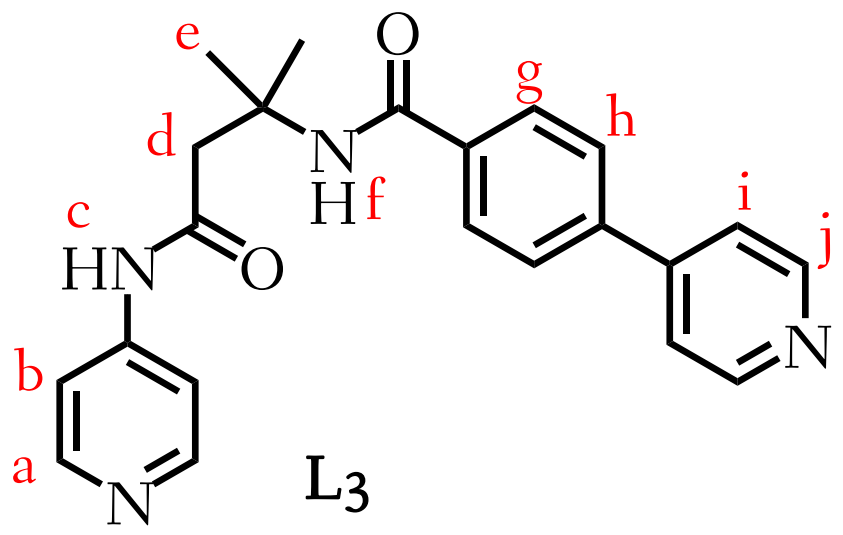

L3

L3 (0.288 g, 76.9%) was synthesized by following the same procedure of (*S*)-**L1**, except that (*S*)-**S1** was replaced by **S3** (1.0 mmol, 0.293 g). ESI-MS *m/z*: 375.1808 (calcd for [**L3** + H]$^{+}$: 375.1816). ^{1}H NMR (400 MHz, DMSO-d_6, ppm): δ = 10.32 (s, 1H, H_c), 8.72 – 8.64 (m, 2H, H_j), 8.42 – 8.37 (m, 2H, H_a), 8.11 (s, 1H, H_f), 7.92 (d, *J* = 8.5 Hz, 2H, H_g), 7.89 (d, *J* = 8.5 Hz, 2H, H_h), 7.78 – 7.74 (m, 2H, H_i), 7.58 – 7.52 (m, 2H, H_b), 2.93 (s, 2H, H_d), 1.51 (s, 6H, H_e). ^{13}C NMR (151 MHz, DMSO-d_6, ppm): δ = 170.53, 165.90, 150.34, 146.10, 145.53, 139.38, 136.21, 128.12, 126.63, 121.33, 113.24, 52.61, 45.69, 26.98.

Synthesis of (*S*)-S4

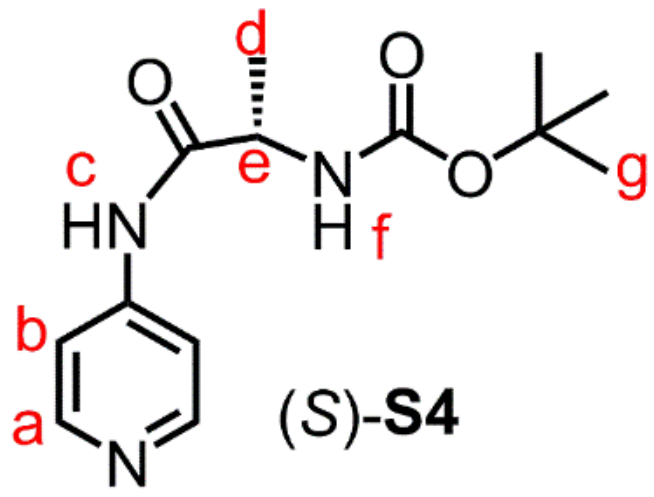

(*S*)-S4

(*S*)-S4 (1.136 g, 71.4%) was synthesized by following the same procedure of (*S*)-**S1**, except that Boc-*L*-α-homoalanine was replaced by Boc-L-Ala-OH (6.0 mmol, 1.135 g). ^{1}H NMR (400 MHz, DMSO-d_6, ppm): δ = 10.32 (s, 1H, H_c), 8.45 – 8.38 (m, 2H, H_a), 7.60 – 7.54 (m, 2H, H_b), 7.18 (d, *J* = 7.0 Hz, 1H, H_f), 4.10 (p, *J* = 7.2 Hz, 1H, H_e), 1.38 (s, 9H, H_g), 1.26 (d, *J* = 7.3 Hz, 3H, H_d).

Synthesis of (*S*)-L4

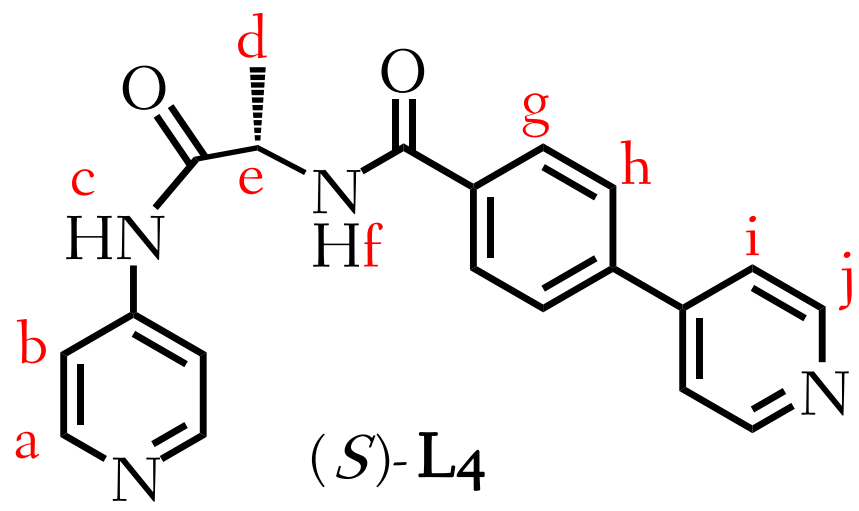

(*S*)-L4

(*S*)-L4 (0.319 g, 92.1%) was synthesized by following the same procedure of (*S*)-**L1**, except that (*S*)-**S1** was replaced by **(*S*)-S4** (1.0 mmol, 0.265 g). ESI-MS *m/z*: 347.1503 (calcd for [**(*S*)-L4** + H]$^+$: 347.1503). ^{1}H NMR (600 MHz, DMSO-d_6, ppm): δ = 10.56 (s, 1H, H_c), 8.90 (s, 1H, H_f), 8.71 – 8.65 (m, 2H, H_j), 8.45 – 8.41 (m, 2H, H_a), 8.10 – 8.06 (m, 2H, H_g), 7.96 – 7.90 (m, 2H, H_h), 7.80 – 7.74 (m, 2H, H_i), 7.63 – 7.60 (m, 2H, H_b), 4.62 (m, 1H, H_e), 1.47 (d, *J* = 7.2 Hz, 3H, H_d). ^{13}C NMR (151 MHz, DMSO-d_6, ppm): δ =

172.75, 165.86, 150.38, 150.36, 146.01, 145.71, 139.86, 134.23, 128.47, 126.71, 121.37, 113.29, 50.28, 17.39.

Synthesis of S5

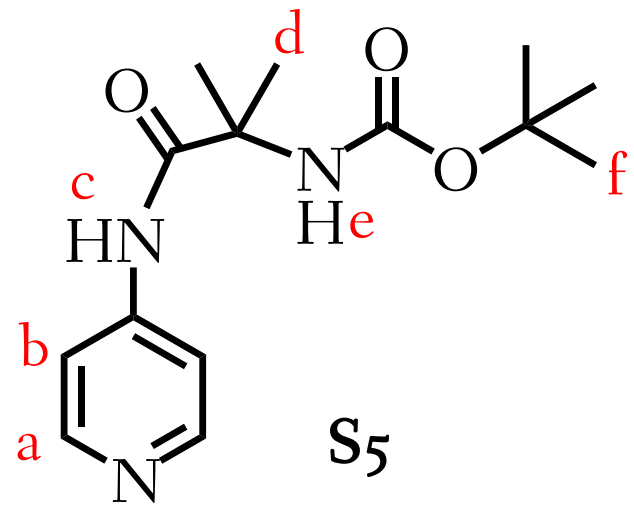

S5

S5 (1.195 g, 71.4%) was synthesized by following the same procedure of (*S*)-**S1**, except that Boc-*L*-β-homoalanine was replaced by Boc-Aib-OH (6.0 mmol, 1.219 g). ^{1}H NMR (600 MHz, DMSO-d_6, ppm): δ = 9.82 (s, 1H, H_c), 8.38 (d, *J* = 5.5 Hz, 2H, H_a), 7.65 (s, 2H, H_b), 7.05 (s, 1H, H_e), 1.36 – 1.33 (br, s, 15H, $H_{f,d}$).

Synthesis of L5

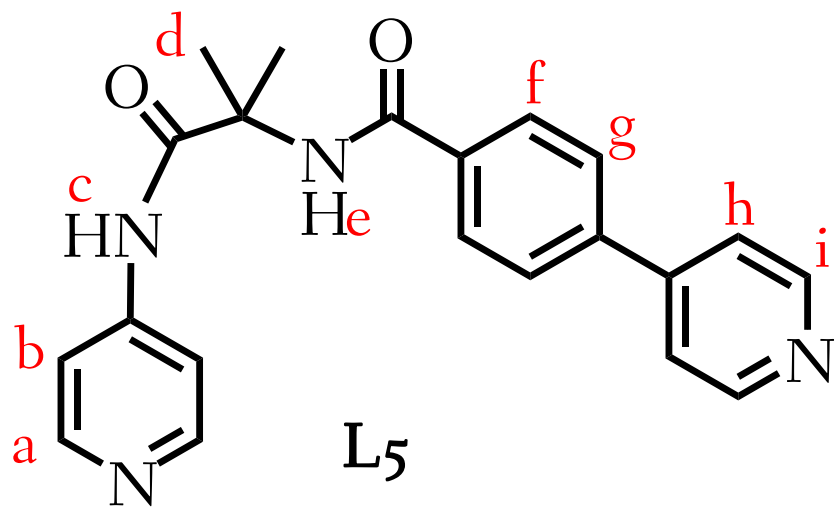

L5

L5 (0.177 g, 49.1%) was synthesized by following the same procedure of (*S*)-**L1**, except that (*S*)-**S1** was replaced by **S5** (1.0 mmol, 0.279 g). ESI-MS *m/z*: 361.1668 (calcd for [**L5** + H]$^+$: 361.1659). ^{1}H NMR (400 MHz, DMSO-d_6, ppm): δ = 9.84 (s, 1H, H_c), 8.69 – 8.64 (m, 2H, H_i), 8.58 (s, 1H, H_e), 8.40 – 8.33 (m, 2H, H_a), 8.18 – 8.04 (m, 2H, H_f), 7.92 (d, *J* = 8.0 Hz, 2H, H_g), 7.80 – 7.77 (m, 2H, H_h), 7.66 – 7.60 (m, 2H, H_b), 1.55 (s, 6H, H_d). ^{13}C NMR (151 MHz, DMSO-d_6, ppm): δ = 173.91, 165.53, 150.35, 150.03, 146.24, 146.05, 139.80, 134.63, 128.61, 126.56, 121.37, 113.64, 57.17, 24.76.

3. Synthesis of Binuclear Building Blocks

The binuclear half-sandwich organometallic complexes **B1**$(OTf)_2$, **B2**$(OTf)_2$, and **B3**$(OTf)_2$ were prepared following the reported procedures.[1-3] The experimental quantities, workup procedures, and characterization data are provided below.

Synthesis of B1$(OTf)_2$

The MeOH (60 mL) solution of $[Cp^*RhCl_2]_2$ (0.185 g, 0.3 mmol) and AgOTf (0.308 g, 1.2 mmol) was stirred for 12 h in the dark, and the AgCl precipitate in the solution was removed by centrifugation. To a MeOH (10 mL) solution of 2,5-dihydroxy-1,4-benzoquinone (0.042 mg, 0.3 mmol) was added 6 mL of a NaOH methanol solution (0.1 mmol mL^{-1}), and after 30 min, the above centrifuged solution was added to continue the reaction for 24 h. After the reaction mixture was filtered through a filter membrane, the filtrate was concentrated to 6 mL by rotary evaporation, and then an appropriate amount of Et₂O was added to precipitate the solid. The solid was collected and dried to obtain the brown product **B1$(OTf)_2$** (0.260 g, 95%). Single crystals of **B1**$(OTf)_2$ suitable for X-ray diffraction were obtained by vapor diffusion of diethyl ether into a methanol solution of the binuclear building block at room temperature (0.254 g, 93% isolated yield). ^1H NMR (400 MHz, CD_3OD, ppm): δ= 5.73 (s, 2H), 1.74 (s, 30H).

Synthesis of B2$(OTf)_2$

B2$(OTf)_2$ (0.302 g, 94%) was synthesized by following the same procedure of **B1$(OTf)_2$**, except that 2,5-dihydroxy-1,4-benzoquinone was replaced by 2,5-Dibromo-3,6-dihydroxycyclohexa-2,5-diene-1,4-dione (0.089 g, 0.3 mmol). Single crystals of **B2**$(OTf)_2$ suitable for X-ray diffraction were obtained by vapor diffusion of diethyl ether into a methanol solution of the binuclear building block at room temperature (0.279 g, 87% isolated yield). ^1H NMR (400 MHz, CD_3OD, ppm): δ= 1.78 (s, 30H).

Synthesis of B3$(OTf)_2$

B3$(OTf)_2$ (0.311 g, 95%) was synthesized by following the same procedure of **B1$(OTf)_2$,** except that $[Cp^*RhCl_2]_2$ was replaced by $[Cp^*IrCl_2]_2$ (0.239 g, 0.3 mmol). Single crystals of **B3**$(OTf)_2$ suitable for X-ray diffraction were obtained by vapor diffusion of diethyl ether into a methanol solution of the binuclear building block at room temperature (0.298 g, 91% isolated yield). ^1H NMR (400 MHz, CD_3OD, ppm): δ = 5.97 (s, 2H), 1.72 (s, 30H).

Single-crystal X-ray diffraction shows an *anti* arrangement of the two Cp* groups in each of the three isolated binuclear precursors.

4. Synthesis of Coordination Assemblies

Synthesis of Λ-Rh-1

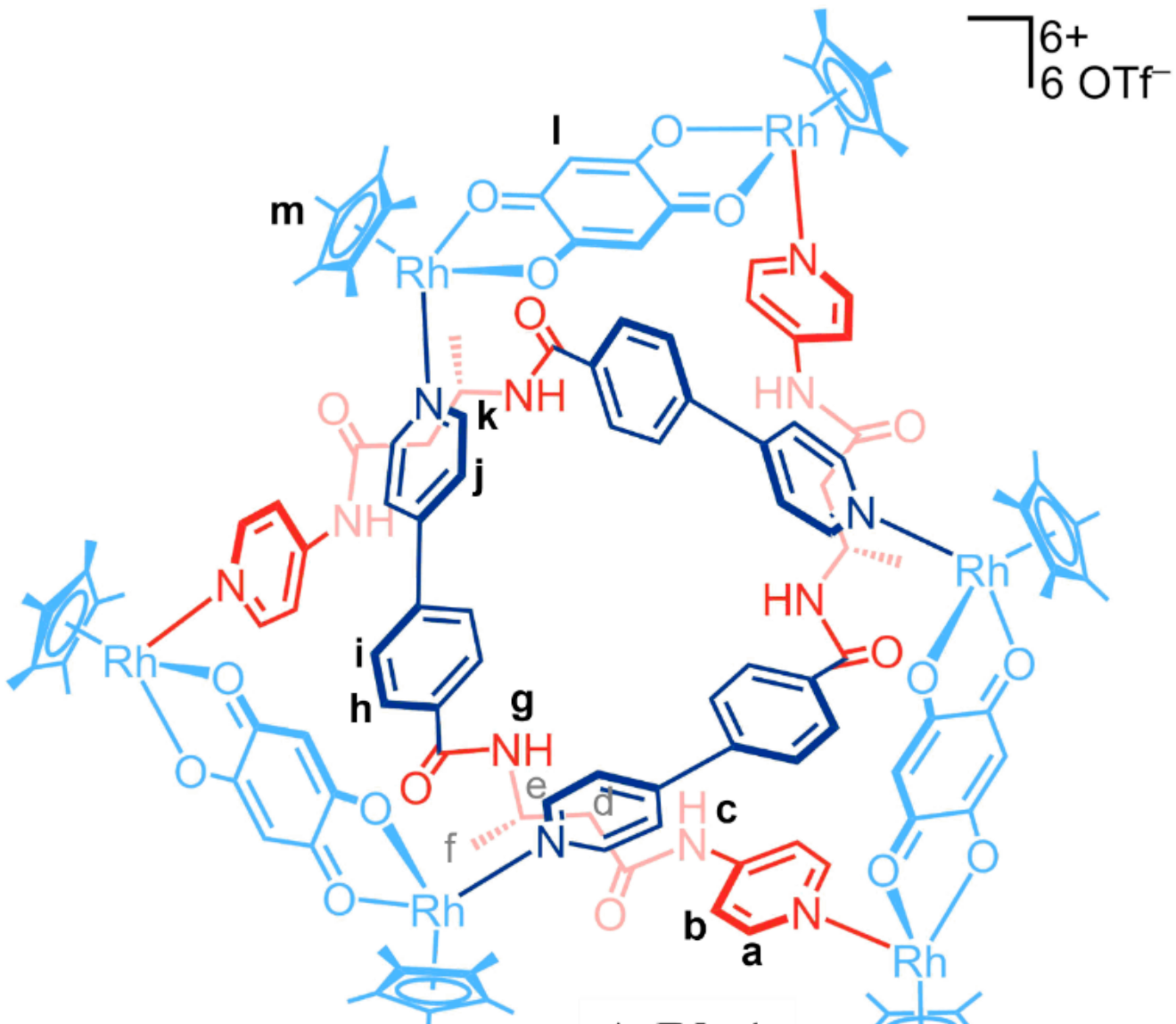

Λ-**Rh-1**

The MeOH (6 mL) solution of ligand (*S*)-**L1** (0.02 mmol, 7.2 mg) and **B1**$(OTf)_2$ (0.02 mmol, 18.2 mg) was stirred at room temperature for 1 day. The reaction mixture was filtered through a filter membrane, and the obtained filtrate was concentrated to 2 mL, and then 6 mL of diethyl ether was added to form orange solid of Λ-**Rh-1**, which was then washed with diethyl ether and dried under vacuum (23.2mg, 91%). Single crystals of Λ-**Rh-1** suitable for X-ray diffraction were obtained by vapor diffusion of diethyl ether into a methanol solution of the assembly at room temperature (21.9mg, 86% isolated yield). ESI-MS *m/z*: 805.3751 (calcd for $[M - 4\ OTf]^{4+}$: 805.3760). ^{1}H NMR (600 MHz, CD_3OD, ppm): δ = 8.55 – 8.52(m, 2H, H_a), 7.53 (d, *J* = 6.5 Hz, 2H, H_k), 7.18 (s, 2H, H_j), 7.05 (s, 2H, H_i), 6.89 – 6.84 (m, 2H, H_b), 6.11 (s, 1H, H_l), 5.55 (d, *J* = 8.4 Hz, 2H, H_h), 5.50 (s, 1H, H_l), 4.96 – 4.93 (m, 1H, H_e), 3.00 – 2.95 (m, 1H, H_d), 2.83 – 2.79 (m, 1H, H_d), 1.77 (s, 15H, H_m), 1.51 (s, 15H, H_m), 1.40 (d, *J* = 6.8 Hz, 3H, H_f). ^{13}C NMR (151 MHz, CD_3OD, ppm): δ = 185.60, 185.32, 184.65, 184.40, 172.49, 166.37, 153.06, 152.01, 151.75,

149.12, 139.87, 135.08, 128.34, 127.03, 126.39, 122.97, 120.86, 116.05, 104.13, 102.55, 97.25, 96.76, 43.63, 42.63, 21.24, 8.88, 8.53.

Synthesis of Δ-Rh-1

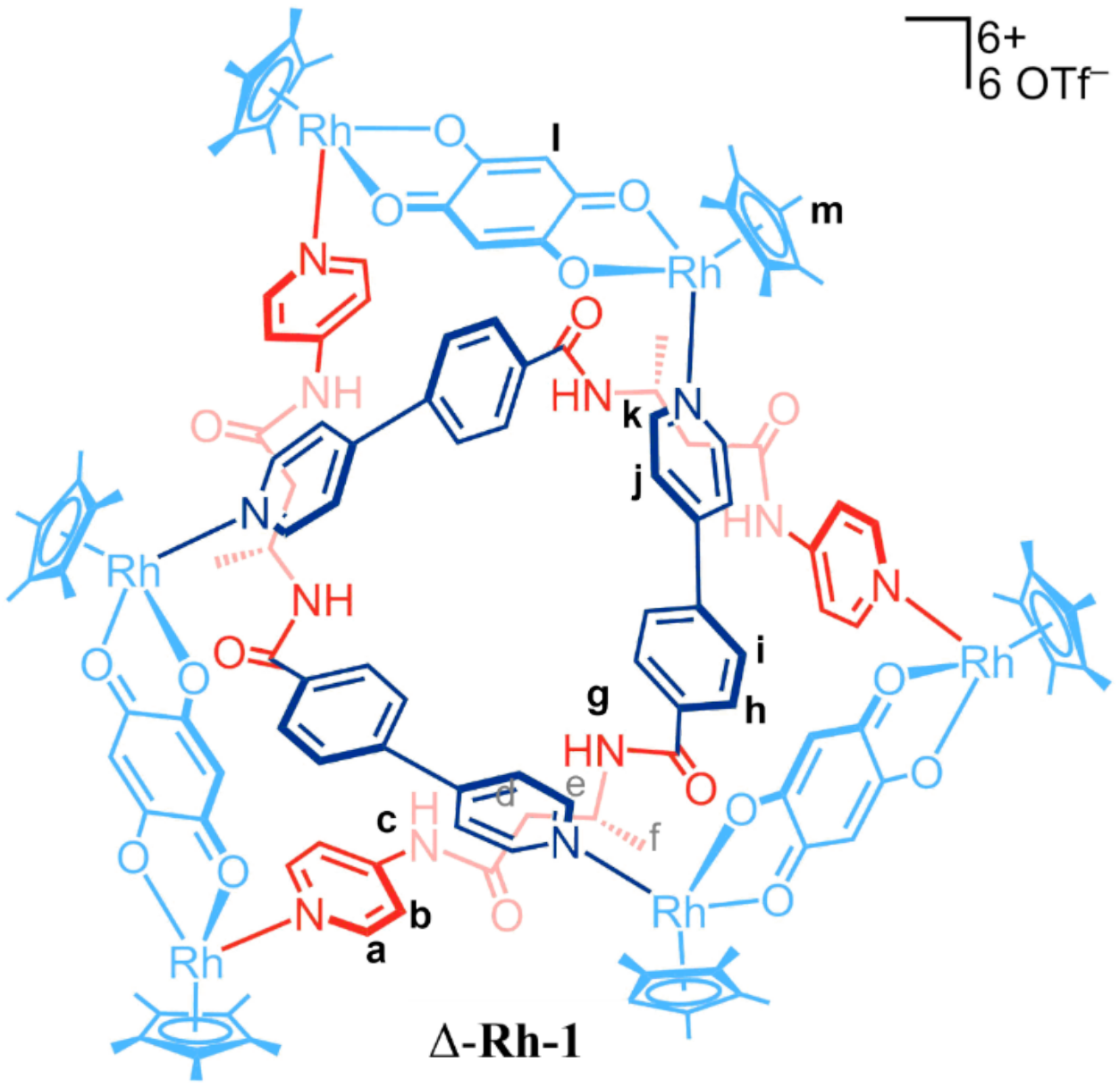

Δ-Rh-1

Δ-**Rh-1** (23.9 mg, 94%) was synthesized by following the same procedure of Λ-**Rh-1**, except that (*S*)-**L1** was replaced by (*R*)-**L1** (0.02 mmol, 7.2 mg). Single crystals of Δ-**Rh-1** suitable for X-ray diffraction were obtained by vapor diffusion of diethyl ether into a methanol solution of the assembly at room temperature (21.6mg, 85% isolated yield). ESI-MS *m*/*z*: 805.3760 (calcd for $[M - 4\ \mathrm{OTf}]^{4+}$: 805.3760). ^{1}H NMR (600 MHz, CD_3OD, ppm): δ = 8.55 – 8.52 (m, 2H, H_a), 7.53 (d, *J* = 6.5 Hz, 2H, H_k), 7.18 (s, 2H, H_j), 7.05 (s, 2H, H_i), 6.89 – 6.84 (m, 2H, H_b), 6.11 (s, 1H, H_l), 5.55 (d, *J* = 8.4 Hz, 2H, H_h), 5.50 (s, 1H, H_l), 4.97 – 4.93 (m, 1H, H_e), 2.98 – 2.95 (m, 1H, H_d), 2.82 – 2.79 (m, 1H, H_d), 1.77 (s, 15H, H_m), 1.51 (s, 15H, H_m), 1.40 (d, *J* = 6.8 Hz, 3H, H_f). ^{13}C NMR (151 MHz, CD_3OD, ppm): δ = 185.59, 185.31, 184.64, 184.39, 172.49, 166.37, 153.06, 152.02, 151.75, 149.12, 139.87, 135.11, 128.35, 127.03, 126.39, 122.99, 120.88, 116.05, 104.13, 102.55, 97.25, 96.77, 43.63, 42.63, 21.24, 8.88, 8.53.

Synthesis of Λ-Rh-1Br

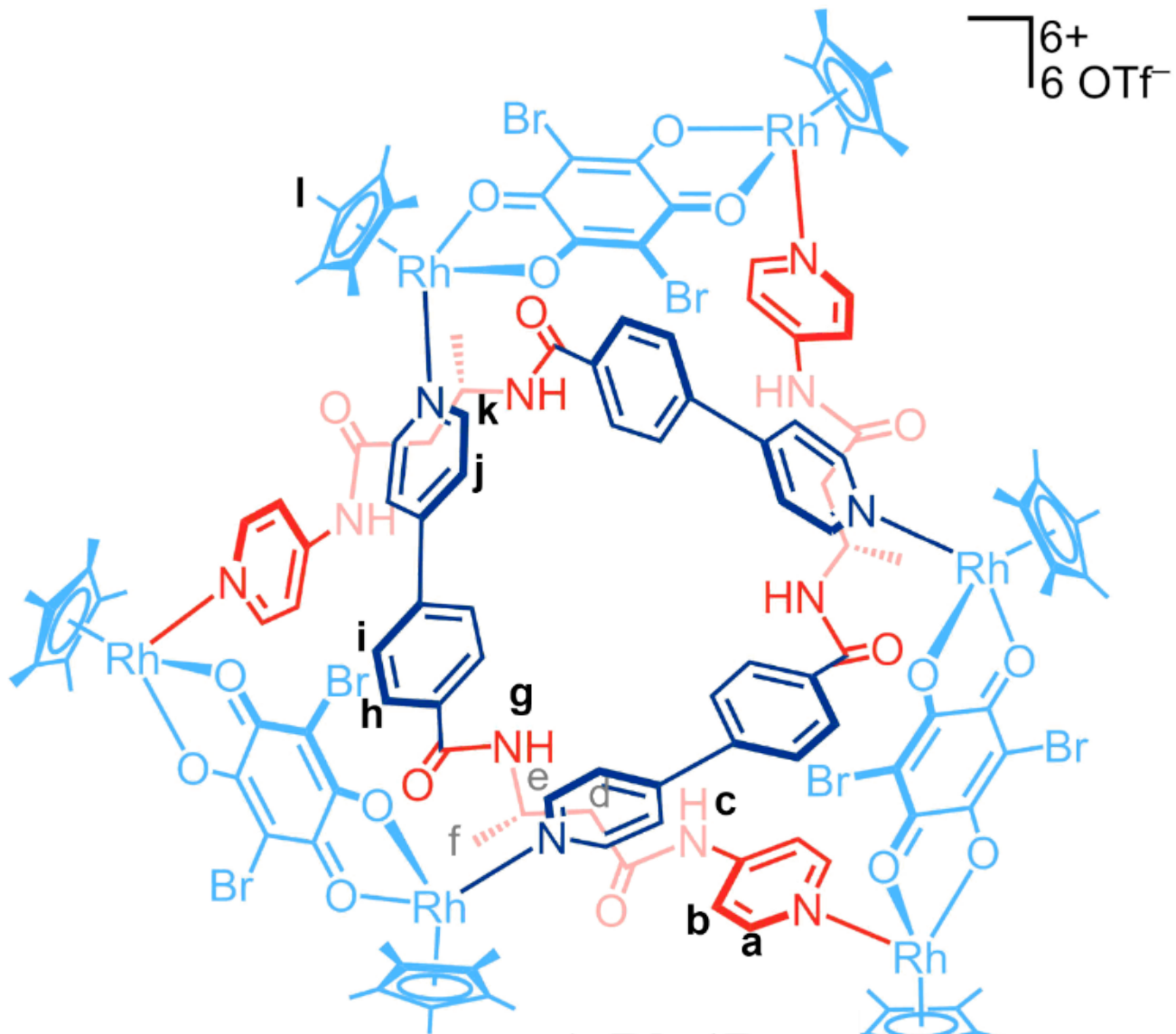

Λ-**Rh-1Br**

The MeOH (6 mL) solution of ligand (*S*)-**L1** (0.02 mmol, 7.2 mg) and **B2**(OTf)$_2$ (0.02 mmol, 21.4 mg) was stirred at room temperature for 1 day. The reaction mixture was filtered through a filter membrane, and the obtained filtrate was concentrated to 2 mL, and then 6 mL of diethyl ether was added to form orange solid of Λ-**Rh-1Br**, which was then washed with diethyl ether and dried under vacuum (27.2mg, 95%). ESI-MS *m*/*z*: 923.9911 (calcd for $[M - 4\ \mathrm{OTf}]^{4+}$: 923.9906). ^{1}H NMR (600 MHz, CD_3OD, ppm): δ = 8.54 (d, *J* = 5.7 Hz, 2H, H_a), 7.57 (d, *J* = 6.5 Hz, 2H, H_k), 7.21 (s, 2H, H_j), 7.05 (d, *J* = 6.1 Hz, 2H, H_b), 6.91 (s, 2H, H_i), 5.46 (d, *J* = 8.3 Hz, 2H, H_h), 4.94 – 4.89 (m, 1H, H_e), 3.02 – 2.97 (m, 1H, H_d), 2.79 – 2.76 (m, 1H, H_d), 1.77 (s, 15H, H_l), 1.56 (s, 15H, H_l), 1.37 (d, *J* = 6.9 Hz, 3H, H_f). ^{13}C NMR (151 MHz, CD_3OD, ppm): δ = 172.56, 165.85, 152.82, 151.91, 149.28, 139.80, 135.20, 129.45, 127.96, 126.86, 125.03, 122.92, 120.81, 118.70, 116.09, 97.86, 97.47, 43.54, 42.49, 21.42, 8.97, 8.61.

Synthesis of Δ-Rh-1Br

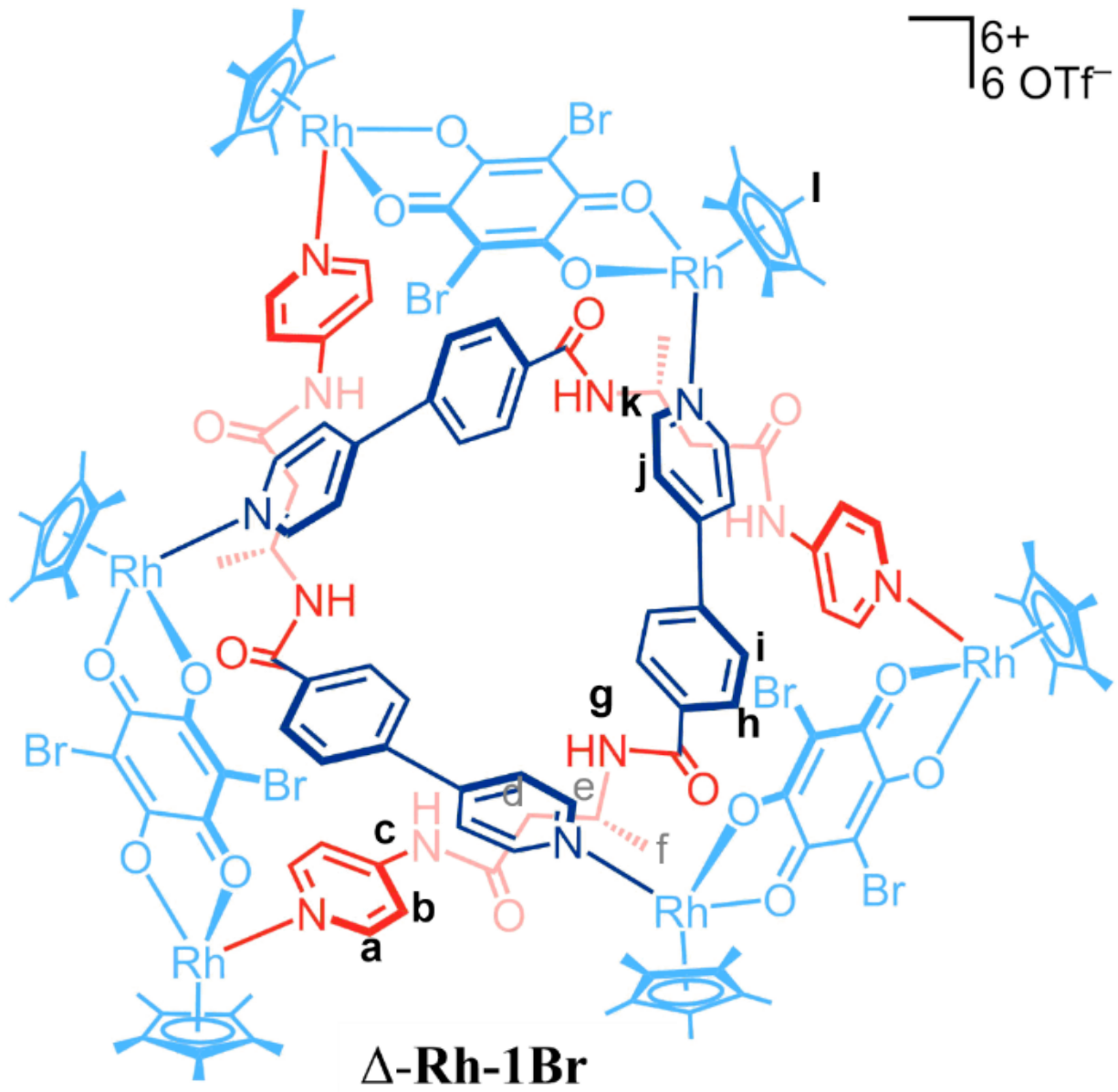

Δ-Rh-1Br

Δ-**Rh-1Br** (27.8mg, 97%) was synthesized by following the same procedure of Λ-**Rh-1Br**, except that (*S*)-**L1** was replaced by (*R*)-**L1** (0.02 mmol, 7.2 mg). ESI-MS *m/z*: 923.9919 (calcd for $[M - 4\ \mathrm{OTf}]^{4+}$: 923.9906). ^{1}H NMR (600 MHz, CD_3OD, ppm): δ = 8.54 (d, *J* = 5.7 Hz, 2H, H_a), 7.57 (d, *J* = 6.5 Hz, 2H, H_k), 7.21 (s, 2H, H_j), 7.05 (d, *J* = 6.1 Hz, 2H, H_b), 6.92 (s, 2H, H_i), 5.46 (d, *J* = 8.3 Hz, 2H, H_h), 4.94 – 4.89 (m, 1H, H_e), 3.02 – 2.97 (m, 1H, H_d), 2.79 – 2.76 (m, 1H, H_d), 1.77 (s, 15H, H_l), 1.56 (s, 15H, H_l), 1.37 (d, *J* = 6.9 Hz, 3H, H_f). ^{13}C NMR (151 MHz, CD_3OD, ppm): δ = 172.56, 165.85, 152.82, 151.92, 149.28, 139.81, 135.20, 129.30, 127.96, 126.86, 125.04, 122.93, 120.82, 118.71, 116.09, 97.86, 97.46, 43.53, 42.49, 21.42, 8.97, 8.60.

Synthesis of Λ-Ir-1

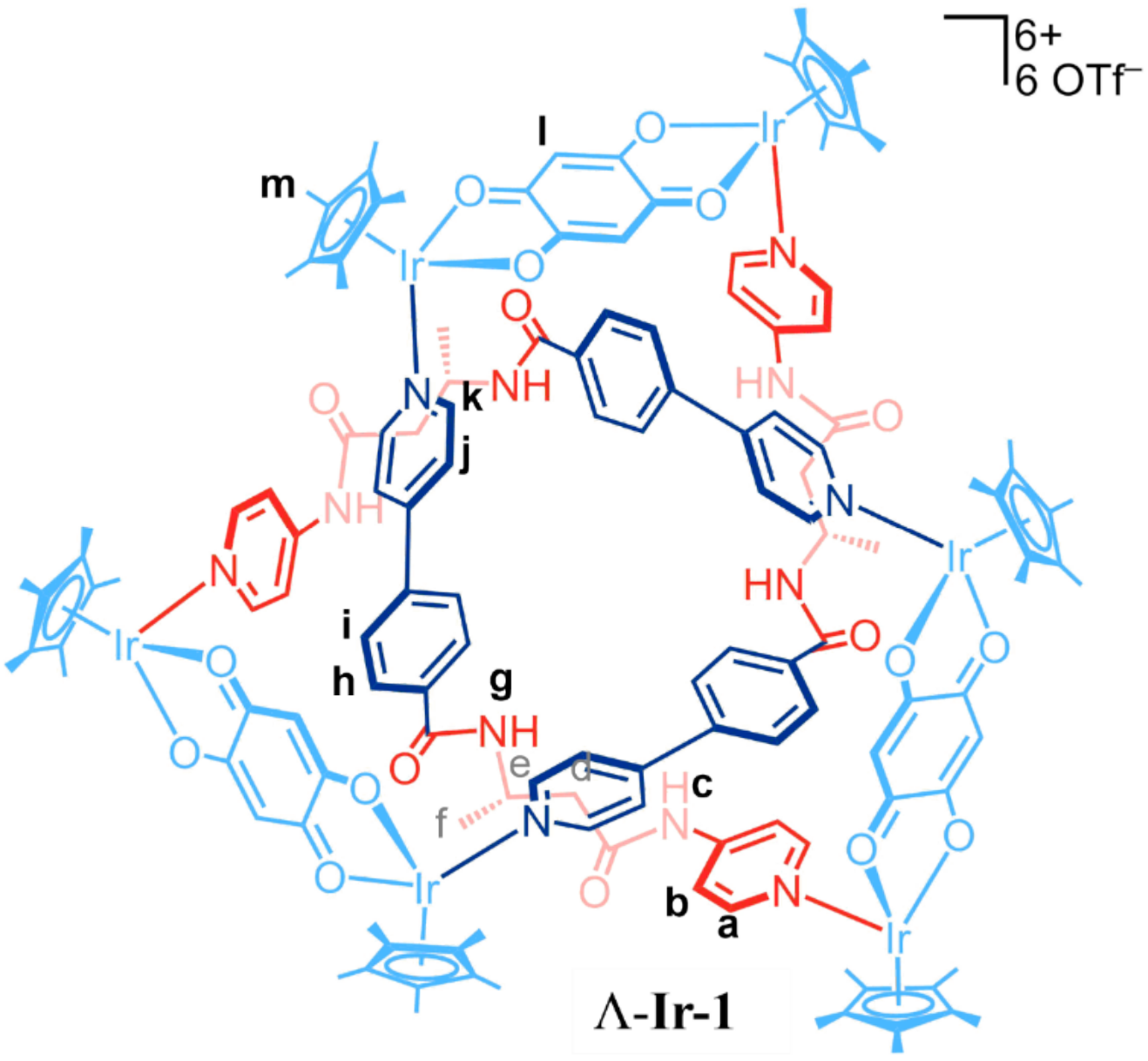

Λ-**Ir-1**

The MeOH (6 mL) solution of ligand (*S*)-**L1** (0.02 mmol, 7.2 mg) and **B3**(OTf)$_2$ (0.02 mmol, 21.8 mg) was stirred at room temperature for 1 day. The reaction mixture was filtered through a filter membrane, and the obtained filtrate was concentrated to 2 mL, and then 6 mL of diethyl ether was added to form purple solid of Λ-**Ir-1**, which was then washed with diethyl ether and dried under vacuum (27.3 mg, 94%). Single crystals of Λ-**Ir-1** suitable for X-ray diffraction were obtained by vapor diffusion of diethyl ether into a methanol solution of the assembly at room temperature (23.5mg, 81% isolated yield). ESI-MS *m/z*: 939.7084 (calcd for $[M - 4\ OTf]^{4+}$: 939.7115). ^1H NMR (600 MHz, CD_3OD, ppm): δ = 8.57 – 8.54 (m, 2H, H_a), 7.79 – 7.77 (m, 1H, H_j), 7.61 (d, *J* = 6.5 Hz, 2H, H_k), 7.20 (s, 1H, H_j), 7.13 – 7.10 (m, 2H, H_b), 6.92 (s, 2H, H_i), 6.35 (s, 1H, H_l), 5.77 (s, 1H, H_l), 5.70 (d, *J* = 8.2 Hz, 2H, H_h), 4.98 – 4.91 (m, 1H, H_e), 3.07 – 2.99 (m, 1H, H_d), 2.87 – 2.80 (m, 1H, H_d), 1.72 (s, 15H, H_m), 1.48 (s, 15H, H_m), 1.42 (d, *J* = 6.8 Hz, 3H, H_f). ^{13}C NMR (151 MHz, CD_3OD, ppm): δ = 187.28, 187.13, 186.39, 185.96, 172.55, 165.85, 153.49, 152.57, 152.49, 149.43, 139.79, 134.96, 128.00, 127.32, 126.95, 122.98, 120.87, 116.03, 104.12, 102.09, 88.83, 88.34, 43.56, 42.74, 21.46, 8.94, 8.64.

Synthesis of Δ-Ir-1

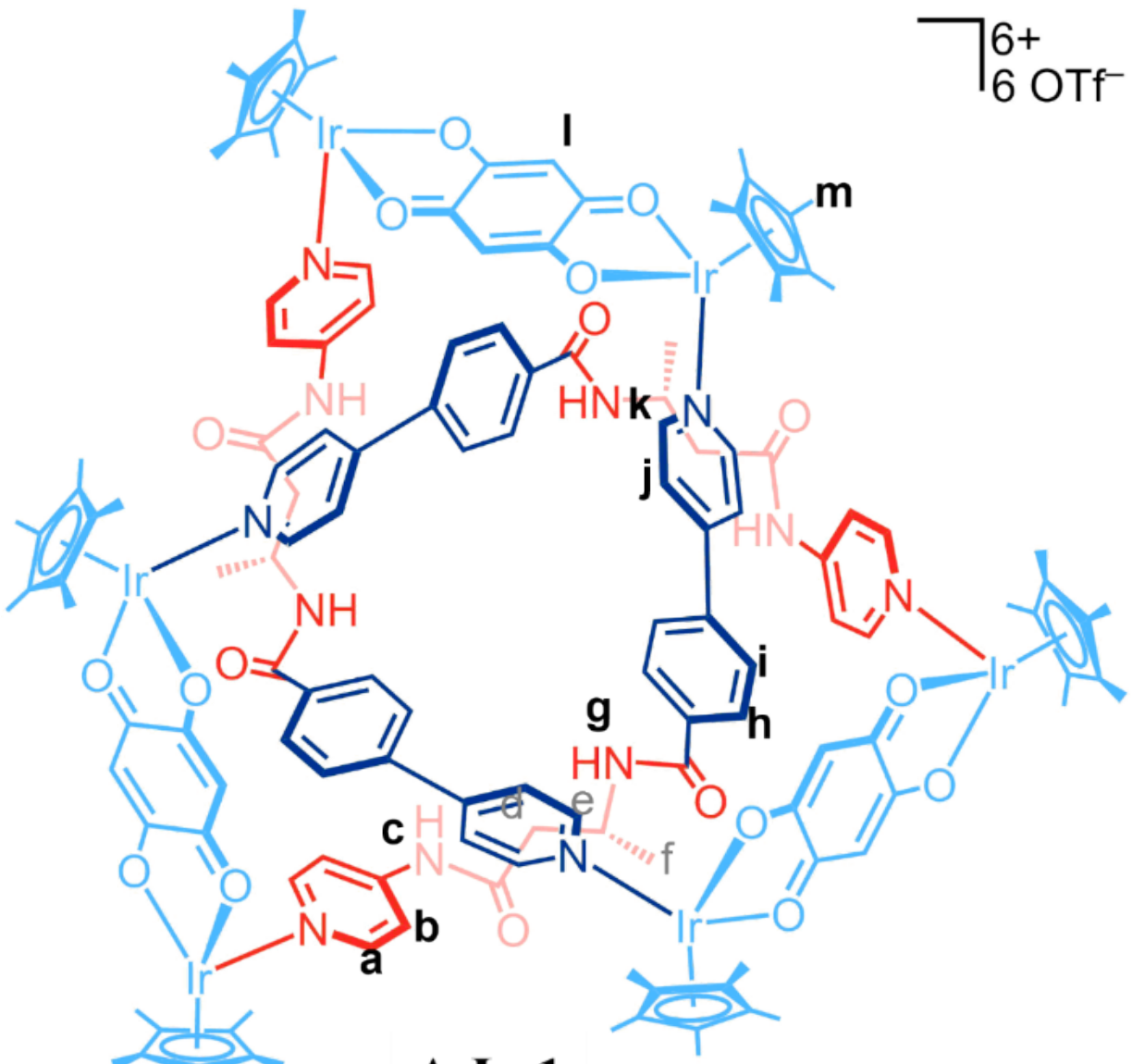

Δ-Ir-1

The MeOH (6 mL) solution of ligand (*R*)-**L1** (0.02 mmol, 7.2 mg) and **B3**(OTf)$_2$ (0.02 mmol, 21.8 mg) was stirred at room temperature for 1 day. The reaction mixture was filtered through a filter membrane, and the obtained filtrate was concentrated to 2 mL, and then 6 mL of diethyl ether was added to form purple solid of Δ-**Ir-1**, which was then washed with diethyl ether and dried under vacuum (26.4 mg, 91%). Single crystals of Δ-**Ir-1** suitable for X-ray diffraction were obtained by vapor diffusion of diethyl ether into a methanol solution of the assembly at room temperature (23.8mg, 82% isolated yield). ESI-MS *m/z*: 939.7100 (calcd for $[M - 4\ OTf]^{4+}$: 939.7115). ^{1}H NMR (600 MHz, CD$_3$OD, ppm): δ = 8.57 – 8.55 (m, 2H, H$_a$), 7.78 – 7.77 (m, 1H, H$_j$), 7.61 (d, *J* = 6.5 Hz, 2H, H$_k$), 7.25 (s, 1H, H$_j$), 7.14 – 7.12 (m, 2H, H$_b$), 6.91 (s, 2H, H$_i$), 6.35 (s, 1H, H$_l$), 5.77 (s, 1H, H$_l$), 5.70 (d, *J* = 8.2 Hz, 2H, H$_h$), 4.98 – 4.93 (m, 1H, H$_e$), 3.09 – 2.97 (m, 1H, H$_d$), 2.87 – 2.82 (m, 1H, H$_d$), 1.73 (s, 15H, H$_m$), 1.49 (s, 15H, H$_m$), 1.42 (d, *J* = 6.7 Hz, 3H, H$_f$). ^{13}C NMR (151 MHz, CD$_3$OD, ppm): δ = 187.28, 187.13, 186.39, 185.95, 172.55, 165.92, 153.49, 152.57, 152.49, 149.43, 139.79, 134.99, 128.01, 127.32, 126.95, 122.98, 120.87, 116.03, 104.12, 102.09, 88.83, 88.34, 43.60, 42.74, 21.48, 8.94, 8.64.

Self-assembly using equimolar (*S*)-L1 and (*R*)-L1

The racemic assembly was prepared using the procedure described for Λ-**Rh-1**, with an equimolar mixture of (*S*)-**L1** and (*R*)-**L1** (3.6 mg, 0.01mmol each) in place of (*S*)-**L1**. The total ligand-to-**B1**$(OTf)_2$ molar ratio was 1:1. The resulting orange precipitate was collected, washed with diethyl ether, and dried under vacuum (24.6 mg, 97%). Vapor diffusion of diethyl ether into a methanol solution of the assembly at room temperature afforded a crystalline racemic mixture of Λ-**Rh-1** and Δ-**Rh-1** (21.4 mg, 84% isolated yield). ESI-MS *m/z*: 805.3732 (calcd for [trefoil knot – 4 OTf]$^{4+}$: 805.3760). ^{1}H NMR (600 MHz, CD_3OD, ppm): *δ* = 8.54 – 8.53 (m, 2H, H_a), 7.53 (d, *J* = 6.4 Hz, 2H, H_k), 7.18 (s, 2H, H_j), 7.06 (s, 2H, H_i), 6.86 – 6.84 (m, 2H, H_b), 6.11 (s, 1H, H_l), 5.55 (d, *J* = 8.2 Hz, 2H, H_h), 5.50 (s, 1H, H_l), 4.97 – 4.93 (m, 1H, H_e), 2.98 – 2.94 (m, 1H, H_d), 2.82 – 2.79 (m, 1H, H_d), 1.77 (s, 15H, H_m), 1.51 (s, 15H, H_m), 1.40 (d, *J* = 6.8 Hz, 3H, H_f).

Synthesis of Rh-2*S*

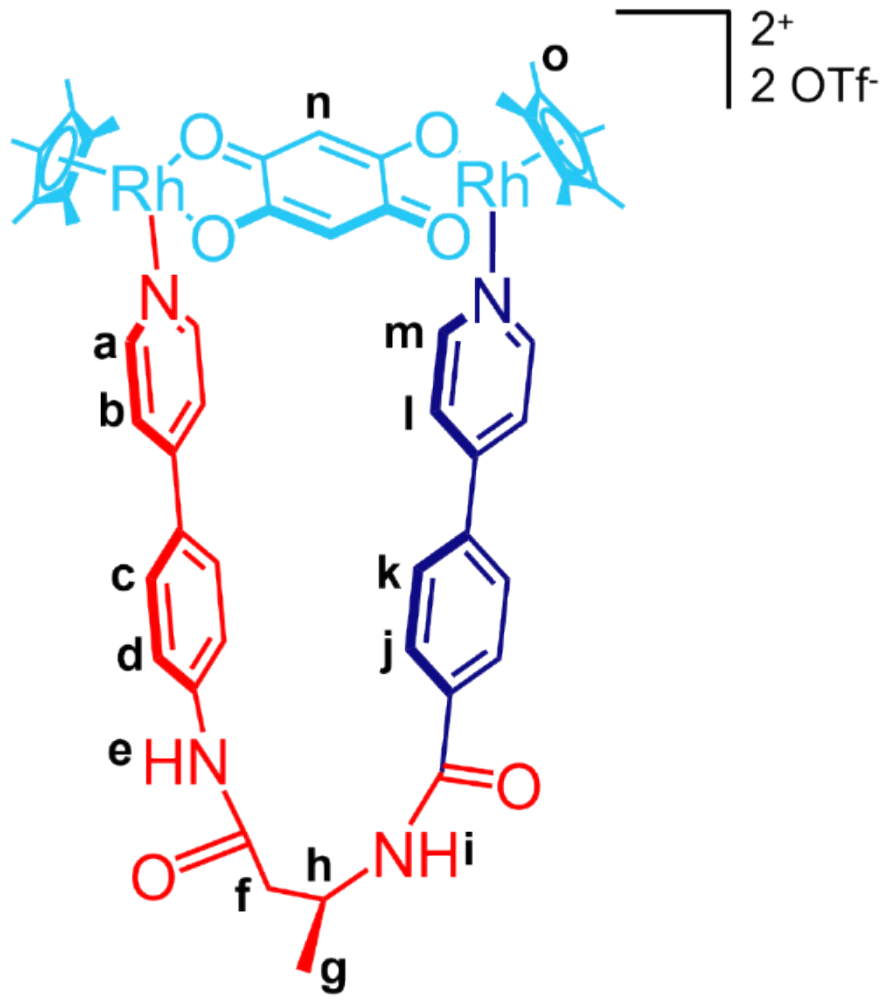

Rh-2*S* (25.3 mg, 94%) was synthesized by following the same procedure of Λ-**Rh-1**, except that (*S*)-**L1** was replaced by (*S*)-**L2** (0.02 mmol, 8.7 mg). Single crystals of **Rh-2*S*** suitable for X-ray diffraction were obtained by vapor diffusion of diethyl ether into a methanol solution of the assembly at room temperature (22.6mg, 84% isolated yield). ESI-MS *m/z*: 1199.1783 (calcd for [**Rh-2*S*** – OTf]$^{1+}$: 1199.1825). ^{1}H NMR (600 MHz, CD_3OD, ppm): δ = 8.31 – 8.29 (m, 2H, H_m), 8.22 – 8.19 (m, 2H, H_a), 7.72 –7.68 (m, 4H, $H_{l,b}$), 7.66 (d, *J* = 8.3 Hz, 2H, H_k), 7.61 (s, 4H, $H_{c,d}$), 7.53 – 7.49 (m, 2H, H_j), 5.66 (s, 1H, H_n), 5.62 (s, 1H, H_n), 4.68 – 4.59 (m, 1H, H_h), 2.84 (dd, *J* = 12.8, 5.5 Hz, 1H, H_f), 2.65 – 2.60 (m, 1H, H_f), 1.71 (s, 15H, H_o), 1.70 (s, 15H, H_o), 1.48 (d, *J* = 6.8 Hz, 3H, H_g). ^{13}C NMR (151 MHz, CD_3OD, ppm): δ = 184.71, 184.61, 184.40, 184.37, 172.22, 168.94, 168.87, 152.75, 152.45, 151.73, 151.51, 142.31, 139.62, 137.17, 131.09, 129.59, 128.59, 128.12, 125.52, 124.23, 122.88, 121.00, 120.77,118.66, 102.83, 102.76, 97.16, 97.09, 97.04, 46.12, 44.90, 21.06, 21.04, 8.70, 8.69.

Synthesis of Ir-2*S*

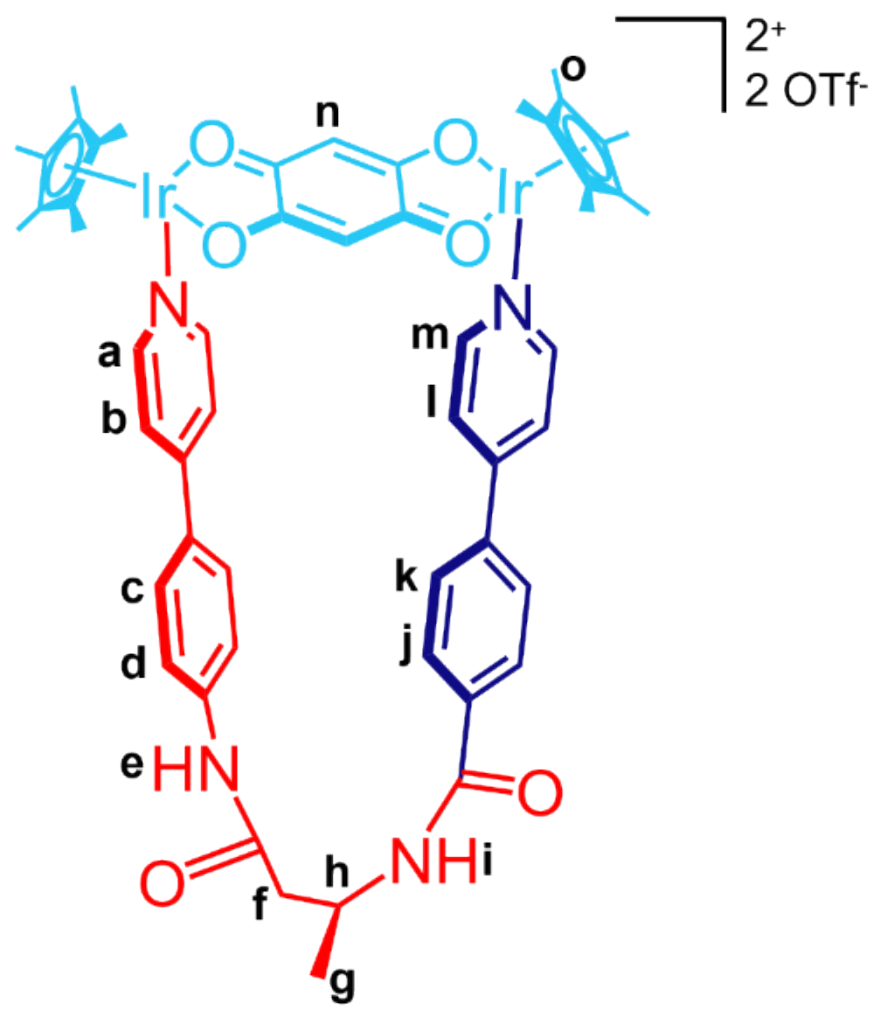

Ir-2*S* (28.7 mg, 94%) was synthesized by following the same procedure of Λ-**Ir-1**, except that (*S*)-**L1** was replaced by (*S*)-**L2** (0.02 mmol, 8.7 mg). Single crystals of **Ir-2*S*** suitable for X-ray diffraction were obtained by vapor diffusion of diethyl ether into a methanol solution of the assembly at room temperature (25.3mg, 83% isolated yield). ESI-MS *m/z*: 1379.2915 (calcd for [**Ir-2*S*** – OTf]$^{1+}$: 1379.2978). ^{1}H NMR (600 MHz, CD_3OD, ppm): δ = 8.31 – 8.30 (m, 2H, H_m), 8.21 – 8.20 (m, 2H, H_a), 7.73 – 7.72 (m, 4H, $H_{l,b}$), 7.68 – 7.66 (m, 2H, H_k), 7.61 – 7.60 (m, 4H, $H_{c,d}$), 7.51 – 7.50 (m, 2H, H_j), 5.91 (s, 1H, H_n), 5.86 (s, 1H, H_n), 4.68 – 4.62 (m, 1H, H_h), 2.81 (dd, *J* = 12.9, 5.6 Hz, 1H, H_f), 2.66 – 2.62 (m, 1H, H_f), 1.68 (s, 15H, H_o), 1.67 (s, 15H, H_o), 1.46 (d, *J* = 6.8 Hz, 3H, H_g). ^{13}C NMR (151 MHz, CD_3OD, ppm): δ = 186.61, 186.53, 186.48, 186.17, 172.25, 168.78, 153.22, 152.88, 152.03, 151.79, 142.56, 139.20, 137.31, 130.61, 129.62, 128.69,

128.17, 125.89, 124.51, 122.88, 120.96, 120.77, 118.66, 102.72, 102.64, 88.70, 88.68, 88.66, 46.13, 44.90, 21.02, 8.82, 8.80.

Synthesis of Rh-3

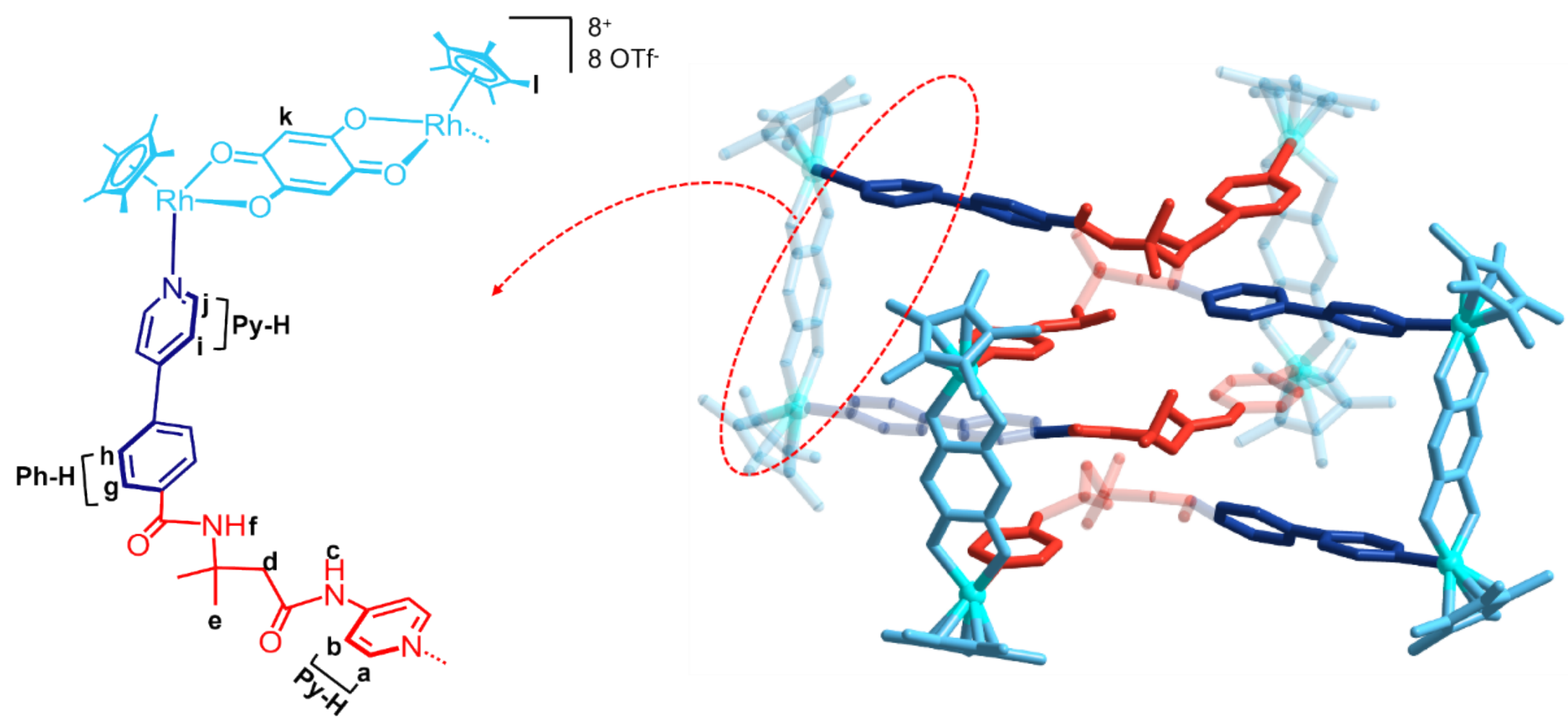

Rh-3 (24.4 mg, 95%) was synthesized by following the same procedure of Λ-**Rh-1**, except that (*S*)-**L1** was replaced by **L3** (0.02 mmol, 7.5 mg). Single crystals of **Rh-3** suitable for X-ray diffraction were obtained by vapor diffusion of diethyl ether into a methanol solution of the assembly at room temperature (21.6 mg, 84% isolated yield). No ESI-MS signals assignable to **Rh-3** were detected under the conditions examined. In the ^{1}H NMR assignments below, the suffix "mc" identifies resonances assigned to the coexisting tetranuclear metallomacrocycle, whereas labels without this suffix refer to the **Rh-3** component. ^{1}H NMR (600 MHz, CD_3OD, ppm): δ = 8.48 – 8.43 (m, 1H, $H_{py\text{-}H}$), 8.41 – 8.39 (m, 1H, $H_{py\text{-}H}$), 8.30 – 8.29 (m, 2H, H-mc), 8.09 – 8.08 (m, 1H, $H_{py\text{-}H}$), 8.02 – 8.01 (m, 2H, H-mc), 7.91 – 7.90 (m, 1H, $H_{py\text{-}H}$), 7.79 – 7.78 (m, 2H, $H_{py\text{-}H}$), 7.68 – 7.66 (m, 4H, H-mc), 7.66 – 7.64 (m, 1H, $H_{py\text{-}H}$), 7.47 – 7.44 (m, 2H, H-mc), 7.38 (s, 1H, $H_{py\text{-}H}$), 6.90 (s, 1H, $H_{Ph\text{-}H}$), 6.52 (s, 1H, $H_{Ph\text{-}H}$), 6.02 (s, 1H, $H_{Ph\text{-}H}$), 5.76 (s, 1H, $H_{Ph\text{-}H}$), 5.67 (s, 2H, H_k), 5.54 (s, 2H, H-mc), 2.96 (s, 2H, H-mc), 2.76 (s, 1H, H_d), 2.70 (s, 1H, H_d), 1.85 (s, 15H, H-mc), 1.74 (s, 15H, H_l), 1.72 (s, 15H, H_l), 1.68 (s, 15H, H_l), 1.66 (s, 15H, H_l), 1.51 (s, 15H, H-mc), 1.43 (s, 6H, H_e). ^{13}C NMR (151 MHz, CD_3OD, ppm): δ = 185.13, 184.98, 184.85, 184.83, 153.48, 152.91, 152.84, 152.80, 139.58, 129.55, 129.33, 128.50, 128.36, 128.11, 125.66, 124.56, 123.23, 122.91, 120.80, 118.69, 117.12, 116.77, 102.84, 97.18, 96.85, 55.38, 54.39, 28.86, 27.08, 8.92, 8.73, 8.70, 8.65, 8.64, 8.44.

Synthesis of Ir-3

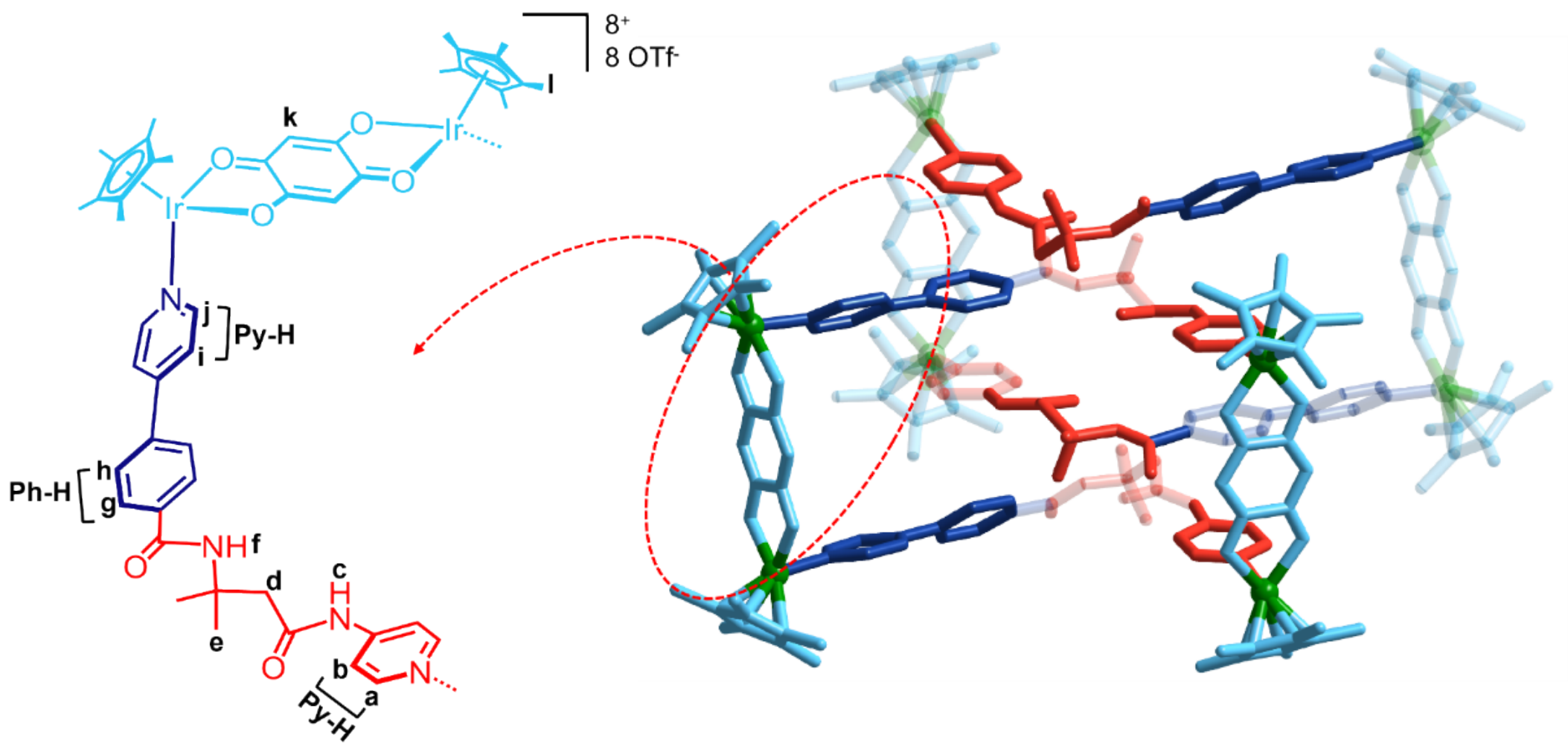

Ir-3 (26.7 mg, 91%) was synthesized by following the same procedure of Λ-**Ir-1**, except that (*S*)-**L1** was replaced by **L3** (0.02 mmol, 7.5 mg). Single crystals of **Ir-3** suitable for X-ray diffraction were obtained by vapor diffusion of diethyl ether into a methanol solution of the assembly at room temperature (24.0 mg, 82% isolated yield). ESI-MS *m/z*: 1805.0275 (calcd for [**Ir-3** – 3 OTf]$^{3+}$: 1805.0263); 827.8715 (calcd for [M_{mc} – 3OTf]$^{3+}$: 827.8699). Here, M_{mc} denotes the tetranuclear metallomacrocycle salt, including its four triflate counterions. In the ^{1}H NMR assignments below, the suffix "mc" identifies resonances assigned to the coexisting tetranuclear metallomacrocycle, whereas labels without this suffix refer to the **Ir-3** component. ^{1}H NMR (600 MHz, CD_3OD, ppm): δ = 8.50 – 8.47 (br, 1H, H_{py-H}), 8.43 – 8.42 (m, 1H, H_{py-H}), 8.33 – 8.31 (m, 2H, H-mc), 8.10 (d, *J* = 6.5 Hz, 1H, H_{py-H}), 8.04 (d, *J* = 6.8 Hz, 2H, H-mc), 7.95 (d, *J* = 6.1 Hz, 1H, H_{py-H}), 7.83 – 7.82 (m, 1H, H_{py-H}), 7.79 – 7.76 (m, 1H, H_{py-H}), 7.71 – 7.69 (m, 4H, H-mc), 7.67 – 7.65 (m, 1H, H_{py-H}), 7.53 – 7.50 (m, 2H, H-mc), 7.40 (s, 1H, H_{py-H}), 7.07 (s, 1H, H_{Ph-H}), 6.54 (s, 1H, H_{Ph-H}), 6.30 (s, 0.5H, H_{Ph-H}), 6.19 (s, 0.5H, H_{Ph-H}), 6.01 – 5.98 (m, 1H, H_{Ph-H}), 5.92 (s, 2H, H_k), 5.81 (s, 2H, H-mc), 2.96 (s, 2H, H-mc), 2.76 (s, 2H, H_d), 1.82 (s, 15H, H-mc), 1.70 (s, 15H, H_l), 1.69 (s, 15H, H_l), 1.64 (s, 15H, H_l), 1.62 (s, 15H, H_l), 1.49 (s, 15H, H-mc), 1.42 (s, 6H, H_e). ^{13}C NMR (151 MHz, CD_3OD, ppm): δ = 186.69, 186.60, 185.86, 172.75, 153.79, 153.40, 153.23, 153.18, 139.19, 138.97, 138.59, 129.63,

129.37, 128.57, 128.43, 128.26, 126.00, 125.16, 125.03, 122.92, 120.81, 118.69, 117.30, 116.86, 116.72, 102.74, 102.64, 88.94, 88.74, 88.69, 88.45, 88.38, 54.40, 28.87, 27.09, 9.05, 8.81, 8.78, 8.75, 8.58.

Synthesis of Rh-4Br*S*

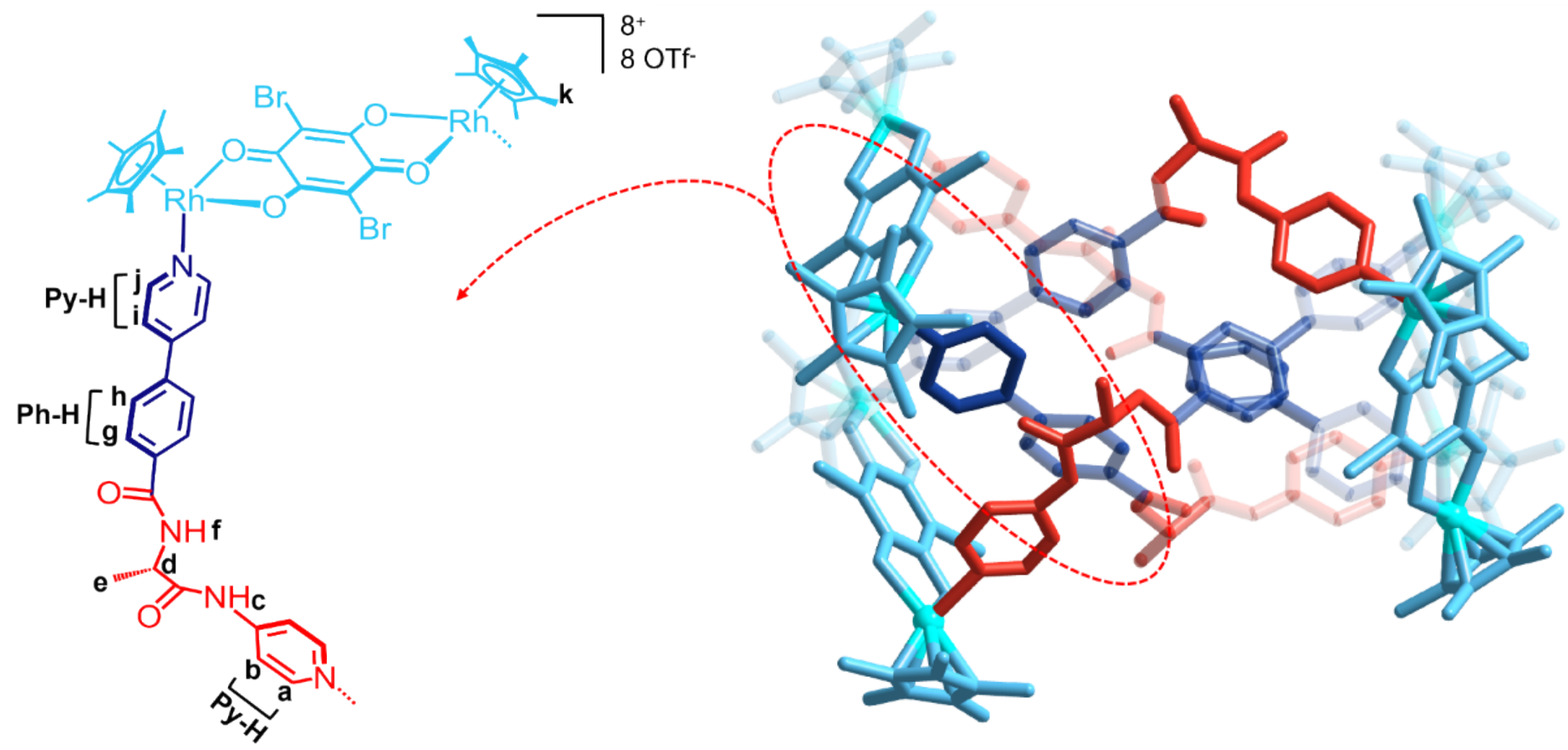

Rh-4Br*S* was synthesized by following the same procedure of Λ-**Rh-1Br**, except that (*S*)-**L1** was replaced by (*S*)-**L4** (0.02 mmol, 6.9 mg). The isolated material was obtained in a yield of 26.9 mg (95%). Vapor diffusion of diethyl ether into a methanol solution of the (*S*)-**L4/B2**(OTf)₂ assembly at room temperature afforded crystals of the figure-eight knot **Rh-4Br*S*** (21.2 mg, 75% isolated yield). No ESI-MS signals assignable to **Rh-4Br*S*** were detected under the conditions examined. In the ^{1}H NMR assignments below, the suffix "mc" identifies resonances assigned to the coexisting tetranuclear metallomacrocycle, whereas labels without this suffix refer to the figure-eight knot component. ^{1}H NMR (600 MHz, CD_3OD, ppm): δ = 8.44 (s, 1H, H$_{py\text{-}H}$), 8.38 (d, *J* = 5.7 Hz, 2H, H-mc), 8.30 (s, 1H, H$_{py\text{-}H}$), 8.17 – 8.16 (m, 2H, H-mc), 8.11 (s, 1H, H$_{py\text{-}H}$), 8.00 (s, 1H, H$_{py\text{-}H}$), 7.93 – 7.91 (m, 2H, H-mc), 7.86 – 7.84 (m, 1H, H$_{Py\text{-}H}$), 7.79 (s, 4H, H-mc), 7.76 – 7.74 (m, 2H, H$_{py\text{-}H,ph\text{-}H}$), 7.71 – 7.69 (m, 2H, H-mc), 7.61 – 7.60 (m, 1H, H$_{ph\text{-}H}$), 7.37 (s, 1H, H$_{Py\text{-}H}$), 7.01 (s, 1H, H$_{Py\text{-}H}$), 6.75 (d, *J* = 6.5 Hz, 1H, H$_{ph\text{-}H}$), 6.18 (s, 1H, H$_{ph\text{-}H}$), 4.63 (d, *J* = 7.4 Hz, 1H, H-mc), 4.49 – 4.41 (m, 1H, H$_d$), 1.91 (s, 15H, H$_k$), 1.76 (s, 15H, H-mc), 1.72 (s, 15H, H-mc), 1.66 (s, 15H, H$_k$), 1.65 (s, 3H, H-mc), 1.56 – 1.55 (m, 3H, H$_e$). ^{13}C NMR (151 MHz, CD_3OD, ppm): δ = 179.01, 152.72, 129.74, 128.39, 124.98, 122.87, 120.76, 118.65, 116.50, 98.30, 98.03, 97.88, 97.85, 97.56, 96.11, 66.90, 61.54, 15.44, 14.46, 9.39, 9.22, 8.78, 8.74, 8.72, 8.68.

Synthesis of Rh-5 and Rh-5′

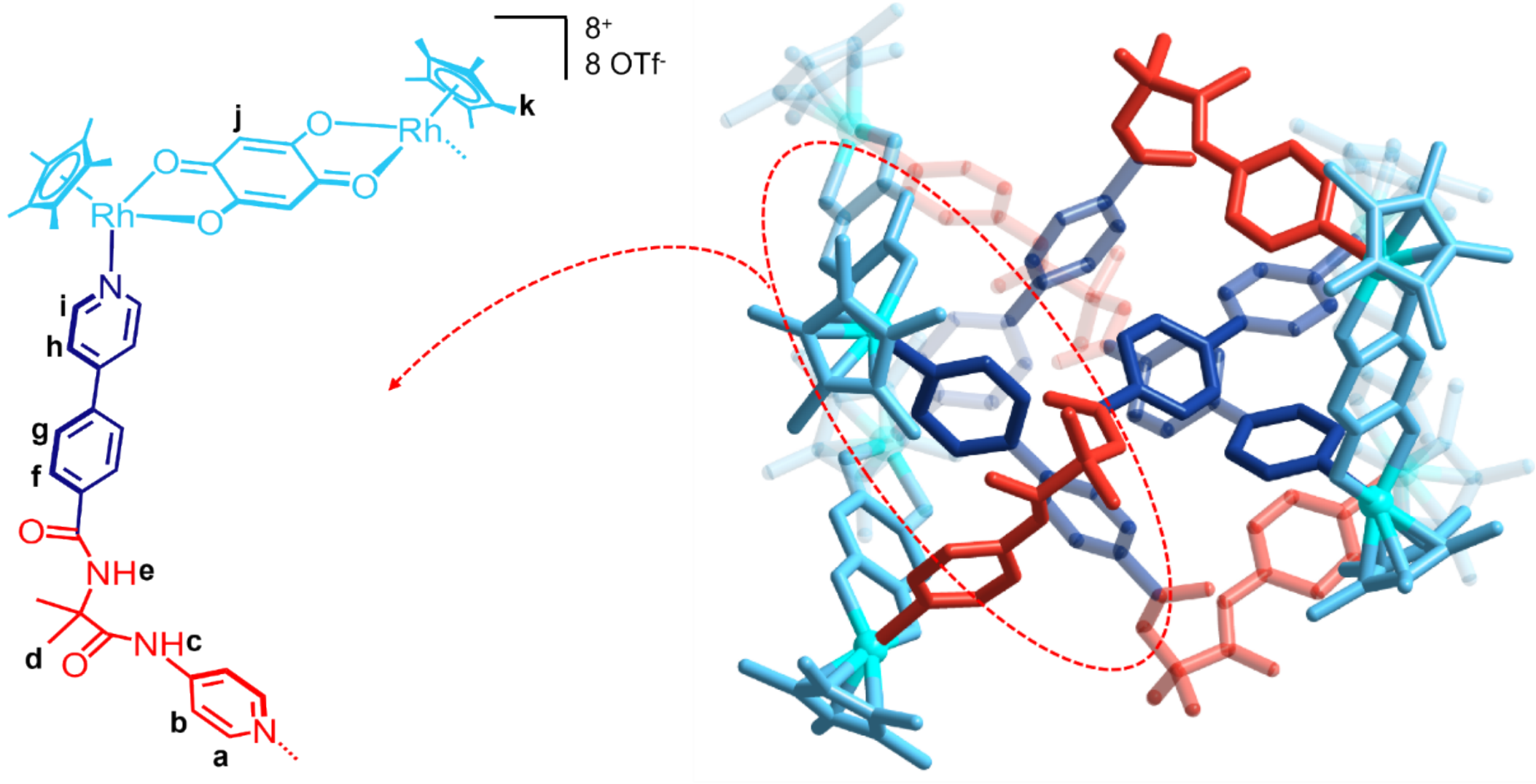

Rh-5 and **Rh-5′** were synthesized by following the same procedure of Λ-**Rh-1**, except that (*S*)-**L1** was replaced by **L5** (0.02 mmol, 7.2 mg). The isolated material was obtained in a combined yield of 23.0 mg (91%). Vapor diffusion of diethyl ether into a methanol solution of the **L5**/**B1**(OTf)$_2$ assembly at room temperature afforded crystals of the figure-eight knot **Rh-5** and the tetranuclear metallomacrocycle **Rh-5′** (20.9 mg, 82% combined isolated yield). ESI-MS *m/z*: 1547.8510 (calcd for [**Rh-5** – 3 OTf]$^{3+}$: 1547.8542); 699.4503 (calcd for [**Rh-5′** – 3 OTf]$^{3+}$: 699.4510). In the ^{1}H NMR assignments below, the suffix "mc" identifies resonances assigned to the coexisting tetranuclear metallomacrocycle **Rh-5′**, whereas labels without this suffix refer to the figure-eight knot component. ^{1}H NMR (600 MHz, CD$_3$OD, ppm): δ = 8.45 – 8.43 (m, 2H, H$_i$-mc), 8.06 (d, *J* = 6.7 Hz, 2H, H$_a$-mc), 8.00 (d, *J* = 6.7 Hz, 2H, H$_h$-mc), 7.77 – 7.75 (m, 4H, H$_{b,f}$-mc), 7.59 – 7.54 (m, 2H, H$_i$), 7.53 (d, *J* = 6.5 Hz, 2H, H$_a$), 7.39 (d, *J* = 8.3 Hz, 2H, H$_g$-mc), 6.89 (s, 2H, H$_b$), 6.48 (s, 2H, H$_h$), 6.34 (s, 1H, H$_j$), 6.17 (s, 1H, H$_j$), 5.69 (s, 2H, H$_j$-mc), 1.84 (s, 15H, H$_k$), 1.71 (s, 15H, H$_k$-mc), 1.67 (s, 15H, H$_k$-mc), 1.62 (s, 3H, H$_d$), 1.60 (s, 3H, H$_d$), 1.59 (s, 15H, H$_k$), 1.48 (s, 6H, H$_d$-mc).^{13}C NMR (151 MHz, CD$_3$OD, ppm): δ = 185.24, 185.09, 184.99, 184.94, 184.83, 184.78, 175.83, 168.57, 166.83, 152.86, 152.55, 151.65, 149.49, 148.54, 139.83, 137.23, 136.22, 135.80, 129.64, 129.14,

128.73, 127.50, 126.03, 125.03, 122.92, 120.80, 118.69, 117.13, 116.36, 104.03, 103.13, 102.91, 97.60, 97.20, 96.84, 59.70, 26.19, 23.69, 9.12, 8.64.

Synthesis of Rh-5Br

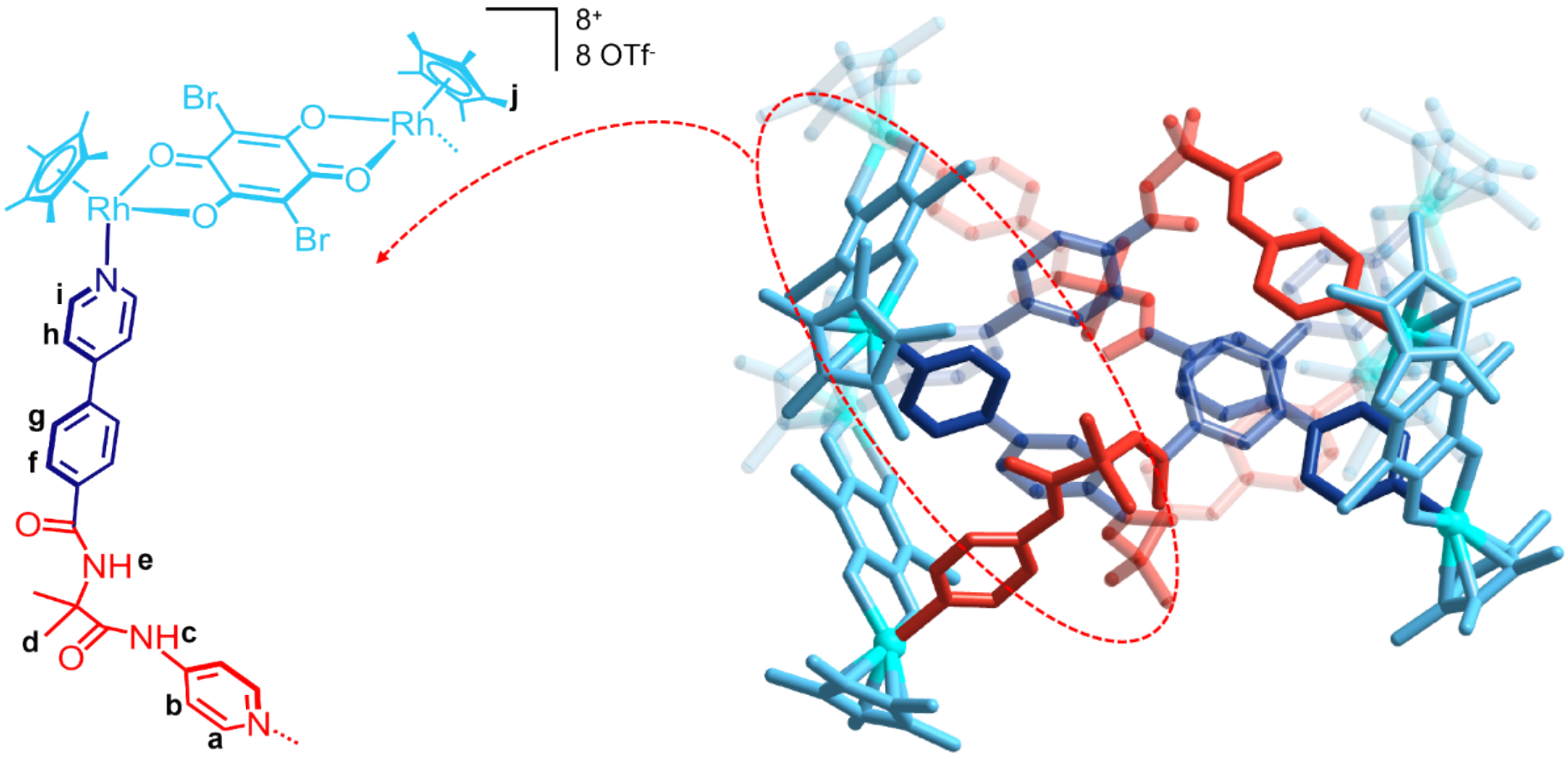

Rh-5Br was synthesized by following the same procedure of Λ-**Rh-1Br**, except that (*S*)-**L1** was replaced by **L5** (0.02 mmol, 7.2 mg). The isolated material was obtained in a yield of 26.3 mg (92%). Vapor diffusion of diethyl ether into a methanol solution of the **L5**/**B2**(OTf)$_2$ assembly at room temperature afforded crystals of the figure-eight knot **Rh-5Br** (20.3 mg, 71% isolated yield). ESI-MS *m/z*: 1758.2709 (calcd for [**Rh-5Br** – 3 OTf]$^{3+}$: 1758.2798); 804.3278 (calcd for [M_{mc} – 3 OTf]$^{3+}$: 804.3301). Here, M_{mc} denotes the tetranuclear metallomacrocycle salt, including its four triflate counterions. In the ^{1}H NMR assignments below, the suffix "mc" identifies resonances assigned to the coexisting tetranuclear metallomacrocycle, whereas labels without this suffix refer to the figure-eight knot component. ^{1}H NMR (600 MHz, CD_3OD, ppm): δ = 8.47 – 8.46 (m, 2H, H_i-mc), 8.08 (d, *J* = 6.5 Hz, 2H, H_a-mc), 8.00 – 7.99 (m, 2H, H_h-mc), 7.78 – 7.76 (m, 4H, $H_{b,f}$-mc), 7.63 (d, *J* = 6.6 Hz, 2H, H_i), 7.49 – 7.48 (m, 2H, H_a), 7.43 (d, *J* = 7.9 Hz, 2H, H_g-mc), 6.93 (s, 2H, H_h), 6.25 (s, 2H, H_b), 1.89 (s, 15H, H_j), 1.74 (s, 15H, H_j-mc), 1.71 (s, 15H, H_j-mc), 1.63 (s, 15H, H_j), 1.58 (s, 6H, H_d), 1.48 (s, 6H, H_d-mc). ^{13}C NMR (151 MHz, CD_3OD, ppm): δ = 178.91, 178.84, 178.75, 178.68, 178.62, 176.10, 175.37, 168.54, 167.26, 152.69, 152.40, 151.76, 150.64, 149.56, 148.73, 137.24, 136.21, 136.14, 129.99, 129.21, 128.68, 127.28, 126.10, 122.87, 120.76, 118.65, 117.25,

116.63, 98.29, 97.98, 97.74, 97.50, 97.18, 96.19, 96.06, 66.89, 59.80, 59.03, 26.26, 24.90, 23.70, 15.43, 9.34, 8.74.

Synthesis of Ir-5 and Ir-5′

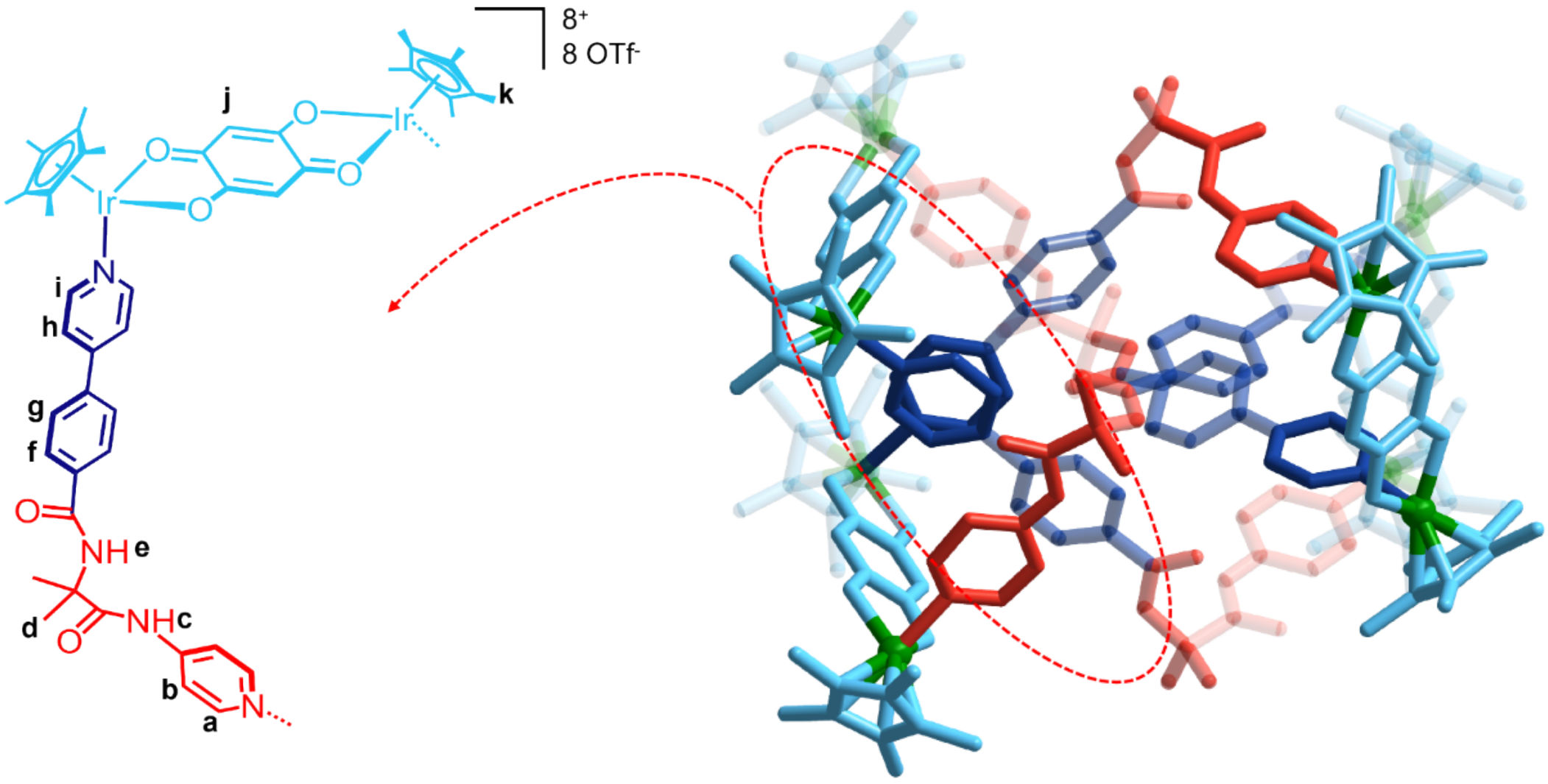

Ir-5 and **Ir-5′** were synthesized by following the same procedure of Λ-**Ir-1**, except that (*S*)-**L1** was replaced by **L5** (0.02 mmol, 7.2 mg). The isolated material was obtained in a combined yield of 26.7 mg (92%). Vapor diffusion of diethyl ether into a methanol solution of the **L5**/**B3**$(OTf)_2$ assembly at room temperature afforded crystals of the figure-eight knot **Ir-5** and the tetranuclear metallomacrocycle **Ir-5′** (24.9 mg, 86% combined isolated yield). ESI-MS *m/z*: 1786.3269 (calcd for [**Ir-5** – 3 OTf]$^{3+}$: 1786.3388); 818.5212 (calcd for [**Ir-5′** – 3 OTf]$^{3+}$: 818.5261). In the ^{1}H NMR assignments below, the suffix "mc" identifies resonances assigned to the coexisting tetranuclear metallomacrocycle **Ir-5′**, whereas labels without this suffix refer to the figure-eight knot component. ^{1}H NMR (600 MHz, CD_3OD, ppm): δ = 8.47 – 8.45 (m, 2H, H_i-mc), 8.08 (d, *J* = 6.8 Hz, 2H, H_a-mc), 8.04 – 8.02 (m, 2H, H_h-mc), 7.79 – 7.77 (m, 4H, $H_{b,f}$-mc), 7.55 (d, *J* = 6.7 Hz, 4H, $H_{i,a}$), 7.38 (d, *J* = 8.3 Hz, 2H, H_g-mc), 6.92 (s, 2H, H_b), 6.63 (s, 1H, H_j), 6.48 (s, 1H, H_j), 5.93 (s, 2H, H_j-mc), 1.82 (s, 15H, H_k), 1.67 (s, 15H, H_k-mc), 1.63 (s, 15H, H_k-mc), 1.60 (s, 3H, H_d), 1.57 (s, 3H, H_d), 1.54 (s, 15H, H_k), 1.49 (s, 6H, H_d-mc). ^{13}C NMR (151 MHz, CD_3OD, ppm): δ = 186.72, 186.57, 176.09, 168.50, 153.32, 152.93, 152.16, 150.78, 139.41, 136.38, 129.76, 129.13, 128.82, 128.50, 126.33, 125.83, 125.02, 122.91, 120.80, 118.68, 117.55, 117.25, 116.43, 102.63, 88.61, 88.38, 59.04, 24.89, 8.80, 8.78, 8.76, 8.73.

5. Molecular Structures

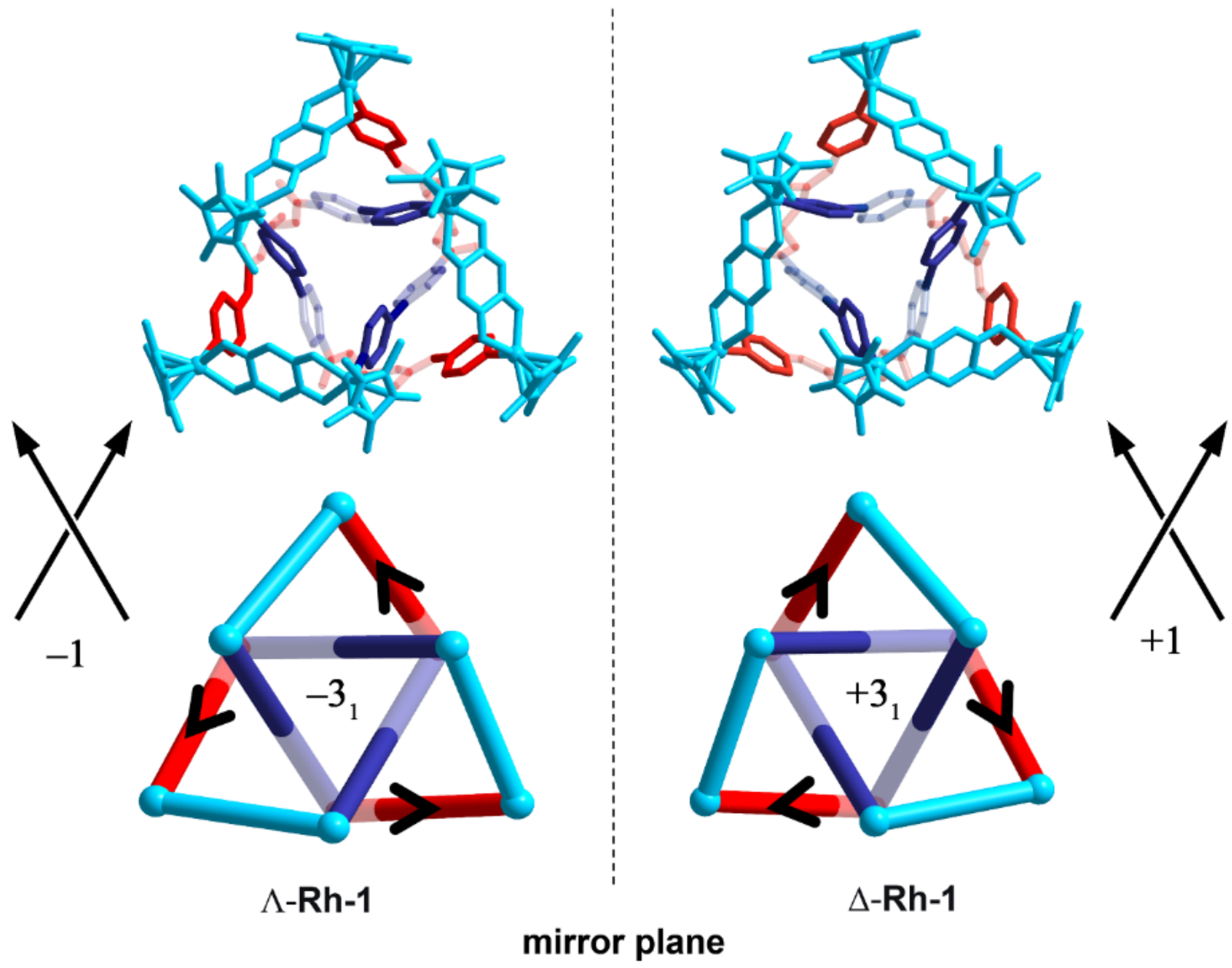

Fig. S2. Single-crystal structures and topological representations of the trefoil knots Λ-**Rh-1** and Δ-**Rh-1**. Counteranions and hydrogen atoms are omitted for clarity.

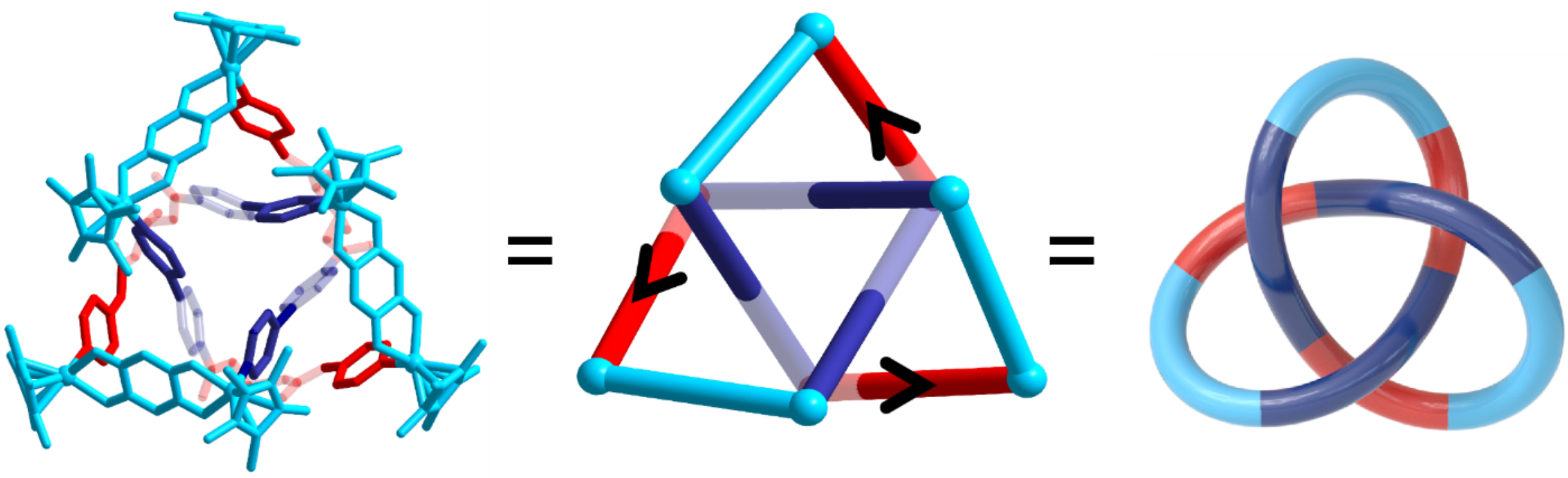

Fig. S3. Crystal structure and schematic representations illustrating the trefoil (3_1) topology of Λ-**Rh-1**. Counteranions and hydrogen atoms are omitted for clarity.

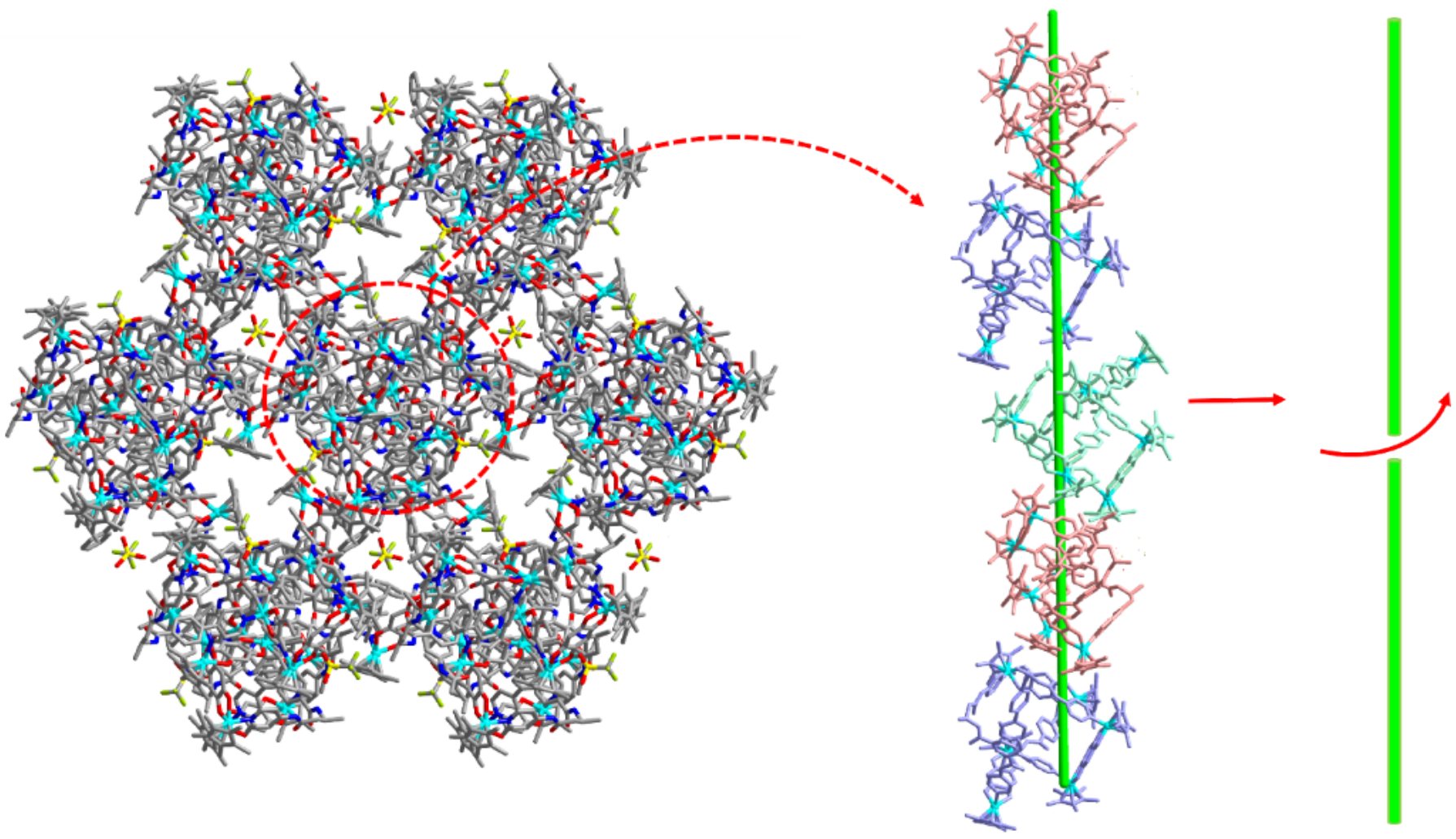

Fig. S4. Crystal packing of Λ-**Rh-1**, illustrating a representative 3_1 helical arrangement of trefoil knots along the $[\bar{1}1\bar{1}]$ direction. Hydrogen atoms are omitted for clarity.

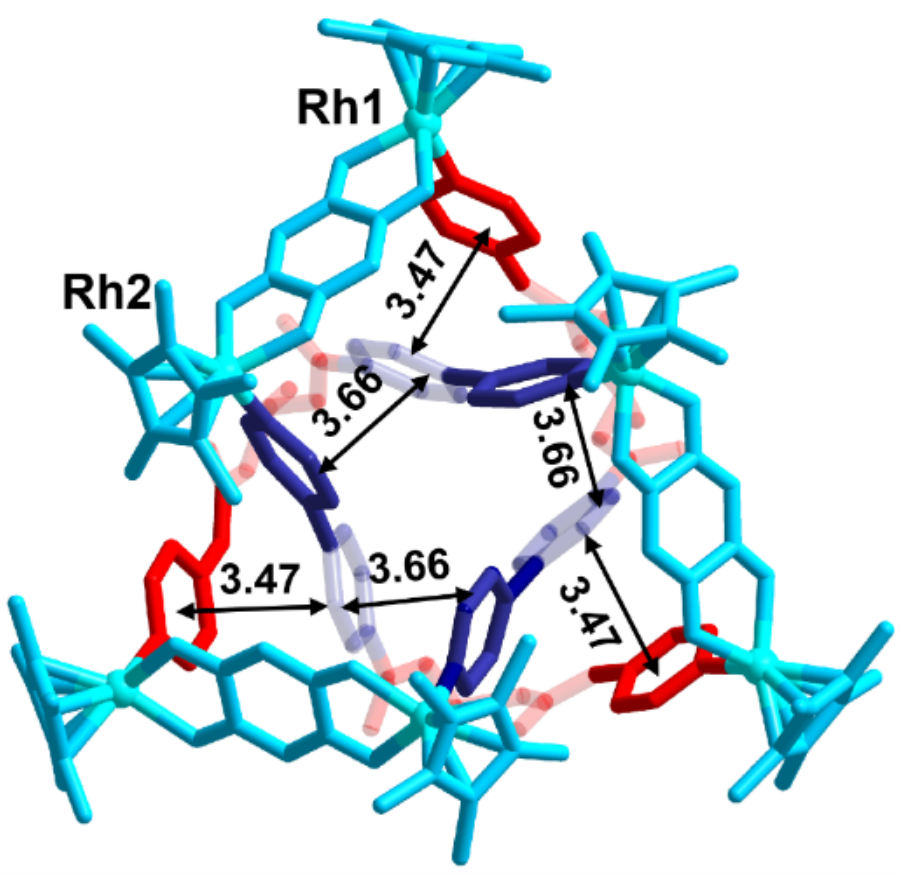

Fig. S5. The □-□ stacking interactions in trefoil knot Λ-**Rh-1** (unit: Å). Counteranions and hydrogen atoms are omitted for clarity.

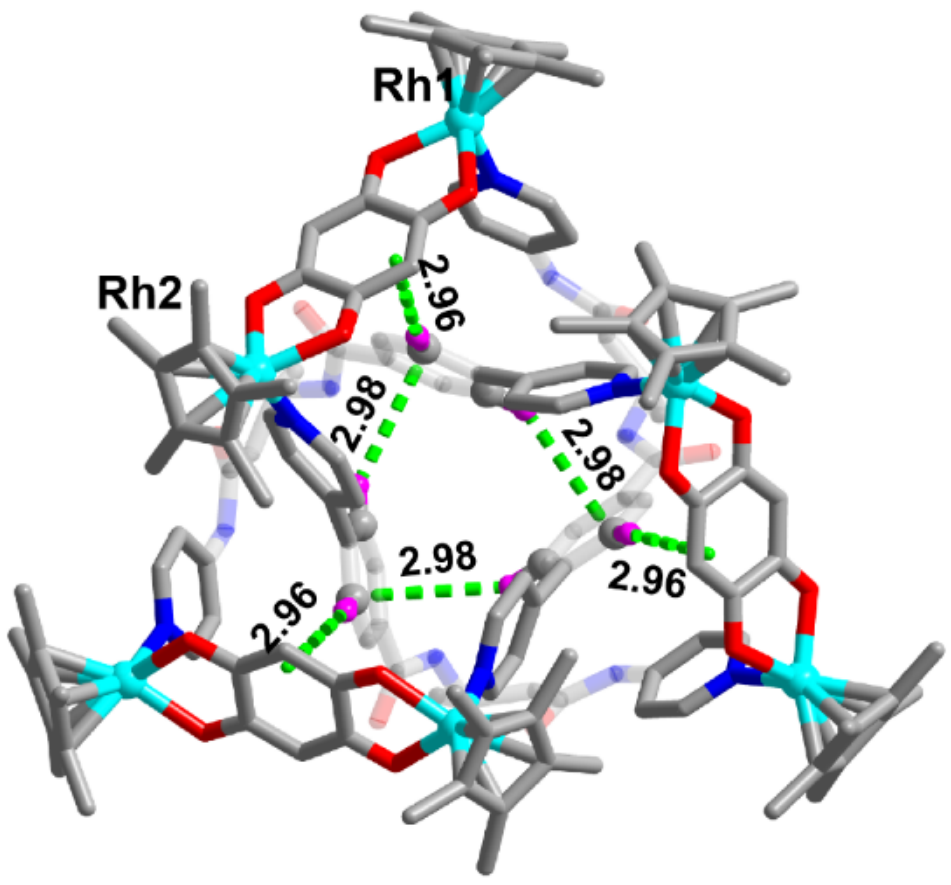

Fig. S6. The edge-to-face CH···□ interactions (bright green dotted lines) in trefoil knot Λ-**Rh-1** (unit: Å). Counteranions and non-essential hydrogen atoms are omitted for clarity. Color code: C, gray; H, pink; N, blue; O, red; Rh, turquoise.

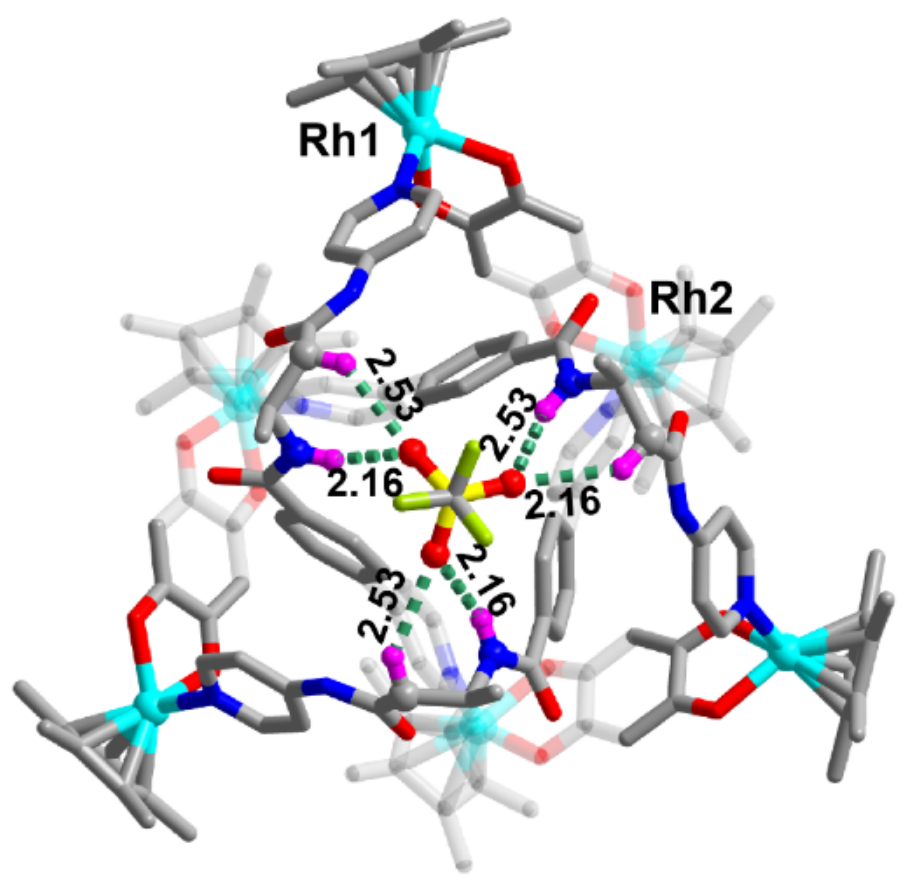

Fig. S7. The C-H···O hydrogen bonds and the N-H···O hydrogen bonds (sea green dotted lines) between trefoil knot Λ-**Rh-1**$^{6+}$ and a triflate anion (unit: Å). Other counteranions and non-essential hydrogen atoms are omitted for clarity. Color code: C, gray; H, pink; N, blue; O, red; F, lime; S, yellow; Rh, turquoise.

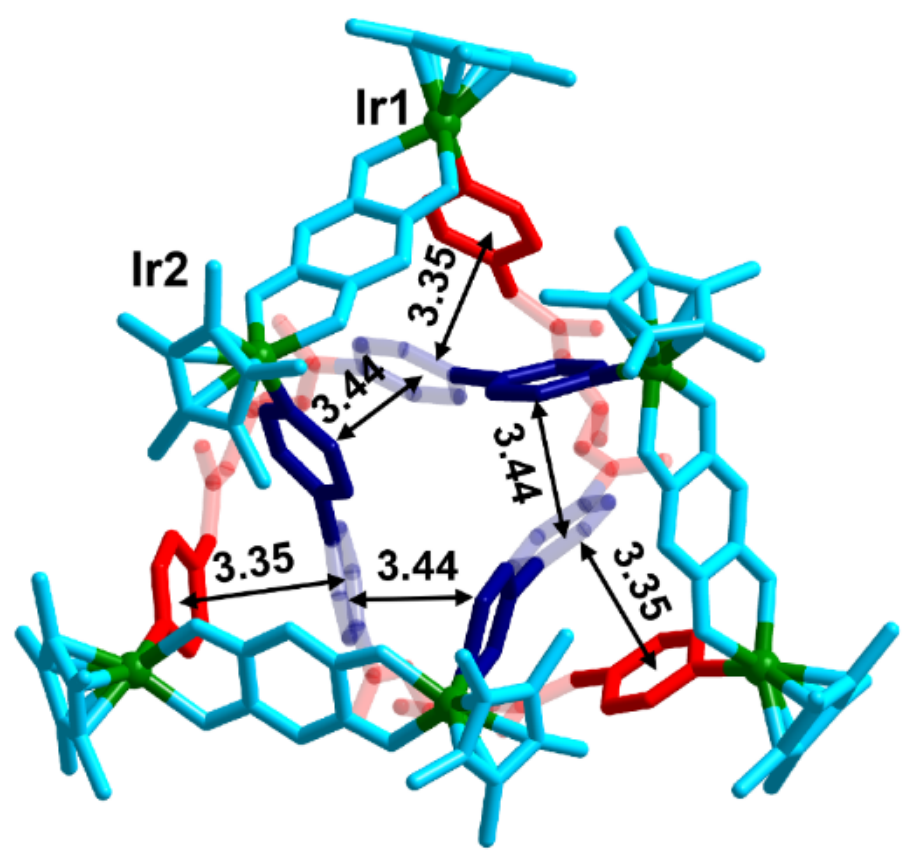

Fig. S8. The □-□ stacking interactions in trefoil knot Λ-**Ir-1** (unit: Å). Counteranions and hydrogen atoms are omitted for clarity.

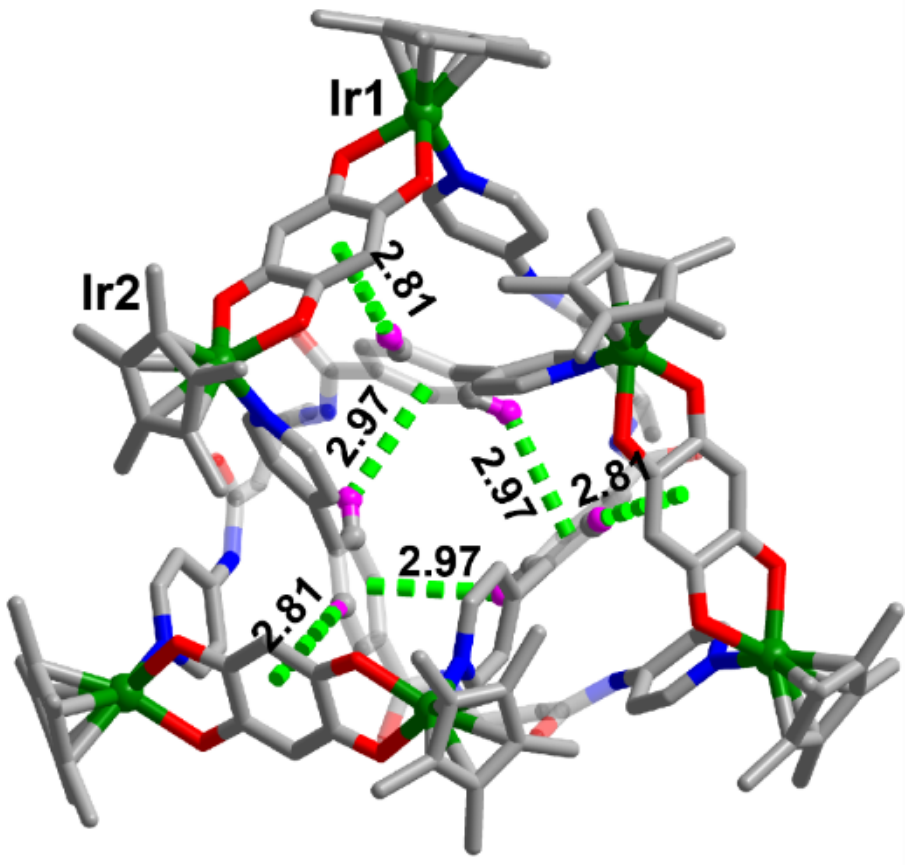

Fig. S9. The edge-to-face C-H···□ interactions (bright green dotted lines) in trefoil knot Λ-**Ir-1** (unit: Å). Counteranions and non-essential hydrogen atoms are omitted for clarity. Color code: C, gray; H, pink; N, blue; O, red; Ir, green.

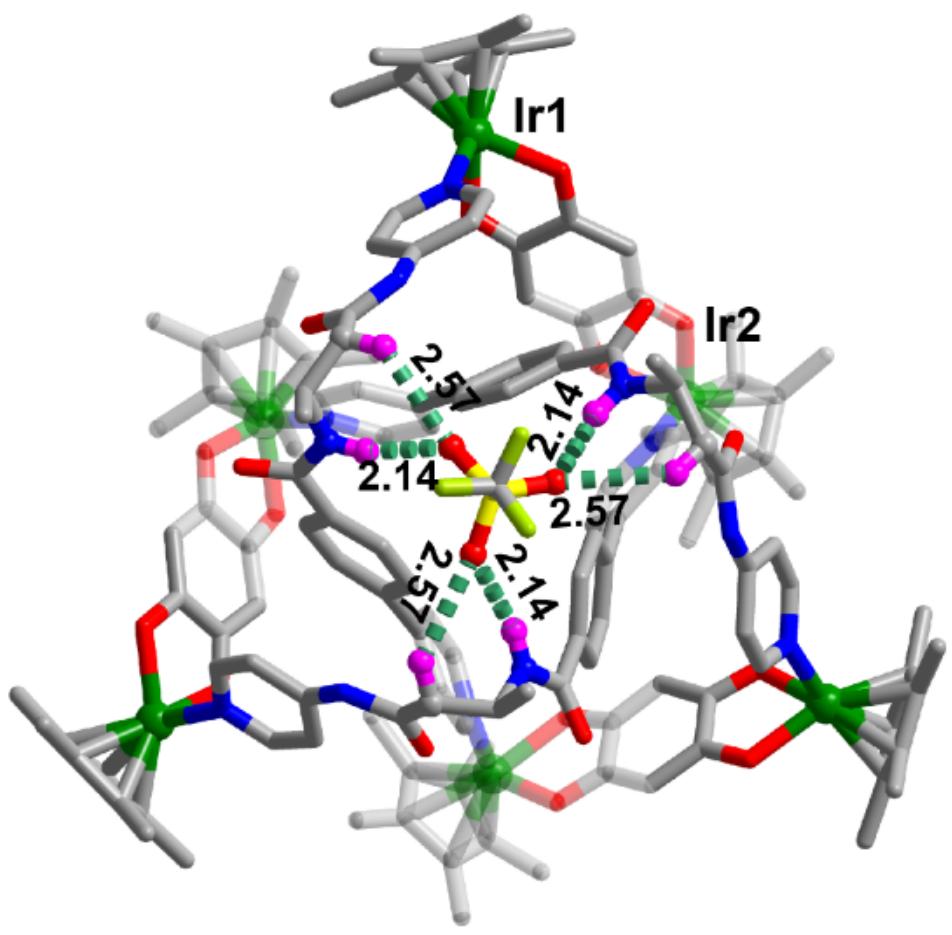

Fig. S10. The C-H···O hydrogen bonds and the N-H···O hydrogen bonds (sea green dotted lines) between trefoil knot Λ-**Ir-1**$^{6+}$ and a triflate anion (unit: Å). Other counteranions and non-essential hydrogen atoms are omitted for clarity. Color code: C, gray; H, pink; N, blue; O, red; F, lime; S, yellow; Ir, green.

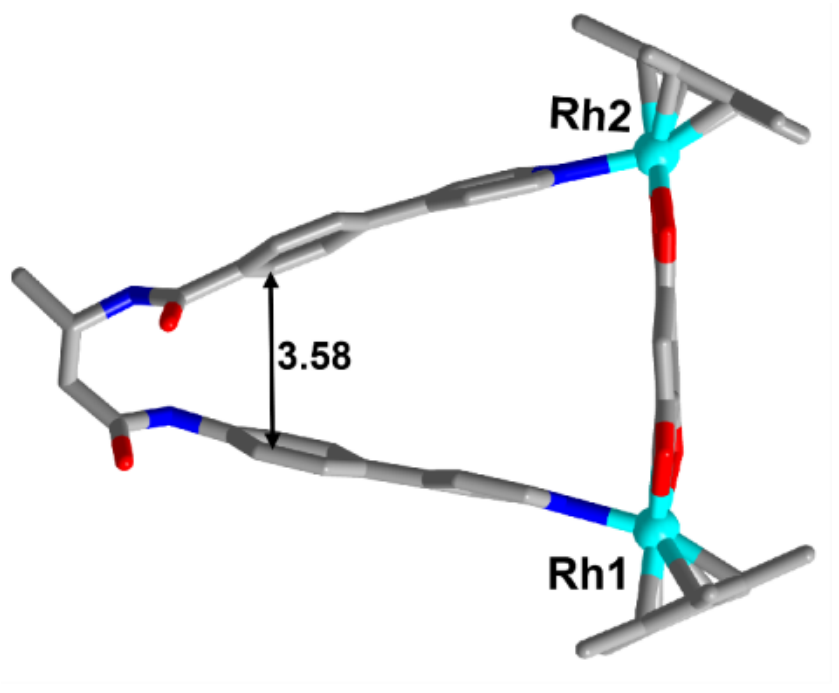

Fig. S11. The π-π stacking interactions in **Rh-2*S*** (unit: Å). Counteranions and non-essential hydrogen atoms are omitted for clarity. Color code: C, gray; N, blue; O, red; Rh, turquoise.

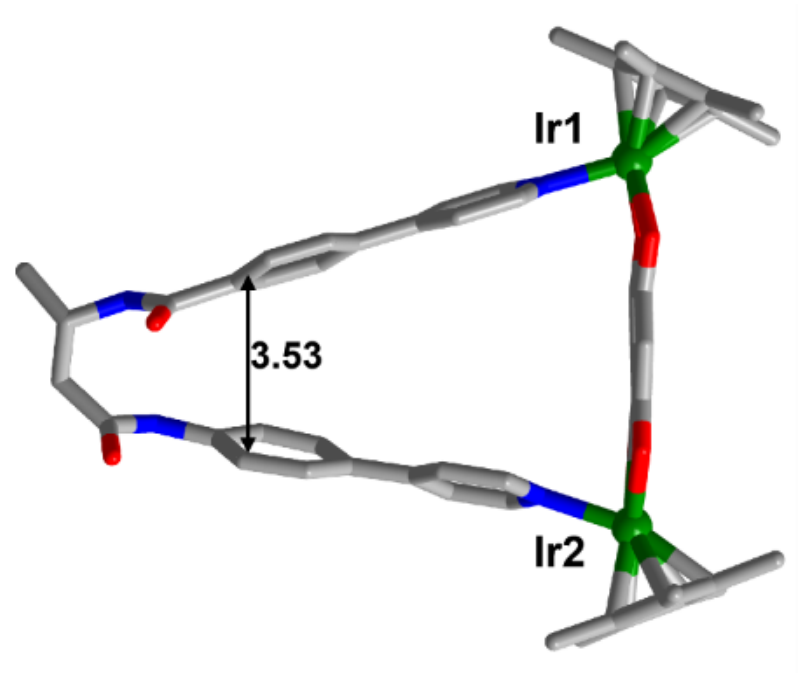

Fig. S12. The π-π stacking interactions in **Ir-2*S***(unit: Å). Counteranions and non-essential hydrogen atoms are omitted for clarity. Color code: C, gray; N, blue; O, red; Ir, green.

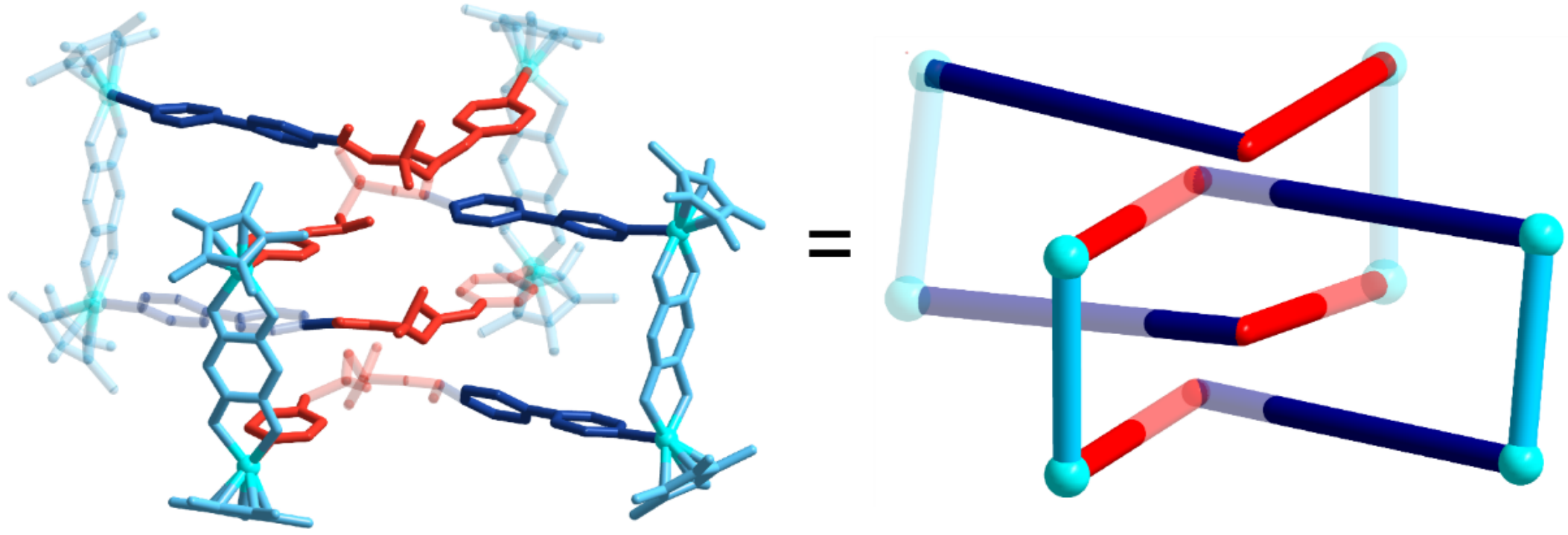

Fig. S13. Single-crystal structure and schematic representation of **Rh-3**, a non-interlocked nested dimer of two tetranuclear metallomacrocycles. Counteranions and hydrogen atoms are omitted for clarity.

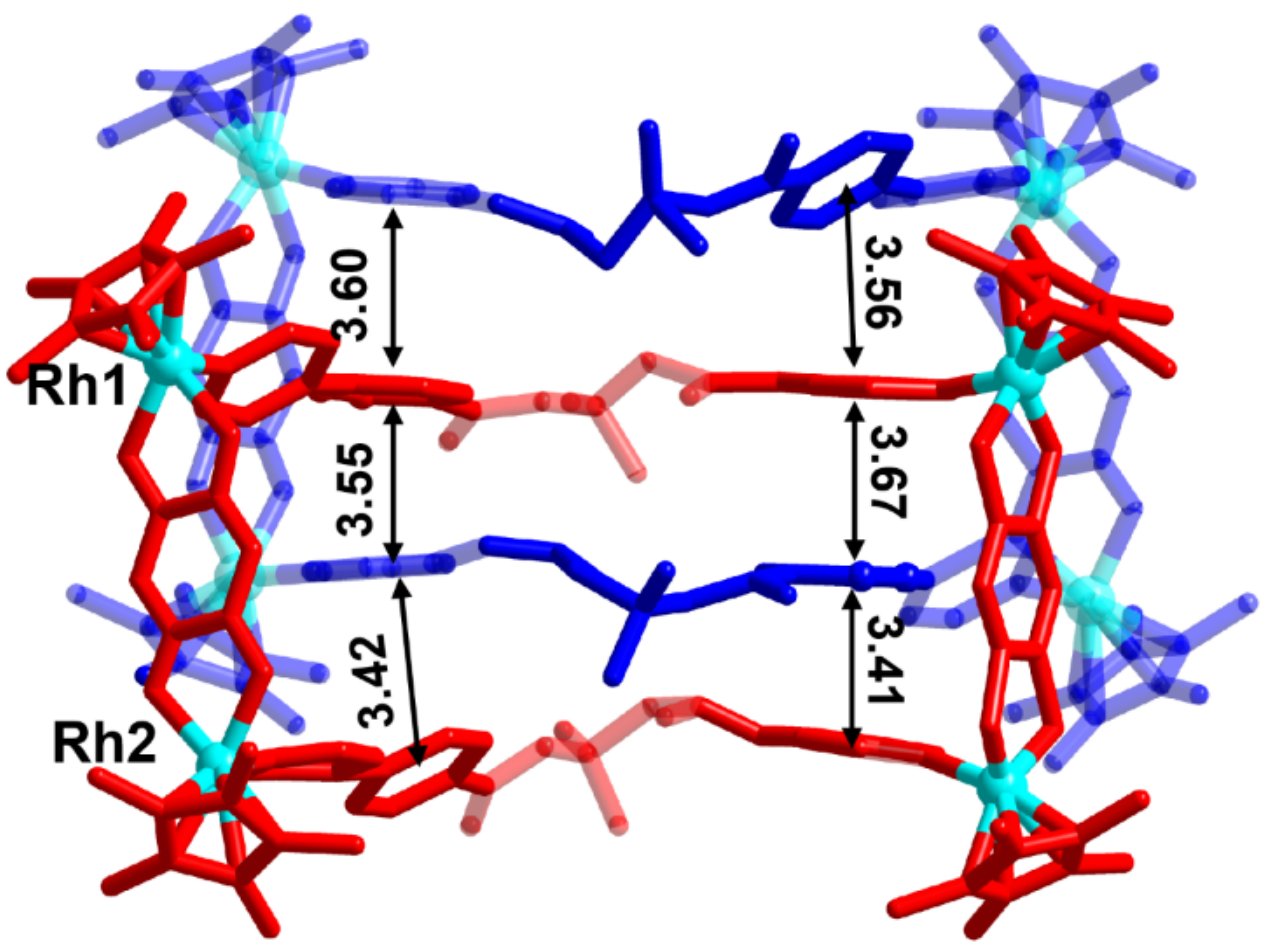

Fig. S14. The □-□ stacking interactions in **Rh-3** (unit: Å). Counteranions and hydrogen atoms are omitted for clarity. Color code: Rh, turquoise.

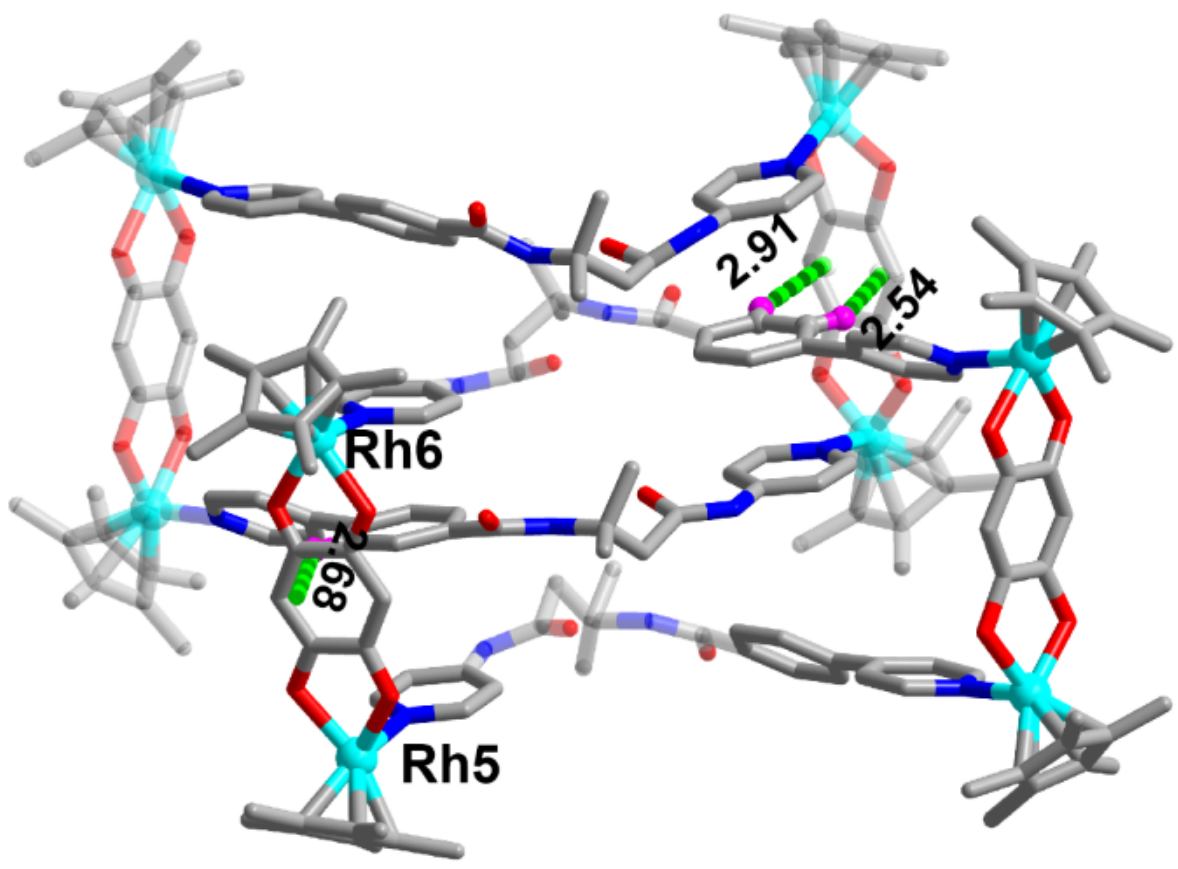

Fig. S15. The edge-to-face CH···□ interactions (bright green dotted lines) in **Rh-3** (unit: Å). Counteranions and non-essential hydrogen atoms are omitted for clarity. Color code: C, gray; H, pink; N, blue; O, red; Rh, turquoise.

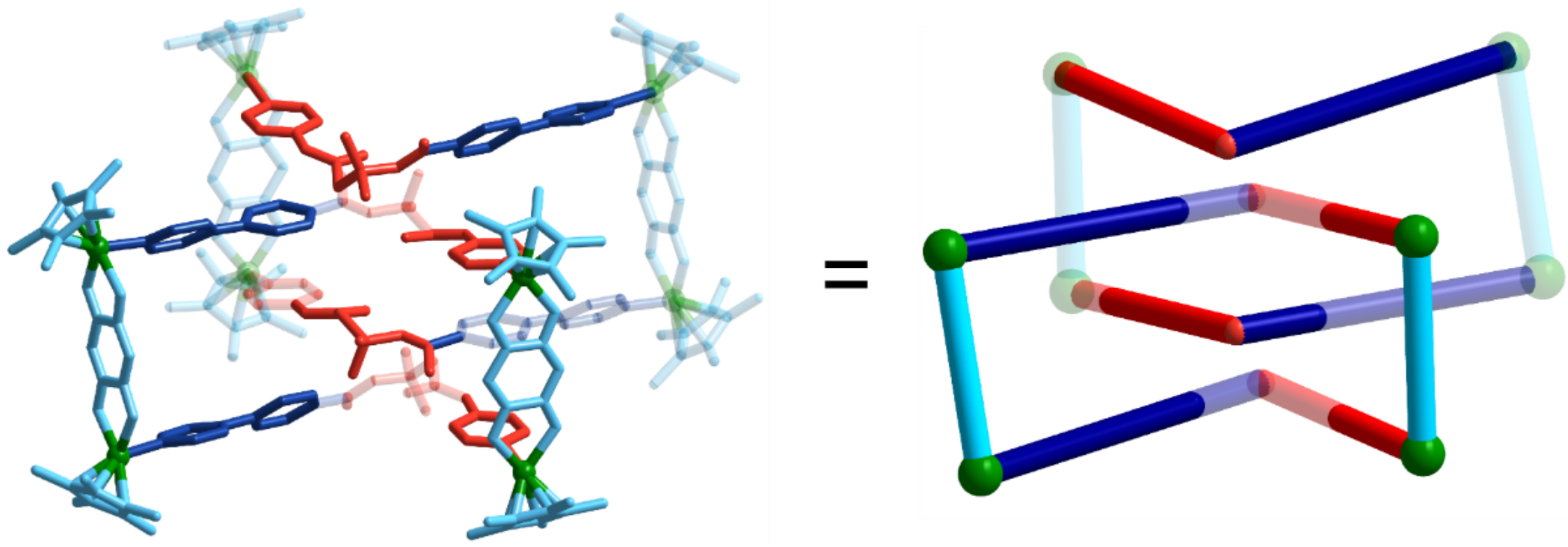

Fig. S16. Single-crystal structure and schematic representation of **Ir-3**, a non-interlocked nested dimer of two tetranuclear metallomacrocycles. Counteranions and hydrogen atoms are omitted for clarity.

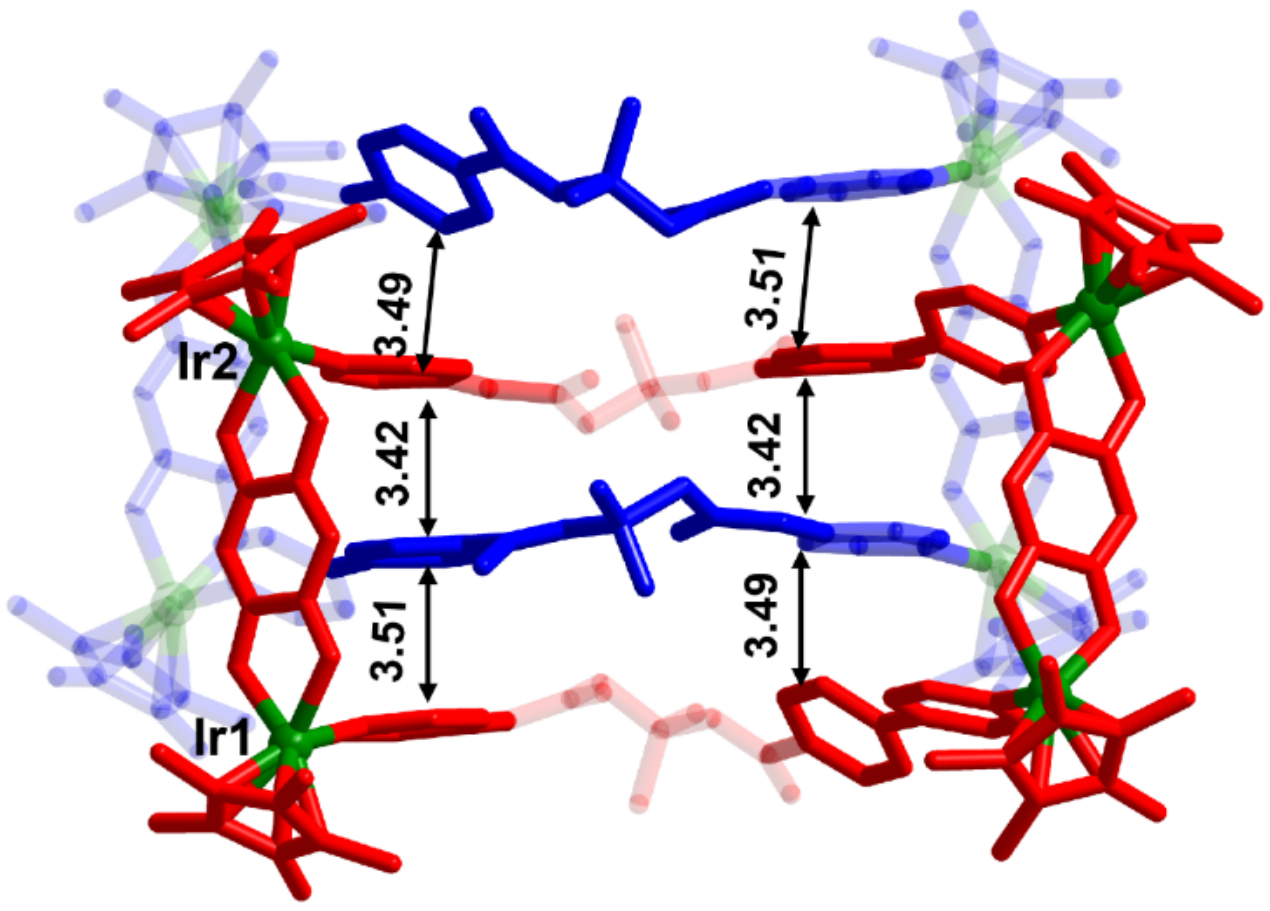

Fig. S17. The □-□ stacking interactions in **Ir-3** (unit: Å). Counteranions and hydrogen atoms are omitted for clarity. Color code: Ir, green.

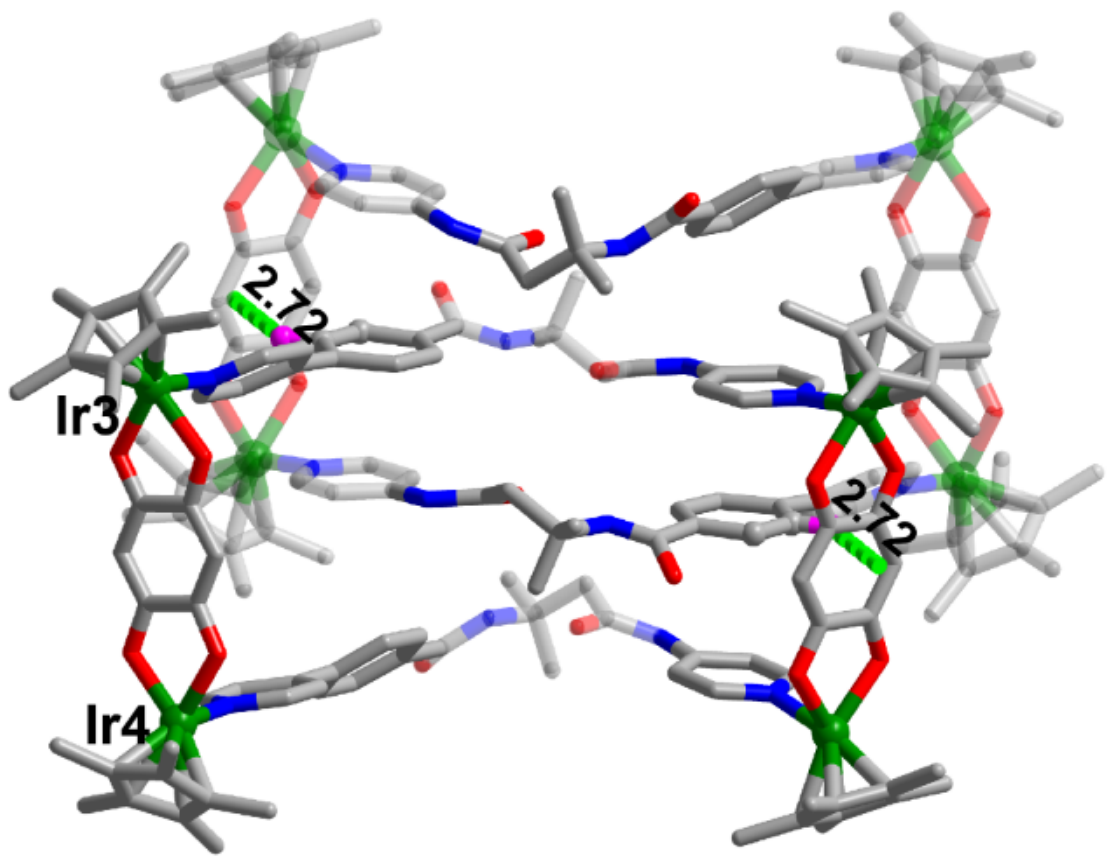

Fig. S18. The edge-to-face CH···□ interactions (bright green dotted lines) in **Ir-3** (unit: Å). Counteranions and non-essential hydrogen atoms are omitted for clarity. Color code: C, gray; H, pink; N, blue; O, red; Ir, green.

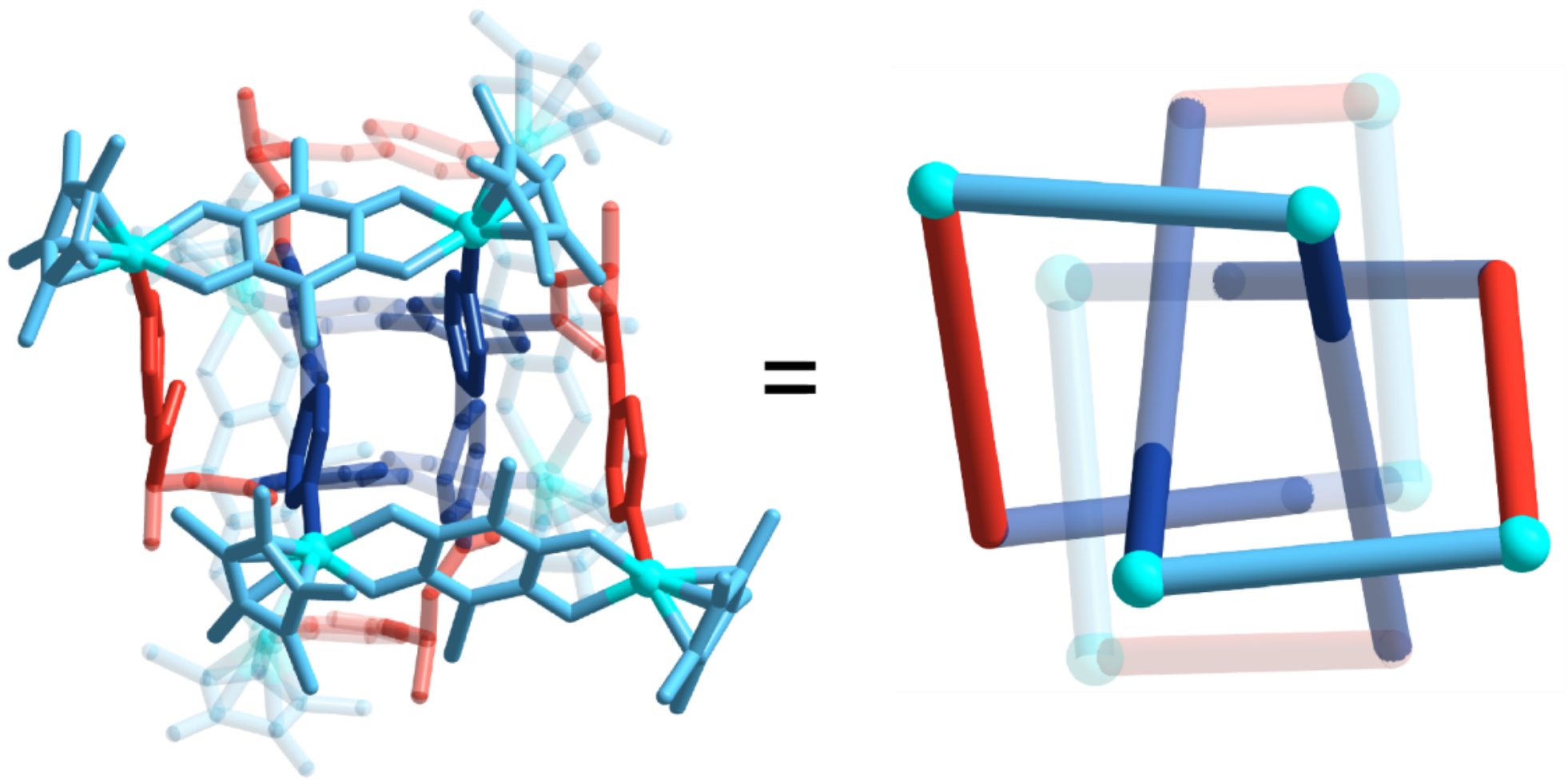

Fig. S19. Single-crystal structure and schematic representation of the figure-eight (4_1) knot **Rh-4Br*S***. Counteranions and hydrogen atoms are omitted for clarity.

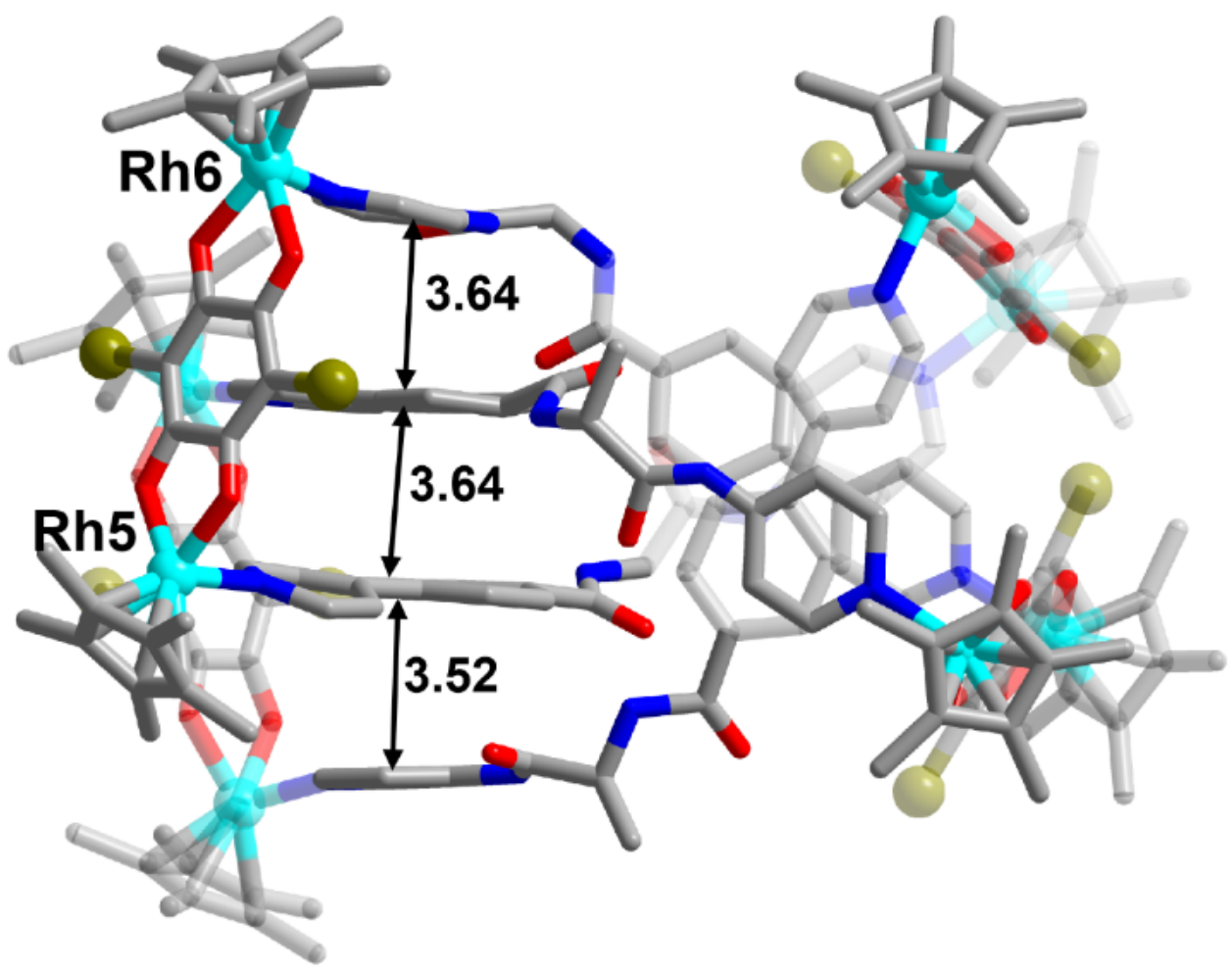

Fig. S20. The □-□ stacking interactions in **Rh-4Br*S*** (unit: Å). Counteranions and non-essential hydrogen atoms are omitted for clarity. Color code: C, gray; N, blue; O, red; Rh, turquoise; Br, dark yellow.

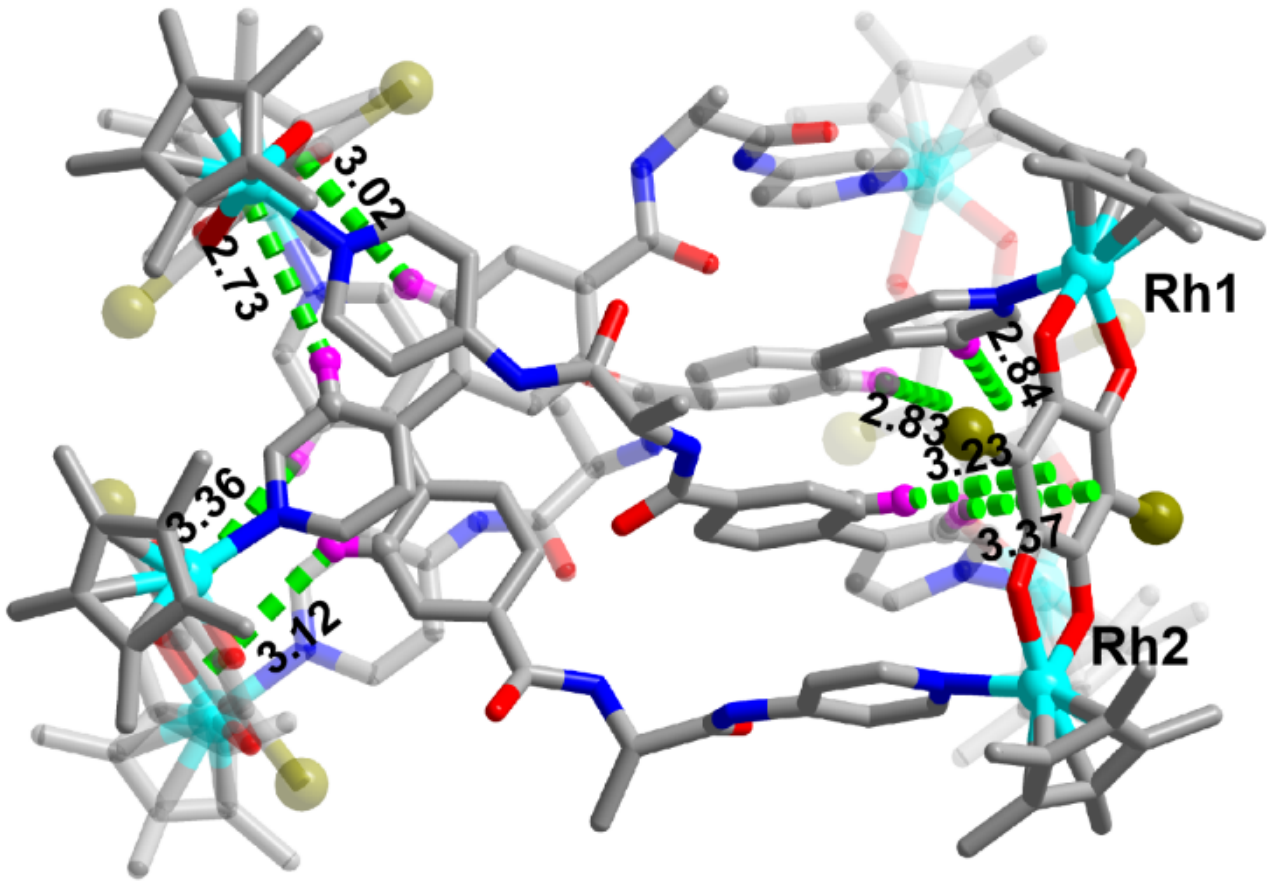

Fig. S21. The edge-to-face CH···π interactions (bright green dotted lines) in **Rh-4Br*S*** (unit: Å). Counteranions and non-essential hydrogen atoms are omitted for clarity. Color code: C, gray; H, pink; N, blue; O, red; Rh, turquoise; Br, dark yellow.

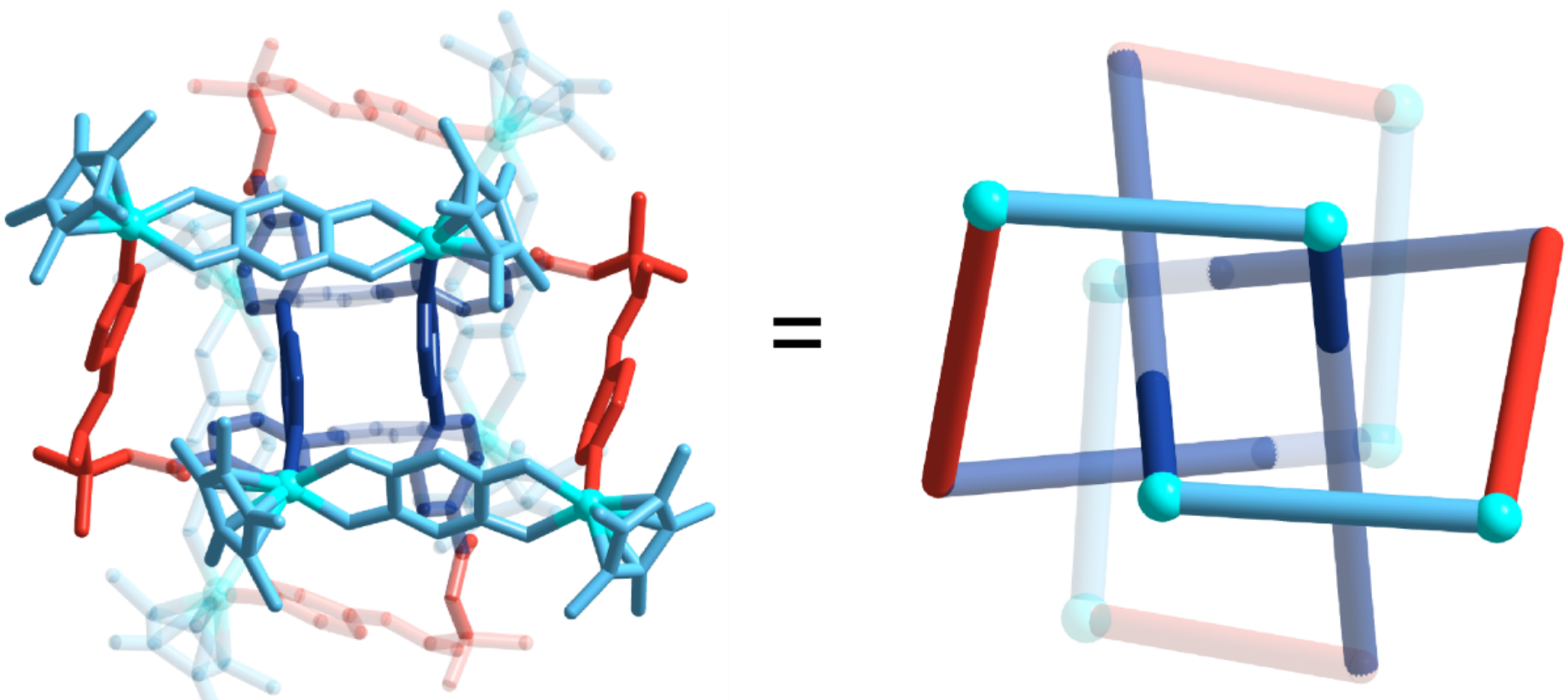

Fig. S22. Single-crystal structure and schematic representation of the figure-eight (4_1) knot **Rh-5**. Counteranions and hydrogen atoms are omitted for clarity.

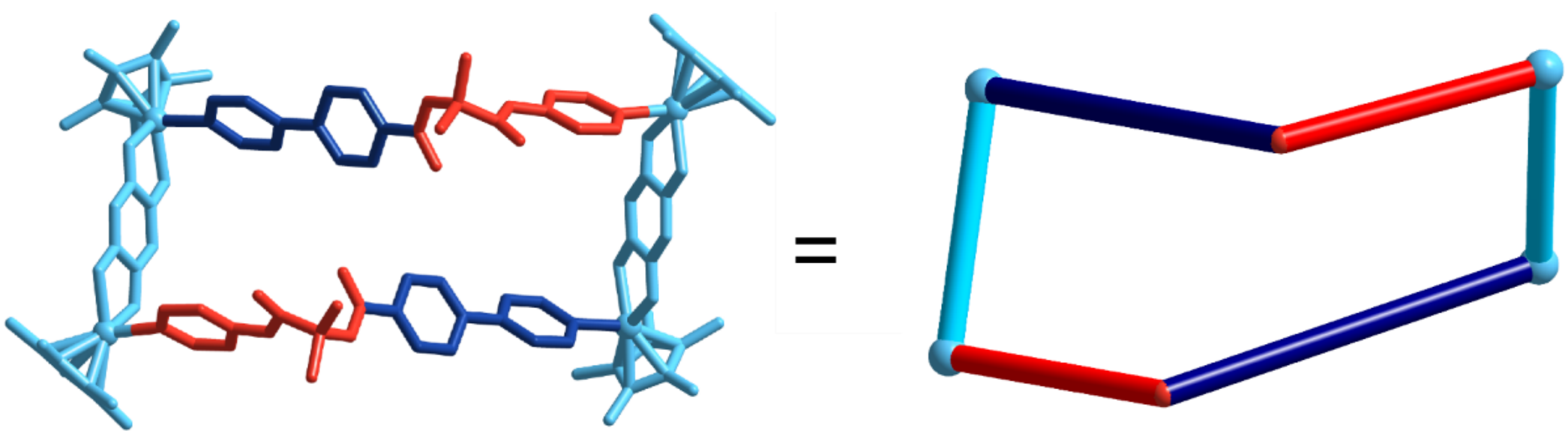

Fig. S23. Single-crystal structure and schematic representation of the tetranuclear metallomacrocycle **Rh-5′**. Counteranions and hydrogen atoms are omitted for clarity.

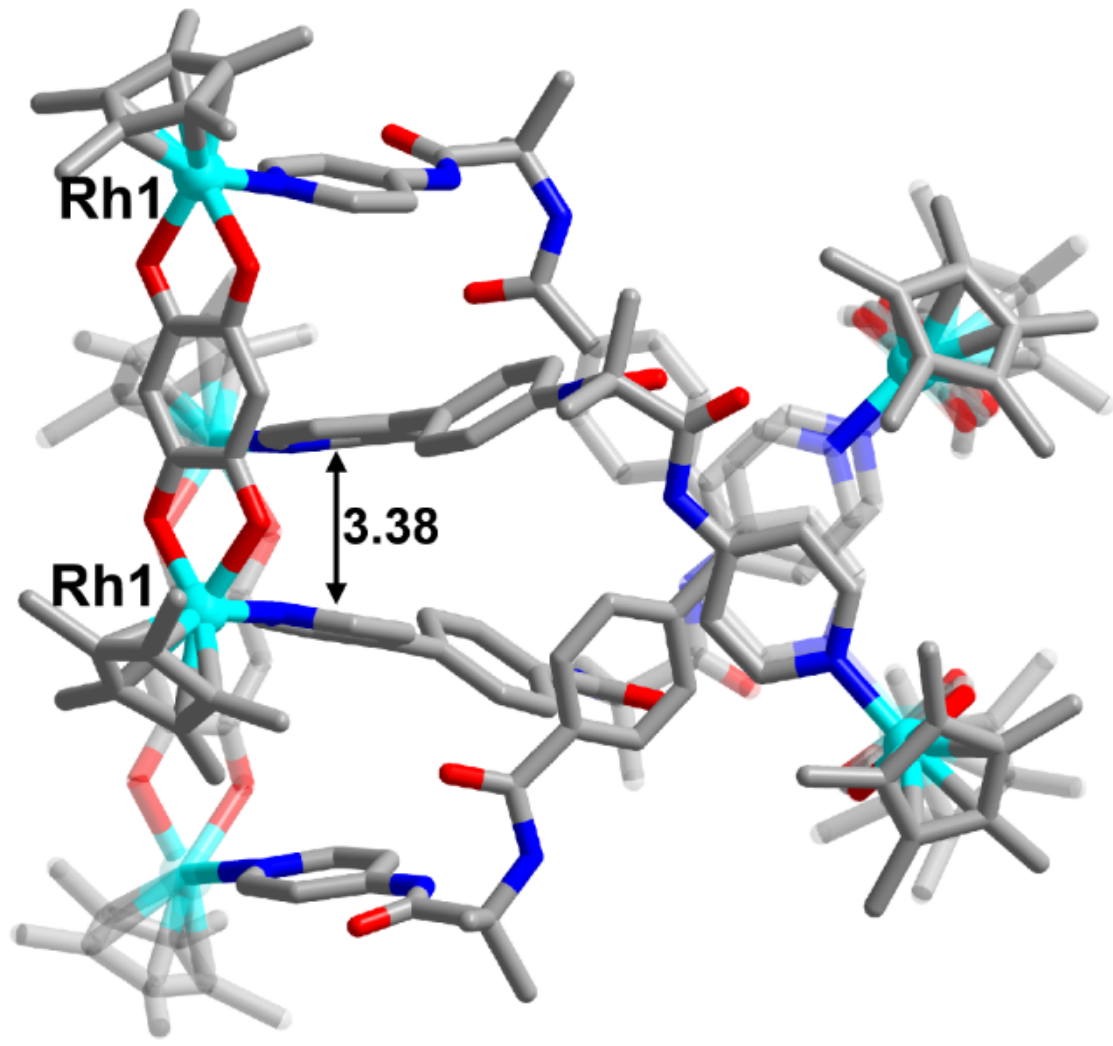

Fig. S24. The □-□ stacking interactions in **Rh-5** (unit: Å). Counteranions and non-essential hydrogen atoms are omitted for clarity. Color code: C, gray; N, blue; O, red; Rh, turquoise.

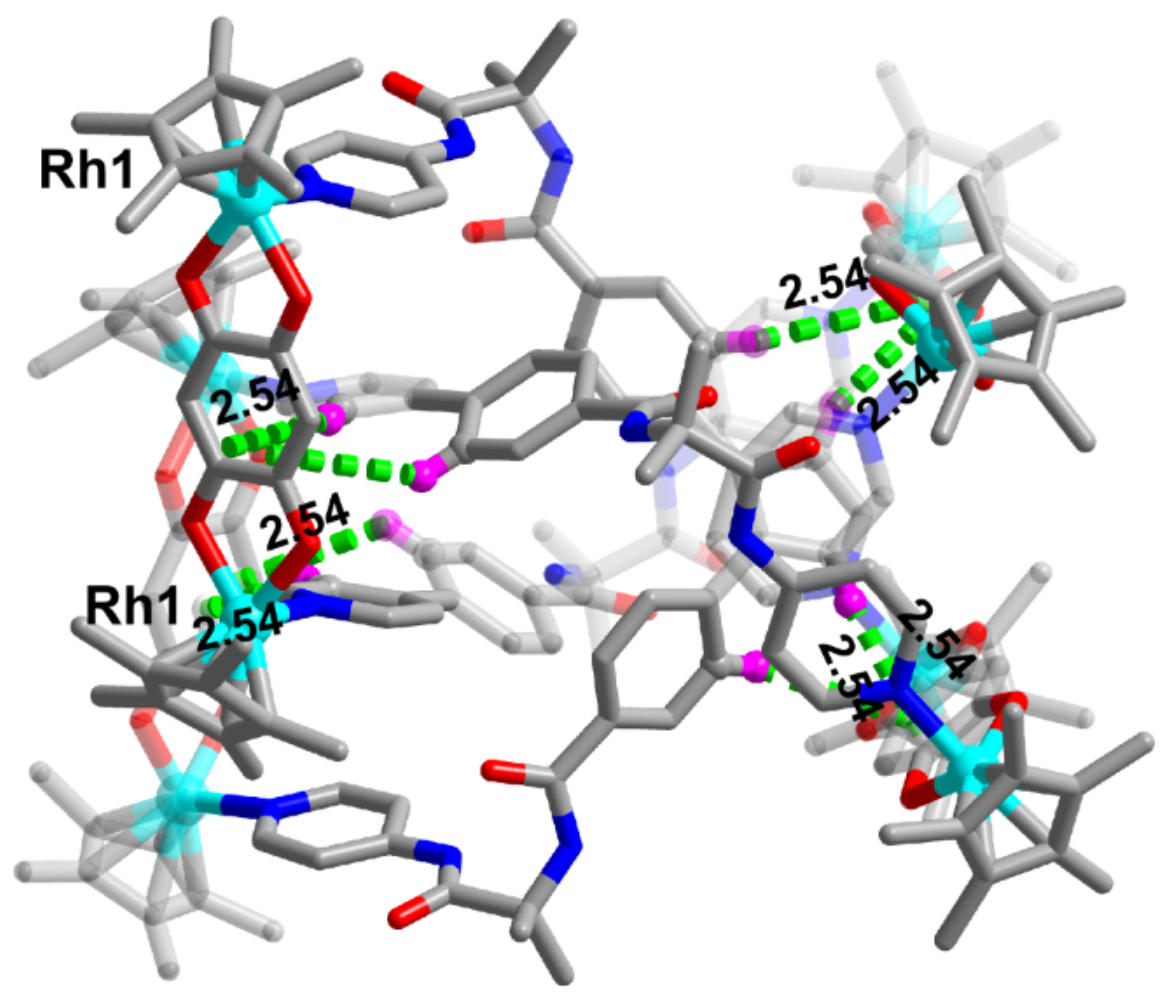

Fig. S25. The edge-to-face CH···□ interactions (bright green dotted lines) in **Rh-5** (unit: Å). Counteranions and non-essential hydrogen atoms are omitted for clarity. Color code: C, gray; H, pink; N, blue; O, red; Rh, turquoise.

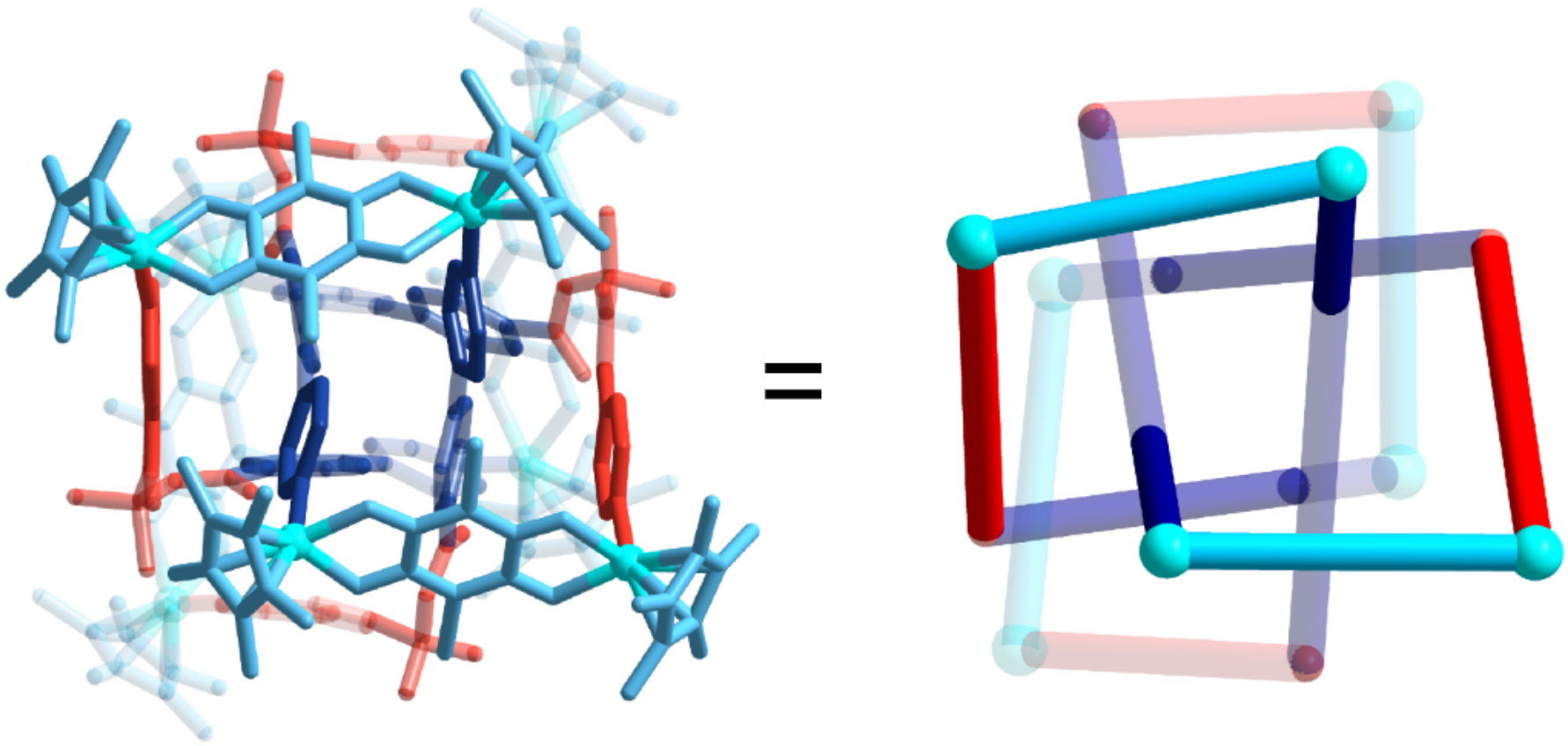

Fig. S26. Single-crystal structure and schematic representation of the figure-eight (4_1) knot **Rh-5Br**. Counteranions and hydrogen atoms are omitted for clarity.

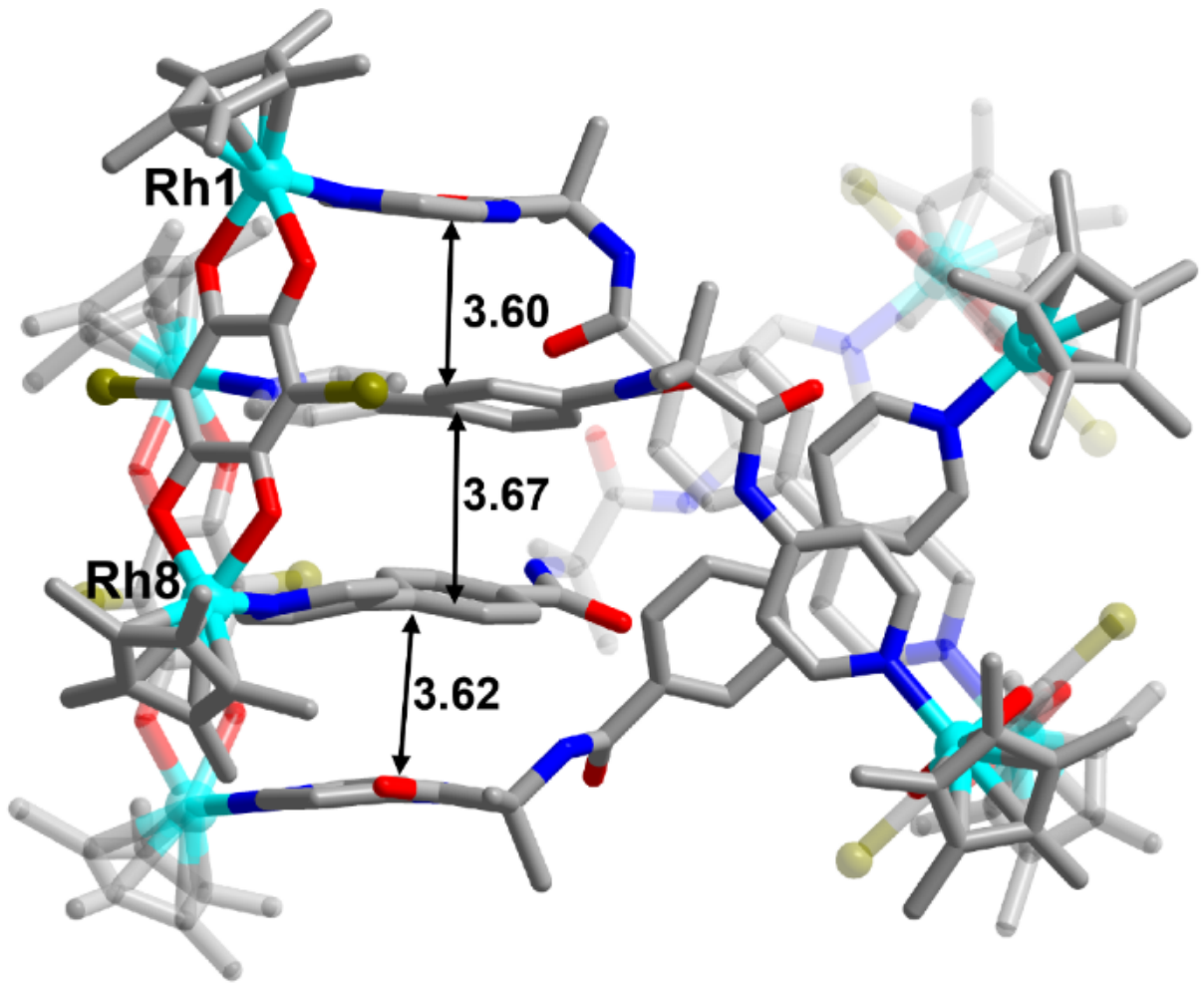

Fig. S27. The □-□ stacking interactions in **Rh-5Br** (unit: Å). Counteranions and non-essential hydrogen atoms are omitted for clarity. Color code: C, gray; N, blue; O, red; Rh, turquoise; Br, dark yellow.

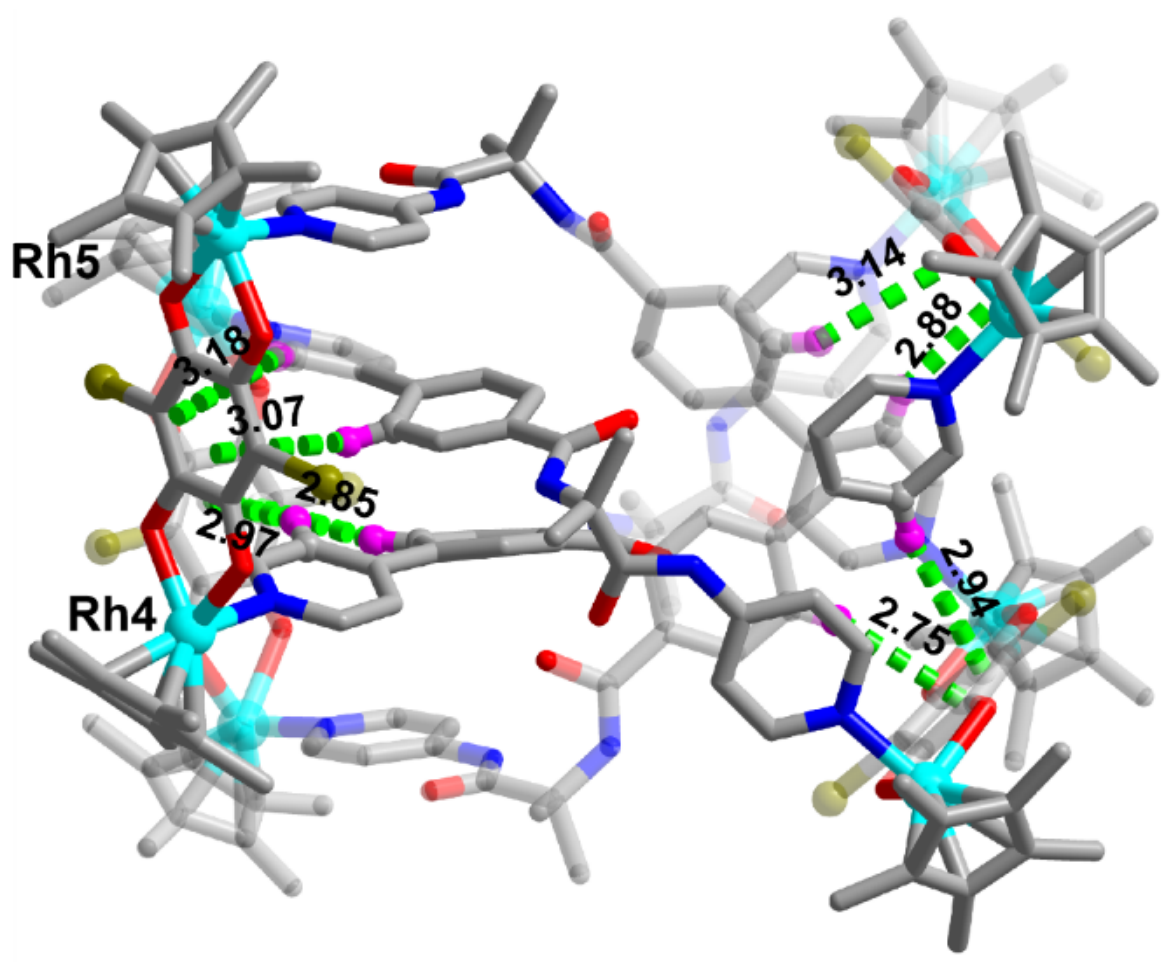

Fig. S28. The edge-to-face CH···□ interactions (bright green dotted lines) in **Rh-5Br** (unit: Å). Counteranions and non-essential hydrogen atoms are omitted for clarity. Color code: C, gray; H, pink; N, blue; O, red; Rh, turquoise; Br, dark yellow.

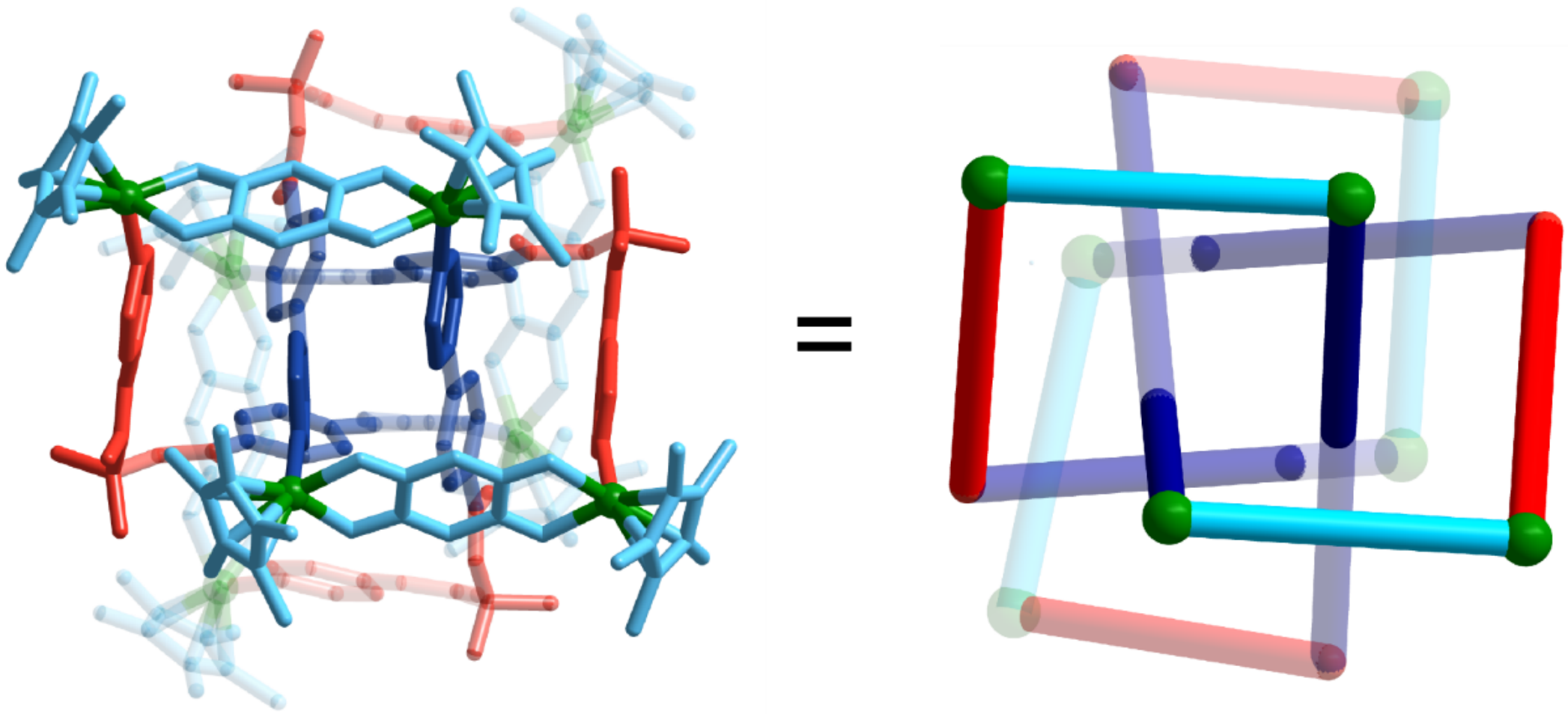

Fig. S29. Single-crystal structure and schematic representation of the figure-eight (4_1) knot **Ir-5**. Counteranions and hydrogen atoms are omitted for clarity.

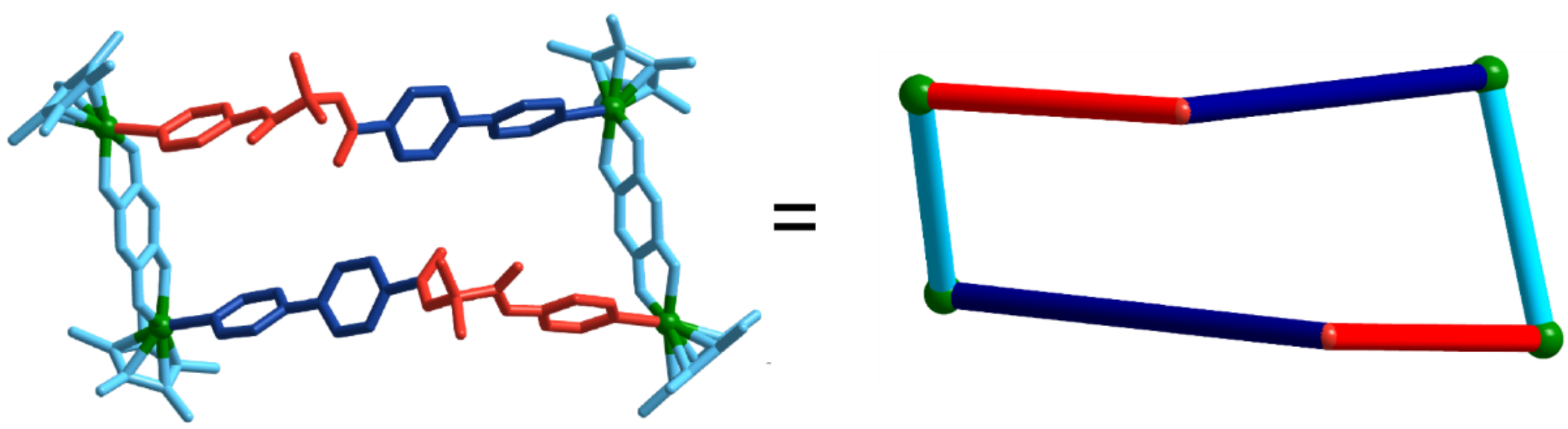

Fig. S30. Single-crystal structure and schematic representation of the tetranuclear metallomacrocycle **Ir-5′**. Counteranions and hydrogen atoms are omitted for clarity.

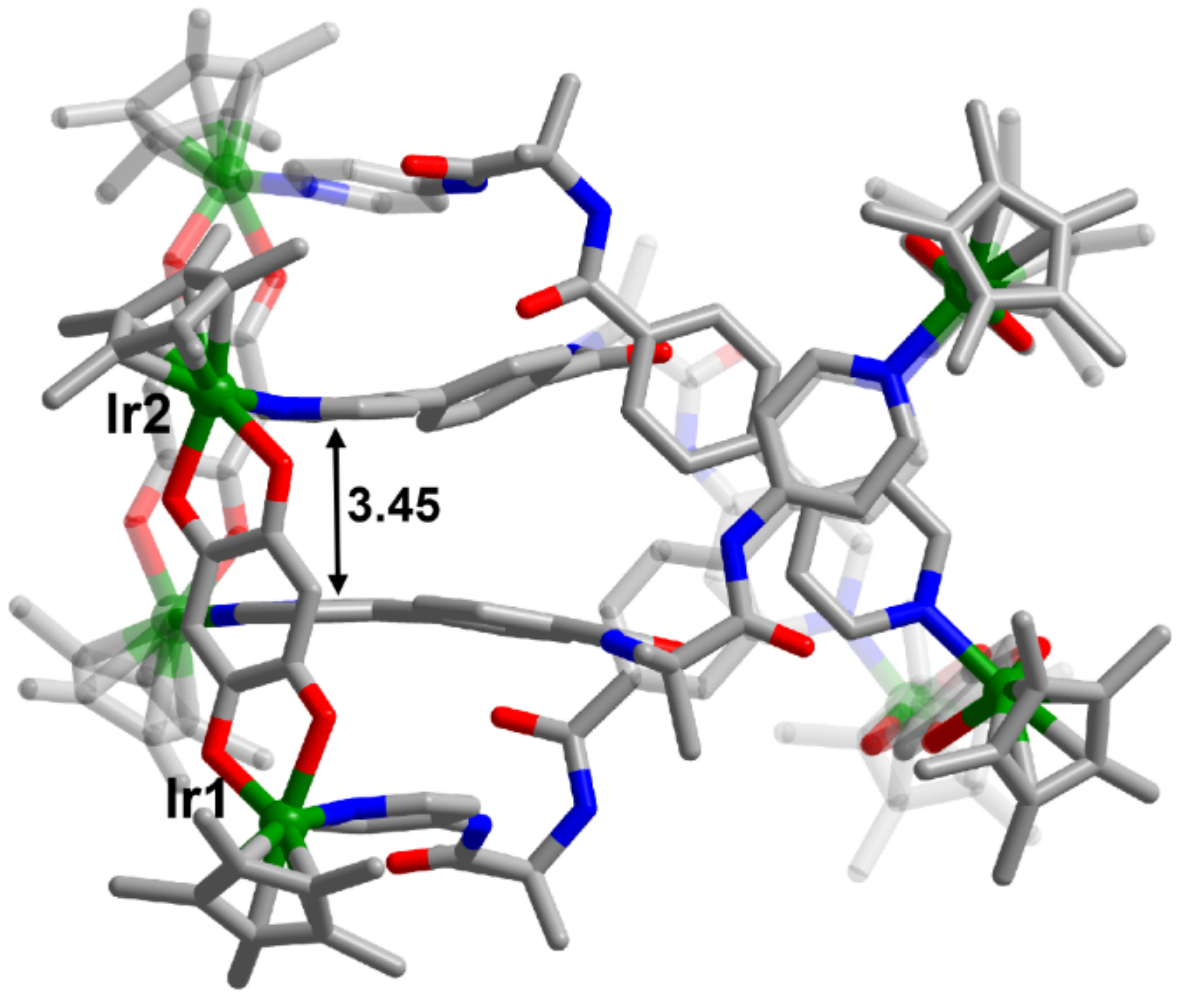

Fig. S31. The π-π stacking interactions in **Ir-5** (unit: Å). Counteranions and non-essential hydrogen atoms are omitted for clarity. Color code: C, gray; N, blue; O, red; Ir, green.

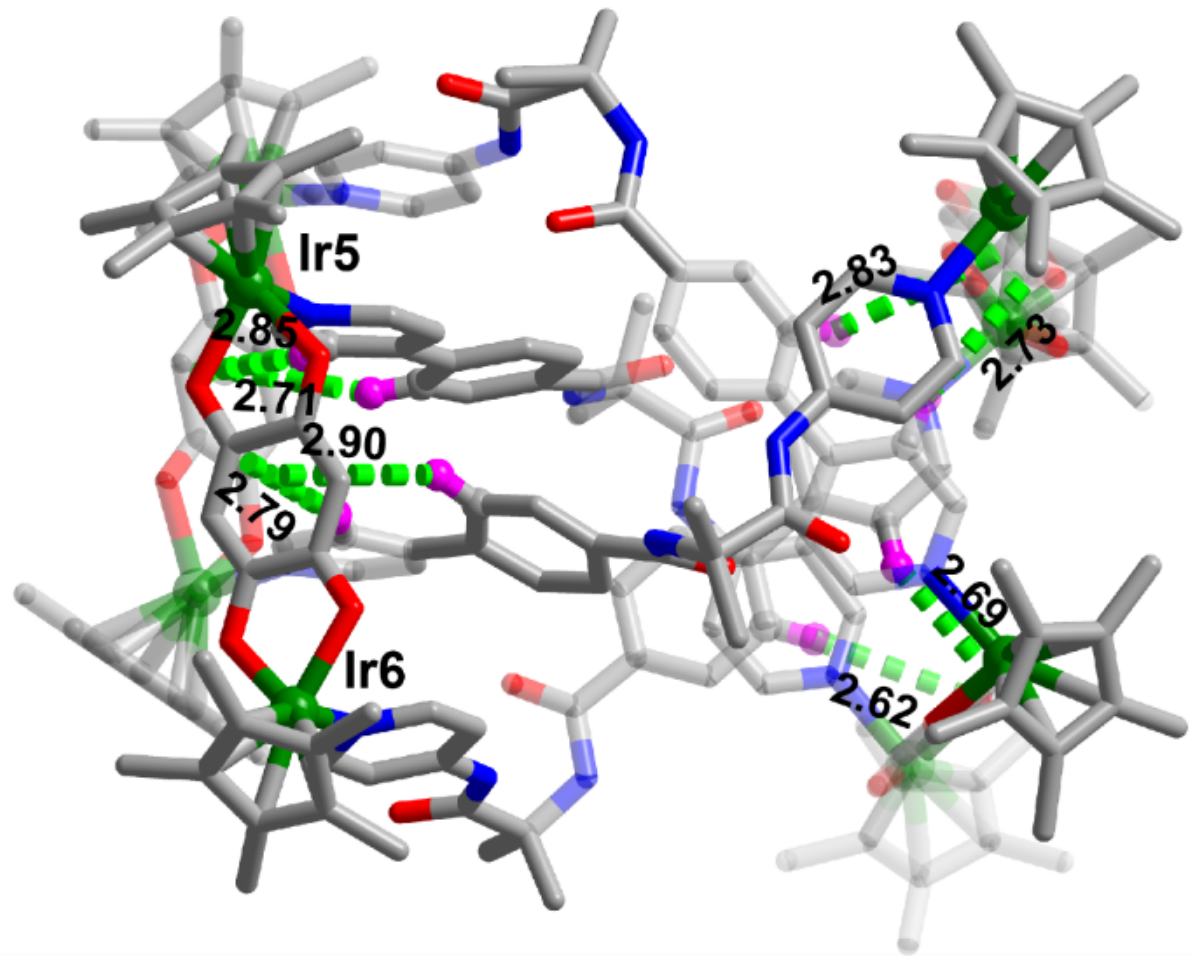

Fig. S32. The edge-to-face CH···π interactions (bright green dotted lines) in **Ir-5** (unit: Å). Counteranions and non-essential hydrogen atoms are omitted for clarity. Color code: C, gray; H, pink; N, blue; O, red; Ir, green.

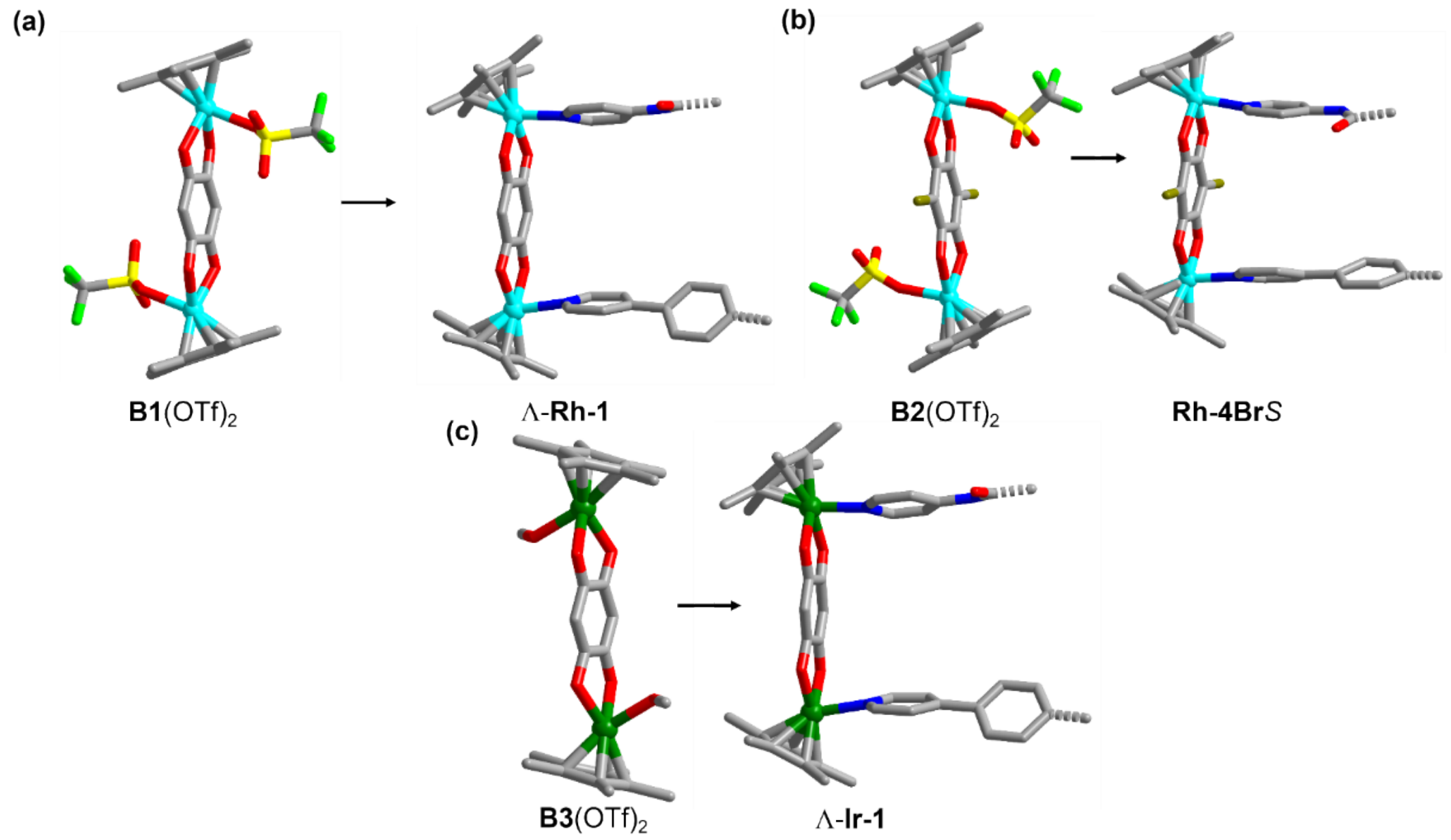

Fig. S33. Comparison of the relative orientations of the Cp* groups in the crystal structures of the isolated binuclear precursors and representative assemblies: (a) **B1**(OTf)$_2$ and Λ-**Rh-1**; (b) **B2**(OTf)$_2$ and **Rh-4Br*S***; and (c) **B3**(OTf)$_2$ and Λ-**Ir-1**. The Cp* groups adopt *anti* arrangements in the isolated precursors and *syn* arrangements in the assemblies. Selected structural fragments are shown for clarity.

6.NMR Spectra

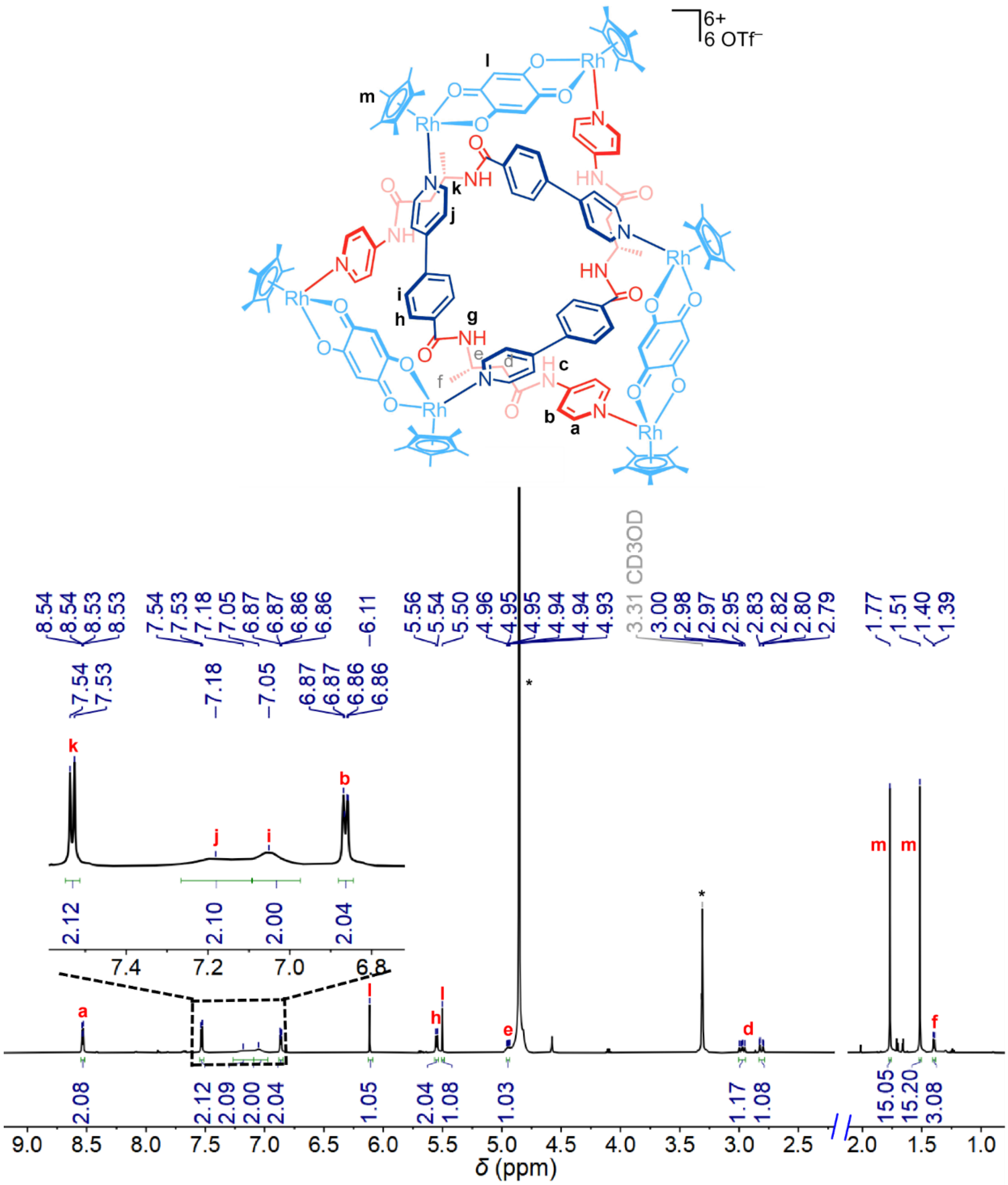

Fig. S34. ^{1}H NMR spectrum of Λ-**Rh-1** in CD_3OD (600 MHz, *c* = 6.0 mM, with respect to trefoil knots).

The hydrogen protons of NH on the ligand lose their NMR signal due to the exchange with D in CD_3OD.

* = solvent molecules.

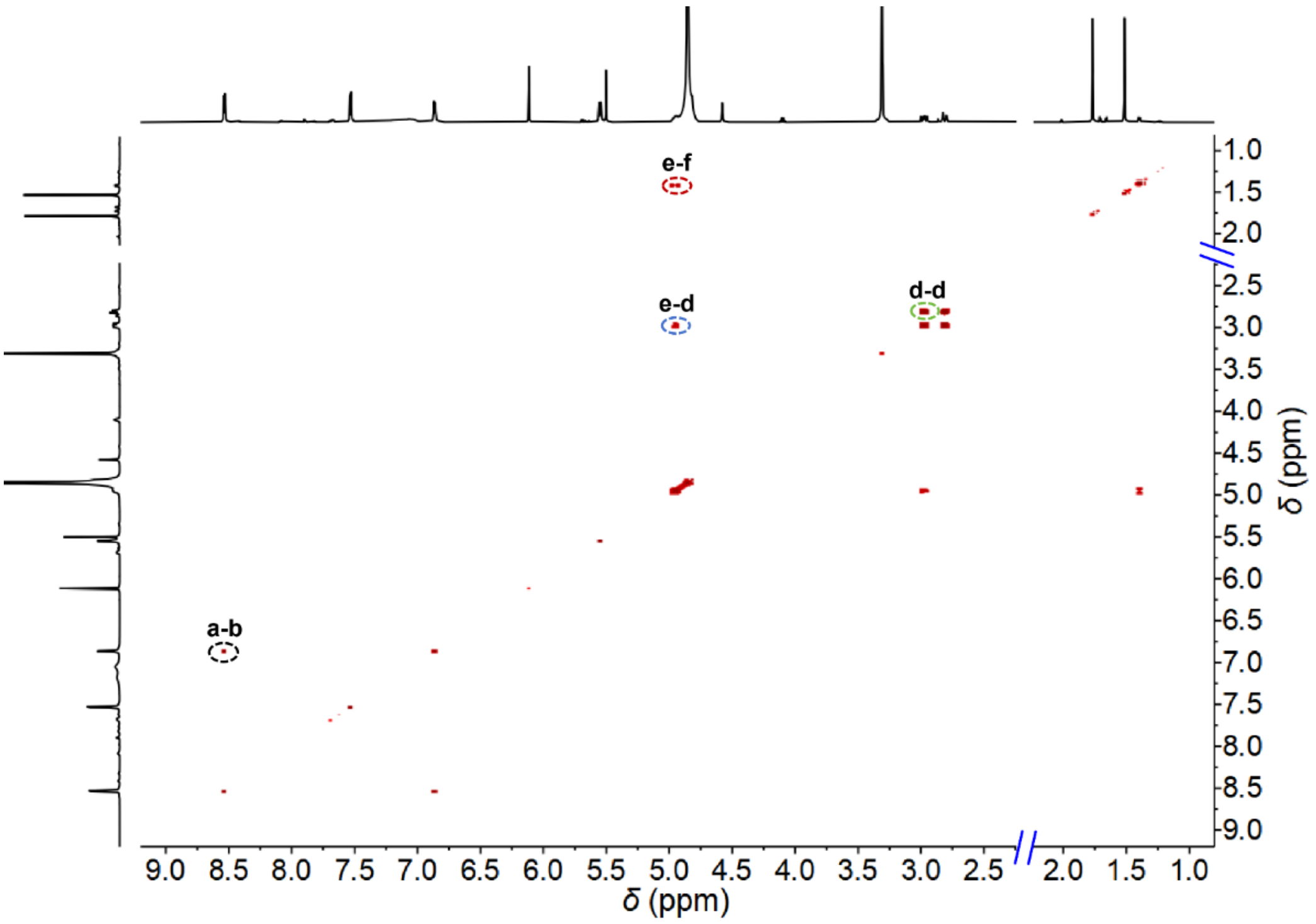

Fig. S35. ^{1}H-^{1}H COSY spectrum of Λ-**Rh-1** in CD_3OD (600 MHz, c = 6.0 mM, with respect to trefoil knots).

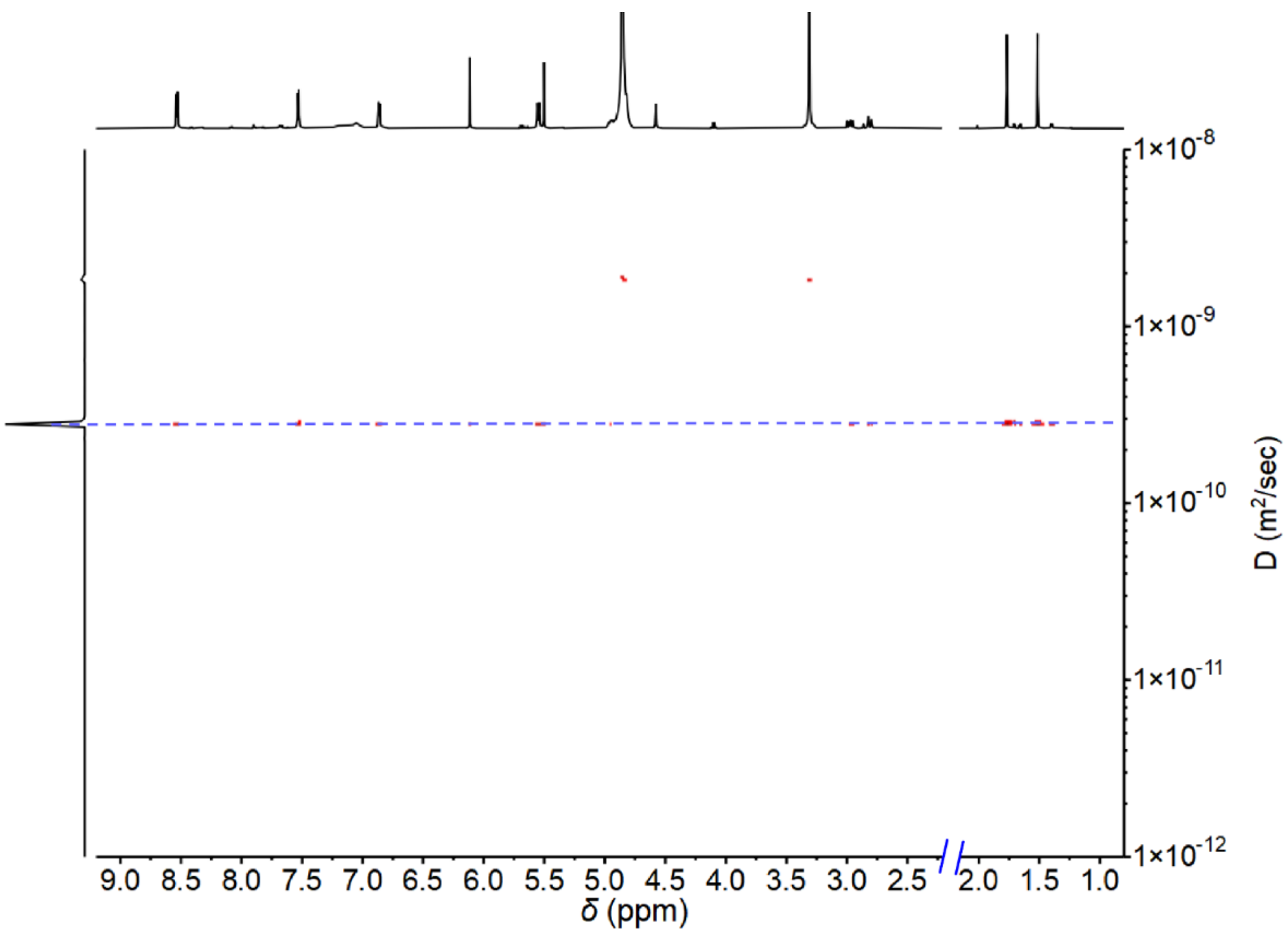

Fig. S36. ^{1}H DOSY spectrum of Λ-**Rh-1** in CD_3OD (600 MHz, c = 6.0 mM, where $c = c_{Rh}/6$). The assigned trefoil-knot resonances exhibit a diffusion coefficient of $D = 2.83 \times 10^{-10}$ m^2 s^{-1}.

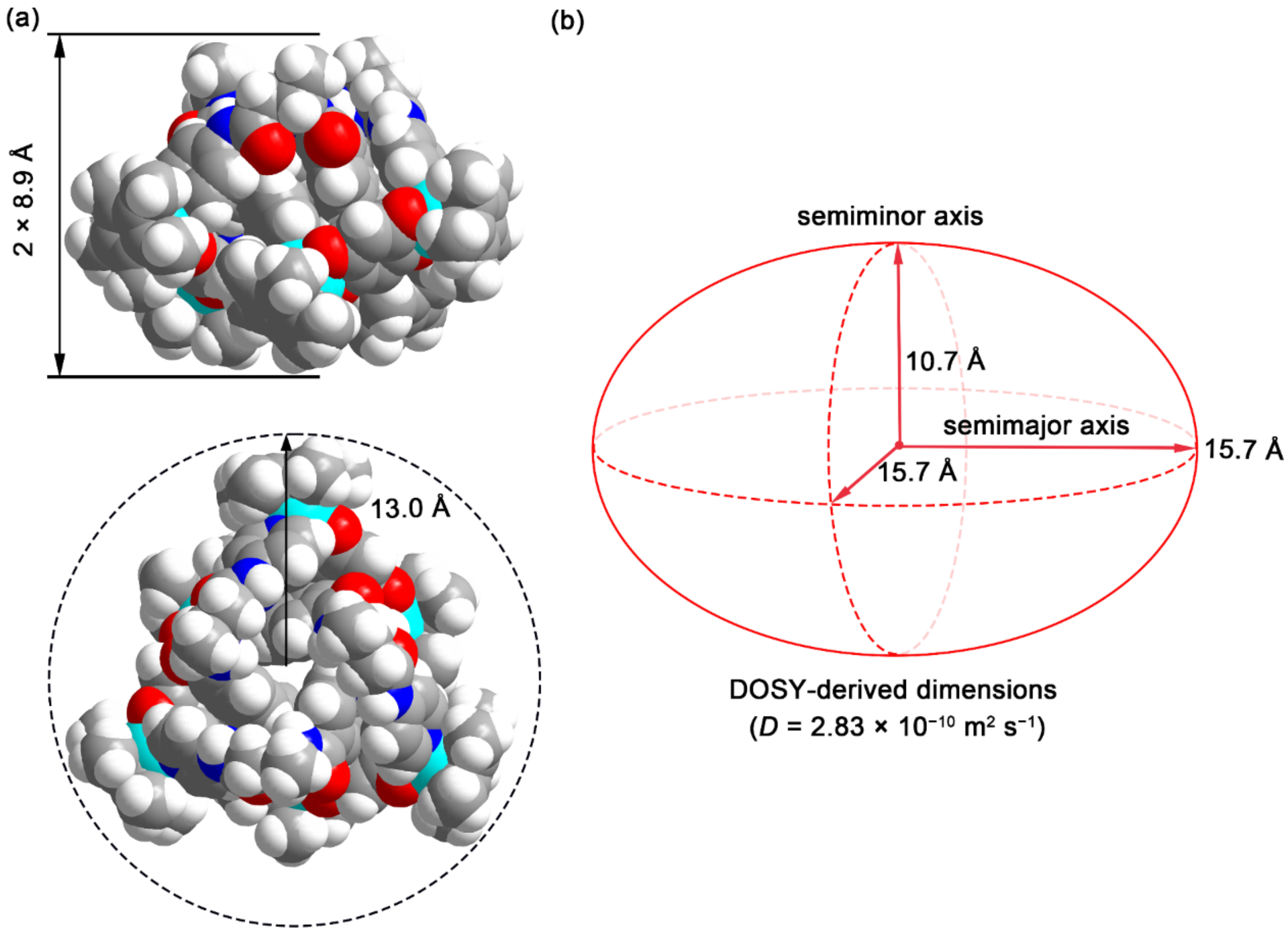

Fig. S37. Comparison of crystallographic and hydrodynamic dimensions for the trefoil knot Λ-**Rh-1** species in solution. (a) Crystallographic geometric reference extracted from the crystal structure of trefoil knot Λ-**Rh-1** (radius × half-height, 13.0 Å × 8.9 Å). (b) Hydrodynamic dimensions estimated from the DOSY diffusion coefficient of the trefoil knot Λ-**Rh-1** (D = 2.83 × 10^{-10} m^2 s^{-1}, Fig. S36) using a modified Stokes–Einstein treatment for an oblate spheroid model[4,5] (semimajor axis × semiminor axis, 15.7 Å × 10.7 Å). The estimated hydrodynamic dimensions are comparable to those of the trefoil knot Λ-**Rh-1** extracted from the crystal structure.

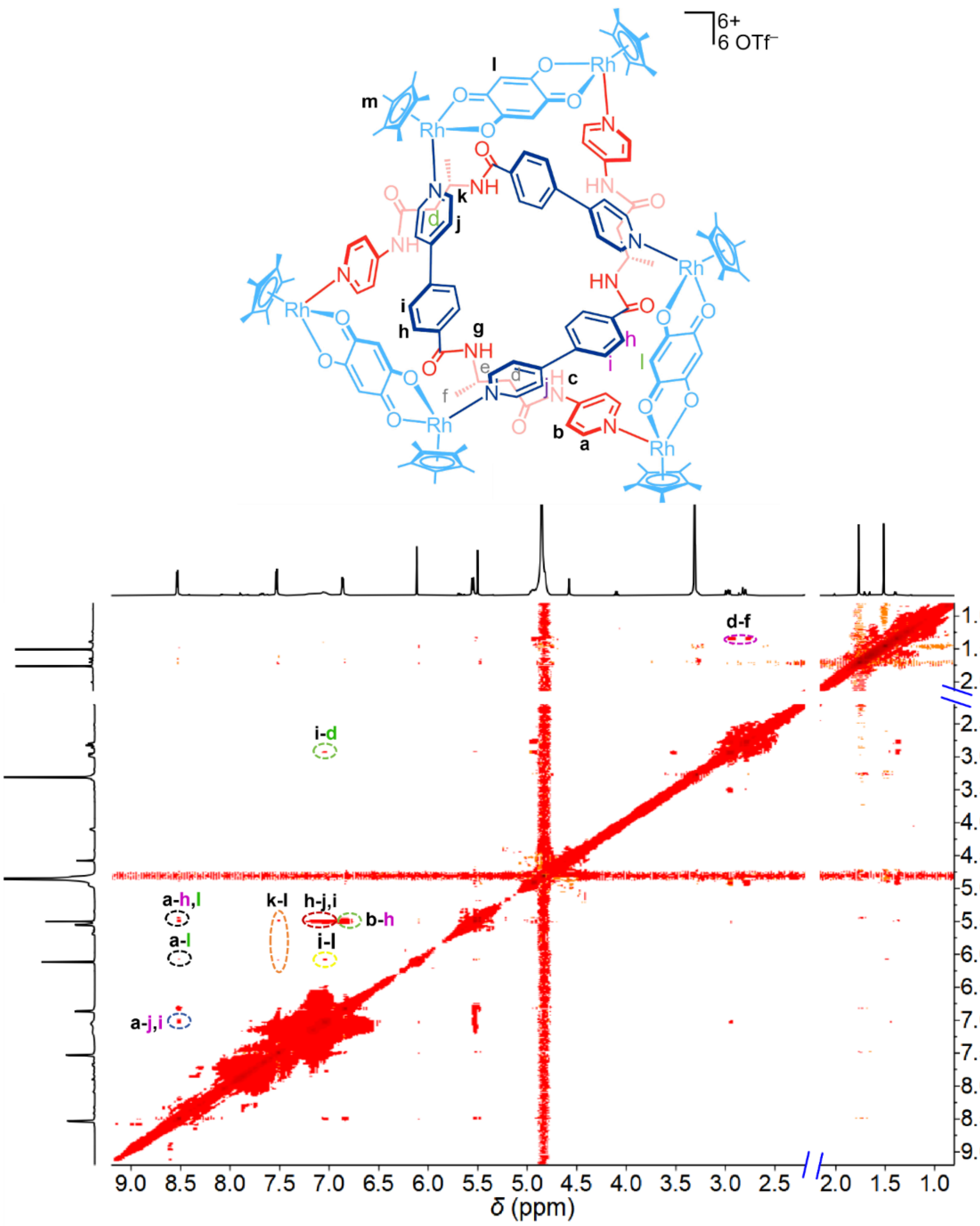

Fig. S38. ^1H-^1H NOESY NMR (600 MHz, CD_3OD, c = 6.0 mM) for Λ-**Rh-1**.

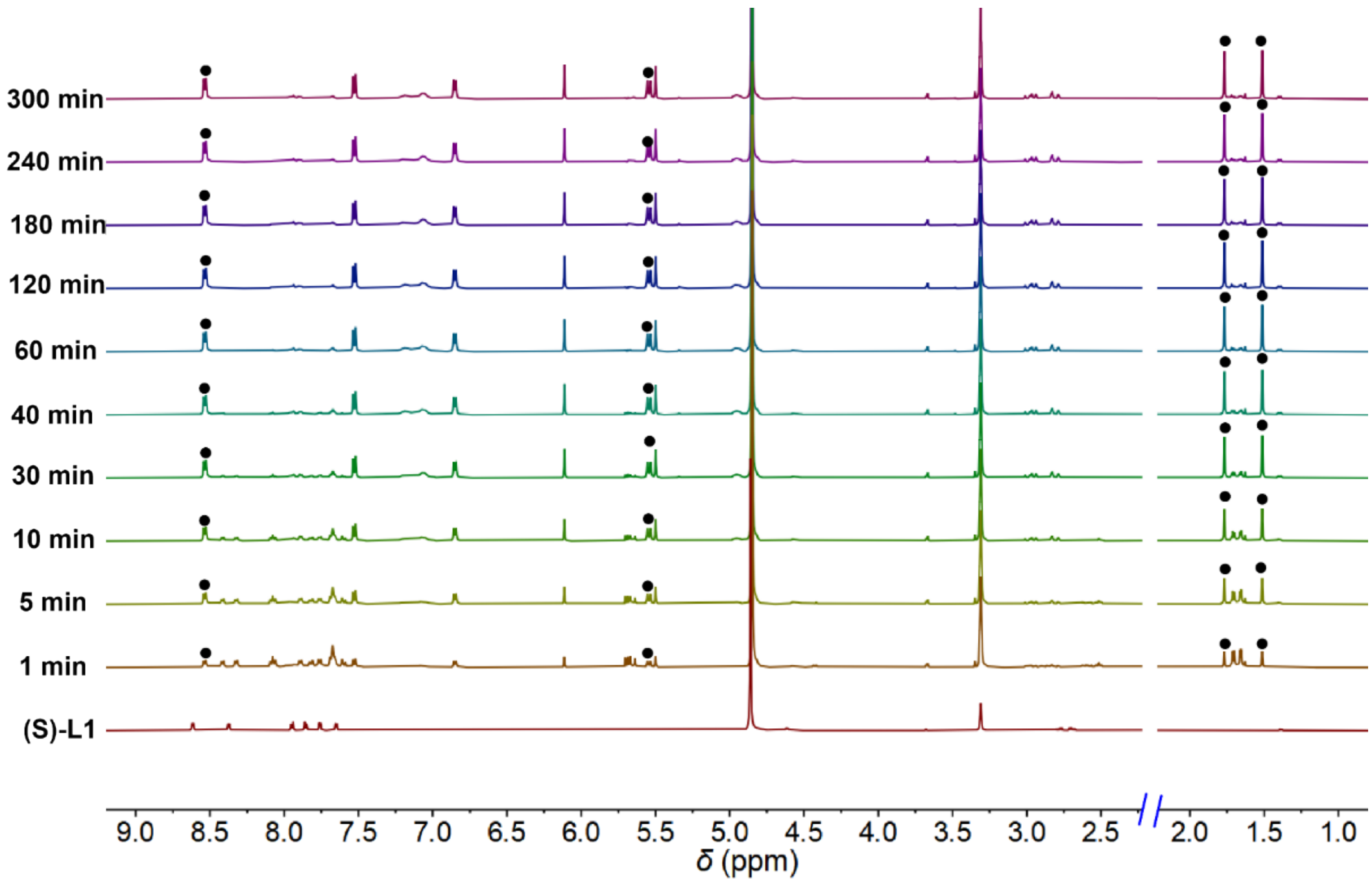

Fig. S39. Time-dependent ^{1}H NMR spectra monitoring self-assembly of (*S*)-**L1** with **B1**($OTf)_2$ to form Λ-**Rh-1** (400 MHz, CD_3OD, room temperature; formal knot concentration = 6.0 mM). Formation is largely complete after approximately 60 min. Selected resonances corresponding to the trefoil-knot assemblies are highlighted with black circles.

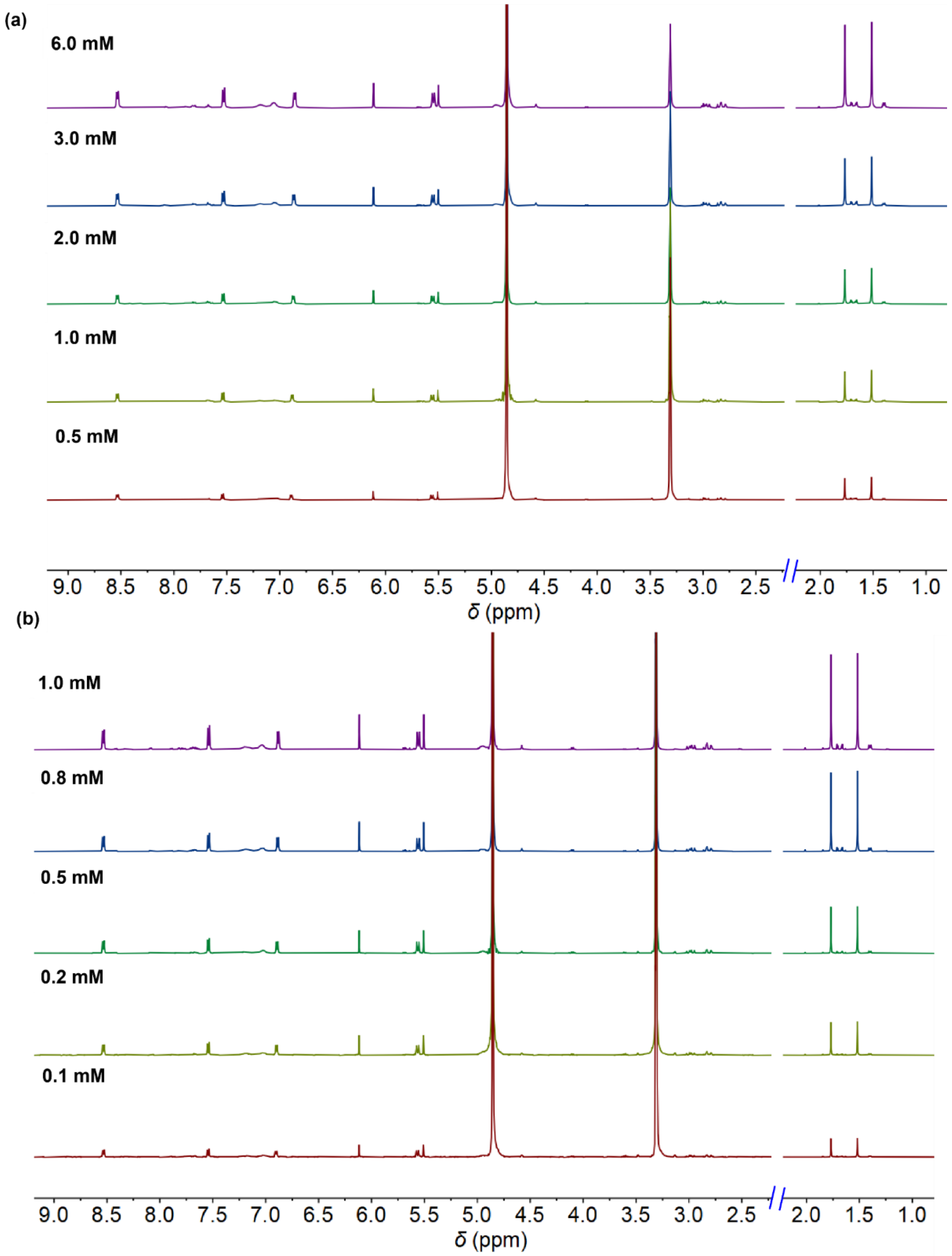

Fig. S40. Concentration-dependent ^{1}H NMR spectra of Λ-**Rh-1** (400 MHz, CD_3OD): (a) $c = 0.5–6.0$ mM and (b) $c = 0.1–1.0$ mM. The spectra show no substantial changes in the positions or relative intensities of the assigned assembly resonances over the concentration range examined.

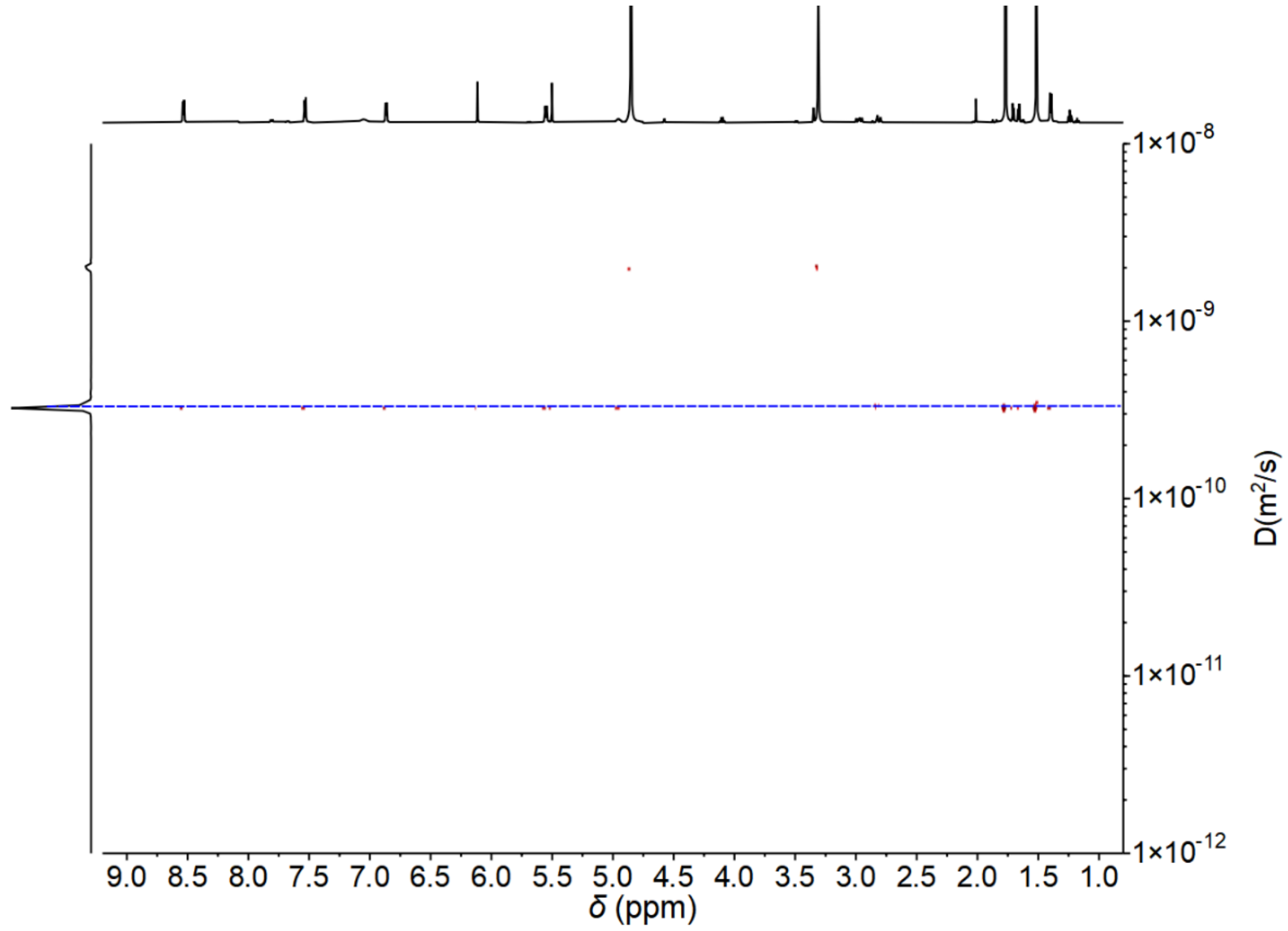

Fig. S41. ^{1}H DOSY spectrum of **Λ-Rh-1** in CD_3OD (600 MHz, c = 3.0 mM, where $c = c_{Rh}/6$). The assigned trefoil-knot resonances exhibit a diffusion coefficient of $D = 3.23 \times 10^{-10}$ m^2 s^{-1}.

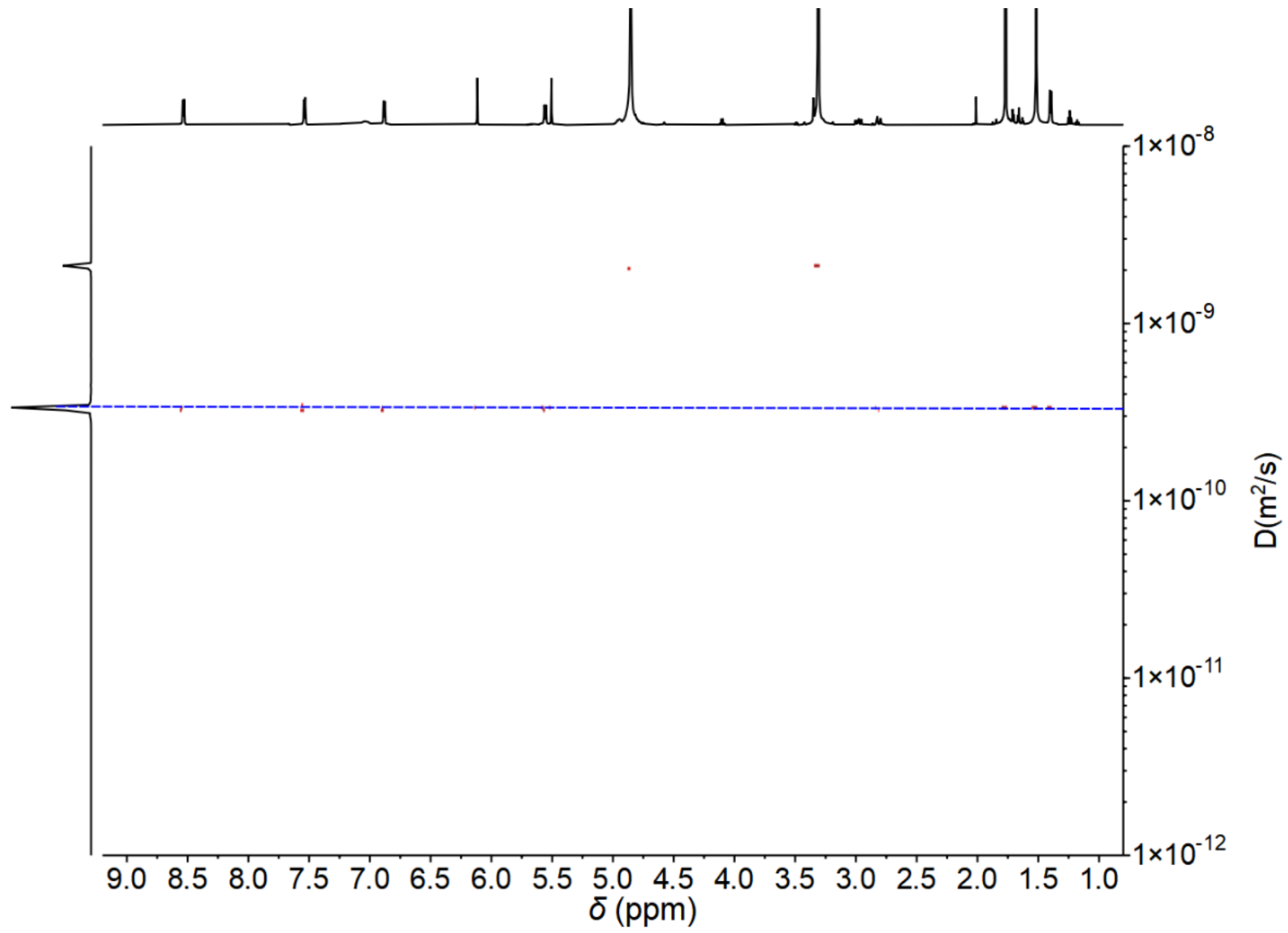

Fig. S42. ^{1}H DOSY spectrum of Λ-**Rh-1** in CD_3OD (600 MHz, c = 1.0 mM, where c = $c_{Rh}/6$). The assigned trefoil-knot resonances exhibit a diffusion coefficient of D = 3.35×10^{-10} m^2 s^{-1}.

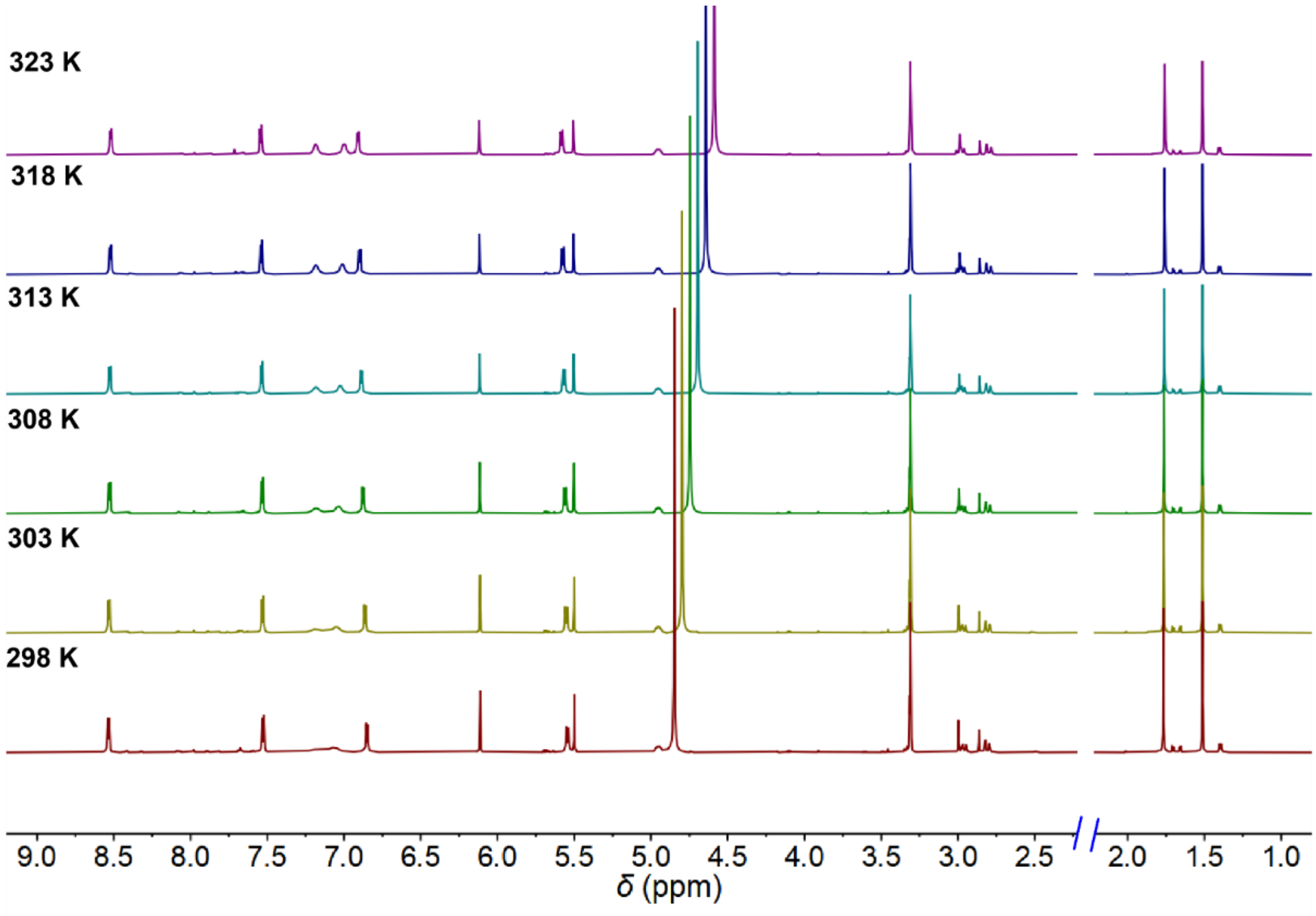

Fig. S43. Variable-temperature ^{1}H NMR spectra of Λ-**Rh-1** in CD_3OD (600 MHz, 298–323 K; c = 6.0 mM). The spectra show no substantial changes over the temperature range examined.

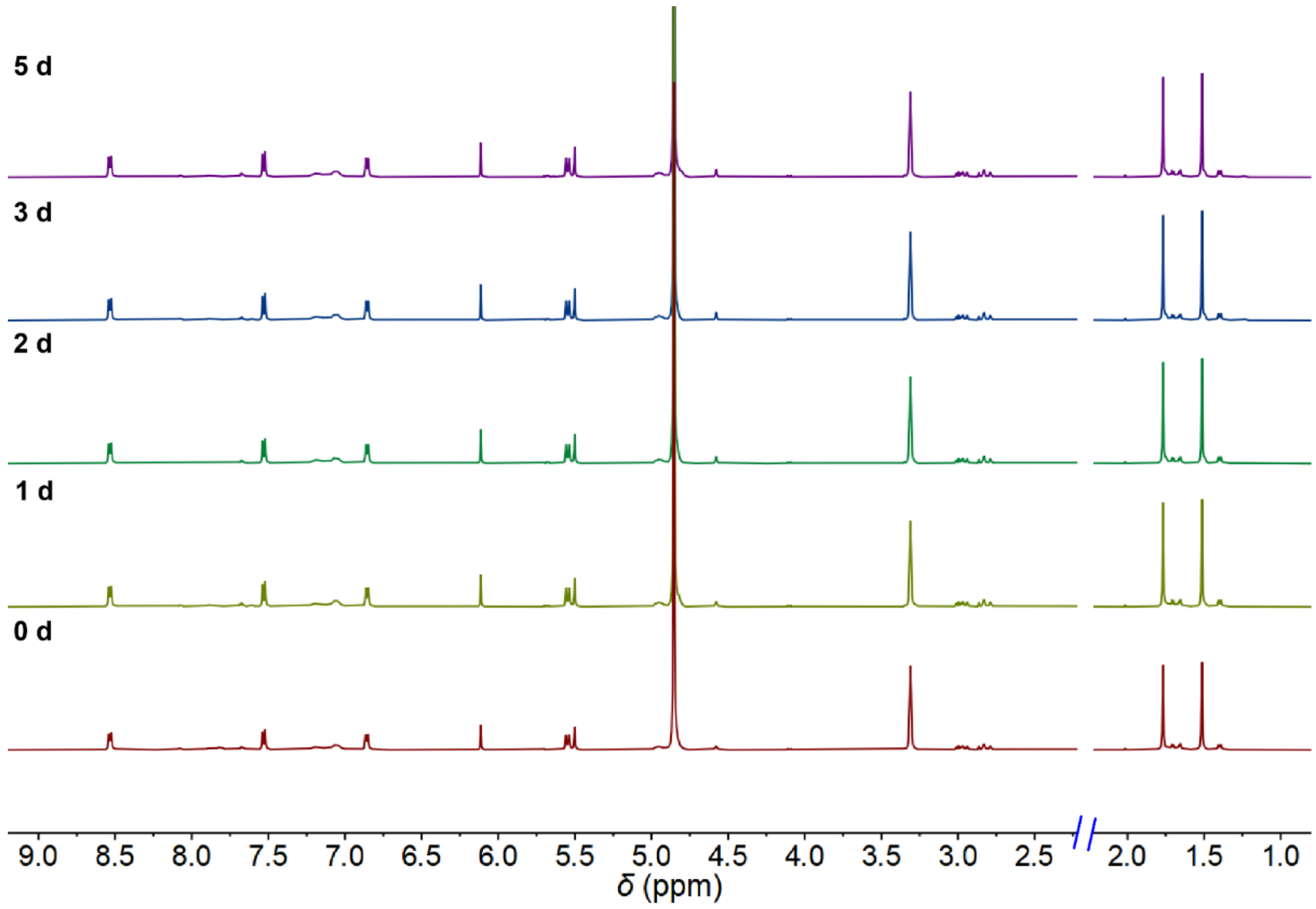

Fig. S44. ^{1}H NMR spectra of Λ-**Rh-1** recorded over 0–5 d in CD_3OD at room temperature (400 MHz, c = 6.0 mM). The spectra remain essentially unchanged over this period.

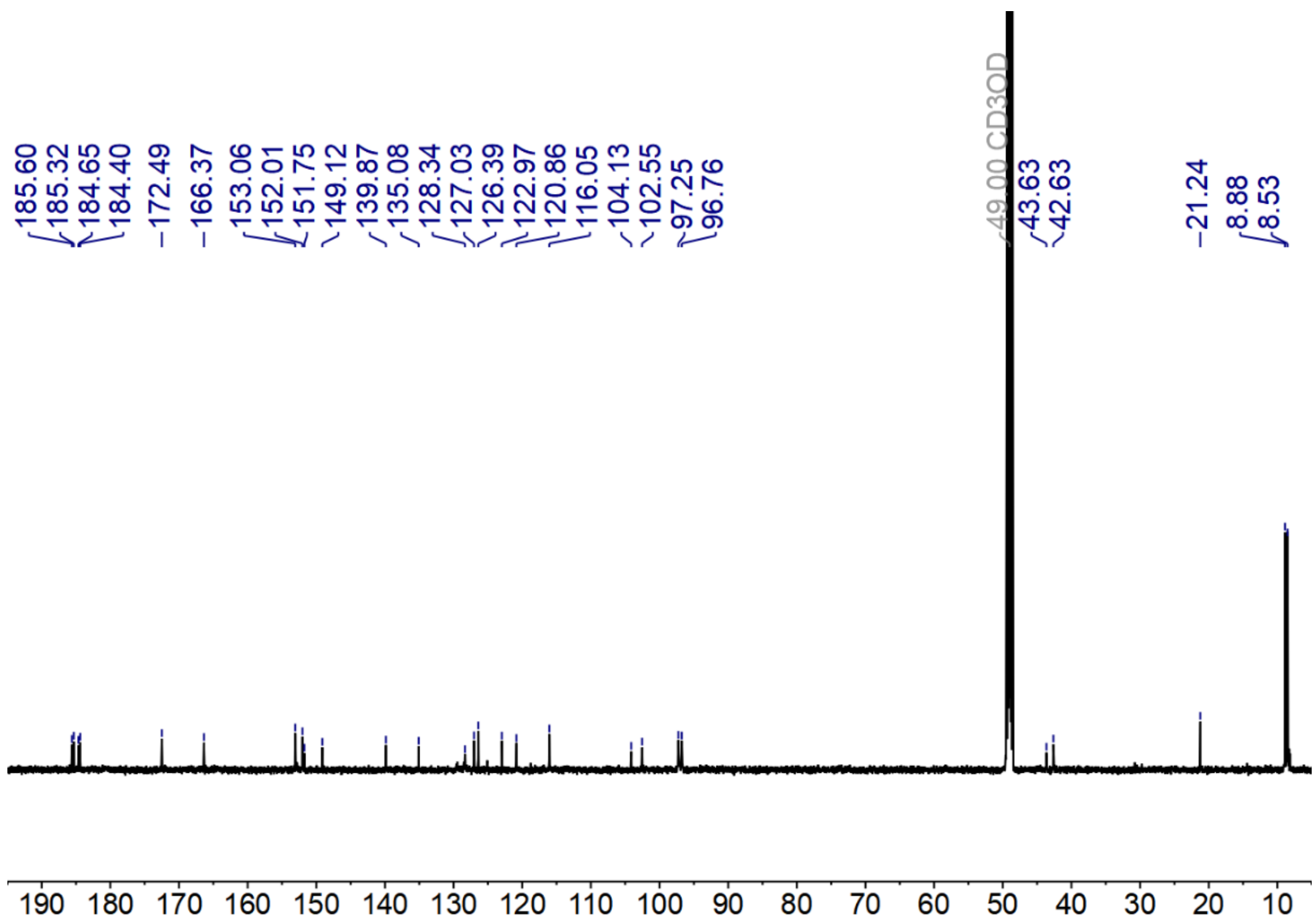

Fig. S45. ^{13}C{^{1}H} NMR spectrum of Λ-**Rh-1** in CD_3OD (151 MHz, c = 6.0 mM).

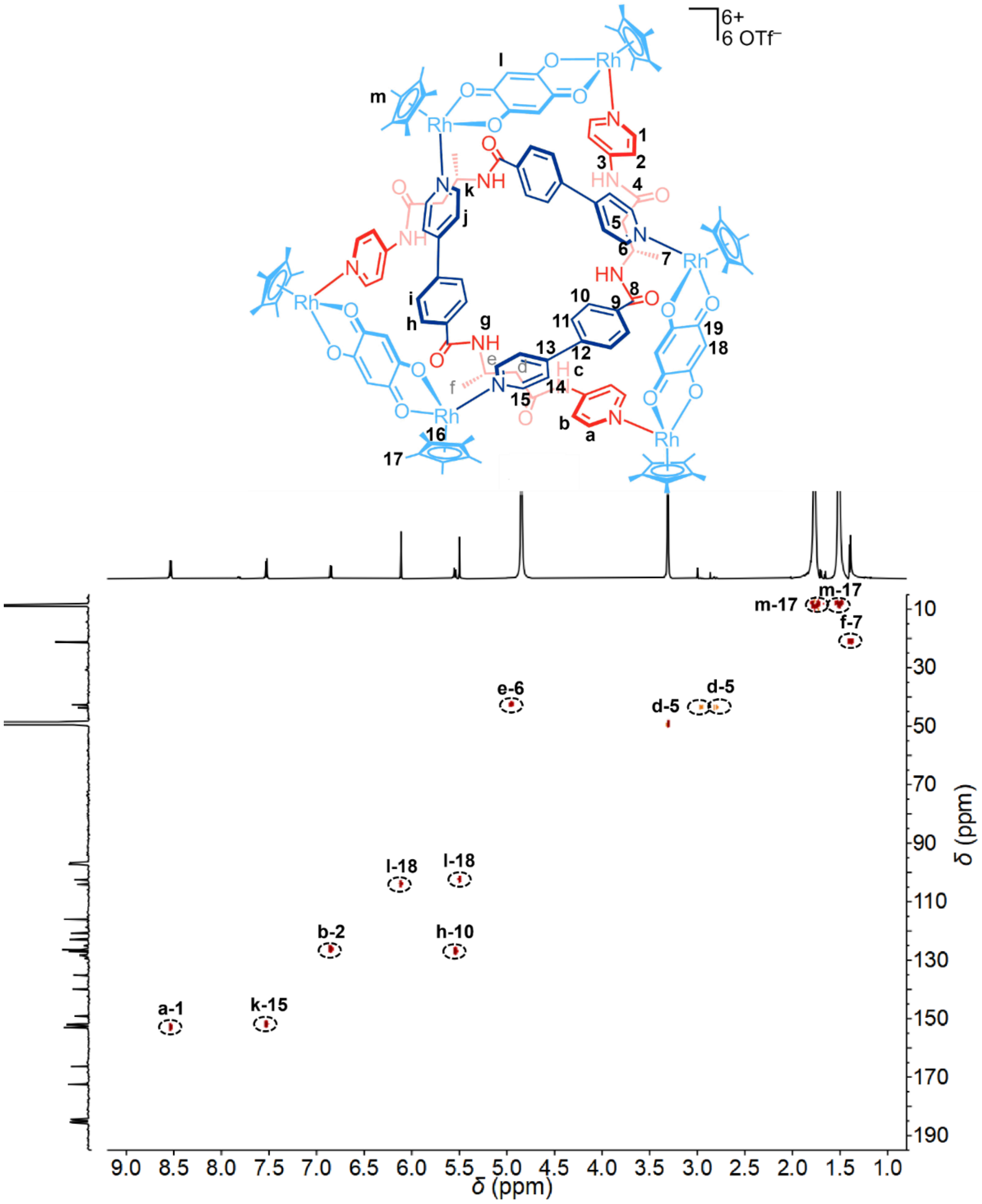

Fig. S46. ^1H–^{13}C HSQC spectrum of Λ-**Rh-1** in CD_3OD (600 MHz, c = 6.0 mM).

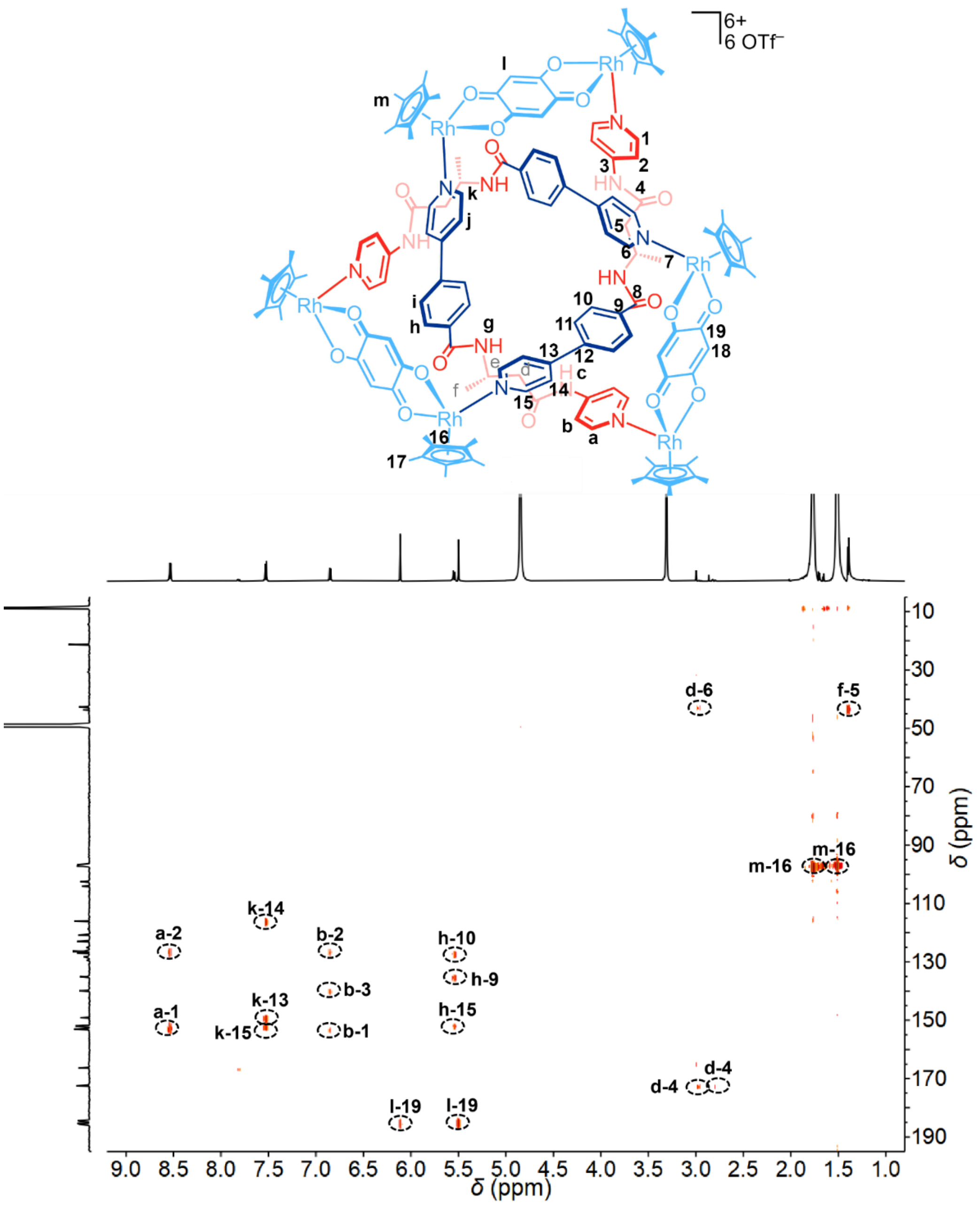

Fig. S47. ^{1}H–^{13}C HMBC spectrum of Λ-**Rh-1** in CD_3OD (600 MHz, c = 6.0 mM).

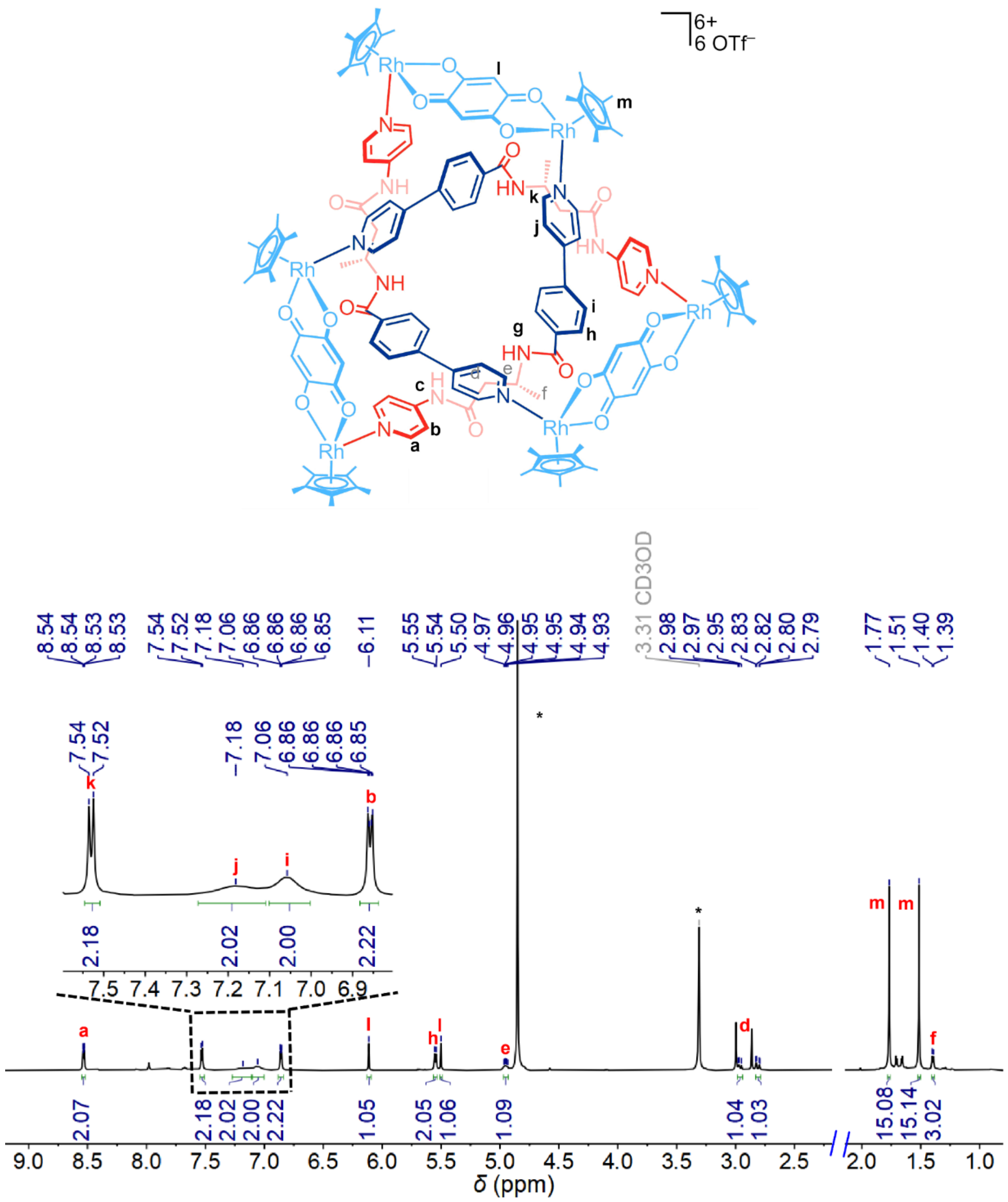

Fig. S48. ^{1}H NMR spectrum of Δ-**Rh-1** in CD_3OD (600 MHz, c = 6.0 mM, with respect to trefoil knots).

The hydrogen protons of NH on the ligand lose their NMR signal due to the exchange with D in CD_3OD.

* = solvent molecules.

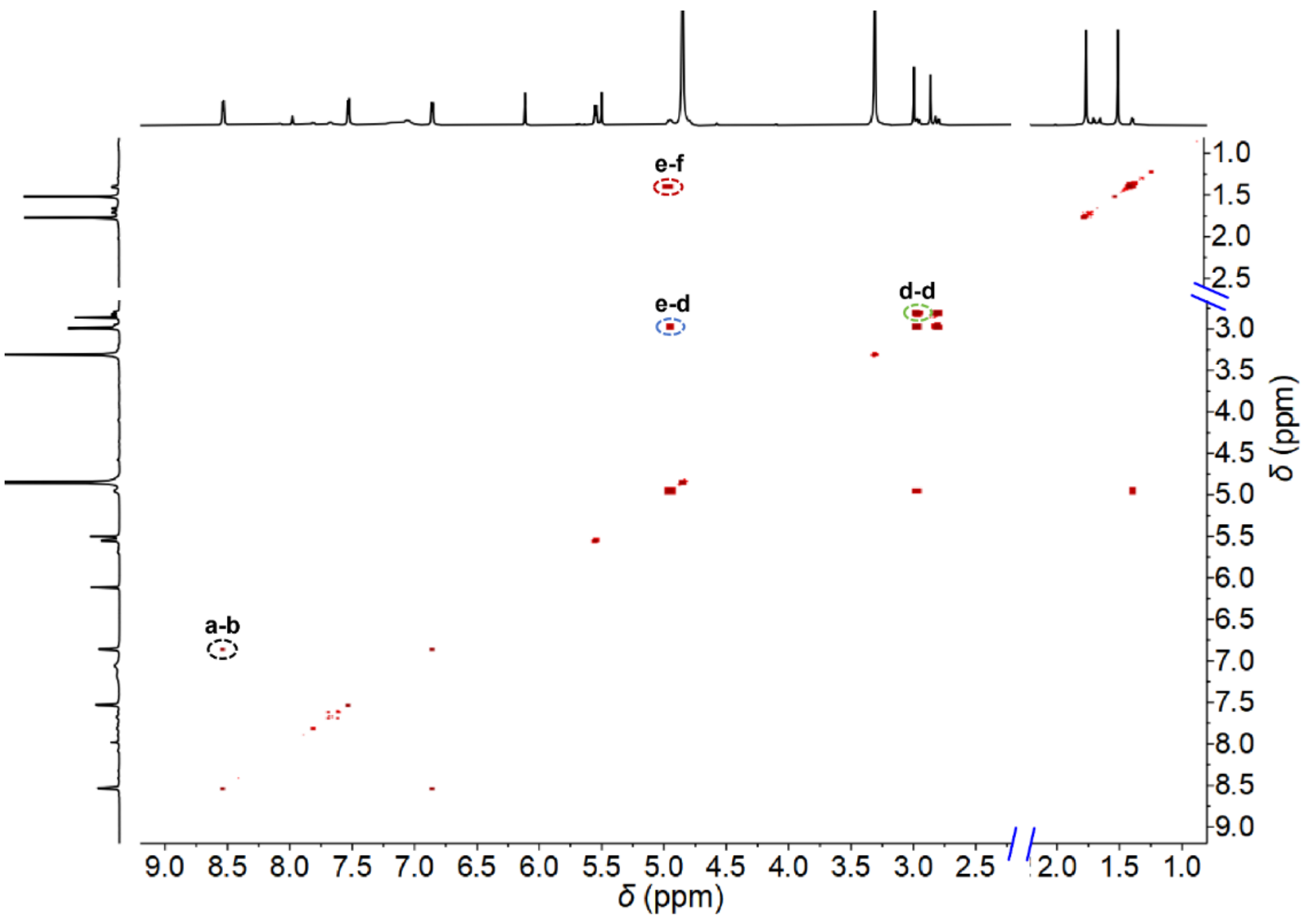

Fig. S49. ^{1}H-^{1}H COSY spectrum of Δ-**Rh-1** in CD_3OD (600 MHz, c = 6.0 mM, with respect to trefoil knots).

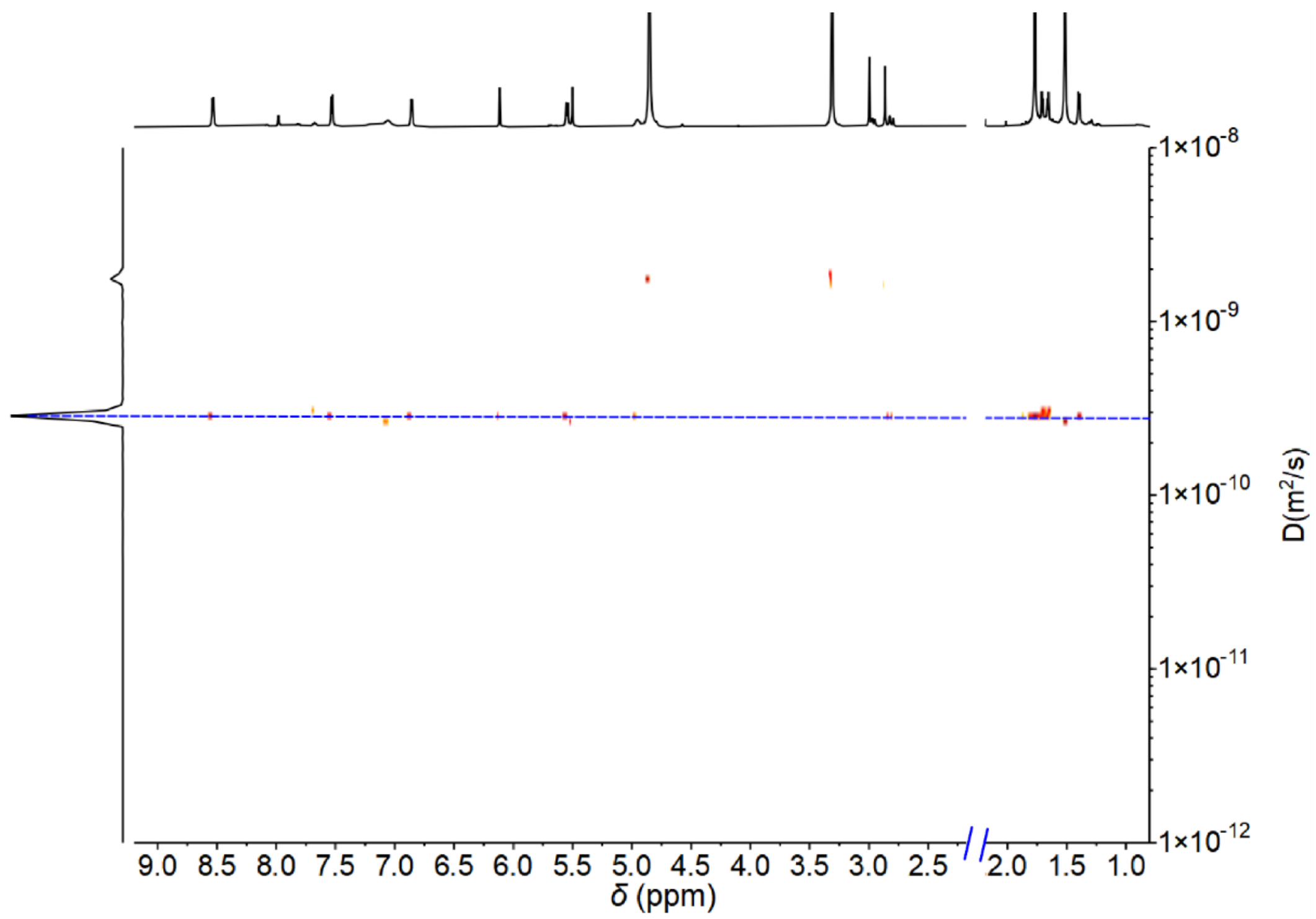

Fig. S50. ¹H DOSY spectrum of Δ-**Rh-1** in CD_3OD (600 MHz, c = 6.0 mM, where c = c_{Rh}/6). The assigned trefoil-knot resonances exhibit a diffusion coefficient of D = 2.83 × 10^{-10} m^2 s^{-1}.

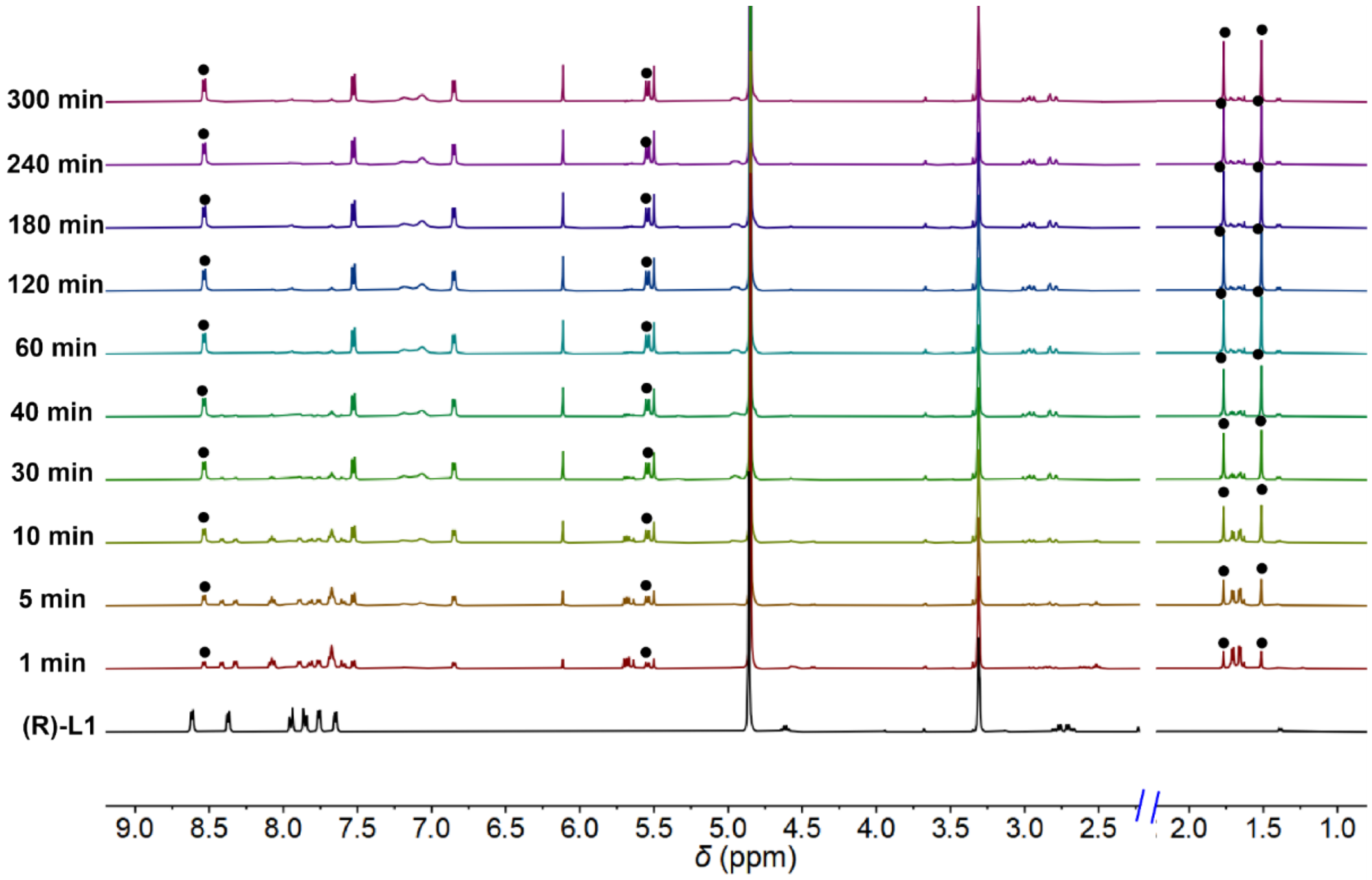

Fig. S51. Time-dependent ¹H NMR spectra monitoring self-assembly of (R)-**L1** with **B1**(OTf)$_2$ to form Δ-**Rh-1** (400 MHz, CD_3OD, room temperature; formal knot concentration = 6.0 mM). The time-dependent spectral changes are similar to those observed for Λ-**Rh-1**. Selected resonances corresponding to the trefoil-knot assemblies are highlighted with black circles.

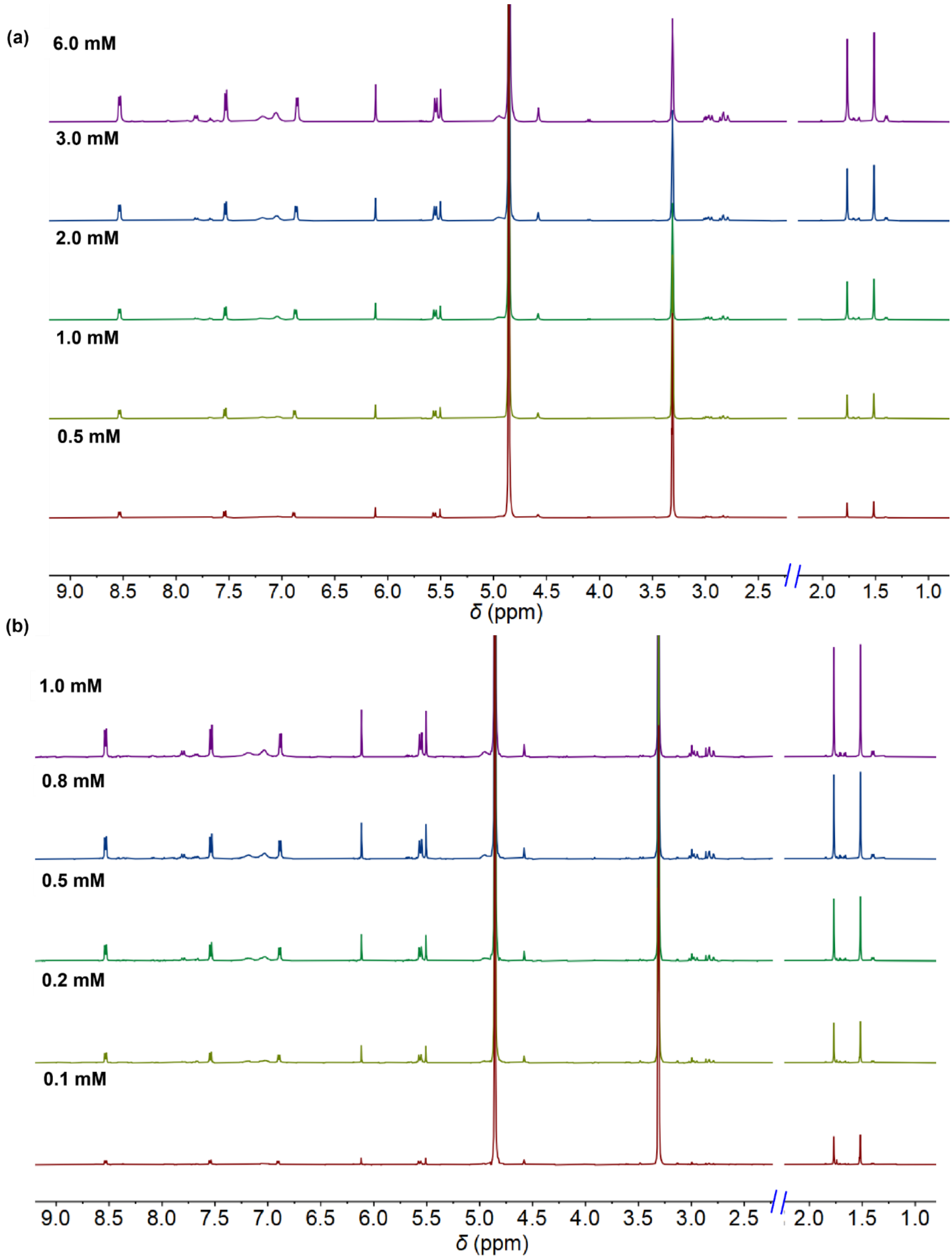

Fig. S52. Concentration-dependent ^{1}H NMR spectra of Δ-**Rh-1** (400 MHz, CD_3OD): (a) $c = 0.5–6.0$ mM and (b) $c = 0.1–1.0$ mM. The spectra show no substantial changes in the positions or relative intensities of the assigned assembly resonances over the concentration range examined.

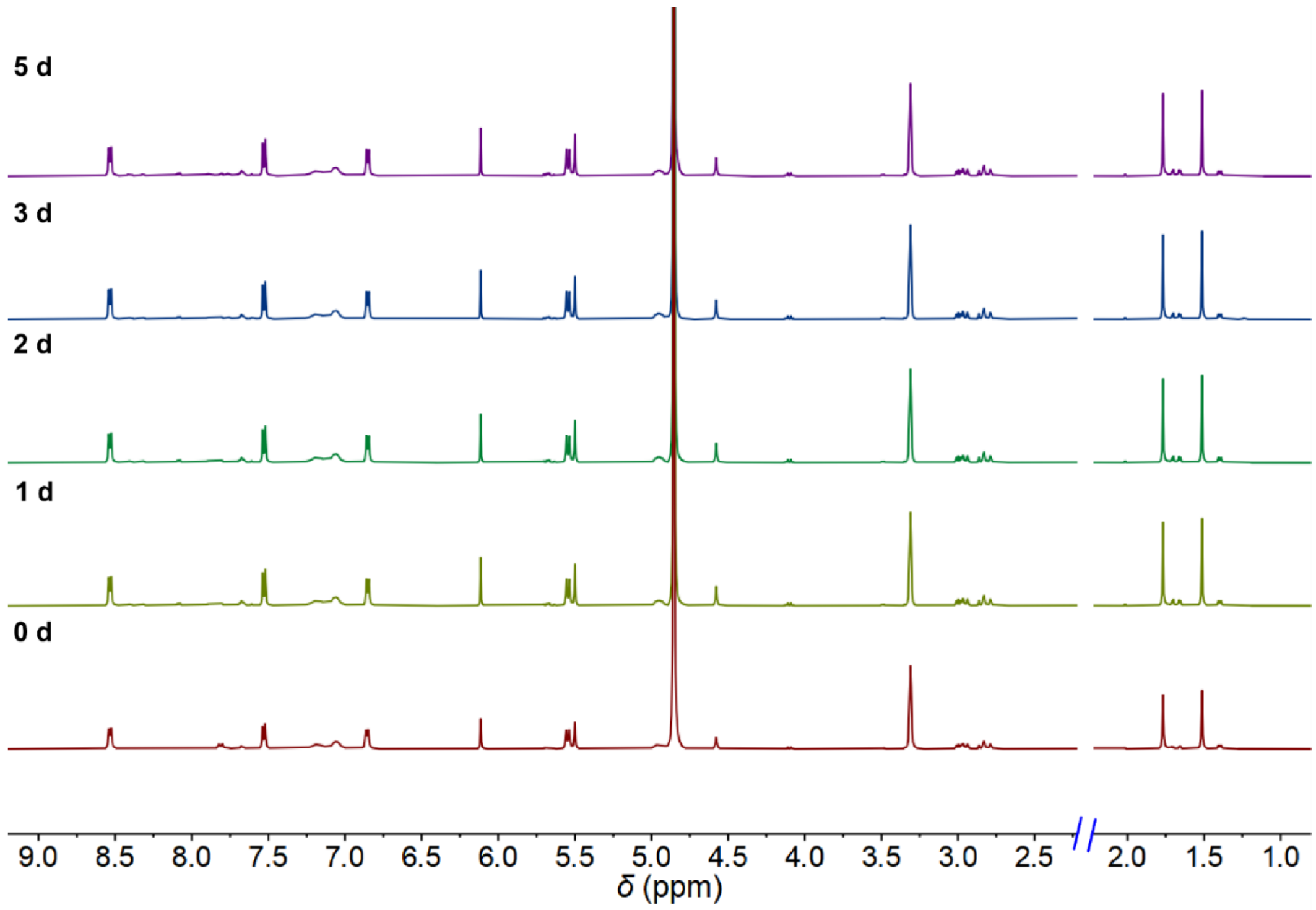

Fig. S53. ^{1}H NMR spectra of Δ-**Rh-1** recorded over 0–5 d in CD_3OD at room temperature (400 MHz, c = 6.0 mM). The spectra remain essentially unchanged over this period.

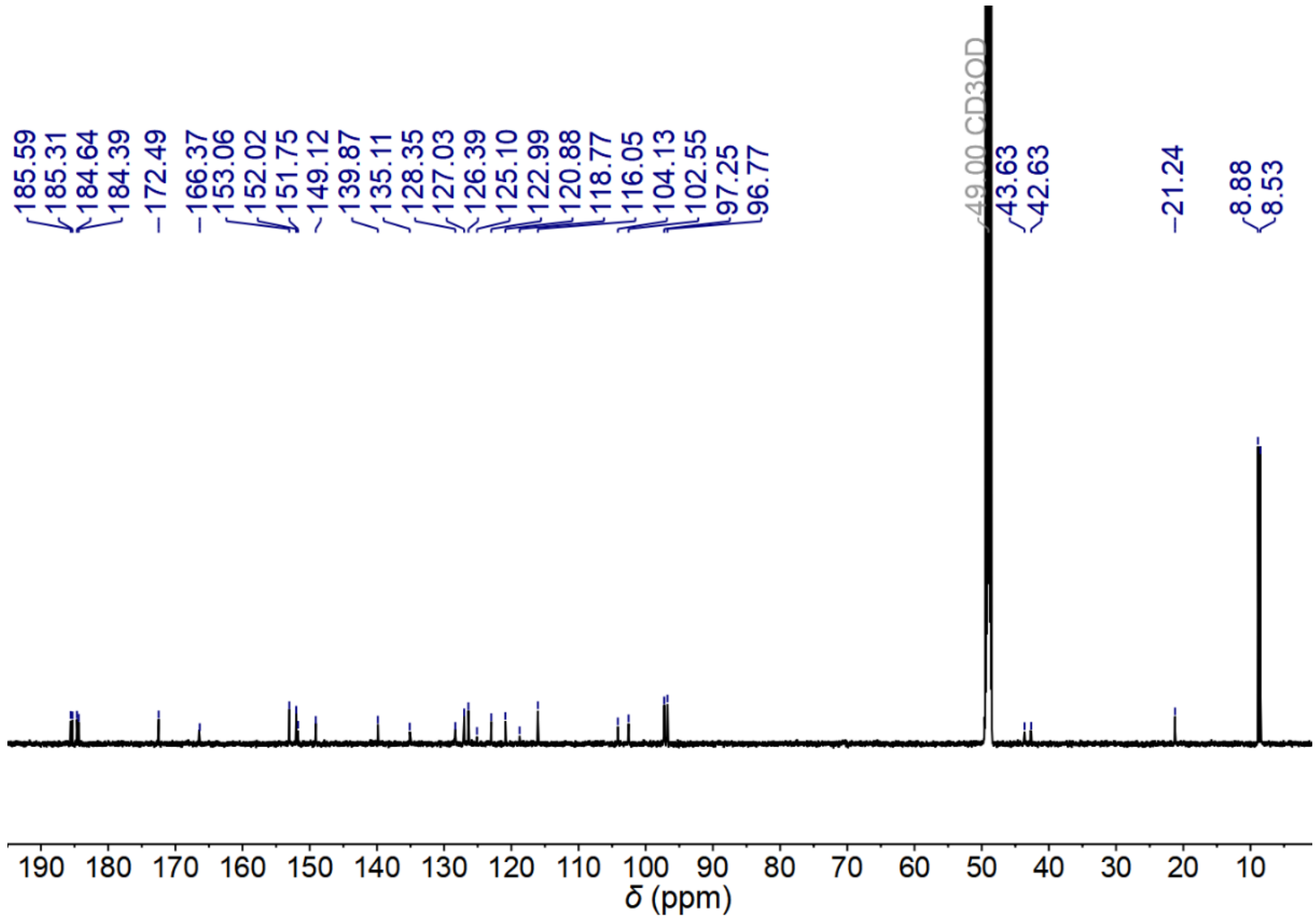

Fig. S54. $^{13}C\{^{1}H\}$ NMR spectrum of Δ-**Rh-1** in CD_3OD (151 MHz, c = 6.0 mM).

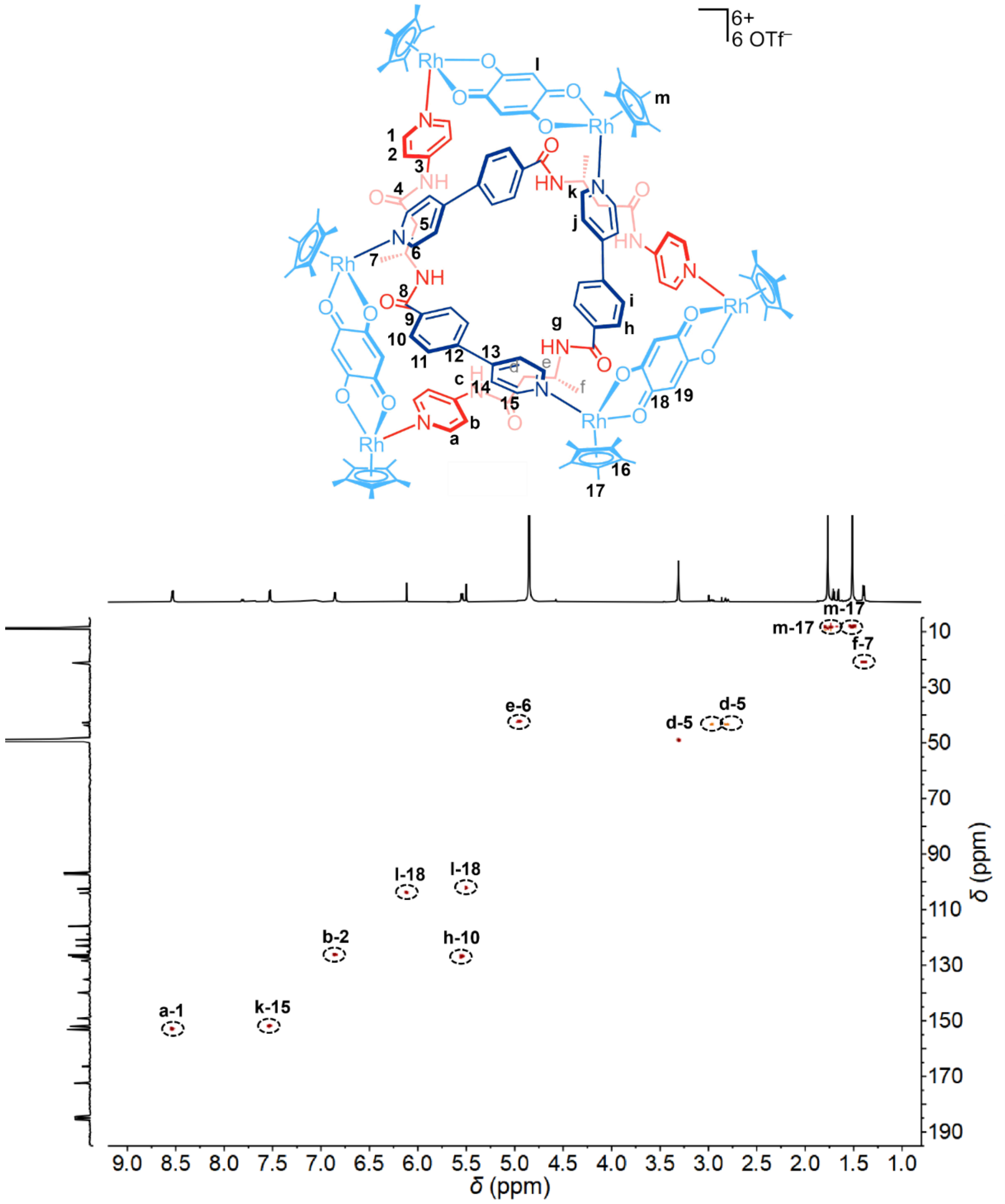

Fig. S55. ^1H–^{13}C HSQC spectrum of Δ-**Rh-1** in CD_3OD (600 MHz, c = 6.0 mM).

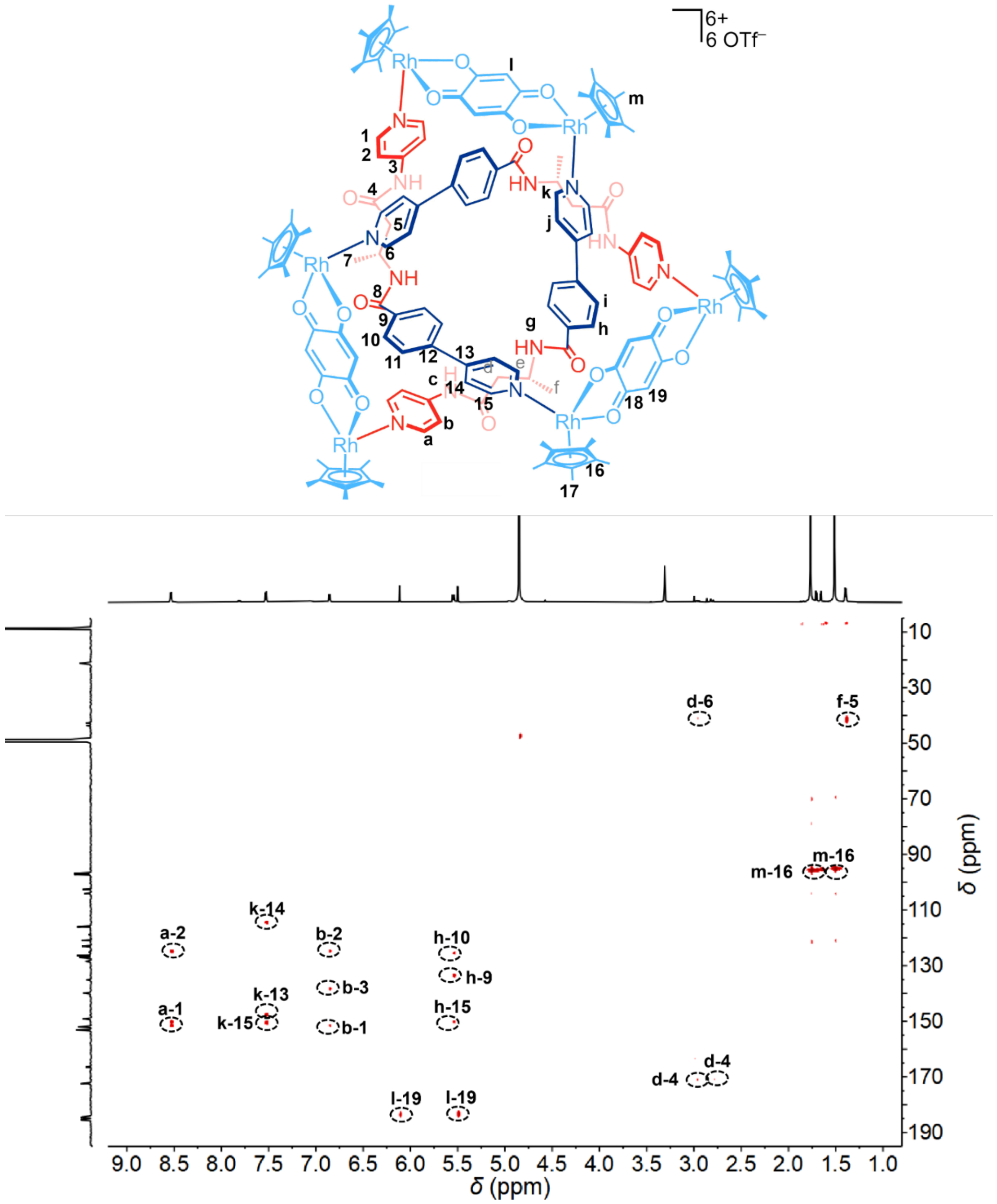

Fig. S56. ^1H–^{13}C HMBC spectrum of Δ-**Rh-1** in CD_3OD (600 MHz, c = 6.0 mM).

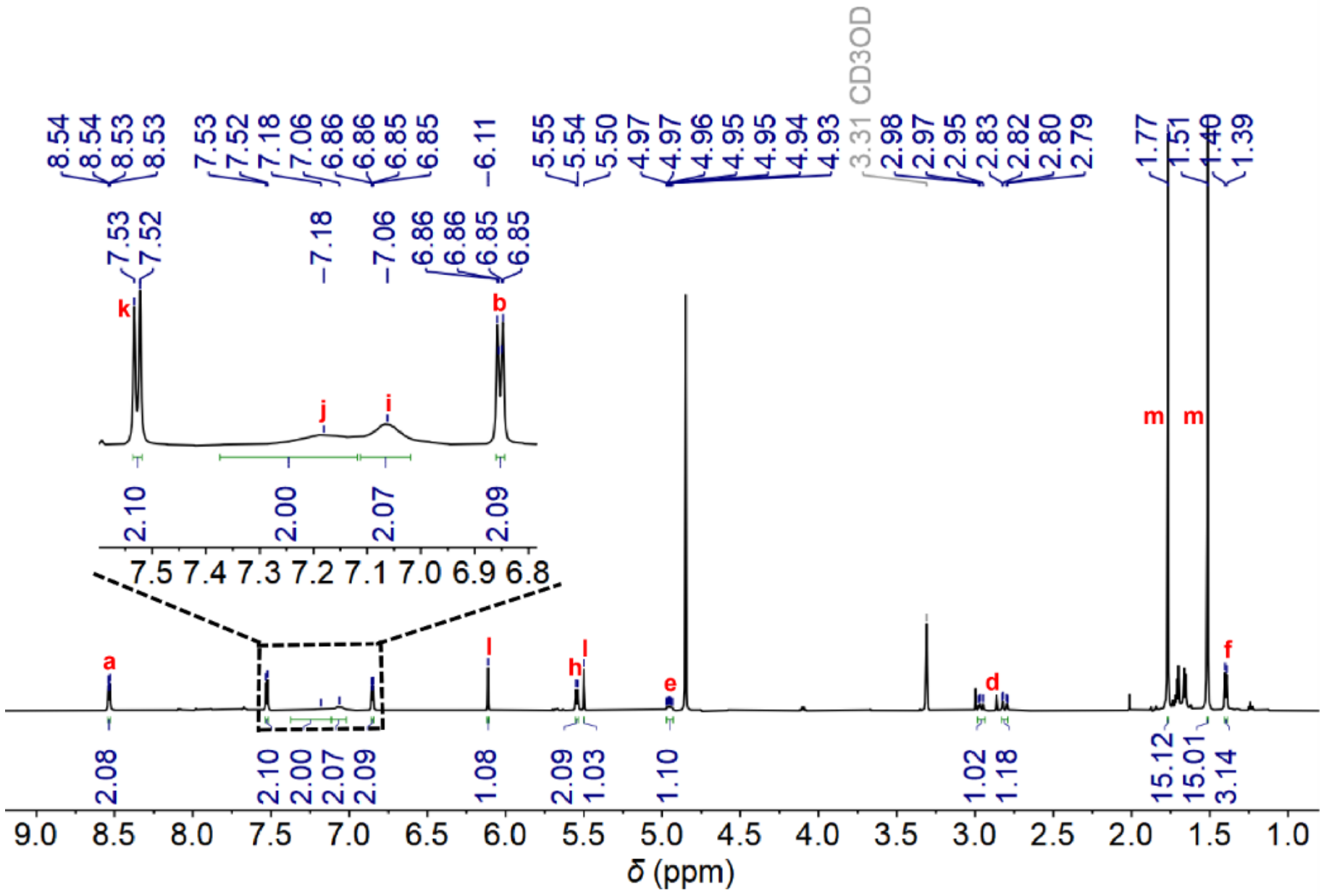

Fig. S57. ^{1}H NMR spectrum of the assembly sample obtained from racemic (*S*)-**L1**/(*R*)-**L1** and **B1**(OTf)$_2$ (600 MHz, CD_3OD; total knot concentration, *c* = 6.0 mM). The resonance pattern matches that of the corresponding enantiopure trefoil knots, consistent with a racemic mixture of Λ-**Rh-1** and Δ-**Rh-1**. The hydrogen protons of NH on the ligand lose their NMR signal due to the exchange with D in CD_3OD.

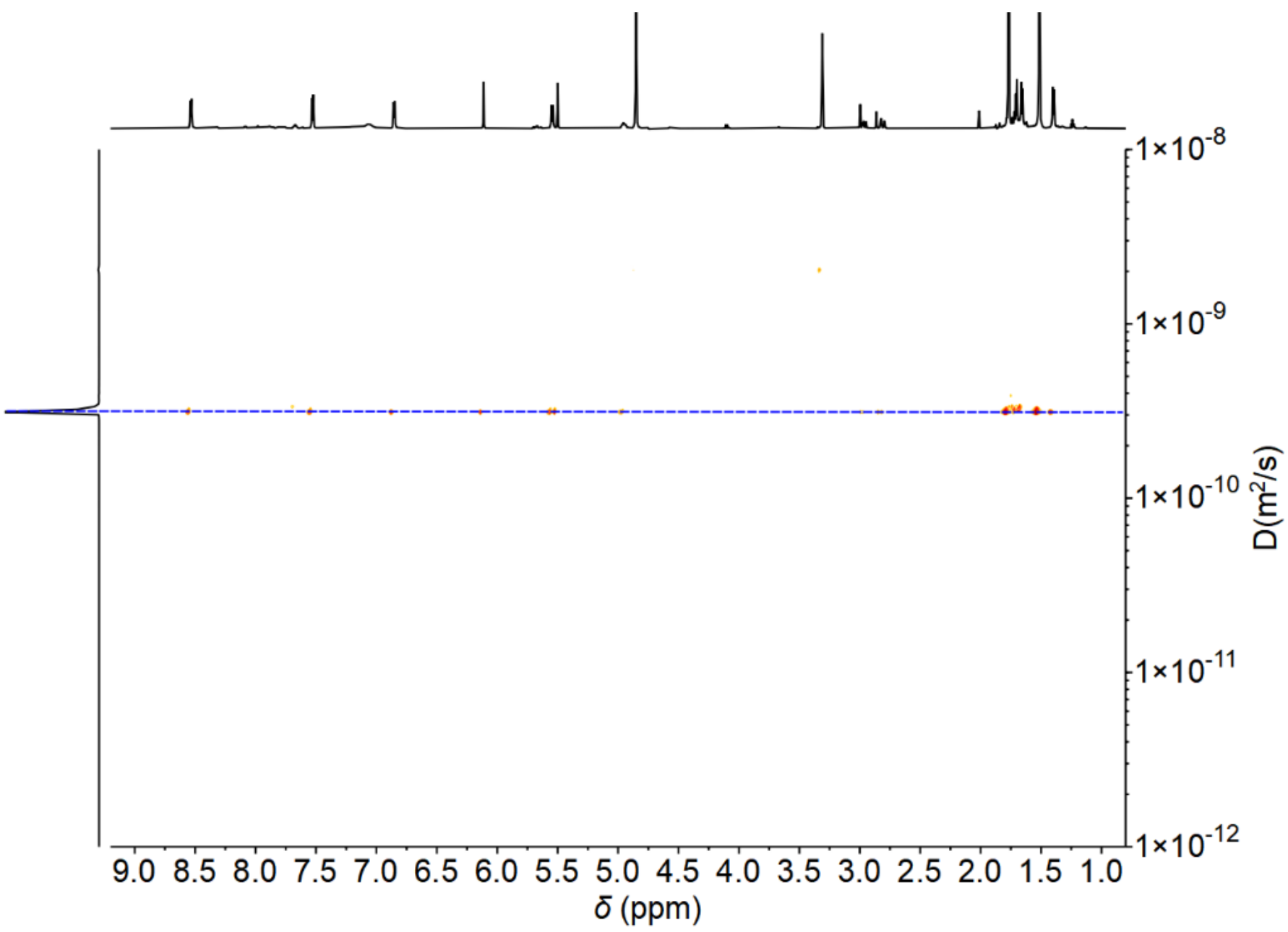

Fig. S58. ¹H DOSY spectrum of the assembly sample obtained from racemic (*S*)-**L1**/(*R*)-**L1** and **B1**(OTf)$_2$ (600 MHz, CD_3OD; total knot concentration, c = 6.0 mM). The assigned assembly resonances exhibit a principal diffusion coefficient of 3.15×10^{-10} m² s⁻¹.

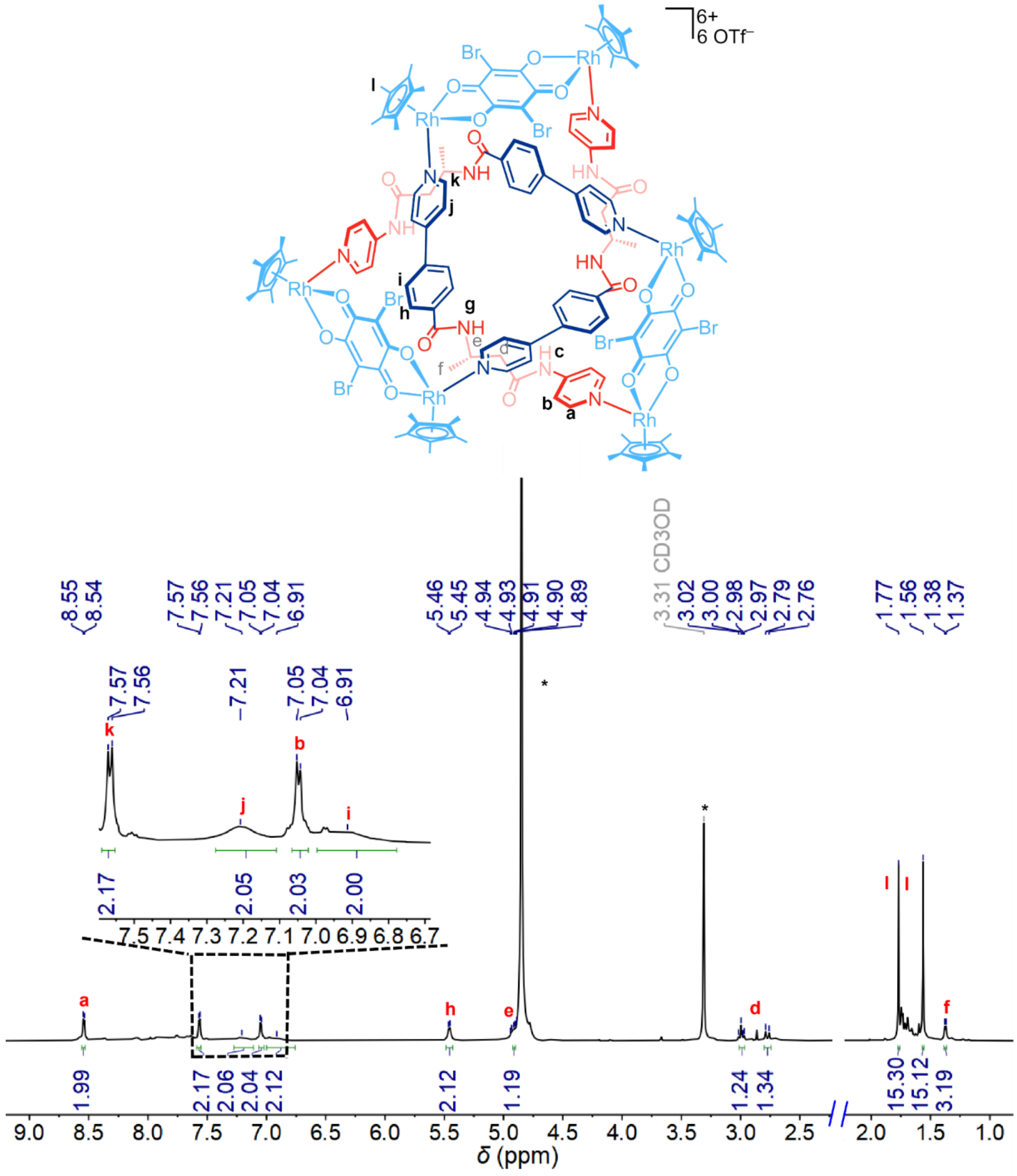

Fig. S59. ¹H NMR spectrum of Λ-**Rh-1Br** in CD_3OD (600 MHz, c = 6.0 mM, with respect to trefoil knots). The hydrogen protons of NH on the ligand lose their NMR signal due to the exchange with D in CD_3OD. * = solvent molecules.

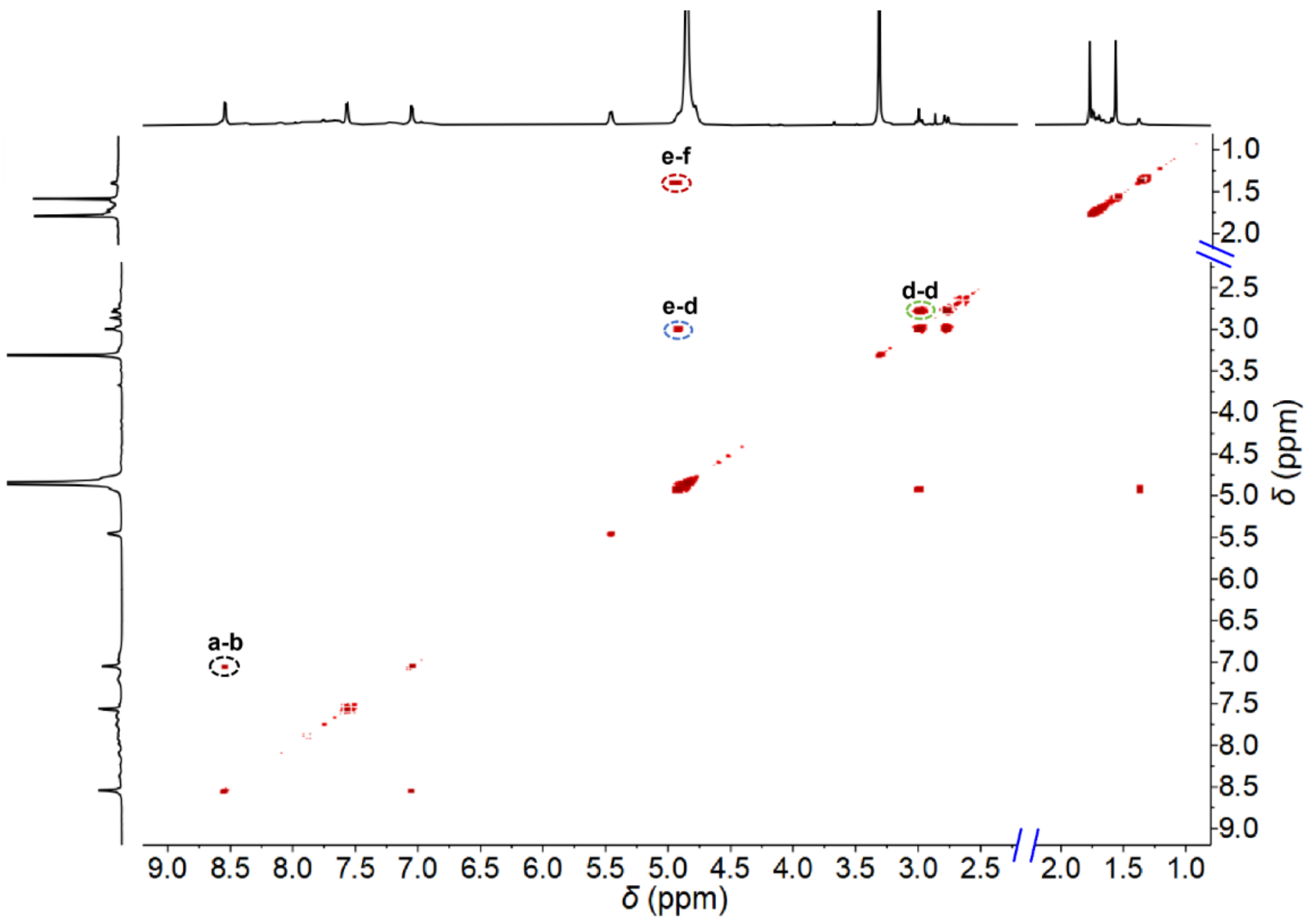

Fig. S60. ^{1}H-^{1}H COSY spectrum of Λ-**Rh-1Br** in CD_3OD (600 MHz, c = 6.0 mM, with respect to trefoil knots).

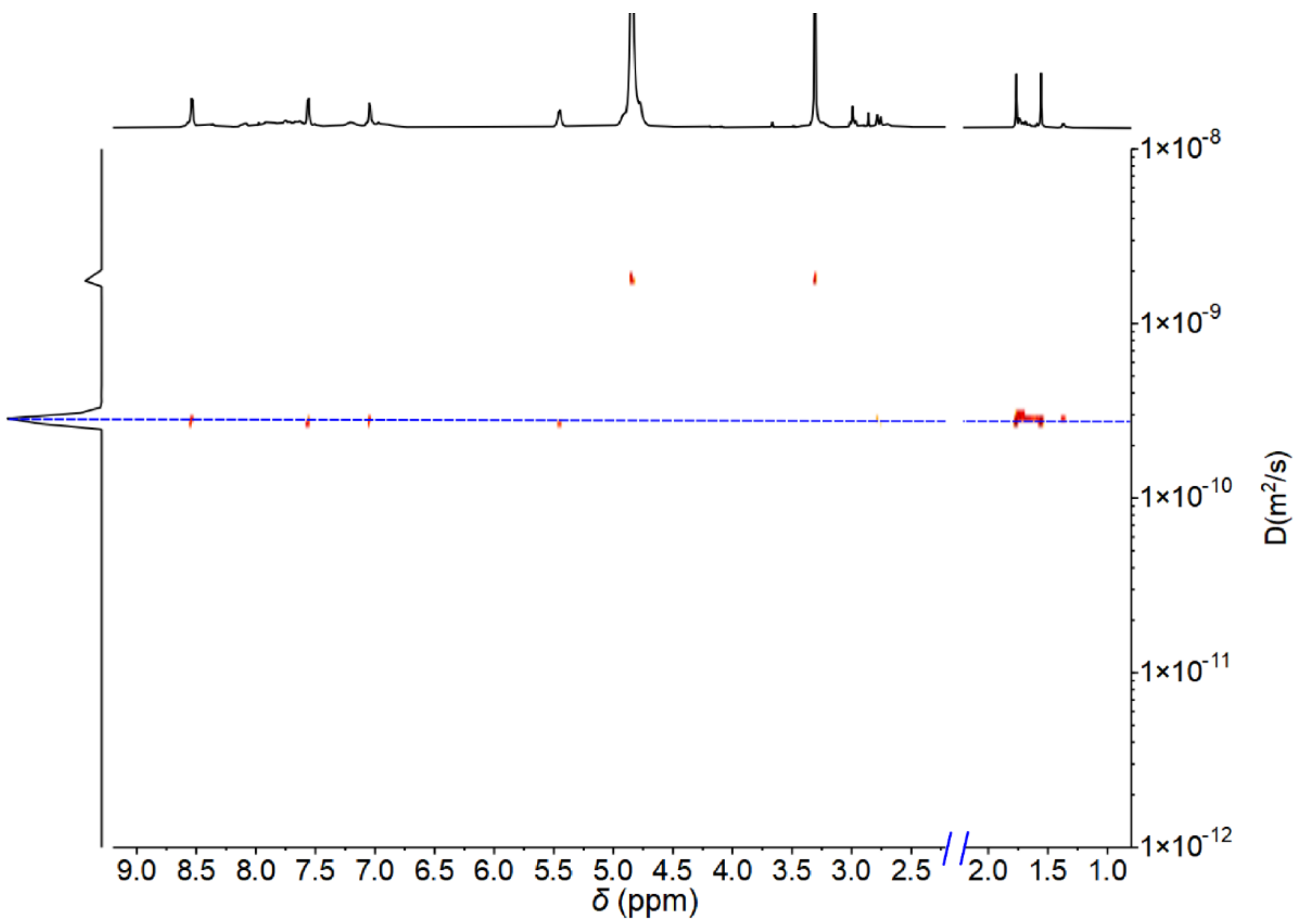

Fig. S61. ^{1}H DOSY spectrum of Λ-**Rh-1Br** in CD_3OD (600 MHz, c = 6.0 mM, where $c = c_{Rh}/6$). The assigned trefoil-knot resonances exhibit a diffusion coefficient of $D = 2.79 \times 10^{-10}$ m^2 s^{-1}.

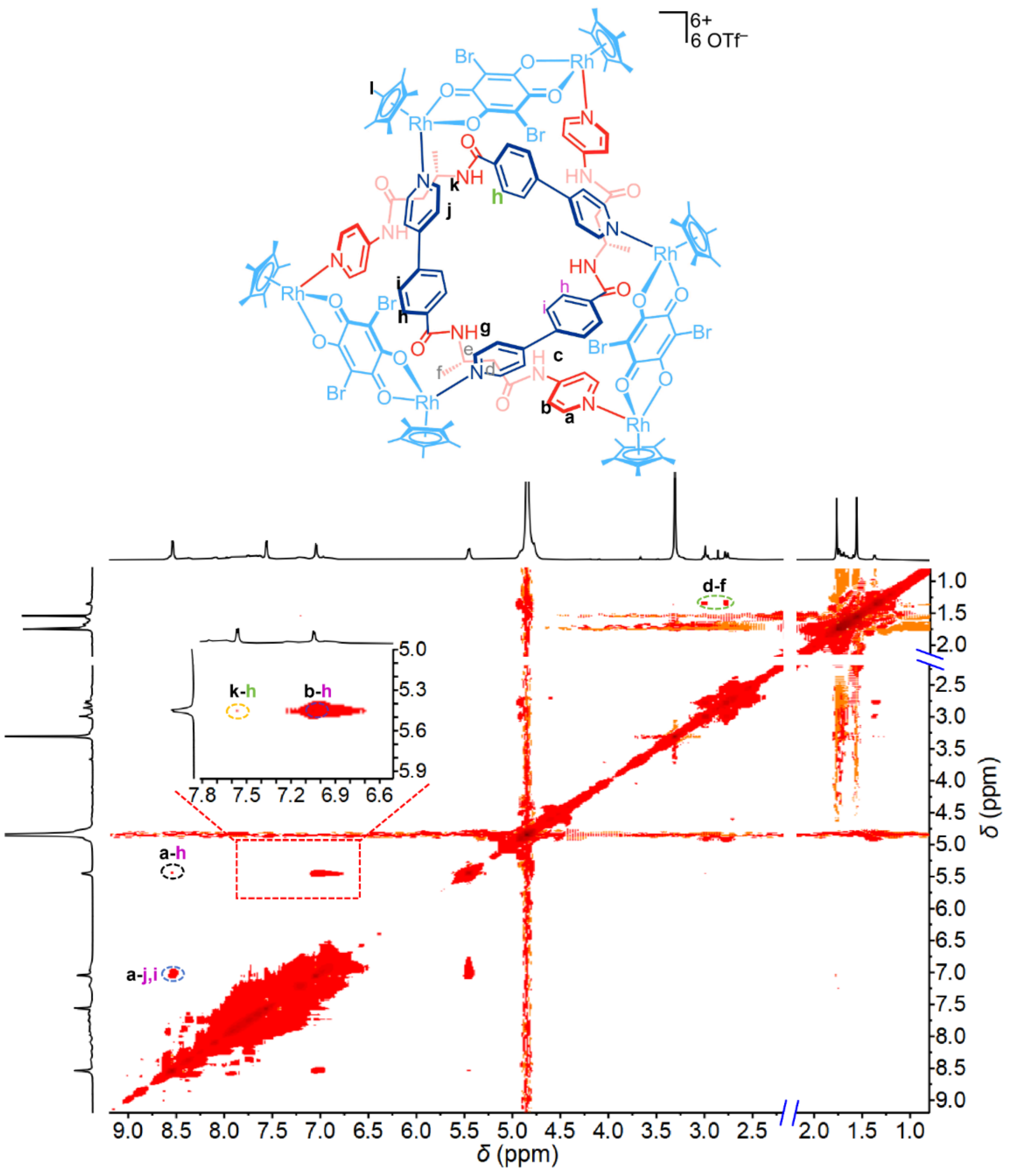

Fig. S62. ¹H-¹H NOESY NMR (600 MHz, CD_3OD, *c* = 6.0 mM) for Λ-**Rh-1Br**.

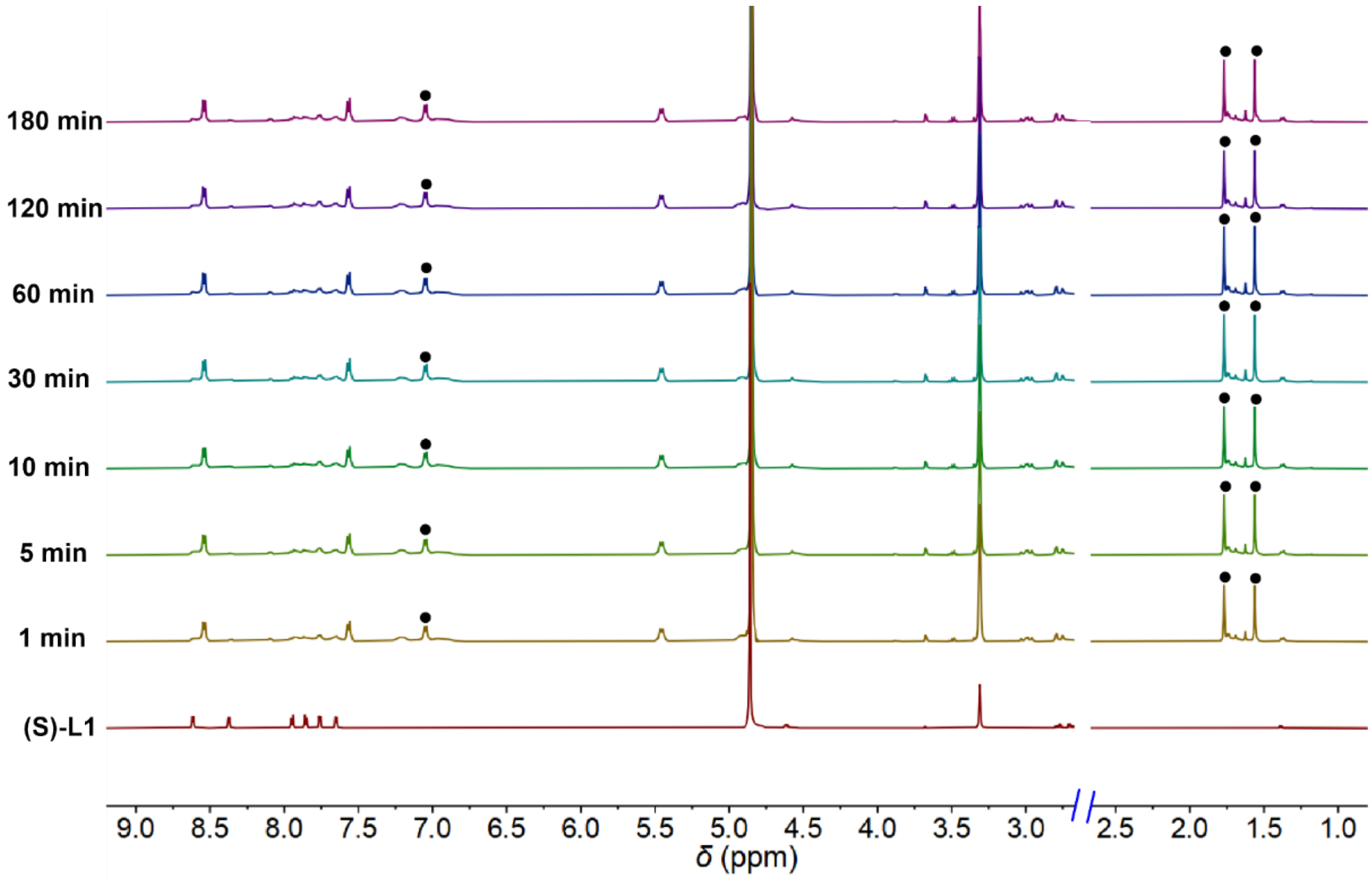

Fig. S63. Time-dependent ¹H NMR spectra monitoring self-assembly of (*S*)-**L1** with **B2**$(OTf)_2$ to form Λ-**Rh-1Br** (400 MHz, CD_3OD, room temperature; formal knot concentration = 6.0 mM). Formation is largely complete within 5 min. Selected resonances corresponding to the trefoil-knot assemblies are highlighted with black circles.

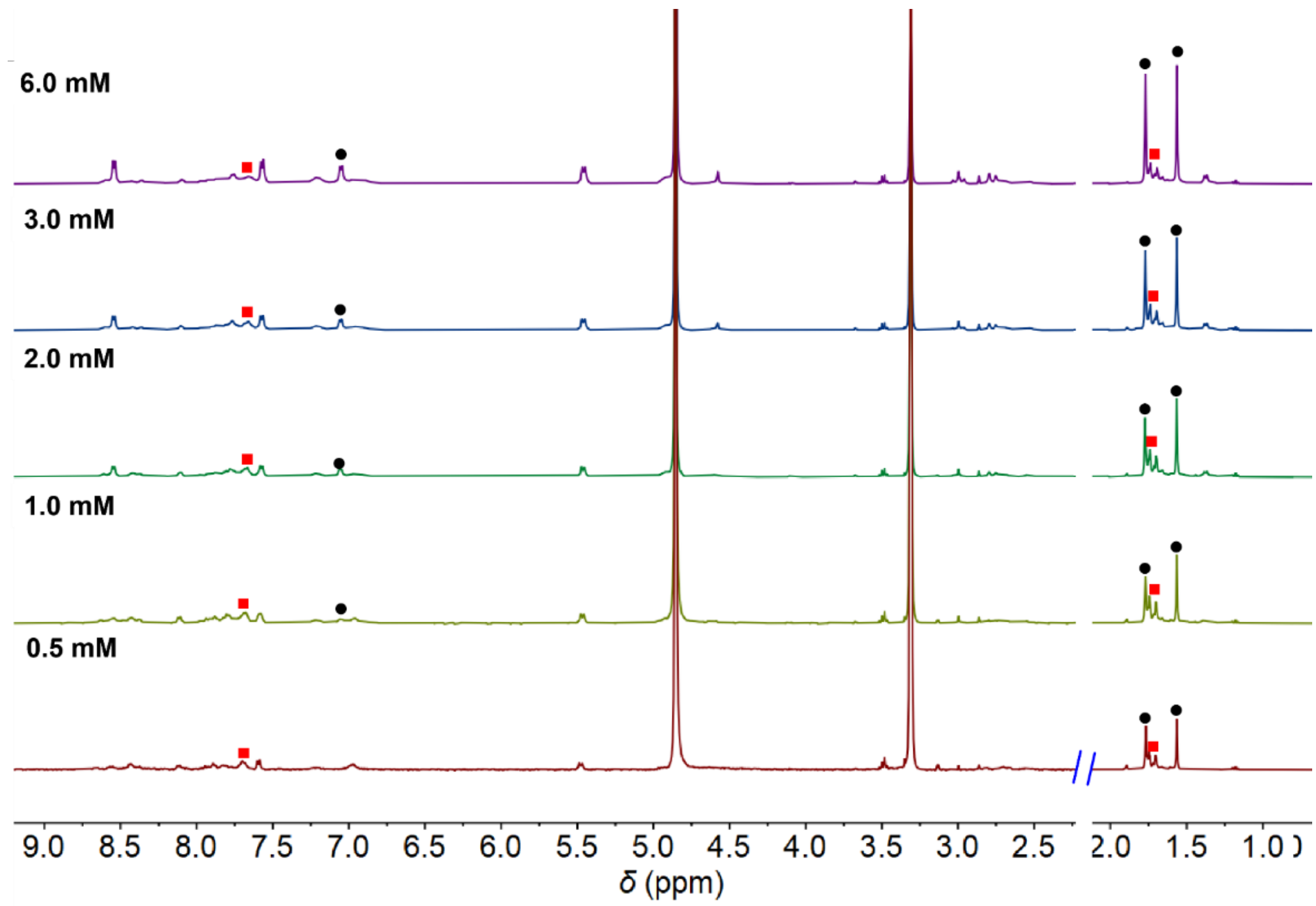

Fig. S64. Concentration-dependent ^{1}H NMR spectra of Λ-**Rh-1Br** over c = 0.5–6.0 mM (600 MHz, CD_3OD). Black circles mark selected resonances assigned to the trefoil knot, and red squares mark selected additional resonances tentatively assigned to tetranuclear metallomacrocyclic species. Upon dilution, the trefoil-knot resonances become less prominent relative to the additional resonances.

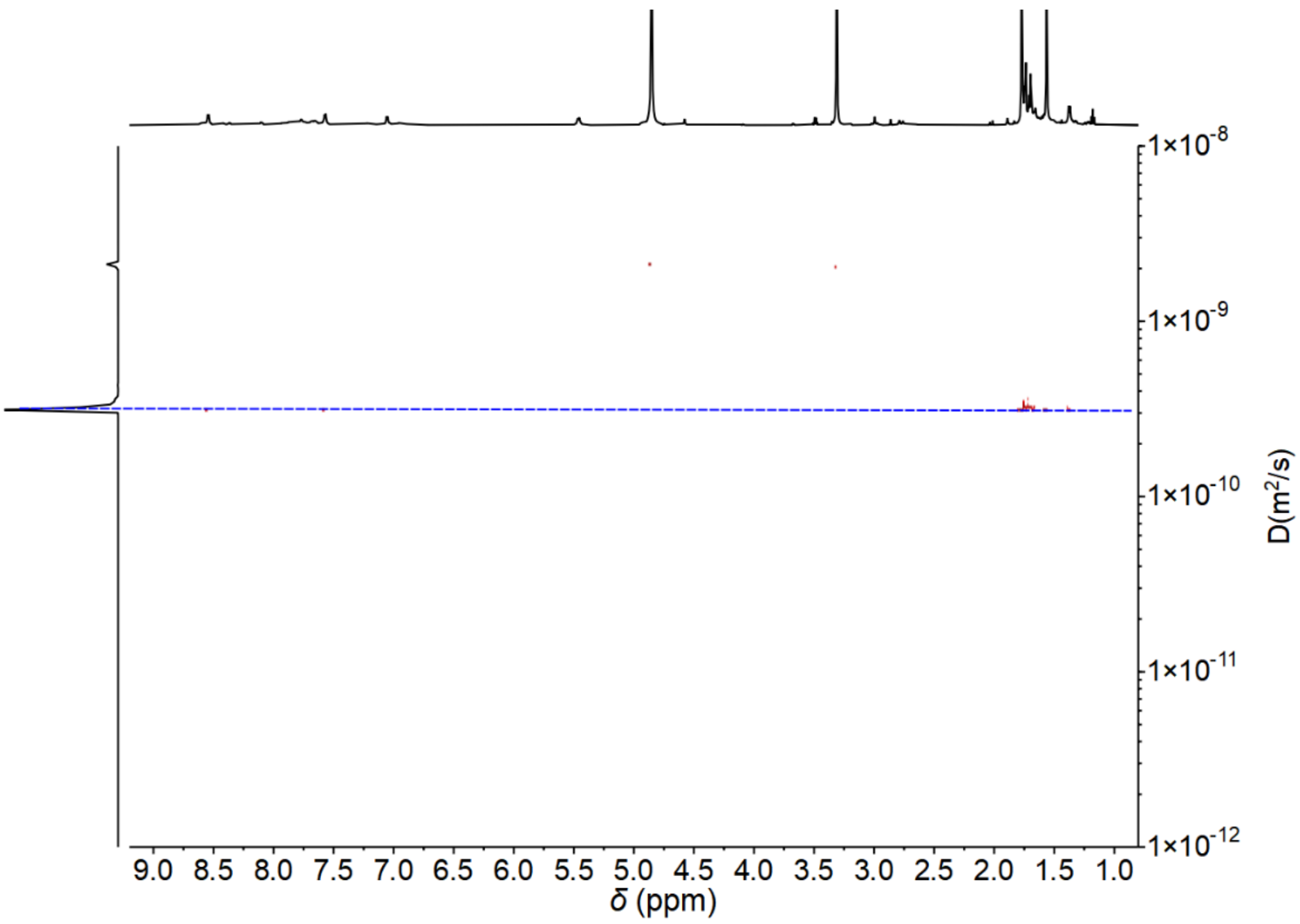

Fig. S65. ^{1}H DOSY spectrum of Λ-**Rh-1Br** in CD_3OD (600 MHz, c = 3.0 mM, where $c = c_{Rh}/6$). The assigned trefoil-knot resonances exhibit a diffusion coefficient of $D = 3.12 \times 10^{-10}$ m^{2} s^{-1}.

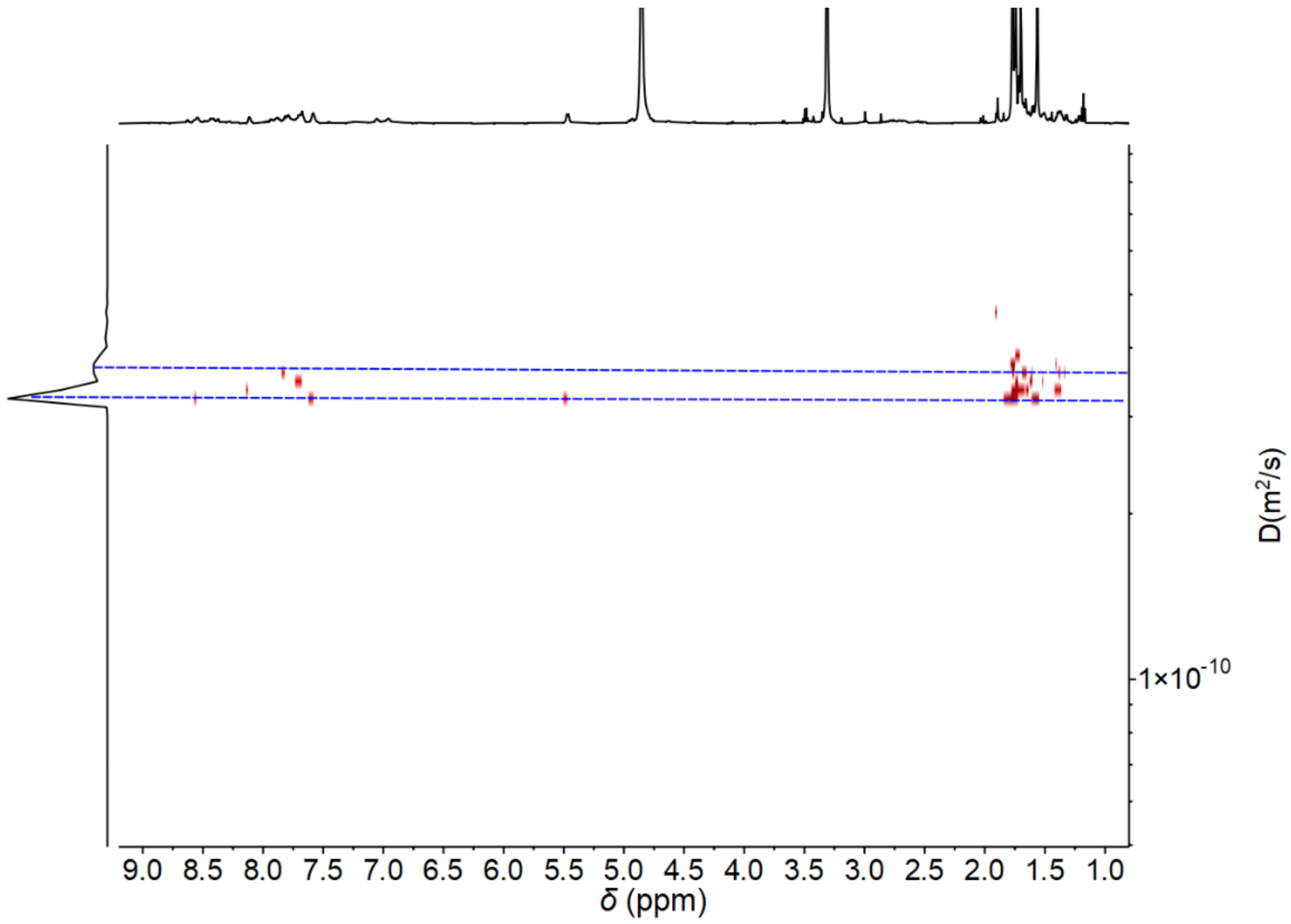

Fig. S66. ^{1}H DOSY spectrum of Λ-**Rh-1Br** (600 MHz, CD_3OD, c = 1.0 mM). The resonances assigned to the trefoil knot exhibit D = 3.24 × 10^{-10} m^2 s^{-1}. A minor set of resonances exhibits a larger diffusion coefficient of 3.62 × 10^{-10} m^2 s^{-1}, consistent with a smaller species, possibly a tetranuclear metallomacrocycle.

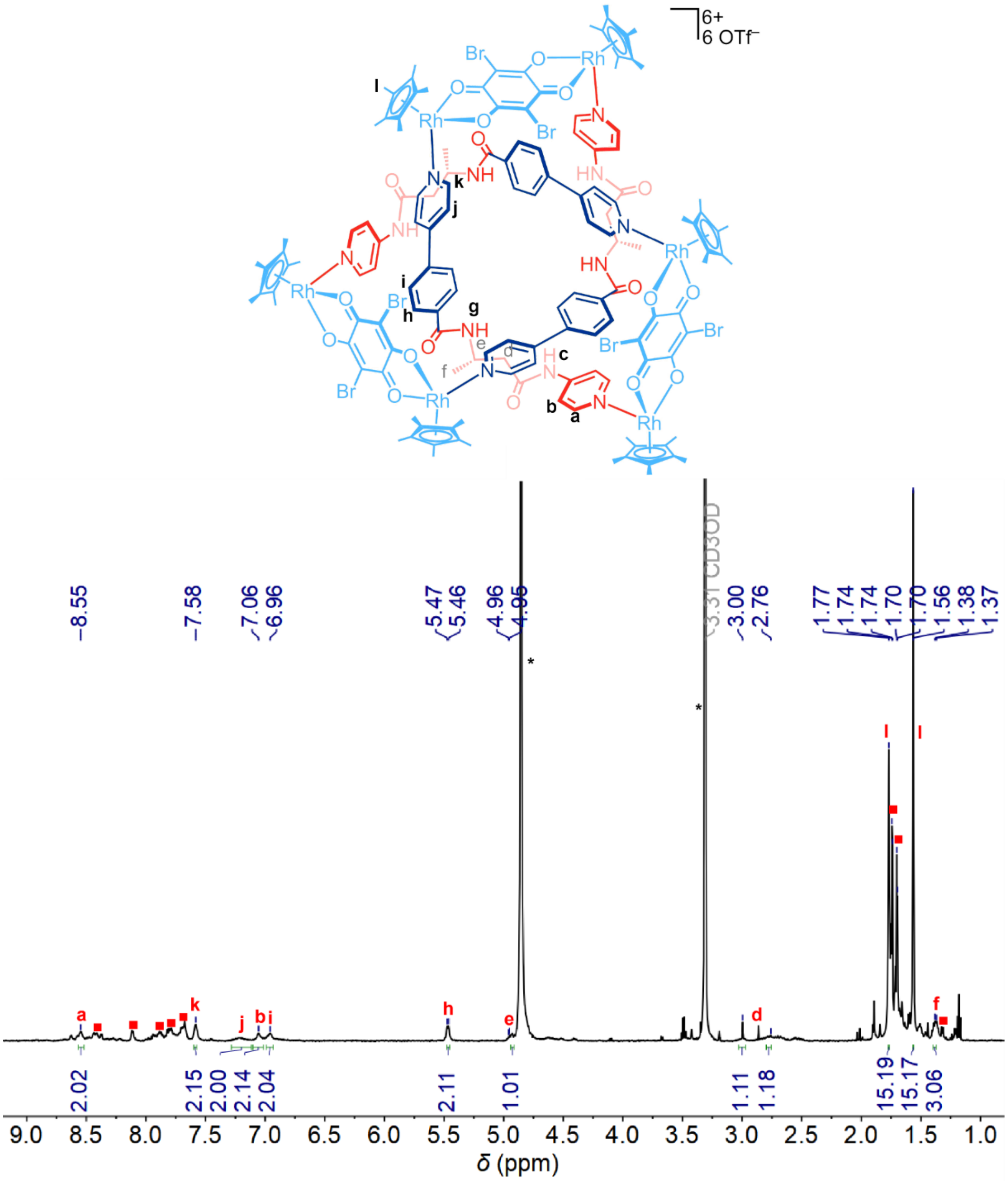

Fig. S67. ^{1}H NMR spectrum of Λ-**Rh-1Br** in CD_3OD (600 MHz, *c* = 1.0 mM, with respect to trefoil knots). The hydrogen protons of NH on the ligand lose their NMR signal due to the exchange with D in CD_3OD. Red squares mark the resonances of a minor solution component. * = solvent molecules.

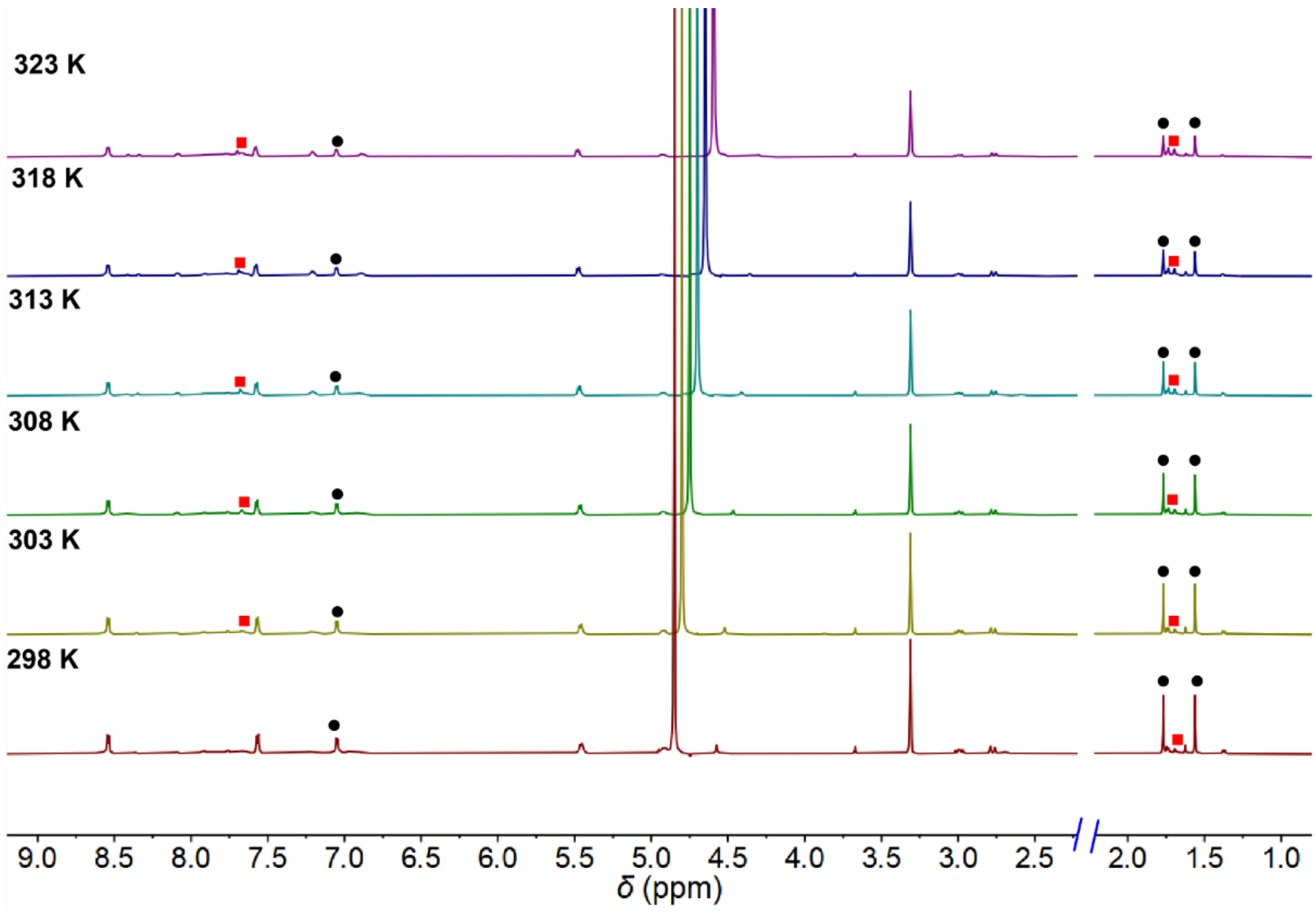

Fig. S68. Variable-temperature ^{1}H NMR spectra of Λ-**Rh-1Br** recorded from 298 to 323 K (600 MHz, CD_3OD, c = 6.0 mM). Black circles mark selected resonances assigned to the trefoil knot, and red squares mark selected additional resonances tentatively assigned to tetranuclear metallomacrocyclic species. Upon heating, the trefoil-knot resonances become less prominent relative to the additional resonances.

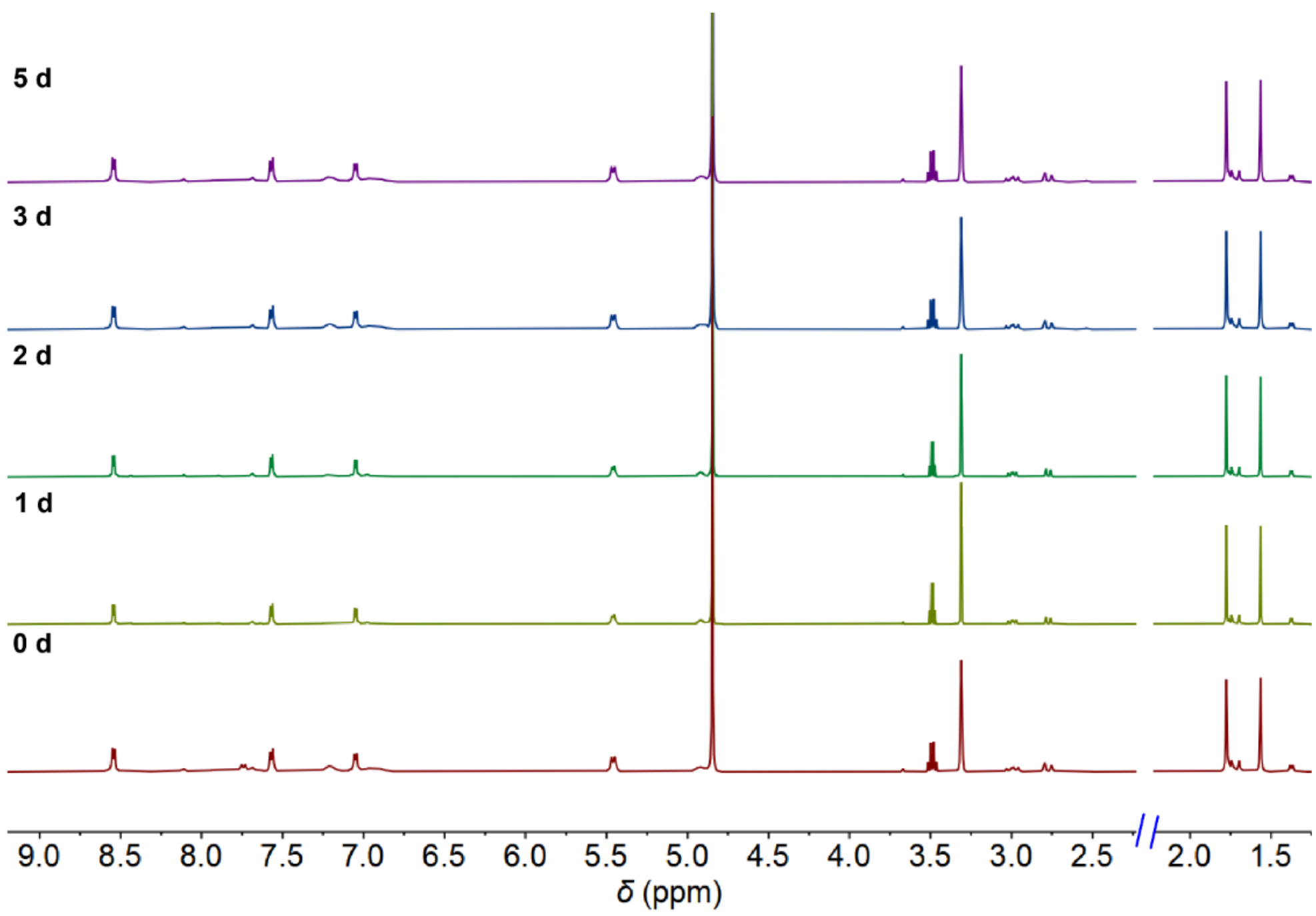

Fig. S69. ¹H NMR spectra of Λ-**Rh-1Br** recorded over 0–5 d (400 MHz, CD_3OD, c = 6.0 mM, room temperature). The spectral profile remains essentially unchanged over this period.

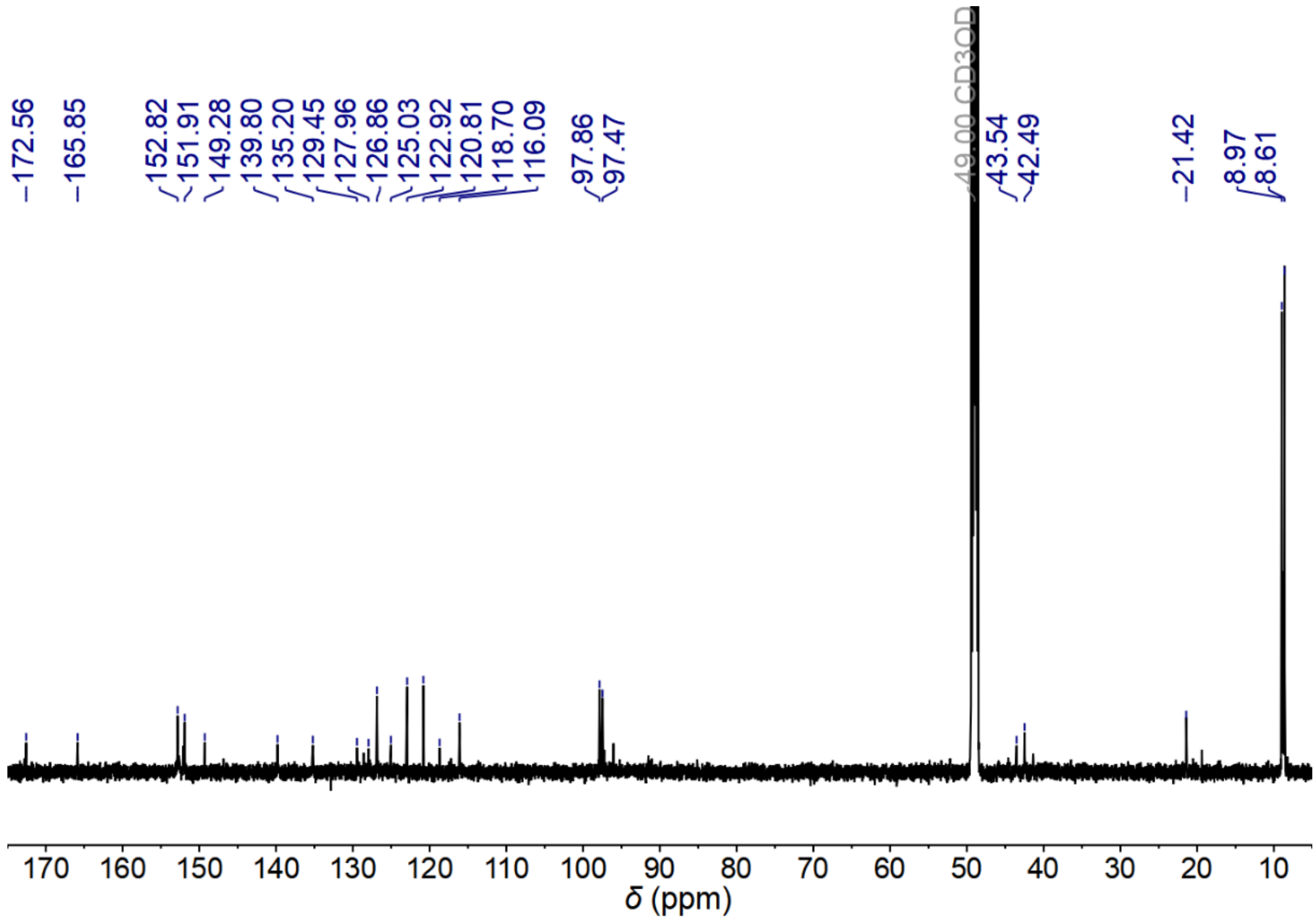

Fig. S70. $^{13}C\{^1H\}$ NMR spectrum of Λ-**Rh-1Br** in CD_3OD (151 MHz, c = 6.0 mM).

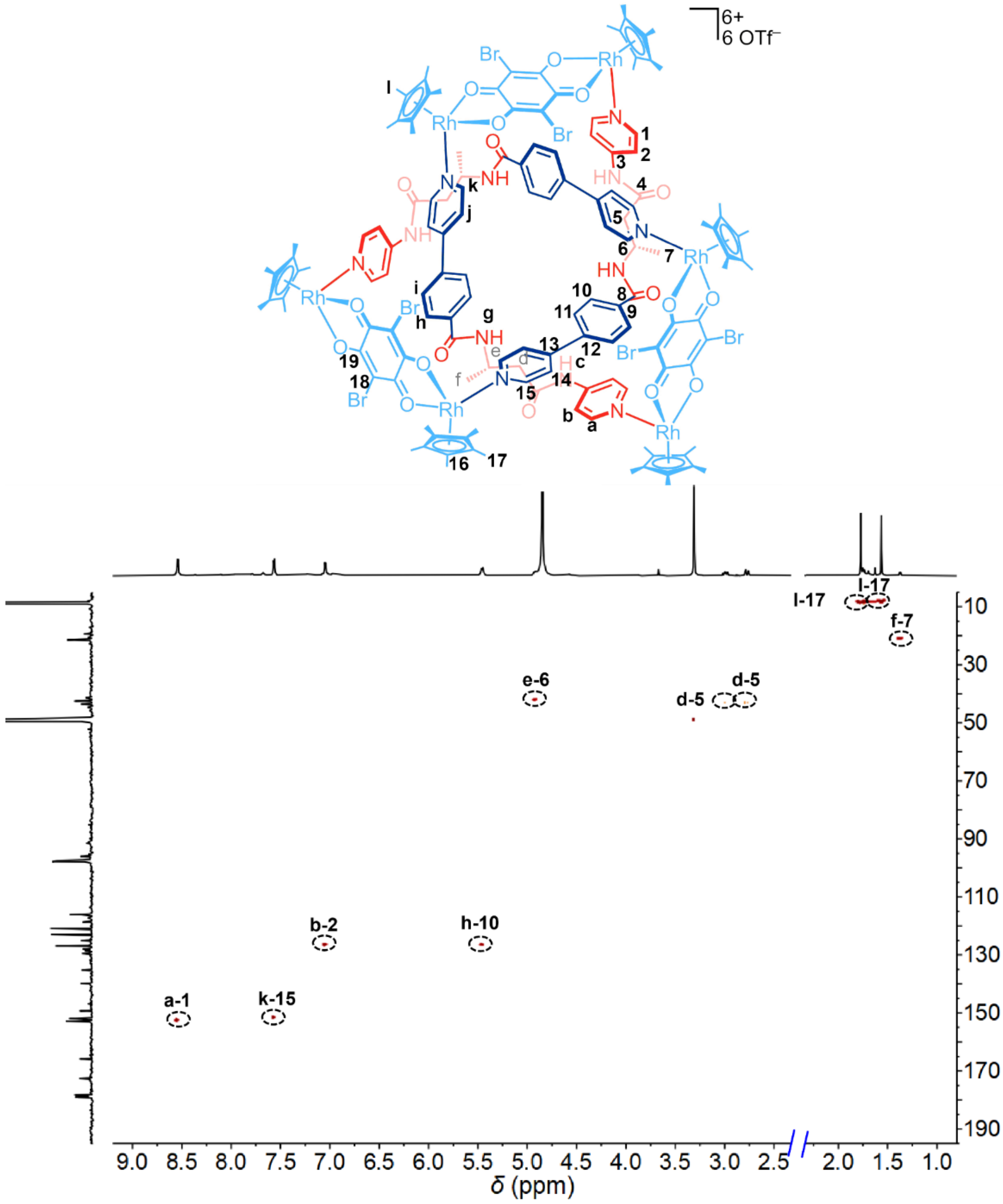

Fig. S71. ^{1}H–^{13}C HSQC spectrum of Λ-**Rh-1Br** in CD_3OD (600 MHz, c = 6.0 mM).

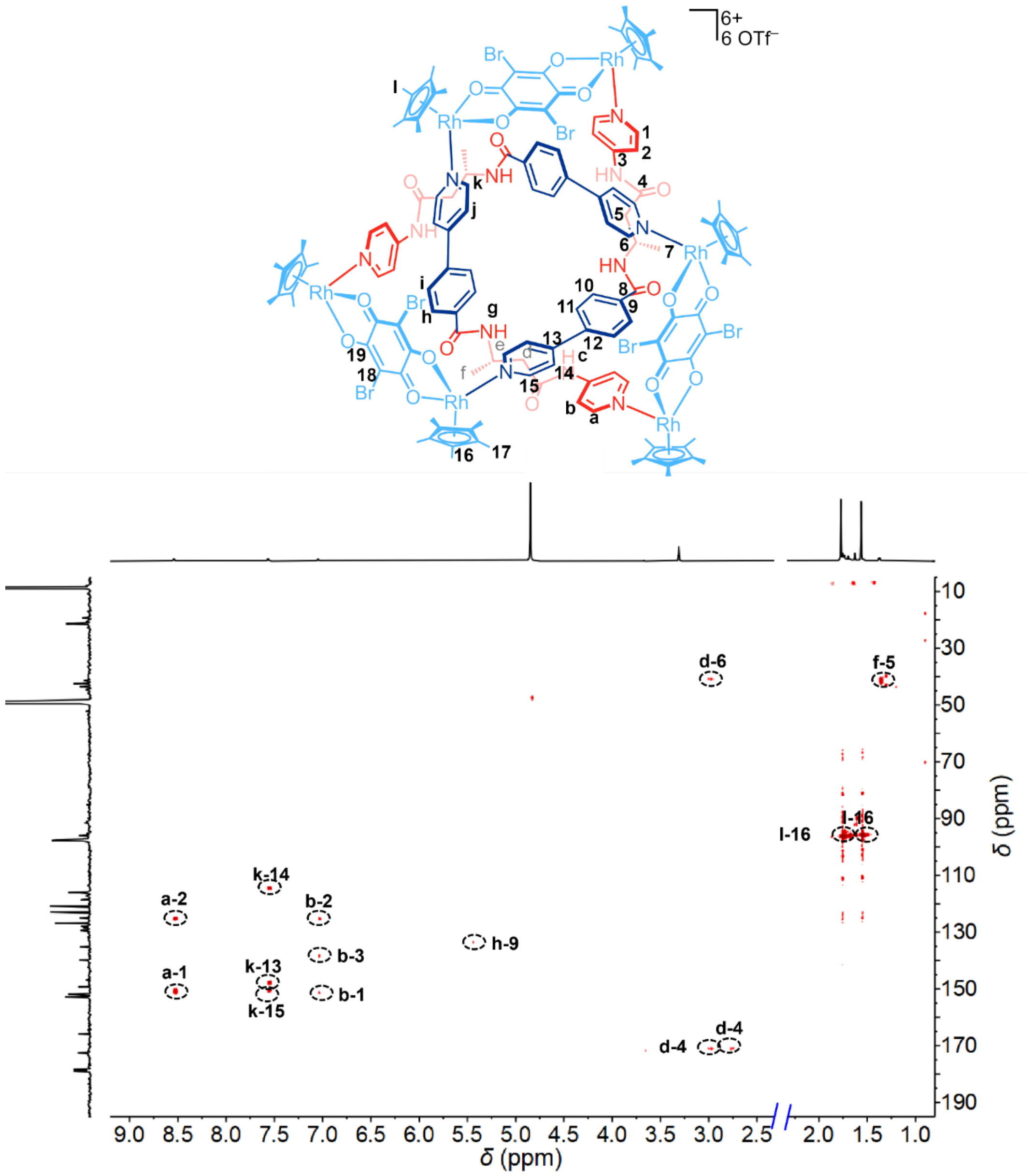

Fig. S72. ^{1}H–^{13}C HMBC spectrum of Λ-Rh-1Br in CD_3OD (600 MHz, c = 6.0 mM).

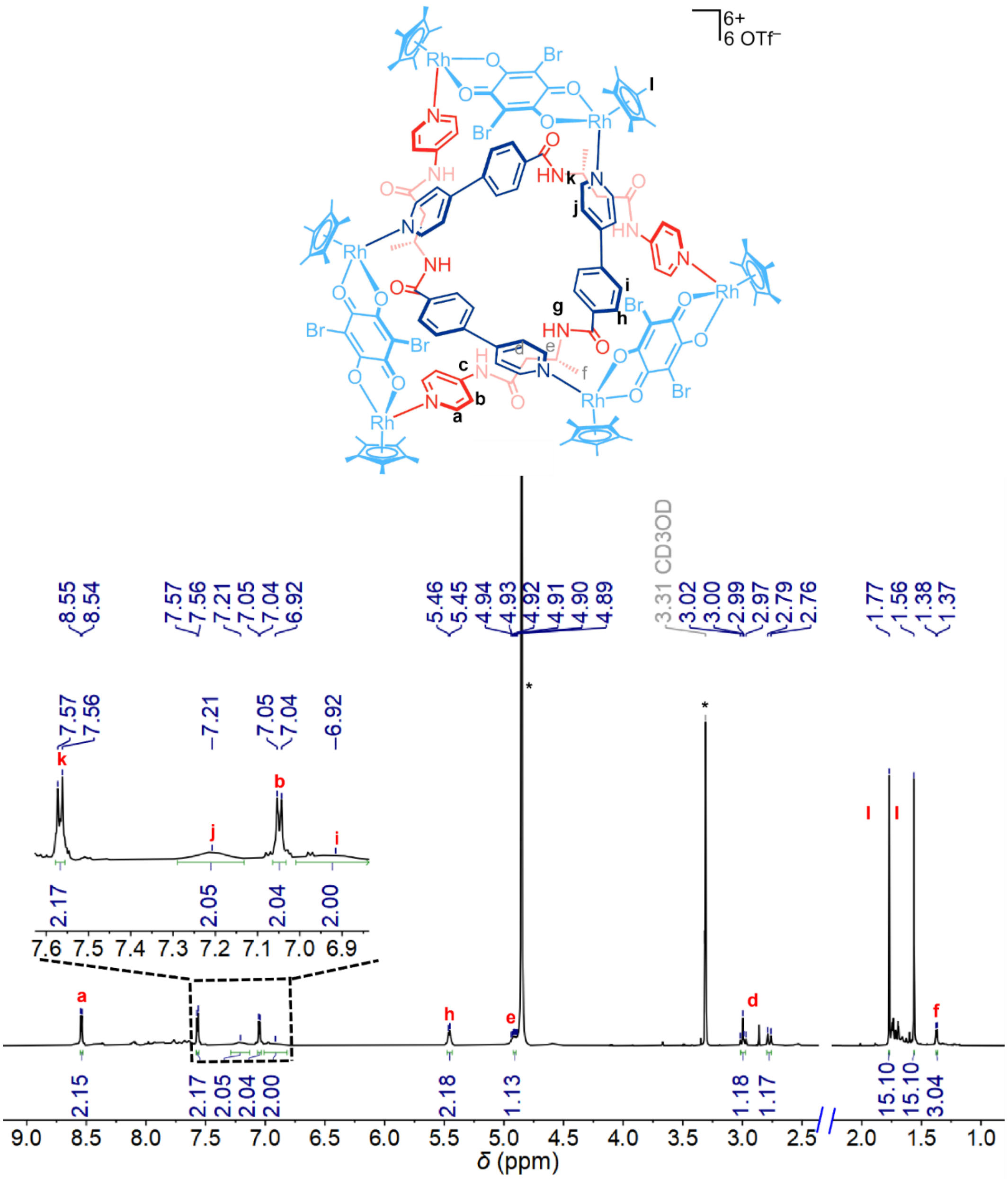

Fig. S73. ^{1}H NMR spectrum of Δ-**Rh-1Br** in CD_3OD (600 MHz, c = 6.0 mM, with respect to trefoil knots). The hydrogen protons of NH on the ligand lose their NMR signal due to the exchange with D in CD_3OD. * = solvent molecules.

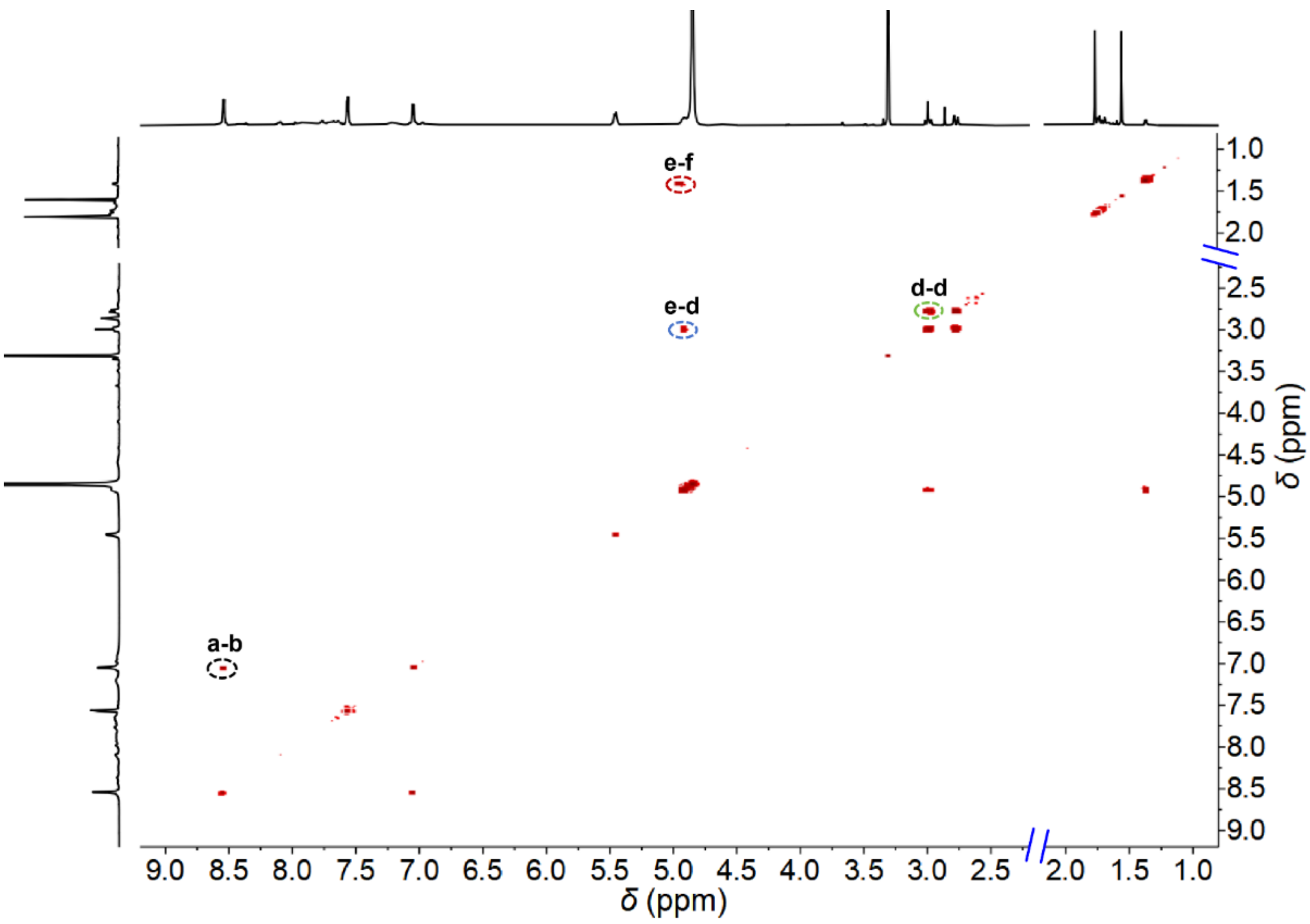

Fig. S74. ^{1}H-^{1}H COSY spectrum of Δ-**Rh-1Br** in CD_3OD (600 MHz, c = 6.0 mM, with respect to trefoil knots).

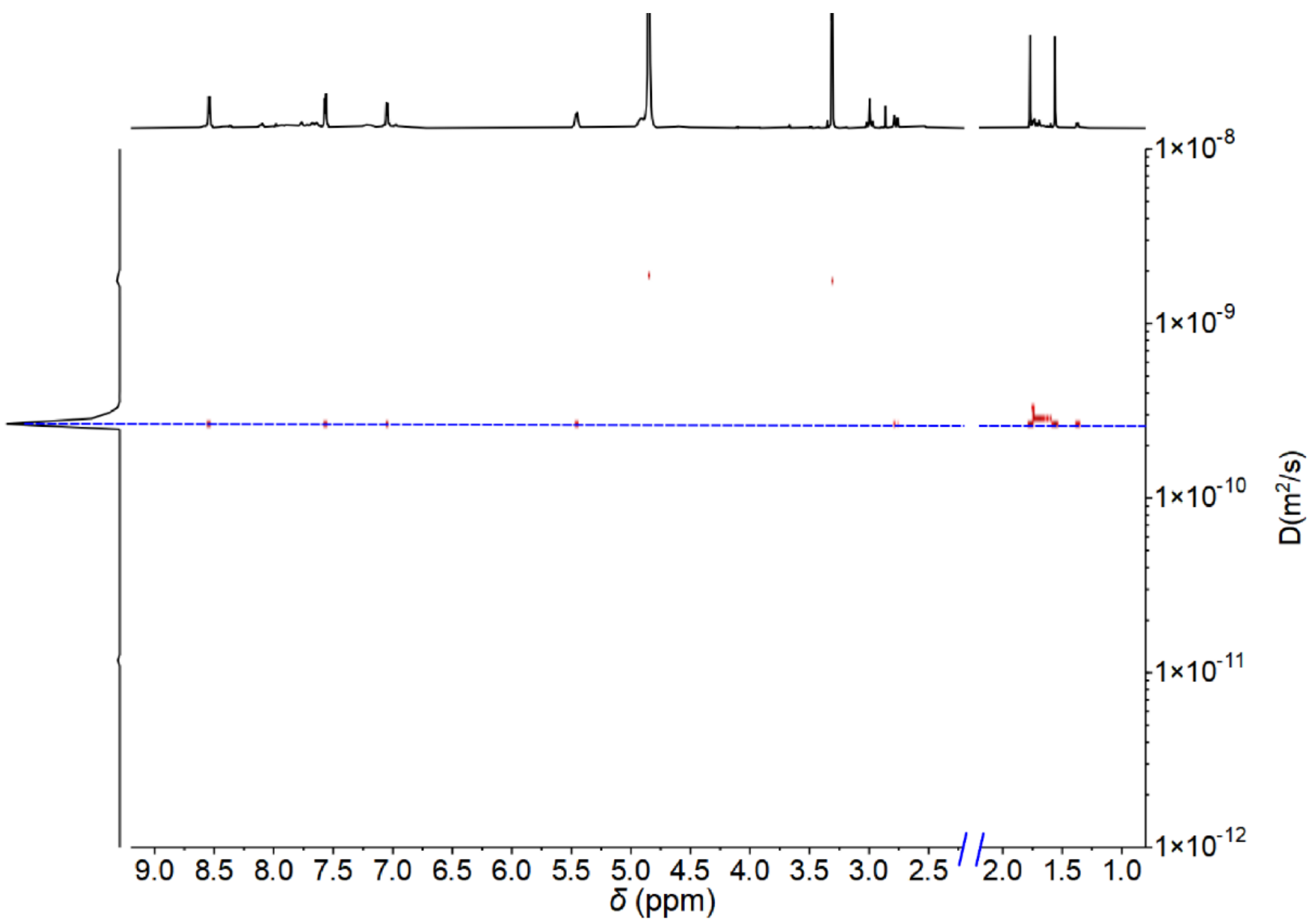

Fig. S75. ^{1}H DOSY spectrum of Δ-**Rh-1Br** in CD_3OD (600 MHz, c = 6.0 mM, where $c = c_{Rh}/6$). The assigned trefoil-knot resonances exhibit a diffusion coefficient of $D = 2.79 \times 10^{-10}$ m² s⁻¹.

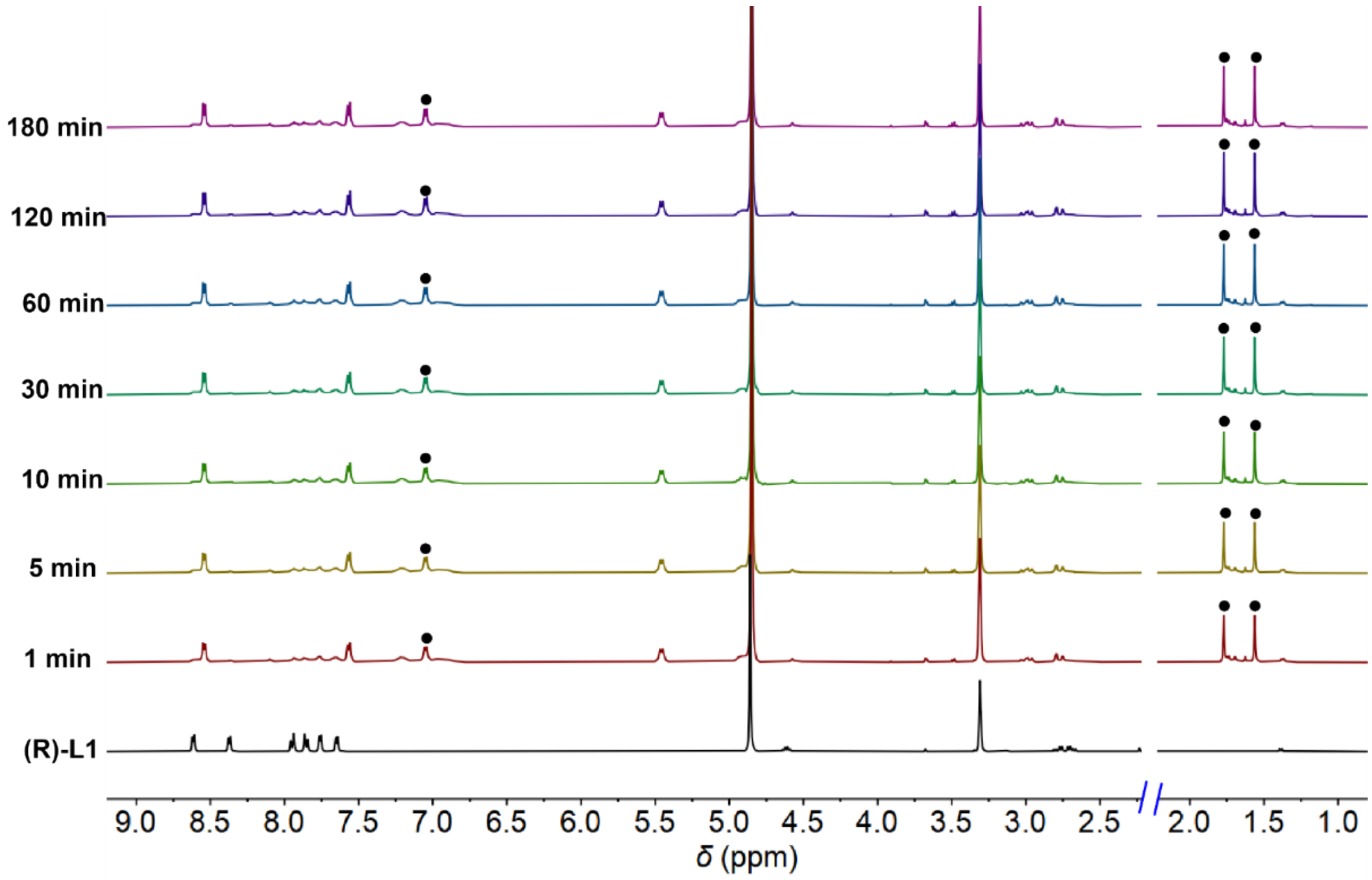

Fig. S76. Time-dependent ^{1}H NMR spectra monitoring self-assembly of (*R*)-**L1** with **B2**(OTf)$_2$ to form Δ-**Rh-1Br** (400 MHz, CD_3OD, room temperature; formal knot concentration = 6.0 mM). Formation is similarly rapid, with the characteristic knot resonances largely established within 5 min. Selected resonances corresponding to the trefoil-knot assemblies are highlighted with black circles.

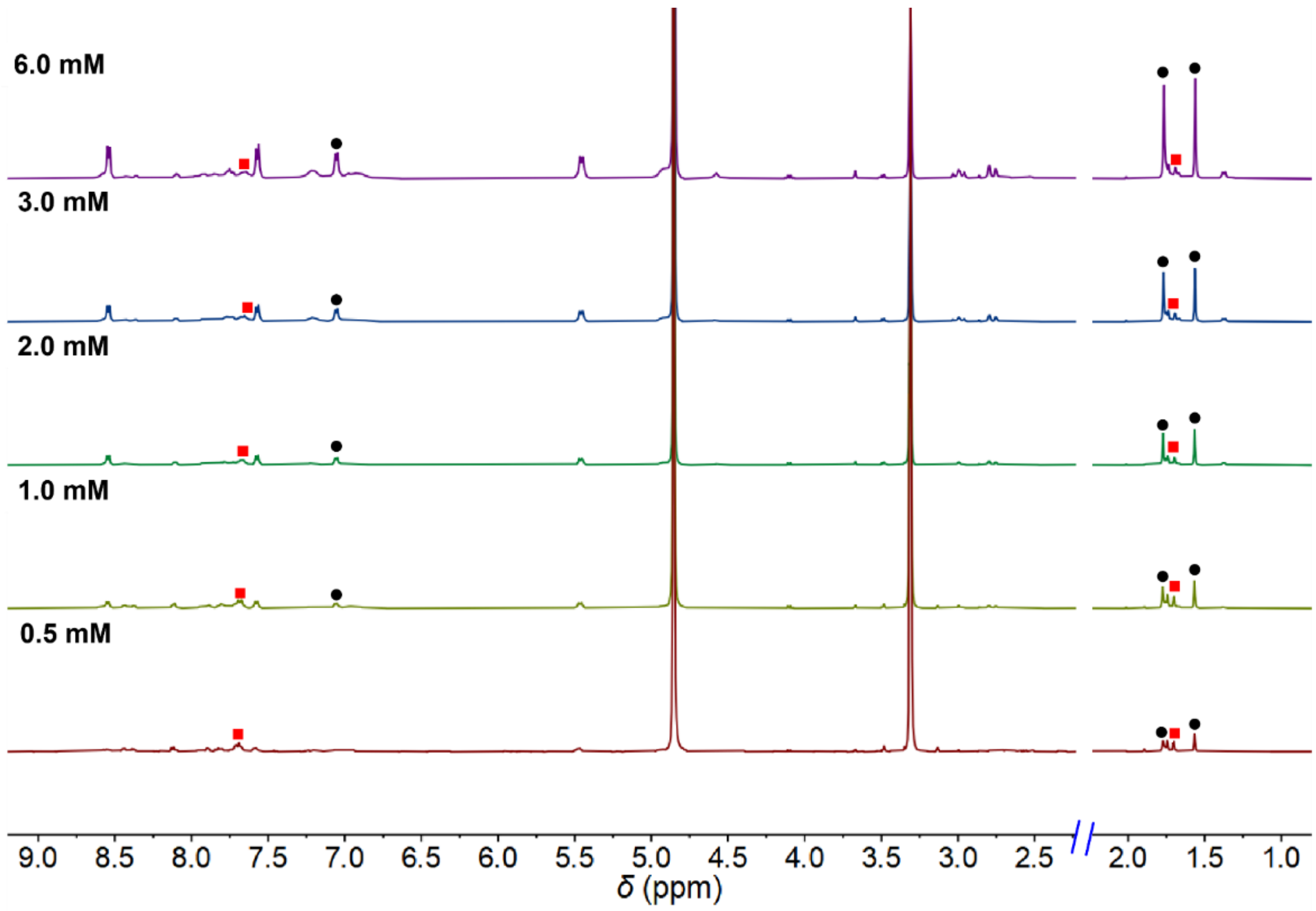

Fig. S77. Concentration-dependent ^{1}H NMR spectra of Δ-**Rh-1Br** over c = 0.5–6.0 mM (400 MHz, CD_3OD). Black circles mark selected resonances assigned to the trefoil knot, and red squares mark selected additional resonances tentatively assigned to tetranuclear metallomacrocyclic species. Upon dilution, the trefoil-knot resonances become less prominent relative to the additional resonances.

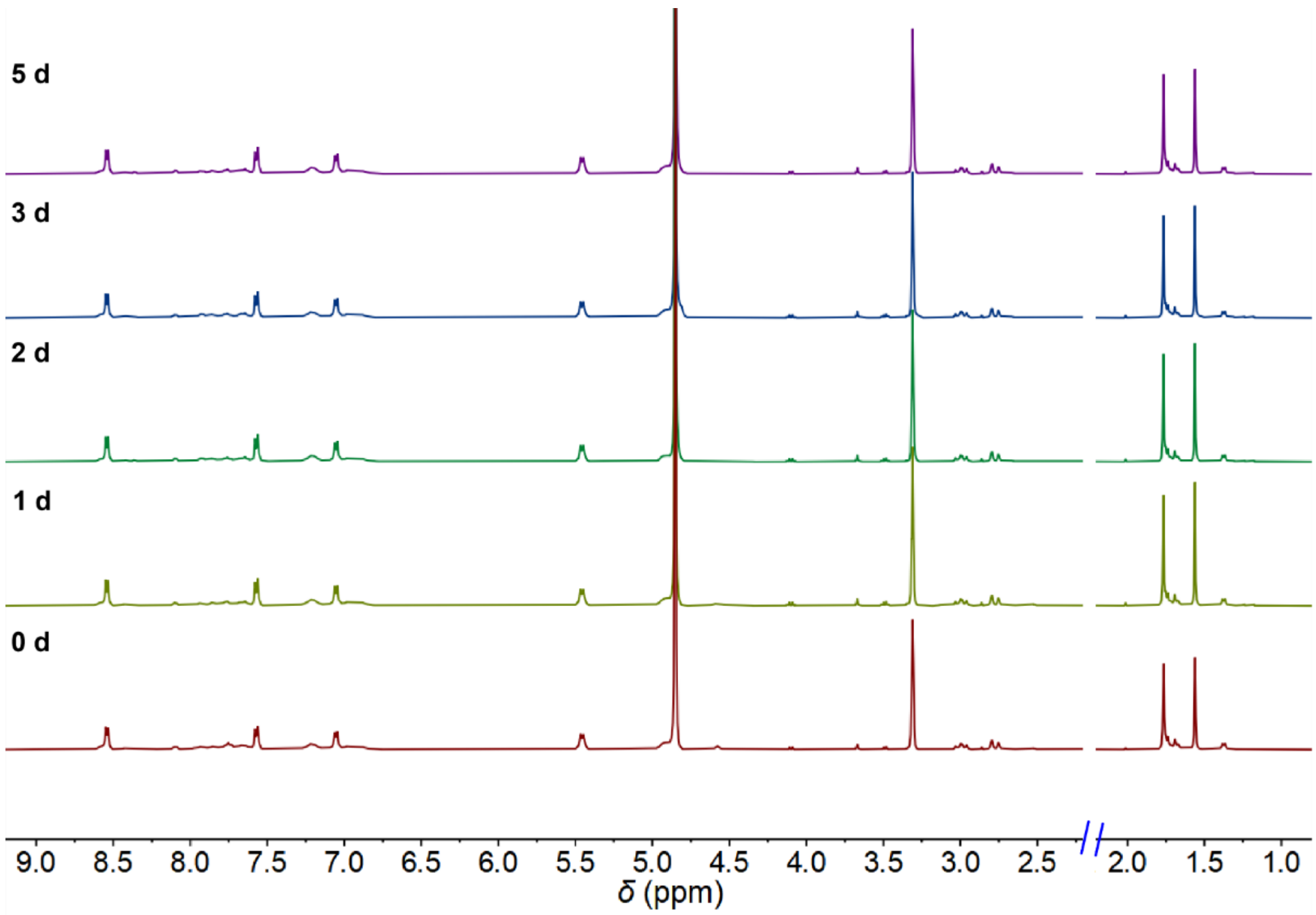

Fig. S78. ^{1}H NMR spectra of Δ-**Rh-1Br** recorded over 0–5 d (400 MHz, CD_3OD, c = 6.0 mM, room temperature). The spectral profile remains essentially unchanged over this period.

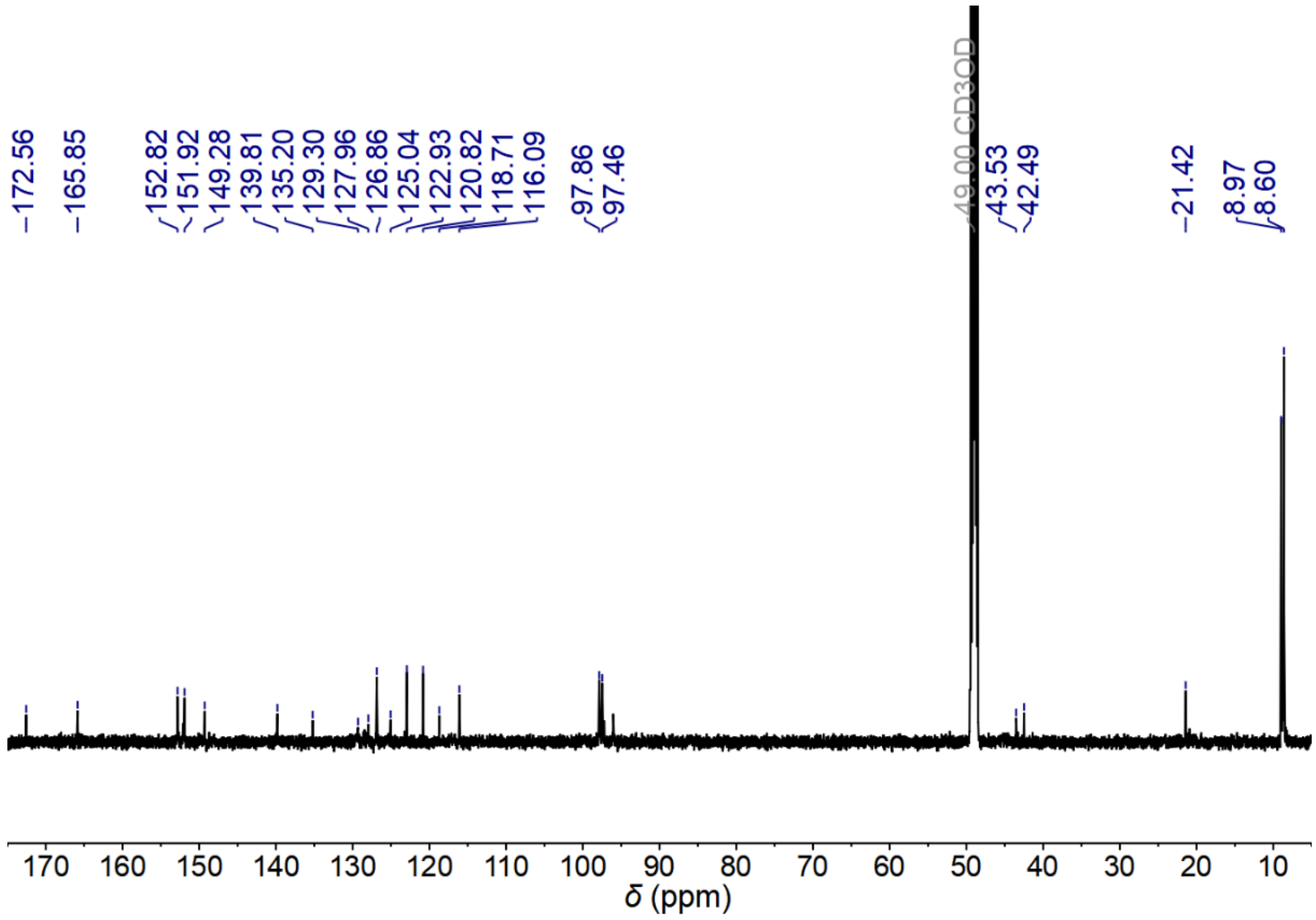

Fig. S79. $^{13}C\{^1H\}$ NMR spectrum of Δ-**Rh-1Br** in CD_3OD (151 MHz, *c* = 6.0 mM).

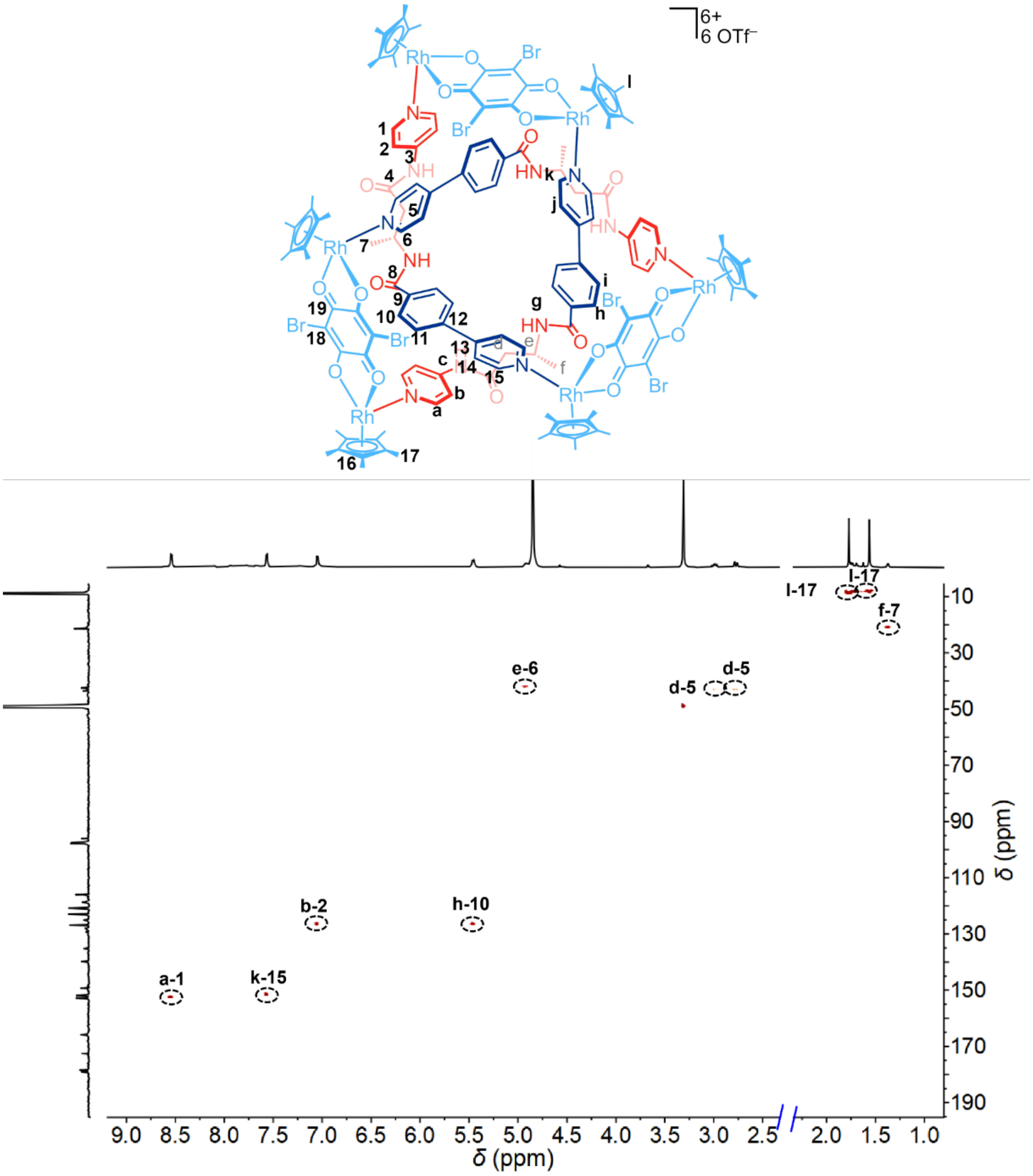

Fig. S80. ^{1}H–^{13}C HSQC spectrum of Δ-**Rh-1Br** in CD_3OD (600 MHz, c = 6.0 mM).

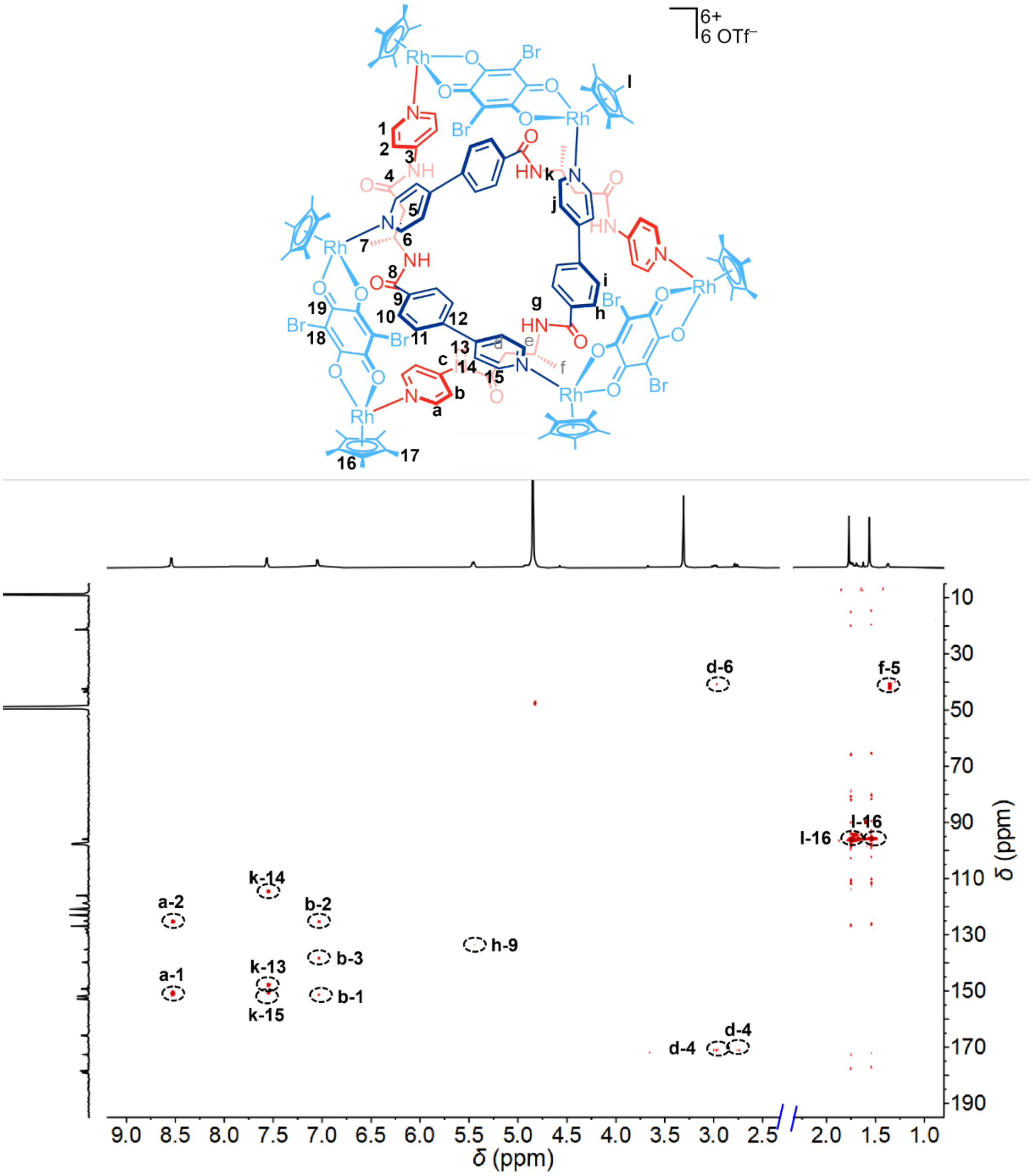

Fig. S81. ^1H–^{13}C HMBC spectrum of Δ-**Rh-1Br** in CD_3OD (600 MHz, c = 6.0 mM).

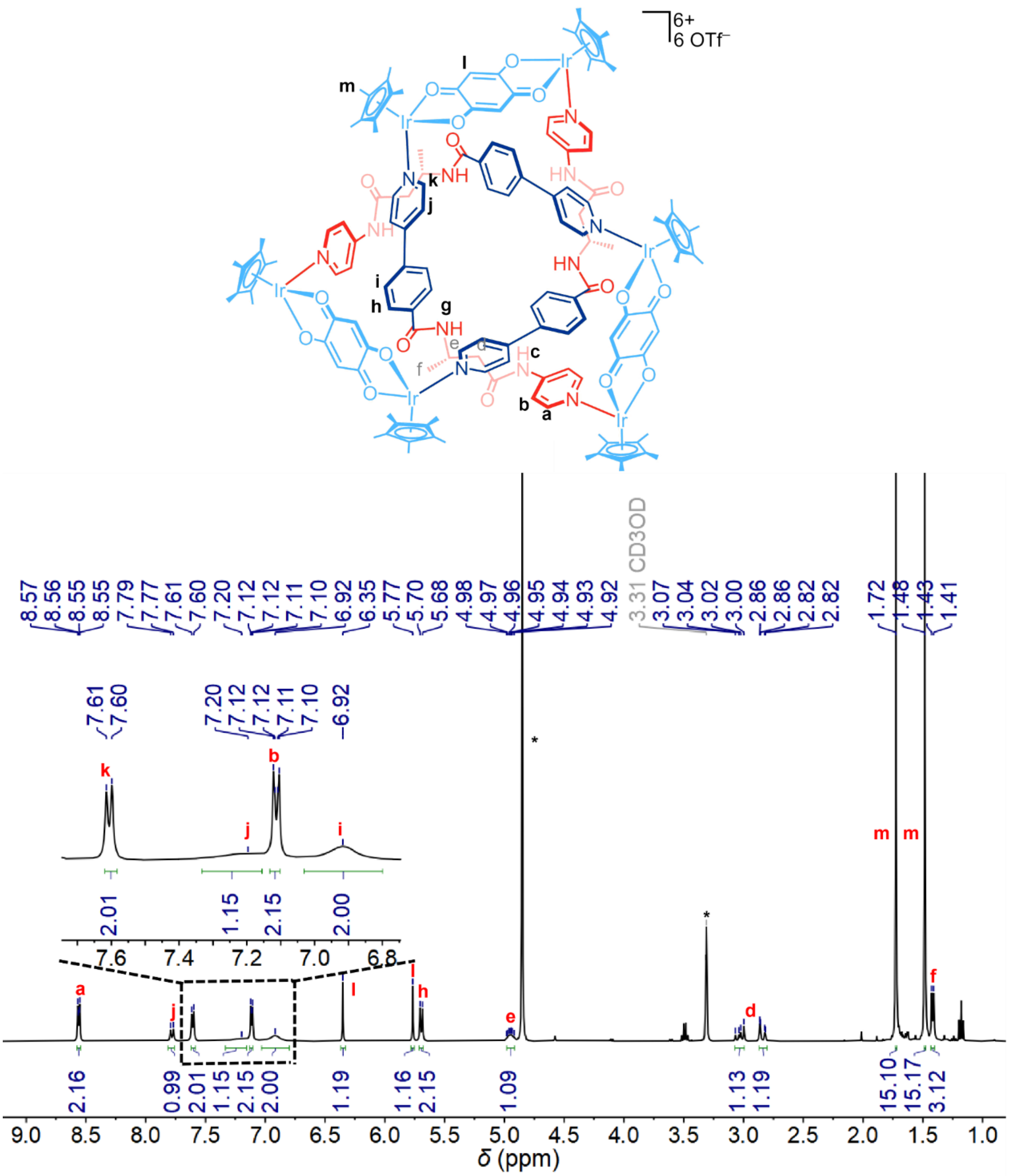

Fig. S82. ^{1}H NMR spectrum of Λ-**Ir-1** in CD_3OD (600 MHz, c = 6.0 mM, with respect to trefoil knots).

The hydrogen protons of NH on the ligand lose their NMR signal due to the exchange with D in CD_3OD.

* = solvent molecules.

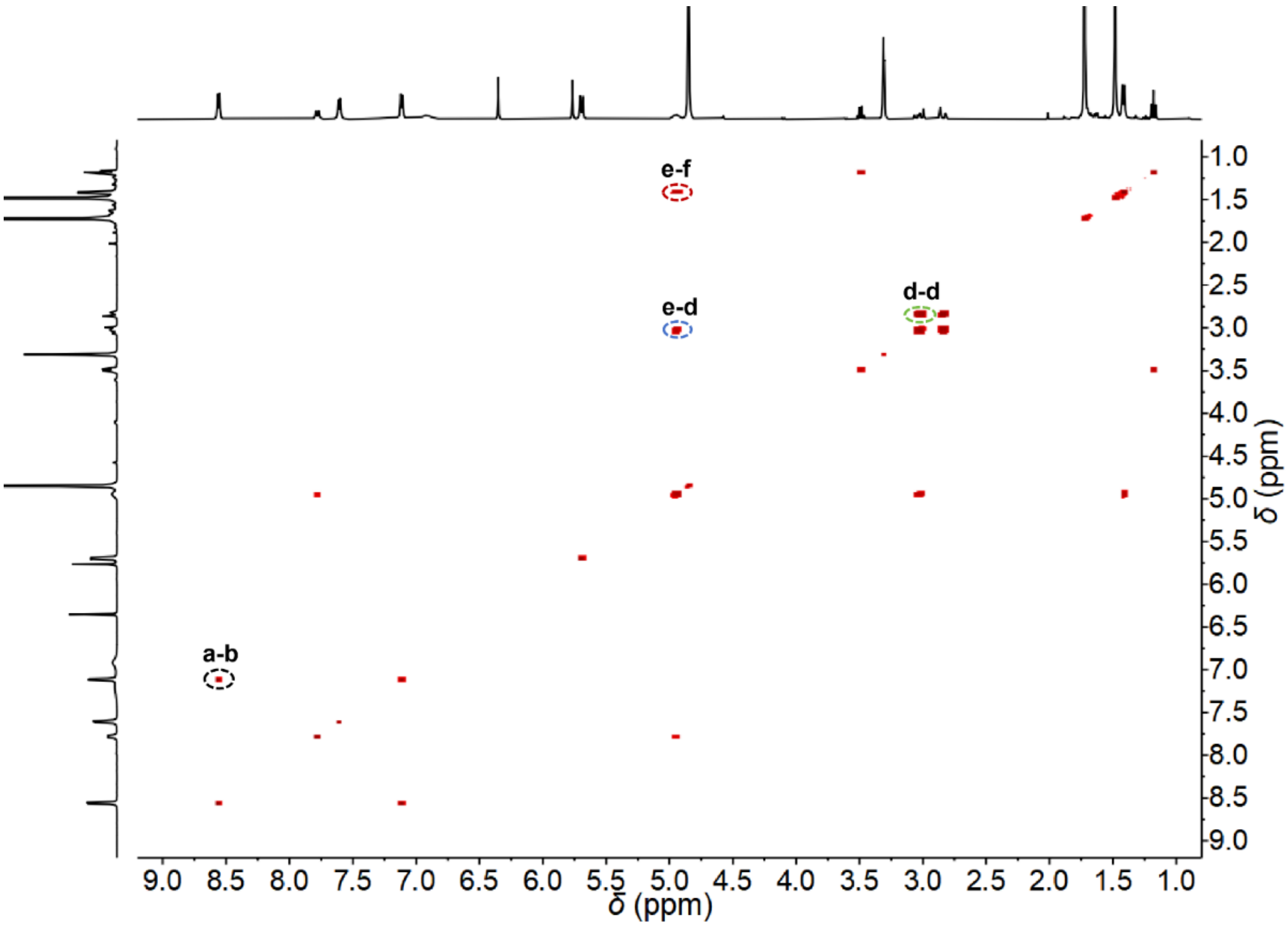

Fig. S83. ^{1}H-^{1}H COSY spectrum of Λ-**Ir-1** in CD_3OD (600 MHz, c = 6.0 mM, with respect to trefoil knots).

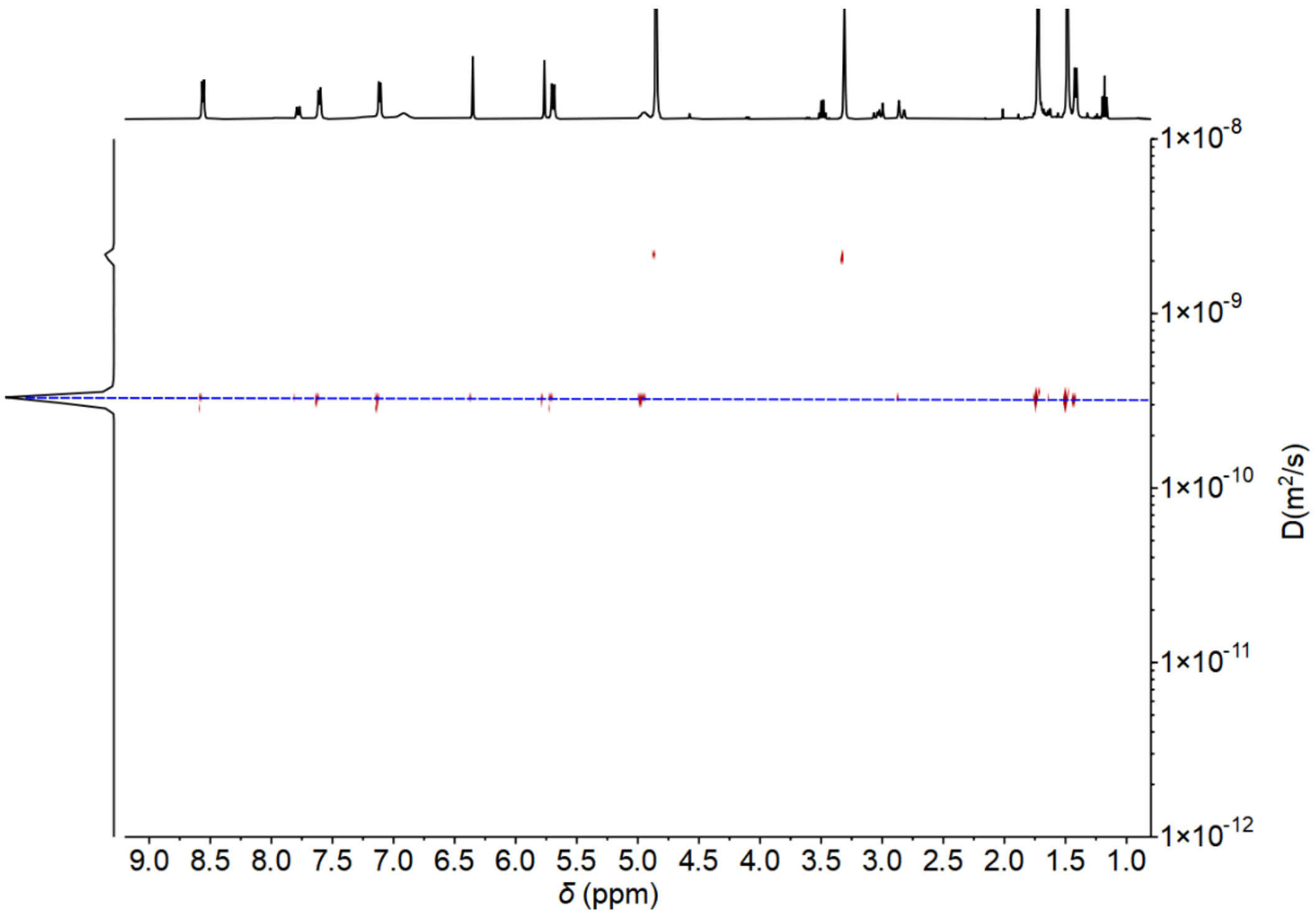

Fig. S84. ^{1}H DOSY spectrum of Λ-**Ir-1** in CD_3OD (600 MHz, c = 6.0 mM, where c = $c_{Ir}/6$). The assigned trefoil-knot resonances exhibit a diffusion coefficient of $D = 3.20 \times 10^{-10}$ m² s⁻¹.

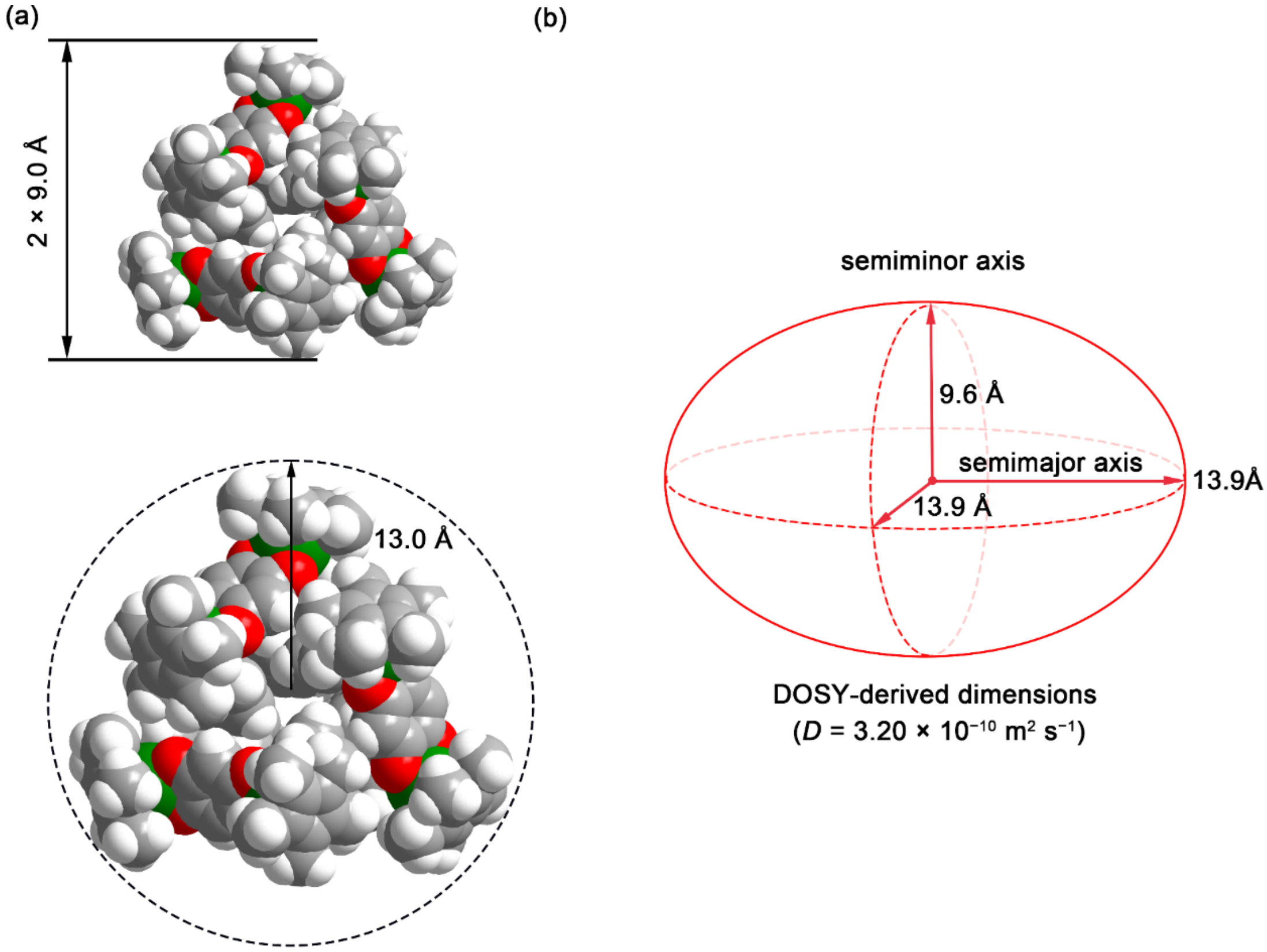

Fig. S85. Comparison of crystallographic and hydrodynamic dimensions for the trefoil knot Λ-**Ir-1** species in solution. (a) Crystallographic geometric reference extracted from the crystal structure of trefoil knot Λ-**Ir-1**(radius × half-height, 13.0 Å × 9.0 Å). (b) Hydrodynamic dimensions estimated from the DOSY diffusion coefficient of the trefoil knot Λ-**Ir-1** (D = 3.20 × 10^{-10} m^2 s^{-1}, Fig. S84) using a modified Stokes–Einstein treatment for an oblate spheroid model[4,5] (semimajor axis × semiminor axis, 13.9 Å × 9.6 Å). The estimated hydrodynamic dimensions are comparable to those of the trefoil knot Λ-**Ir-1** extracted from the crystal structure.

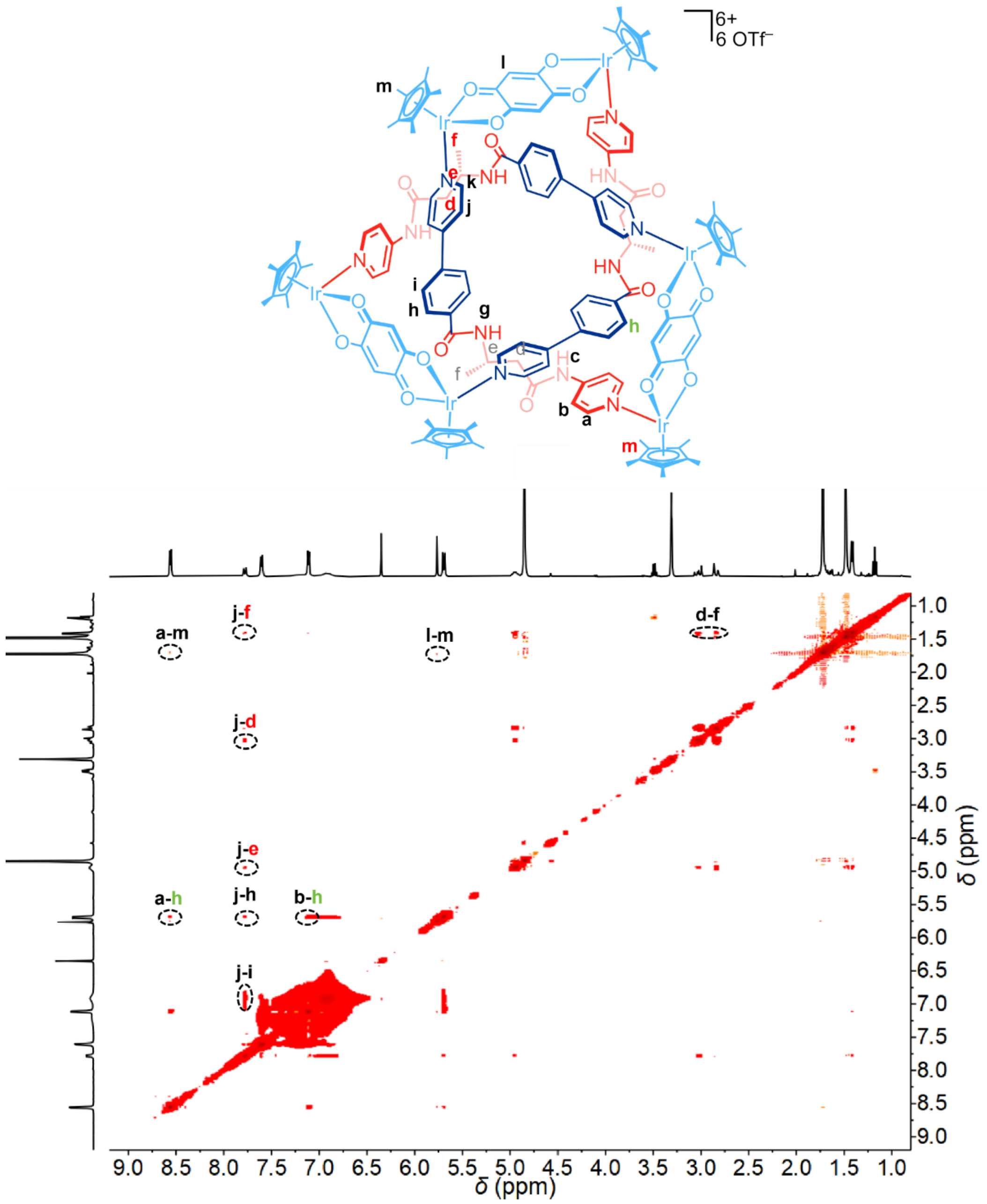

Fig. S86. ^1H-^1H NOESY NMR (600 MHz, CD_3OD, c = 6.0 mM) for Λ-**Ir-1**.

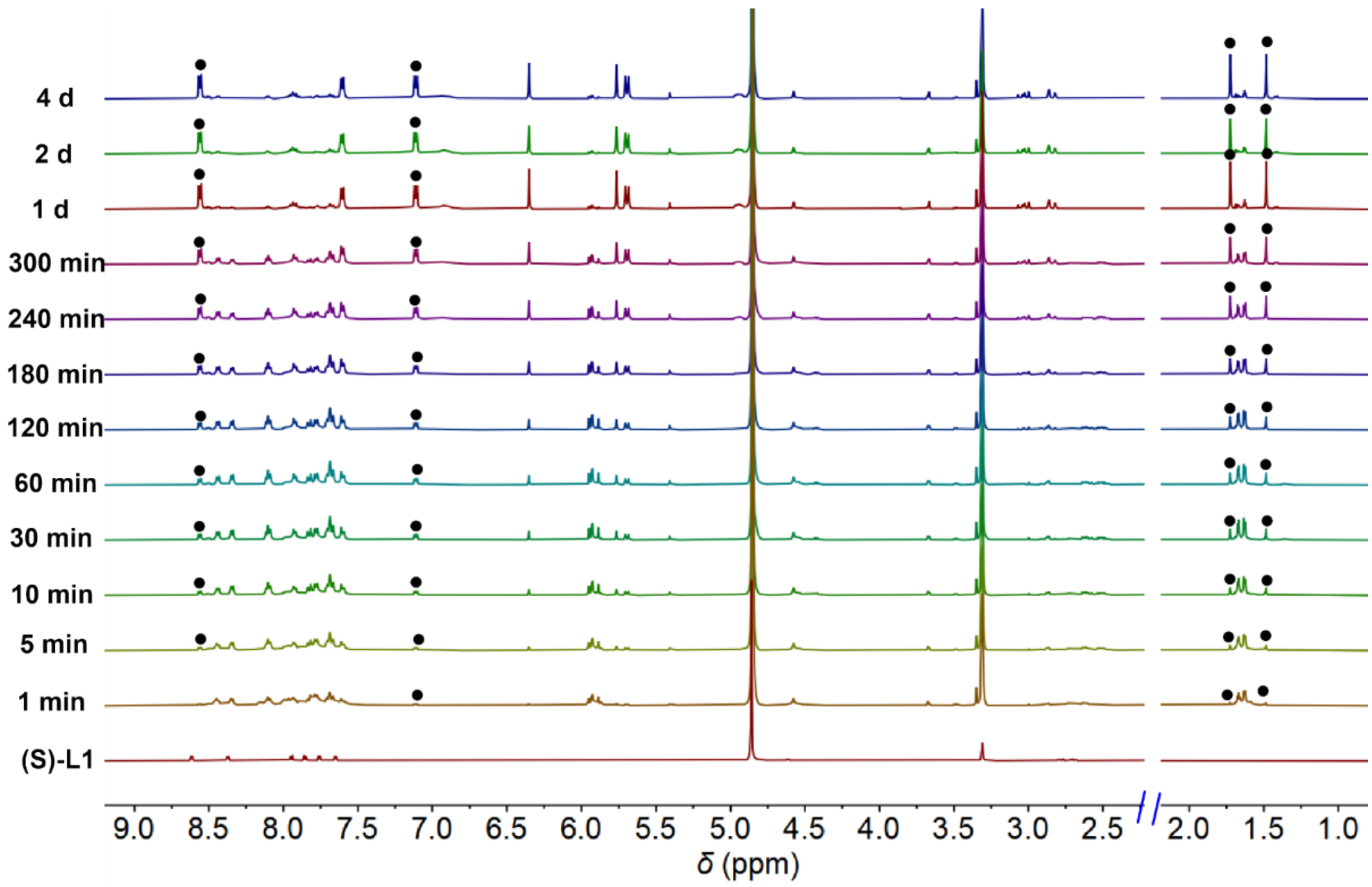

Fig. S87. Time-dependent ^{1}H NMR spectra monitoring self-assembly of (*S*)-**L1** with **B3**(OTf)$_2$ to form Λ-**Ir-1** (400 MHz, CD_3OD, room temperature; formal knot concentration = 6.0 mM). Formation is largely complete after approximately 1d. Selected resonances corresponding to the trefoil-knot assemblies are highlighted with black circles.

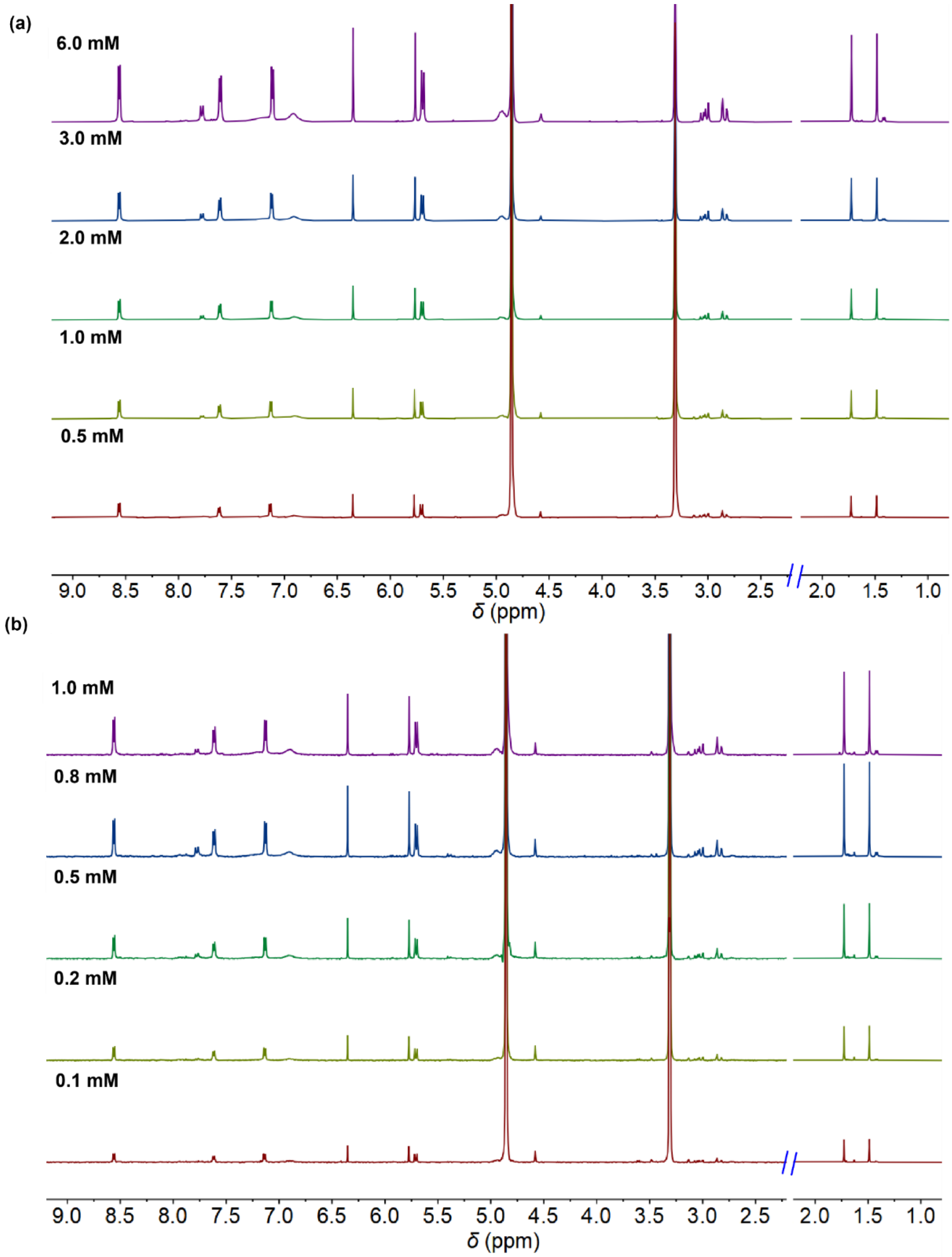

Fig. S88. Concentration-dependent ^{1}H NMR spectra of Λ-**Ir-1** (400 MHz, CD_3OD): (a) $c = 0.5–6.0$ mM and (b) $c = 0.1–1.0$ mM. The spectra show no substantial changes in the positions or relative intensities of the assigned assembly resonances over the concentration range examined.

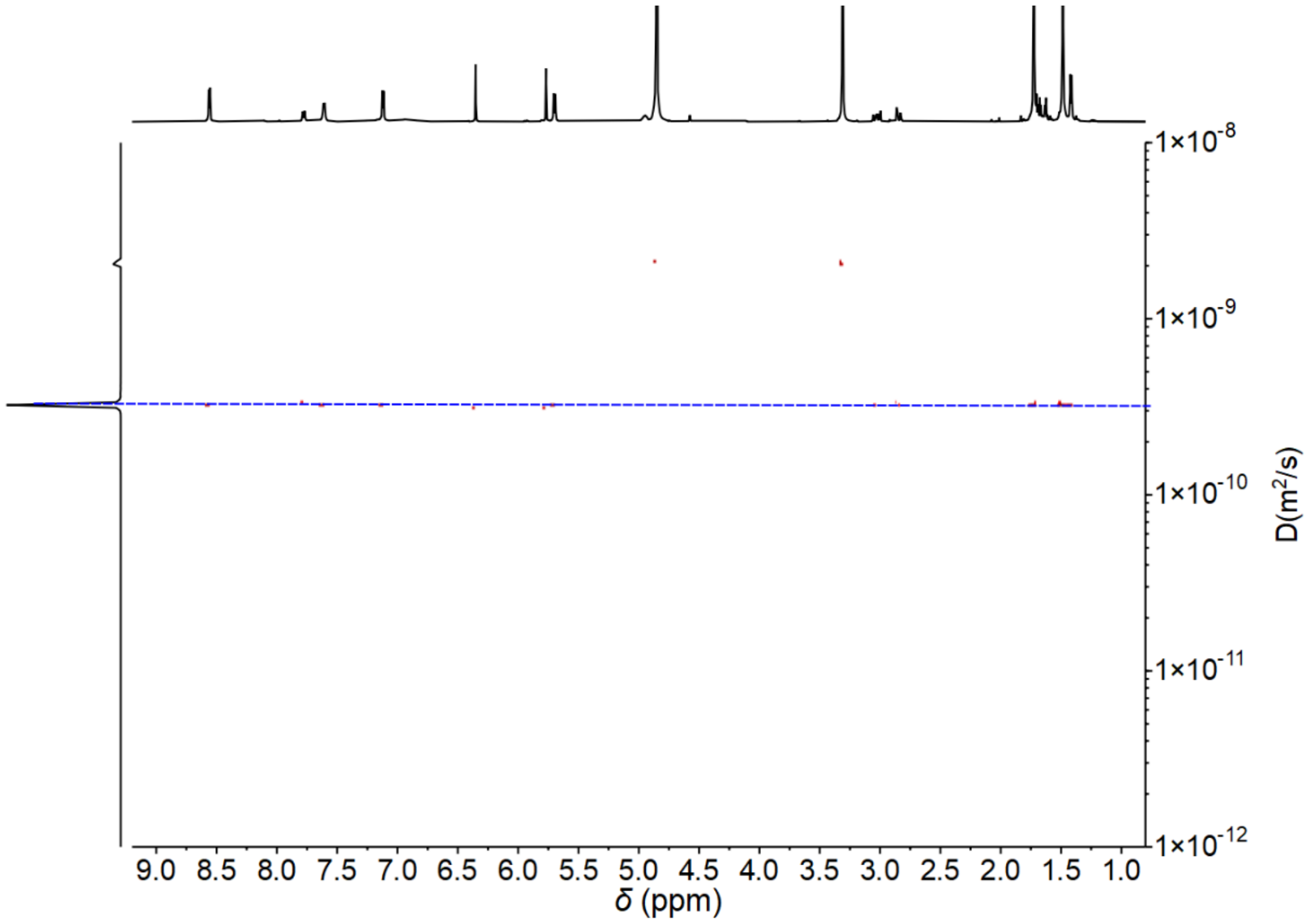

Fig. S89. ^{1}H DOSY spectrum of Λ-**Ir-1** in CD_3OD (600 MHz, c = 3.0 mM, where $c = c_{Ir}/6$). The assigned trefoil-knot resonances exhibit a diffusion coefficient of $D = 3.23 \times 10^{-10}$ m^2 s^{-1}.

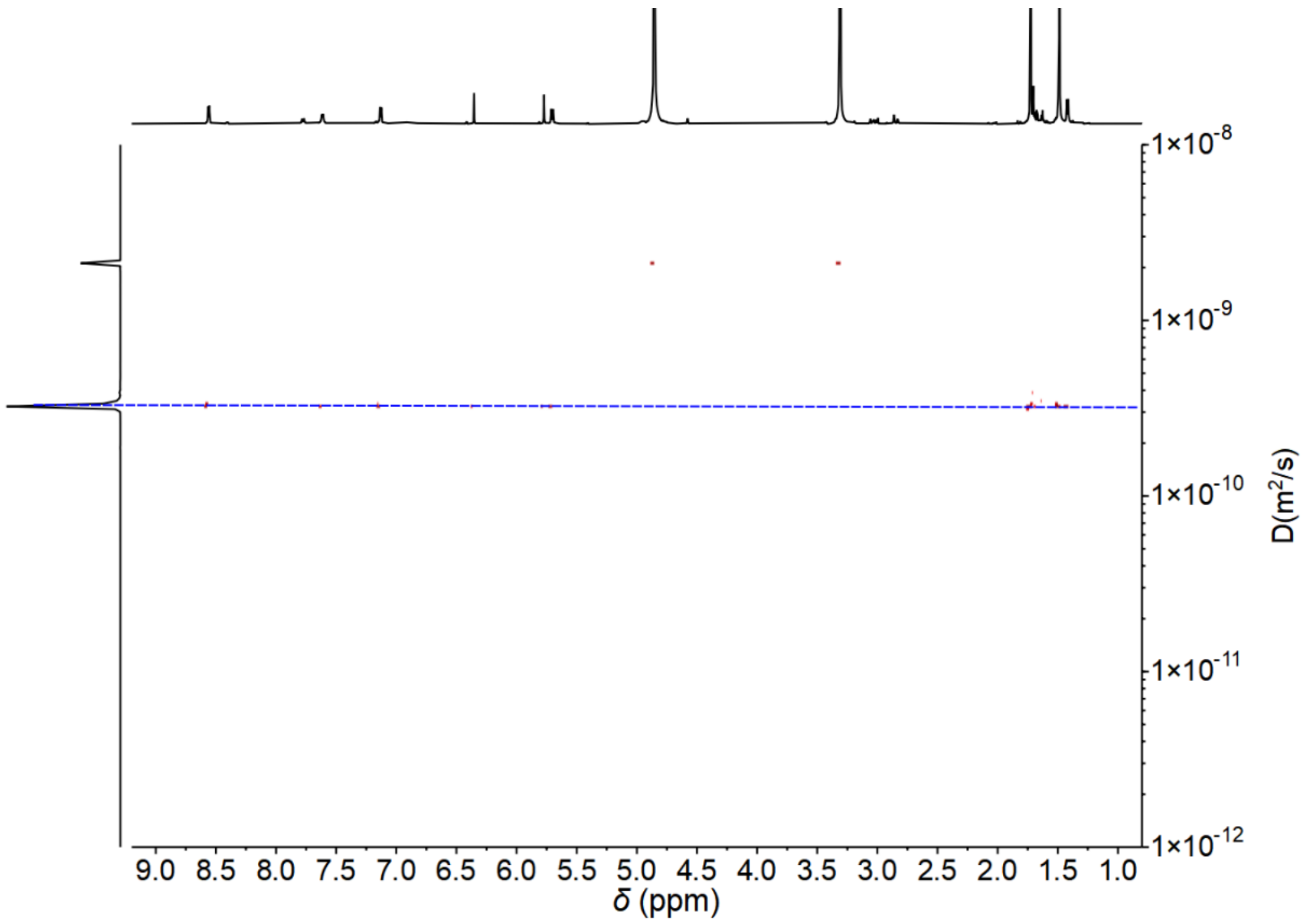

Fig. S90. ^{1}H DOSY spectrum of Λ-**Ir-1** in CD_3OD (600 MHz, c = 1.0 mM, where $c = c_{Ir}/6$). The assigned trefoil-knot resonances exhibit a diffusion coefficient of $D = 3.23 \times 10^{-10}$ m^2 s^{-1}.

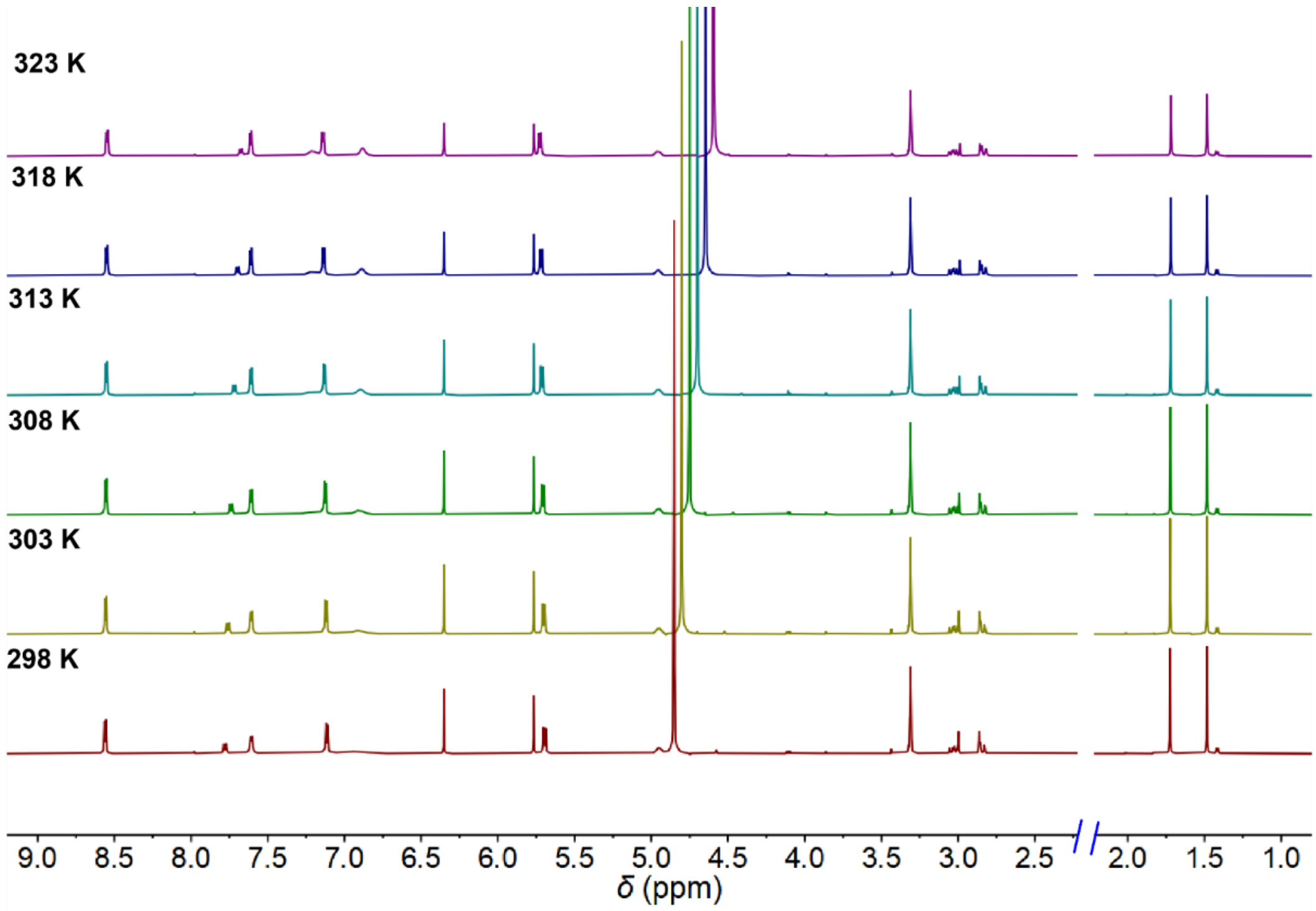

Fig. S91. Variable-temperature ^{1}H NMR spectra of Λ-**Ir-1** in CD_3OD (600 MHz, 298–323 K; c = 6.0 mM). The spectra show no substantial changes over the temperature range examined.

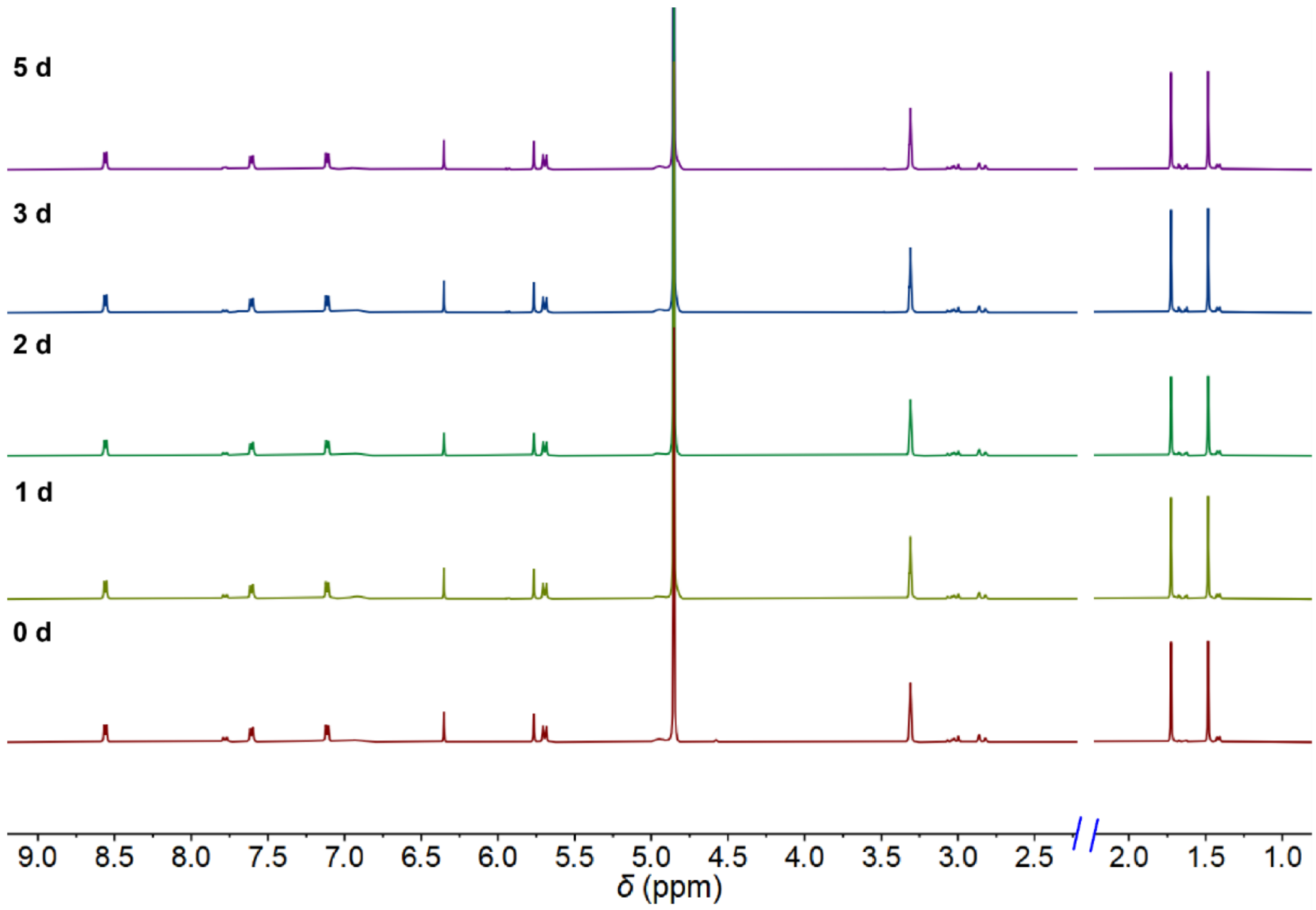

Fig. S92. ^{1}H NMR spectra of Λ-**Ir-1** recorded over 0–5 d in CD_3OD at room temperature (400 MHz, c = 6.0 mM). The spectra remain essentially unchanged over this period.

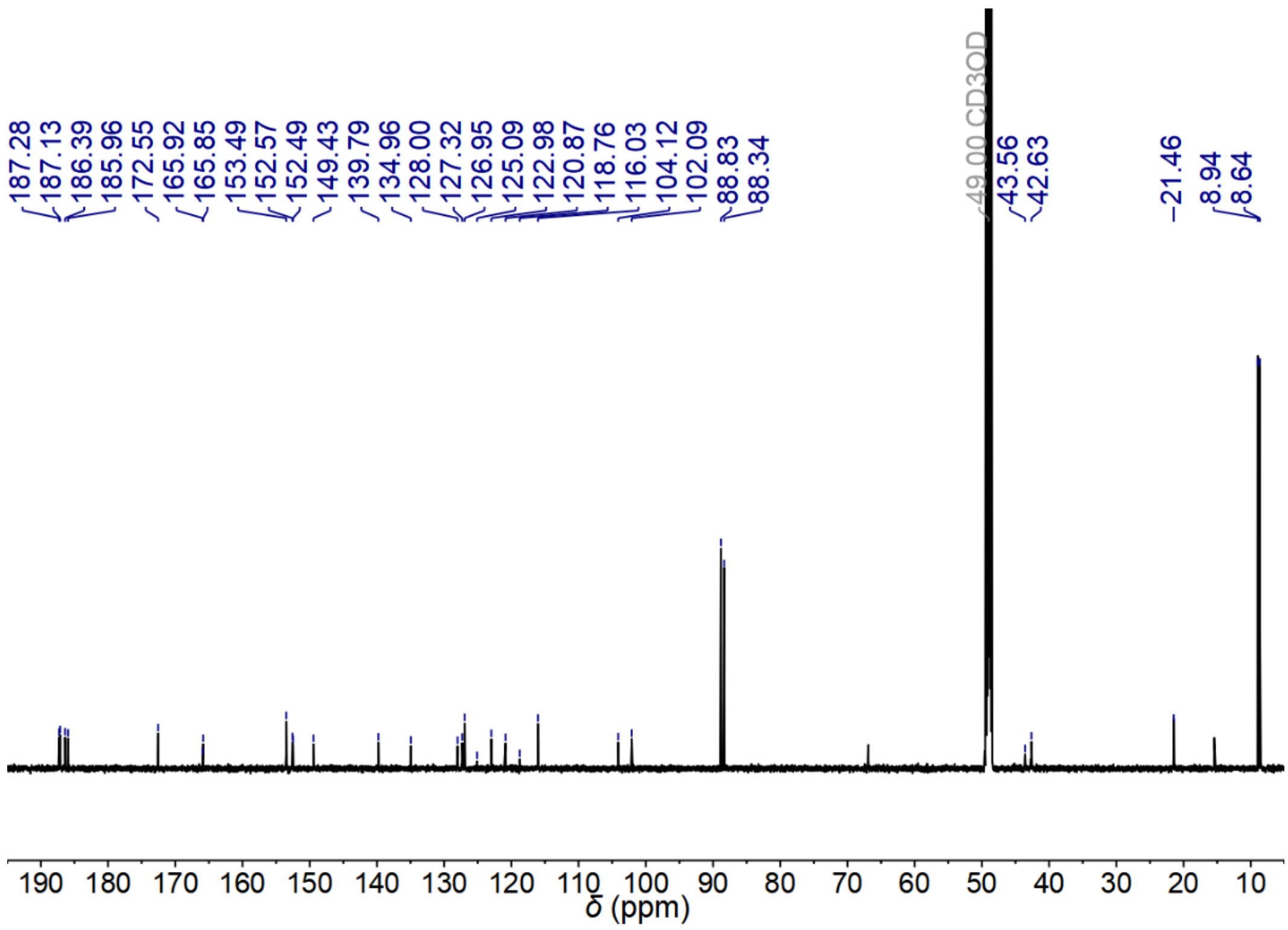

Fig. S93. $^{13}C\{^{1}H\}$ NMR spectrum of Λ-**Ir-1** in CD_3OD (151 MHz, c = 6.0 mM).

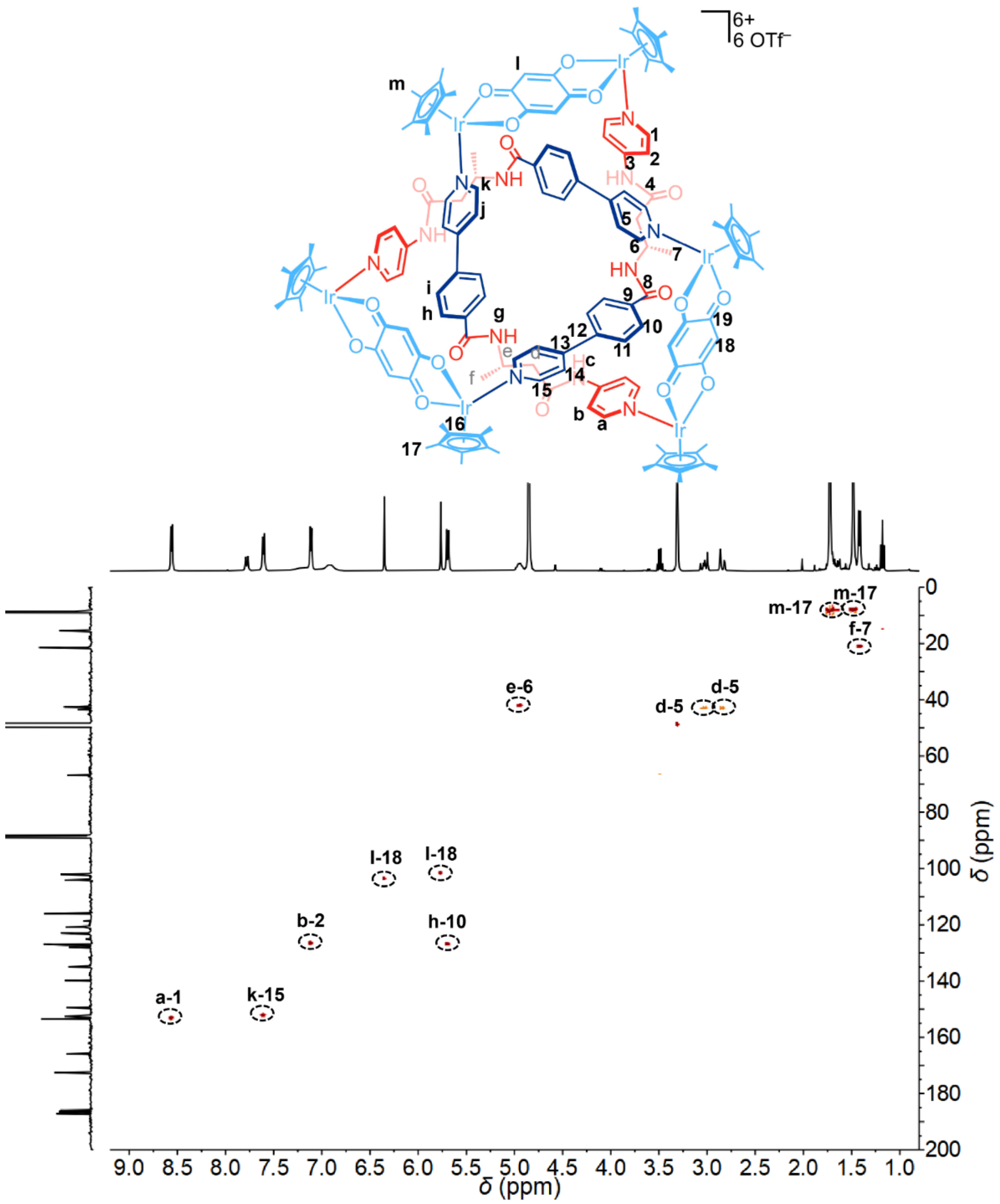

Fig. S94. ^{1}H–^{13}C HSQC spectrum of Λ-**Ir-1** in CD_3OD (600 MHz, c = 6.0 mM).

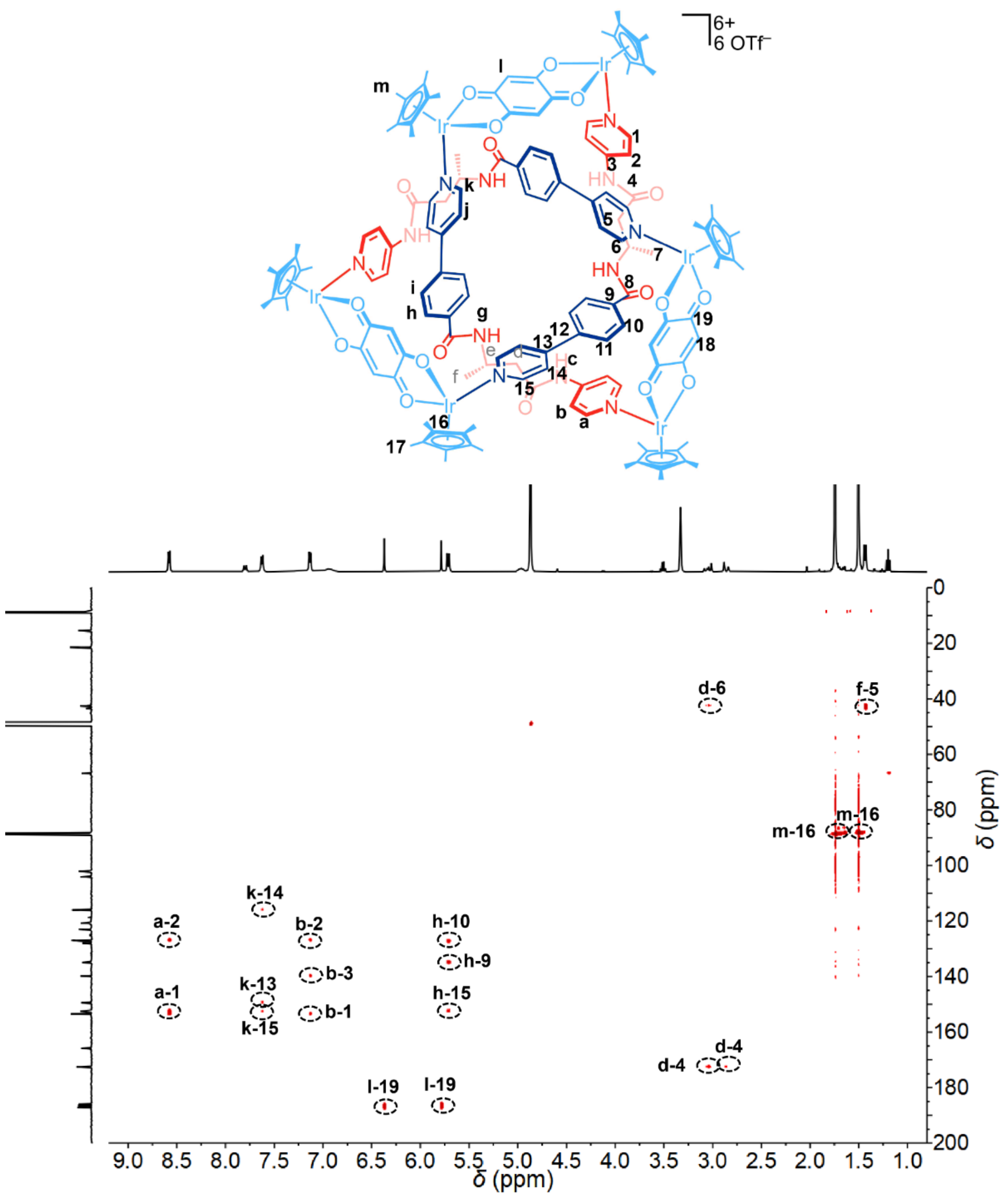

Fig. S95. ^{1}H–^{13}C HMBC spectrum of Λ-**Ir-1** in CD_3OD (600 MHz, c = 6.0 mM).

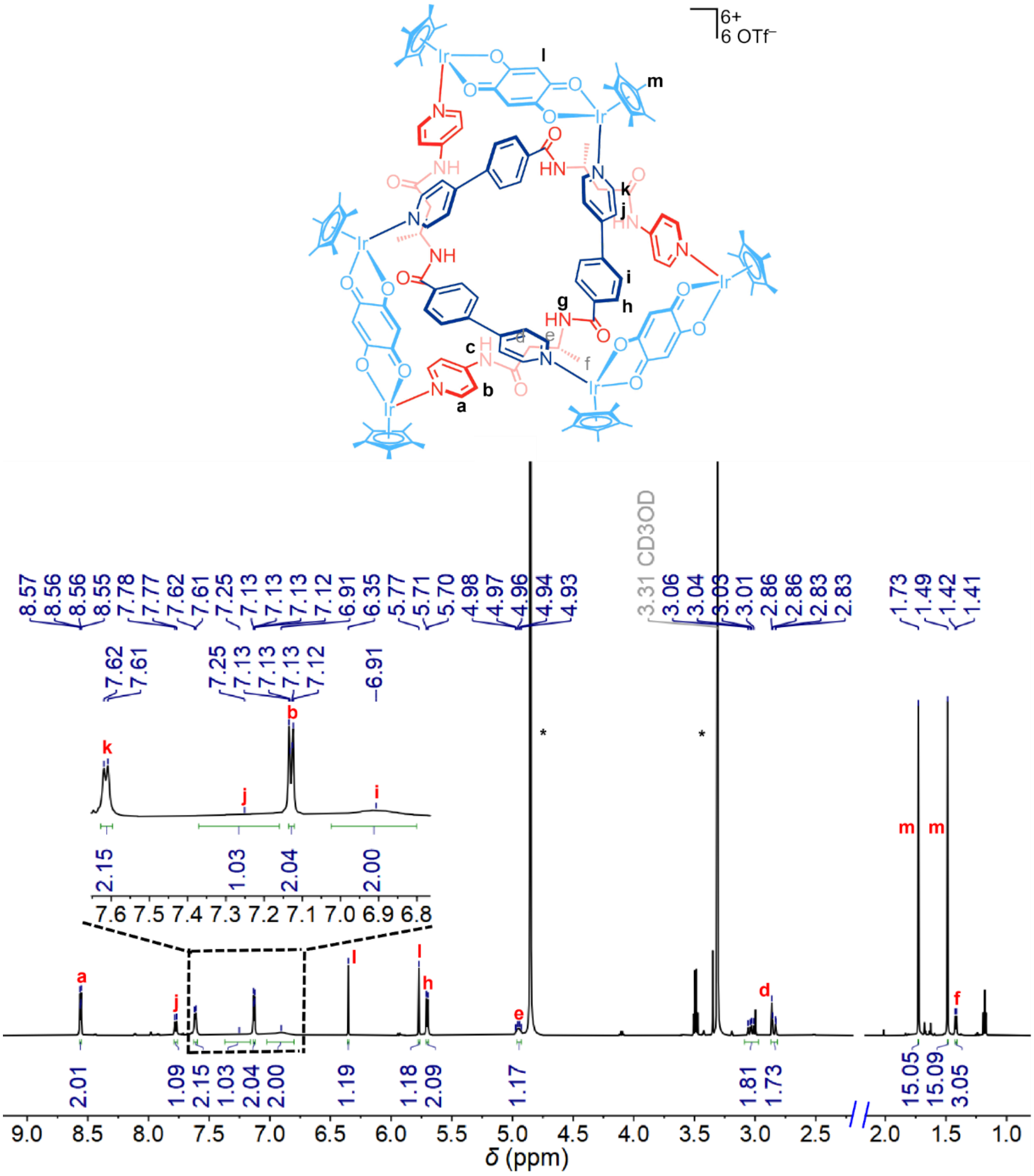

Fig. S96. ^{1}H NMR spectrum of Δ-**Ir-1** in CD_3OD (600 MHz, c = 1.4 mM, with respect to trefoil knots).

The hydrogen protons of NH on the ligand lose their NMR signal due to the exchange with D in CD_3OD.

* = solvent molecules.

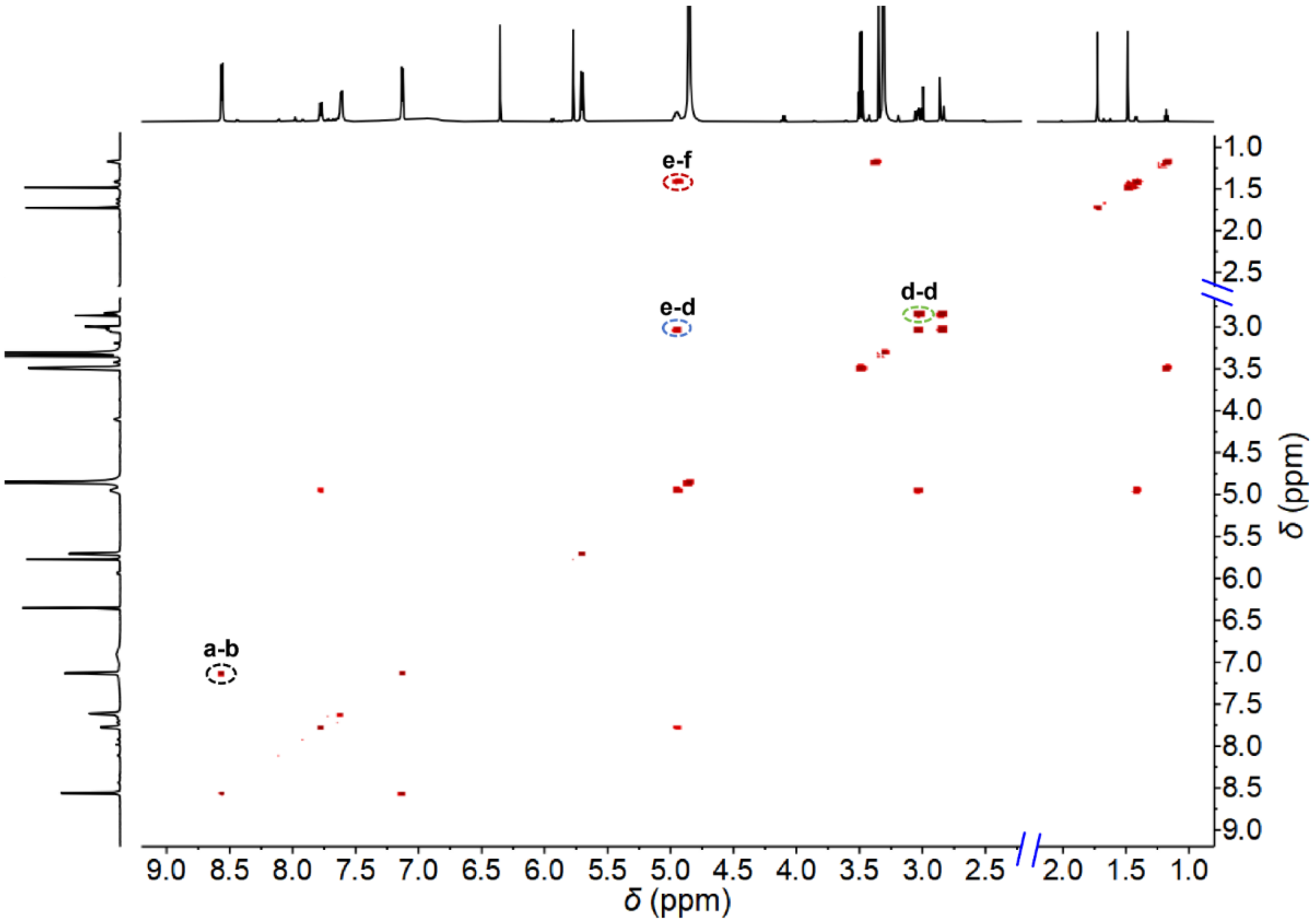

Fig. S97. ^{1}H-^{1}H COSY spectrum of Δ-**Ir-1** in CD_3OD (600 MHz, c = 1.4 mM, with respect to trefoil knots).

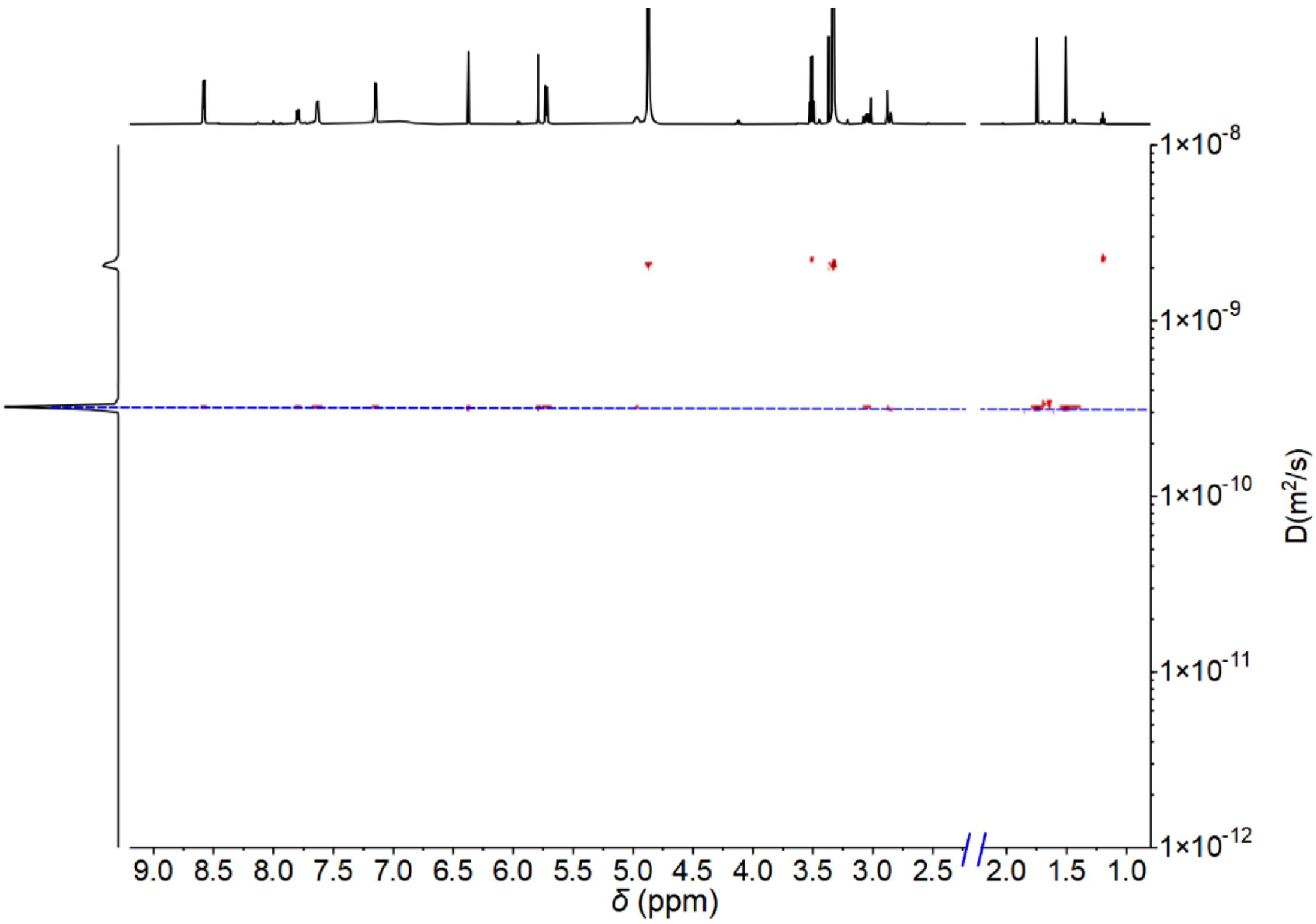

Fig. S98. ^{1}H DOSY spectrum of Δ-**Ir-1** in CD_3OD (600 MHz, c = 1.4 mM, where $c = c_{Ir}/6$). The assigned trefoil-knot resonances exhibit a diffusion coefficient of $D = 3.20 \times 10^{-10}$ m^2 s^{-1}.

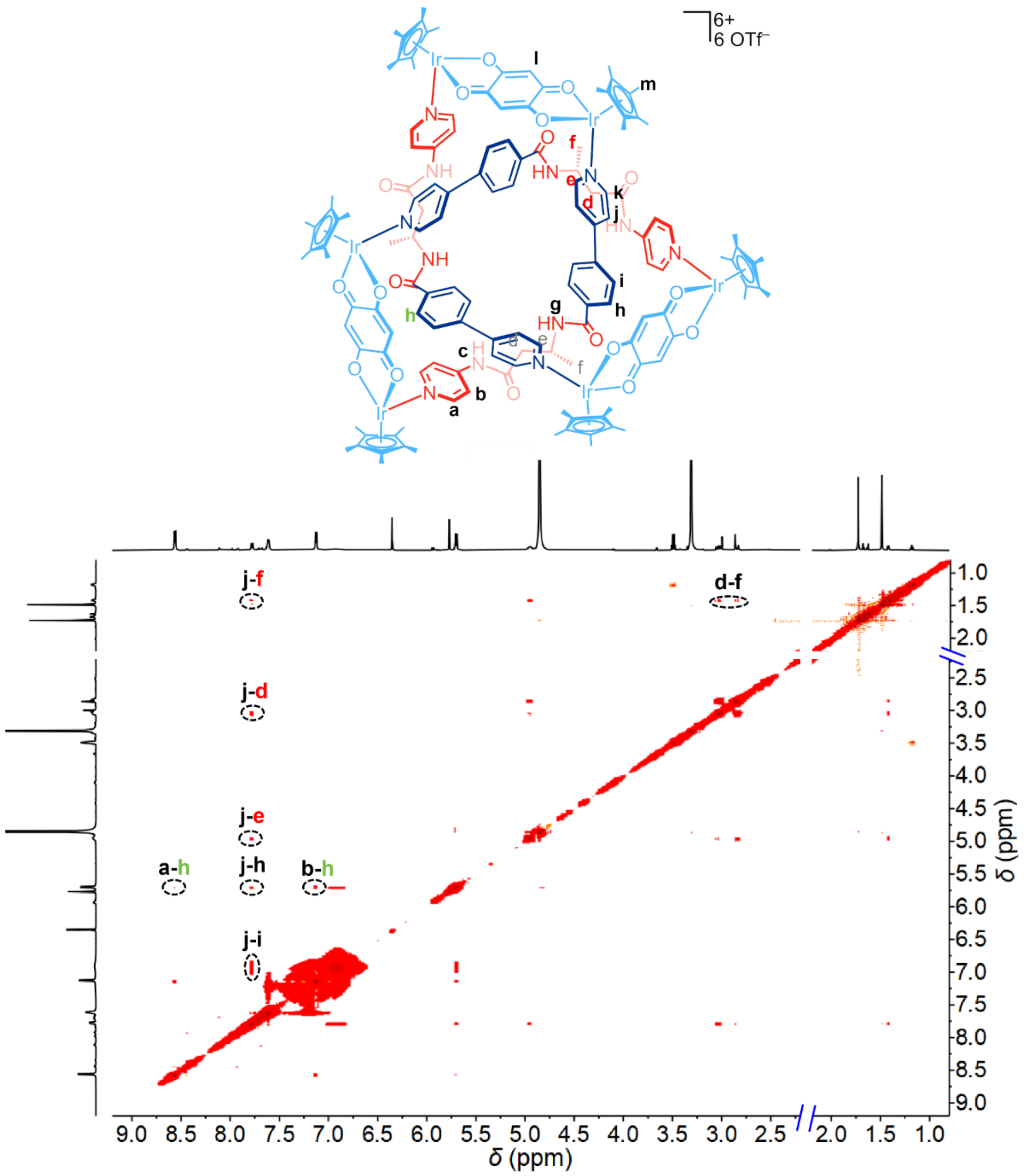

Fig. S99. ^{1}H-^{1}H NOESY NMR (600 MHz, CD_3OD, c = 2.8 mM) for Δ-**Ir-1**.

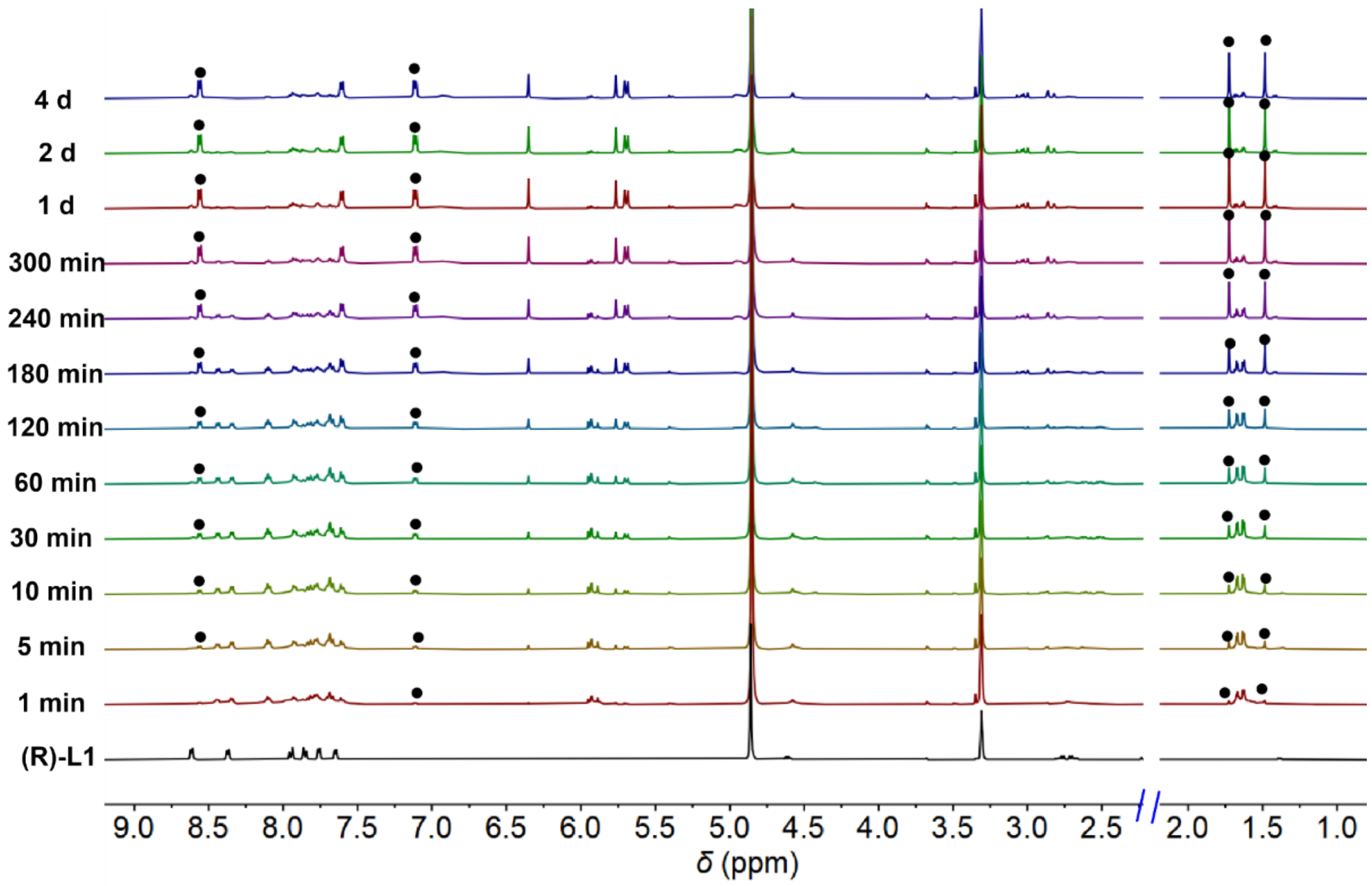

Fig. S100. Time-dependent ^{1}H NMR spectra monitoring self-assembly of (R)-**L1** with **B3**(OTf)$_2$ to form Δ-**Ir-1** (400 MHz, CD_3OD, room temperature; formal knot concentration = 6.0 mM). Formation is largely complete after approximately 1 d. Selected resonances corresponding to the trefoil-knot assemblies are highlighted with black circles.

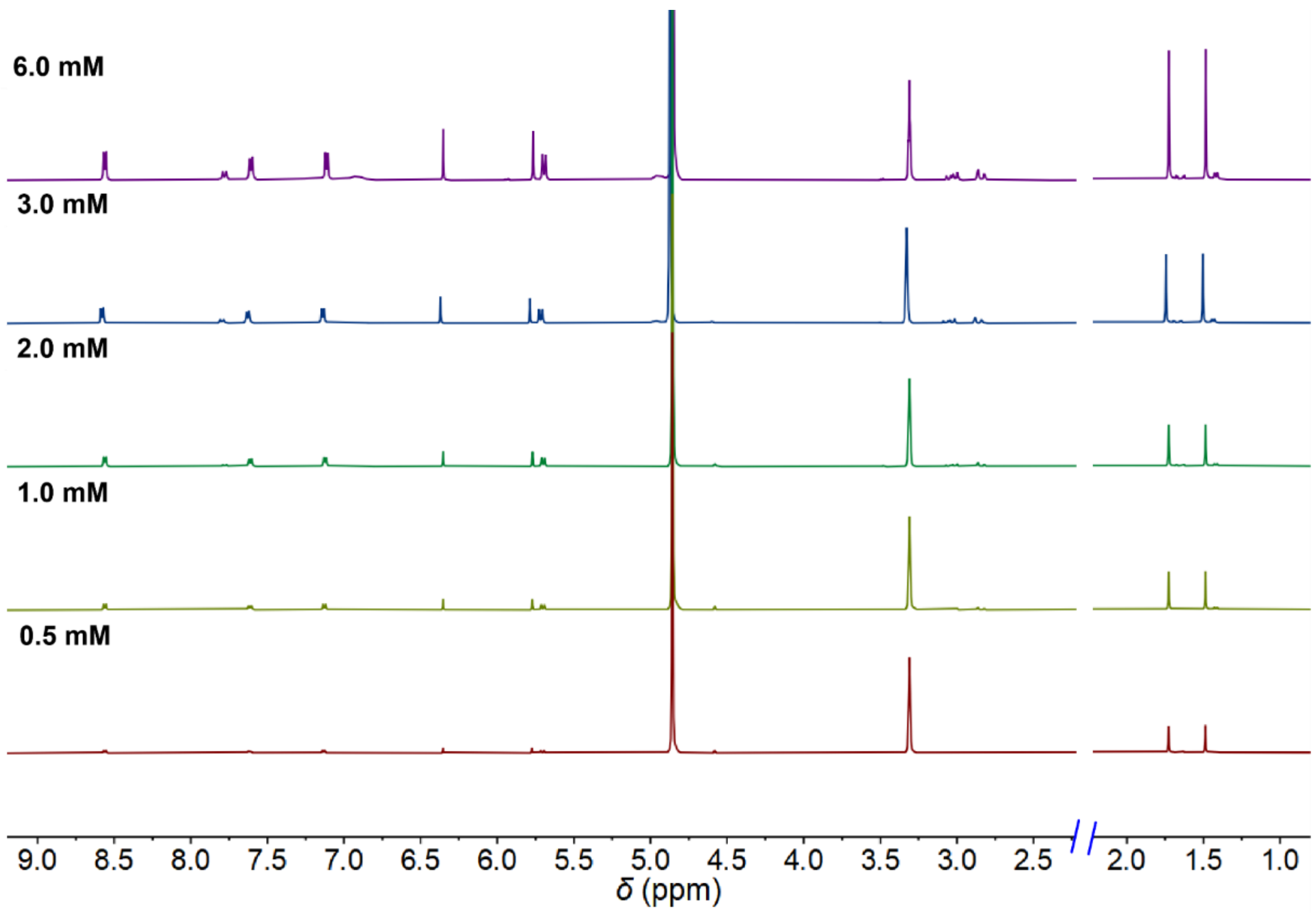

Fig. S101. Concentration-dependent ^{1}H NMR spectra of Δ-**Ir-1** in CD_3OD (400 MHz, c = 0.5–6.0 mM). The spectra show no substantial changes in the positions or relative intensities of the assigned assembly resonances over the concentration range examined.

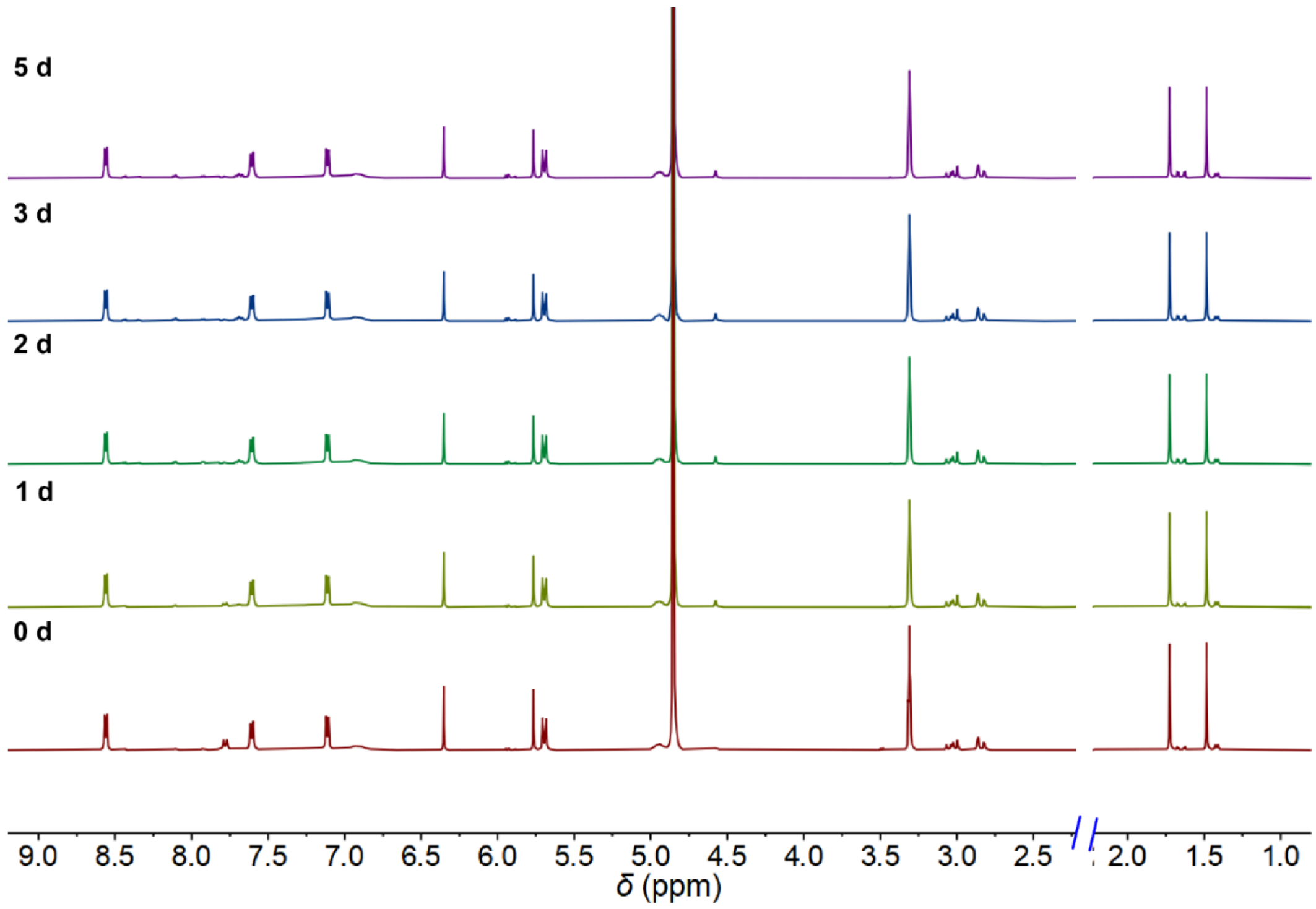

Fig. S102. ^{1}H NMR spectra of Δ-**Ir-1** recorded over 0–5 d in CD_3OD at room temperature (400 MHz, c = 6.0 mM). The spectra remain essentially unchanged over this period.

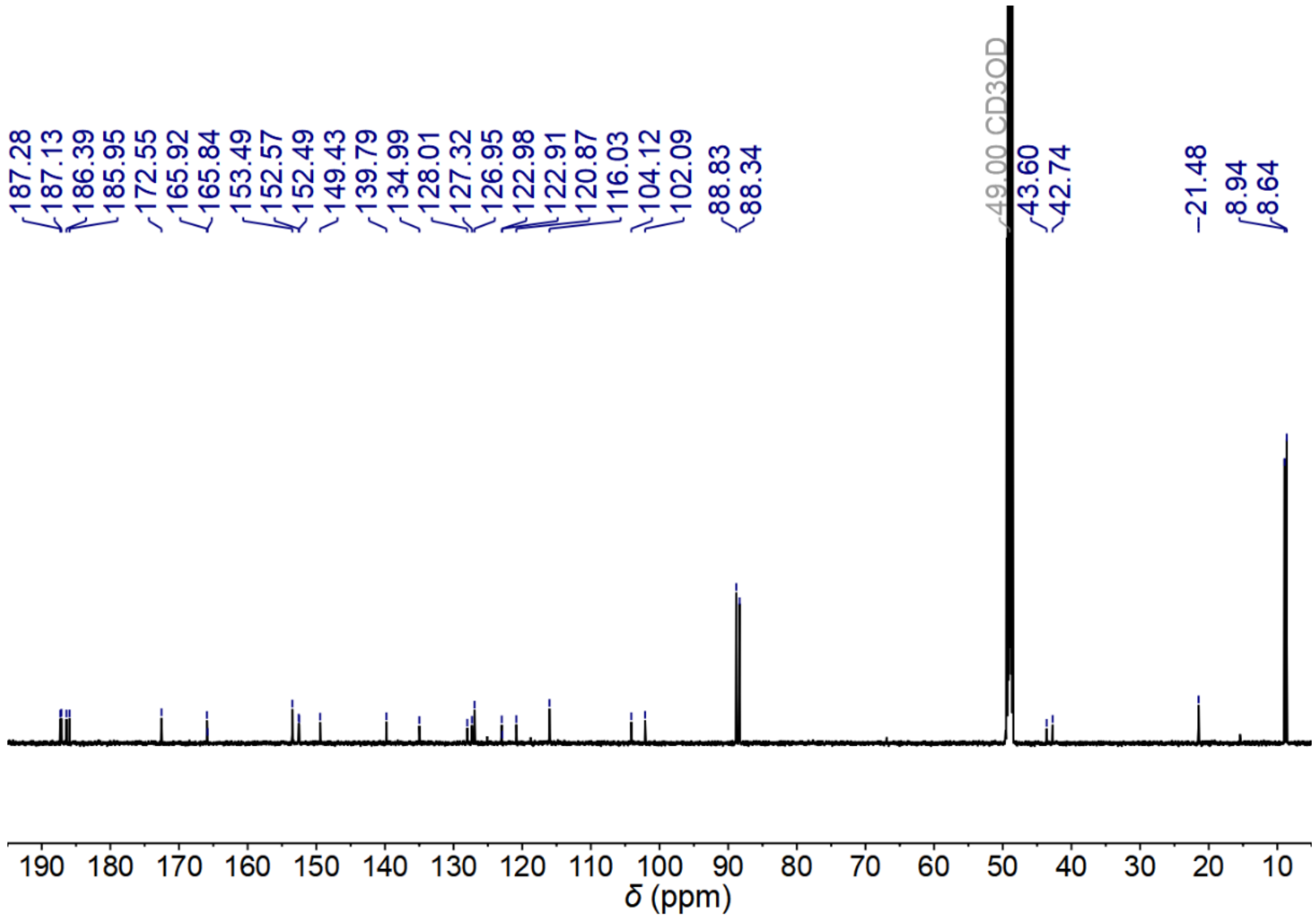

Fig. S103. $^{13}C\{^1H\}$ NMR spectrum of Δ-**Ir-1** in CD_3OD (151 MHz, *c* = 6.0 mM)

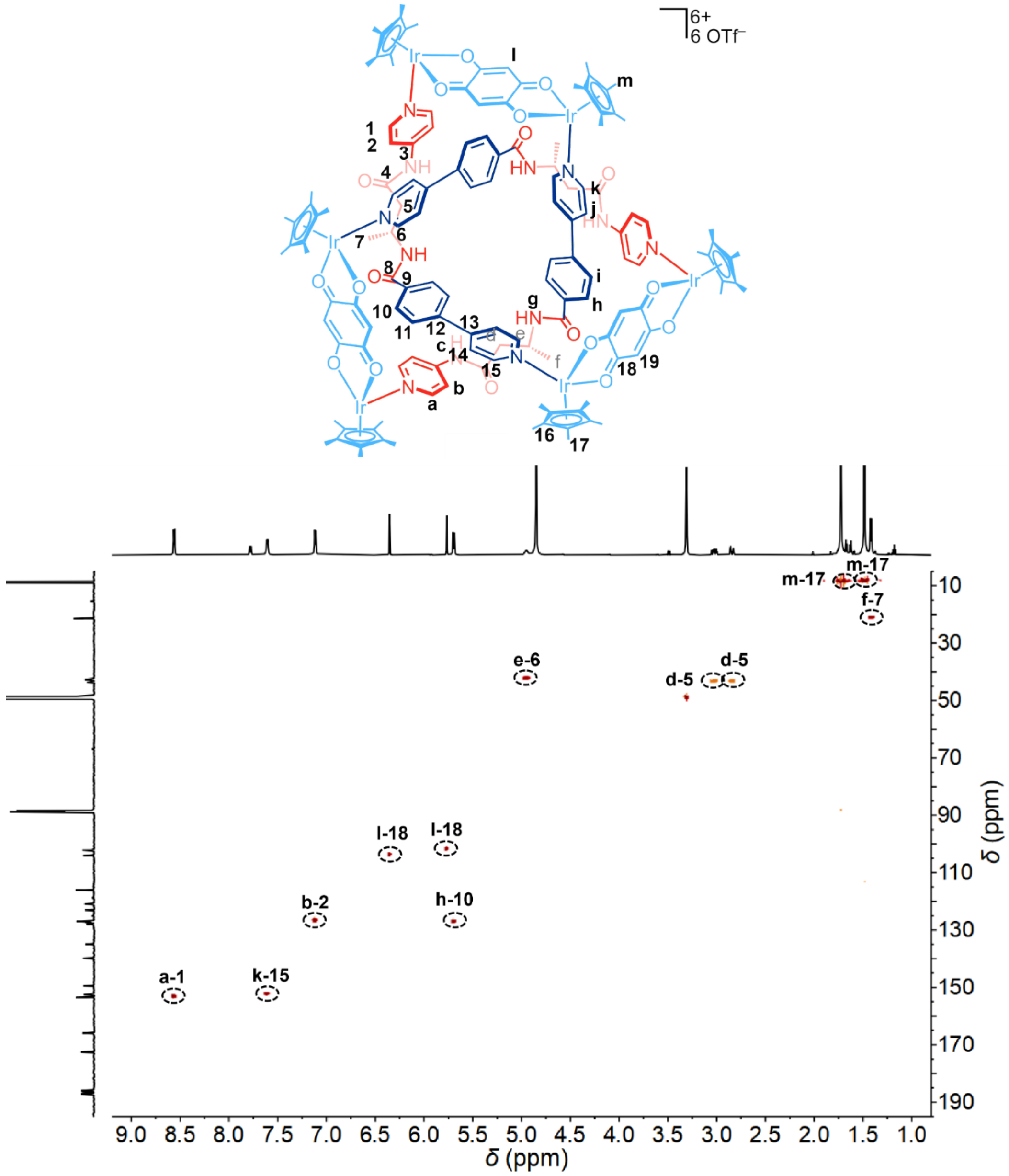

Fig. S104. ¹H–¹³C HSQC spectrum of Δ-**Ir-1** in CD_3OD (600 MHz, c = 6.0 mM).

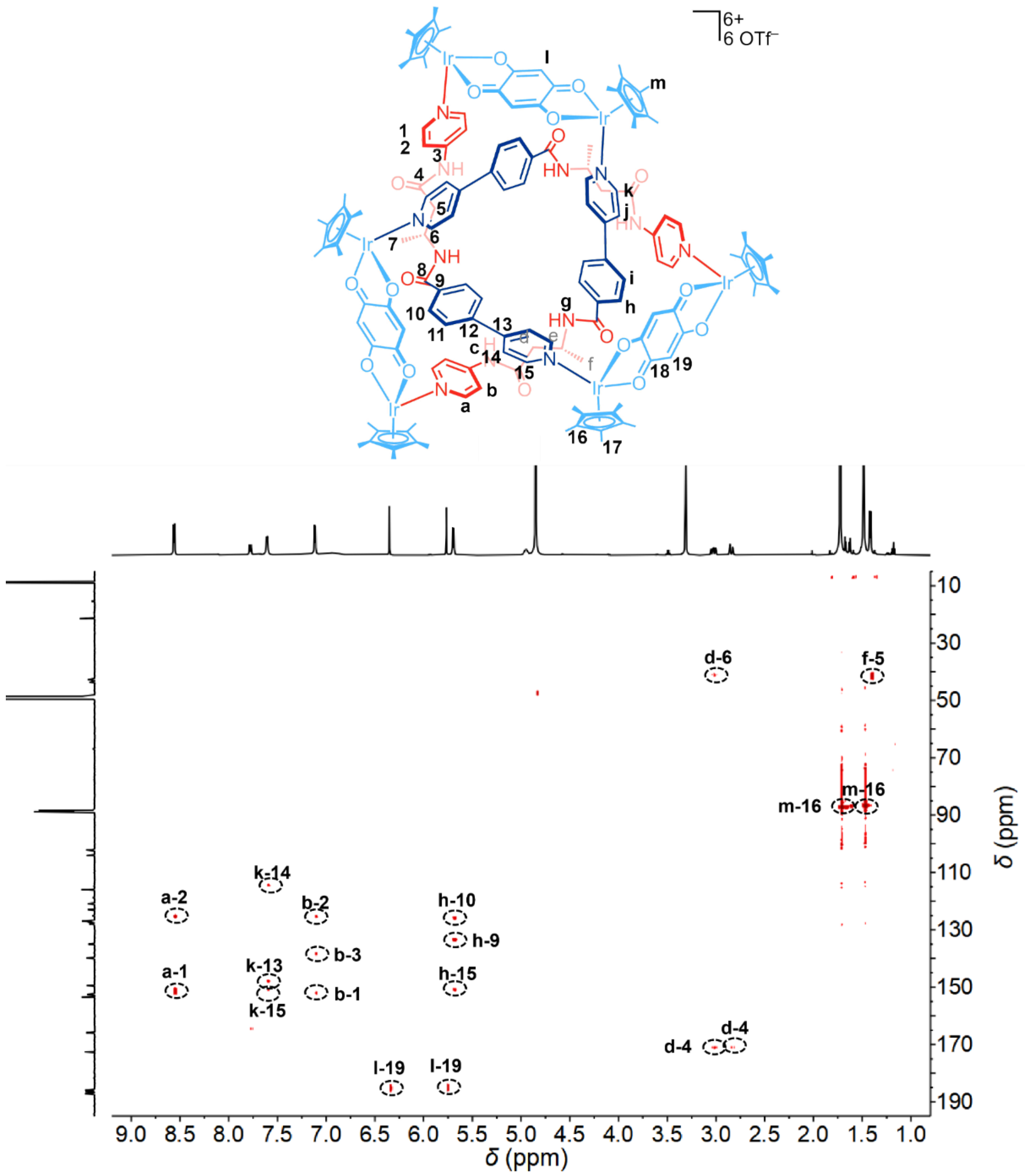

Fig. S105. ^1H–^{13}C HMBC spectrum of Δ-**Ir-1** in CD_3OD (600 MHz, c = 6.0 mM).

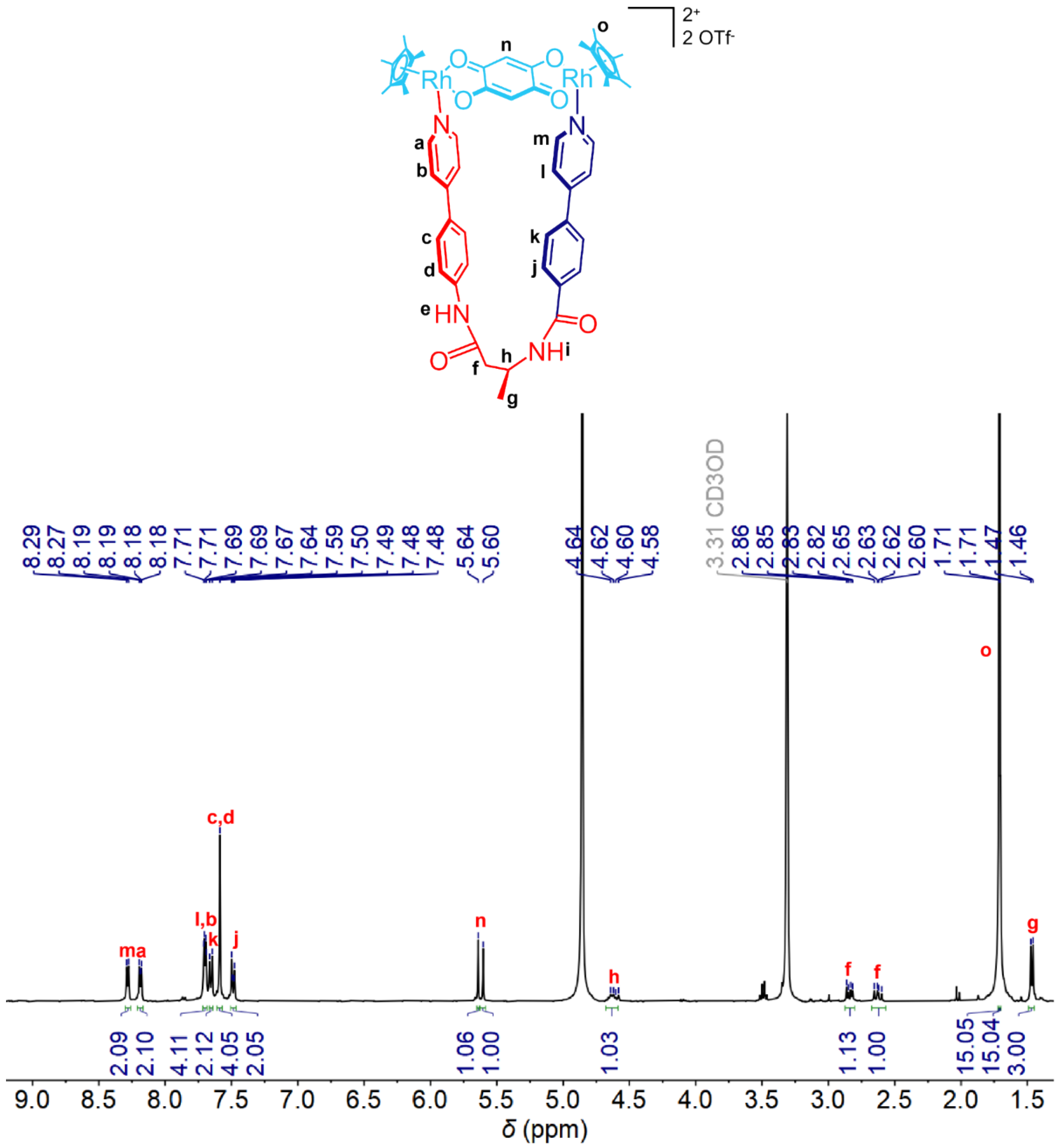

Fig. S106. ^{1}H NMR spectrum of a solution prepared by dissolving crystals of **Rh-2*S*** in CD_3OD (600 MHz, c_{Rh} = 36.0 mM). The hydrogen protons of NH on the ligand lose their NMR signal due to the exchange with D in CD_3OD. The NMR data, together with DOSY and ESI-MS, support retention of the crystallographically characterized D-shaped metallacycle in solution.

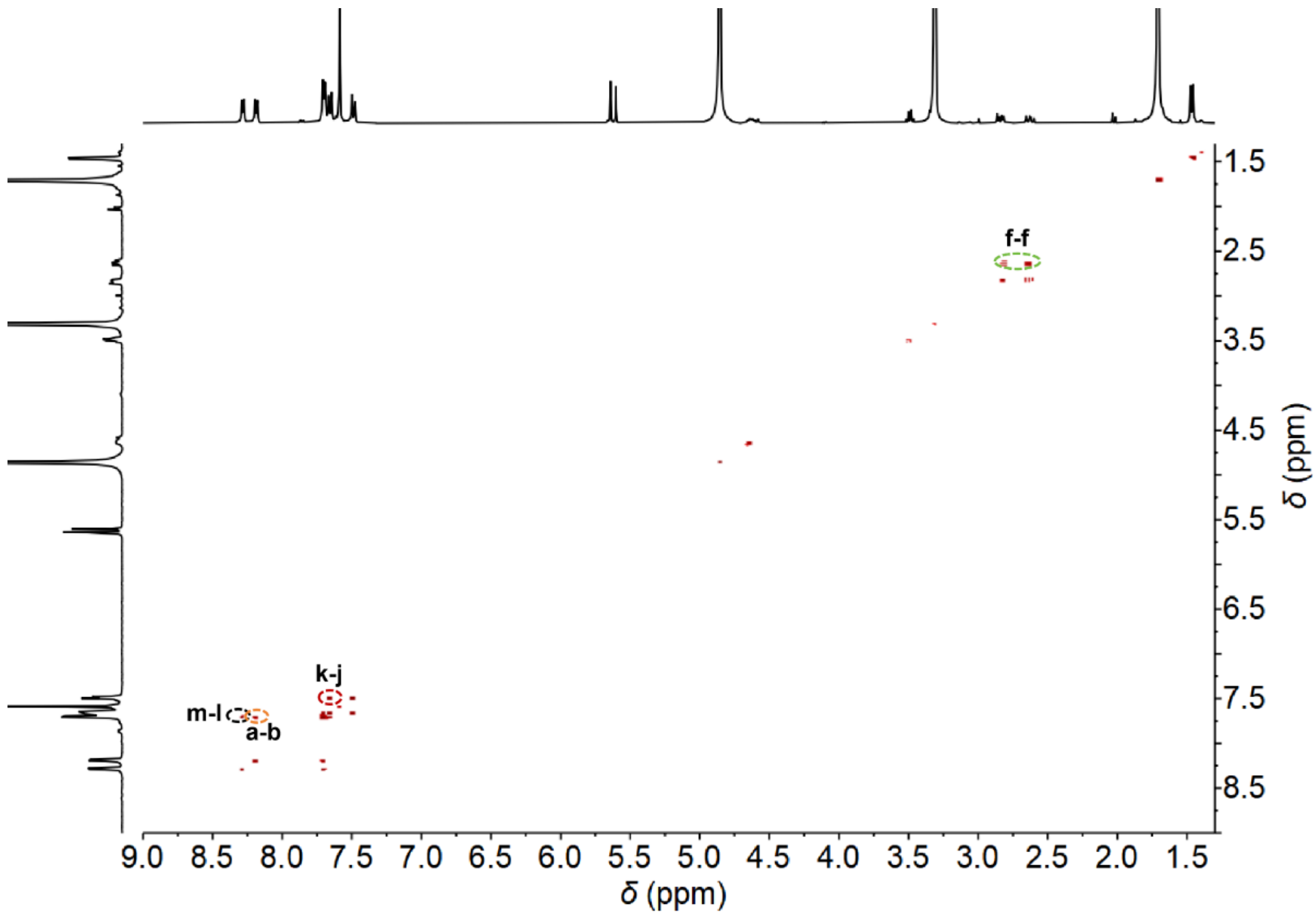

Fig. S107. ^{1}H-^{1}H COSY spectrum of a solution prepared by dissolving crystals of **Rh-2*S*** in CD_3OD (600 MHz, c_{Rh} = 36.0 mM).

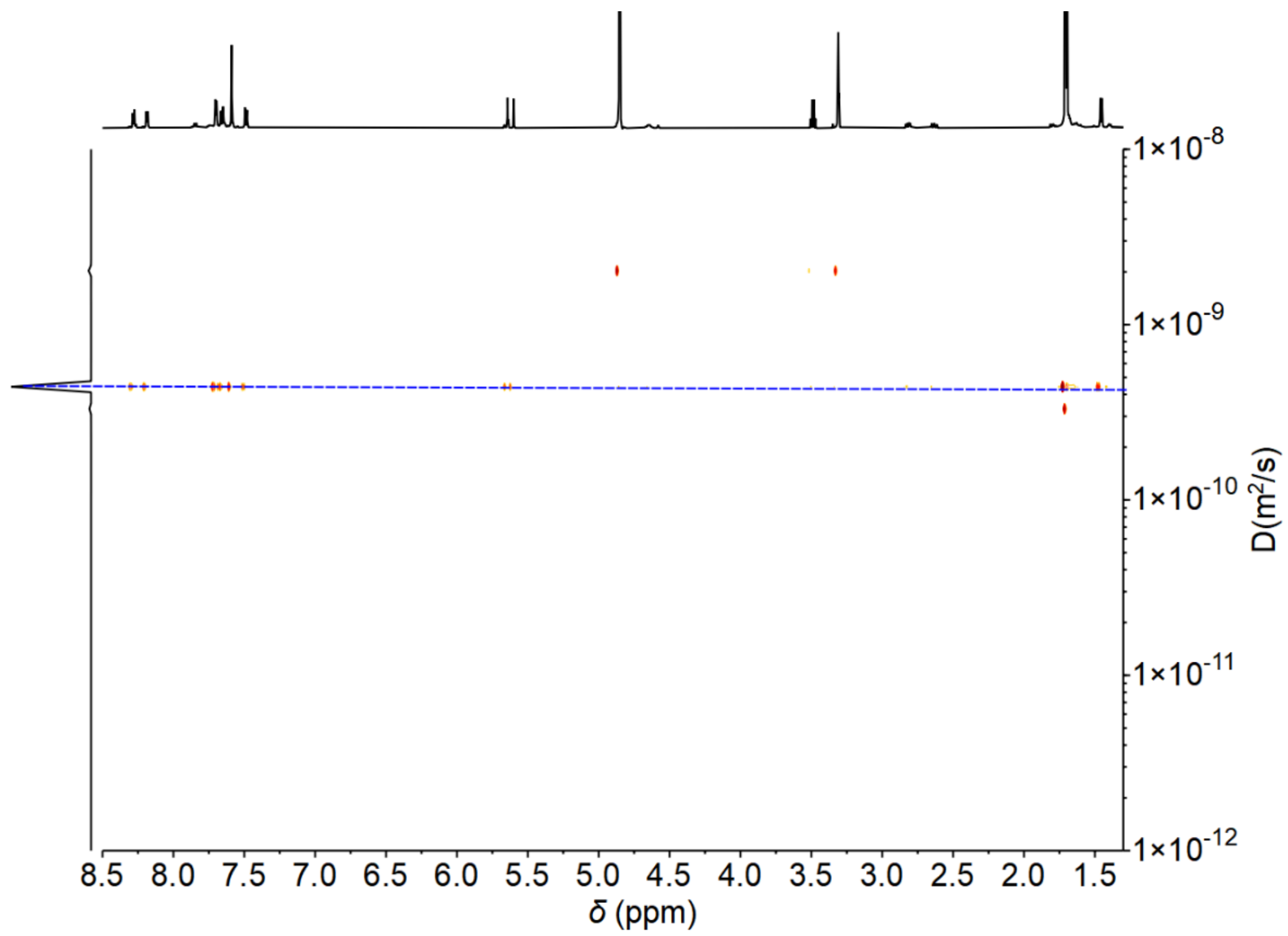

Fig. S108. ^{1}H DOSY spectrum of a solution prepared by dissolving crystals of **Rh-2*S*** in CD_3OD (600 MHz, c_{Rh} = 36.0 mM). The assigned D-shaped metallacycle resonances exhibit a diffusion coefficient of D = 4.40 × 10^{-10} m^2 s^{-1}.

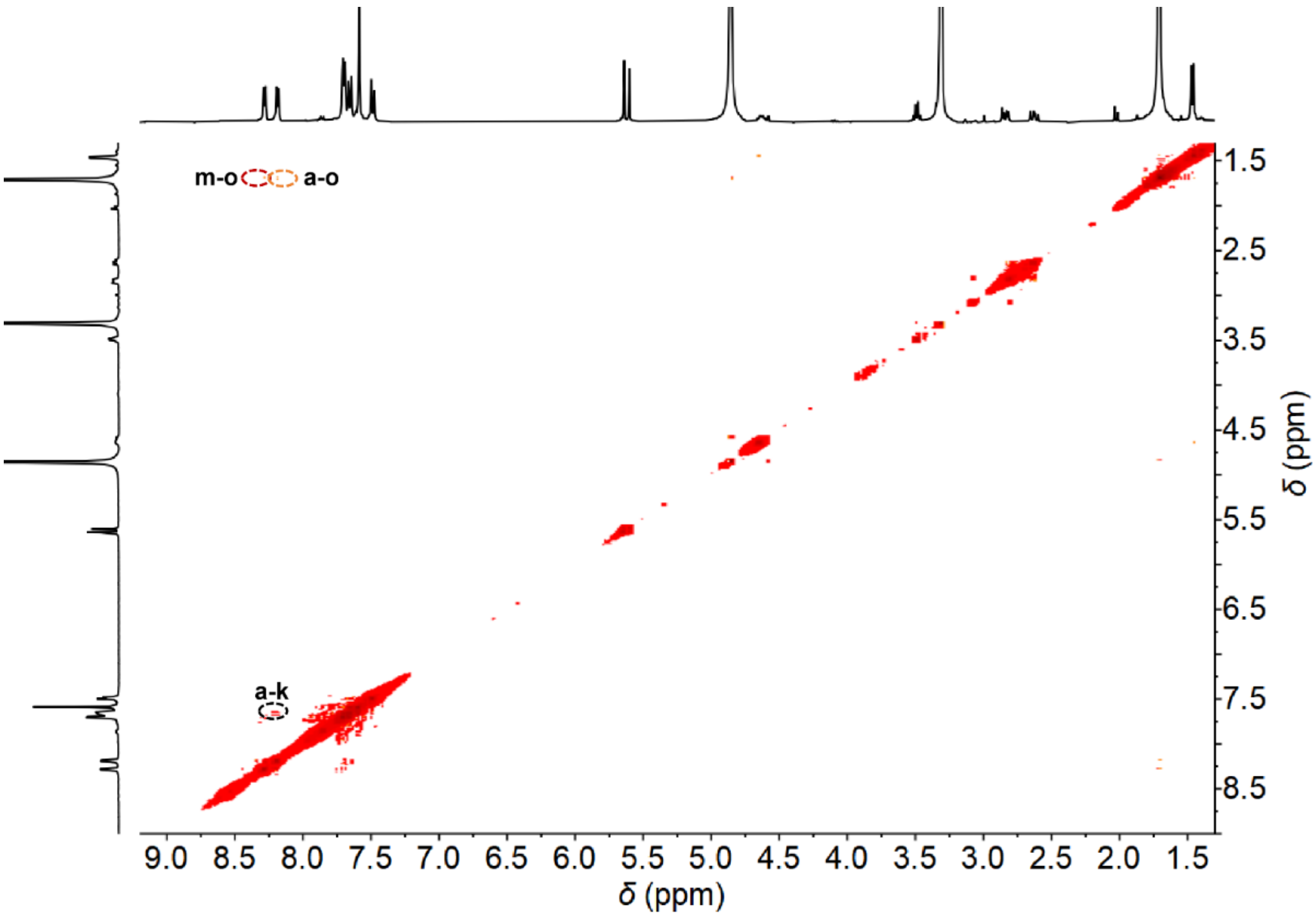

Fig. S109. ^{1}H-^{1}H NOESY spectrum of a solution prepared by dissolving crystals of **Rh-2*S*** in CD_3OD (600 MHz, c_{Rh} = 36.0 mM).

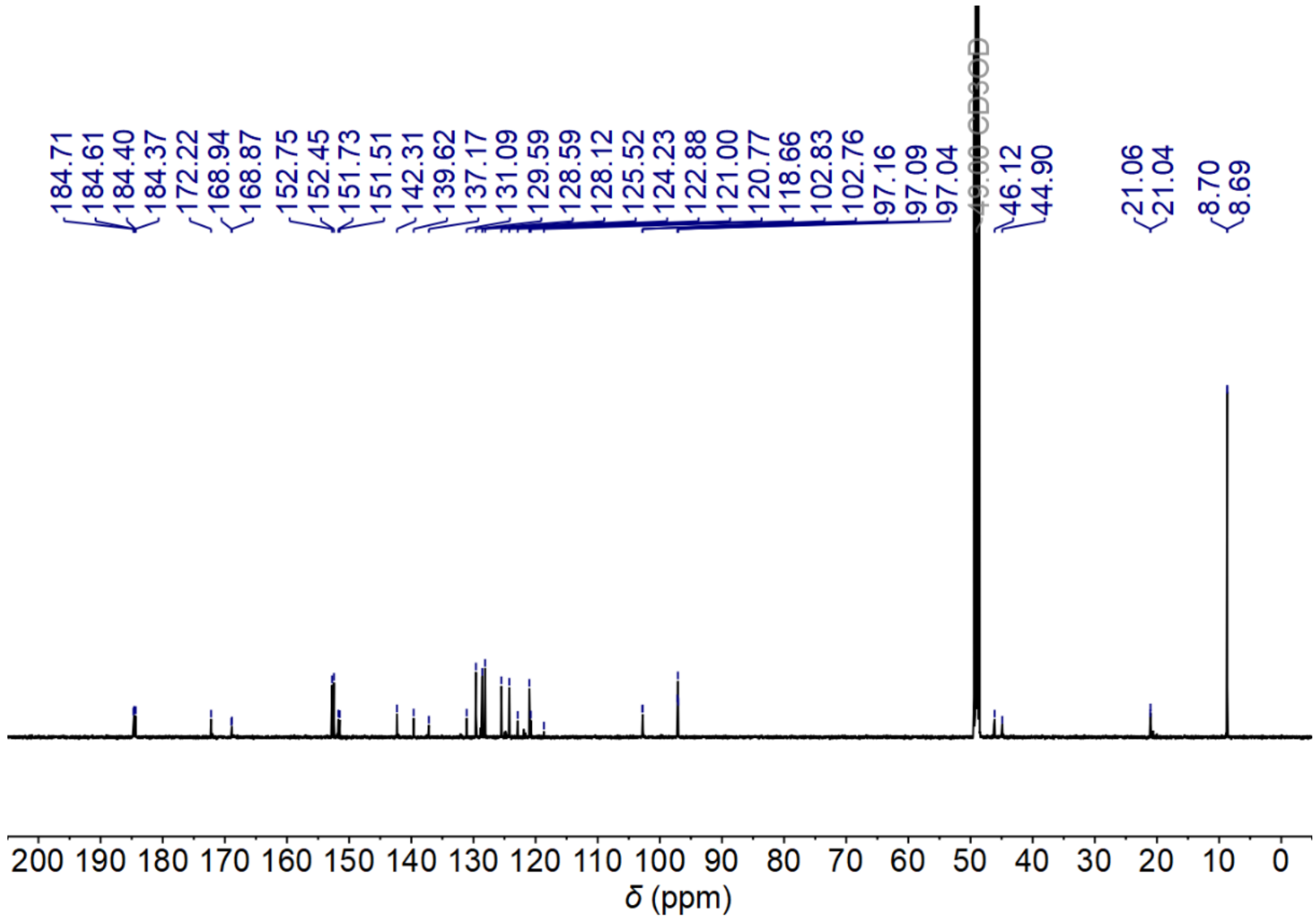

Fig. S110. $^{13}C\{^{1}H\}$ NMR spectrum of a solution prepared by dissolving crystals of **Rh-2*S*** in CD_3OD (151 MHz, c_{Rh} = 36.0 mM).

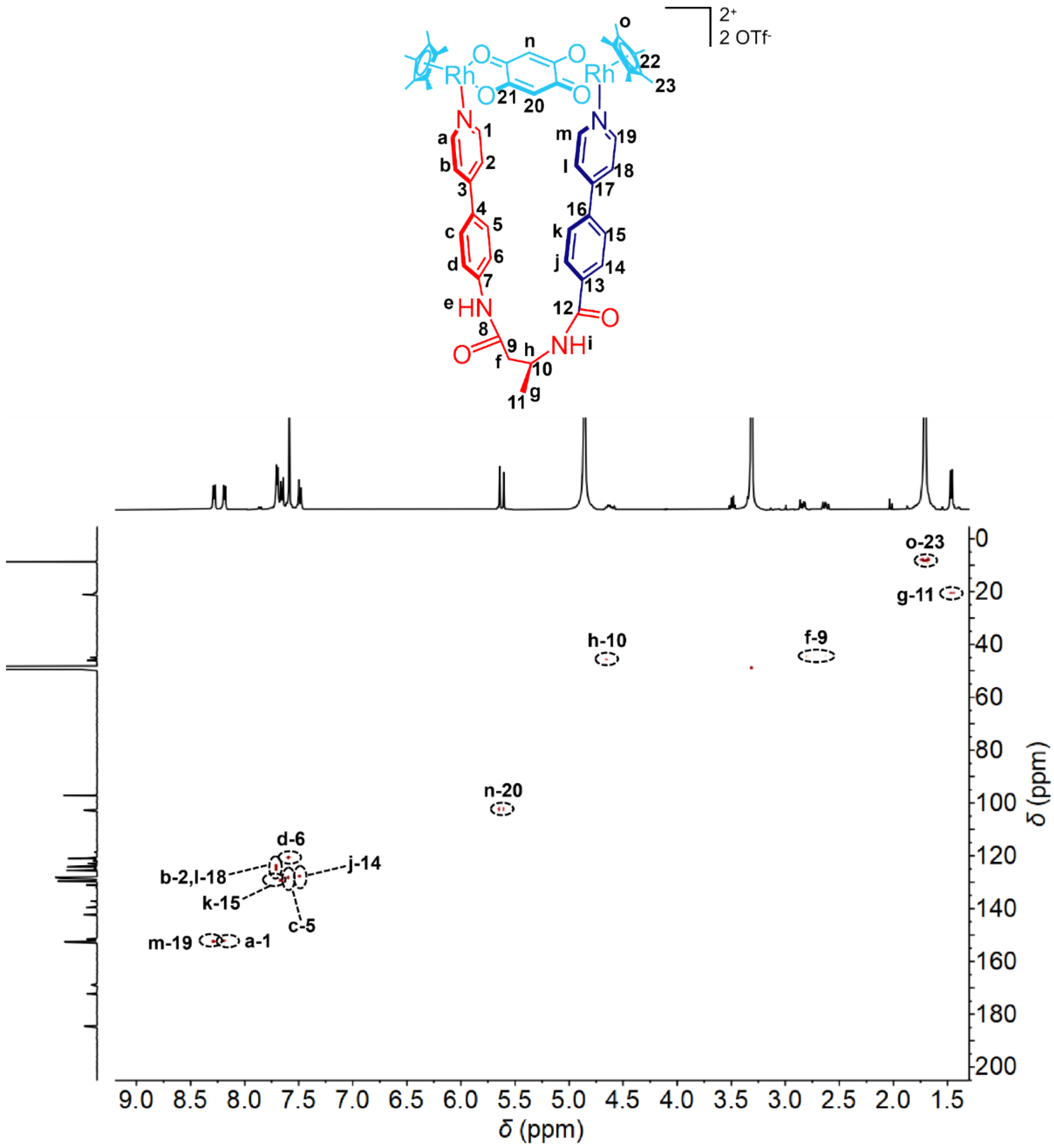

Fig. S111. ^{1}H–^{13}C HSQC spectrum of a solution prepared by dissolving crystals of **Rh-2*S*** in CD_3OD (600 MHz, c_{Rh} = 36.0 mM).

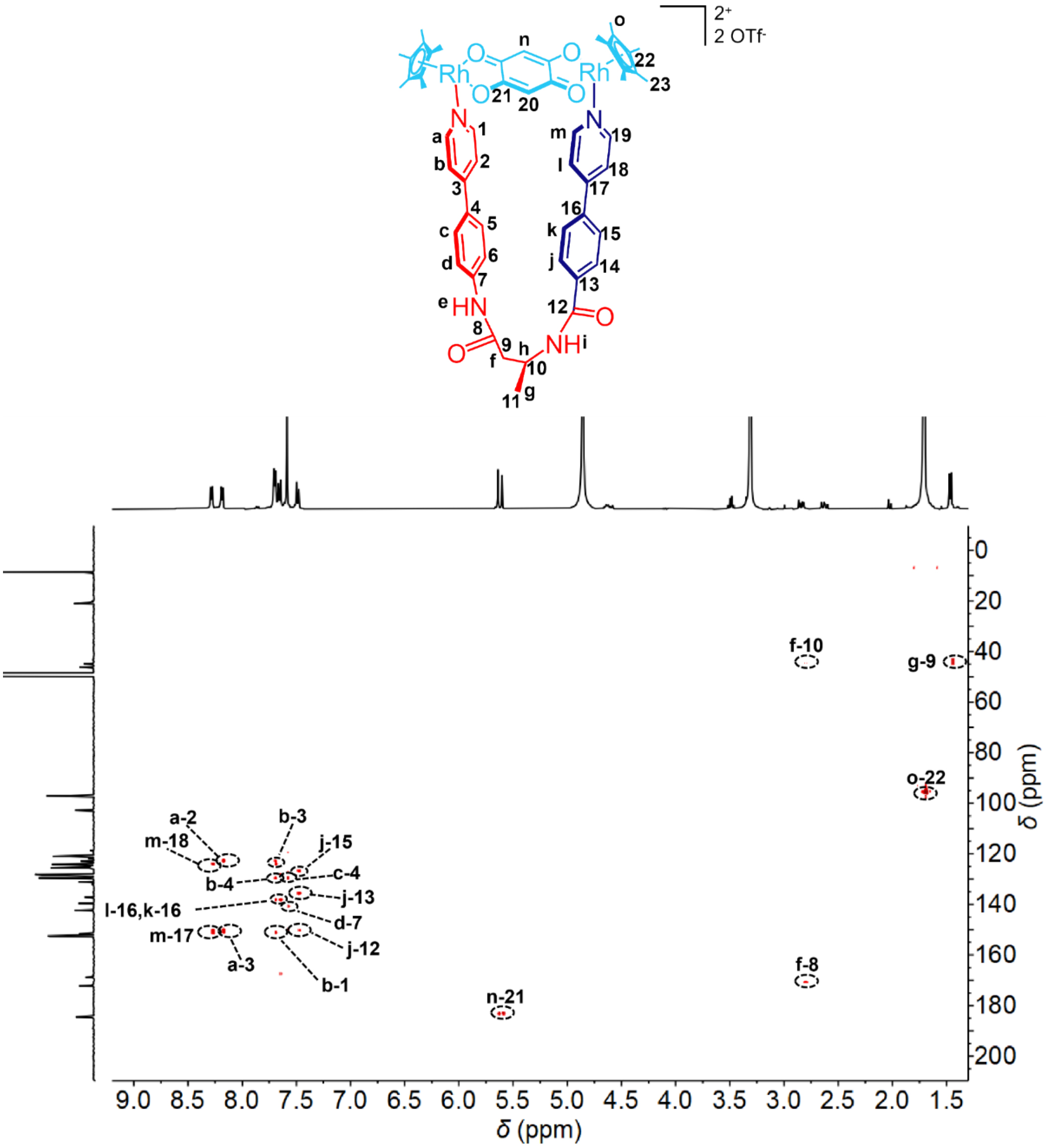

Fig. S112. ^{1}H–^{13}C HMBC spectrum of a solution prepared by dissolving crystals of **Rh-2*S*** in CD_3OD (600 MHz, c_{Rh} = 36.0 mM).

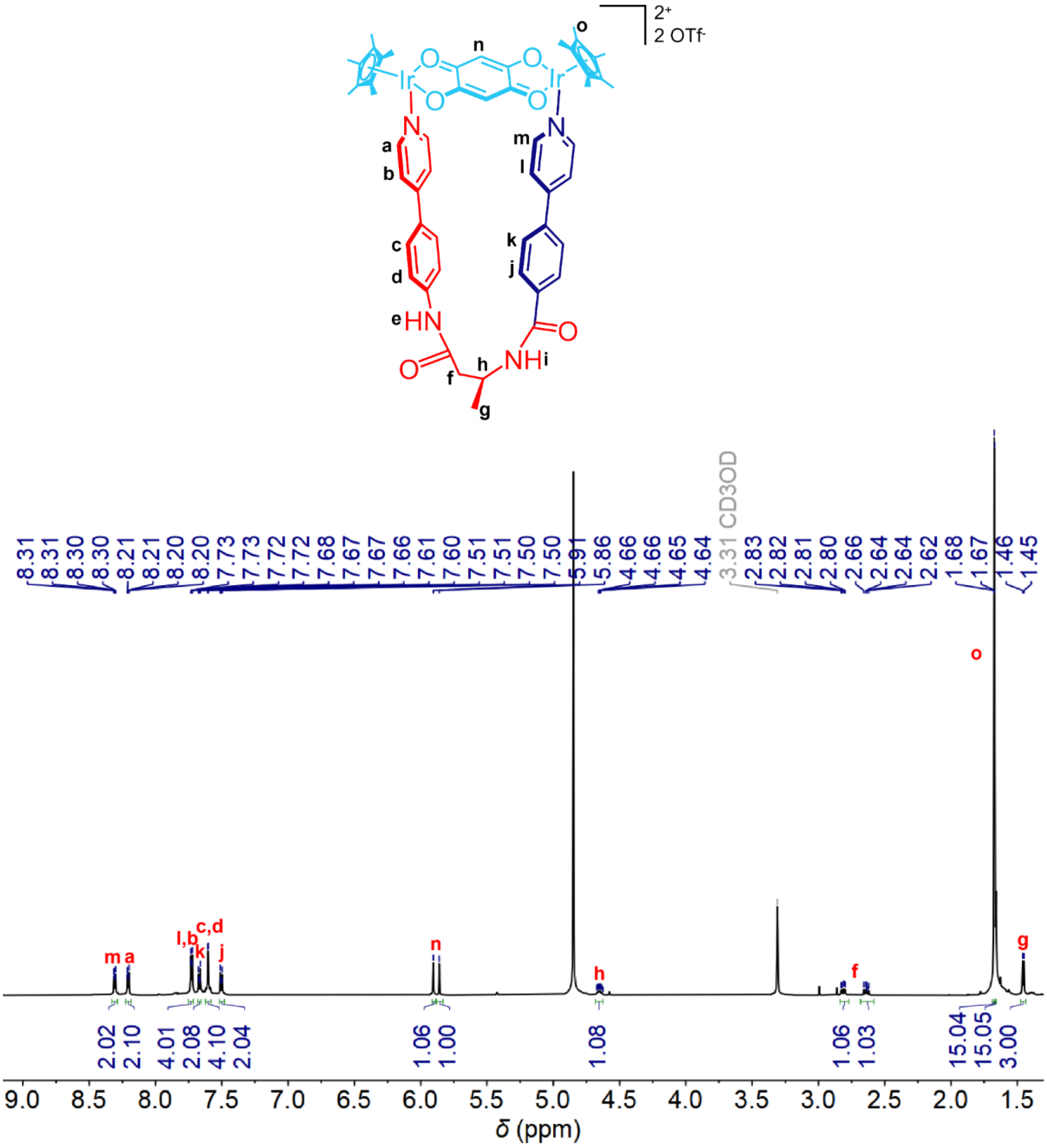

Fig. S113. ^{1}H NMR spectrum of a solution prepared by dissolving crystals of **Ir-2***S* in CD_3OD (600 MHz, c_{Ir} = 36.0 mM). The hydrogen protons of NH on the ligand lose their NMR signal due to the exchange with D in CD_3OD. The NMR data, together with DOSY and ESI-MS, support retention of the crystallographically characterized D-shaped metallacycle in solution.

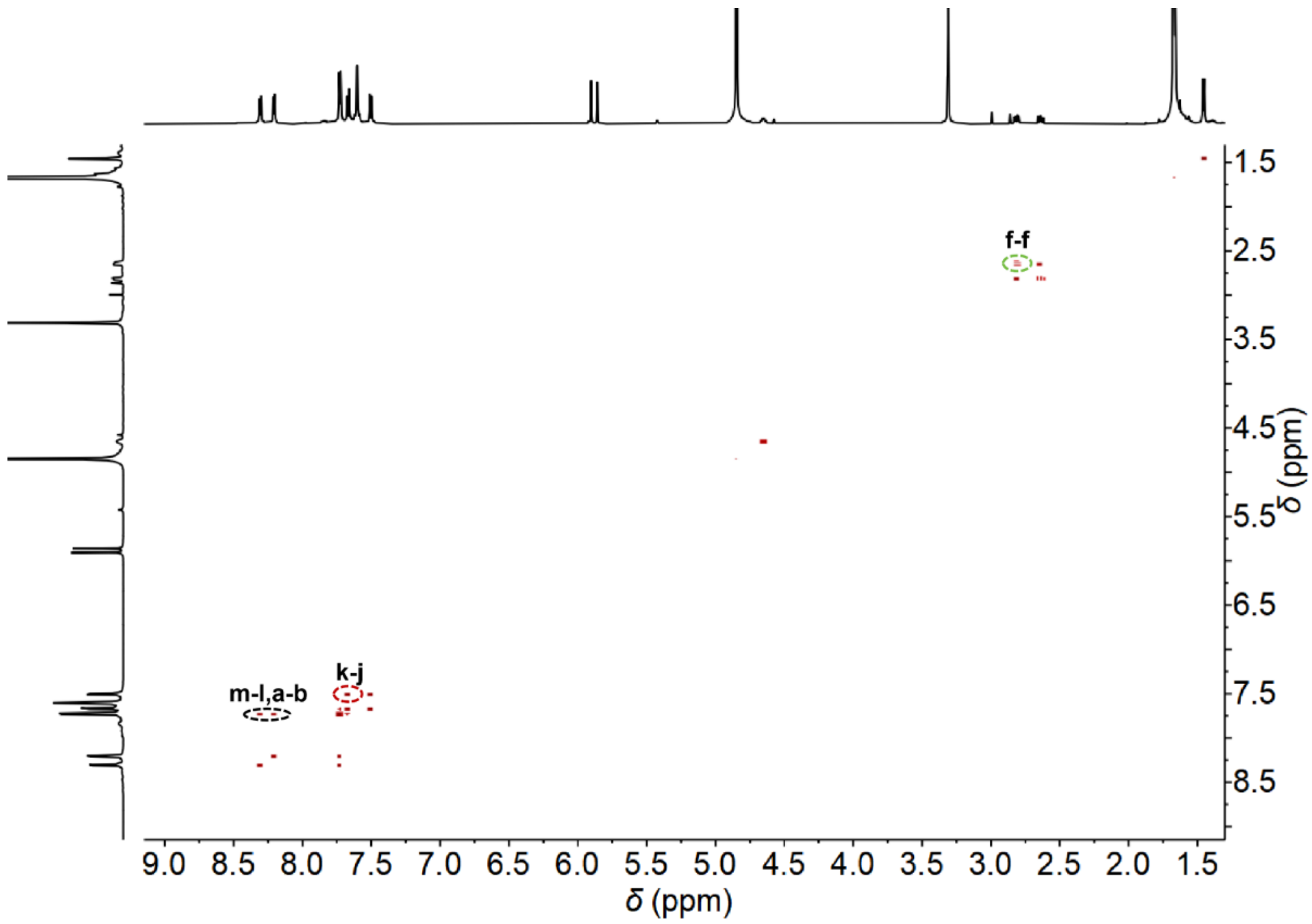

Fig. S114. ^{1}H-^{1}H COSY spectrum of a solution prepared by dissolving crystals of **Ir-2*S*** in CD_3OD (600 MHz, c_{Ir} = 36.0 mM).

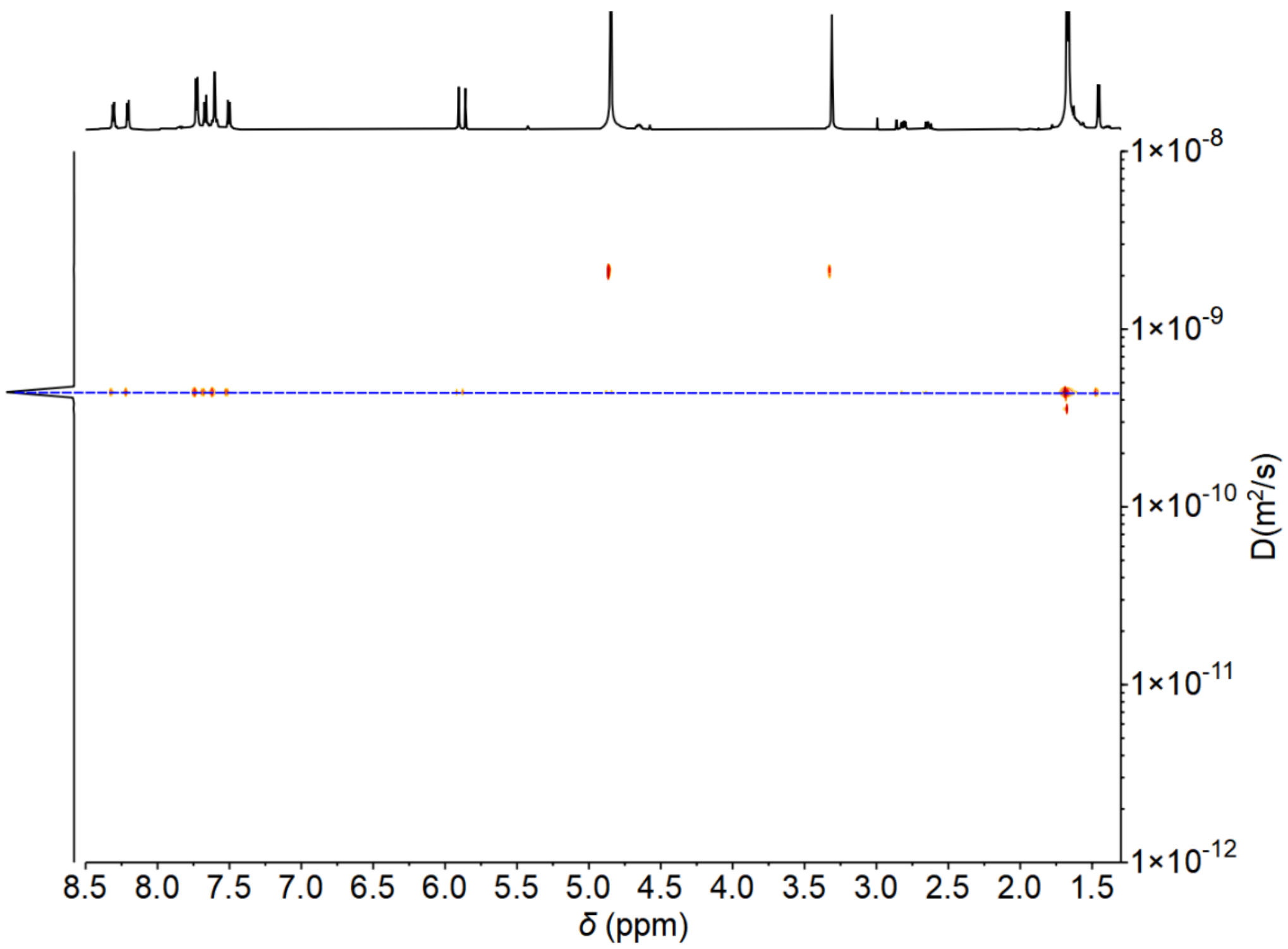

Fig. S115. ¹H DOSY spectrum of a solution prepared by dissolving crystals of **Ir-2*S*** in CD_3OD (600 MHz, c_{Ir} = 36.0 mM). The assigned D-shaped metallacycle resonances exhibit a diffusion coefficient of $D = 4.41 \times 10^{-10}$ m² s⁻¹.

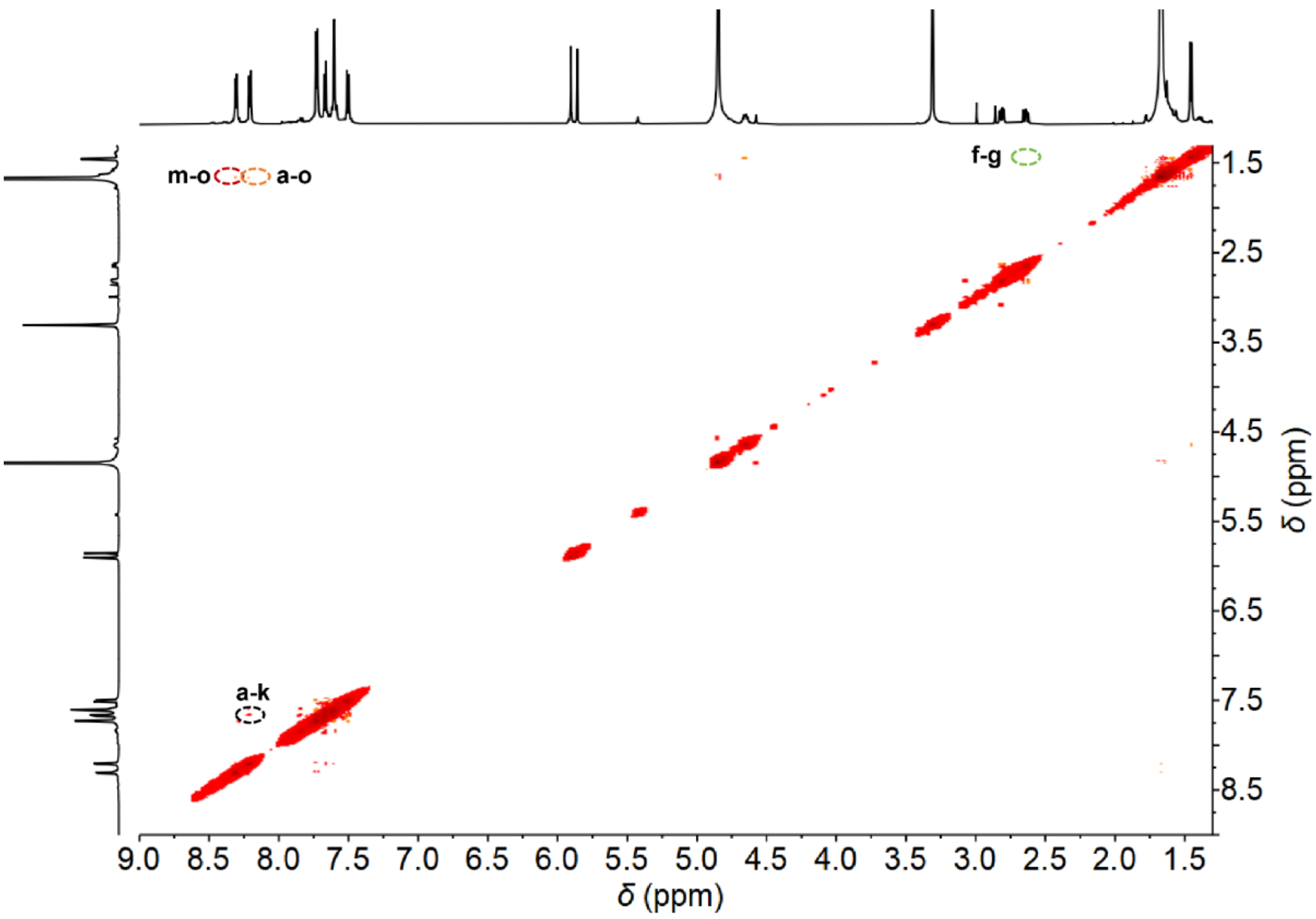

Fig. S116. ¹H-¹H NOESY spectrum of a solution prepared by dissolving crystals of **Ir-2*S*** in CD_3OD (600 MHz, c_{Ir} = 36.0 mM).

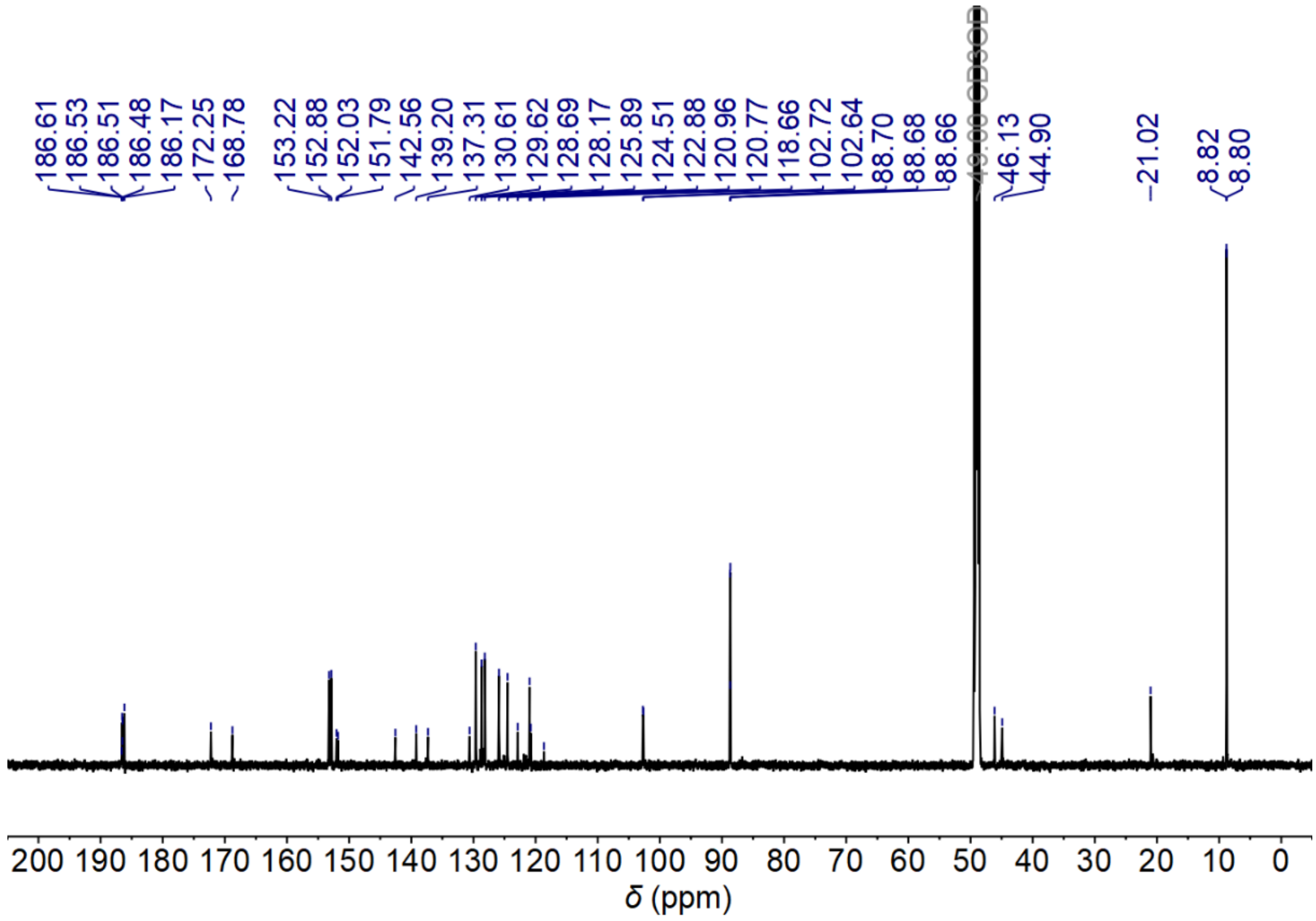

Fig. S117. $^{13}C\{^{1}H\}$ NMR spectrum of a solution prepared by dissolving crystals of **Ir-2***S* in CD_3OD (151 MHz, c_{Ir} = 36.0 mM).

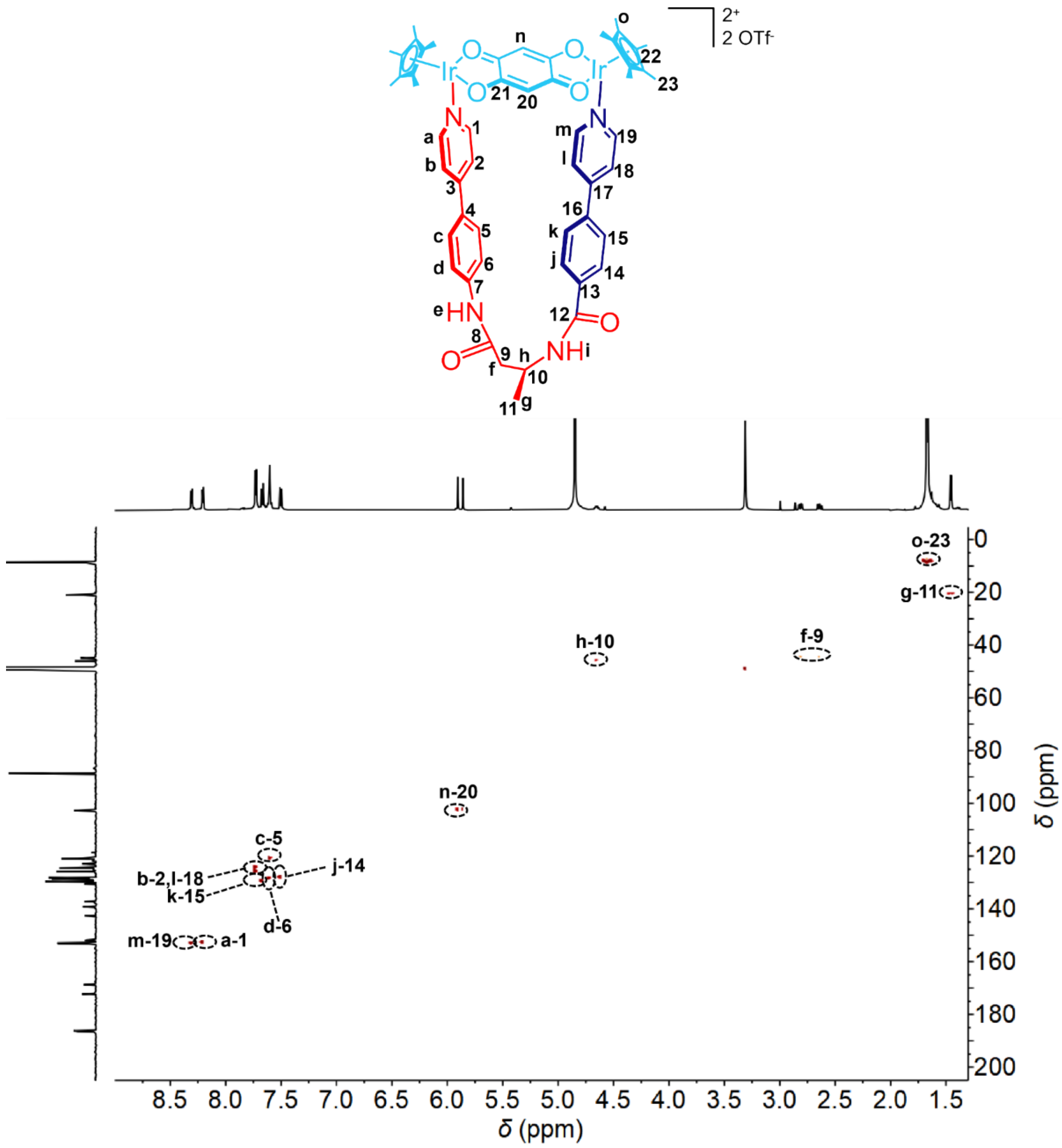

Fig. S118. ^{1}H–^{13}C HSQC spectrum of a solution prepared by dissolving crystals of **Ir-2*S*** in CD_3OD (600 MHz, c_{Ir} = 36.0 mM).

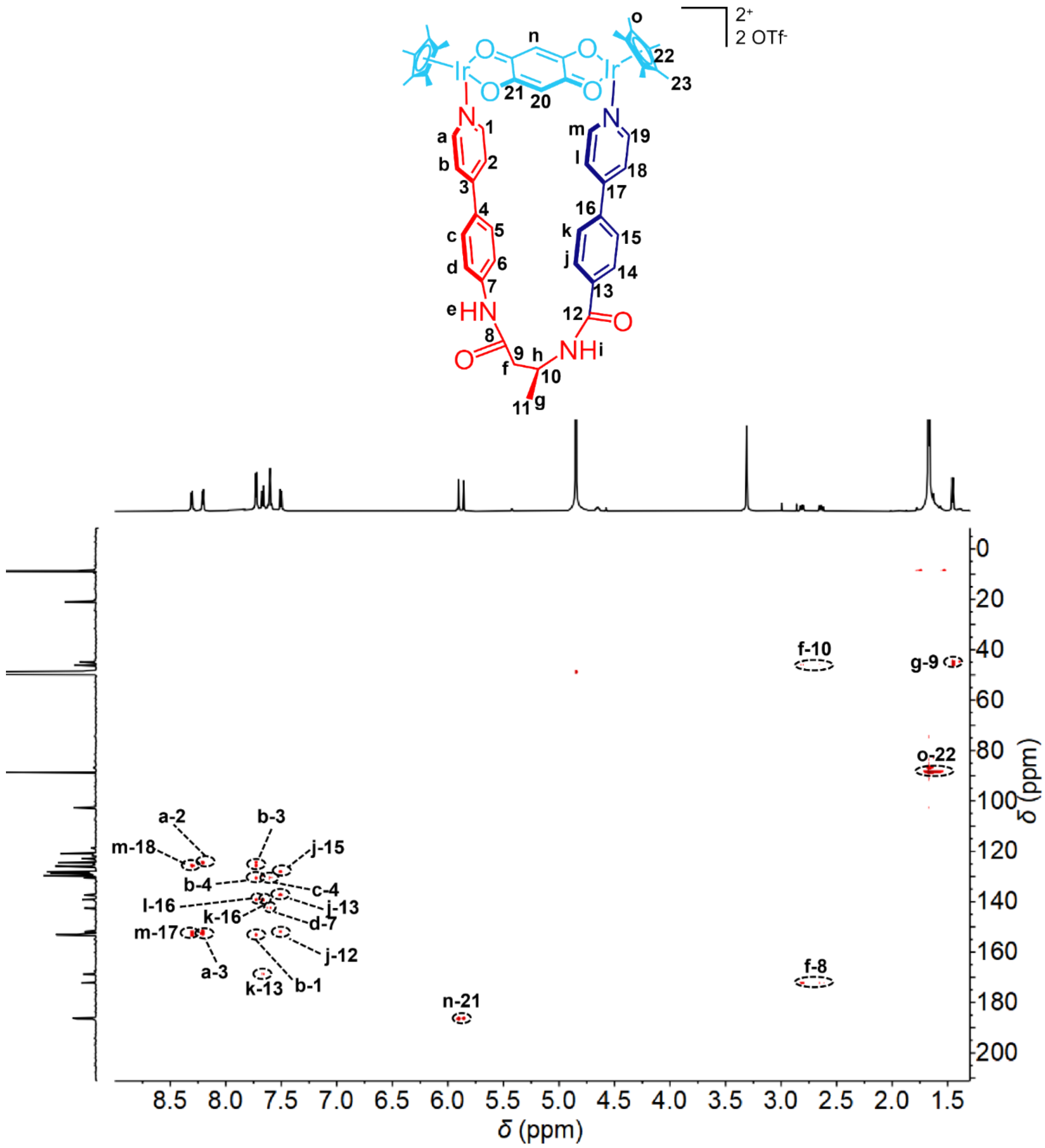

Fig. S119. ^{1}H–^{13}C HMBC spectrum of a solution prepared by dissolving crystals of **Ir-2*S*** in CD_3OD (600 MHz, c_{Ir} = 36.0 mM).

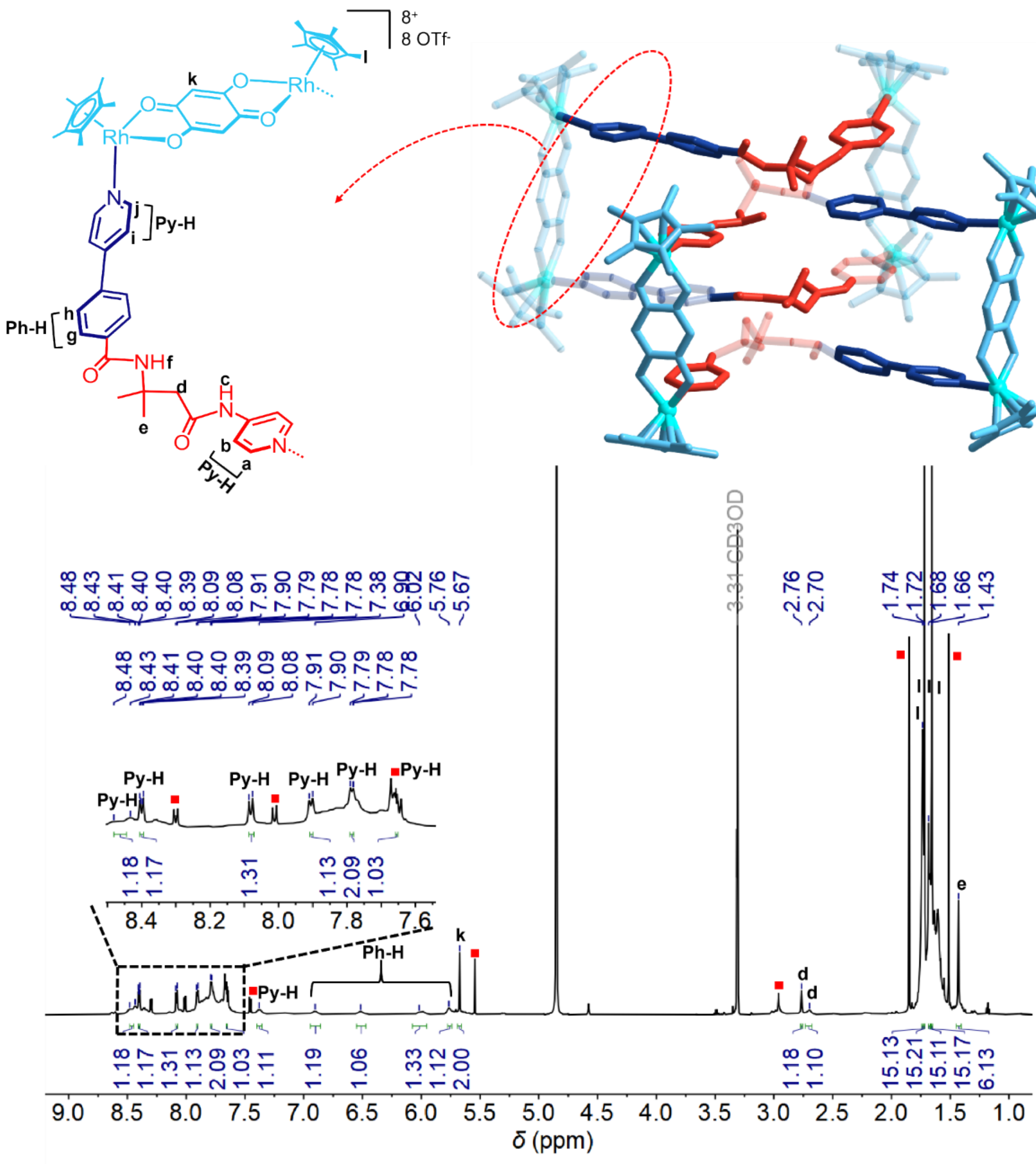

Fig. S120. ^{1}H NMR spectrum of a solution prepared by dissolving crystals of **Rh-3** in CD_3OD (600 MHz, c_{Rh} = 36.0 mM). Resonances assigned to the nested dimer **Rh-3** and the individual tetranuclear metallomacrocycle are observed. Red squares mark the resonances assigned to the metallomacrocycle. These observations are consistent with partial dissociation of the nested dimer in solution. The hydrogen protons of NH on the ligand lose their NMR signal due to the exchange with D in CD_3OD.

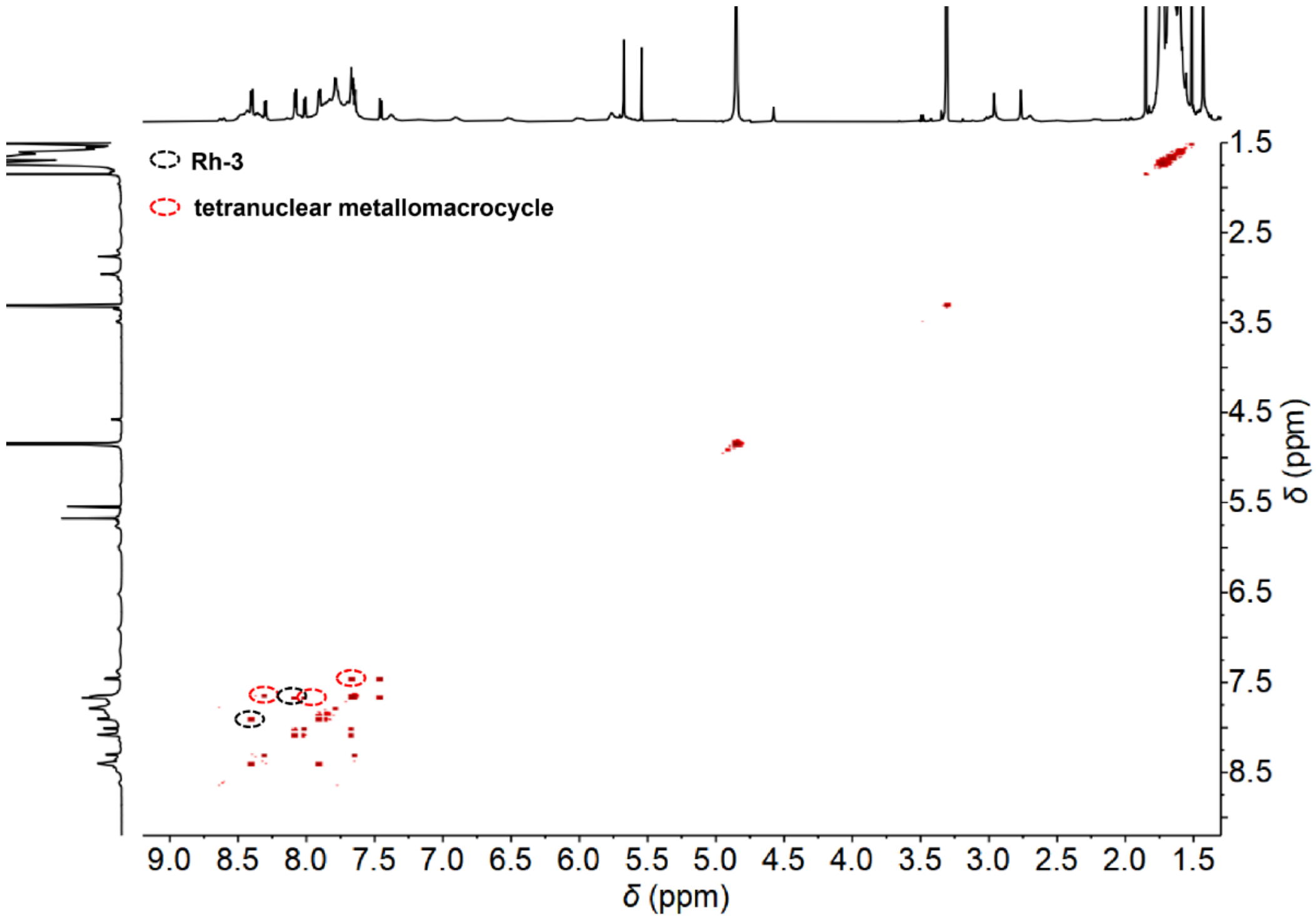

Fig. S121. ^{1}H-^{1}H COSY spectrum of a solution prepared by dissolving crystals of **Rh-3** in CD_3OD (600 MHz, c_{Rh} = 36.0 mM). Black and red dashed ellipses highlight selected cross-peaks assigned to the nested dimer **Rh-3** and a coexisting tetranuclear metallomacrocycle, respectively.

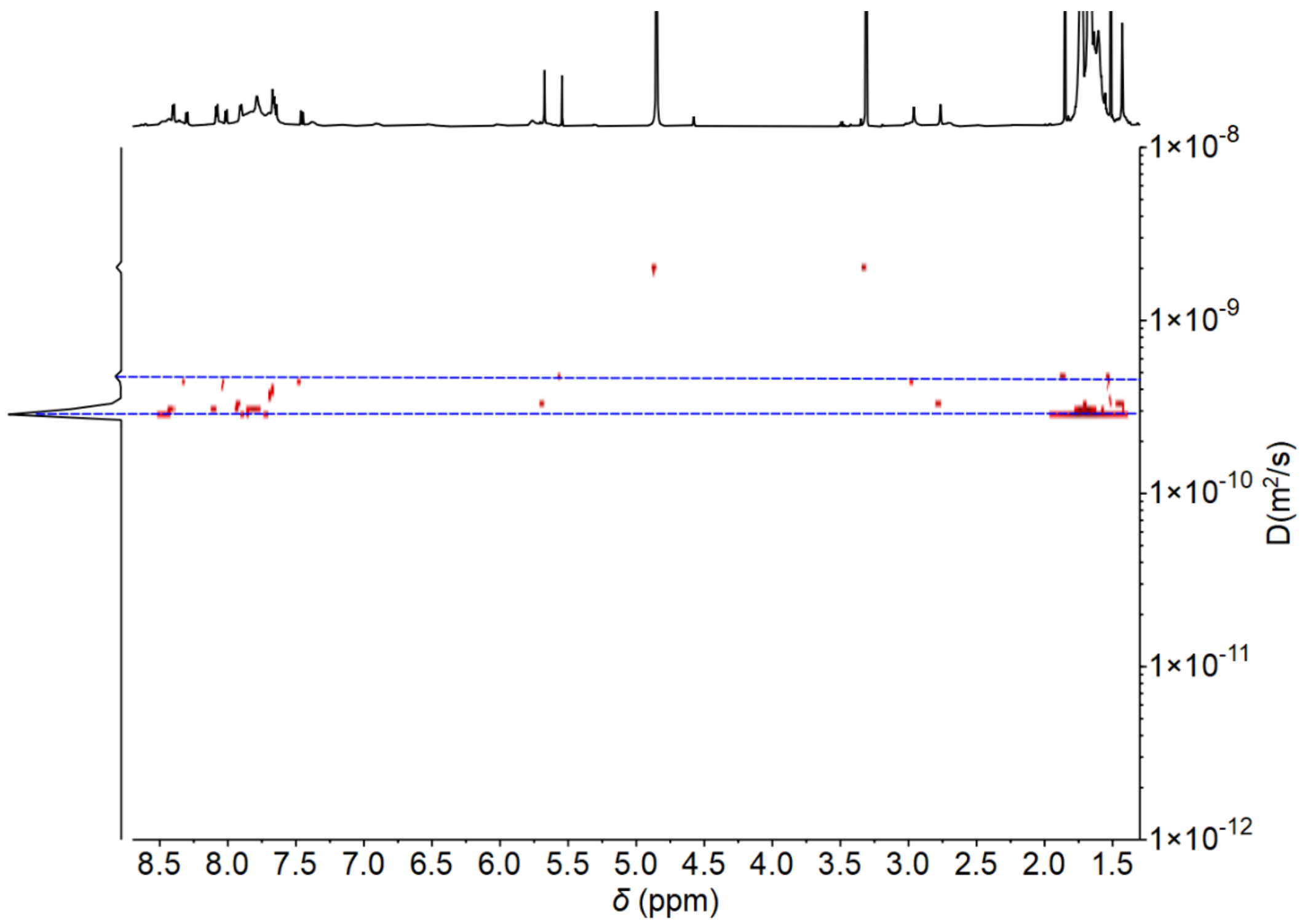

Fig. S122. ¹H DOSY spectrum of a solution prepared by dissolving crystals of **Rh-3** in CD_3OD (600 MHz, c_{Rh} = 36.0 mM). Two diffusion components are assigned to **Rh-3** (D = 2.86 × 10^{-10} m² s⁻¹) and the individual tetranuclear metallomacrocycle (D = 4.77 × 10^{-10} m² s⁻¹).

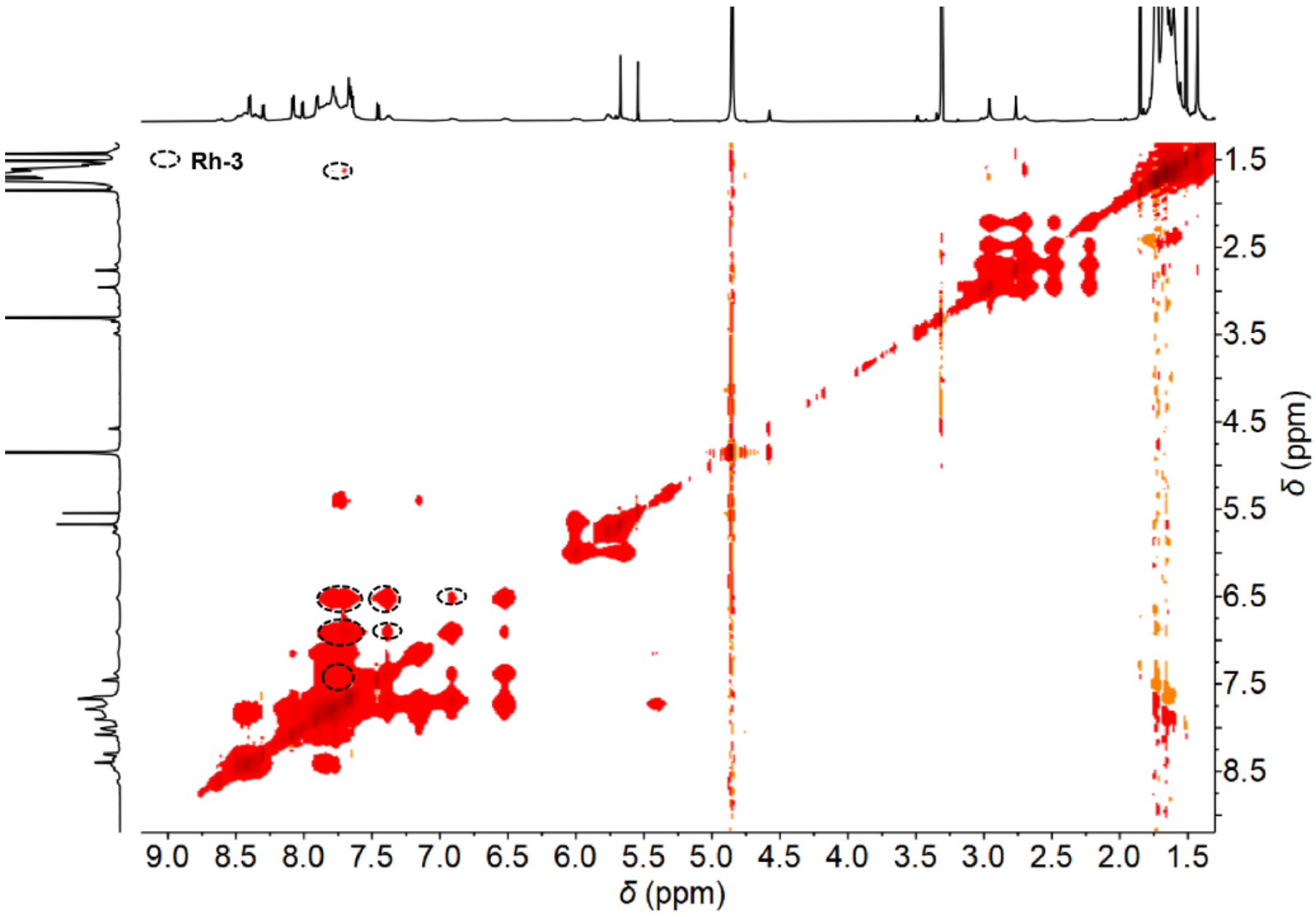

Fig. S123. ¹H-¹H NOESY spectrum of a solution prepared by dissolving crystals of **Rh-3** in CD₃OD (600 MHz, c_{Rh} = 36.0 mM). Black dashed ellipses highlight selected cross-peaks assigned to the nested dimer **Rh-3**.

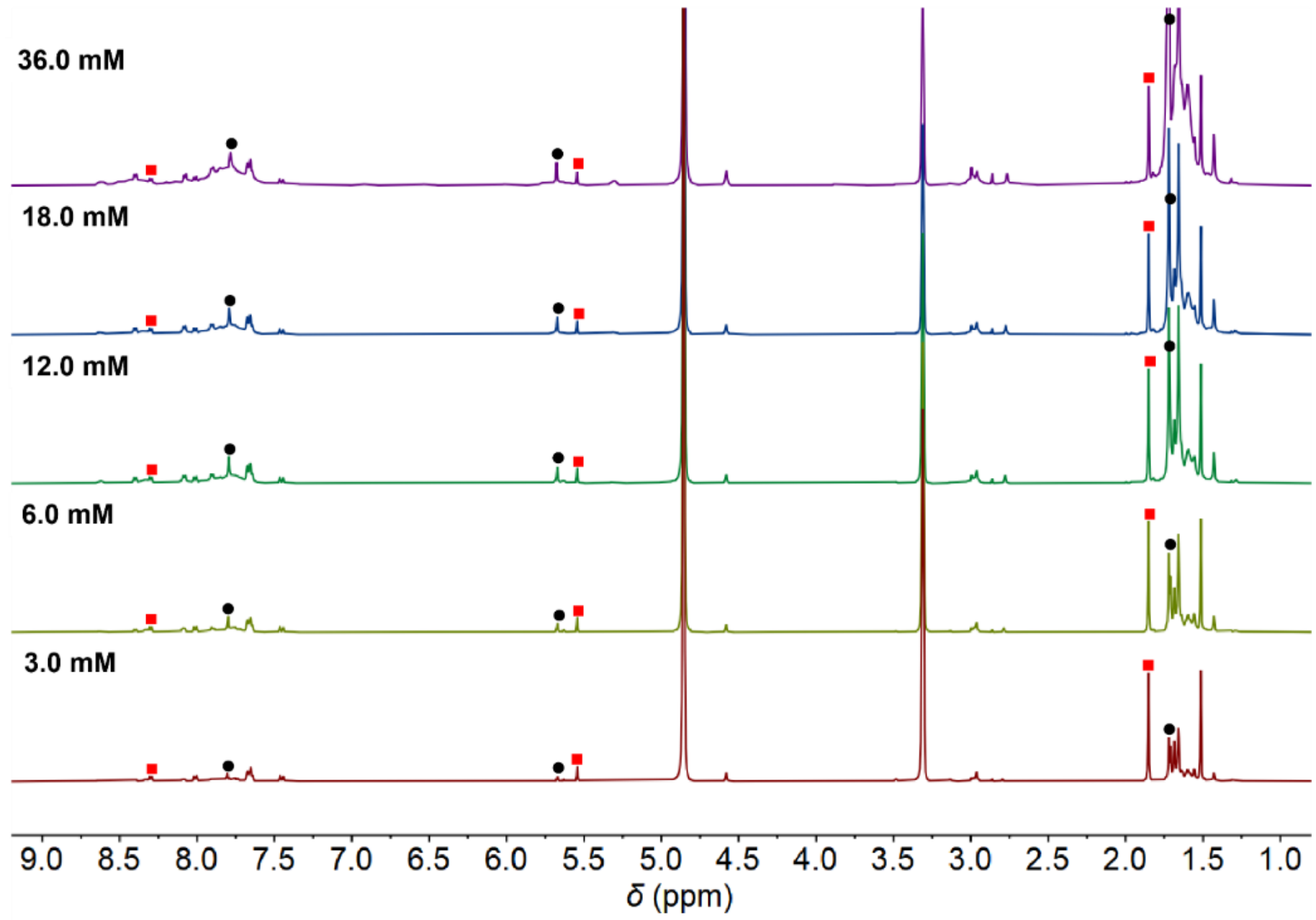

Fig. S124. Concentration-dependent ^{1}H NMR spectra of solutions prepared by dissolving crystalline **Rh-3** in CD_3OD (400 MHz, room temperature; c_{Rh} = 3.0–36.0 mM). Black circles mark selected resonances assigned to the nested dimer **Rh-3**, and red squares mark selected resonances assigned to individual tetranuclear metallomacrocycles. Upon dilution, the nested-dimer resonances become less prominent relative to the resonances marked with red squares.

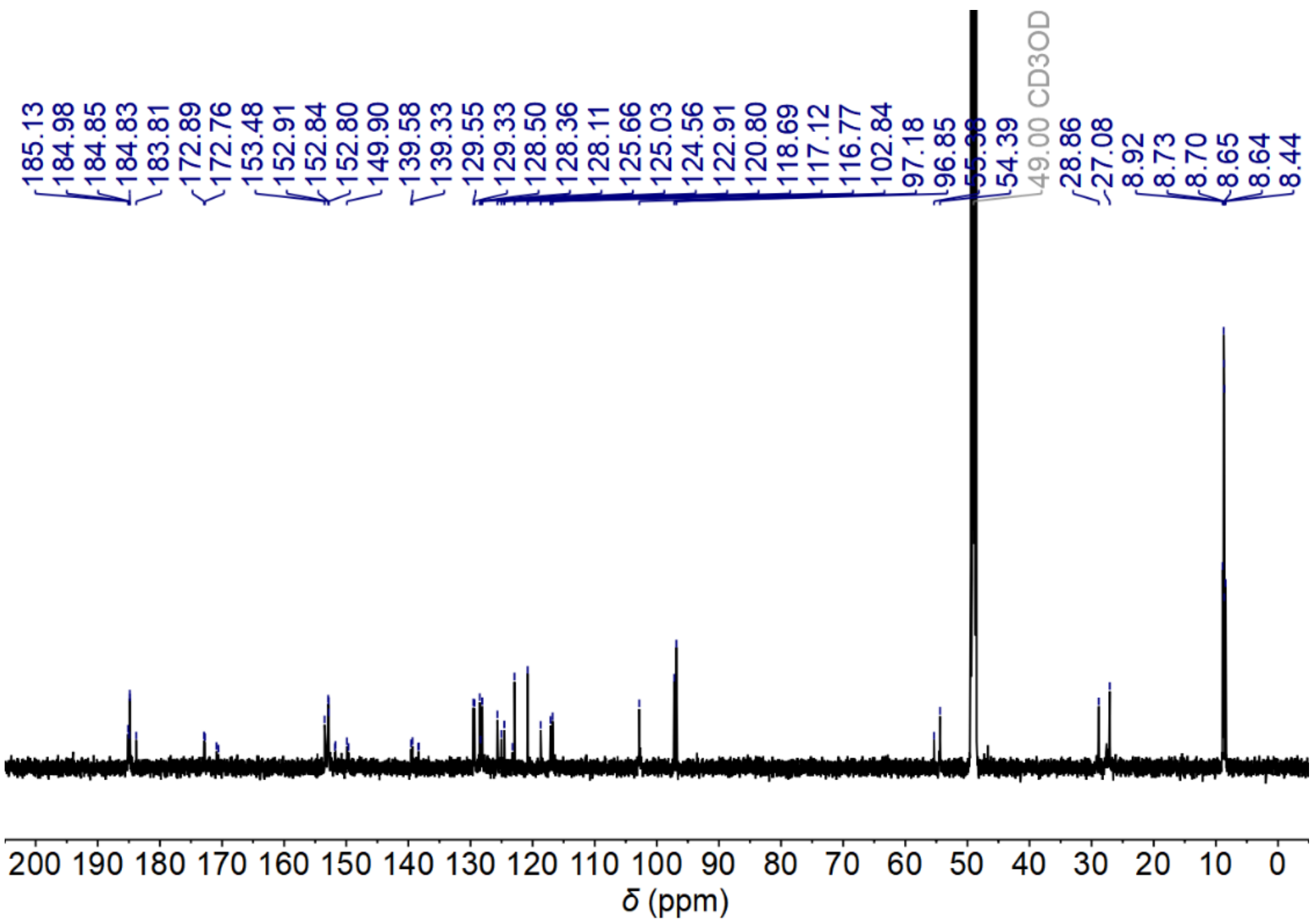

Fig. S125. $^{13}C\{^{1}H\}$ NMR spectrum of a solution prepared by dissolving crystals of **Rh-3** in CD_3OD (151 MHz, c_{Rh} = 36.0 mM).

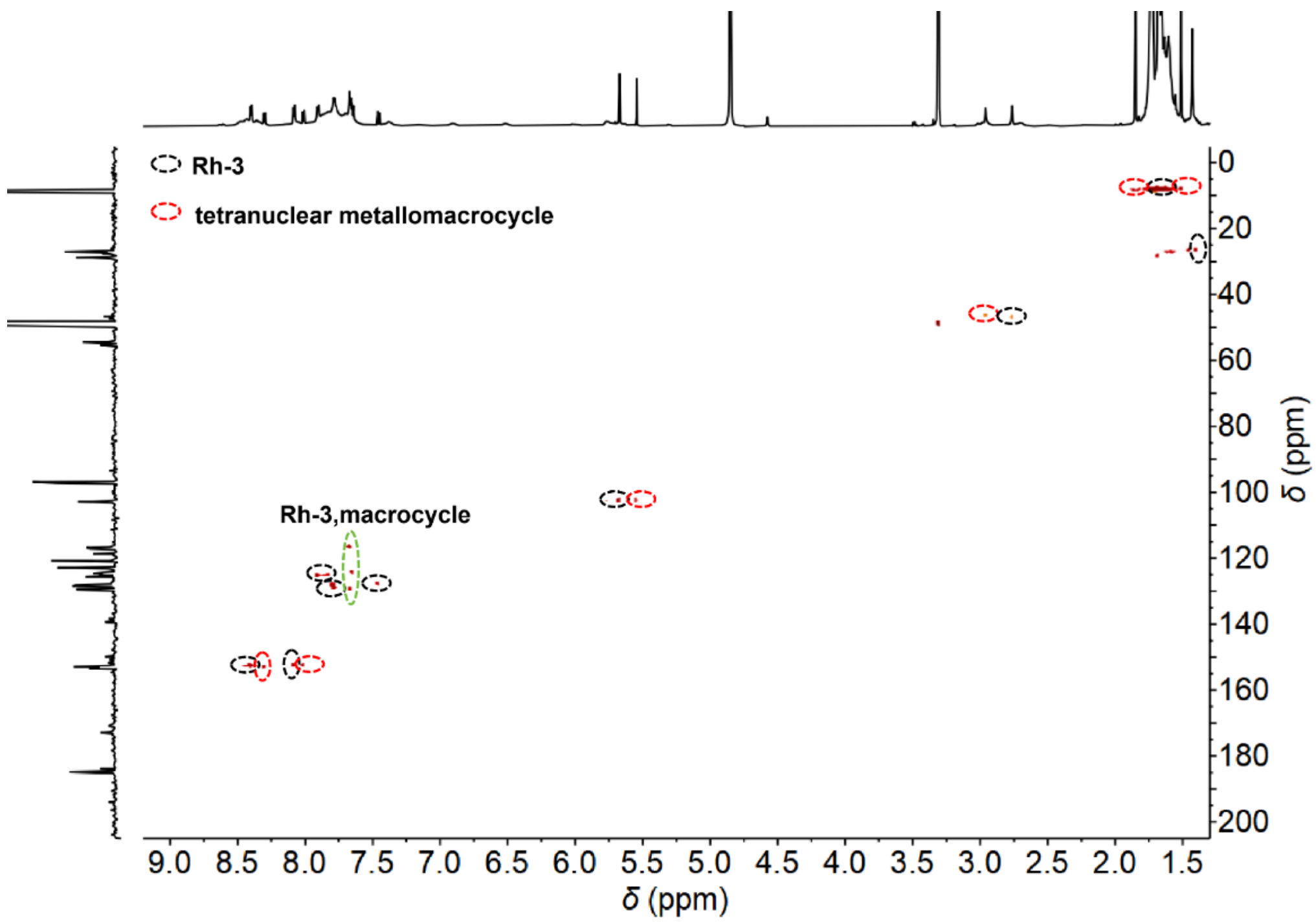

Fig. S126. ^{1}H–^{13}C HSQC spectrum of a solution prepared by dissolving crystals of **Rh-3** in CD_3OD (600 MHz, c_{Rh} = 36.0 mM). Black and red dashed ellipses highlight selected cross-peaks assigned to the nested dimer **Rh-3** and a coexisting tetranuclear metallomacrocycle, respectively.

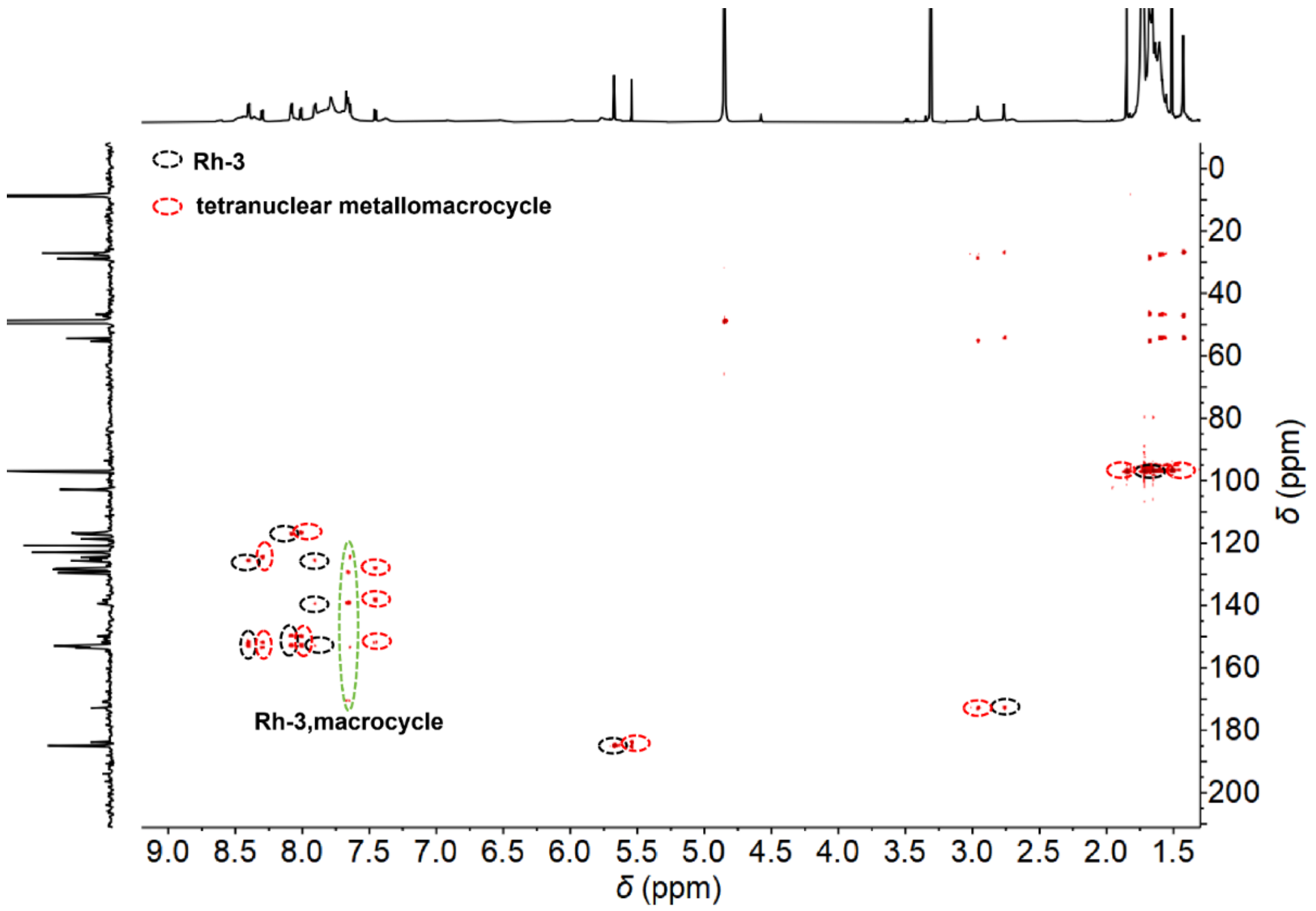

Fig. S127. ^1H–^{13}C HMBC spectrum of a solution prepared by dissolving crystals of **Rh-3** in CD_3OD (600 MHz, c_{Rh} = 36.0 mM). Black and red dashed ellipses highlight selected cross-peaks assigned to the nested dimer **Rh-3** and a coexisting tetranuclear metallomacrocycle, respectively.

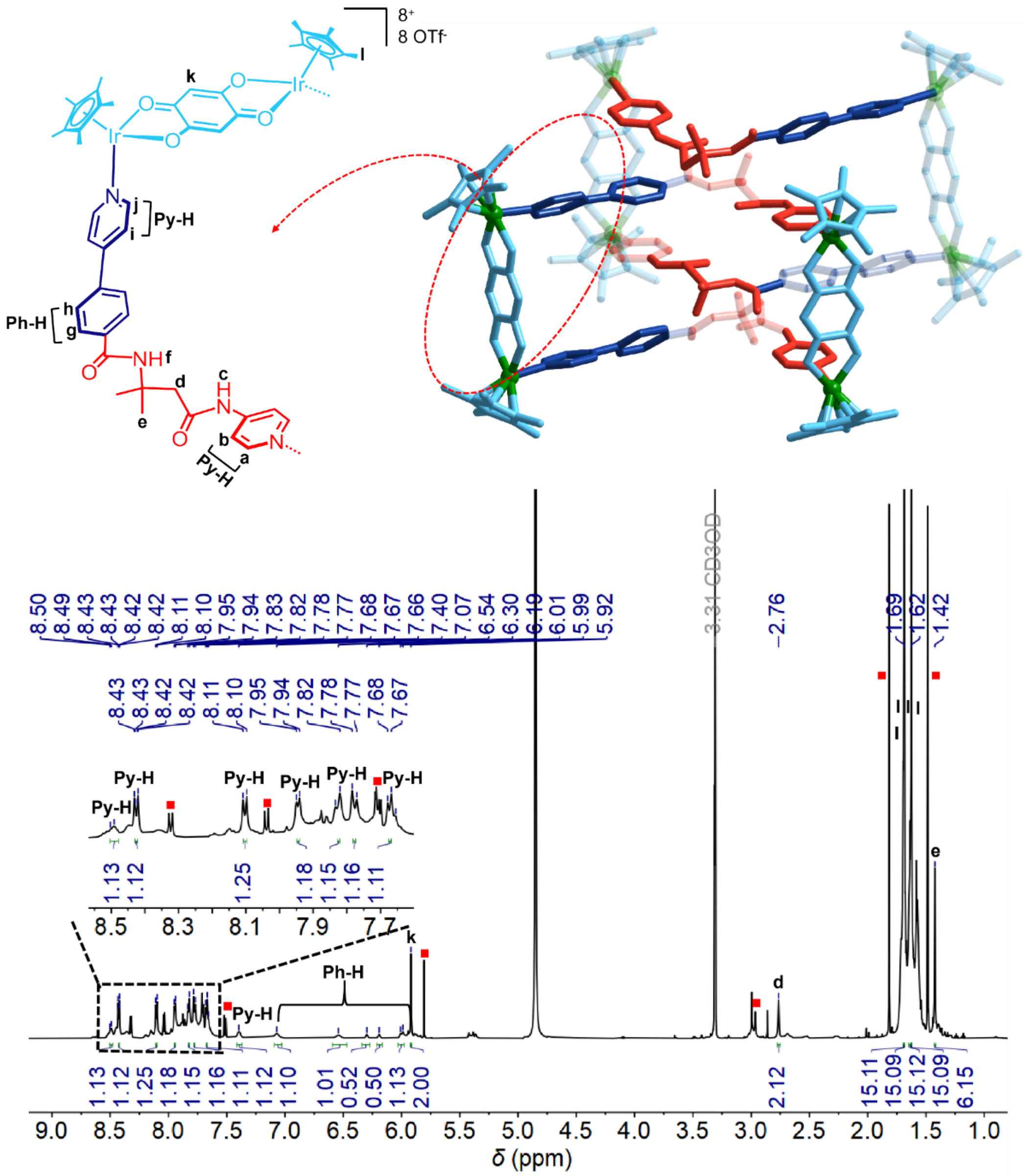

Fig. S128. ^{1}H NMR spectrum of a solution prepared by dissolving crystals of **Ir-3** in CD_3OD (600 MHz, c_{Ir} = 36.0 mM). Resonances assigned to the nested dimer **Ir-3** and the individual tetranuclear metallomacrocycle are observed. Red squares mark the resonances assigned to the metallomacrocycle. These observations are consistent with partial dissociation of the nested dimer in solution. The hydrogen protons of NH on the ligand lose their NMR signal due to the exchange with D in CD_3OD.

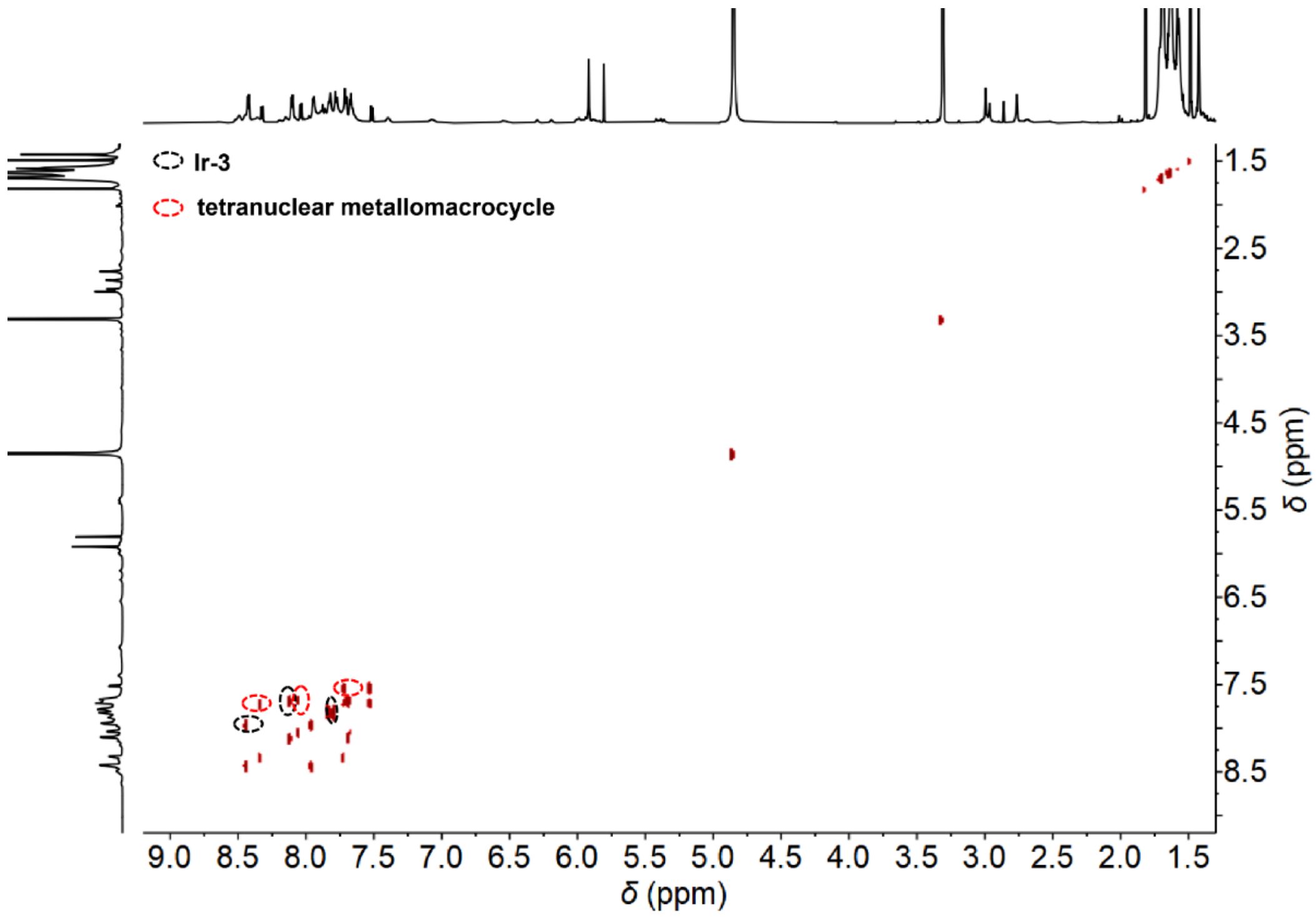

Fig. S129. ^1H-^1H COSY spectrum of a solution prepared by dissolving crystals of **Ir-3** in CD_3OD (600 MHz, c_{Ir} = 36.0 mM). Black and red dashed ellipses highlight selected cross-peaks assigned to the nested dimer **Ir-3** and a coexisting tetranuclear metallomacrocycle, respectively.

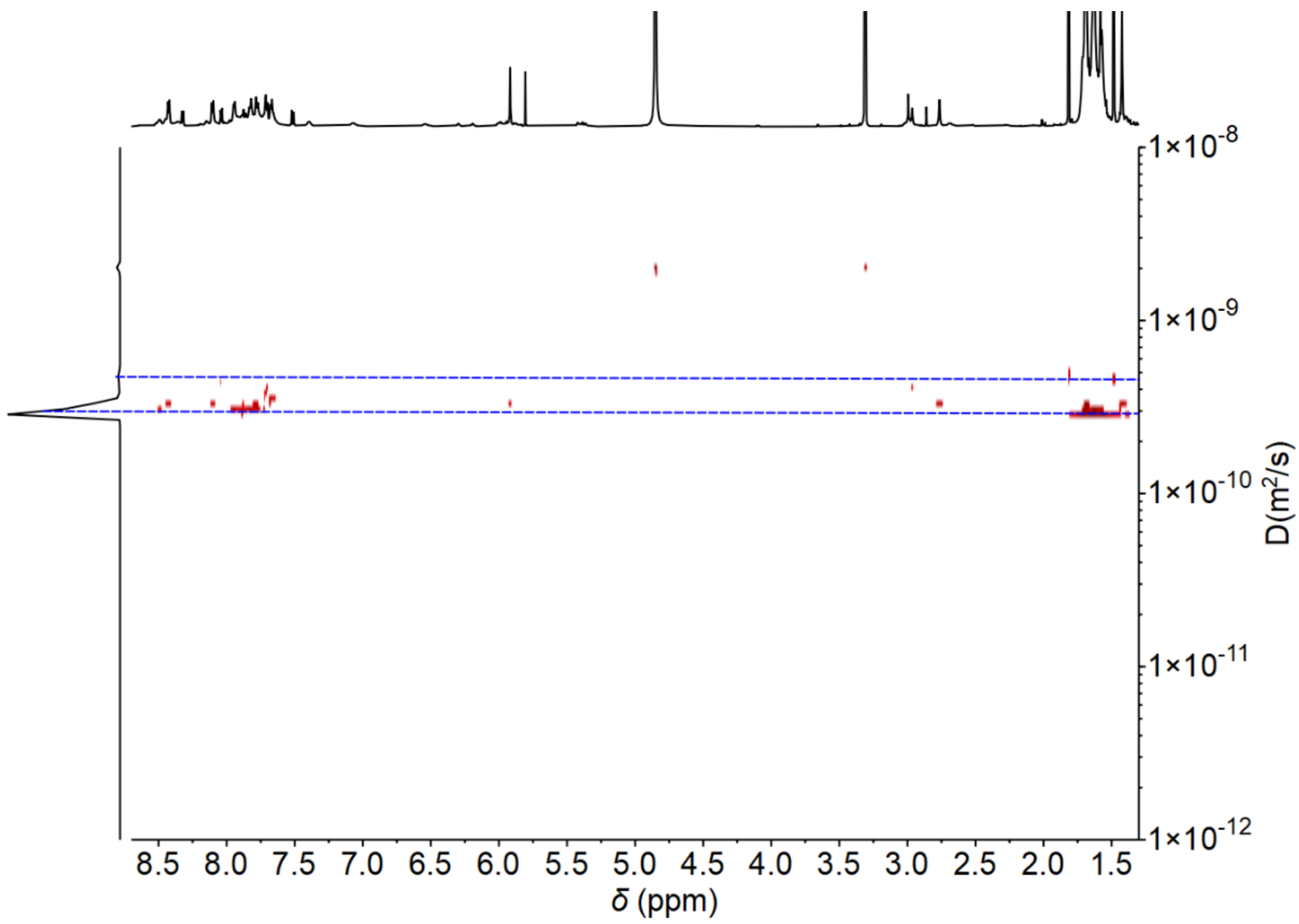

Fig. S130. ¹H DOSY spectrum of a solution prepared by dissolving crystals of **Ir-3** in CD_3OD (600 MHz, c_{Ir} = 36.0 mM). Two diffusion components are assigned to **Ir-3** (D= 2.86 × 10^{-10} m^2 s^{-1}) and the individual tetranuclear metallomacrocycle (D= 4.75 × 10^{-10} m^2 s^{-1}).

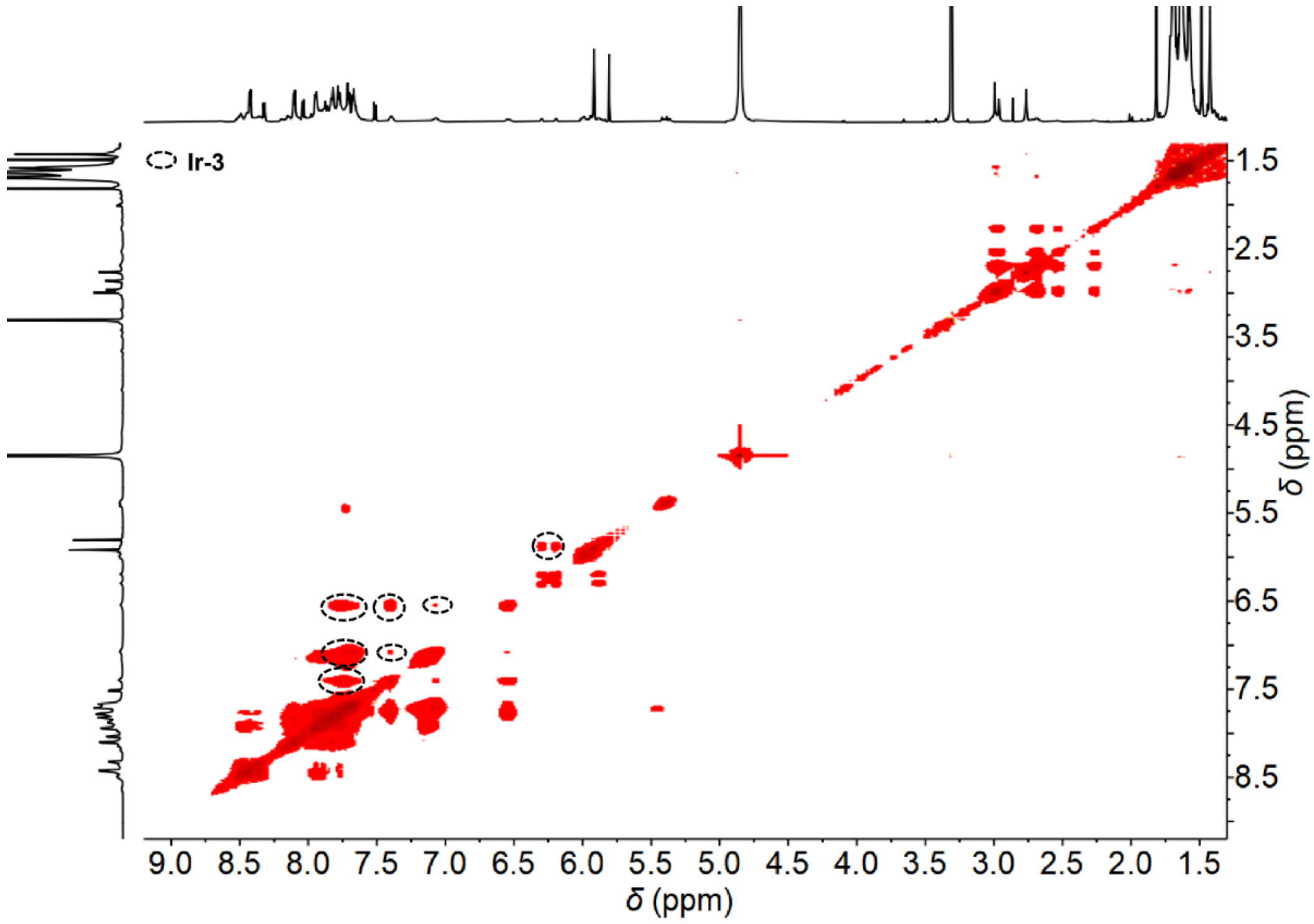

Fig. S131. ¹H-¹H NOESY spectrum of a solution prepared by dissolving crystals of **Ir-3** in CD₃OD (600 MHz, c_{Ir} = 36.0 mM). Black dashed ellipses highlight selected cross-peaks assigned to the nested dimer **Ir-3**.

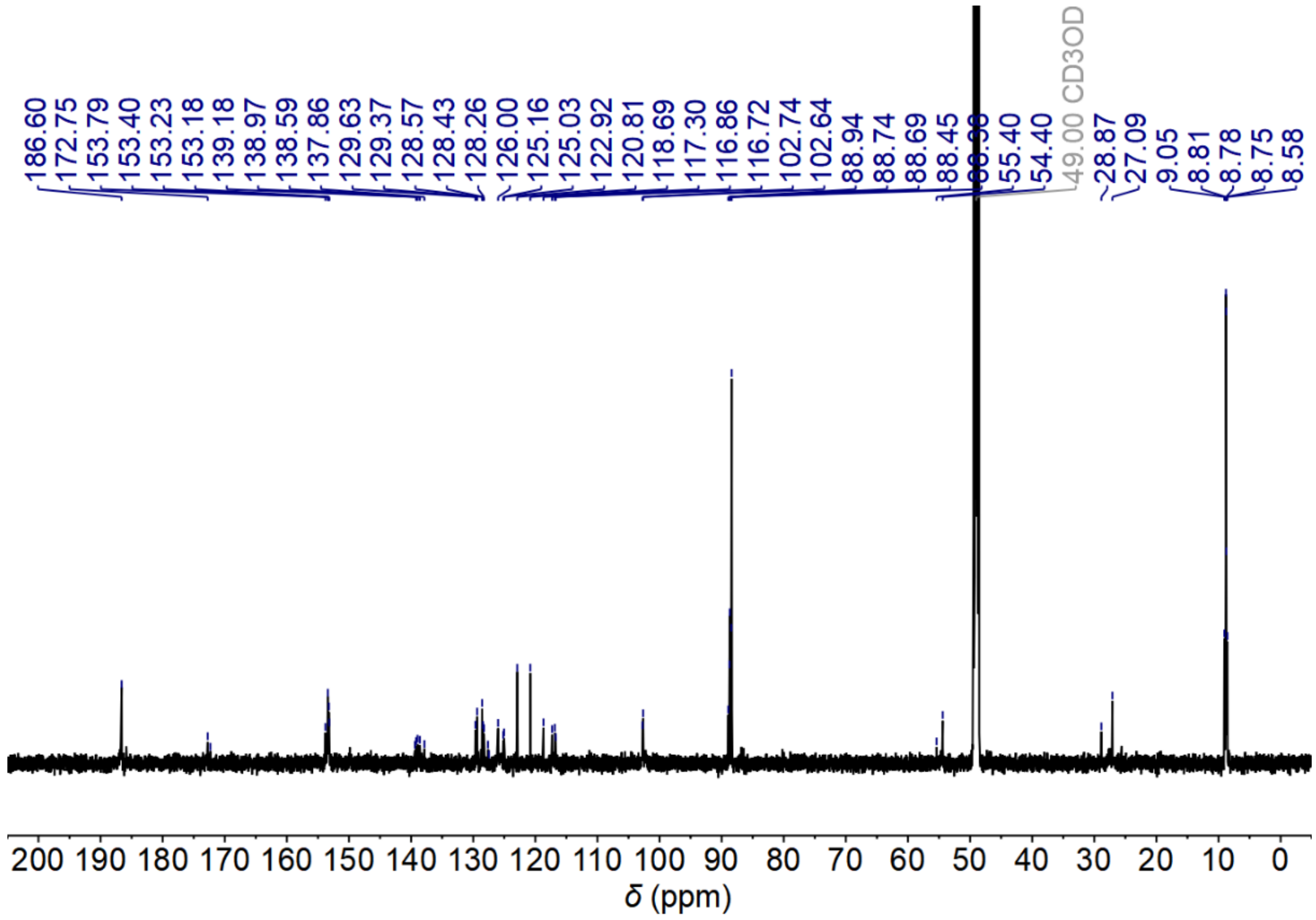

Fig. S132. $^{13}C\{^{1}H\}$ NMR spectrum of a solution prepared by dissolving crystals of **Ir-3** in CD_3OD (151 MHz, c_{Ir} = 36.0 mM).

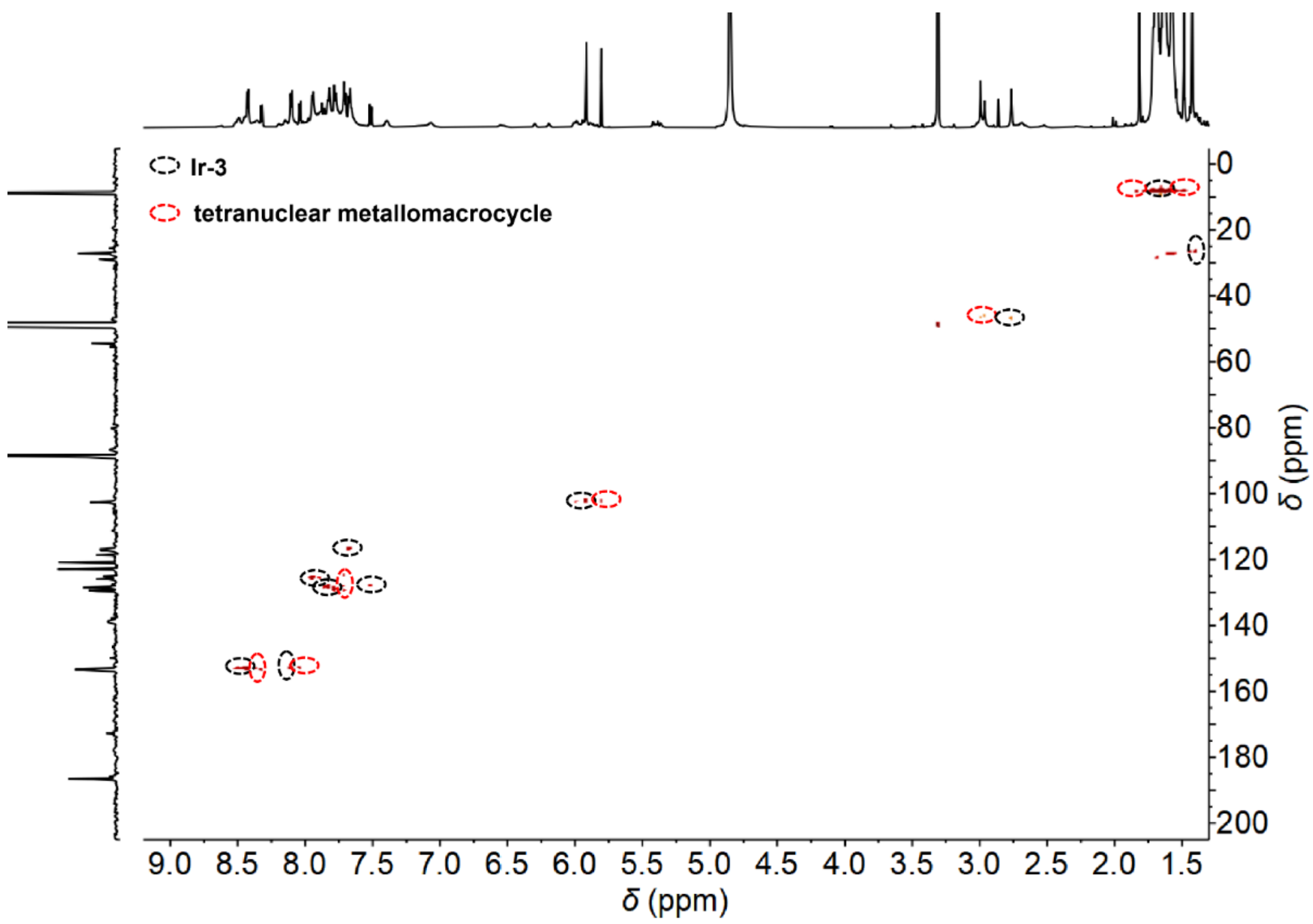

Fig. S133. ^1H–^{13}C HSQC spectrum of a solution prepared by dissolving crystals of **Ir-3** in CD_3OD (600 MHz, c_{Ir} = 36.0 mM). Black and red dashed ellipses highlight selected cross-peaks assigned to the nested dimer **Ir-3** and a coexisting tetranuclear metallomacrocycle, respectively.

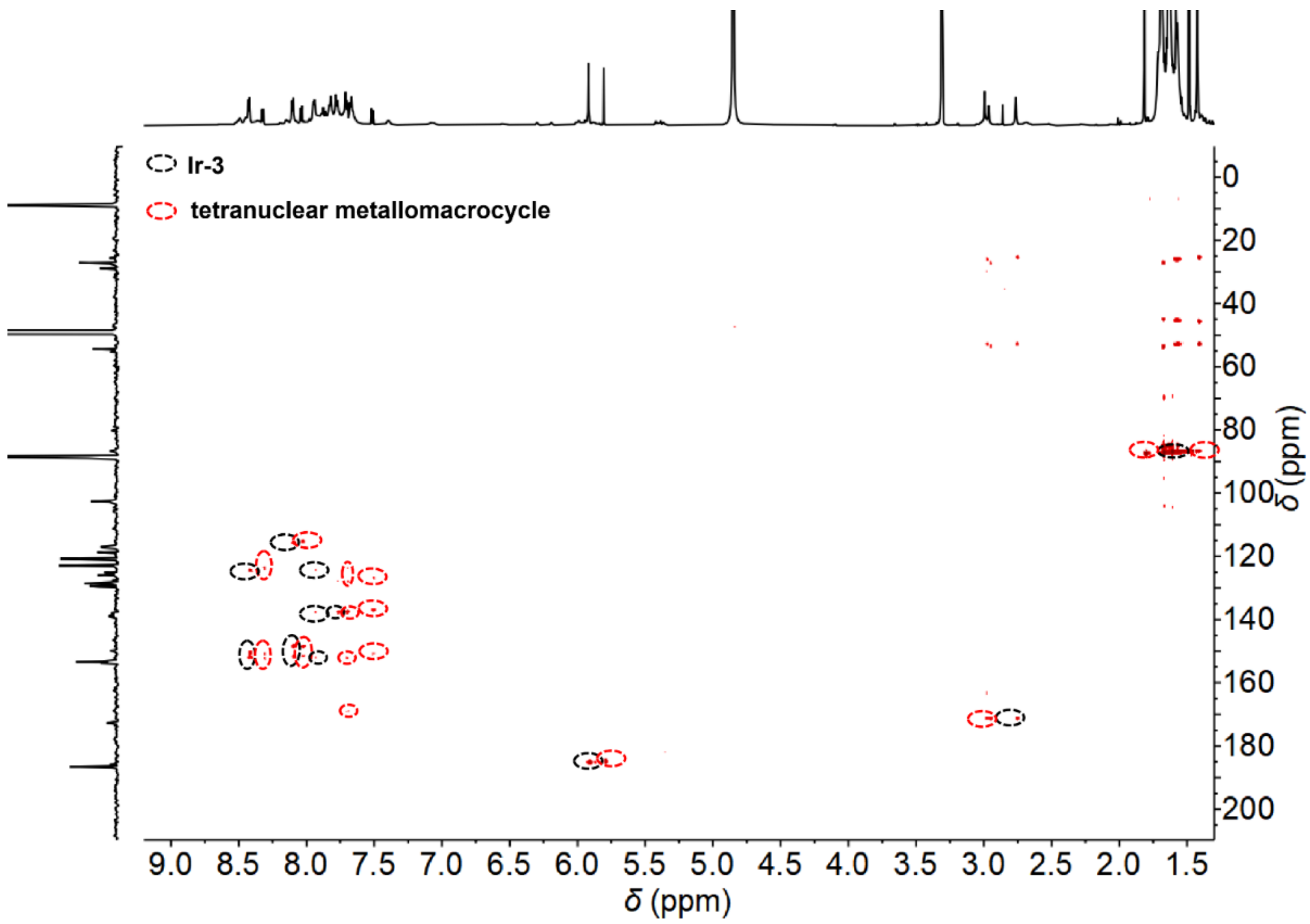

Fig. S134. ^1H–^{13}C HMBC spectrum of a solution prepared by dissolving crystals of **Ir-3** in CD_3OD (600 MHz, c_{Ir} = 36.0 mM). Black and red dashed ellipses highlight selected cross-peaks assigned to the nested dimer **Ir-3** and a coexisting tetranuclear metallomacrocycle, respectively.

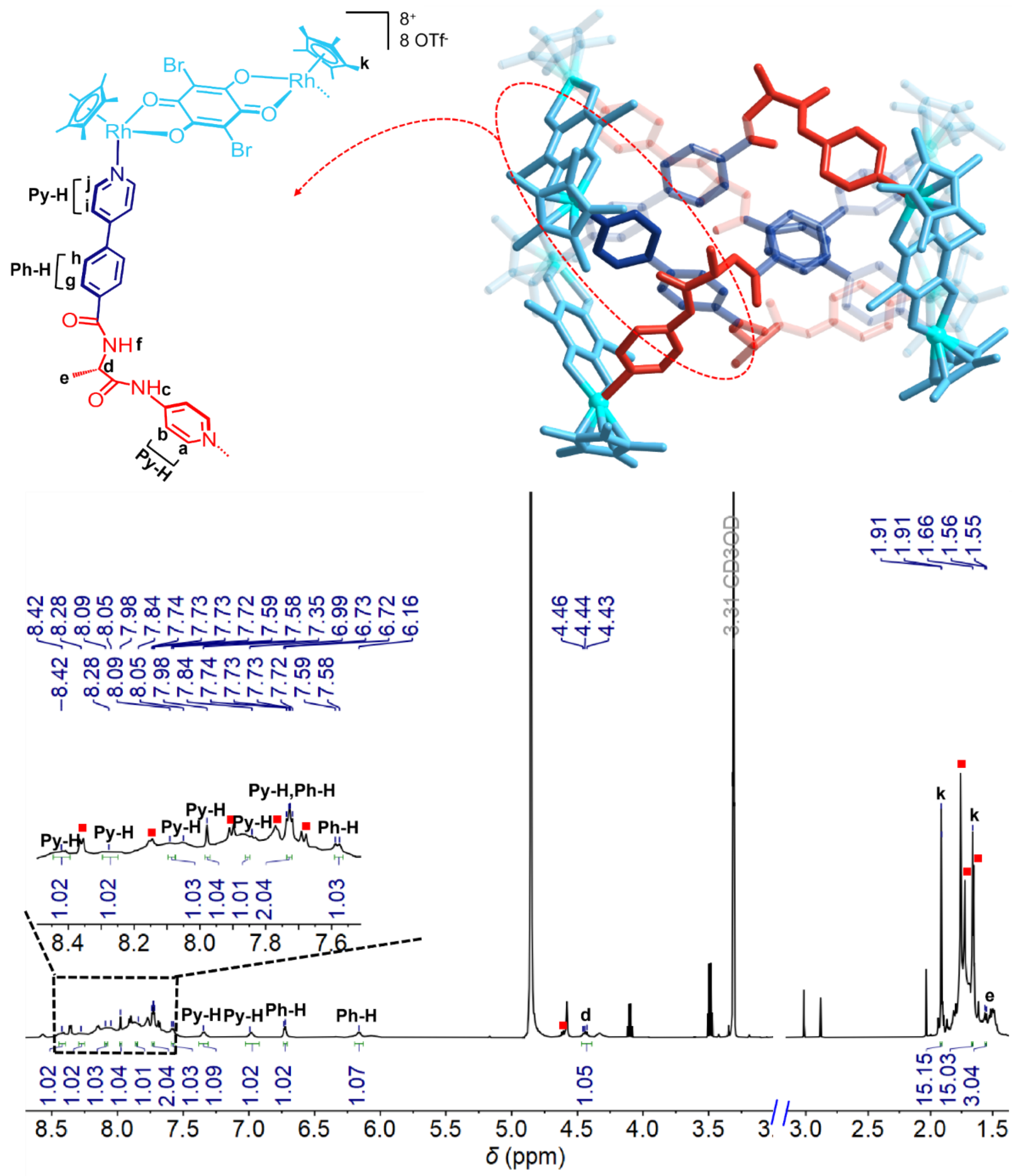

Fig. S135. ^{1}H NMR spectrum of a solution prepared by dissolving crystals of **Rh-4Br*S*** in CD_3OD (600 MHz, c_{Rh} = 36.0 mM). Resonances assigned to the figure-eight knot **Rh-4Br*S*** are observed together with additional resonances tentatively assigned to a tetranuclear metallomacrocycle. Red squares mark the latter resonances. The hydrogen protons of NH on the ligand lose their NMR signal due to the exchange with D in CD_3OD.

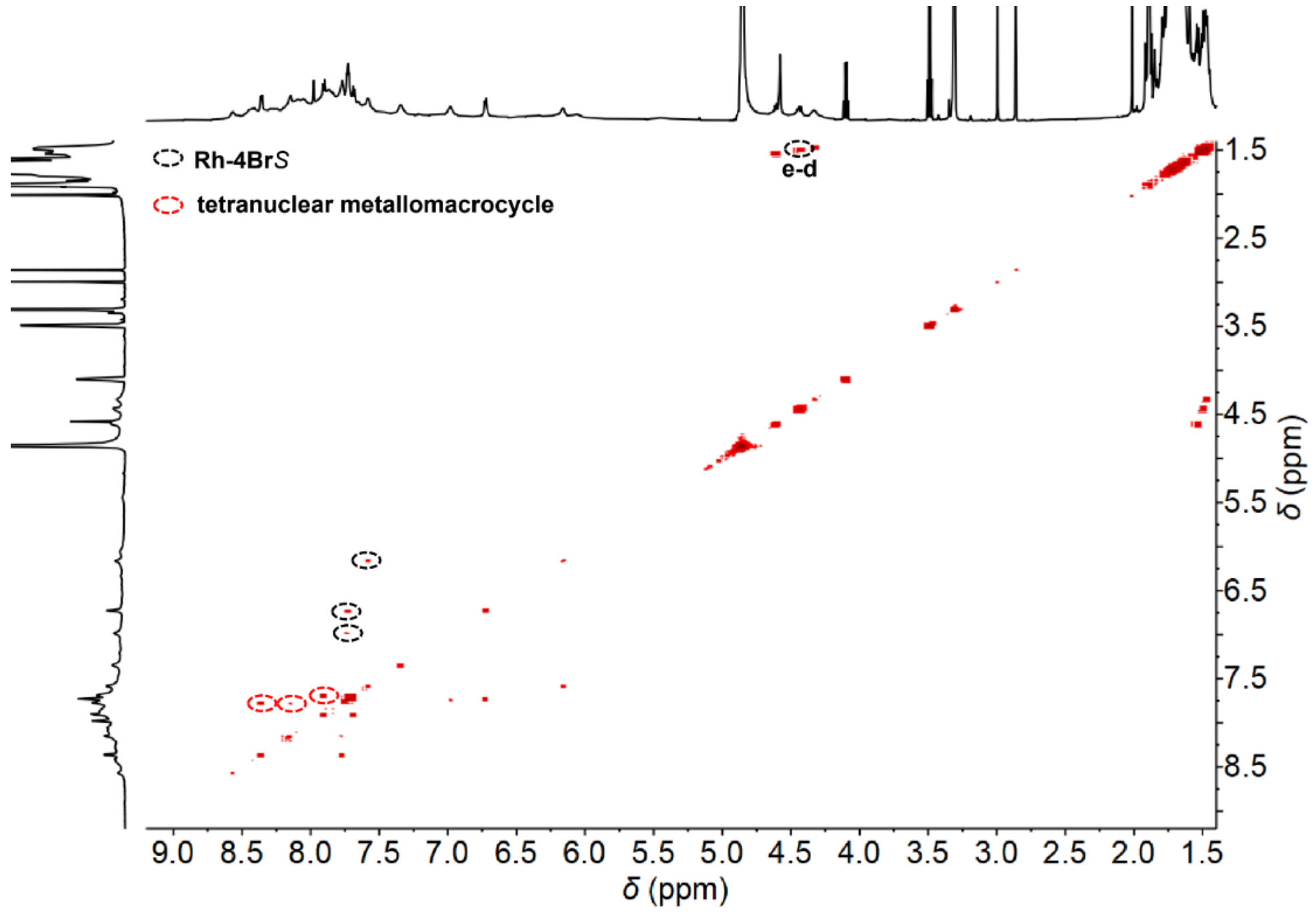

Fig. S136. ^1H-^1H COSY spectrum of a solution prepared by dissolving crystals of **Rh-4Br*S*** in CD_3OD (600 MHz, c_{Rh} = 36.0 mM). Black dashed ellipses highlight selected cross-peaks assigned to the figure-eight knot **Rh-4Br*S***, whereas red dashed ellipses highlight those tentatively assigned to a tetranuclear metallomacrocycle.

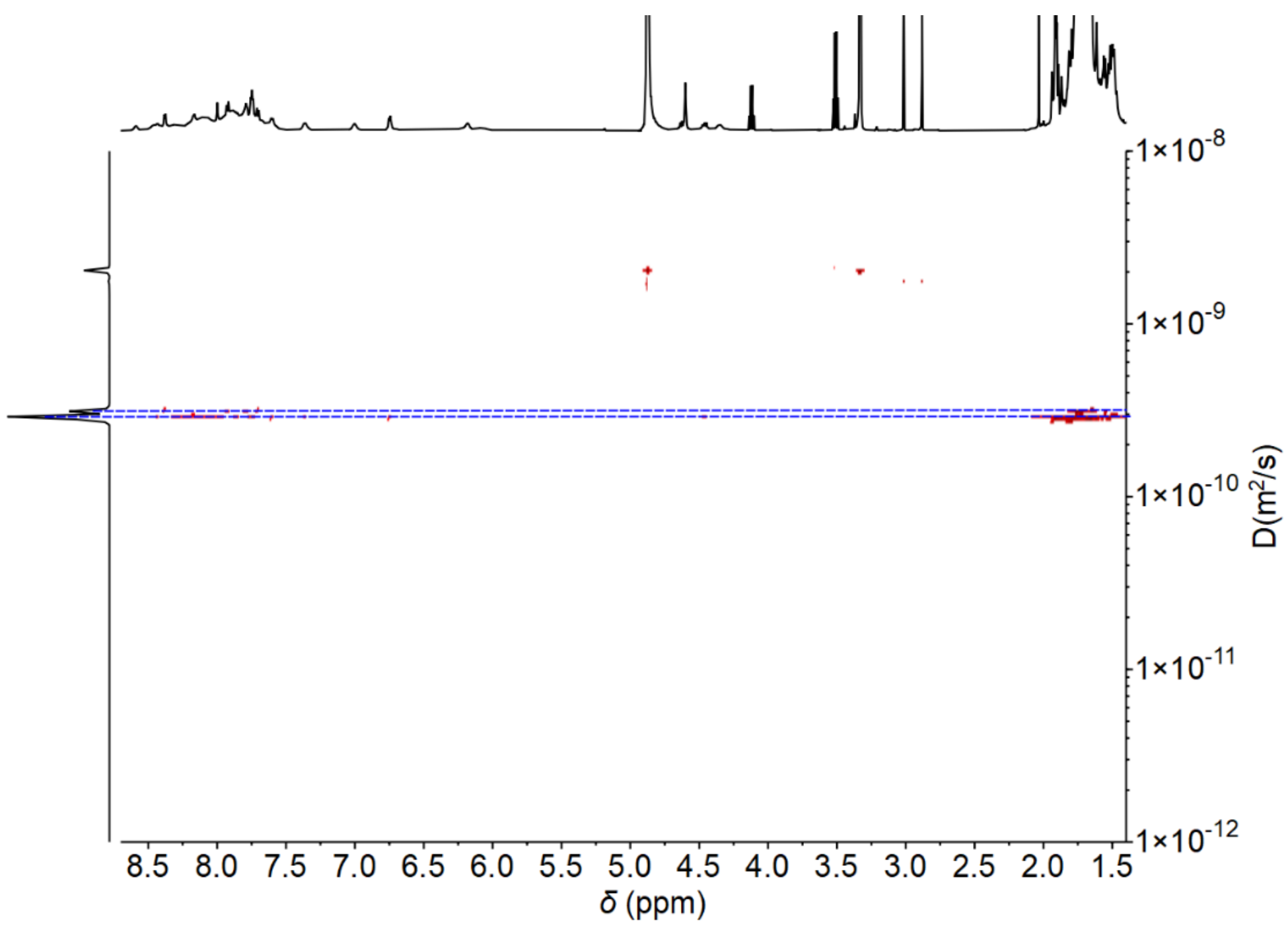

Fig. S137. ¹H DOSY spectrum of a solution prepared by dissolving crystals of **Rh-4Br*S*** in CD_3OD (600 MHz, c_{Rh} = 36.0 mM). Two diffusion components are observed, with $D = 2.90 \times 10^{-10}$ m² s⁻¹ assigned to **Rh-4Br*S*** and $D = 3.12 \times 10^{-10}$ m² s⁻¹ tentatively assigned to a tetranuclear metallomacrocycle.

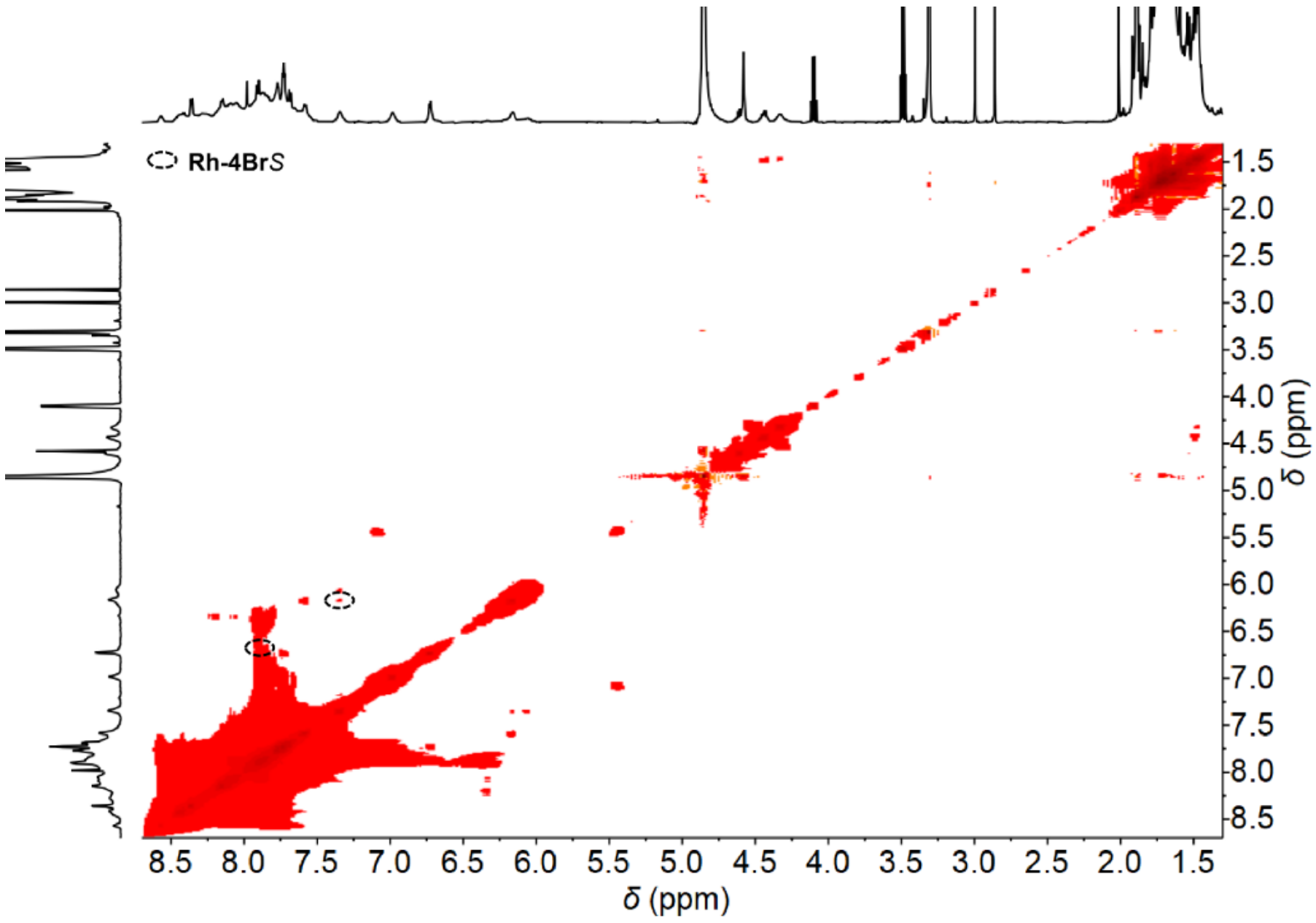

Fig. S138. ¹H-¹H NOESY spectrum of a solution prepared by dissolving crystals of **Rh-4Br*S*** in CD_3OD (600 MHz, c_{Rh} = 36.0 mM). Black dashed ellipses highlight selected cross-peaks assigned to the figure-eight knot **Rh-4Br*S***.

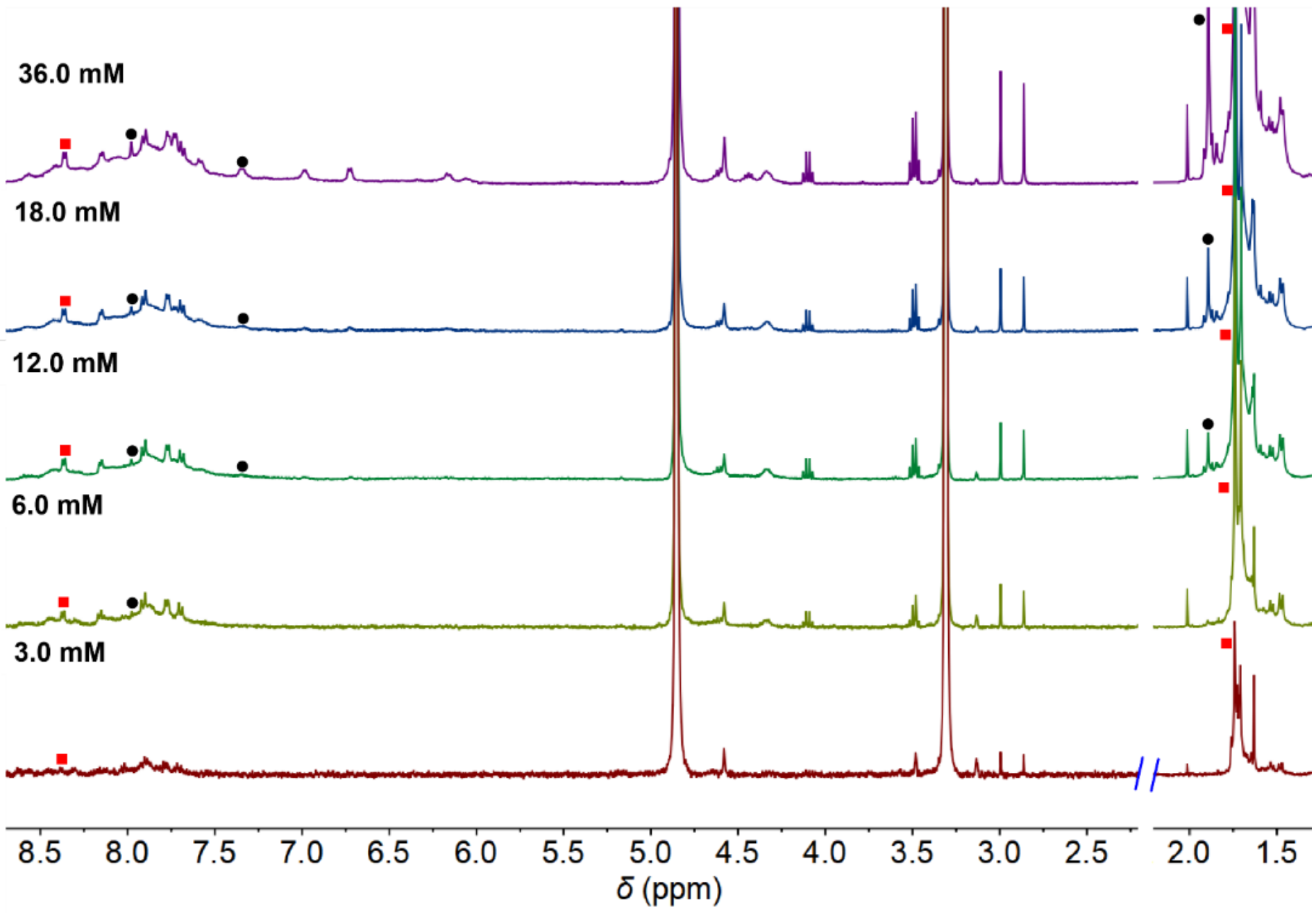

Fig. S139. Concentration-dependent ^{1}H NMR spectra of solutions prepared by dissolving crystals of **Rh-4Br*S*** in CD_3OD (400 MHz, room temperature; c_{Rh} = 3.0–36.0 mM). Black circles mark selected resonances assigned to the figure-eight knot **Rh-4Br*S***, and red squares mark selected additional resonances tentatively assigned to a tetranuclear metallomacrocycle. Upon dilution, the figure-eight-knot resonances become less prominent relative to the additional resonances.

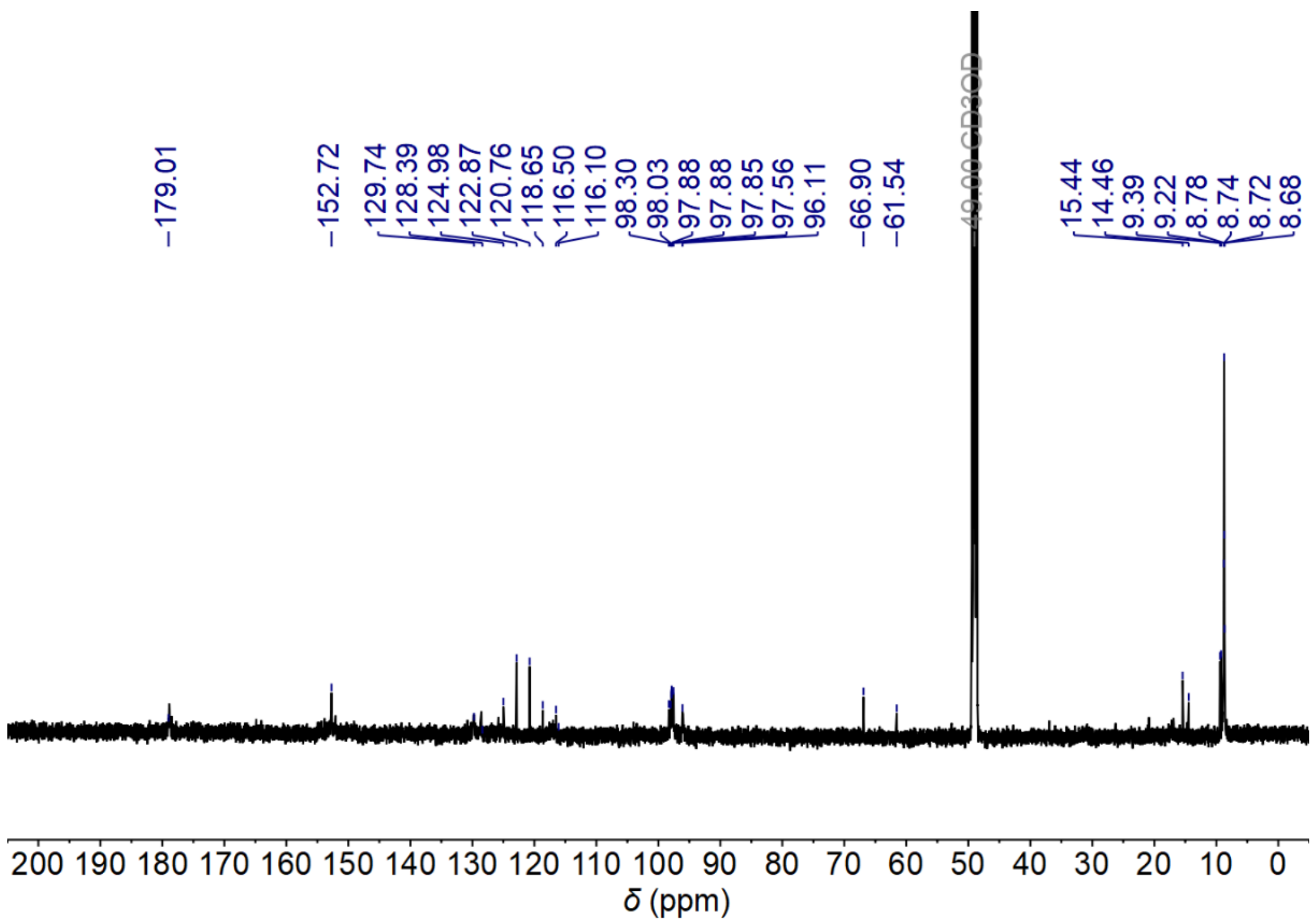

Fig. S140. $^{13}C\{^{1}H\}$ NMR spectrum of a solution prepared by dissolving crystals of **Rh-4Br*S*** in CD_3OD (151 MHz, c_{Rh} = 36.0 mM).

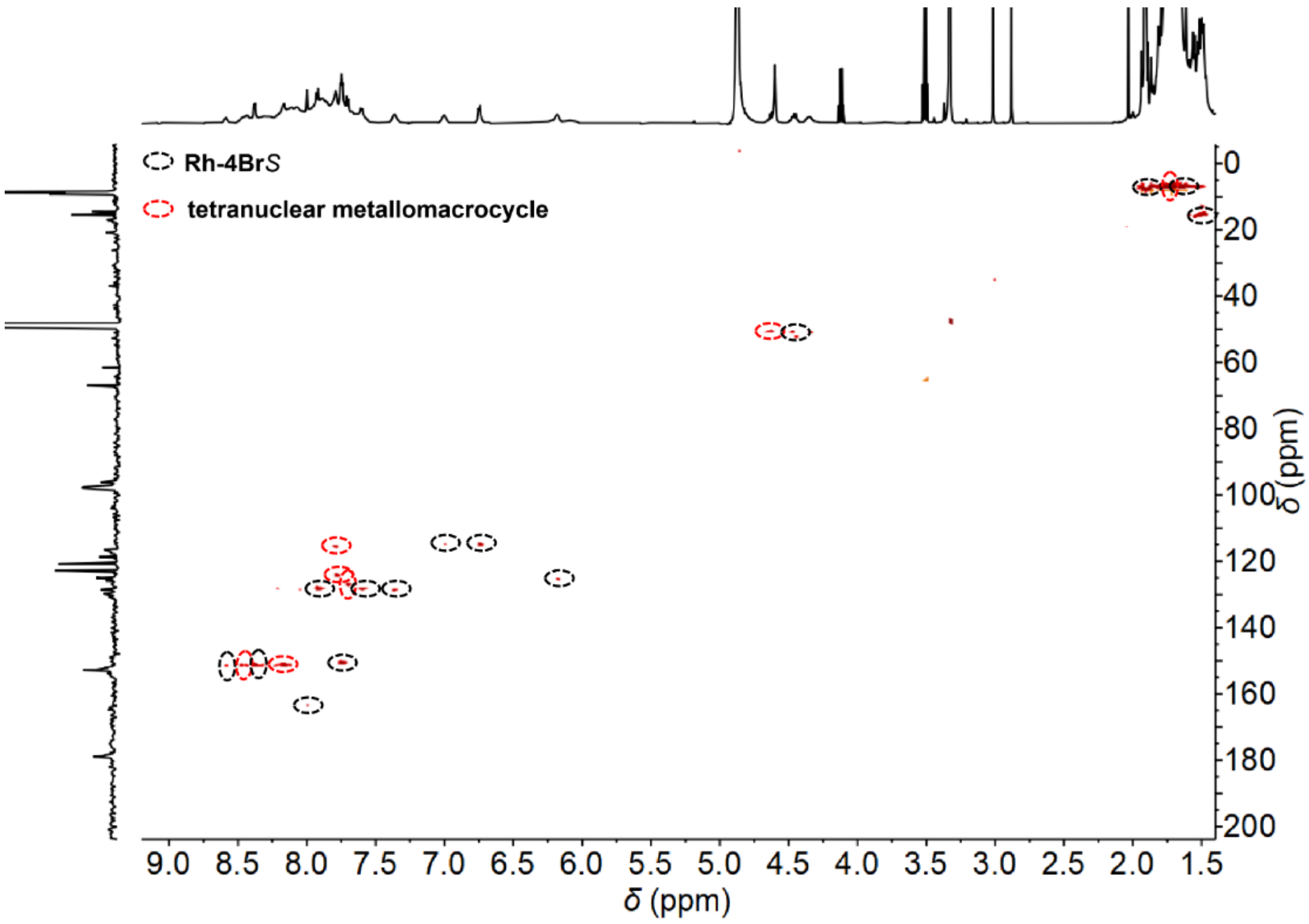

Fig. S141. ^{1}H–^{13}C HSQC spectrum of a solution prepared by dissolving crystals of **Rh-4Br*S*** in CD_3OD (600 MHz, c_{Rh} = 36.0 mM). Black dashed ellipses highlight selected cross-peaks assigned to the figure-eight knot **Rh-4Br*S***, whereas red dashed ellipses highlight those tentatively assigned to a tetranuclear metallomacrocycle.

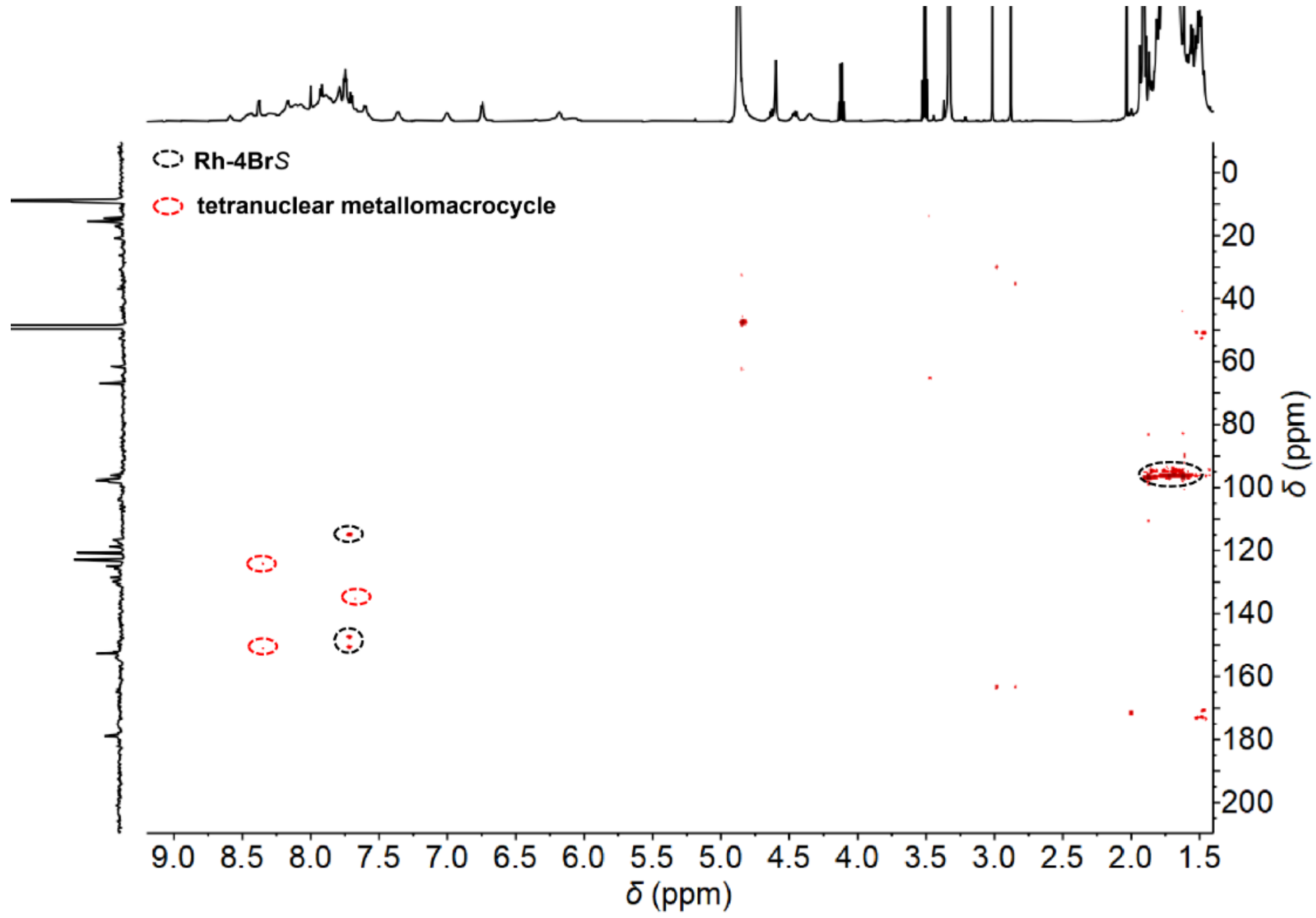

Fig. S142. ^{1}H–^{13}C HMBC spectrum of a solution prepared by dissolving crystals of **Rh-4Br*S*** in CD_3OD (600 MHz, c_{Rh} = 36.0 mM). Black dashed ellipses highlight selected cross-peaks assigned to the figure-eight knot **Rh-4Br*S***, whereas red dashed ellipses highlight those tentatively assigned to a tetranuclear metallomacrocycle.

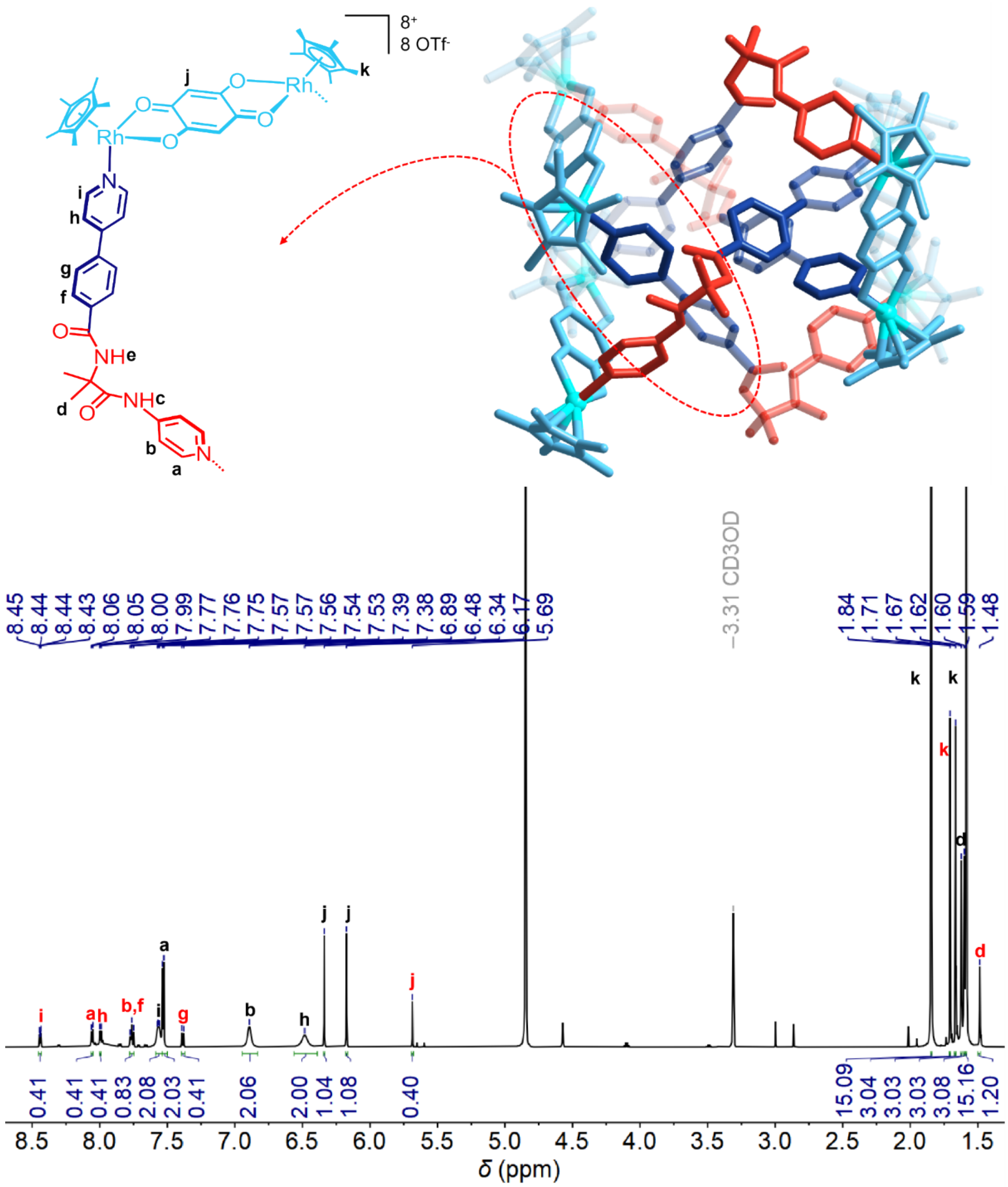

Fig. S143. ^{1}H NMR spectrum of a solution prepared by dissolving crystalline material containing **Rh-5** and **Rh-5′** in CD_3OD (600 MHz, c_{Rh} = 64.0 mM). Black and red letters label proton resonances assigned to the figure-eight knot **Rh-5** and the tetranuclear metallomacrocycle **Rh-5′**, respectively. The knot resonances are more prominent under these conditions. The hydrogen protons of NH on the ligand lose their NMR signal due to the exchange with D in CD_3OD. The resonances expected for four protons (2 H at f and 2 H at g) of the **Rh-5** are not clearly resolved, presumably because of shielding in the tightly entangled **Rh-5**.

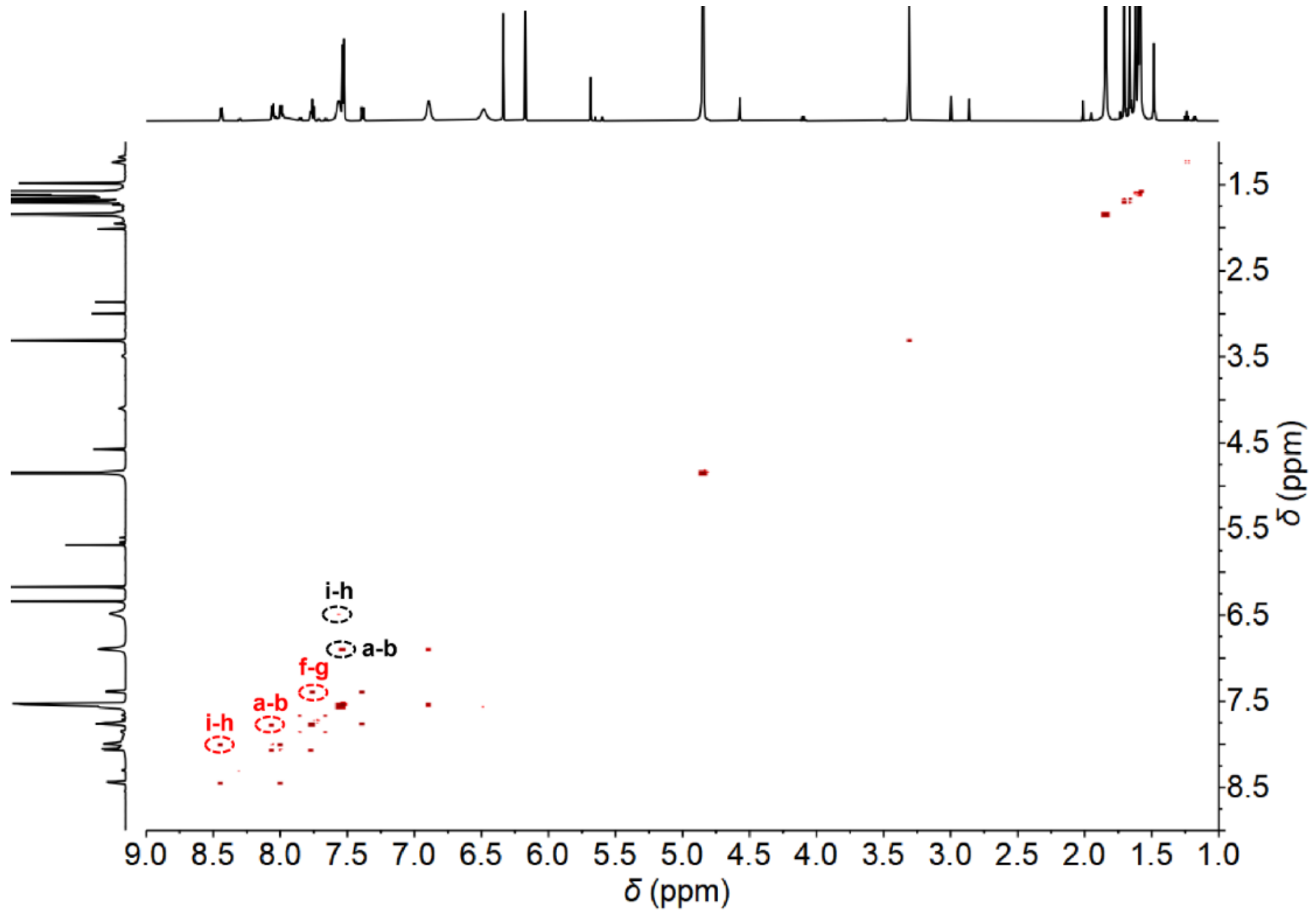

Fig. S144. ^{1}H–^{1}H COSY spectrum of a solution prepared by dissolving crystalline material containing **Rh-5** and **Rh-5′** in CD_3OD (600 MHz, c_{Rh} = 64.0 mM). Black and red dashed ellipses highlight selected cross-peaks assigned to the figure-eight knot **Rh-5** and the tetranuclear metallomacrocycle **Rh-5′**, respectively.

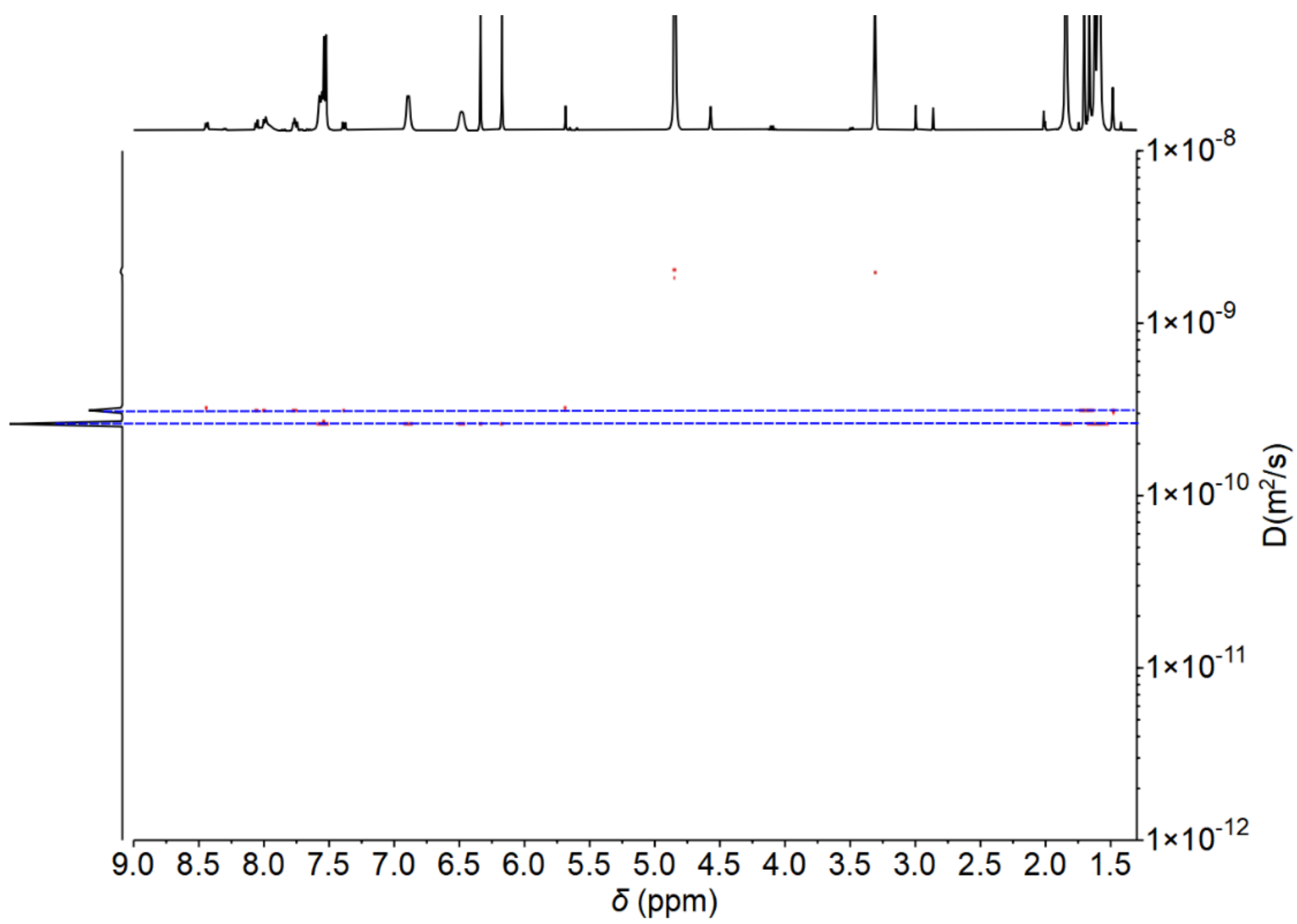

Fig. S145. ^{1}H DOSY spectrum of a solution prepared by dissolving crystalline material containing **Rh-5** and **Rh-5′** in CD_3OD (600 MHz, c_{Rh} = 64.0 mM). Two diffusion components are assigned to **Rh-5** (D = 2.60×10^{-10} m² s⁻¹) and **Rh-5′** (D = 3.12×10^{-10} m² s⁻¹).

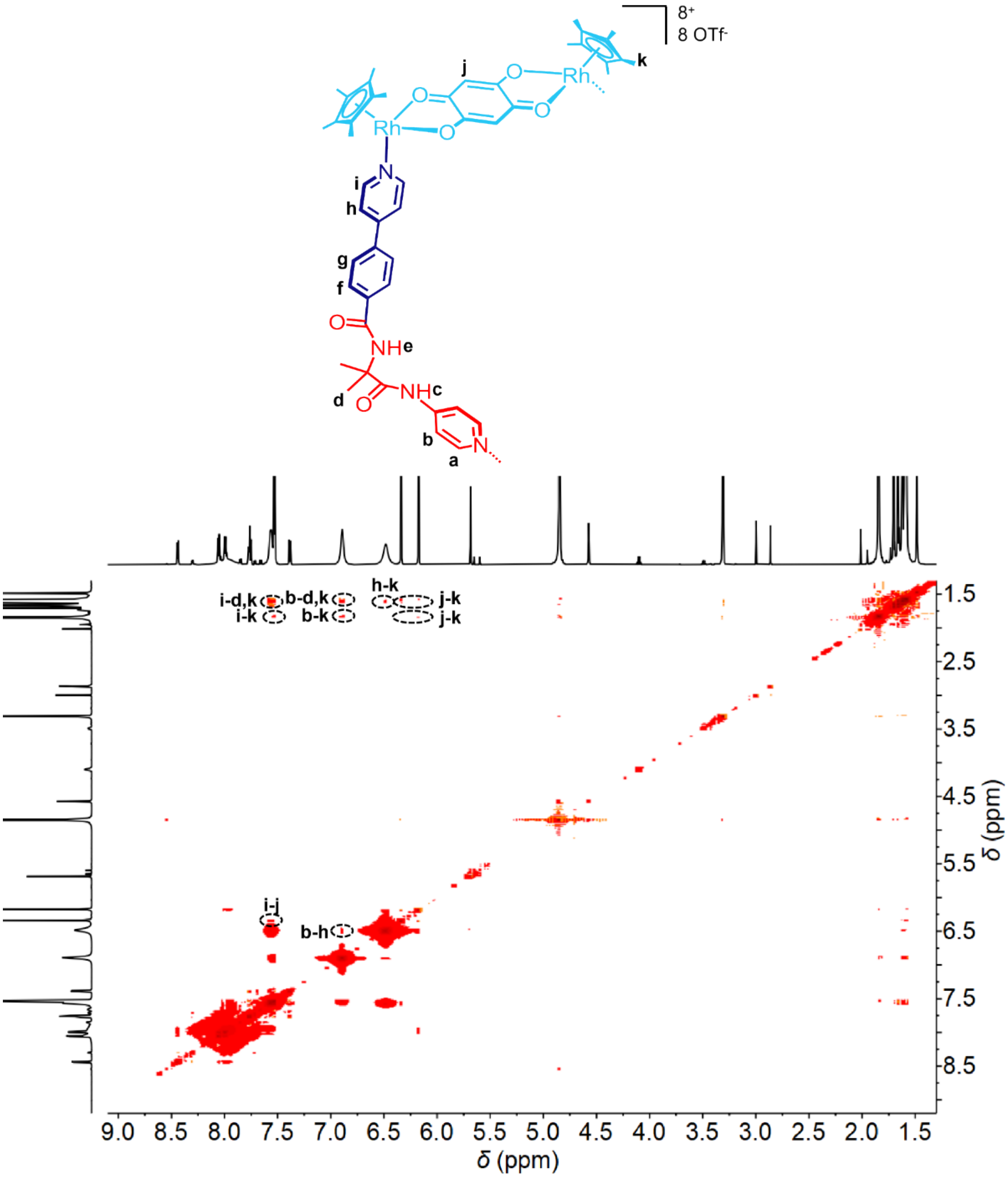

Fig. S146. ^{1}H-^{1}H NOESY spectrum of a solution prepared by dissolving crystalline material containing **Rh-5** and **Rh-5′** in CD_3OD (600 MHz, c_{Rh} = 64.0 mM). Black dashed ellipses highlight selected cross-peaks assigned to the figure-eight knot **Rh-5**.

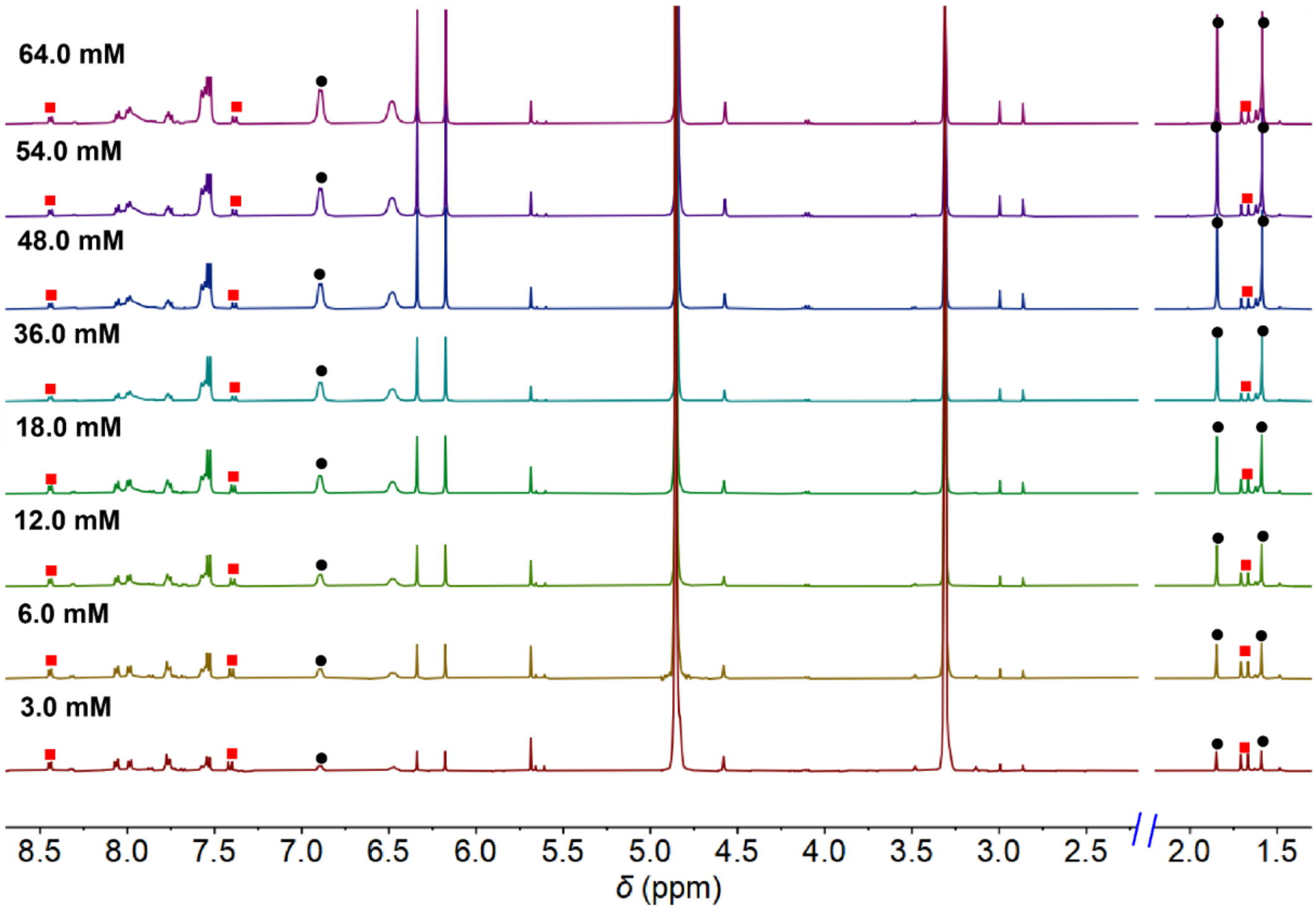

Fig. S147. Concentration-dependent ¹H NMR spectra of solutions prepared by dissolving crystalline material containing **Rh-5** and **Rh-5′** in CD_3OD (600 MHz, room temperature; c_{Rh} = 3.0–64.0 mM). Black circles and red squares mark selected resonances assigned to the figure-eight knot **Rh-5** and the tetranuclear metallomacrocycle **Rh-5′**, respectively. Upon dilution, the resonances of **Rh-5** become less prominent relative to those of **Rh-5′**.

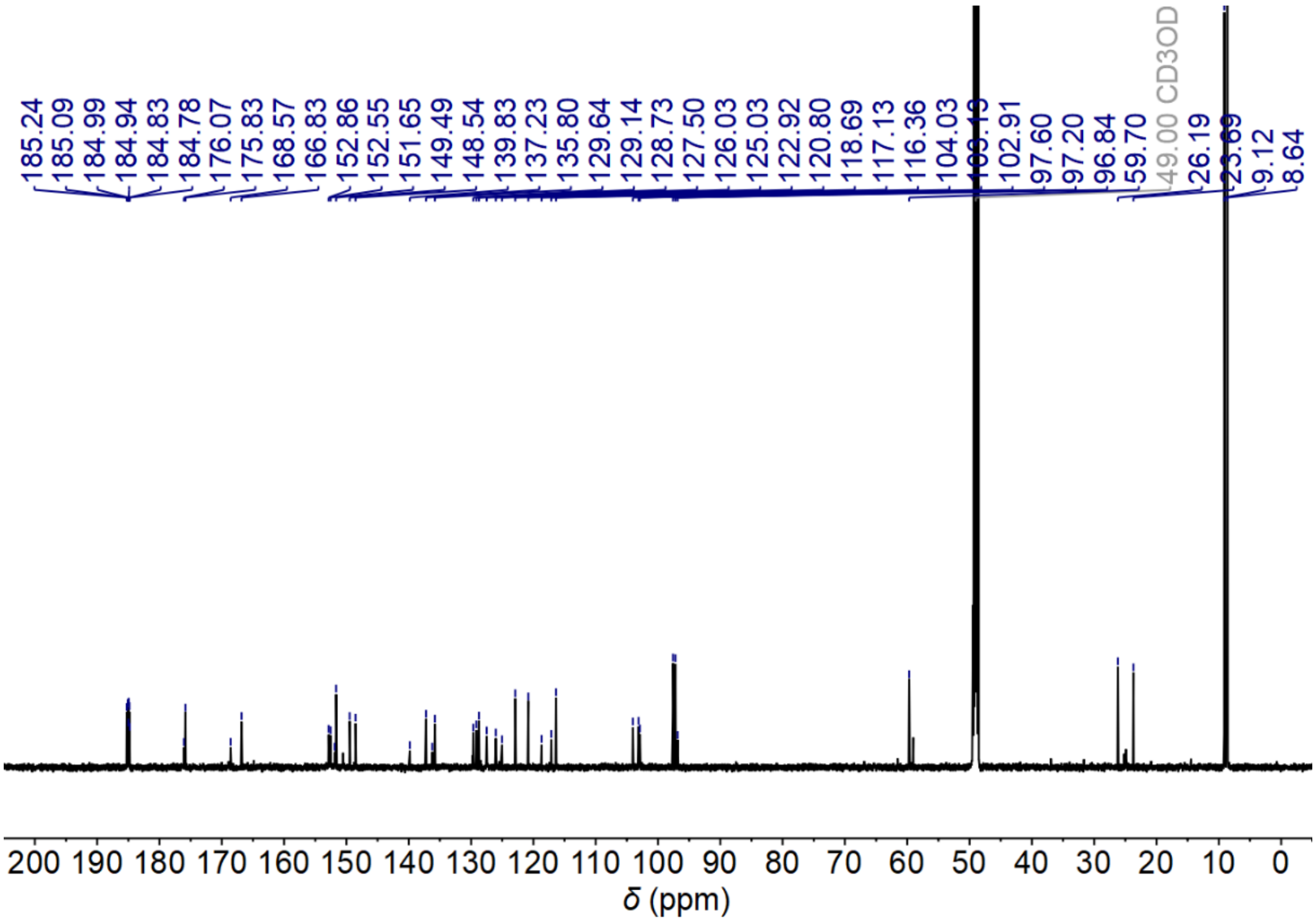

Fig. S148. $^{13}C\{^{1}H\}$ NMR spectrum of a solution prepared by dissolving crystalline material containing **Rh-5** and **Rh-5′** in CD_3OD (151 MHz, c_{Rh} = 64.0 mM).

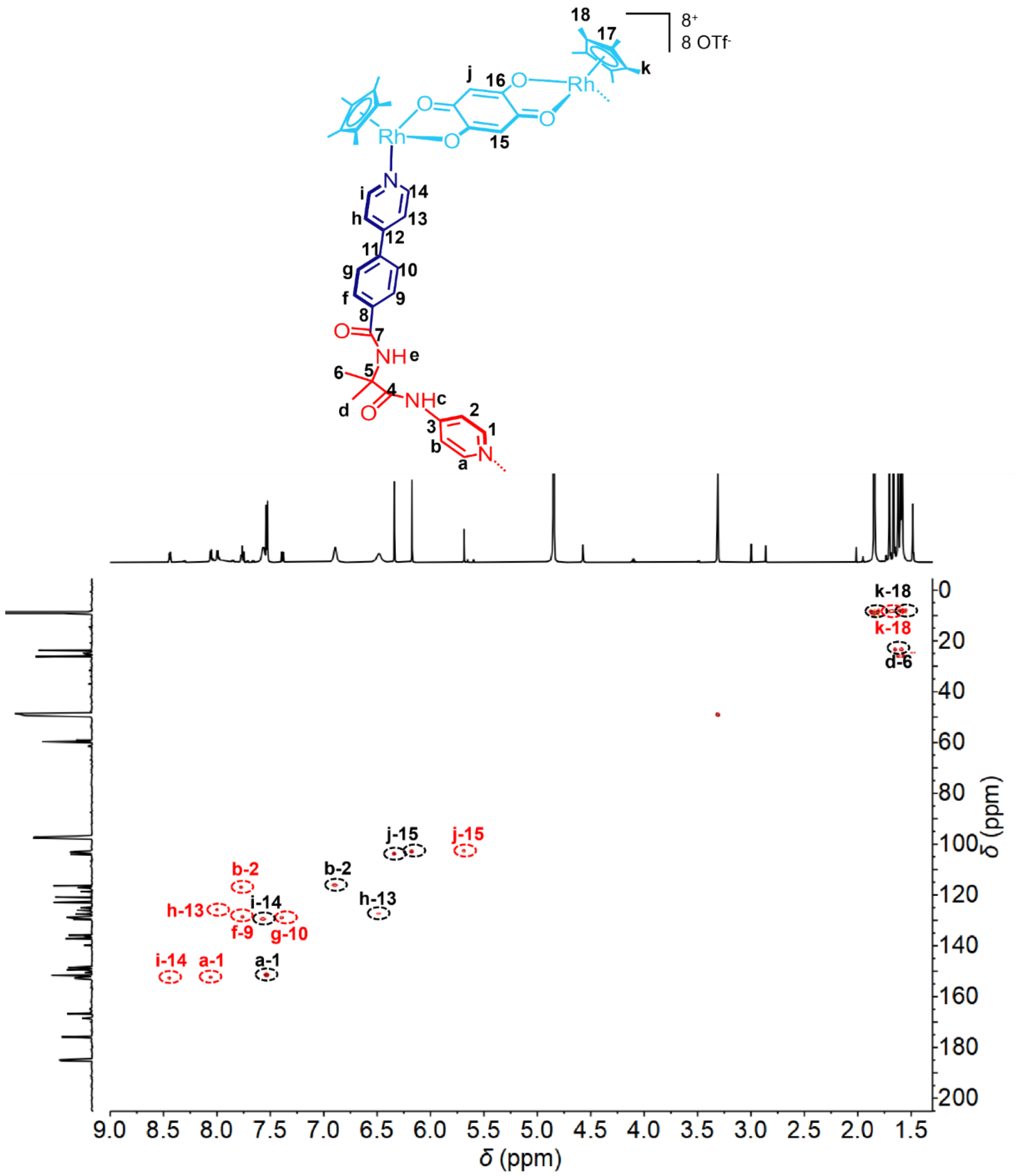

Fig. S149. ^1H–^{13}C HSQC spectrum of a solution prepared by dissolving crystalline material containing **Rh-5** and **Rh-5′** in CD_3OD (600 MHz, c_{Rh} = 64.0 mM). Black and red dashed ellipses highlight selected cross-peaks assigned to the figure-eight knot **Rh-5** and the tetranuclear metallomacrocycle **Rh-5′**, respectively.

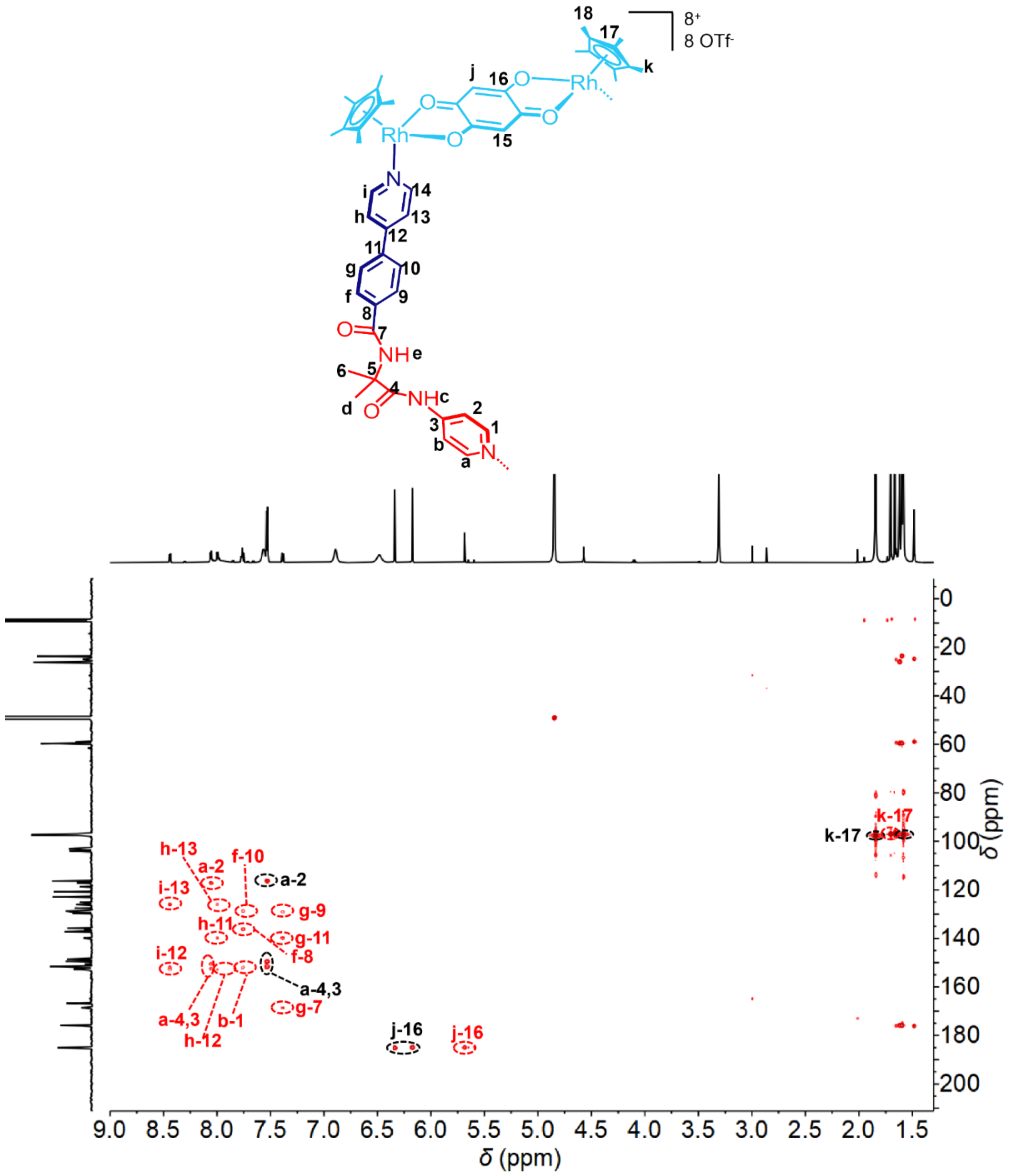

Fig. S150. ^1H–^{13}C HMBC spectrum of a solution prepared by dissolving crystalline material containing **Rh-5** and **Rh-5′** in CD_3OD (600 MHz, c_{Rh} = 64.0 mM). Black and red dashed ellipses highlight selected cross-peaks assigned to the figure-eight knot **Rh-5** and the tetranuclear metallomacrocycle **Rh-5′**, respectively.

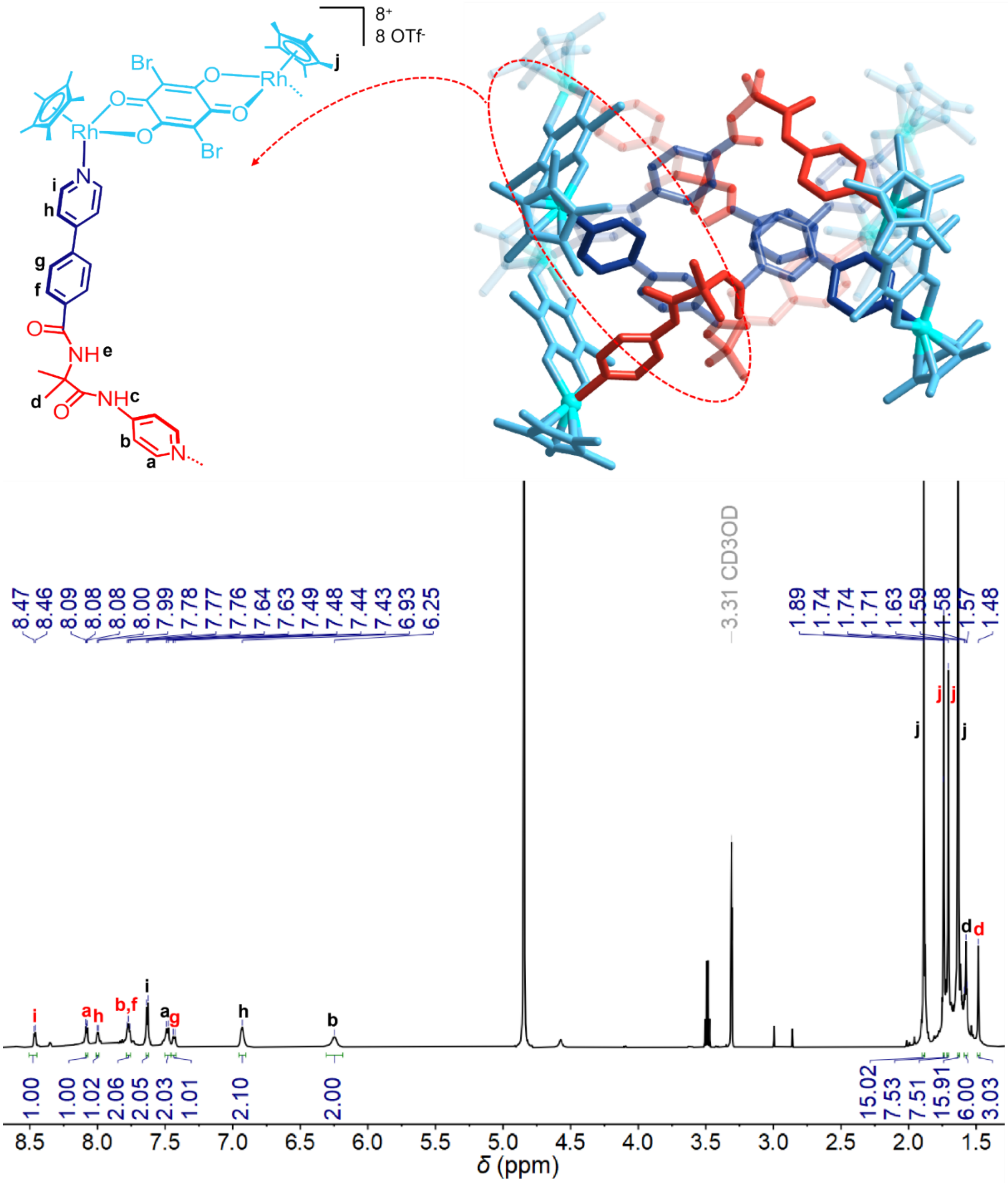

Fig. S151. ^{1}H NMR spectrum of a solution prepared by dissolving crystals of **Rh-5Br** in CD_3OD (600 MHz, c_{Rh} = 72.0 mM). Black and red letters label proton resonances assigned to the figure-eight knot **Rh-5Br** and a coexisting tetranuclear metallomacrocycle, respectively. Both components contribute substantially to the spectrum under these conditions. The hydrogen protons of NH on the ligand lose their NMR signal due to the exchange with D in CD_3OD. The resonances expected for four protons (2 H at f and 2 H at g) of the **Rh-5Br** are not clearly resolved, presumably because of shielding in the tightly entangled **Rh-5Br**.

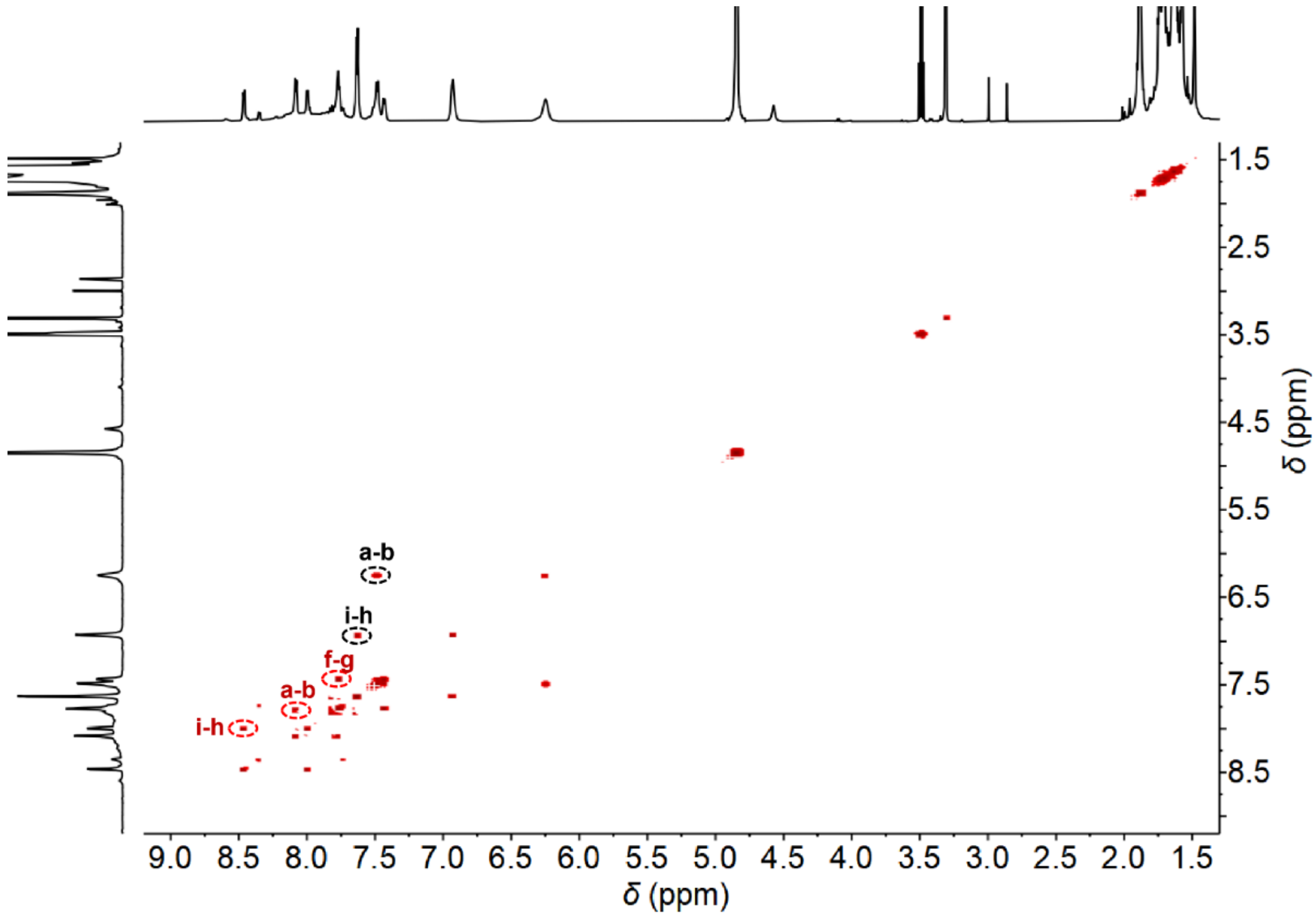

Fig. S152. ^{1}H-^{1}H COSY spectrum of a solution prepared by dissolving crystals of **Rh-5Br** in CD_3OD (600 MHz, c_{Rh} = 72.0 mM). Black and red dashed ellipses highlight selected cross-peaks assigned to the figure-eight knot **Rh-5Br** and a coexisting tetranuclear metallomacrocycle, respectively.

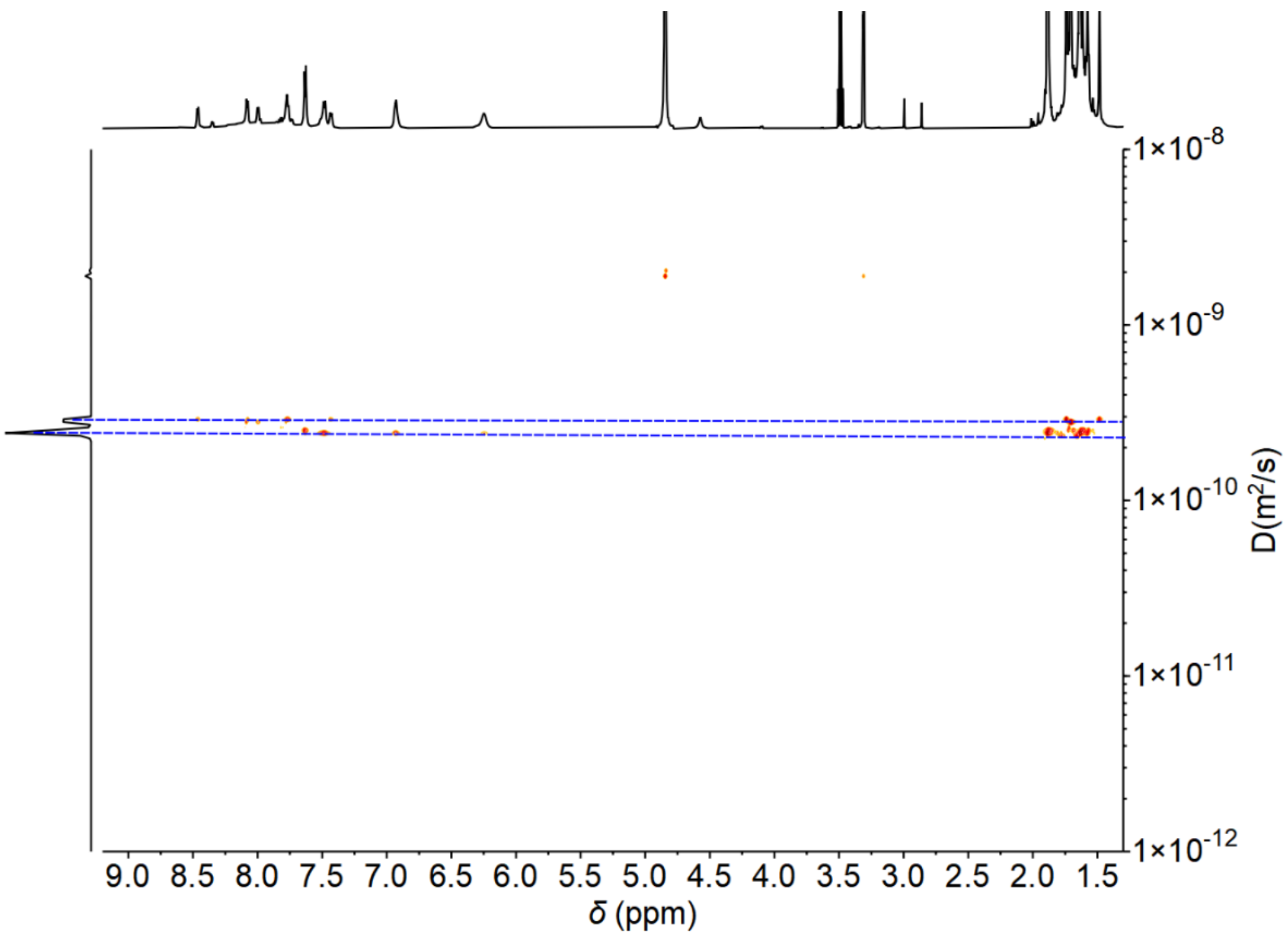

Fig. S153. ^{1}H DOSY spectrum of a solution prepared by dissolving crystals of **Rh-5Br** in CD_3OD (600 MHz, c_{Rh} = 72.0 mM). Two diffusion components are assigned to **Rh-5Br** (D = 2.41 × 10^{-10} m² s⁻¹) and the coexisting tetranuclear metallomacrocycle (D = 2.87 × 10^{-10} m² s⁻¹).

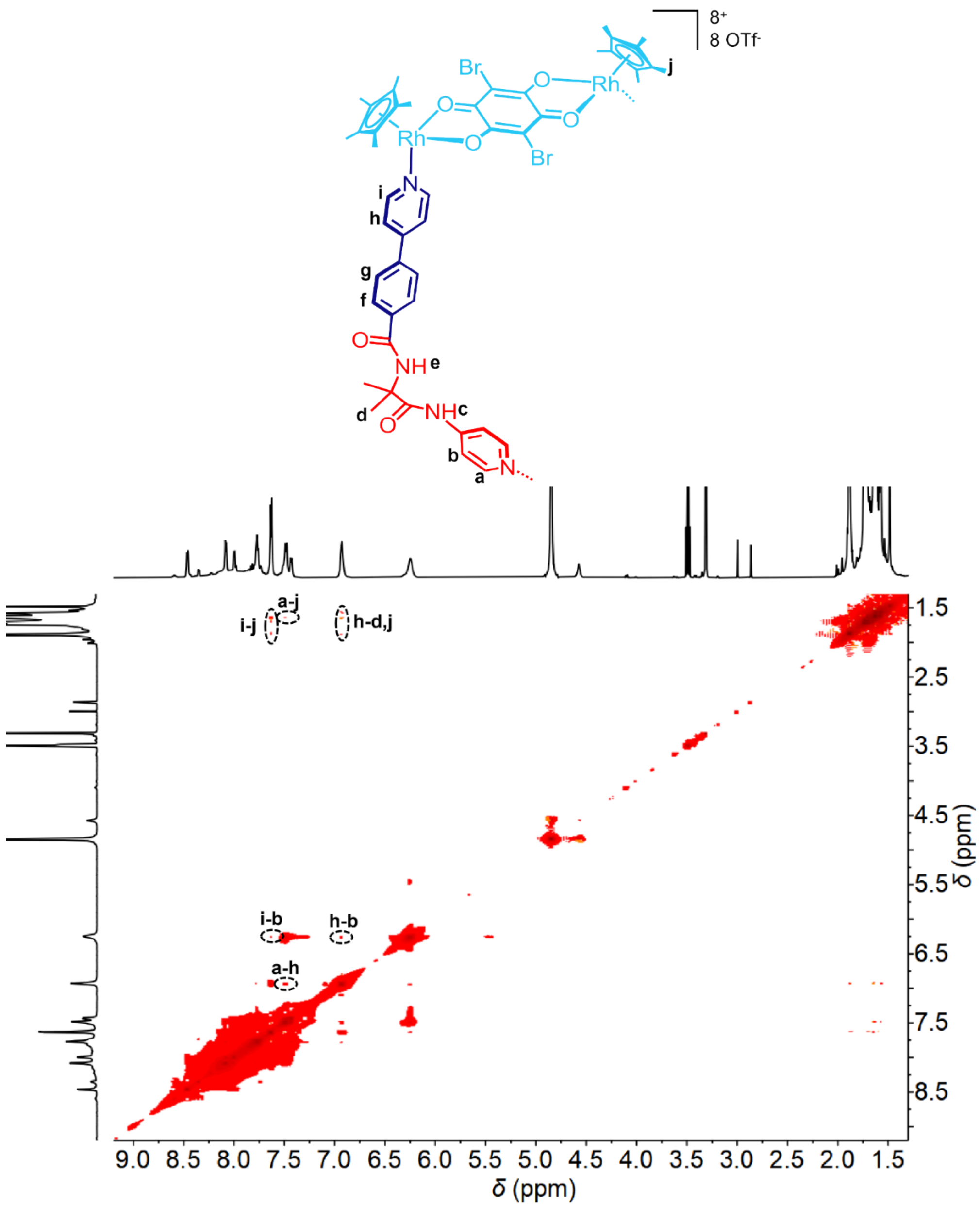

Fig. S154. ^{1}H-^{1}H NOESY spectrum of a solution prepared by dissolving crystals of **Rh-5Br** in CD_3OD (600 MHz, c_{Rh} = 72.0 mM). Black dashed ellipses highlight selected cross-peaks assigned to the figure-eight knot **Rh-5Br**.

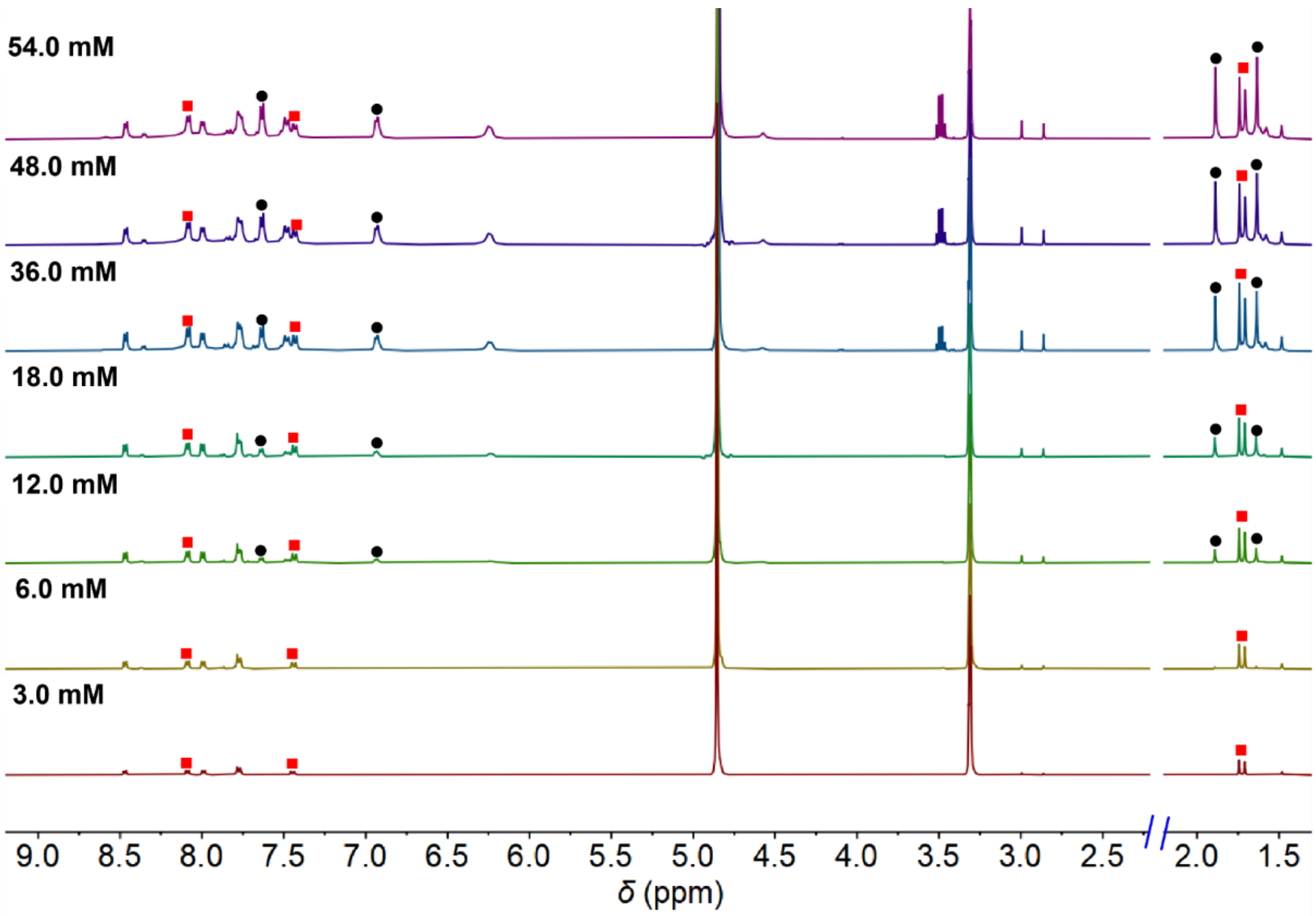

Fig. S155. Concentration-dependent ^{1}H NMR spectra of solutions prepared by dissolving crystals of **Rh-5Br** in CD_3OD (400 MHz, room temperature; c_{Rh} = 3.0–54.0 mM). Black circles mark selected resonances assigned to the figure-eight knot **Rh-5Br**, and red squares mark selected additional resonances assigned to the tetranuclear metallomacrocycle. Upon dilution, the figure-eight-knot resonances become less prominent relative to the additional resonances.

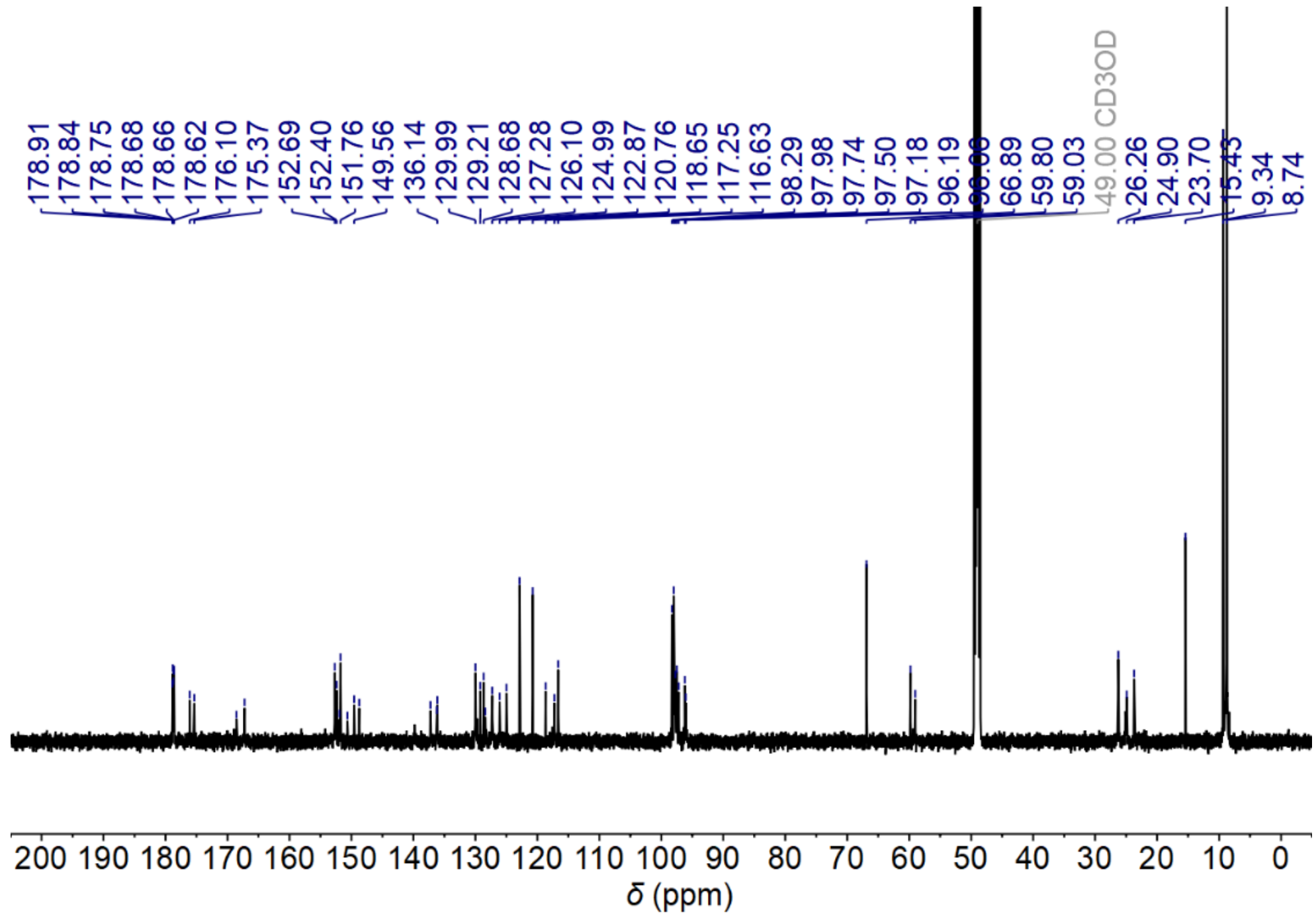

Fig. S156. $^{13}C\{^{1}H\}$ NMR spectrum of a solution prepared by dissolving crystals of **Rh-5Br** in CD_3OD (151 MHz, c_{Rh} = 72.0 mM).

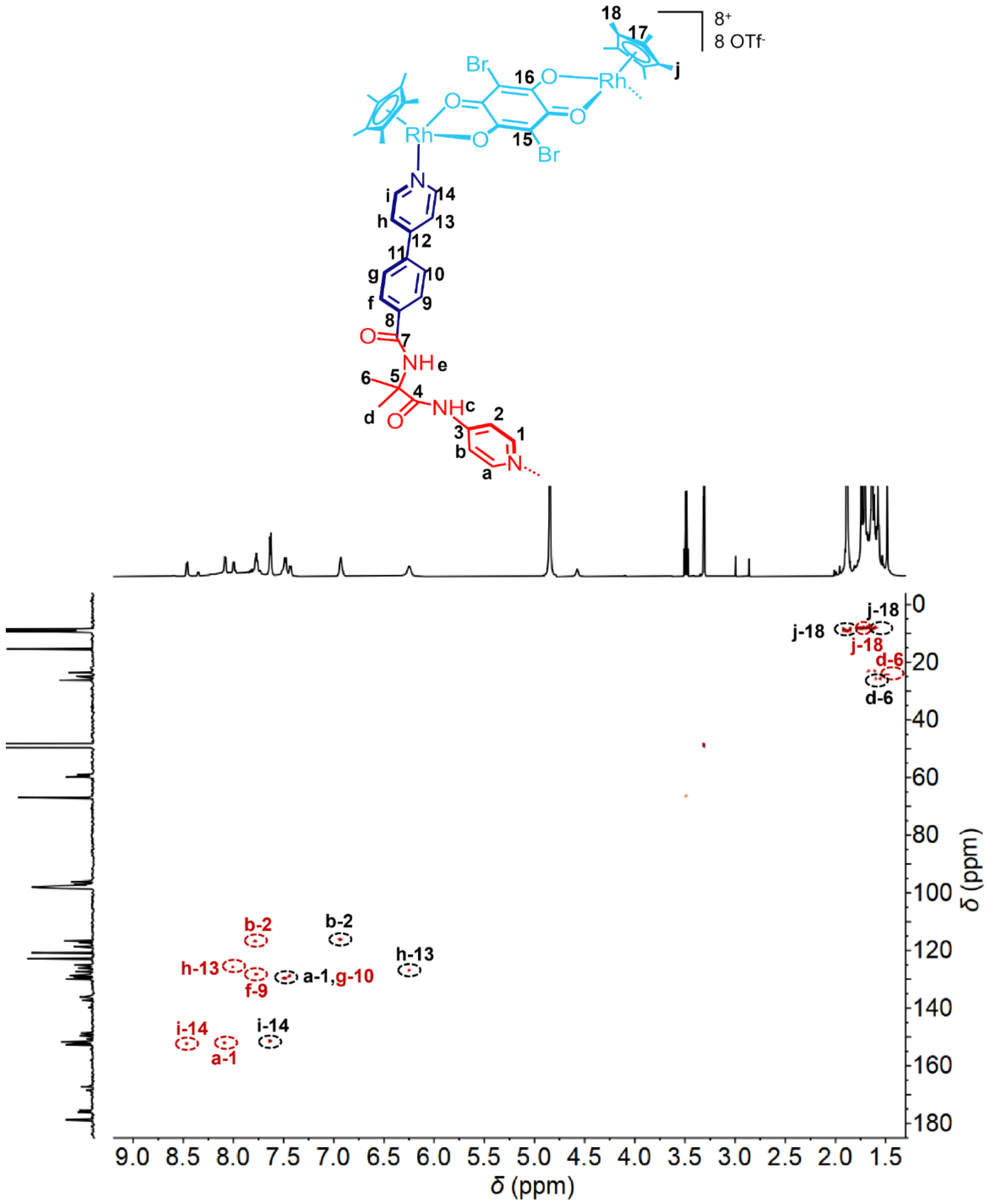

Fig. S157. ^{1}H–^{13}C HSQC spectrum of a solution prepared by dissolving crystals of **Rh-5Br** in CD_3OD (600 MHz, c_{Rh} = 72.0 mM). Black and red dashed ellipses highlight selected cross-peaks assigned to the figure-eight knot **Rh-5Br** and a coexisting tetranuclear metallomacrocycle, respectively.

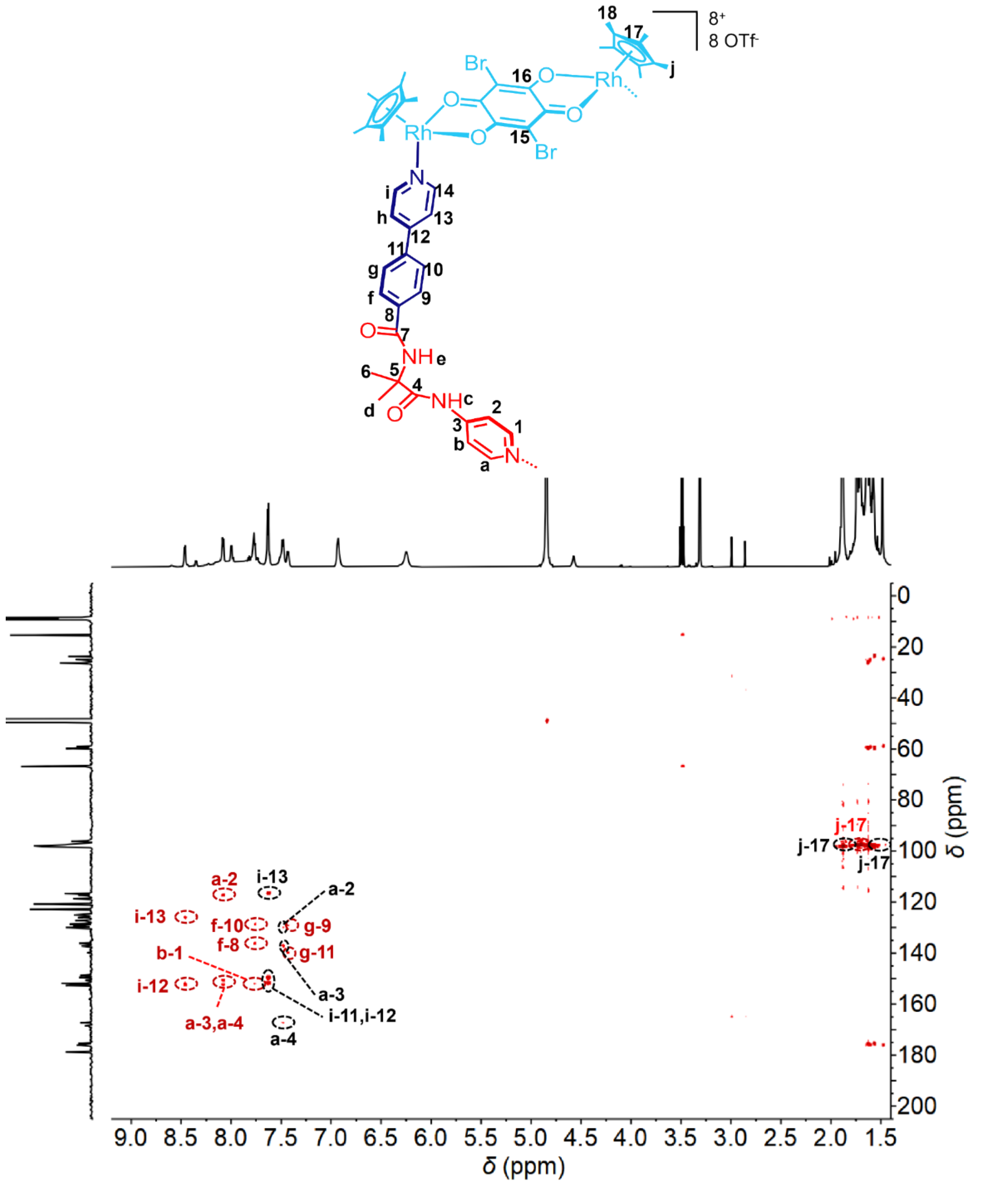

Fig. S158. ^{1}H–^{13}C HMBC spectrum of a solution prepared by dissolving crystals of **Rh-5Br** in CD_3OD (600 MHz, c_{Rh} = 72.0 mM). Black and red dashed ellipses highlight selected cross-peaks assigned to the figure-eight knot **Rh-5Br** and a coexisting tetranuclear metallomacrocycle, respectively.

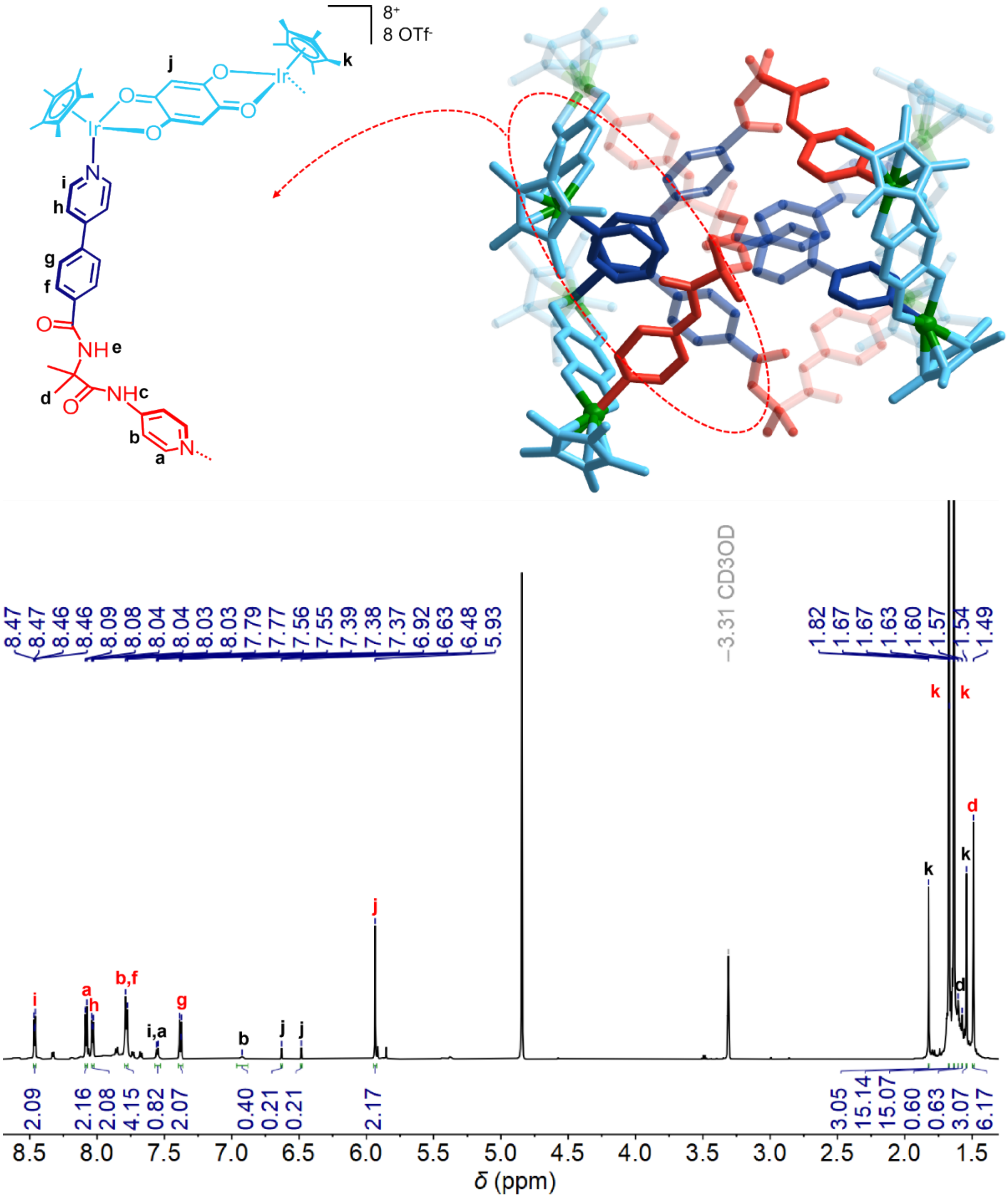

Fig. S159. ^{1}H NMR spectrum of a solution prepared by dissolving crystalline material containing **Ir-5** and **Ir-5'** in CD_3OD (600 MHz, c_{Ir} = 72.0 mM). Black and red letters label proton resonances assigned to the figure-eight knot **Ir-5** and the tetranuclear metallomacrocycle **Ir-5'**, respectively. The metallomacrocycle resonances are more prominent under these conditions. The hydrogen protons of NH on the ligand lose their NMR signal due to the exchange with D in CD_3OD. The resonances expected for six protons (2 H at f; 2 H at g and 2 H at h) of the **Ir-5** are not clearly resolved, presumably because of shielding in the tightly entangled **Ir-5**.

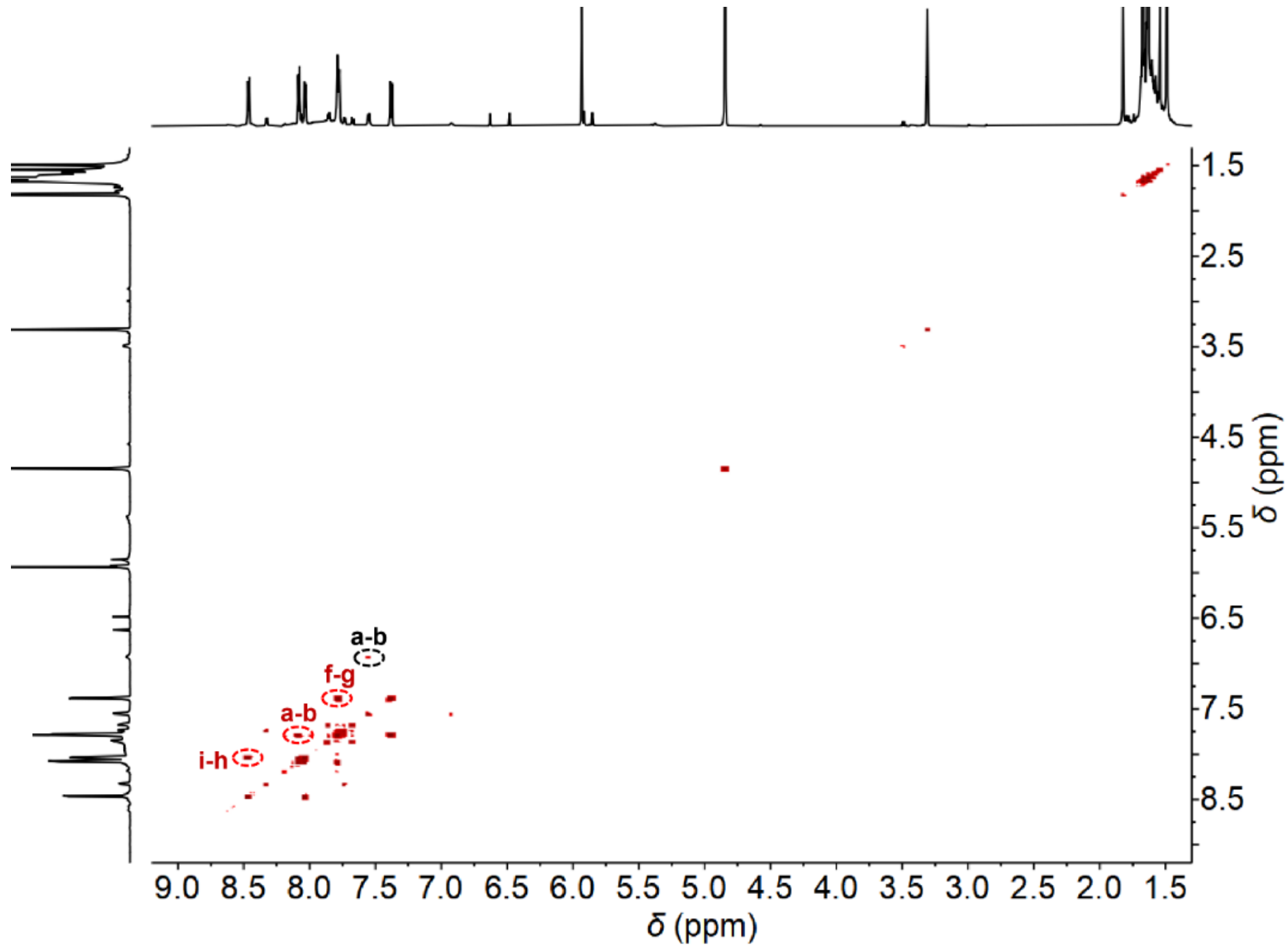

Fig. S160. ^{1}H-^{1}H COSY spectrum of a solution prepared by dissolving crystalline material containing **Ir-5** and **Ir-5′** in CD_3OD (600 MHz, c_{Ir} = 72.0 mM). Black and red dashed ellipses highlight selected cross-peaks assigned to the figure-eight knot **Ir-5** and the tetranuclear metallomacrocycle **Ir-5′**, respectively.

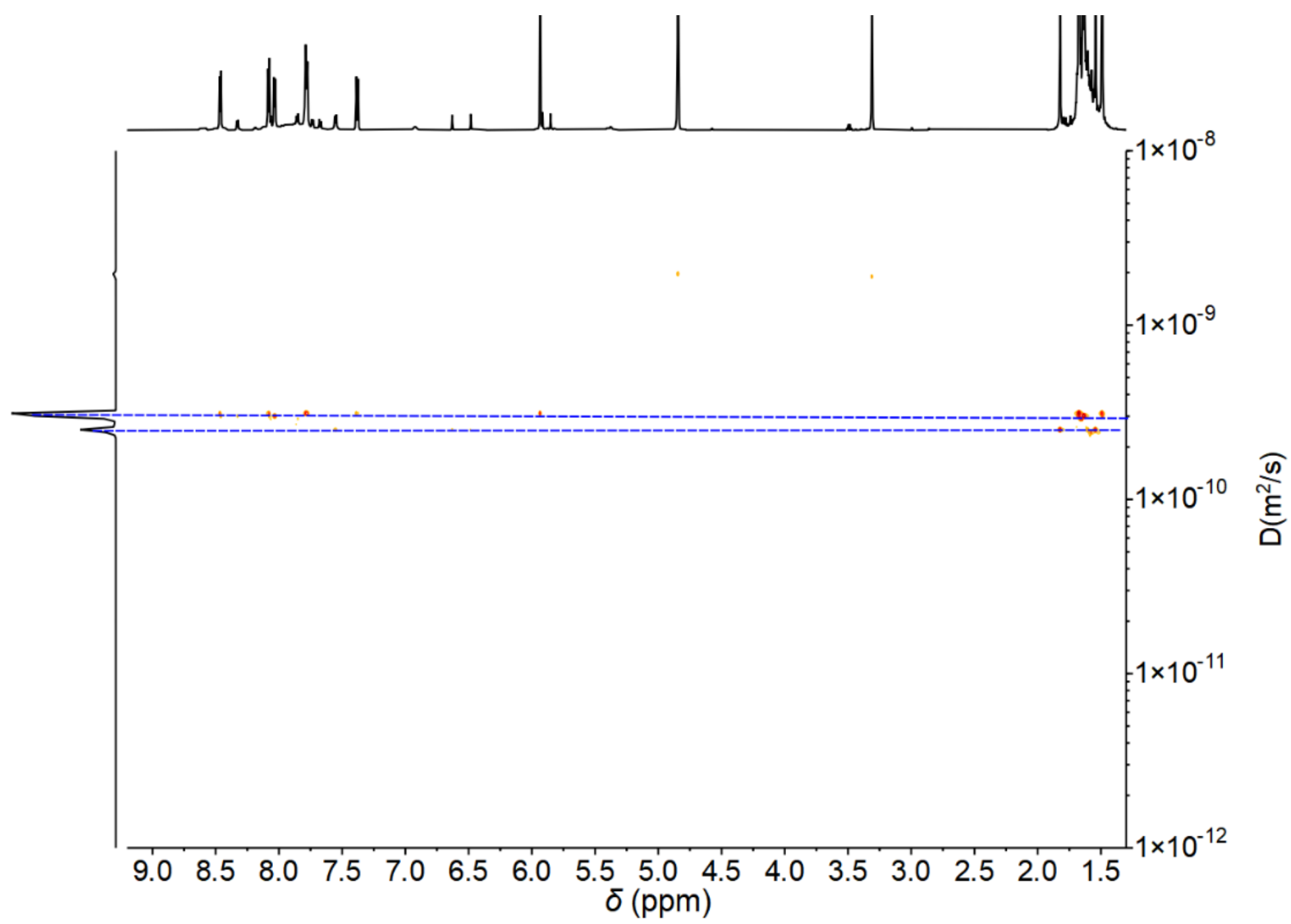

Fig. S161. ^{1}H DOSY spectrum of a solution prepared by dissolving crystalline material containing **Ir-5** and **Ir-5′** in CD_3OD (600 MHz, c_{Ir} = 72.0 mM). Two diffusion components are assigned to **Ir-5** ($D = 2.56 \times 10^{-10}$ m² s⁻¹) and **Ir-5′** ($D = 3.09 \times 10^{-10}$ m² s⁻¹).

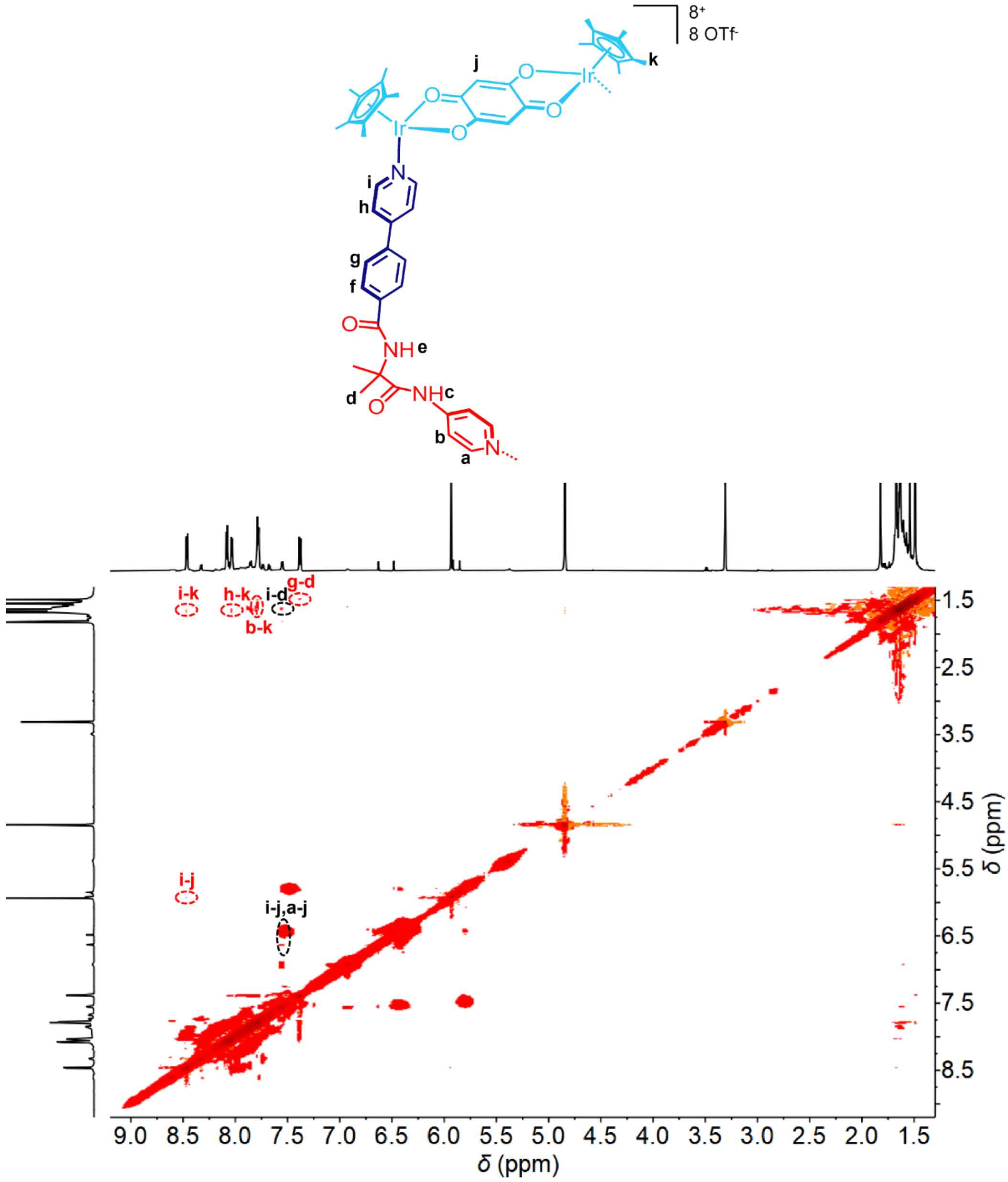

Fig. S162. ^{1}H-^{1}H NOESY spectrum of a solution prepared by dissolving crystalline material containing **Ir-5** and **Ir-5′** in CD_3OD (600 MHz, c_{Ir} = 72.0 mM). Black and red dashed ellipses highlight selected cross-peaks assigned to the figure-eight knot **Ir-5** and the tetranuclear metallomacrocycle **Ir-5′**, respectively.

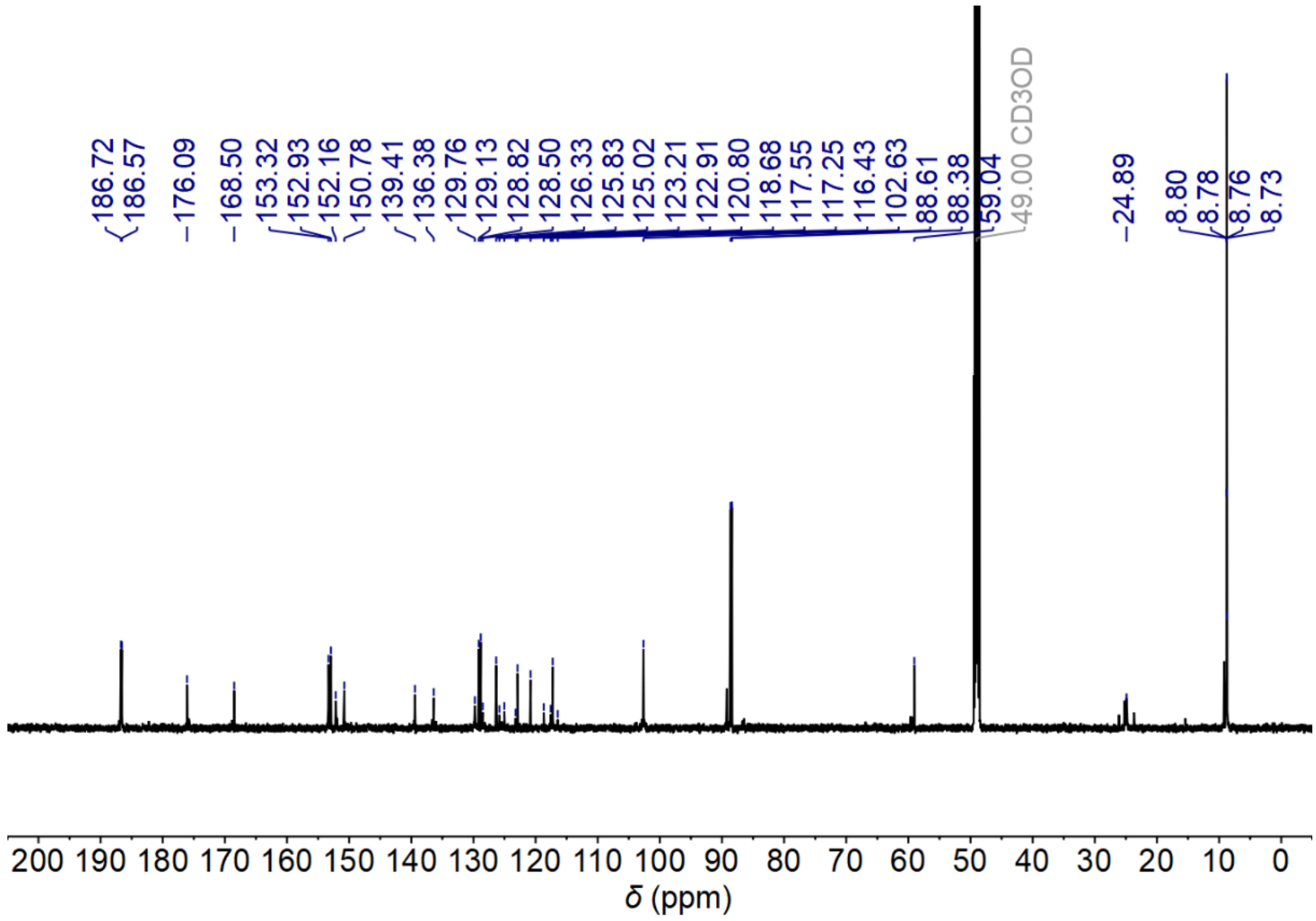

Fig. S163. $^{13}C\{^{1}H\}$ NMR spectrum of a solution prepared by dissolving crystalline material containing **Ir-5** and **Ir-5′** in CD_3OD (151 MHz, c_{Ir} = 72.0 mM).

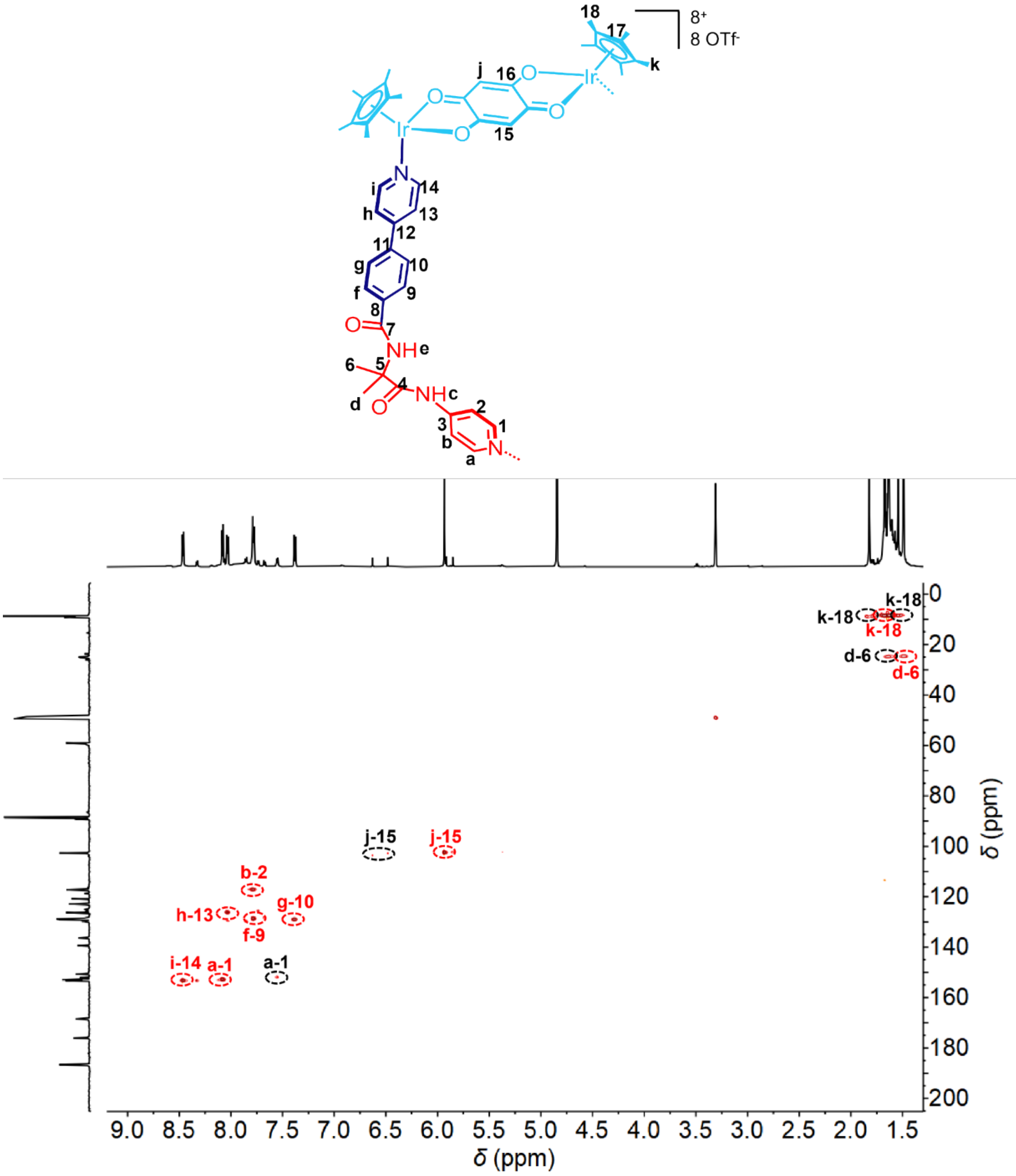

Fig. S164. ^1H–^{13}C HSQC spectrum of a solution prepared by dissolving crystalline material containing **Ir-5** and **Ir-5′** in CD_3OD (600 MHz, c_{Ir} = 72.0 mM). Black and red dashed ellipses highlight selected cross-peaks assigned to the figure-eight knot **Ir-5** and the tetranuclear metallomacrocycle **Ir-5′**, respectively.

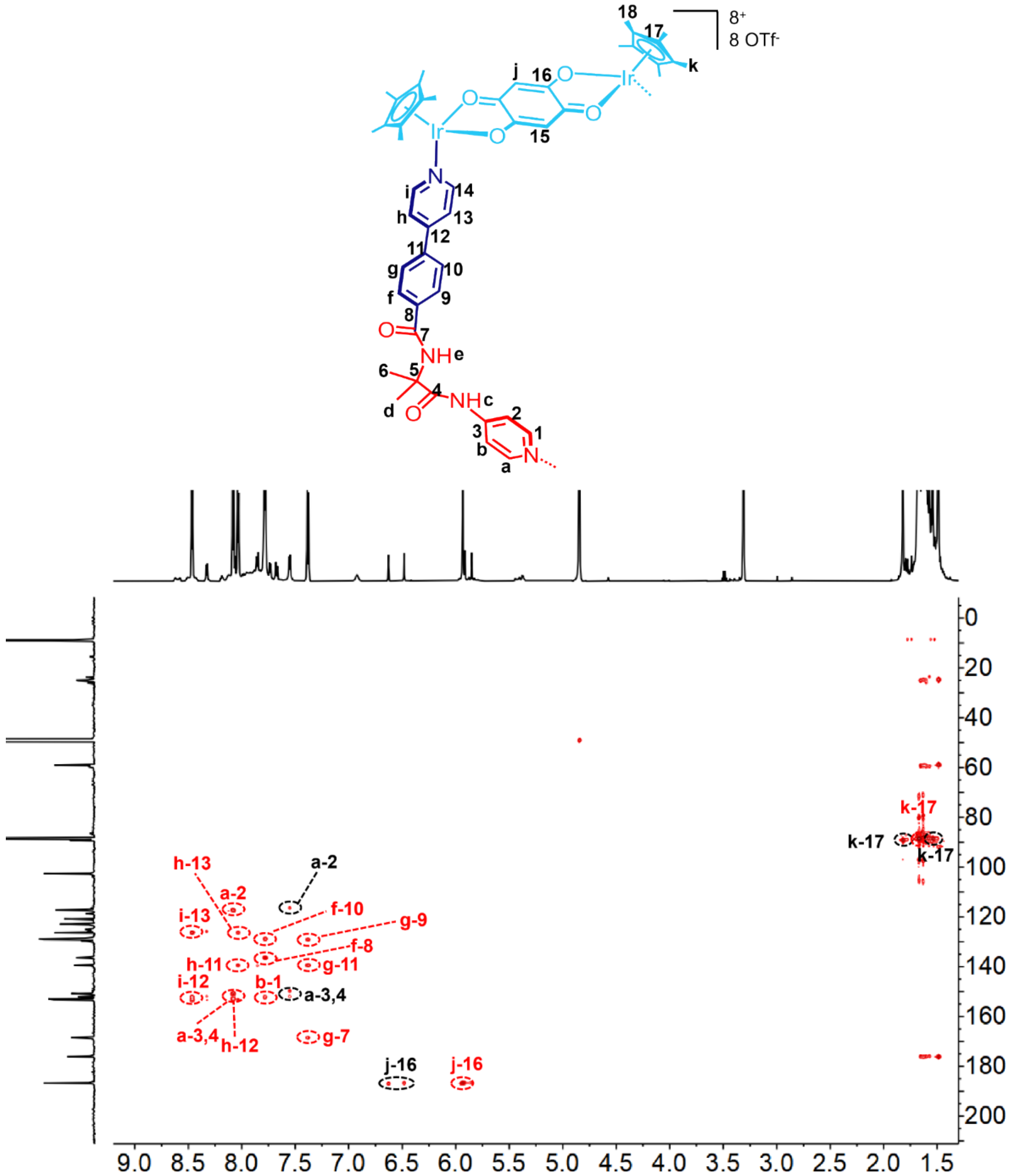

Fig. S165. ^{1}H–^{13}C HMBC spectrum of a solution prepared by dissolving crystalline material containing **Ir-5** and **Ir-5′** in CD_3OD (600 MHz, c_{Ir} = 72.0 mM). Black and red dashed ellipses highlight selected cross-peaks assigned to the figure-eight knot **Ir-5** and the tetranuclear metallomacrocycle **Ir-5′**, respectively.

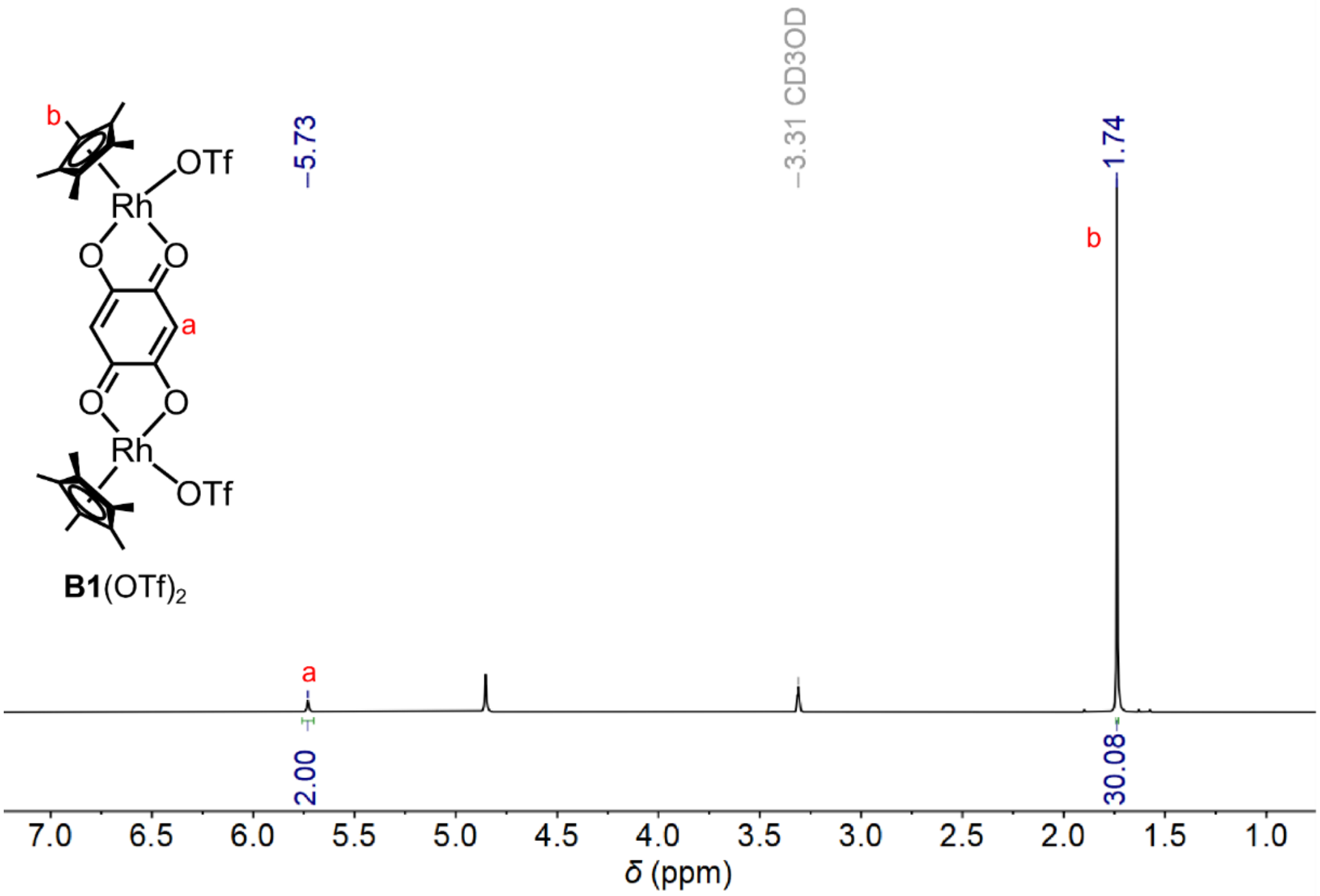

Fig. S166. ^{1}H NMR spectrum of **B1**(OTf)$_2$ in CD_3OD (400 MHz, c_{Rh} = 36.0 mM, corresponding to a binuclear-unit concentration of 18.0 mM).

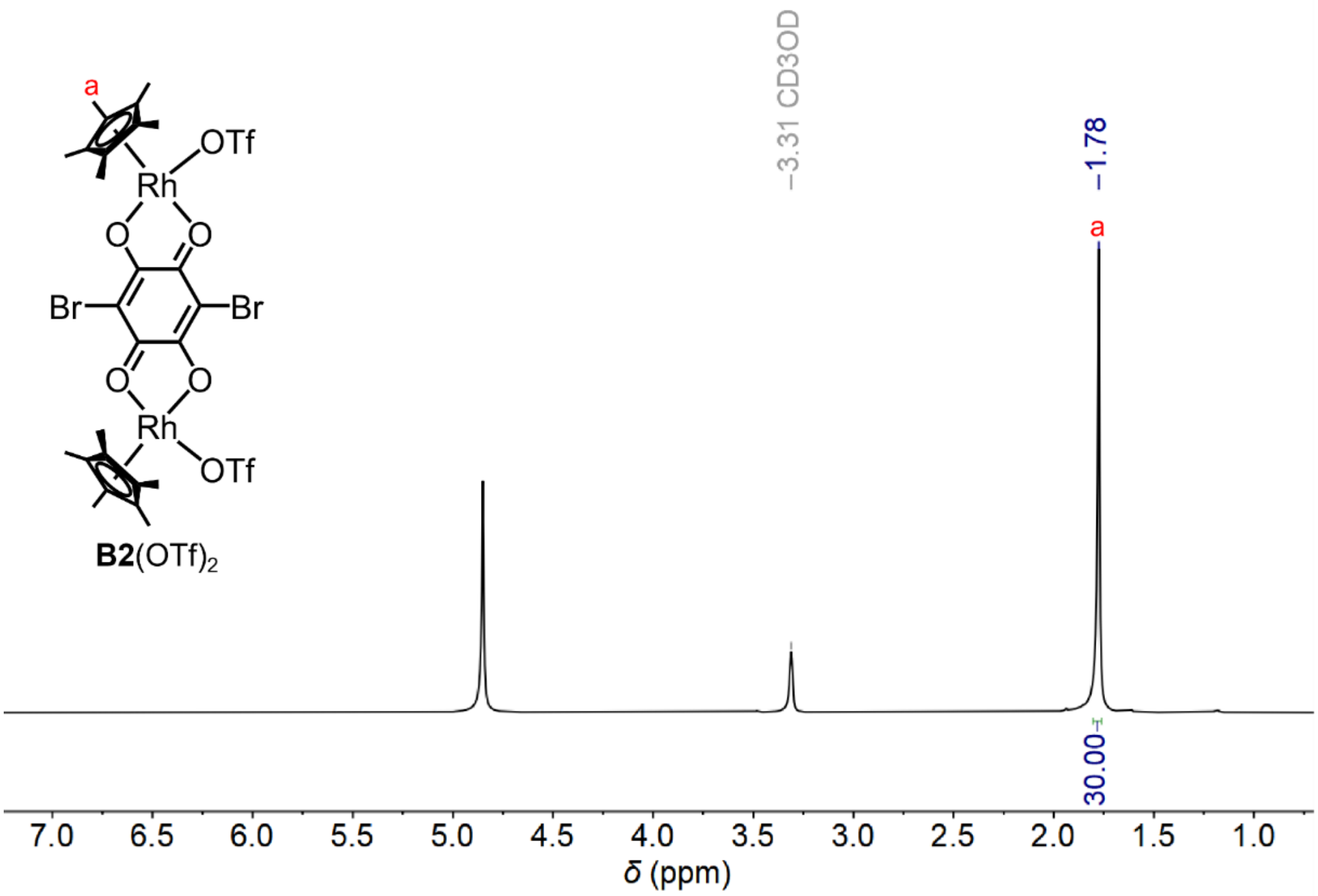

Fig. S167. ^{1}H NMR spectrum of **B2**(OTf)$_2$ in CD_3OD (400 MHz, c_{Rh} = 36.0 mM, corresponding to a binuclear-unit concentration of 18.0 mM).

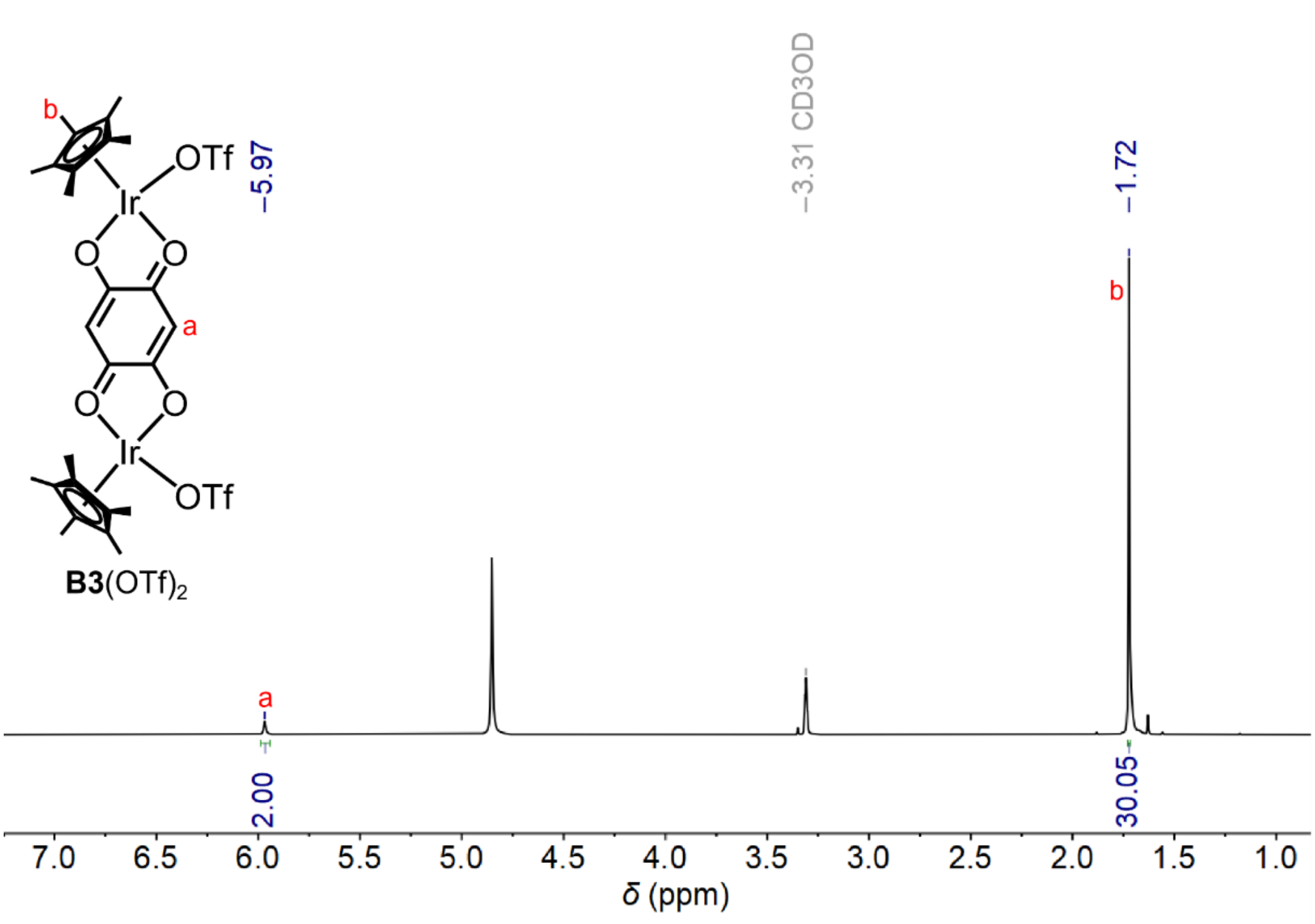

Fig. S168. ^{1}H NMR spectrum of **B3**(OTf)$_2$ in CD_3OD (400 MHz, c_{Ir} = 36.0 mM, corresponding to a binuclear-unit concentration of 18.0 mM).

7. ESI-MS Spectra

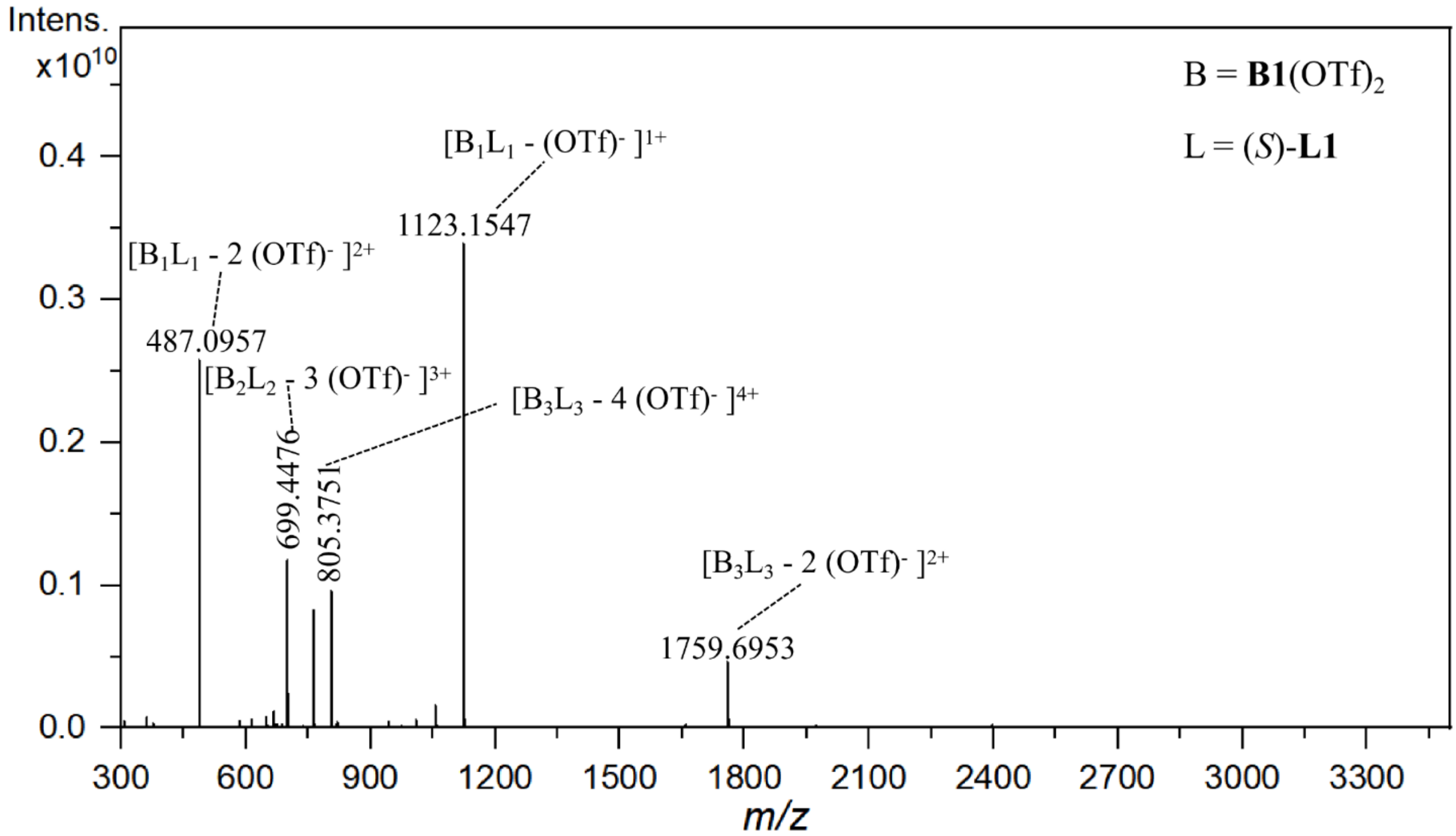

Fig. S169. Full-range ESI-MS spectrum of Λ-**Rh-1** in methanol.

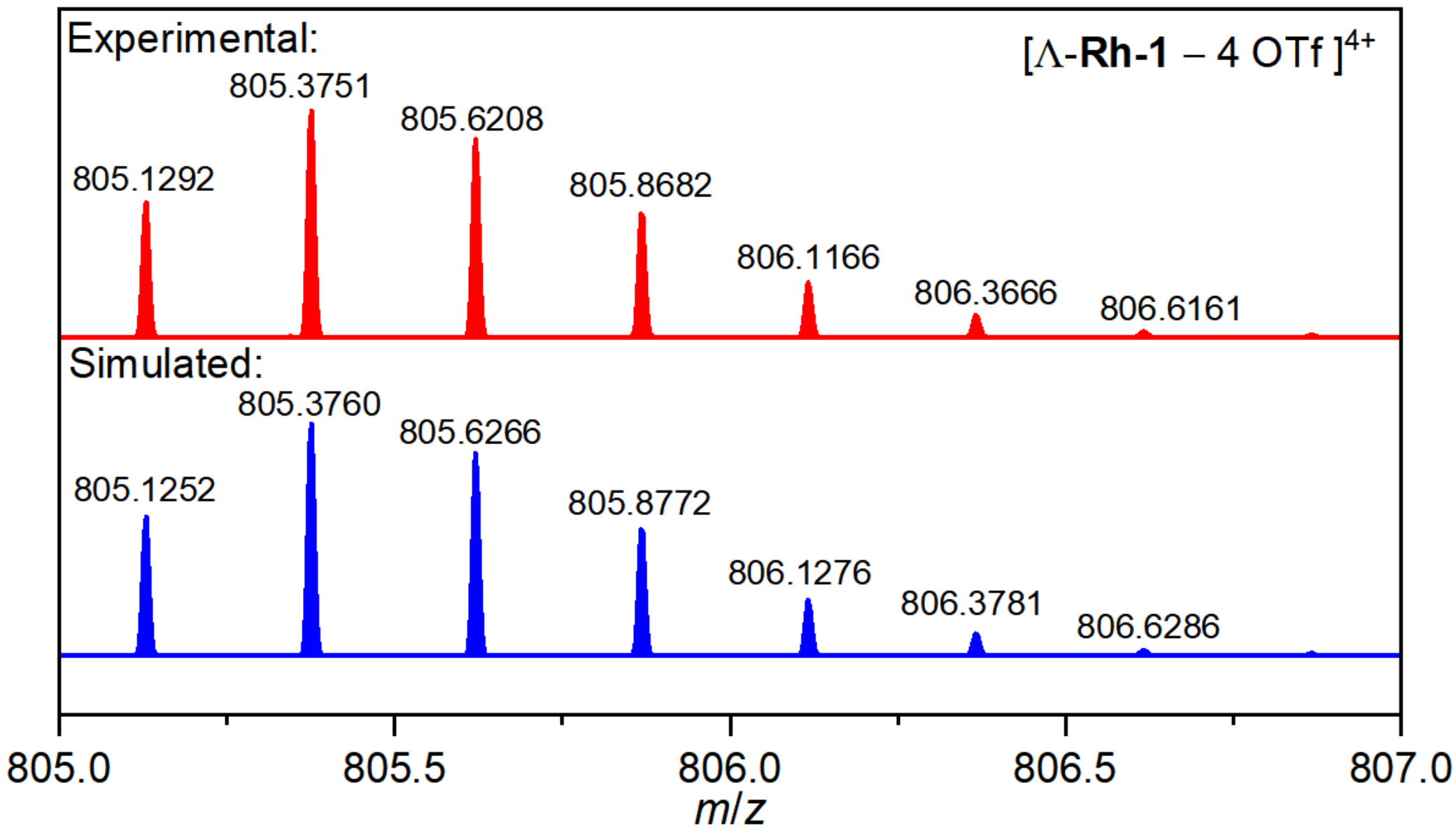

Fig. S170. Experimental and calculated isotope patterns of [Λ-**Rh-1** – 4OTf]$^{4+}$.

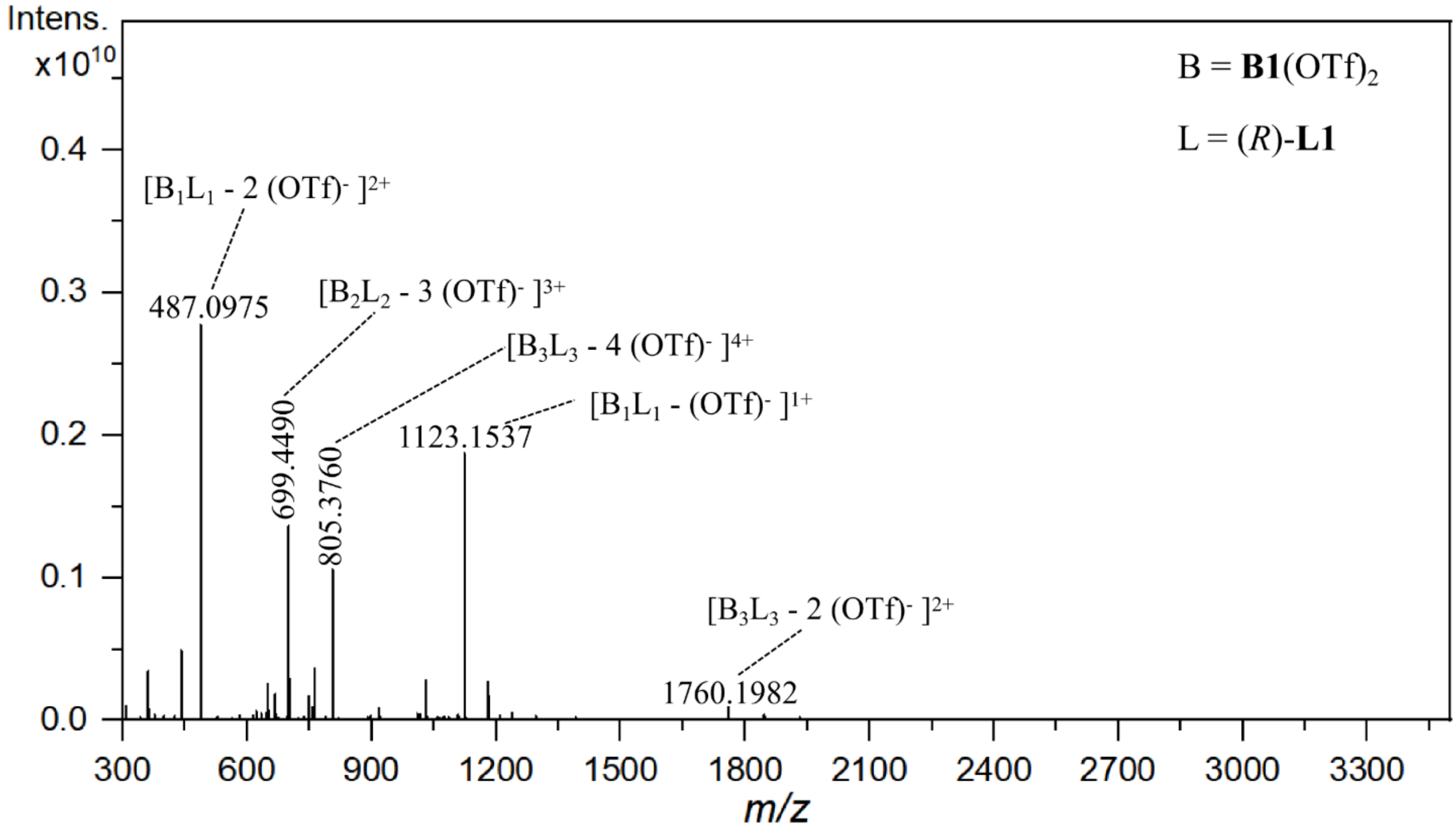

Fig. S171. Full-range ESI-MS spectrum of Δ-**Rh-1** in methanol.

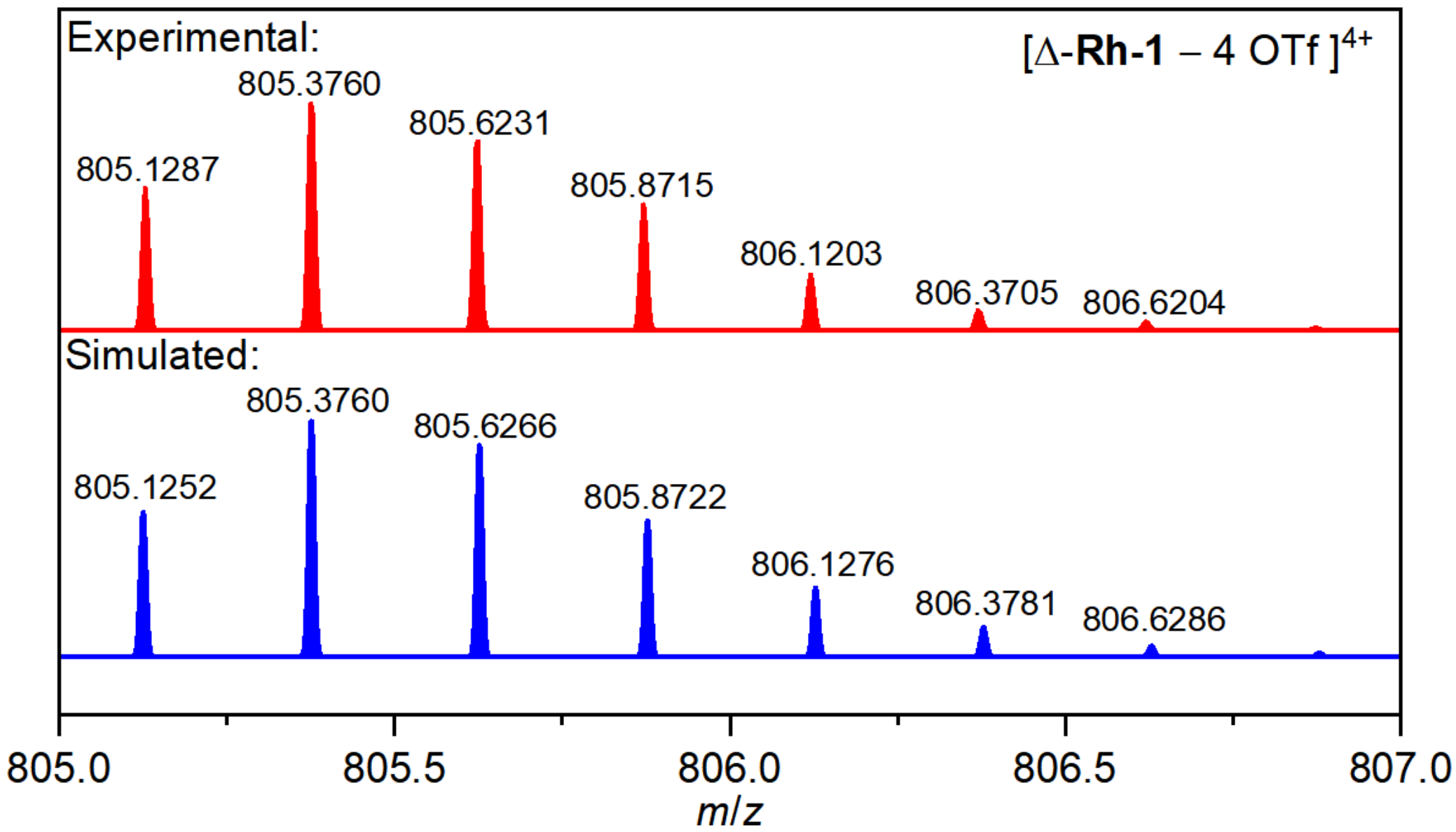

Fig. S172. Experimental and calculated isotope patterns of [Δ-**Rh-1** – 4OTf]$^{4+}$.

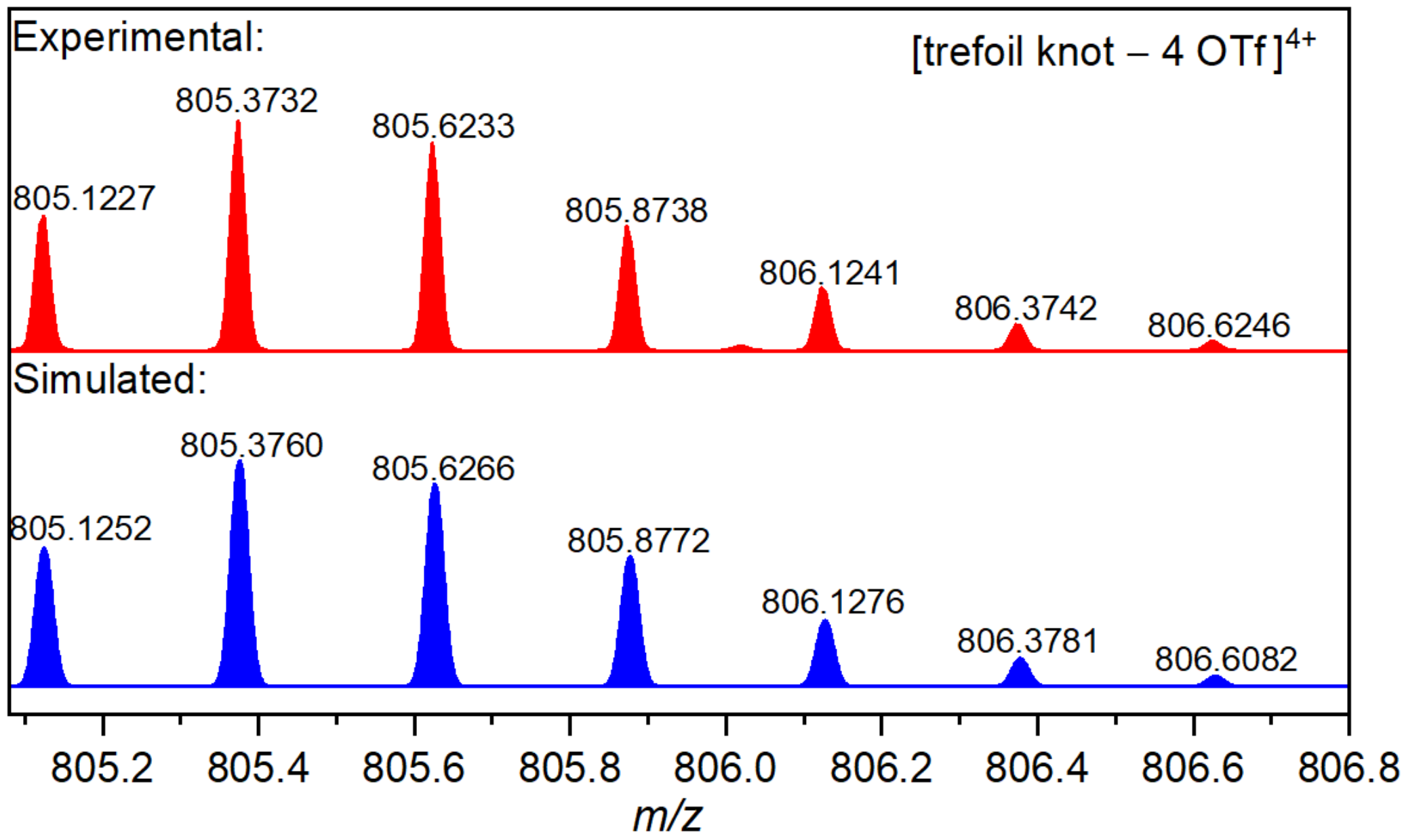

Fig. S173. Experimental and calculated isotope patterns of the quadruply charged ion [trefoil knot – 4 OTf]$^{4+}$ observed for the racemic mixture of Λ-**Rh-1** and Δ-**Rh-1** obtained by self-assembly of equimolar (*S*)-**L1** and (*R*)-**L1** with **B1**$(OTf)_2$. Here, "trefoil knot" denotes the corresponding salt containing six OTf counterions.

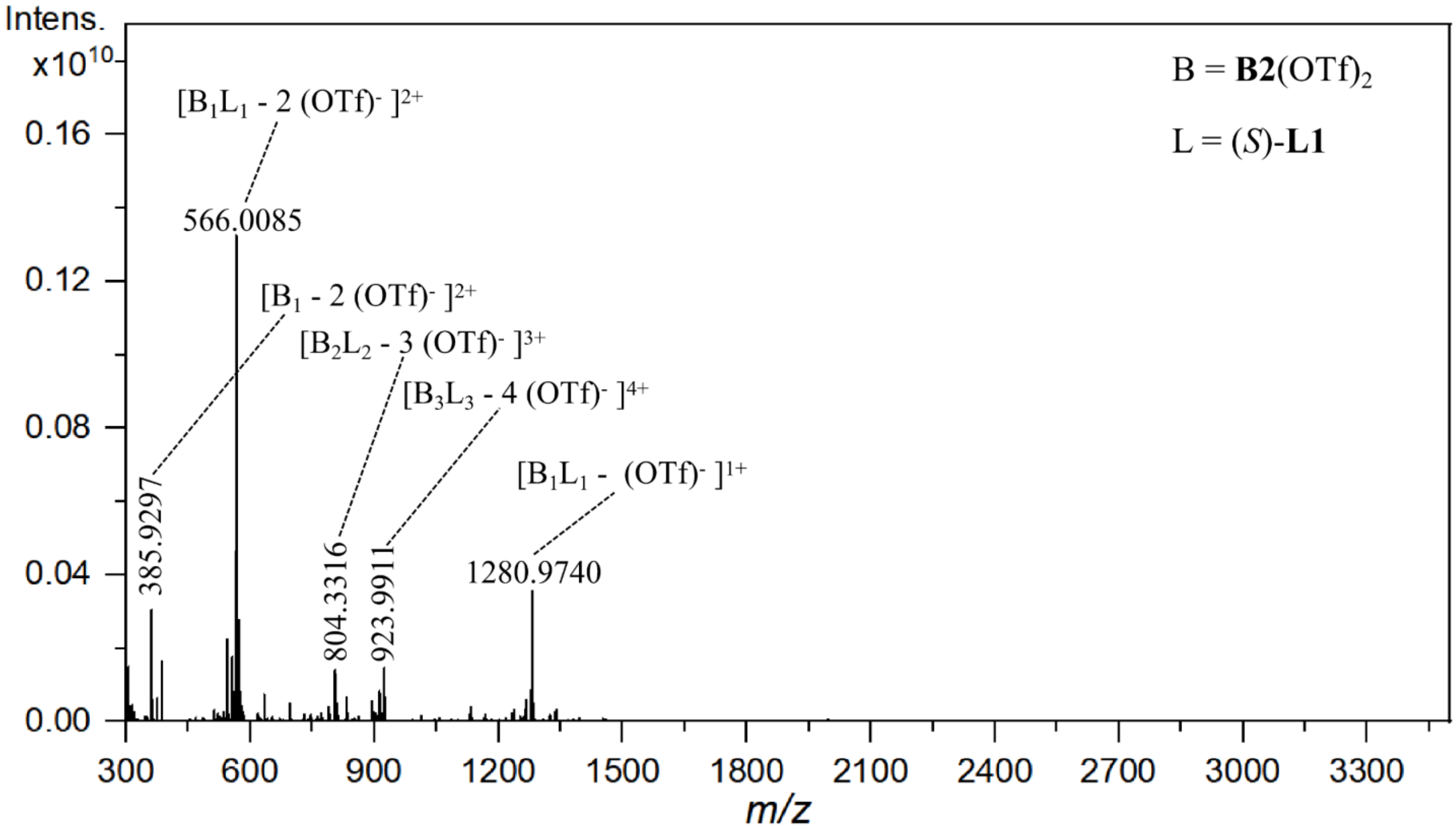

Fig. S174. Full-range ESI-MS spectrum of Λ-**Rh-1Br** in methanol.

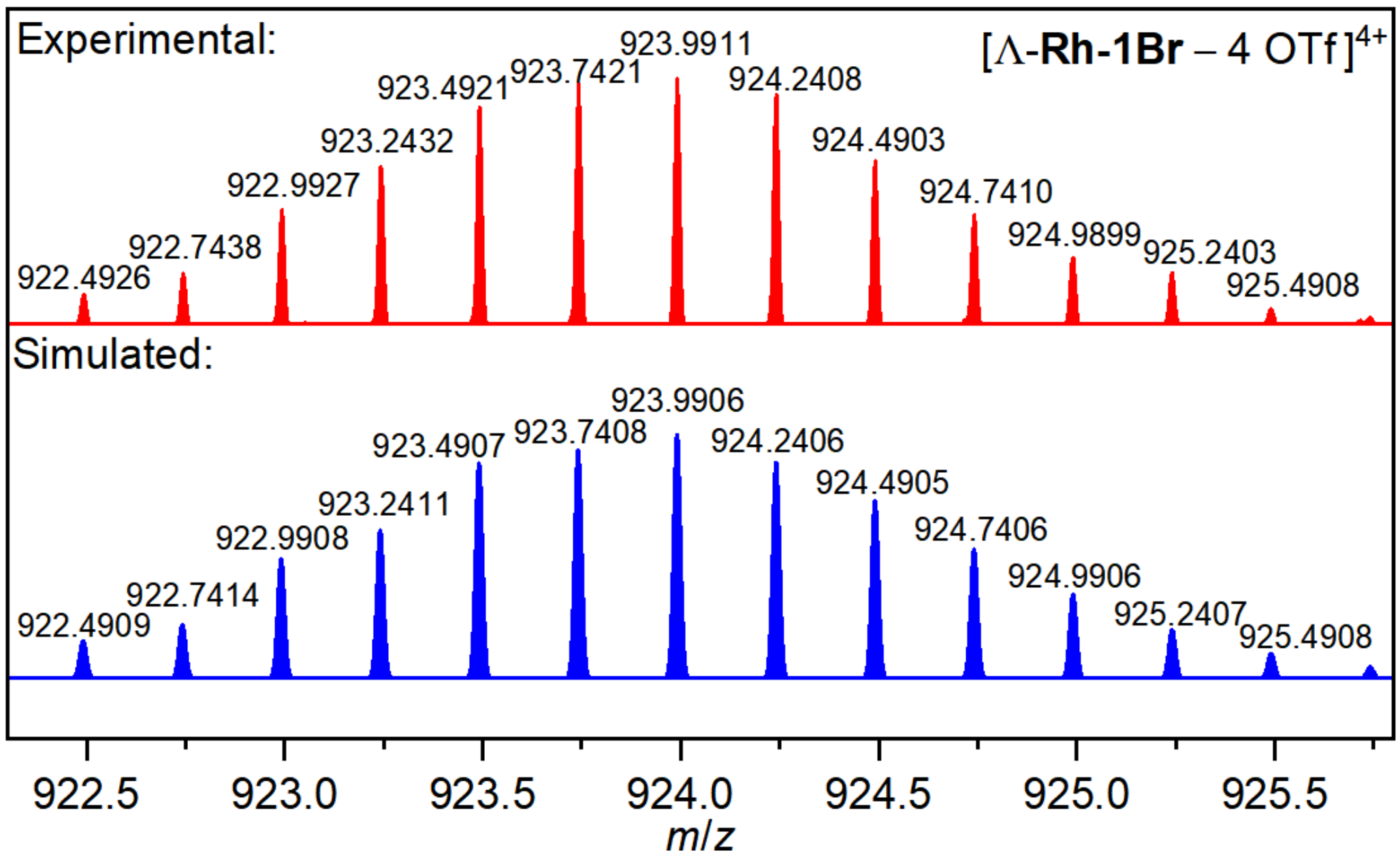

Fig. S175. Experimental and calculated isotope patterns of [Λ-**Rh-1Br** – 4OTf]$^{4+}$.

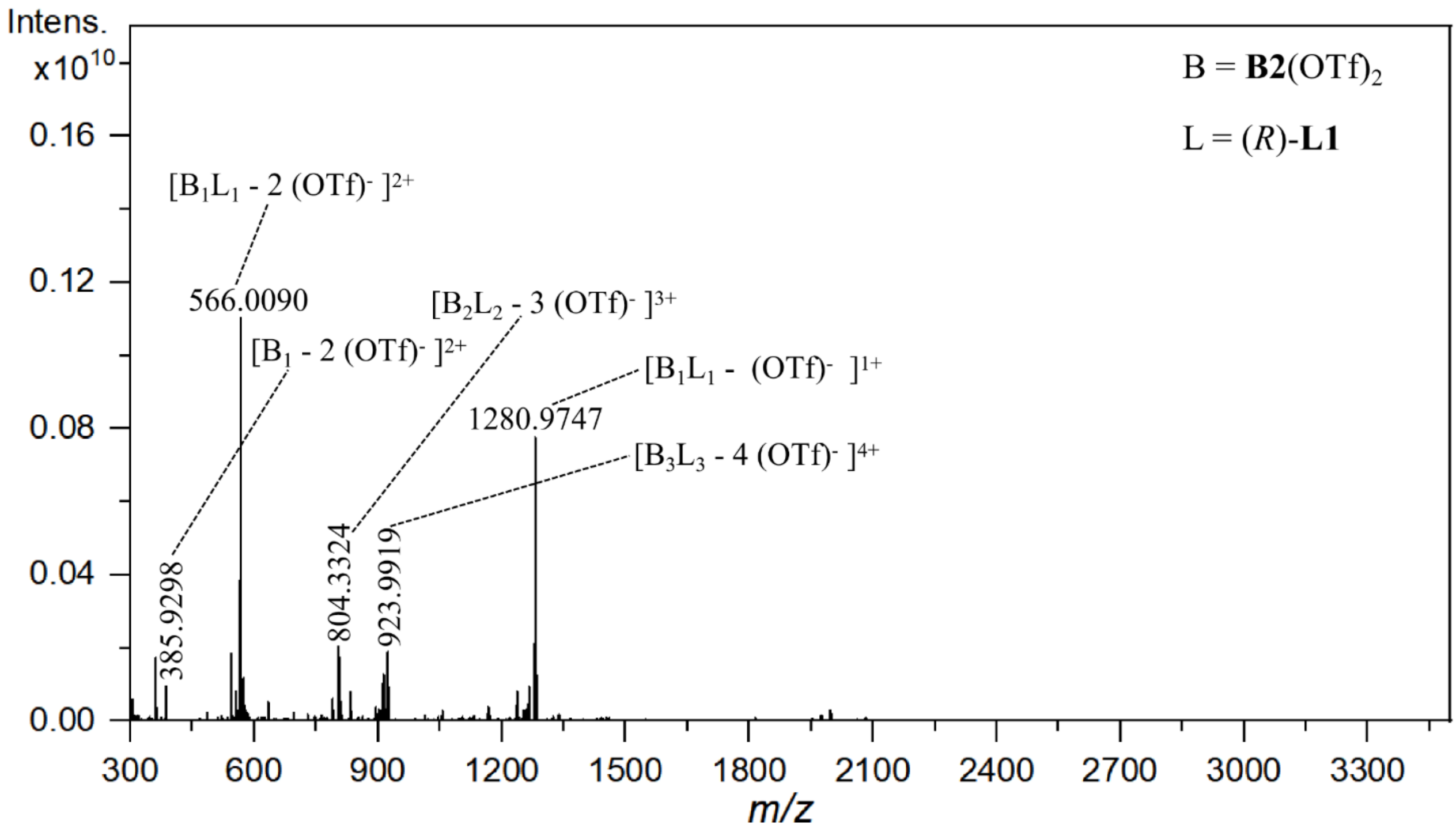

Fig. S176. Full-range ESI-MS spectrum of Δ-**Rh-1Br** in methanol.

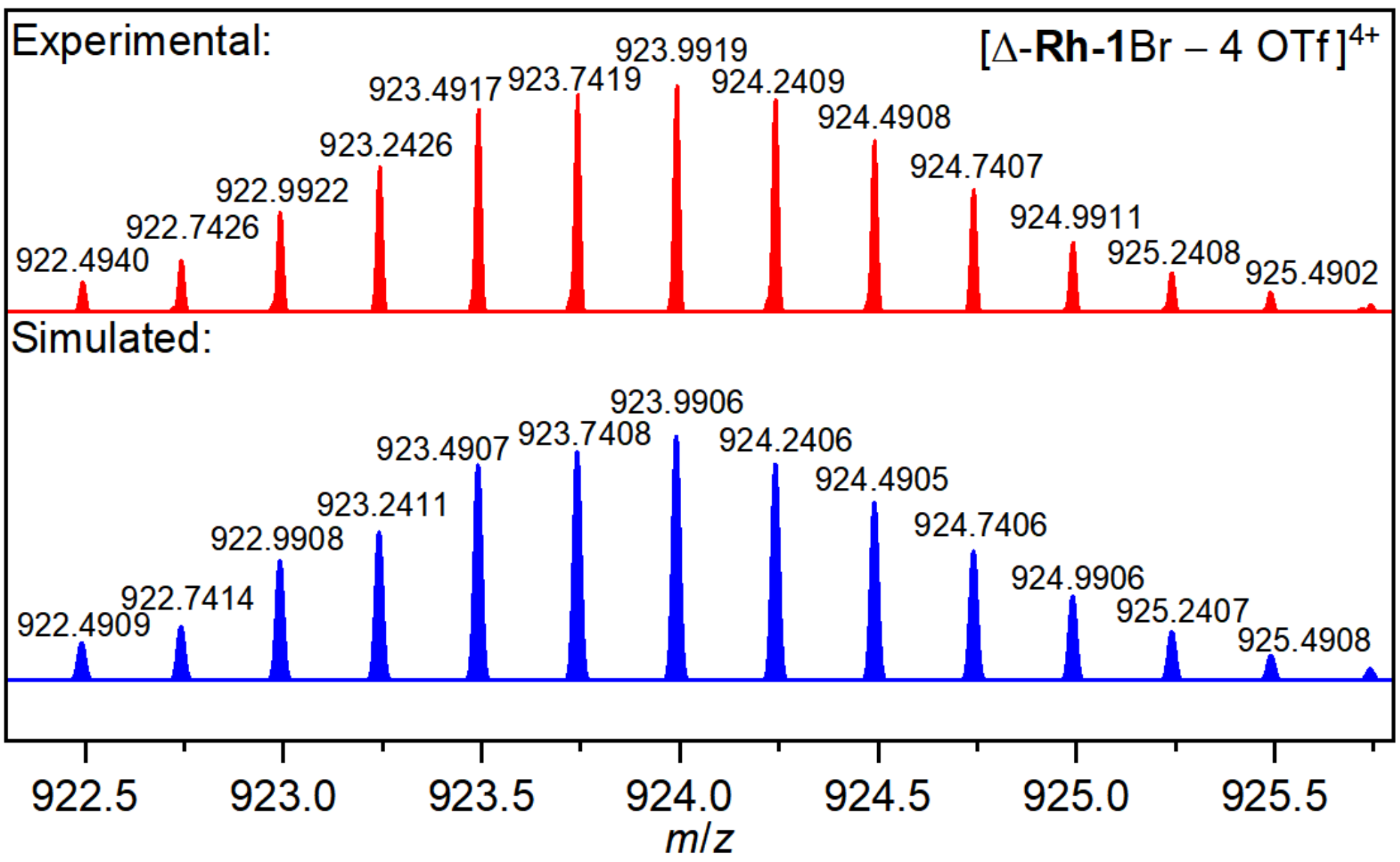

Fig. S177. Experimental and calculated isotope patterns of [Δ-**Rh-1Br** – 4OTf]$^{4+}$.

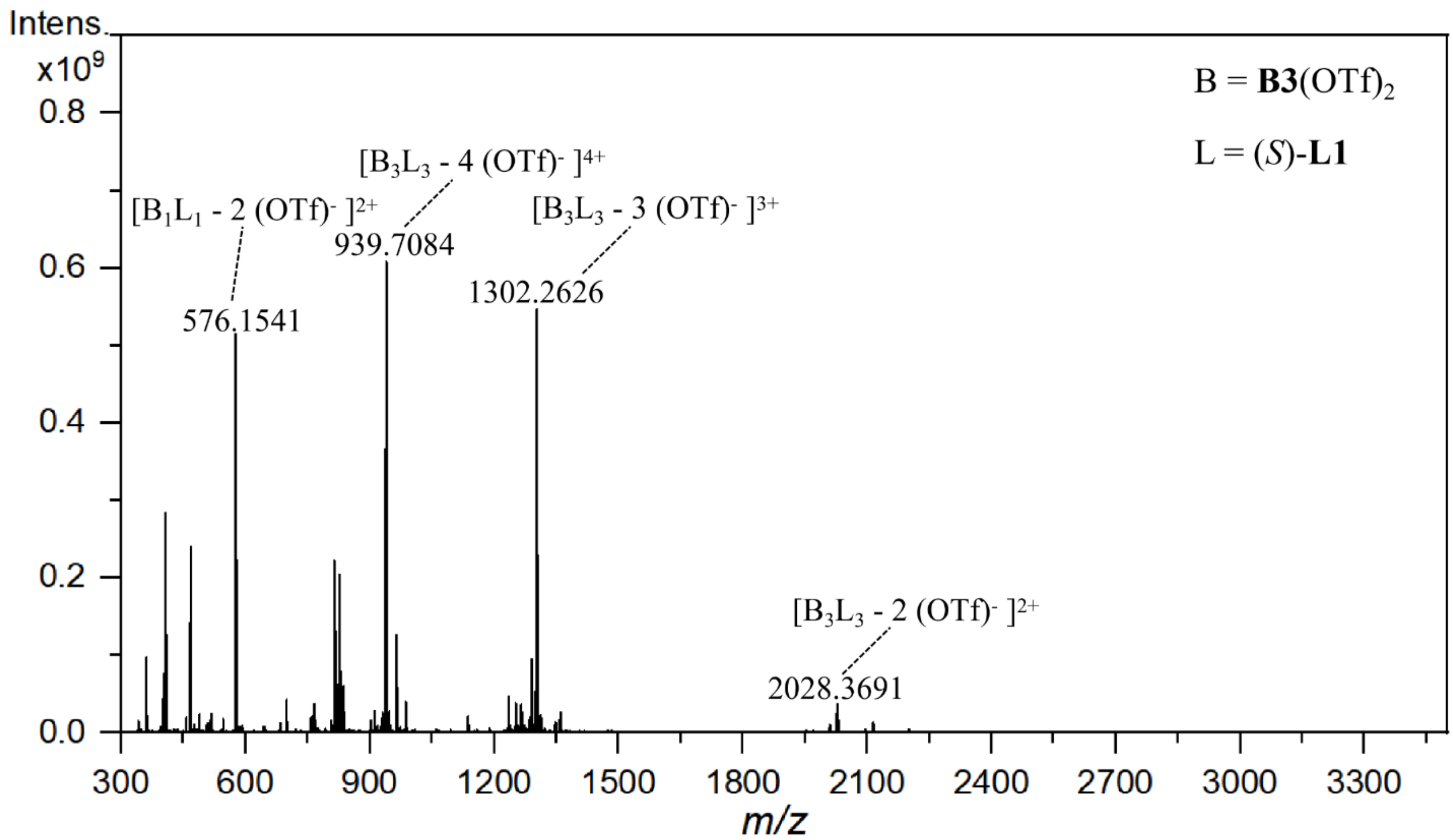

Fig. S178. Full-range ESI-MS spectrum of Λ-**Ir-1** in methanol.

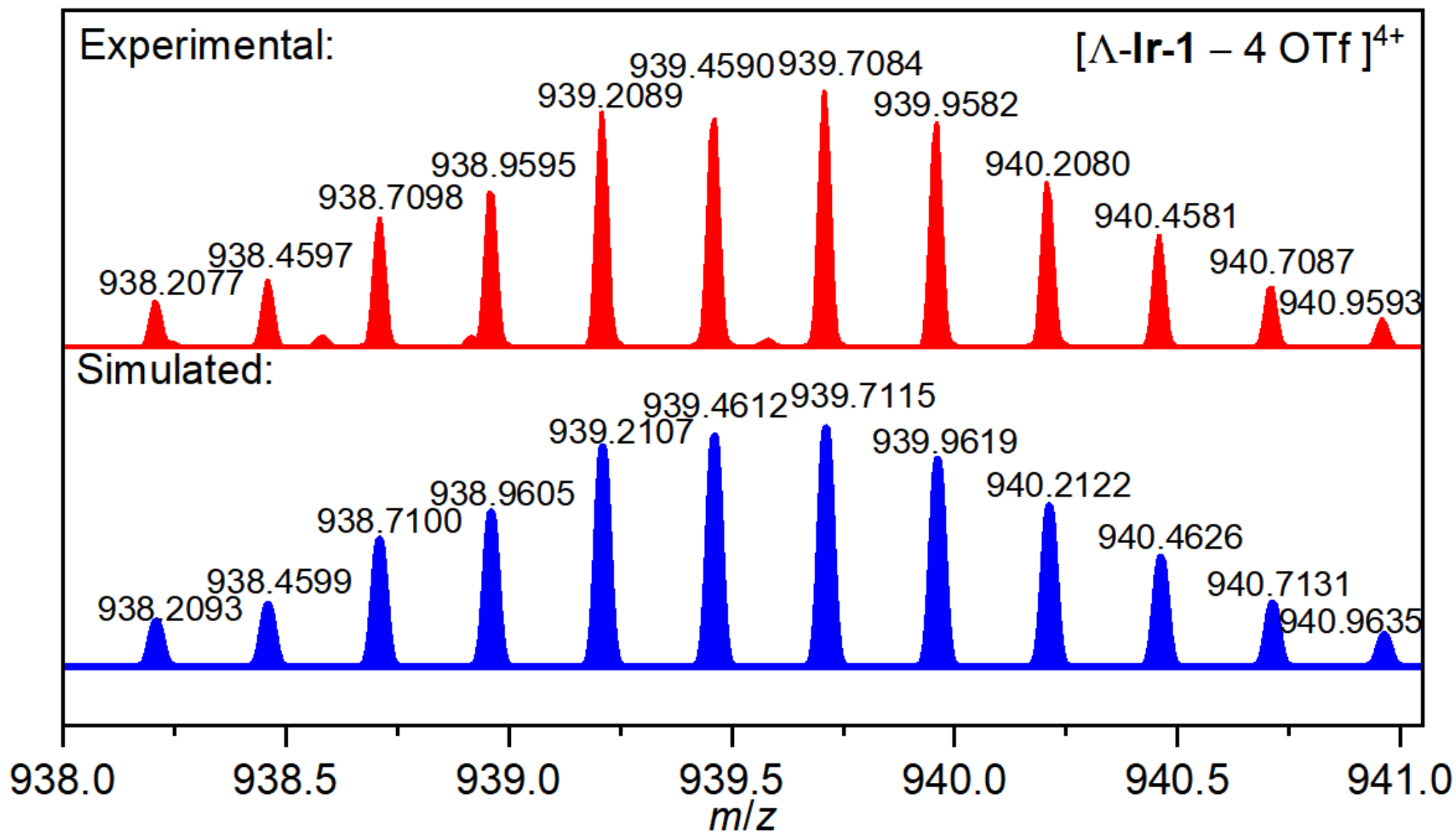

Fig. S179. Experimental and calculated isotope patterns of [Λ-**Ir-1** – 4OTf]$^{4+}$.

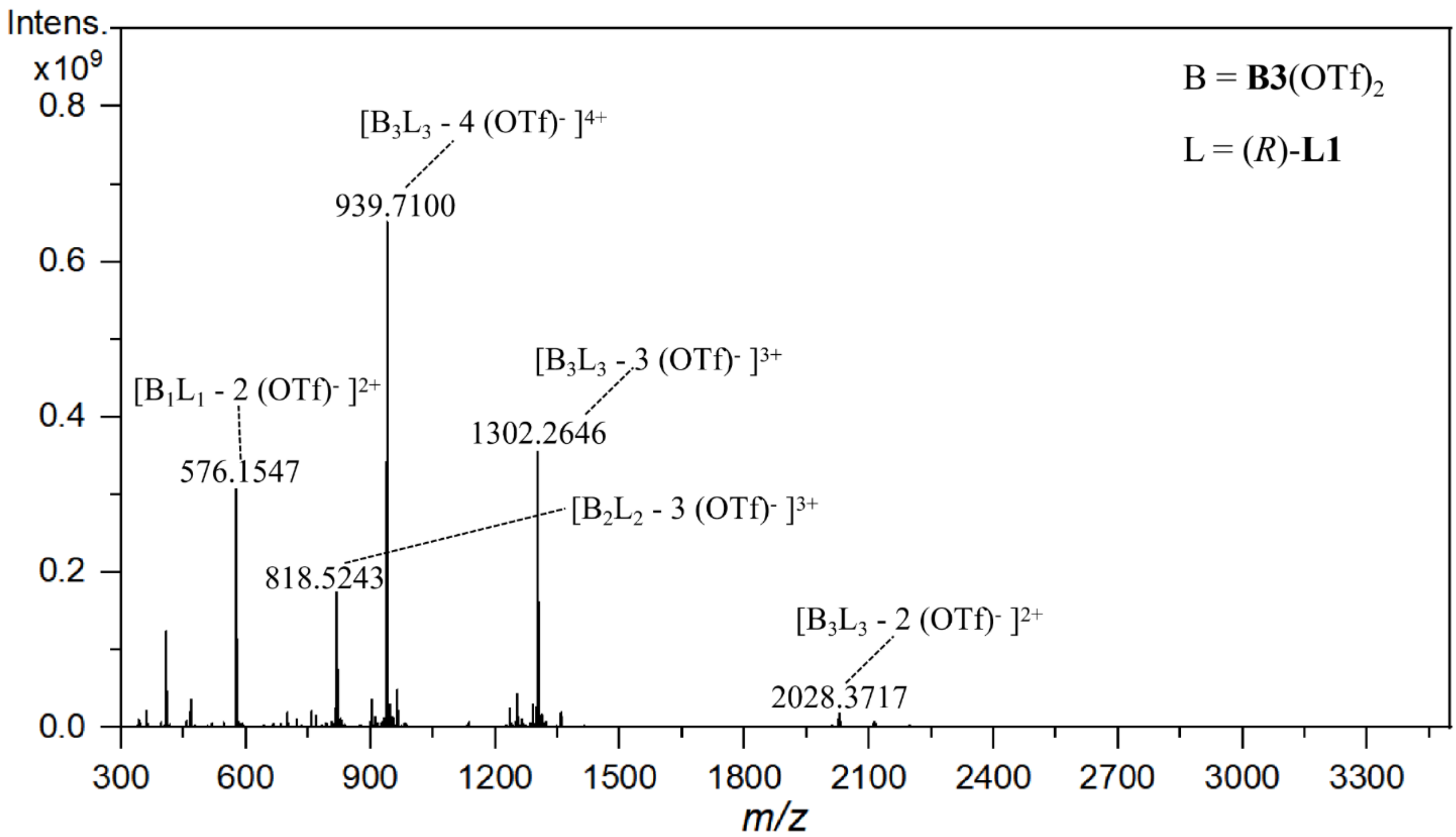

Fig. S180. Full-range ESI-MS spectrum of Δ-**Ir-1** in methanol.

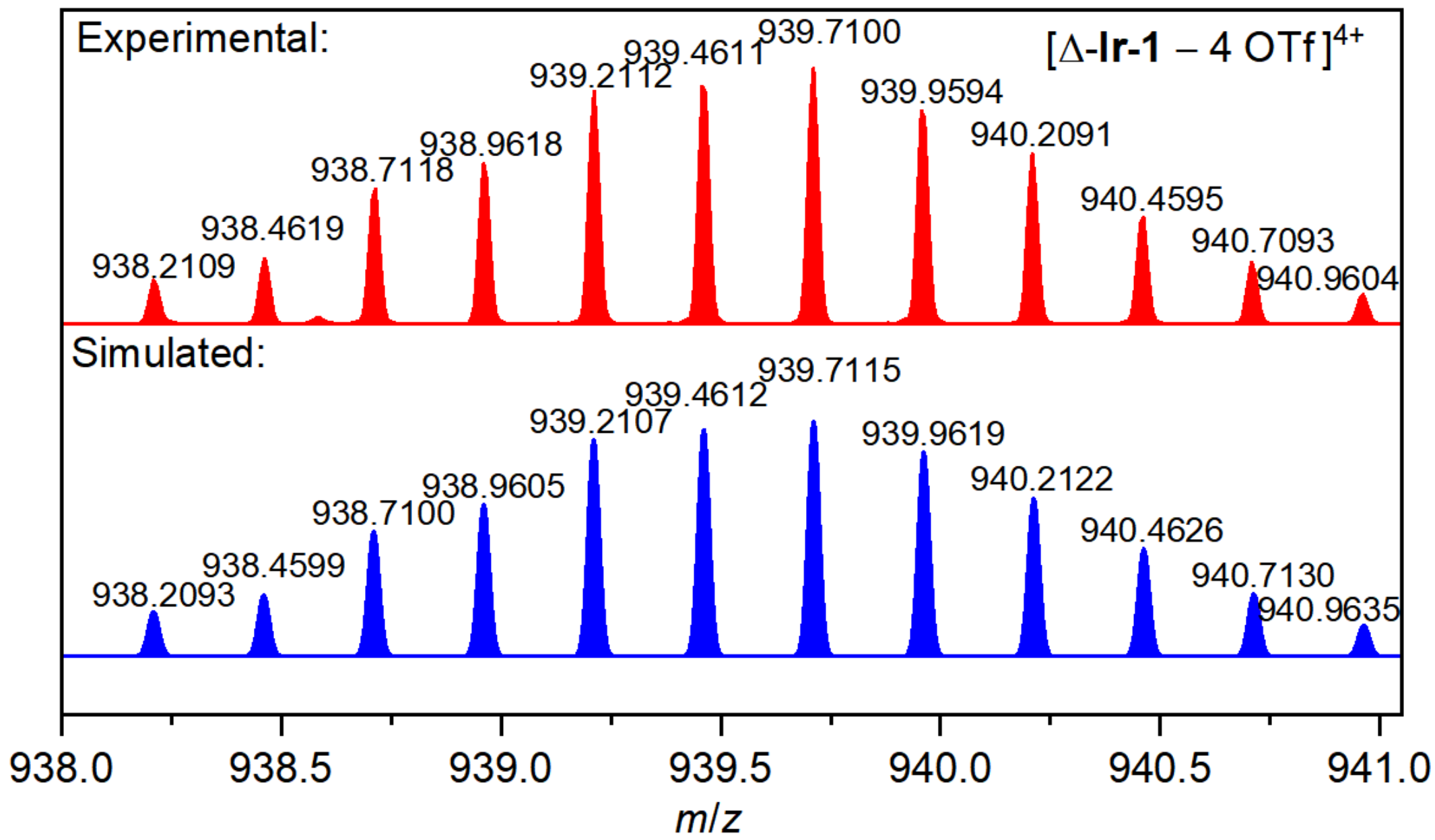

Fig. S181. Experimental and calculated isotope patterns of [Δ-**Ir-1** – 4OTf]$^{4+}$.

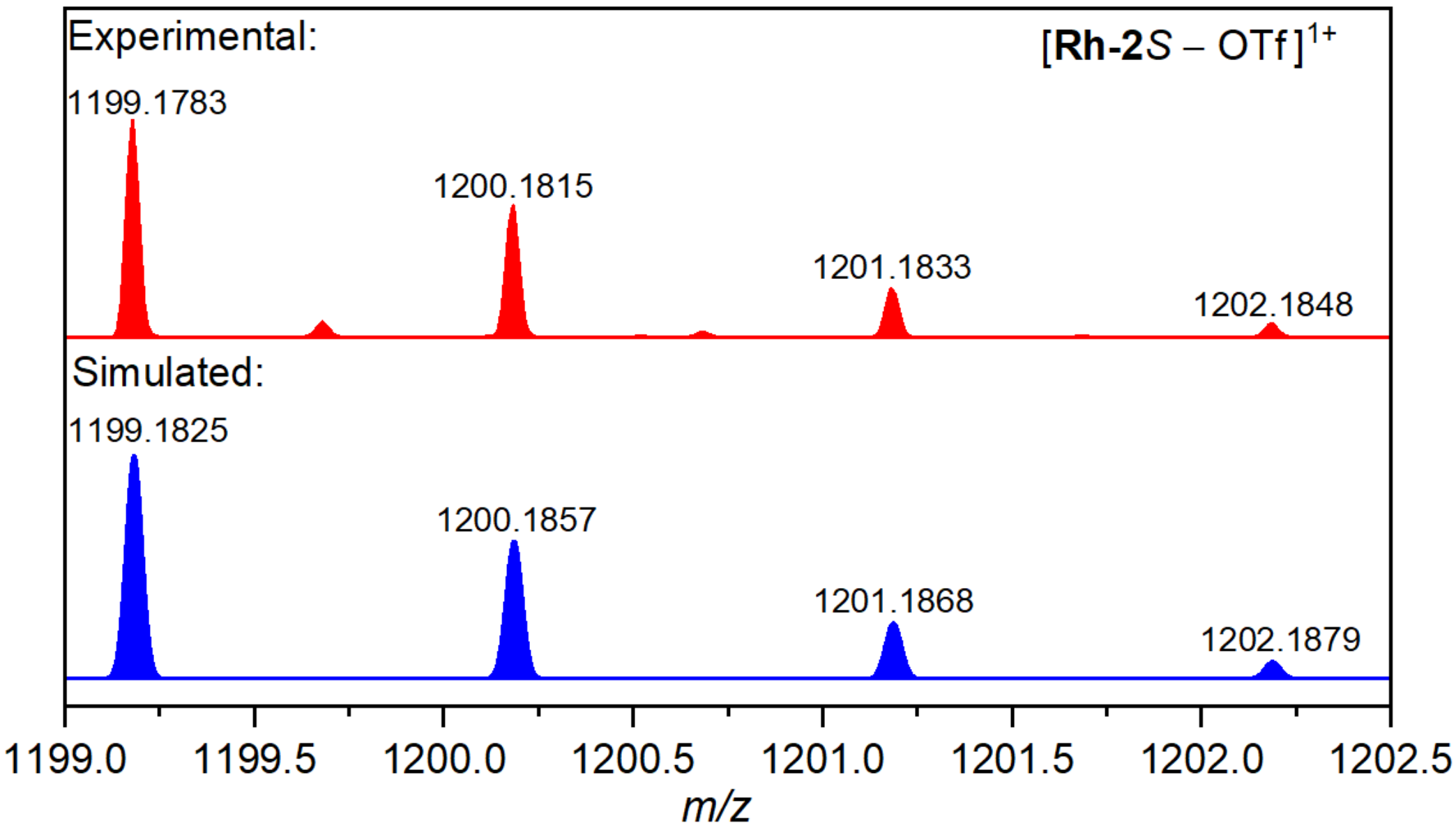

Fig. S182. Experimental and calculated isotope patterns of [**Rh-2***S* – OTf]$^+$.

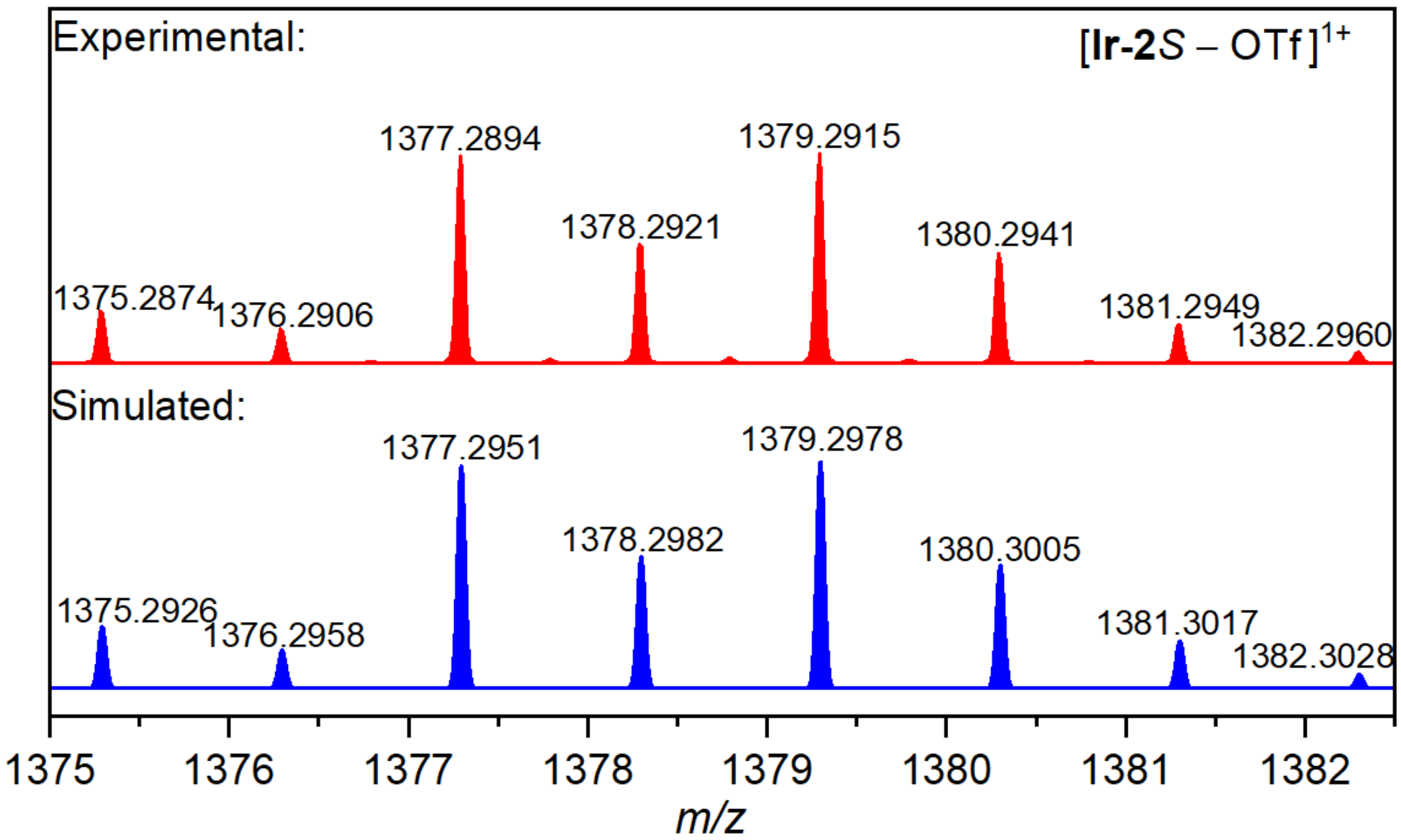

Fig. S183. Experimental and calculated isotope patterns of [**Ir-2***S* – OTf]$^+$.

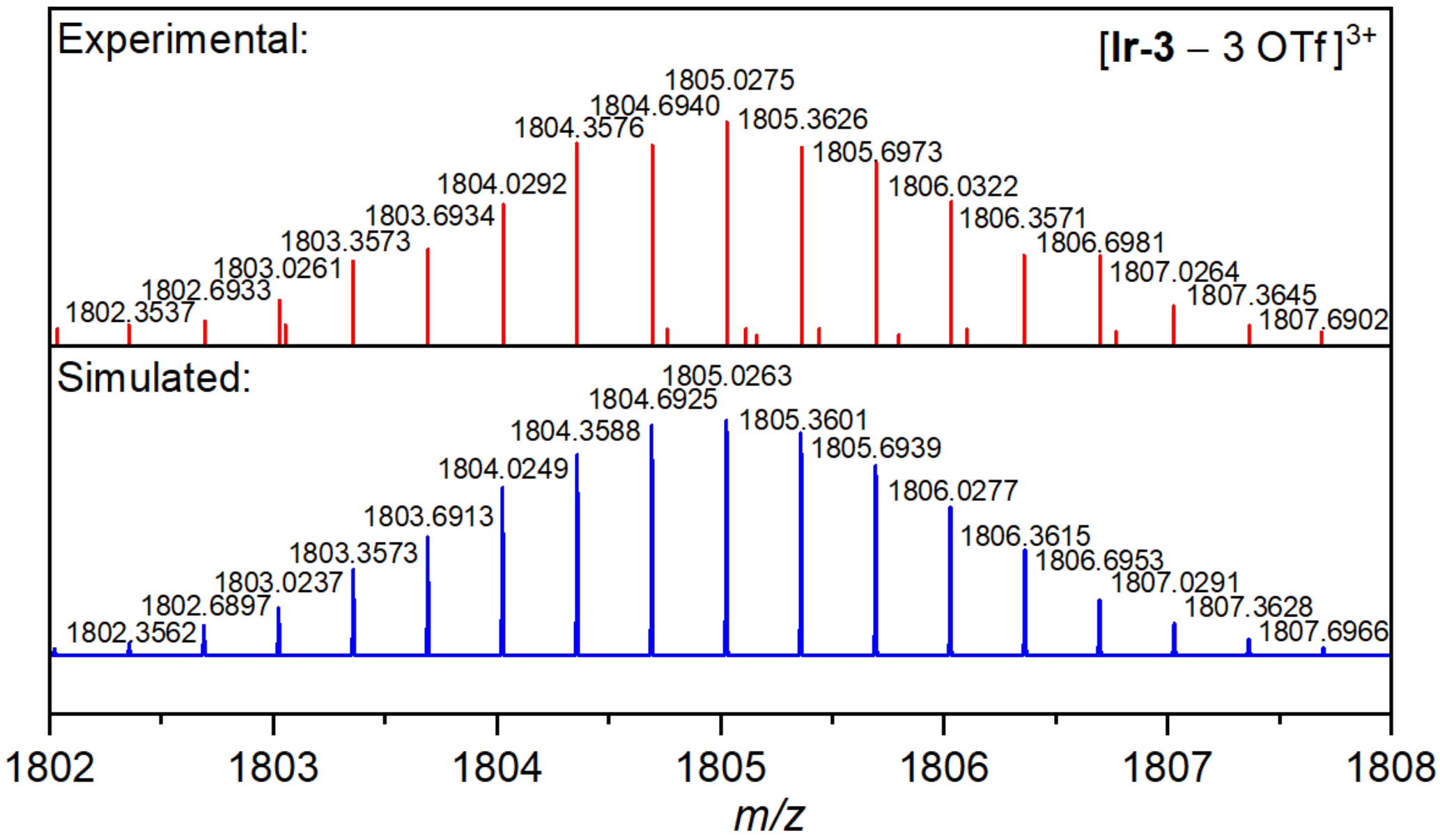

Fig. S184. Experimental and calculated isotope patterns of [**Ir-3** – 3 OTf]$^{3+}$.

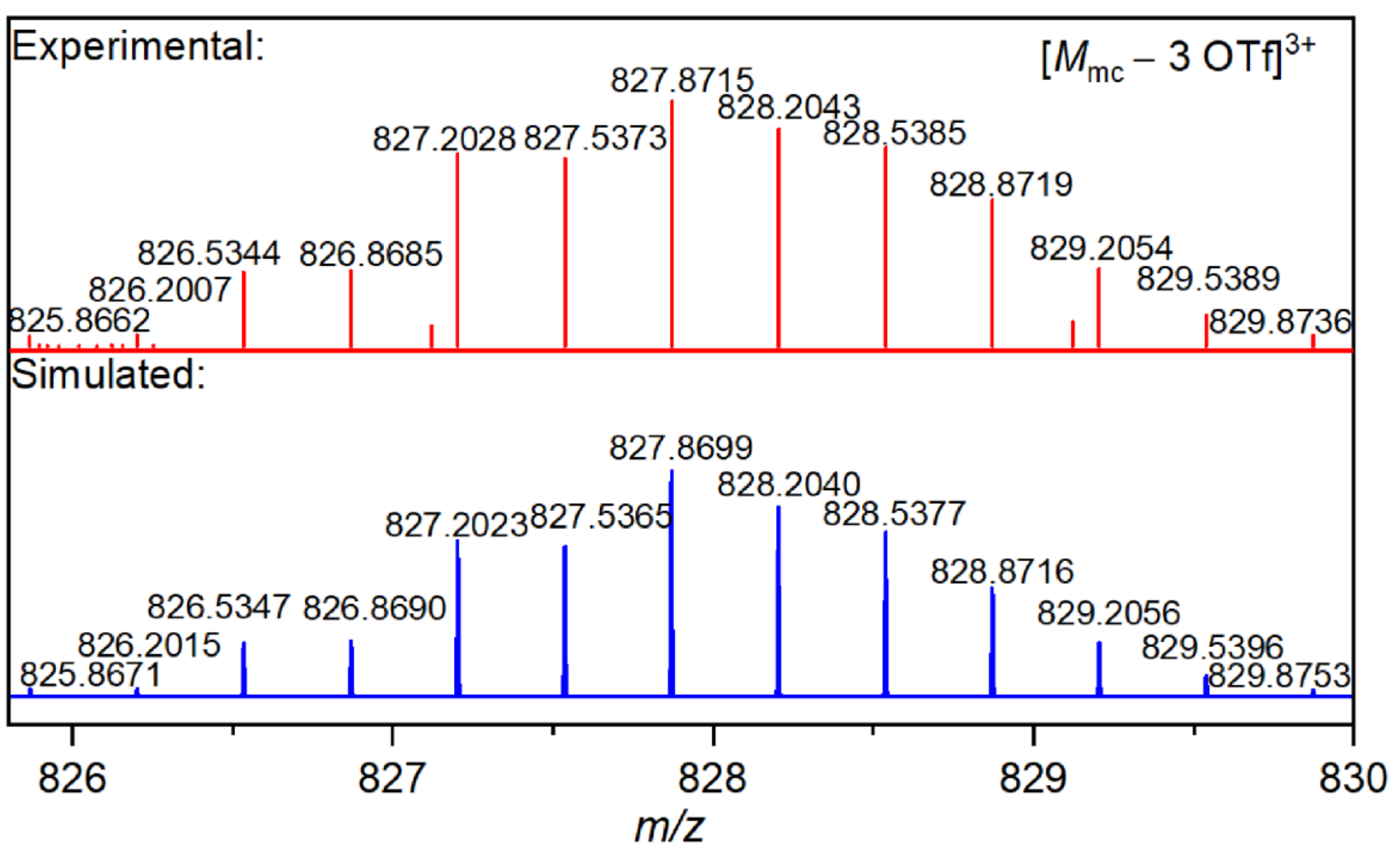

Fig. S185. Experimental and calculated isotope patterns of [M_{mc} – 3OTf]$^{3+}$, where M_{mc} denotes the tetranuclear metallomacrocycle salt, including its four triflate counterions, in the **L3**/**B3**(OTf)$_2$ assembly system.

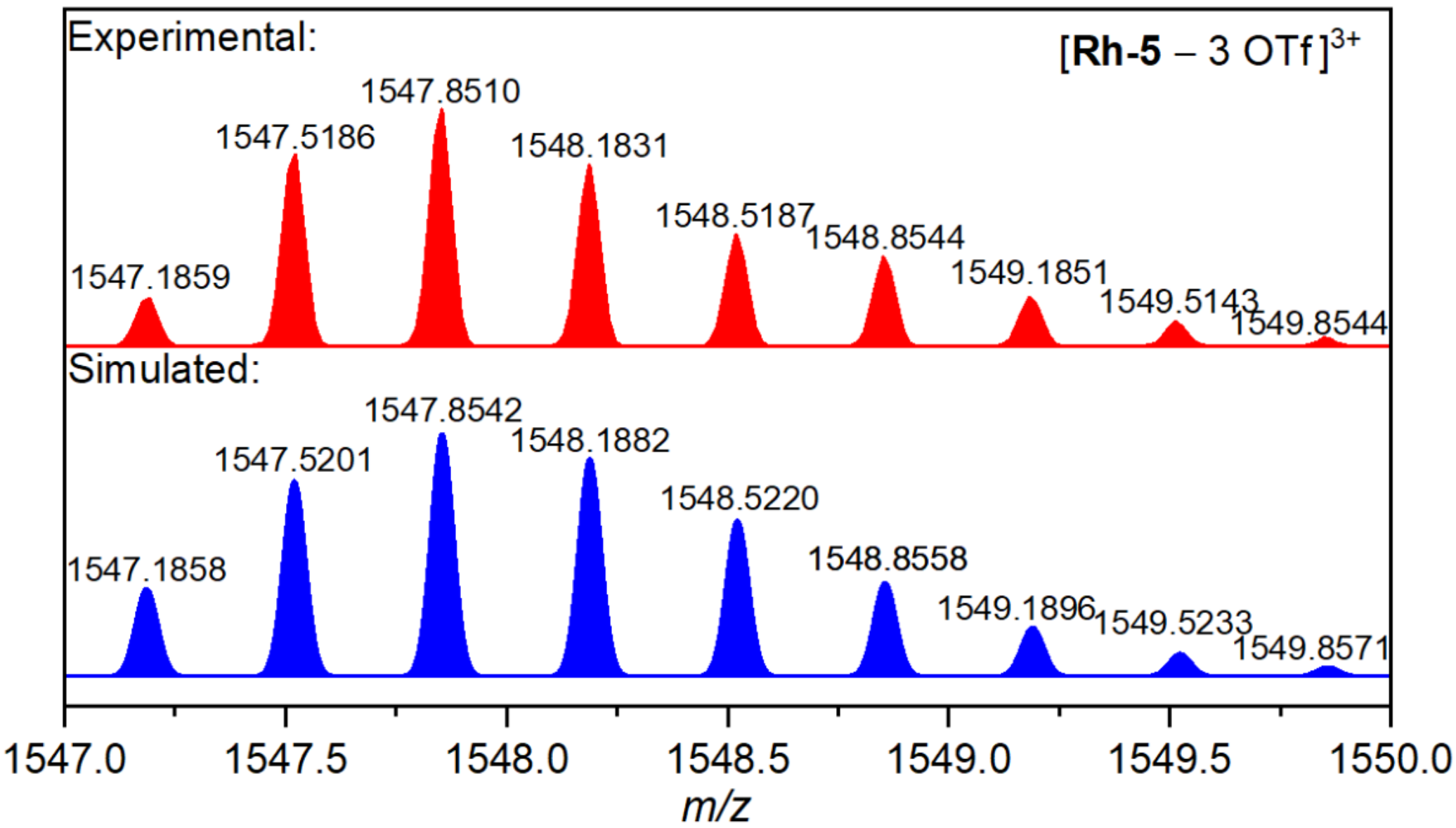

Fig. S186. Experimental and calculated isotope patterns of [**Rh-5** – 3OTf]$^{3+}$.

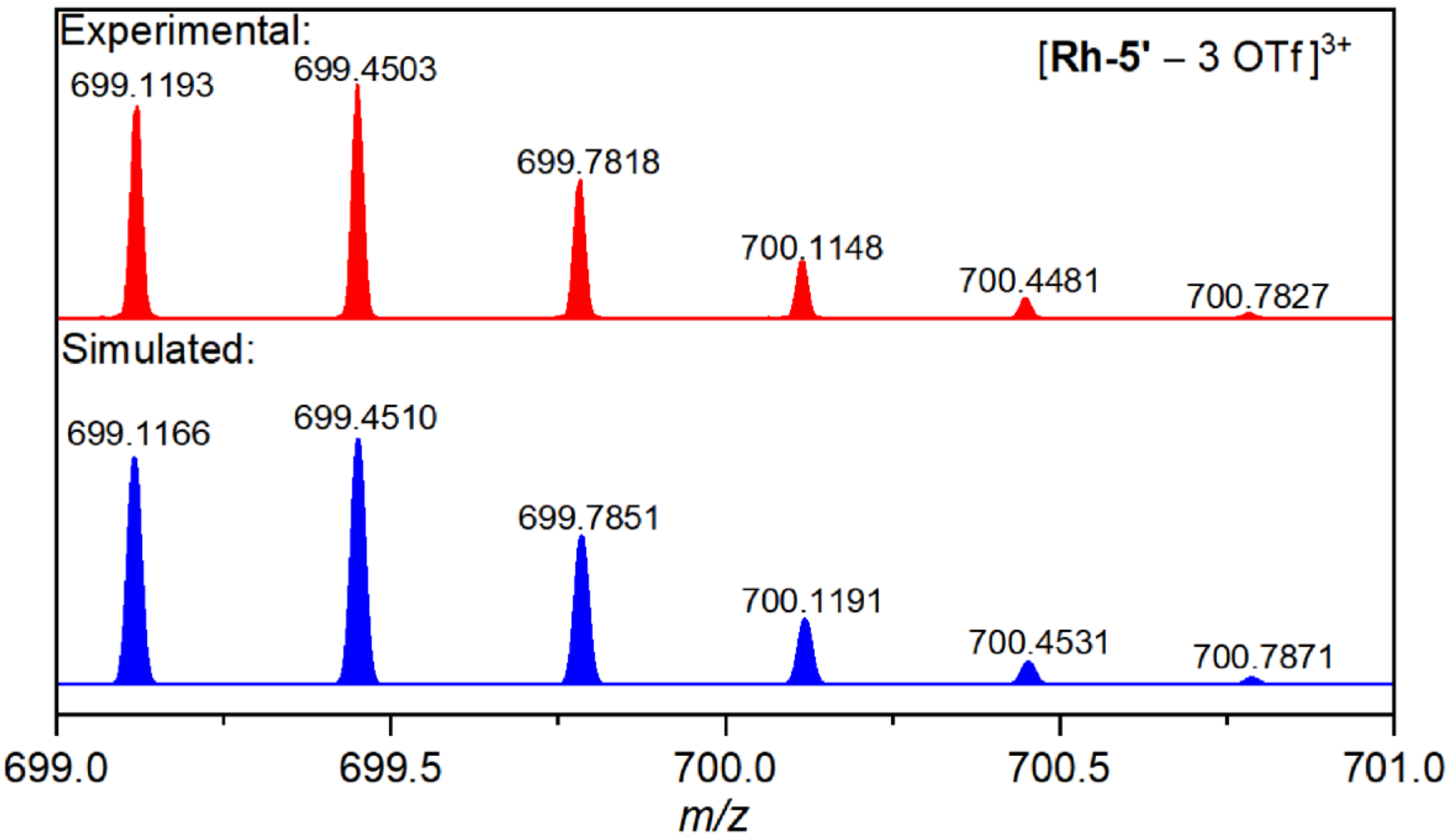

Fig. S187. Experimental and calculated isotope patterns of [**Rh-5'** – 3OTf]$^{3+}$.

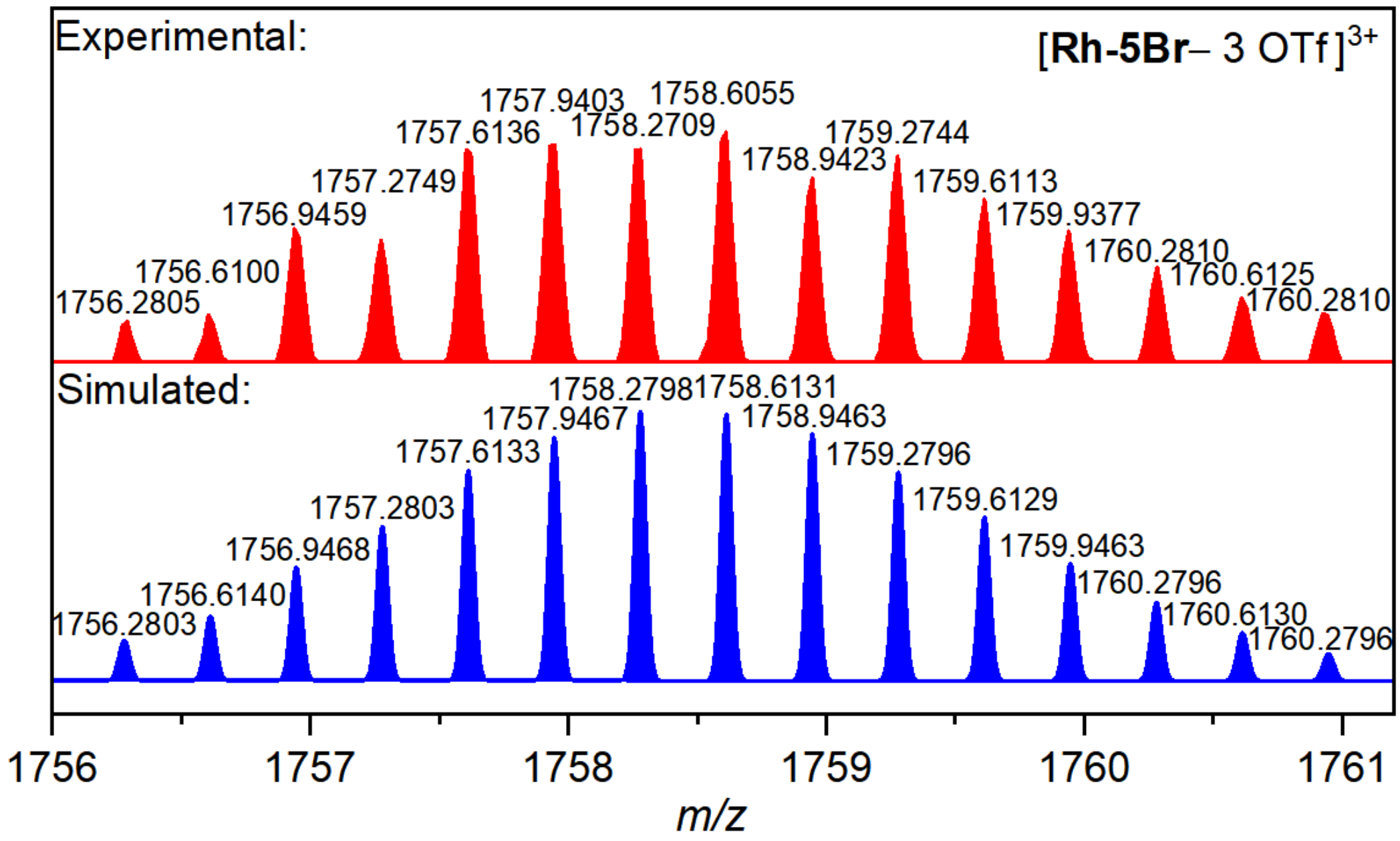

Fig. S188. Experimental and calculated isotope patterns of [**Rh-5Br** – 3OTf]$^{3+}$.

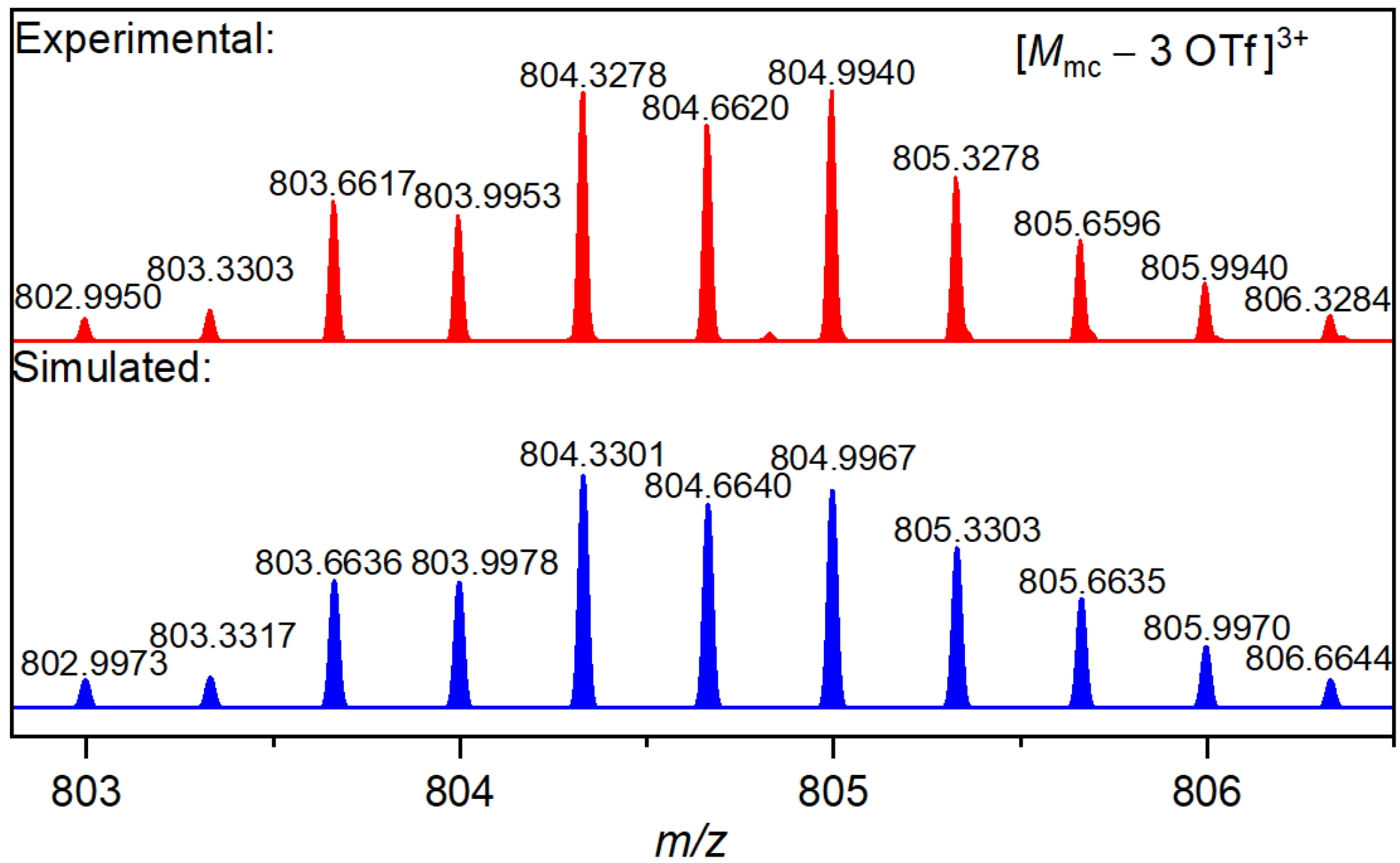

Fig. S189. Experimental and calculated isotope patterns of [M_{mc} – 3OTf]$^{3+}$, where M_{mc} denotes the tetranuclear metallomacrocycle salt, including its four triflate counterions, in the **L5**/**B2**(OTf)$_2$ assembly system.

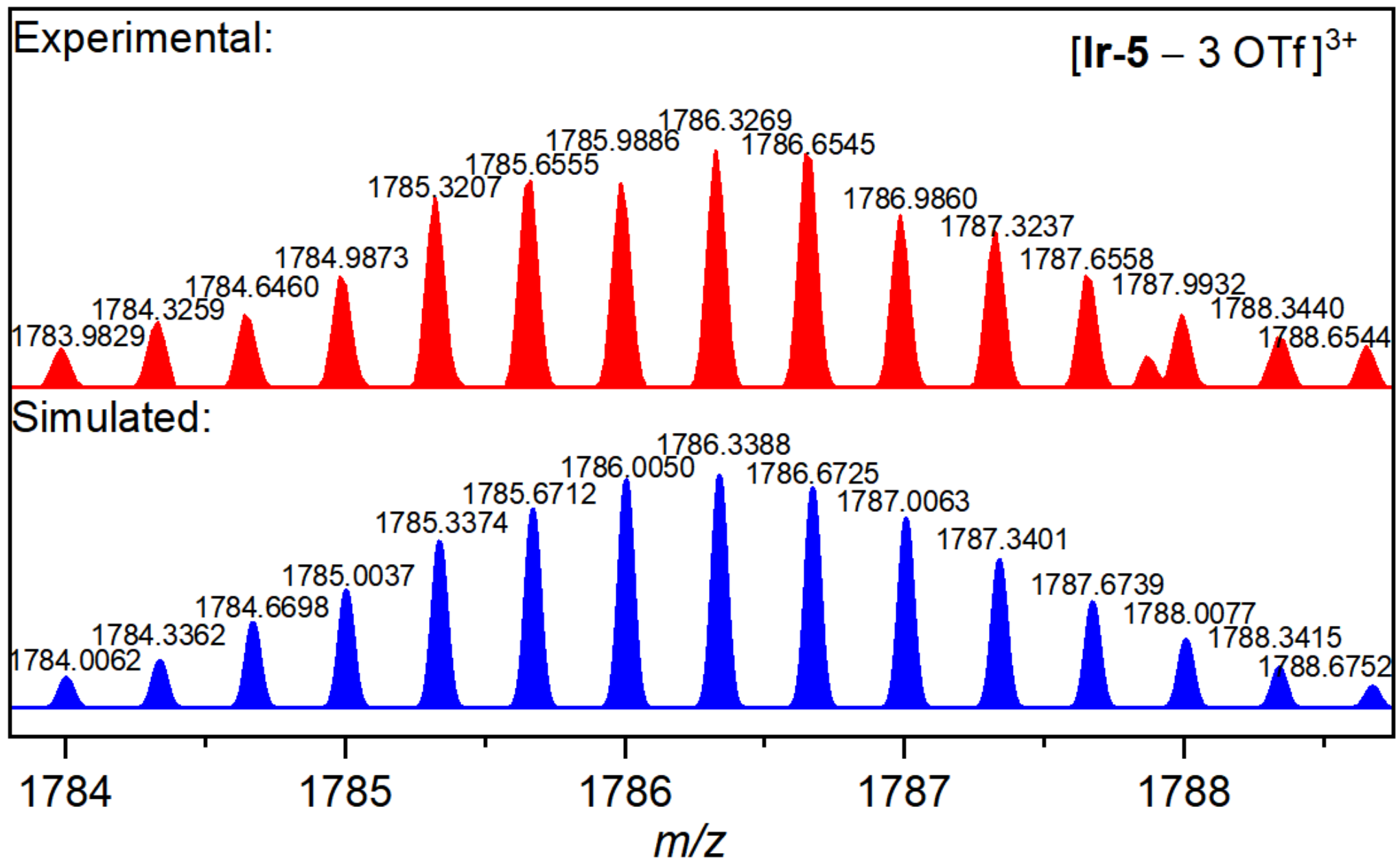

Fig. S190. Experimental and calculated isotope patterns of [**Ir-5** – 3OTf]$^{3+}$.

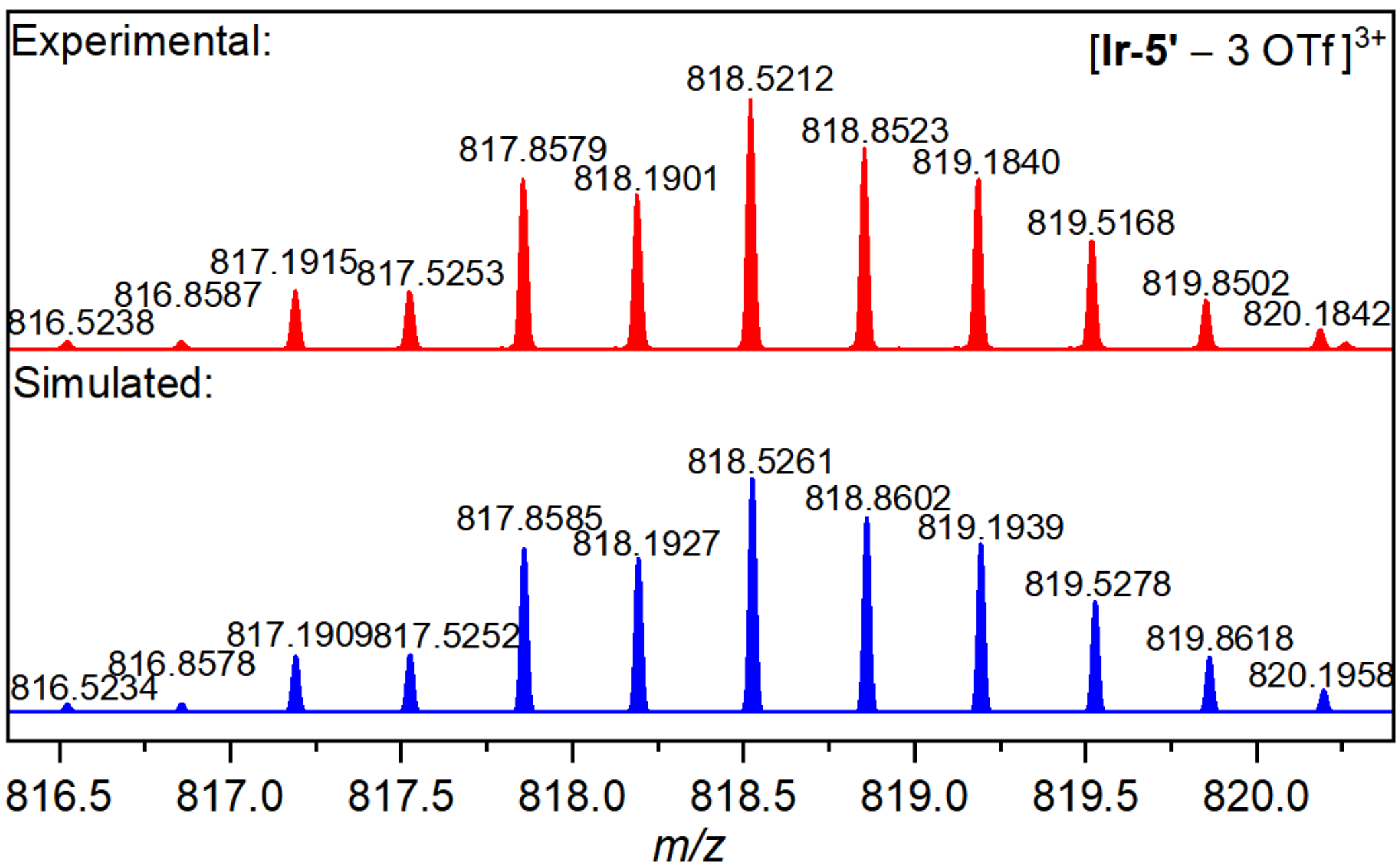

Fig. S191. Experimental and calculated isotope patterns of [**Ir-5**′ – 3OTf]$^{3+}$.

8. UV-Vis and CD Spectra

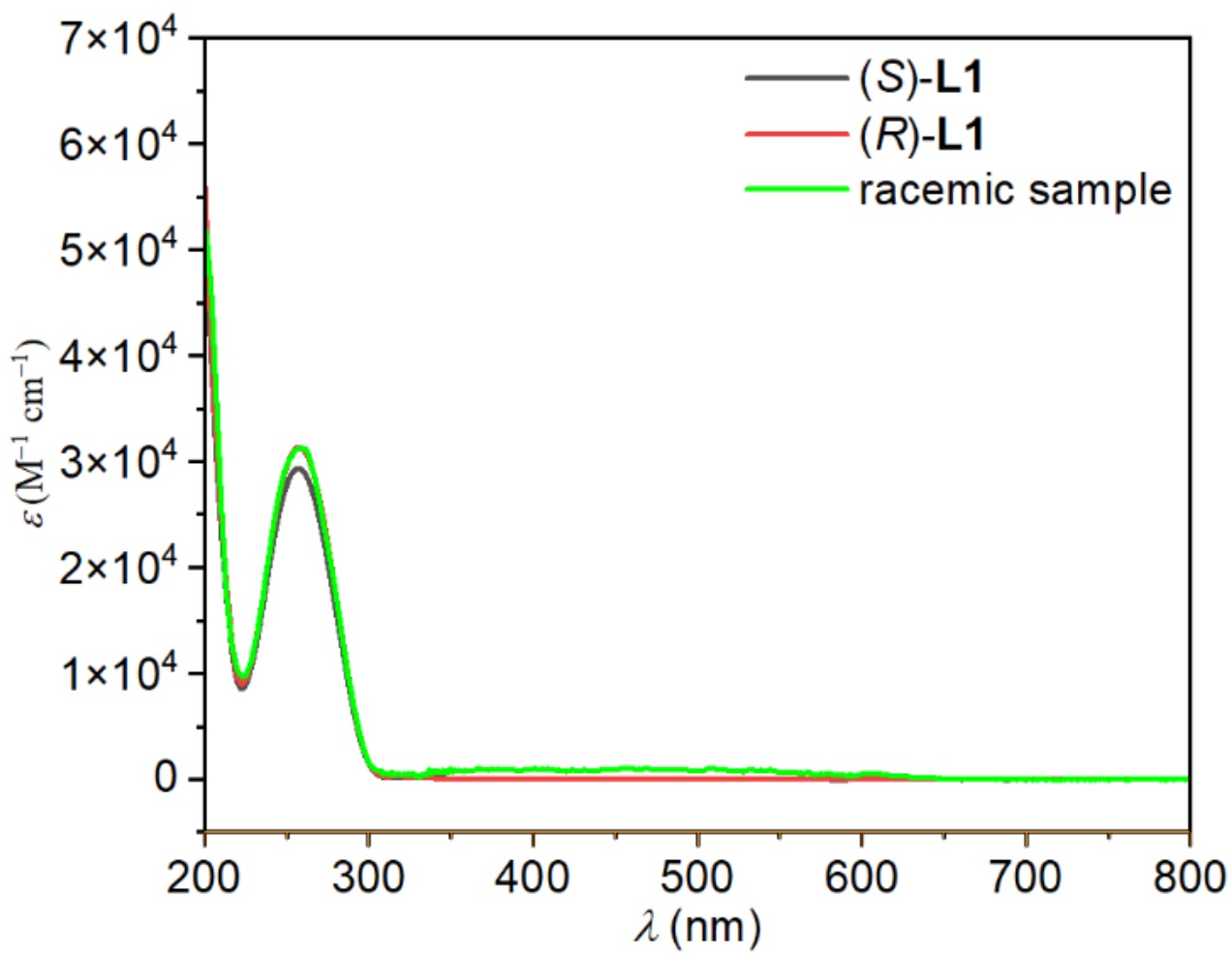

Fig. S192. UV–vis absorption spectra of (*S*)-**L1**, (*R*)-**L1**, and their 1:1 racemic mixture in methanol at room temperature (total ligand concentration, c_{ligand} = 2.4 mM; optical path length, *l* = 0.1 mm). Spectra are expressed as molar absorption coefficients, *ε*, per mole of ligand. The racemic mixture and the individual enantiomers exhibit essentially overlapping absorption spectra.

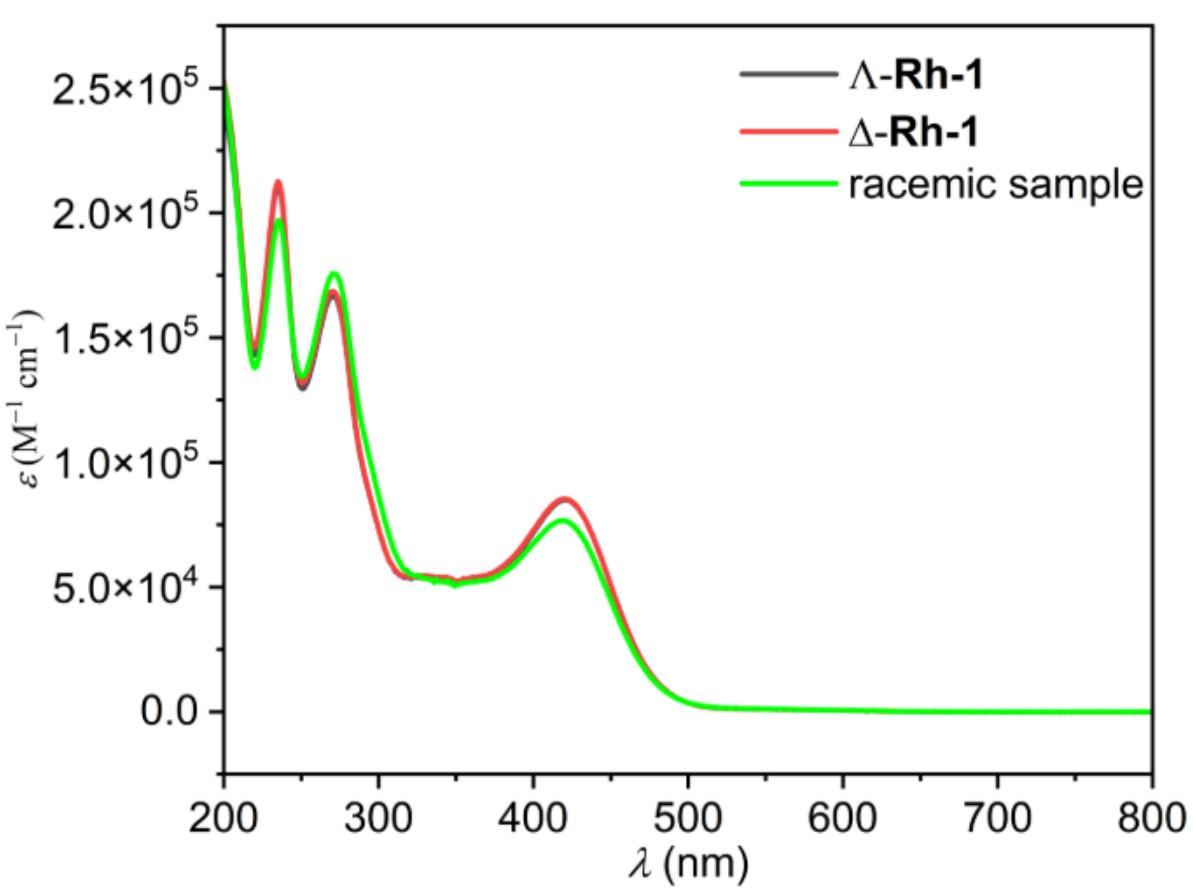

Fig. S193. UV–vis absorption spectra of Λ-**Rh-1**, Δ-**Rh-1**, and the racemic trefoil-knot mixture obtained by self-assembly of equimolar (*S*)-**L1** and (*R*)-**L1** with **B1**$(OTf)_2$ in methanol at room temperature (total knot

concentration, c = 0.8 mM; c_{Rh} = 4.8 mM; optical path length, l = 0.1 mm). Spectra are expressed as molar absorption coefficients, ε, per mole of complete assembly. The racemic mixture and the individual enantiomers exhibit essentially overlapping absorption spectra.

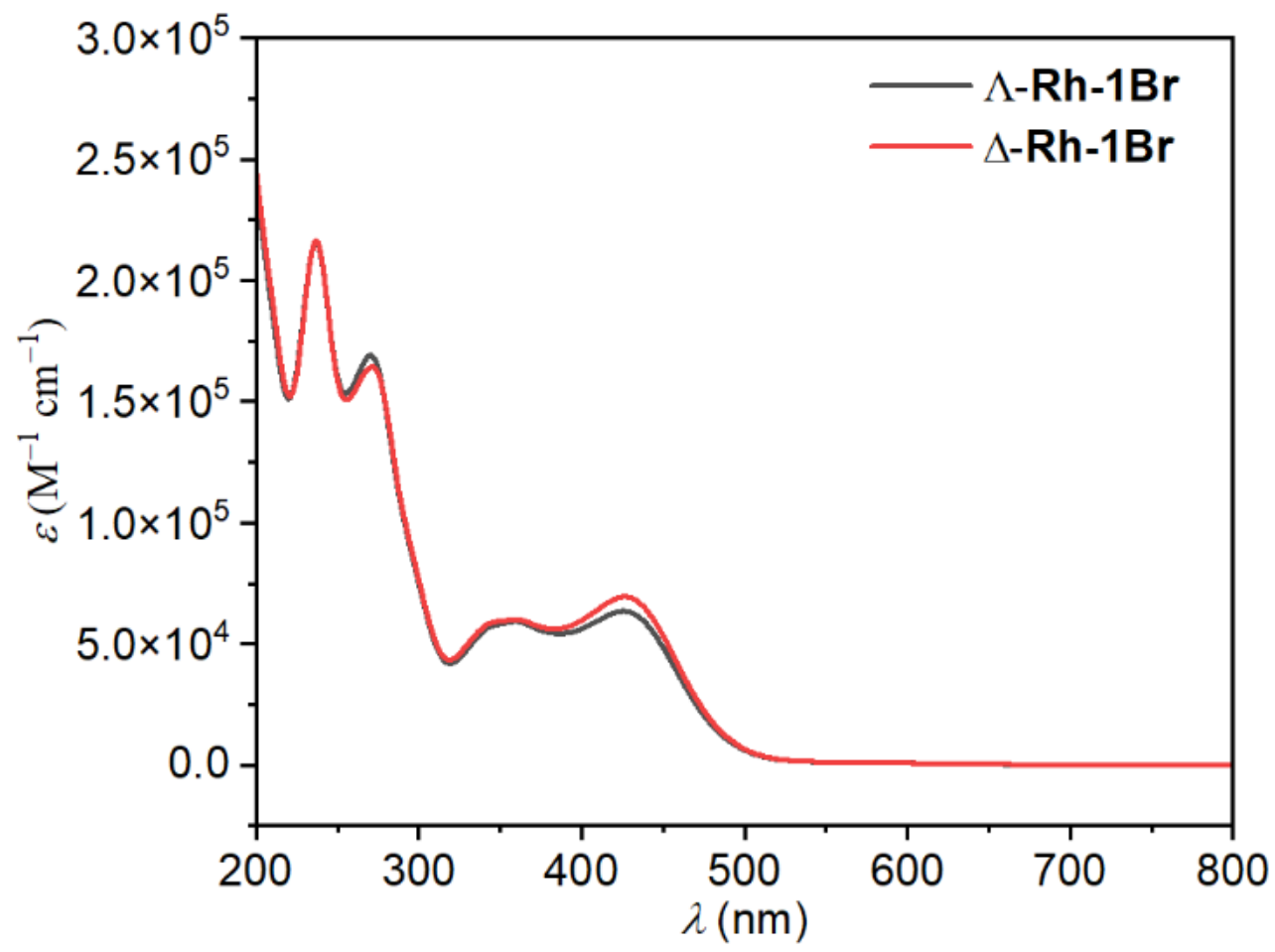

Fig. S194. UV–vis absorption spectra of Λ-**Rh-1Br** and Δ-**Rh-1Br** in methanol at room temperature (c = 0.8 mM, c_{Rh} = 4.8 mM; optical path length l = 0.1 mm). Spectra are expressed as molar absorption coefficients, ε, using concentrations calculated per complete assembly.

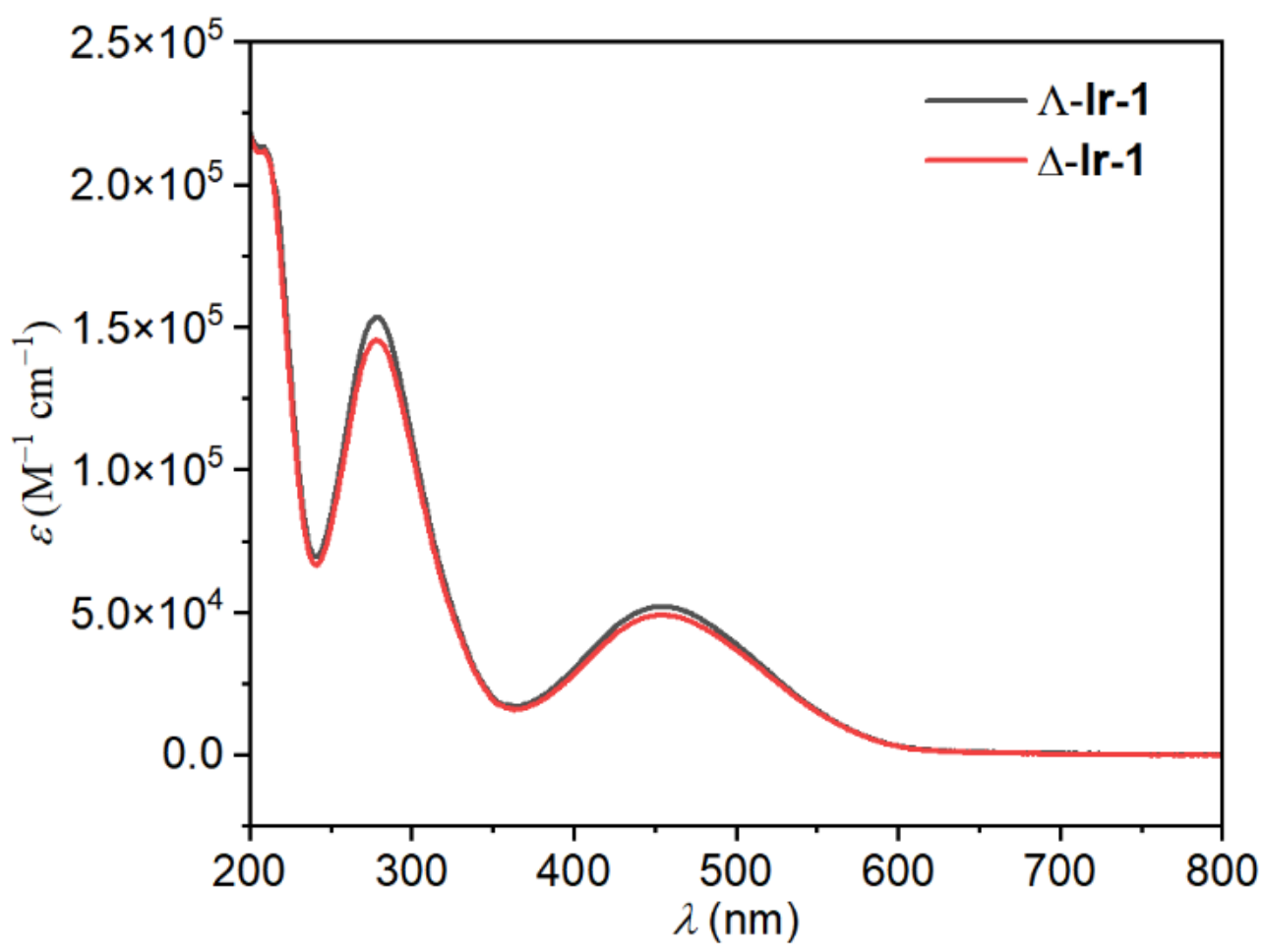

Fig. S195. UV–vis absorption spectra of Λ-**Ir-1** and Δ-**Ir-1** in methanol at room temperature (c = 0.8 mM, c_{Ir} = 4.8 mM; optical path length l = 0.1 mm). Spectra are expressed as molar absorption coefficients, ε, using concentrations calculated per complete assembly.

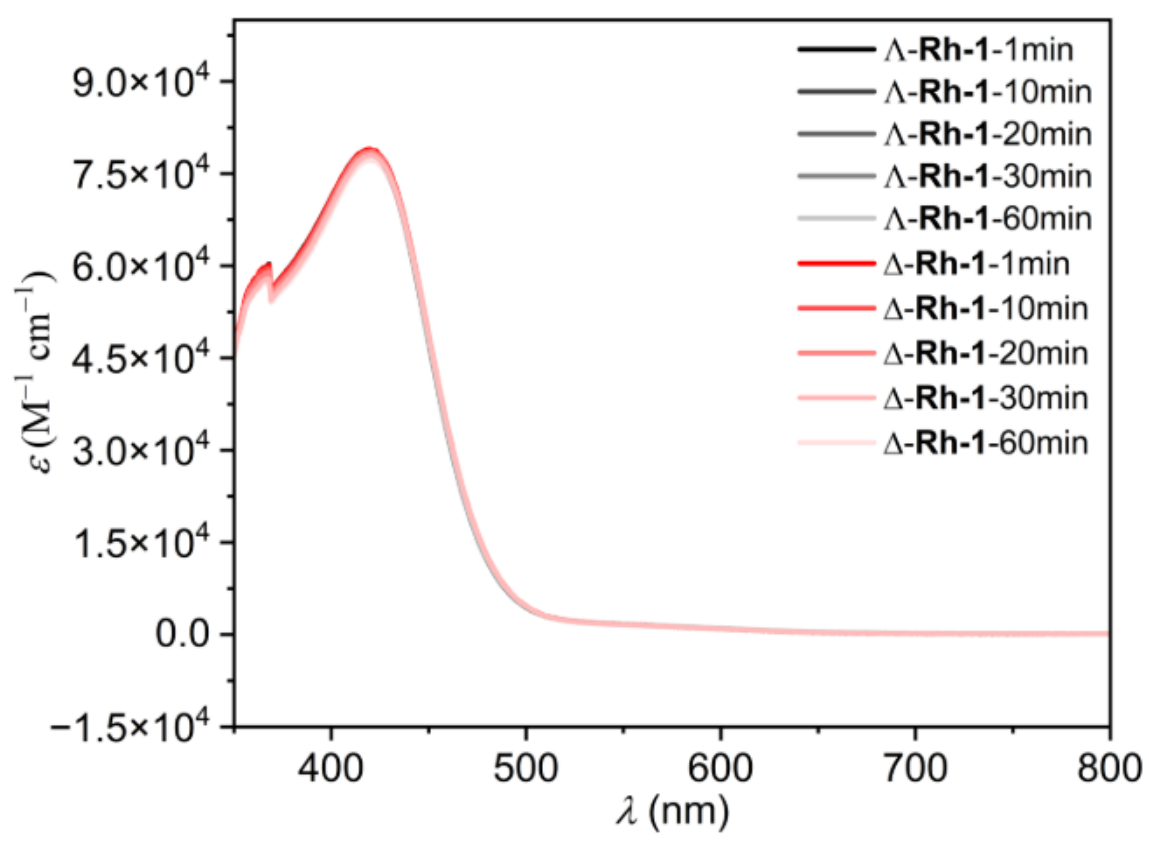

Fig. S196. Time-dependent UV–vis absorption spectra of the reaction mixtures of (S)-**L1** or (R)-**L1** with **B1**$(OTf)_2$ in methanol at room temperature (c_{Rh} = 20.0 mM; optical path length l = 0.1 mm). Spectra are normalized to a formal knot concentration of $c = c_{Rh}/6$ = 3.333 mM and are expressed as molar absorption coefficients, □.

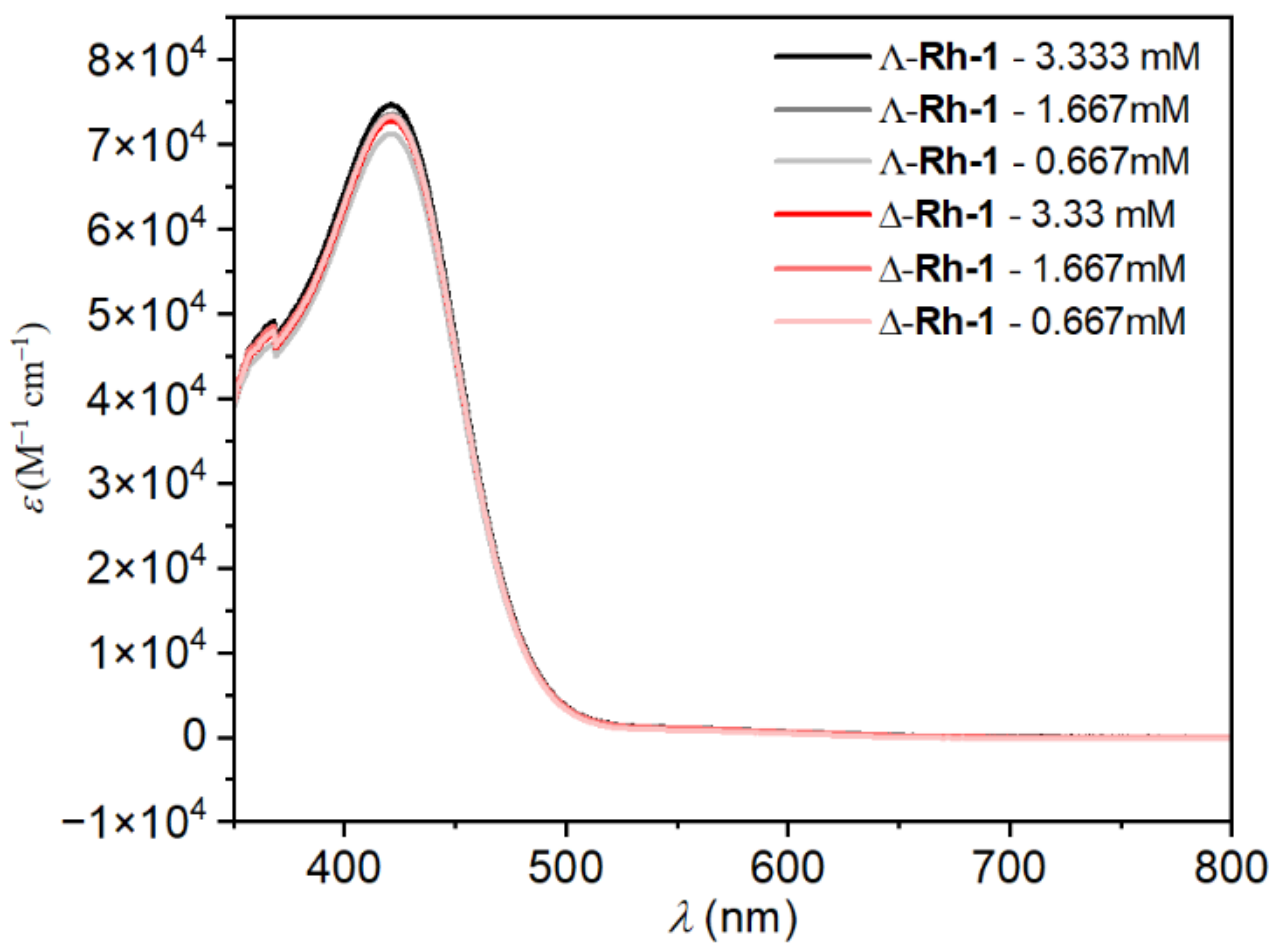

Fig. S197. Concentration-dependent UV–vis absorption spectra of Λ-**Rh-1** and Δ-**Rh-1** in methanol at room temperature. Formal knot concentrations of $c = 0.667$, 1.667, and 3.333 mM ($c_{Rh} = 4.0$, 10.0, and 20.0 mM) were examined using optical path lengths of 0.5, 0.2, and 0.1 mm, respectively. Spectra are expressed as molar absorption coefficients, ε, normalized using $c = c_{Rh}/6$. The molar absorption profiles remain nearly unchanged over this concentration range.

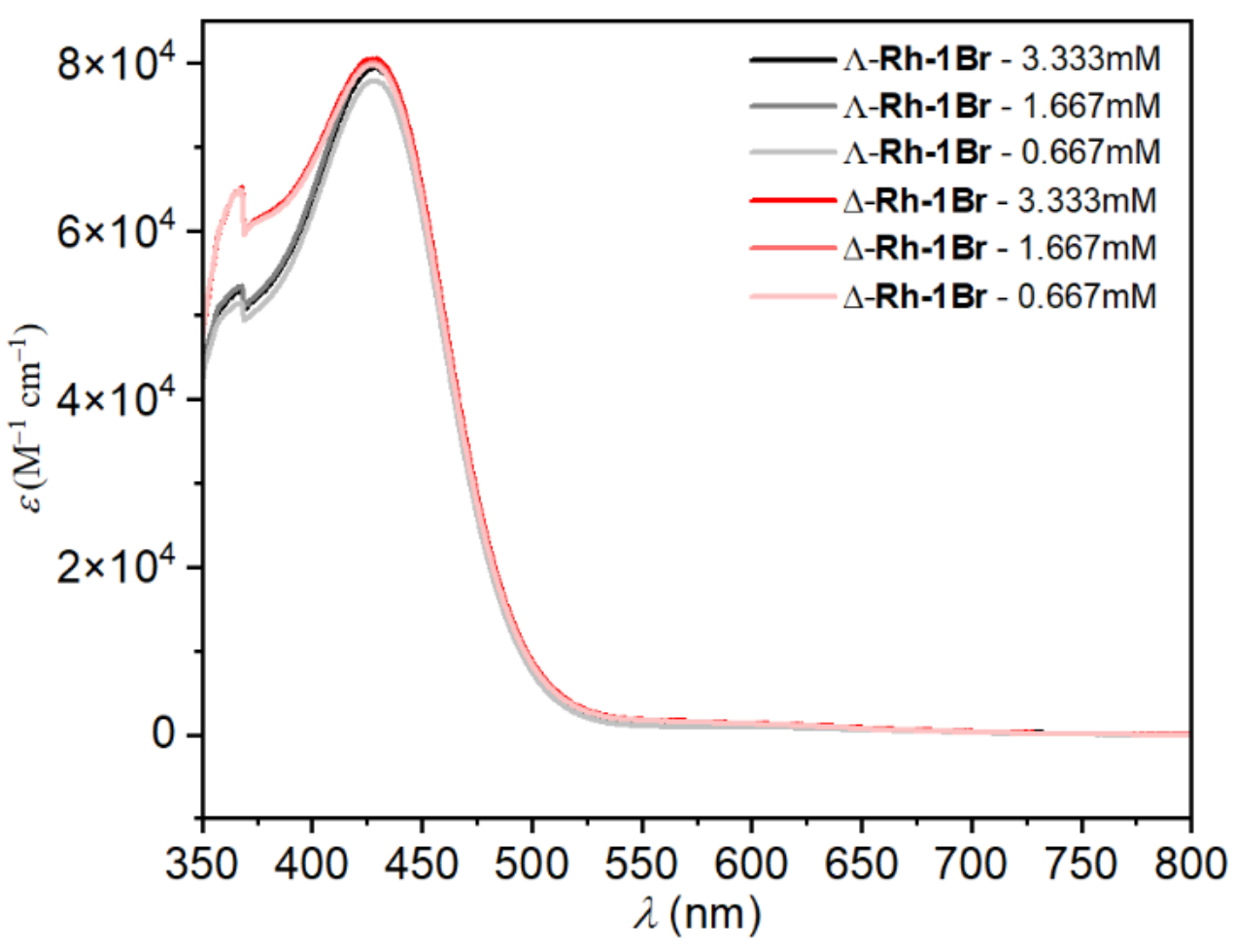

Fig. S198. Concentration-dependent UV–vis absorption spectra of Λ-**Rh-1Br** and Δ-**Rh-1Br** in methanol at room temperature. Formal knot concentrations of $c = 0.667$, 1.667, and 3.333 mM ($c_{Rh} = 4.0$, 10.0, and 20.0 mM) were examined using optical path lengths of 0.5, 0.2, and 0.1 mm, respectively. Spectra are expressed as molar absorption coefficients, ε, normalized using $c = c_{Rh}/6$. The molar absorption profiles remain nearly unchanged over this concentration range.

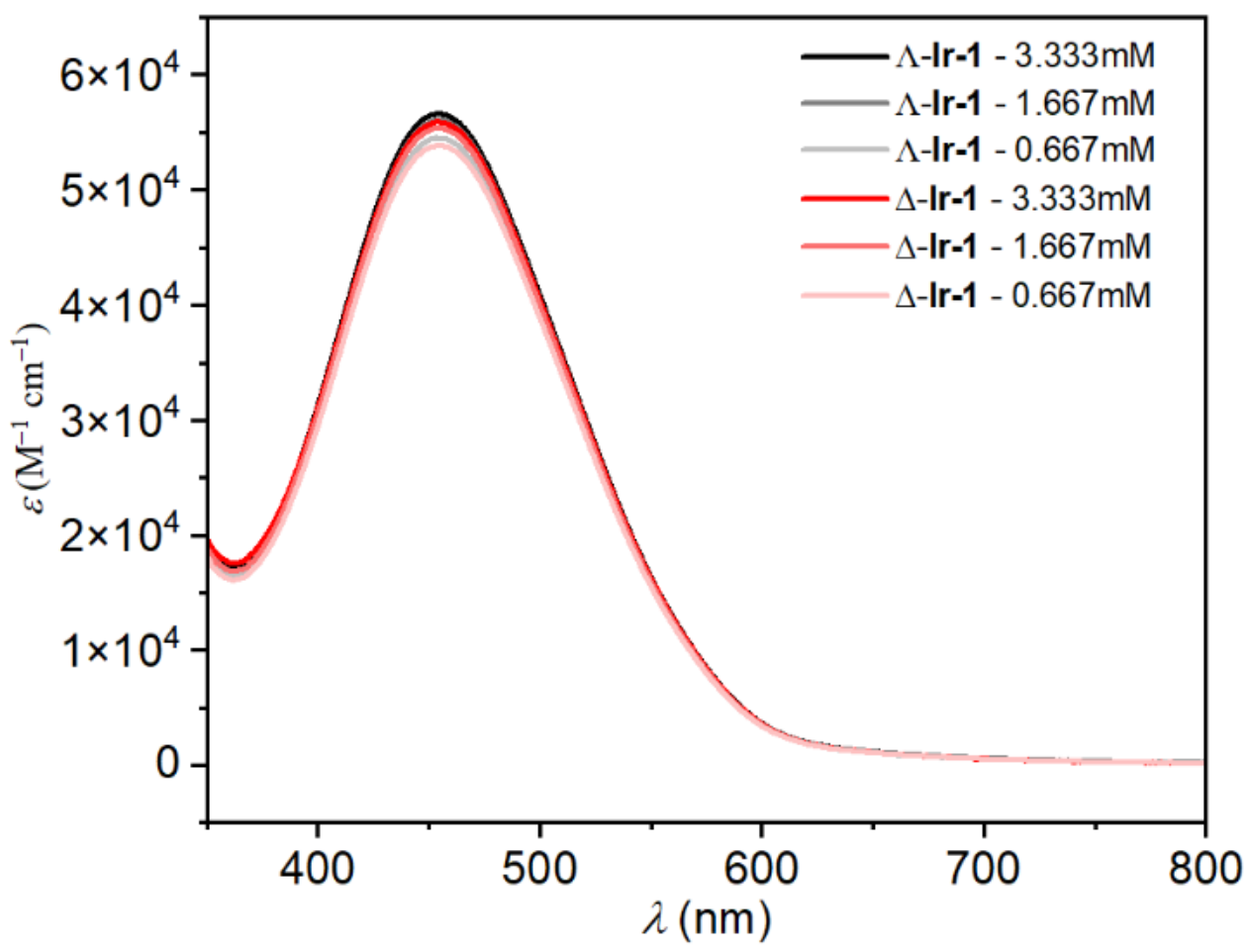

Fig. S199. Concentration-dependent UV–vis absorption spectra of Λ-**Ir-1** and Δ-**Ir-1** in methanol at room temperature. Formal knot concentrations of $c = 0.667$, 1.667, and 3.333 mM ($c_{\mathrm{Ir}} = 4.0$, 10.0, and 20.0 mM) were examined using optical path lengths of 0.5, 0.2, and 0.1 mm, respectively. Spectra are expressed as molar absorption coefficients, ε, normalized using $c = c_{\mathrm{Ir}}/6$. The molar absorption profiles remain nearly unchanged over this concentration range.

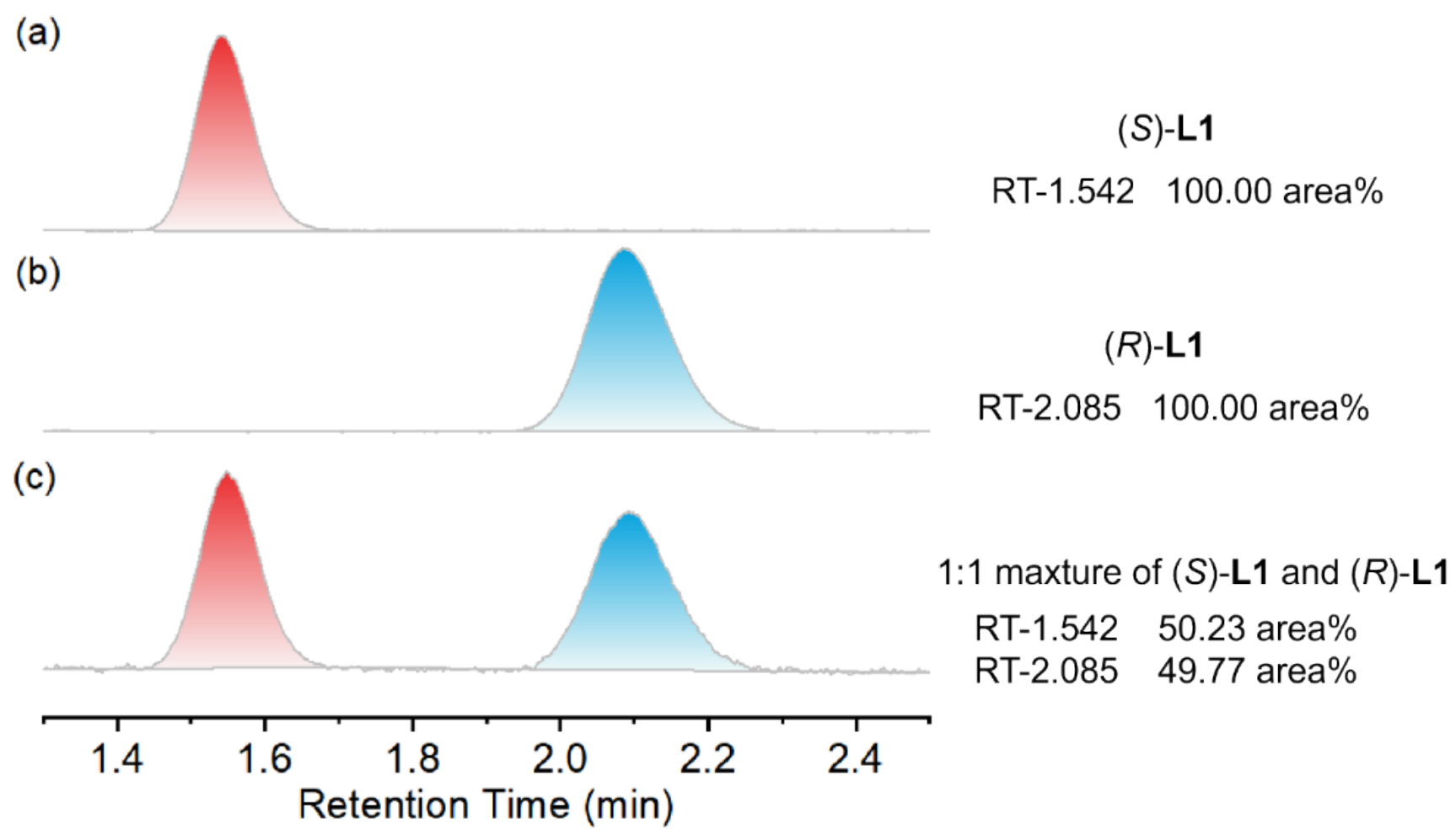

Fig. S200. Chiral SFC chromatograms of (a) (*S*)-**L1**, (b) (*R*)-**L1**, and (c) a 1:1 mixture of (*S*)-**L1** and (*R*)-**L1**. Conditions: Waters ACQUITY UPC² system; OZ-H column (100 × 4.6 mm, 5 μm); CO_2/modifier, 60:40

(v/v); modifier, MeOH containing 0.2% (v/v) of a 7 M NH_3 solution in MeOH; flow rate, 3.0 mL min^{-1}; column temperature, 40 °C; back pressure, 2000 psi; PDA detection at 214 nm; injection volume, 3.0 μL. No opposite-enantiomer peak was detected for either (*S*)-**L1** or (*R*)-**L1** under the conditions used; the reported ee values were greater than 99%.

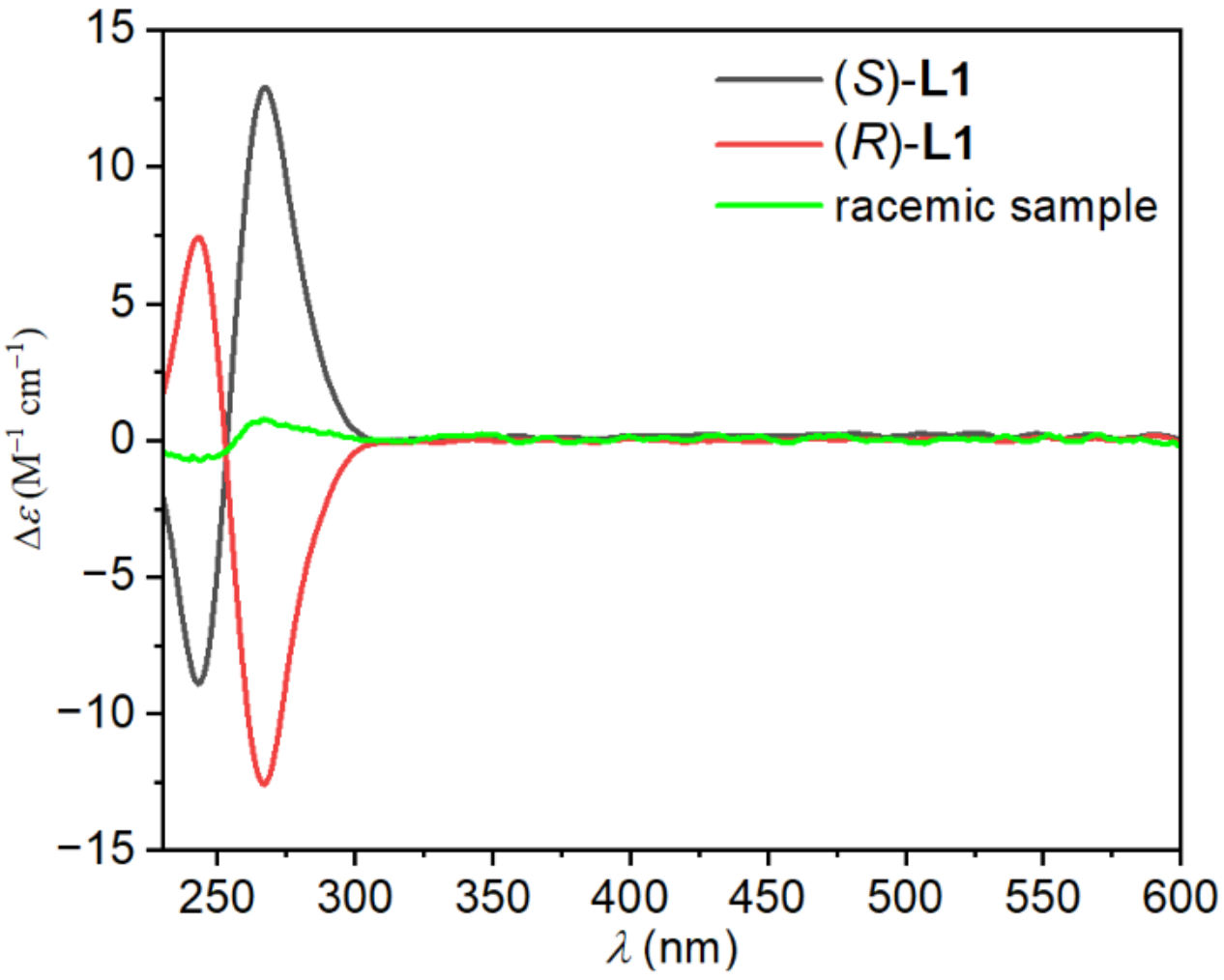

Fig. S201. CD spectra of (*S*)-**L1**, (*R*)-**L1**, and their 1:1 racemic mixture in methanol at room temperature (total ligand concentration, c_{ligand} = 2.4 mM; optical path length, *l* = 0.1 mm). Spectra are expressed as molar circular dichroism, Δε, calculated using the actual total ligand concentration. The individual enantiomers exhibit mirror-image CD profiles, whereas the racemic mixture shows negligible net CD.

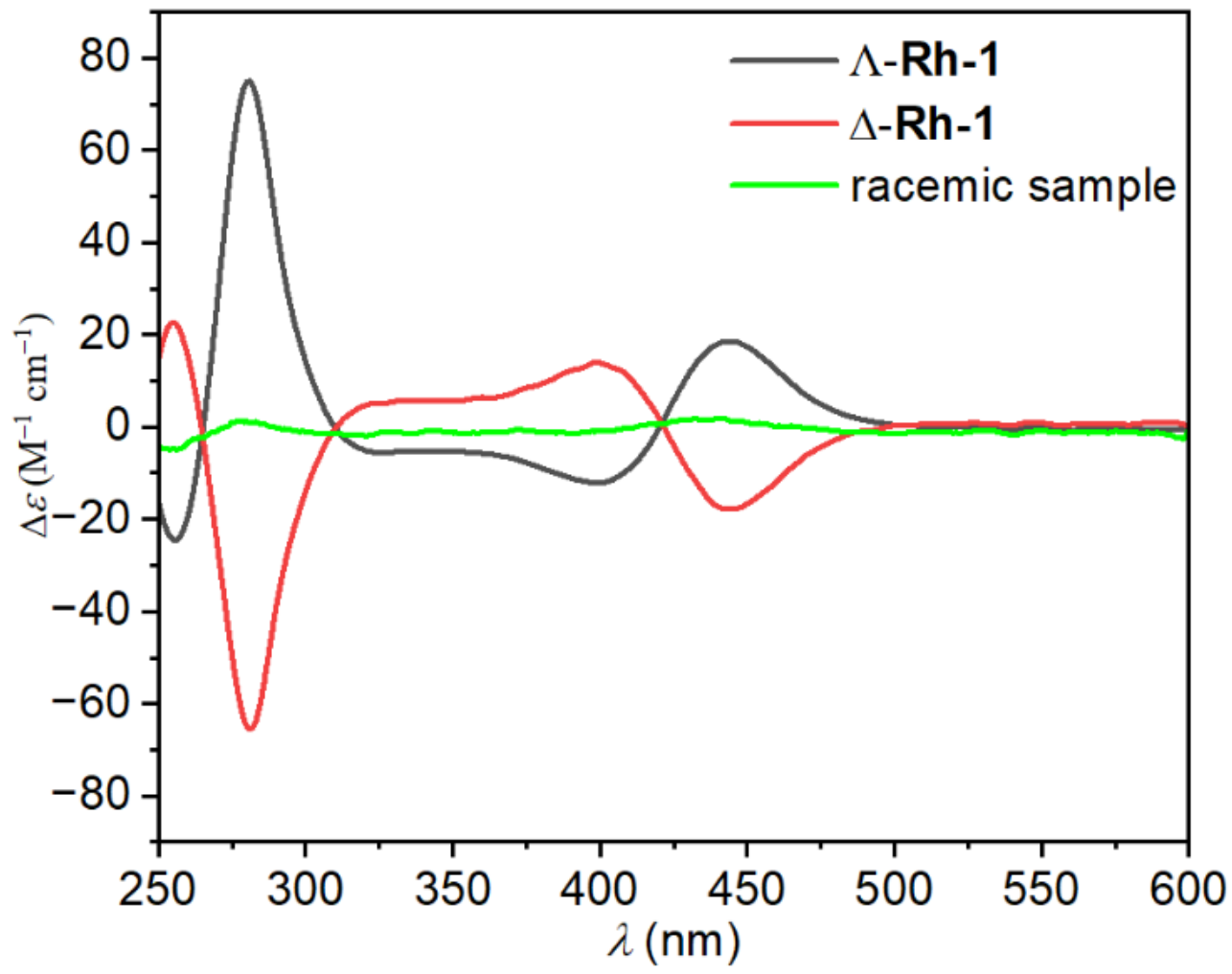

Fig. S202. CD spectra of Λ-**Rh-1**, Δ-**Rh-1**, and the racemic trefoil-knot mixture obtained by self-assembly of equimolar (*S*)-**L1** and (*R*)-**L1** with **B1**$(OTf)_2$ in methanol at room temperature (total knot concentration, *c* = 0.8 mM; c_{Rh} = 4.8 mM; optical path length, *l* = 0.1 mm). Spectra are expressed as molar circular dichroism, Δ*ε*, per mole of complete assembly. Λ-**Rh-1** and Δ-**Rh-1** exhibit mirror-image CD profiles, whereas the racemic mixture shows negligible net CD.

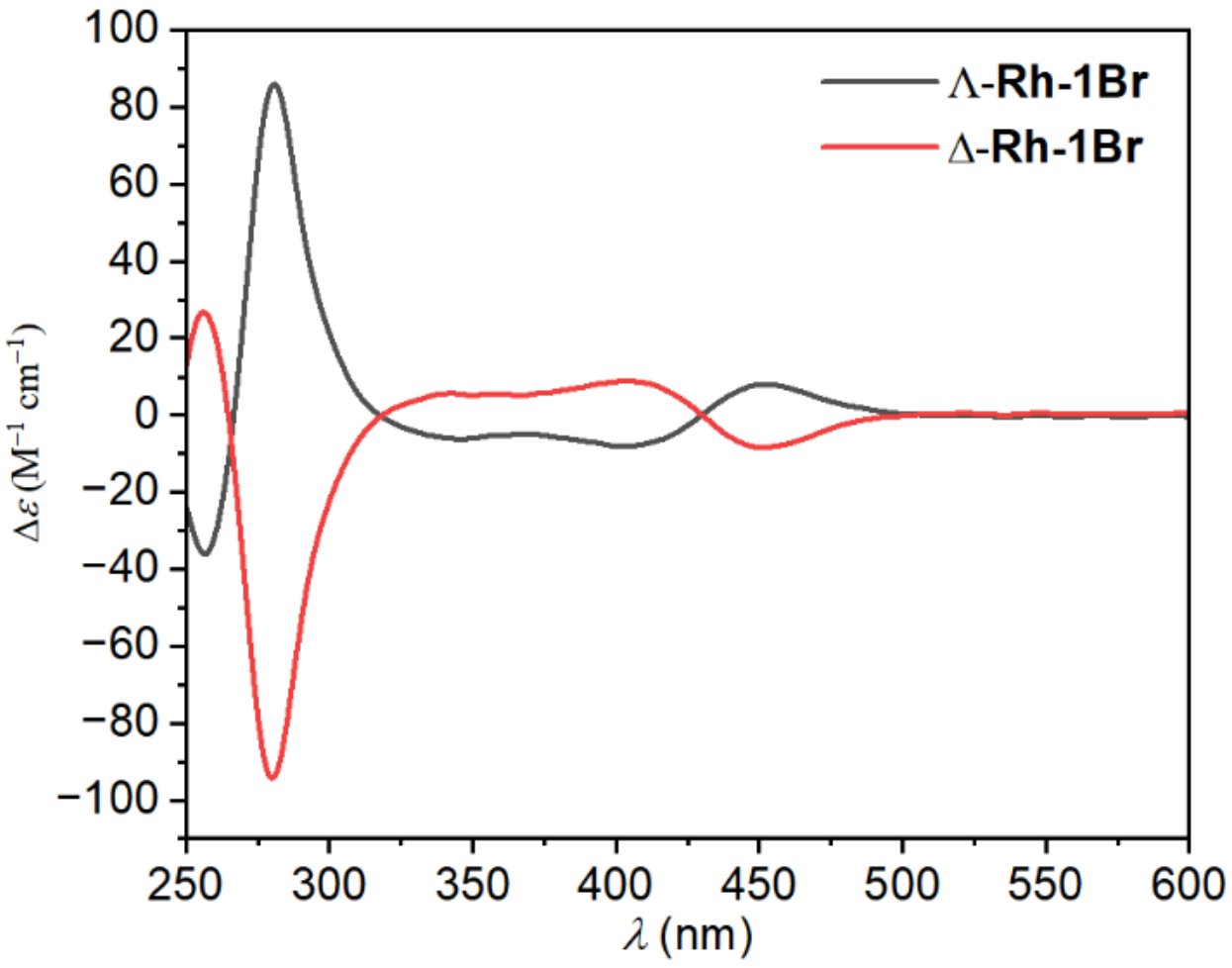

Fig. S203. CD spectra of Λ-**Rh-1Br** and Δ-**Rh-1Br** in methanol at room temperature (c = 0.8 mM, c_{Rh} = 4.8 mM; optical path length l = 0.1 mm). Spectra are expressed as molar circular dichroism, Δε, using concentrations calculated per complete assembly. The two assemblies exhibit approximately mirror-image CD profiles.

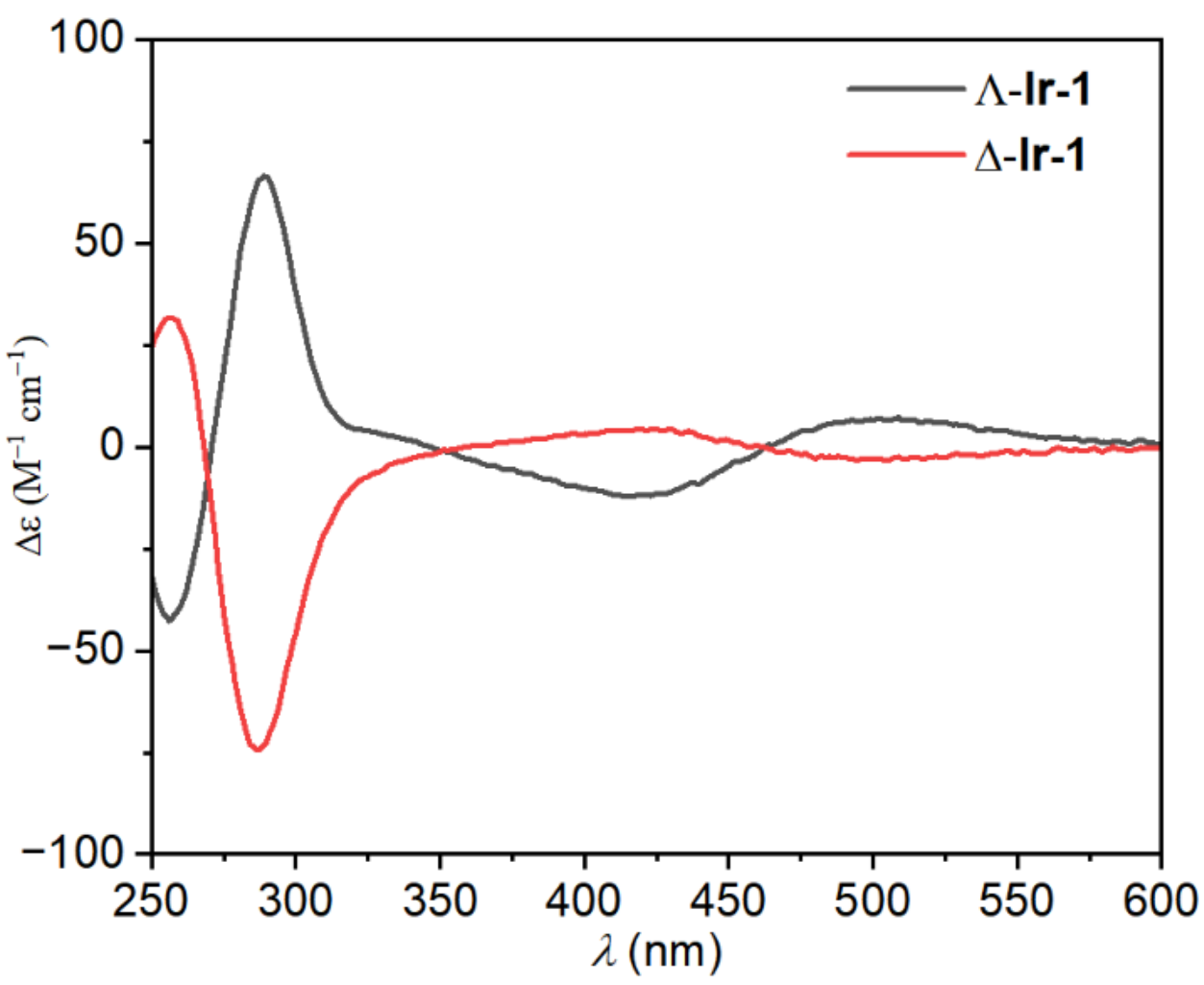

Fig. S204. CD spectra of Λ-**Ir-1** and Δ-**Ir-1** in methanol at room temperature (c = 0.8 mM, c_{Ir} = 4.8 mM; optical path length l = 0.1 mm). Spectra are expressed as molar circular dichroism, Δε, using concentrations calculated per complete assembly. The two assemblies exhibit approximately mirror-image CD profiles.

Table S1. Absorption dissymmetry factors of the trefoil-knot assemblies in MeOH at room temperature.

Assembly	Wavelength / nm[a]	g_{abs}

Λ-Rh-1	284	$+5.8 \times 10^{-4}$
Δ-Rh-1	284	-5.1×10^{-4}
Λ-Rh-1Br	283	$+6.3 \times 10^{-4}$
Δ-Rh-1Br	283	-6.6×10^{-4}
Λ-Ir-1	291	$+4.8 \times 10^{-4}$
Δ-Ir-1	291	-5.4×10^{-4}

[a]Wavelength of maximum $|g_{abs}|$ within the recorded spectral range. Signed g_{abs} values were calculated as g_{abs} = Δε/ε using the corresponding CD and UV–vis data expressed on the same molar basis.

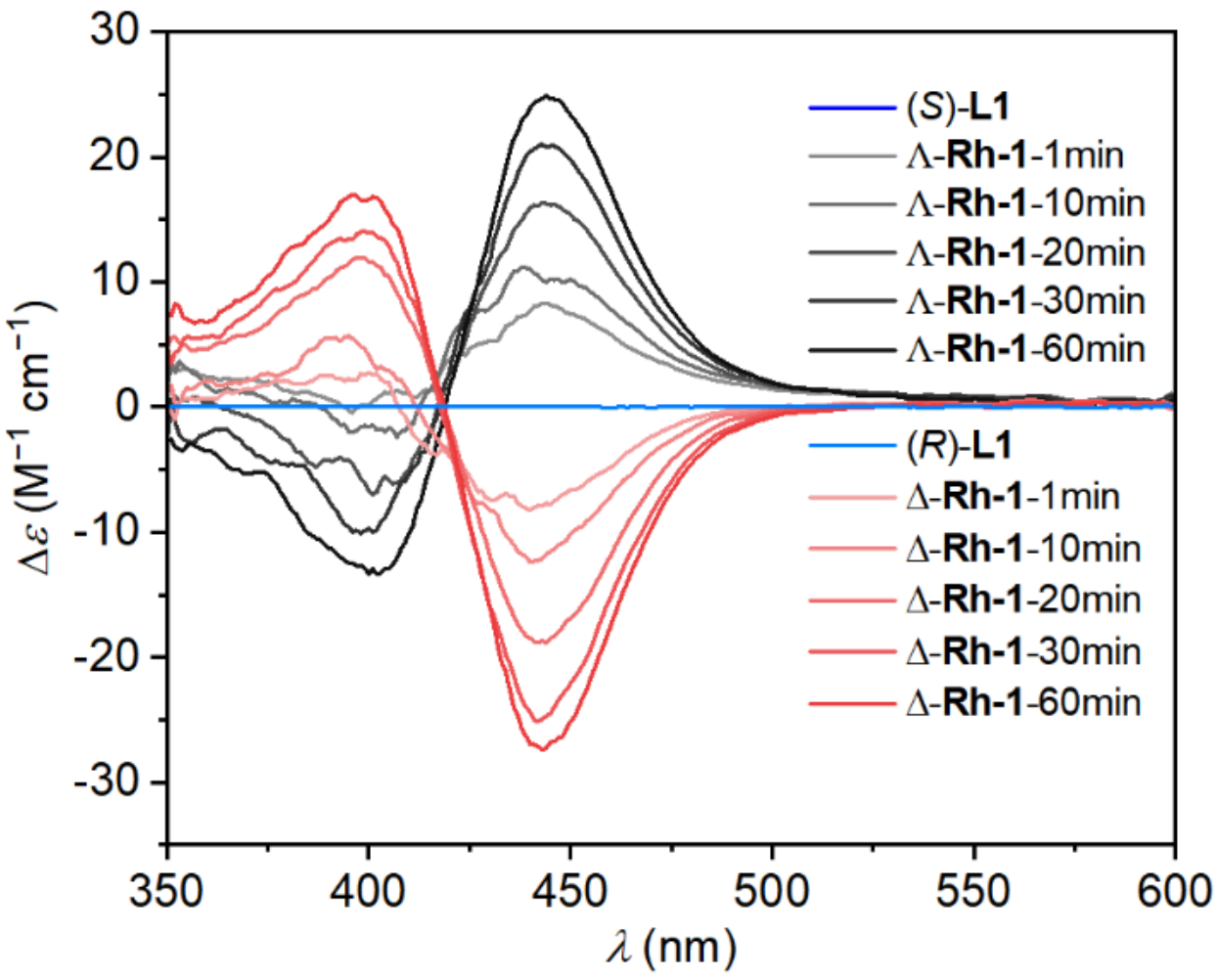

Fig. S205. Time-dependent CD spectra of the reaction mixtures of (*S*)-**L1** or (*R*)-**L1** with **B1**$(OTf)_2$ in methanol at room temperature over 1–60 min (c_{Rh} = 20.0 mM; optical path length l = 0.1 mm). Spectra are normalized to a formal knot concentration of $c = c_{Rh}/6$ = 3.333 mM and are expressed as molar circular

dichroism, Δε. Free-ligand references (c_{ligand} = 10.0 mM; optical path length l = 0.1 mm) are plotted as 3□□$_{ligand}$. Oppositely signed CD responses develop during assembly.

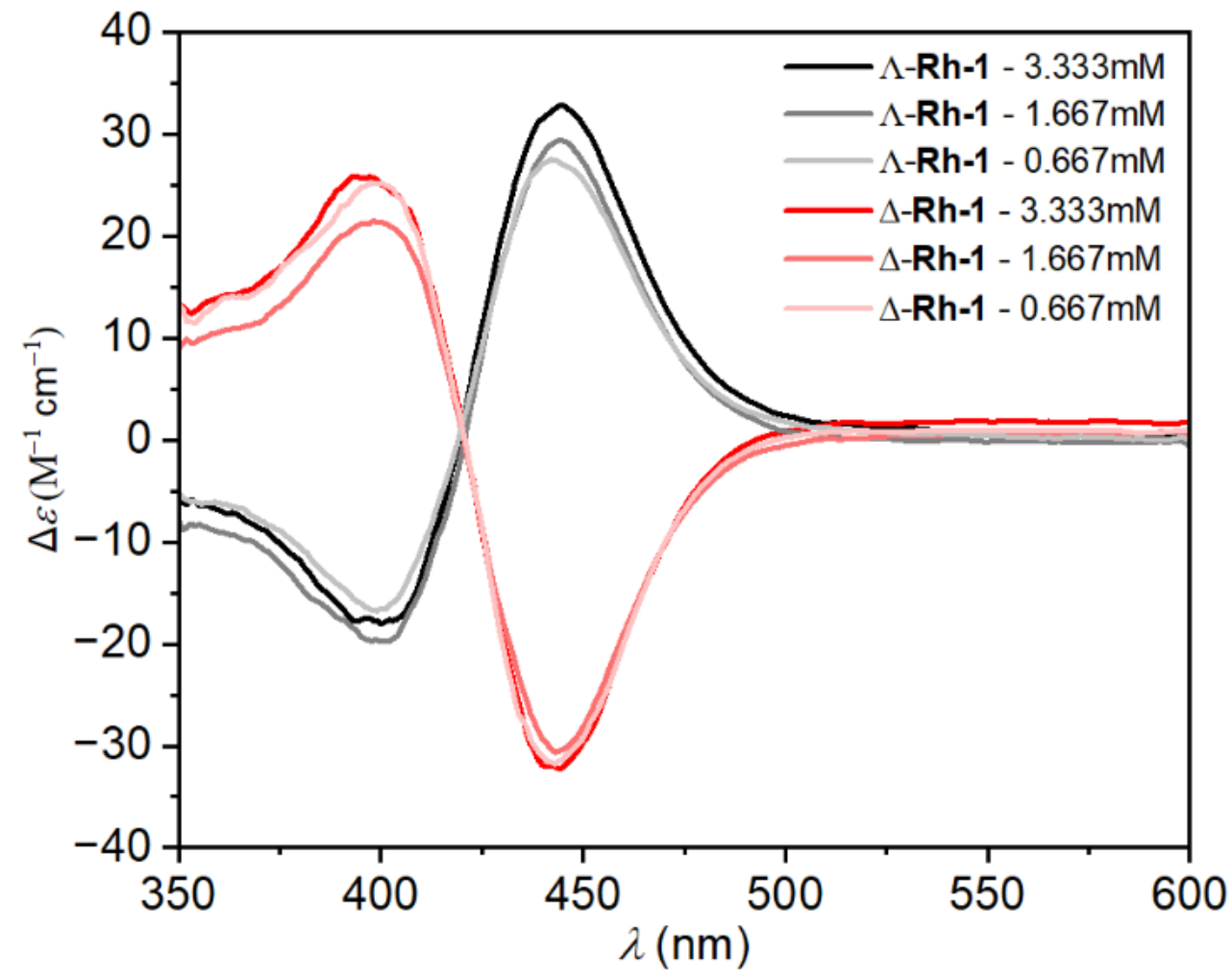

Fig. S206. Concentration-dependent CD spectra of Λ-**Rh-1** and Δ-**Rh-1** in methanol at room temperature. Formal knot concentrations of c = 0.667, 1.667, and 3.333 mM (c_{Rh} = 4.0, 10.0, and 20.0 mM) were examined using optical path lengths of 0.5, 0.2, and 0.1 mm, respectively. Spectra are expressed as molar circular dichroism, Δε, normalized using $c = c_{Rh}/6$. The molar CD profiles show only small changes over this concentration range.

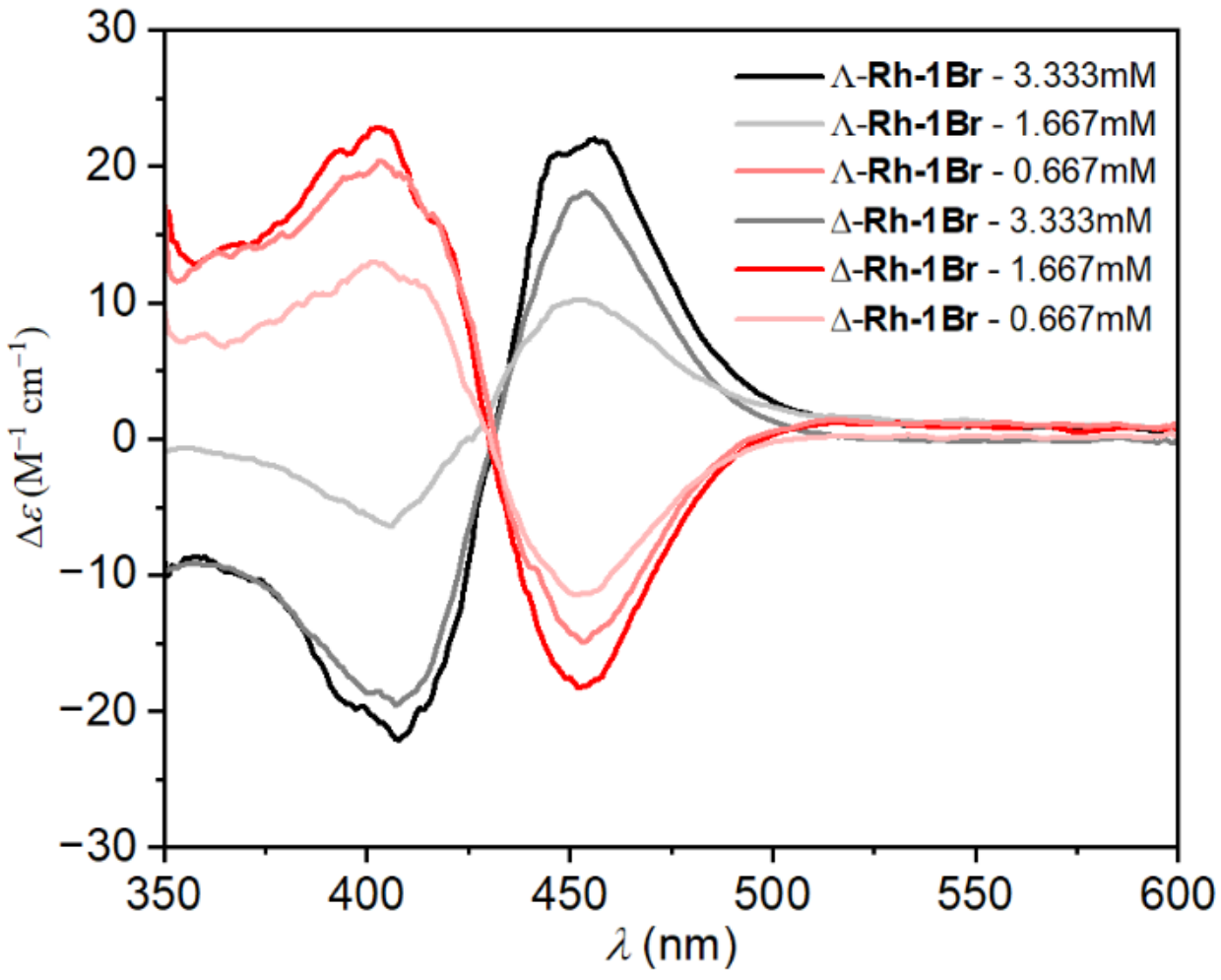

Fig. S207. Concentration-dependent CD spectra of Λ-**Rh-1Br** and Δ-**Rh-1Br** in methanol at room temperature. Formal knot concentrations of $c = 0.667$, 1.667, and 3.333 mM ($c_{\mathrm{Rh}} = 4.0$, 10.0, and 20.0 mM) were examined using optical path lengths of 0.5, 0.2, and 0.1 mm, respectively. Spectra are expressed as molar circular dichroism, Δε, normalized using $c = c_{\mathrm{Rh}}/6$. The molar CD amplitudes decrease upon dilution.

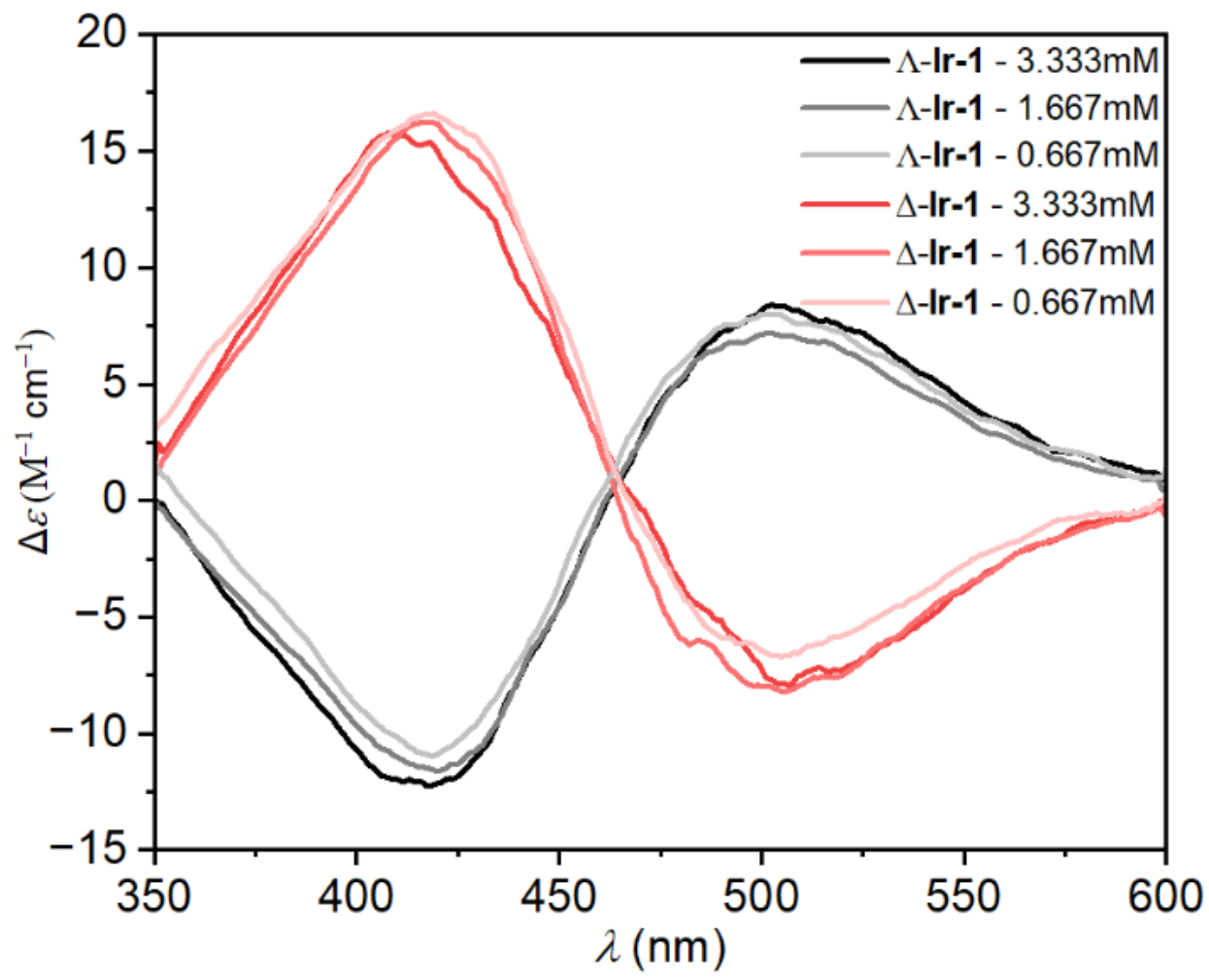

Fig. S208. Concentration-dependent CD spectra of Λ-**Ir-1** and Δ-**Ir-1** in methanol at room temperature. Formal knot concentrations of $c = 0.667$, 1.667, and 3.333 mM ($c_{\mathrm{Ir}} = 4.0$, 10.0, and 20.0 mM) were examined using optical path lengths of 0.5, 0.2, and 0.1 mm, respectively. Spectra are expressed as molar circular dichroism, Δε, normalized using $c = c_{\mathrm{Ir}}/6$. The molar CD profiles show only small changes over this concentration range.

9. X-ray Crystallography Details

Single-crystal X-ray diffraction data for the binuclear building blocks and the crystallographically characterized assemblies were collected on a Rigaku XtaLAB SynergyCustom diffraction system with microfocus rotating anode generator (2.97 kW, Cu $K_{α}$, λ= 1.54178 Å). The structures were solved by the direct method of SHELXS-97[6] and then refined on F^2 by full-matrix least-squares techniques using the SHELXL-2014[7] program. Where necessary, contributions from highly disordered guest molecules were treated using the SQUEEZE routine in PLATON.[8]

The crystallographic data have been deposited with the Cambridge Crystallographic Data Centre for the following structures: Λ-**Rh-1** (CCDC 2566605), Δ-**Rh-1** (CCDC 2566606), Λ-**Ir-1** (CCDC 2566608), Δ-**Ir-1** (CCDC 2566607), **Rh-2***S* (CCDC 2592947), **Ir-2***S* (CCDC 2592948), **Rh-3** (CCDC 2592949), **Ir-3** (CCDC 2592950), **Rh-4Br***S* (CCDC 2592951), **Rh-5** (CCDC 2592952), **Rh-5′** (CCDC 2592953), **Rh-5Br** (CCDC 2592954), **Ir-5** (CCDC 2592955), **Ir-5′** (CCDC 2592956), **B1**$(OTf)_2$ (CCDC 2592957), **B2**$(OTf)_2$ (CCDC 2592958), and **B3**$(OTf)_2$ (CCDC 2592959). The data for crystals 1–5 obtained from the racemic self-assembly experiment have been deposited under CCDC 2592960, 2592961, 2592962, 2592963, and 2592964, respectively. These data are freely available at https://www.ccdc.cam.ac.uk/structures. Crystal data and refinement parameters are summarized in Tables S2–S10.

Table S2. Crystal data and structure refinement for the trefoil knots Λ-**Rh-1** and Δ-**Rh-1**.

Complex	Λ-**Rh-1**	Δ-**Rh-1**
Empirical formula	$C_{142}H_{156}N_{12}O_{21}SF_3Rh_6$[a]	$C_{142}H_{156}N_{12}O_{21}SF_3Rh_6$[b]
Formula weight	3818.65	3818.65
Temperature, K	150(2)	150(2)
Crystal system	cubic	cubic
Space group	$P2_13$	$P2_13$
a, Å	27.2711(3)	27.2045(2)
b, Å	27.2711(3)	27.2045(2)
c, Å	27.2711(3)	27.2045(2)
α, °	90	90
β, °	90	90
γ, °	90	90
Volume, $Å^3$	20281.9(7)	20133.6(4)
Z	4	4
$ρ_{calc}$g, cm^3	1.251	1.260
μ/mm^{-1}	5.106	5.144
F(000)	7752.0	7752.0
Crystal size, mm^3	0.258 × 0.246 × 0.145	0.314 × 0.238 × 0.169
Radiation	CuKα (λ = 1.54178)	$CuK_α$ (λ= 1.54178)
2θ range for data collection, °	4.582 to 153.352	4.594 to 153.102
Index ranges	-30 ≤ *h* ≤ 34, -26 ≤ *k* ≤ 33, -26 ≤ *l* ≤ 21	-27 ≤ *h* ≤ 33, -33 ≤ *k* ≤ 31, -18 ≤ *l* ≤ 30
Reflections collected	45739	45885
Independent reflections	13478 [R_{int}= 0.0336, R_{sigma}= 0.0254]	13418 [R_{int}= 0.0218, R_{sigma}= 0.0158]
Data/restraints/parameters	13478/578/532	13418/0/556
Goodness-of-fit on F^2	0.995	1.218
R_1/wR_2[c] ($I > 2□(I)$)	0.0375/0.1213	0.0366/0.1347
R_1/wR_2[c] (all data)	0.0418/0.1258	0.0391/0.1387
Largest diff. peak and hole, e/$Å^3$	0.42/-0.54	0.29/-0.61
Flack parameter	0.004(5)	0.010(4)

[a]In the asymmetric unit of □-**Rh-1**, there were disordered anions (five triflate anions) and solvents which could not be restrained properly. Therefore, SQUEEZE algorithm was used to omit them.

[b]In the asymmetric unit of Δ-**Rh-1**, there were disordered anions (five triflate anions) and solvents which could not be restrained properly. Therefore, SQUEEZE algorithm was used to omit them.

[c]$R_1 = \square||F_o|-|F_c||/\square|F_o|$, $wR_2 = [\square w(F_o^2-F_c^2)^2/\square w(F_o^2)^2]^{1/2}$

Table S3. Crystal data and structure refinement for the trefoil knots Λ-**Ir-1** and Δ-**Ir-1**.

Complex	Λ-**Ir-1**	Δ-**Ir-1**
Empirical formula	$C_{142}H_{156}N_{12}O_{21}SF_3Ir_6$[a]	$C_{142}H_{156}N_{12}O_{21}SF_3Ir_6$[b]
Formula weight	4354.39	4354.39
Temperature, K	150(2)	150(2)
Crystal system	cubic	cubic
Space group	$P2_13$	$P2_13$
a, Å	27.2831(2)	27.2674(2)
b, Å	27.2831(2)	27.2674(2)
c, Å	27.2831(2)	27.2674(2)
α, °	90	90
β, °	90	90
γ, °	90	90
Volume/Å^3	20308.7(4)	20273.6(4)
Z	4	4
$ρ_{calc}$g, cm^3	1.424	1.427
μ, mm^{-1}	8.657	8.672
F(000)	8520.0	8520.0
Crystal size, mm^3	0.275 × 0.173 × 0.108	0.346 × 0.203 × 0.149
Radiation	CuK$_α$ (λ = 1.54178)	CuK$_α$ (λ = 1.54178)
2θ range for data collection, °	4.58 to 153.342	4.582 to 153.622
Index ranges	-30 ≤ *h* ≤ 21, -30 ≤ *k* ≤ 11, -27 ≤ *l* ≤ 33	-32 ≤ *h* ≤ 29, -21 ≤ *k* ≤ 30, -34 ≤ *l* ≤ 30
Reflections collected	45403	46124
Independent reflections	13211 [R_{int}= 0.0275, R_{sigma}= 0.0240]	13480 [R_{int}= 0.0326, R_{sigma}= 0.0260]
Data/restraints/parameters	13211/578/519	13480/0/508
Goodness-of-fit on F^2	0.931	0.848
R_1/wR_2[c] (I > 2□(I))	0.0387/0.1203	0.0346/0.1062
R_1/wR_2[c] (all data)	0.0486/0.1289	0.0396/0.1107
Largest diff. peak and hole, e/Å^3	0.94/-0.38	0.96/-0.25
Flack parameter	0.012(5)	0.009(5)

[a]In the asymmetric unit of □-**Ir-1**, there were disordered anions (five triflate anions) and solvents which could not be restrained properly. Therefore, SQUEEZE algorithm was used to omit them.

[b]In the asymmetric unit of Δ-**Ir-1**, there were disordered anions (five triflate anions) and solvents which could not be restrained properly. Therefore, SQUEEZE algorithm was used to omit them.

[c]$R_1 = Σ||F_o|-|F_c||/Σ|F_o|$, $wR_2 = [Σw(F_o^2-F_c^2)^2/Σw(F_o^2)^2]^{1/2}$

Table S4. Crystal data and structure refinement for the D-shaped metallacycles **Rh-2*S*** and **Ir-2*S***.

Complex	**Rh-2*S***	**Ir-2*S***
Empirical formula	$C_{54}H_{56}N_4O_9SF_3Rh_2$ [a]	$C_{53}H_{56}N_4O_6Ir_2$ [b]
Formula weight	1143.71	1229.41
Temperature, K	150(2)	150(2)
Crystal system	monoclinic	monoclinic
Space group	*C*2	*C*2
a, Å	18.5692(4)	18.3102(4)
b, Å	16.3359(3)	16.4877(3)
c, Å	23.4192(7)	23.0638(6)
α, °	90	90
β, °	110.927(3)	110.770(3)
γ, °	90	90
Volume/Å3	6635.5(3)	6510.3(3)
Z	4	4
$ρ_{calc}$g, cm3	1.145	1.254
μ, mm^{-1}	4.783	8.117
F(000)	2229.0	2416.0
Crystal size, mm3	0.485 × 0.260 × 0.257	0.148 × 0.138 × 0.090
Radiation	$CuK_α$ (λ = 1.54178)	$CuK_α$ (λ = 1.54178)
2θ range for data collection, °	7.434 to 133.186	4.098 to 133.184
Index ranges	-20 ≤ h ≤ 22, -18 ≤ k ≤ 19, -27 ≤ l ≤ 27	-21 ≤ h ≤ 21, -19 ≤ k ≤ 19, -27 ≤ l ≤ 27
Reflections collected	46658	39391
Independent reflections	11444 [R_{int} = 0.0793, R_{sigma} = 0.0485]	11486 [R_{int} = 0.0589, R_{sigma} = 0.0448]
Data/restraints/parameters	11444/692/574	11486/625/490
Goodness-of-fit on F^2	1.289	0.975
R_1/wR_2 [c] ($I > 2□(I)$)	0.0838/0.2643	0.0605/0.2078
R_1/wR_2 [c] (all data)	0.0901/0.2804	0.0678/0.2230
Largest diff. peak and hole, e/Å3	1.85/-0.58	2.46/-1.23
Flack parameter	0.018(14)	0.046(11)

[a]In the asymmetric unit of **Rh-2***S*, there were disordered anions (one triflate anions) and solvents which could not be restrained properly. Therefore, SQUEEZE algorithm was used to omit them.

[b]In the asymmetric unit of **Ir-2***S*, there were disordered anions (two triflate anions) and solvents which could not be restrained properly. Therefore, SQUEEZE algorithm was used to omit them.

[c]$R_1 = \Sigma||F_o|-|F_c||/\Sigma|F_o|$, $wR_2 = [\Sigma w(F_o^2-F_c^2)^2/\Sigma w(F_o^2)^2]^{1/2}$

Table S5. Crystal data and structure refinement for the nested dimers **Rh-3** and **Ir-3**.

Complex	**Rh-3**	**Ir-3**
Empirical formula	$C_{192}H_{216}N_{16}O_{24}Rh_8$[a]	$C_{97}H_{108}N_8O_{15}SF_3Ir_4$[b]
Formula weight	1977.54	1591.66
Temperature, K	150(2)	150(2)
Crystal system	monoclinic	monoclinic
Space group	*P*n	*P*2₁/n
a, Å	17.9455(4)	17.9799(2)
b, Å	13.8885(3)	14.0957(2)
c, Å	51.3855(14)	50.5164(9)
α, °	90	90
β, °	92.692(2)	92.7844(14)
γ, °	90	90
Volume/Å^3	12793.0(5)	12787.7(3)
Z	2	4
ρ_{calc}g, cm^3	1.027	0.827
μ, mm^{-1}	4.469	6.637
F(000)	4064.0	3028.0
Crystal size, mm^3	0.393 × 0.157 × 0.136	0.303 × 0.148 × 0.125
Radiation	CuK$_\alpha$ (λ= 1.54178)	CuK$_\alpha$ (λ= 1.54178)
2θ range for data collection, °	5.144 to 153.166	5.142 to 153.766
Index ranges	-22 ≤ h ≤ 20, -16 ≤ k ≤ 17, -63 ≤ l ≤ 64	-22 ≤ *h* ≤ 22, -13 ≤ *k* ≤ 17, -63 ≤ *l* ≤ 59
Reflections collected	88889	92076
Independent reflections	36394 [R_{int}= 0.0680, R_{sigma}= 0.0571]	24987 [R_{int}= 0.0485, R_{sigma}= 0.0341]
Data/restraints/parameters	36394/3879/1790	24987/1256/1055
Goodness-of-fit on F^2	1.050	1.152
R_1/wR_2[c] ($I > 2\sigma(I)$)	0.1417/0.3640	0.1025/0.2533
R_1/wR_2[c] (all data)	0.1645/0.3881	0.1238/0.2684
Largest diff. peak and hole, e/Å^3	1.28/-1.07	3.69/-1.11
Flack parameter	0.416(17)	–

[a]In the asymmetric unit of **Rh-3**, there were disordered anions (eight triflate anions) and solvents which could not be restrained properly. Therefore, SQUEEZE algorithm was used to omit them.

[b]In the asymmetric unit of **Ir-3**, there were disordered anions (three triflate anions) and solvents which could not be restrained properly. Therefore, SQUEEZE algorithm was used to omit them.

[c]$R_1 = \Sigma||F_o|-|F_c||/\Sigma|F_o|$, $wR_2 = [\Sigma w(F_o^2-F_c^2)^2/\Sigma w(F_o^2)^2]^{1/2}$

Table S6. Crystal data and structure refinement for the figure-eight knots **Rh-4Br*S*** and **Rh-5Br**.

Complex	**Rh-4Br*S***	**Rh-5Br**
Empirical formula	$C_{377}H_{384}N_{32}O_{75}S_9F_{27}Br_{16}Rh_{16}$[a]	$C_{189}H_{200}N_{16}O_{27}SF_3Br_8Rh_8$[b]
Formula weight	10289.80	6203.37
Temperature, K	150(2)	150(2)
Crystal system	monoclinic	triclinic
Space group	$P2_1$	$P\bar{1}$
a, Å	24.0296(3)	16.7857(2)
b, Å	44.4115(5)	22.9068(3)
c, Å	24.8174(3)	35.4703(7)
α, °	90	75.9250(10)
β, °	109.4740(10)	76.7700(10)
γ, °	90	80.6290(10)
Volume/Å^3	24969.8(5)	12794.8(3)
Z	2	2
ρ_{calc}g, cm^3	1.369	1.610
μ, mm^{-1}	6.618	6.965
F(000)	10274.0	6252.0
Crystal size, mm^3	0.129 × 0.110 × 0.078	0.379 × 0.218 × 0.203
Radiation	CuK$_\alpha$ (λ = 1.54184)	CuK$_\alpha$ (λ = 1.54178)
2θ range for data collection, °	3.9 to 154.422	4.002 to 133.19
Index ranges	$-29 \le h \le 30$, $-54 \le k \le 53$, $-30 \le l \le 30$	$-19 \le h \le 17$, $-27 \le k \le 27$, $-36 \le l \le 42$
Reflections collected	326213	138180
Independent reflections	97243 [R_{int} = 0.1135, R_{sigma} = 0.0748]	44131 [R_{int} = 0.0390, R_{sigma} = 0.0313]
Data/restraints/parameters	97243/5226/4659	44131/7529/2101
Goodness-of-fit on F^2	1.033	0.990
R_1/wR_2[c] ($I > 2\sigma(I)$)	0.1019/0.2905	0.0971/0.2123
R_1/wR_2[c] (all data)	0.1403/0.3390	0.1157/0.2208
Largest diff. peak and hole, e/Å^3	3.33/-1.64	1.61/-1.12
Flack parameter	0.059(4)	–

[a]In the asymmetric unit of **Rh-4Br*S***, there were disordered anions (seven triflate anions) and solvents which could not be restrained properly. Therefore, SQUEEZE algorithm was used to omit them.

[b]In the asymmetric unit of **Rh-5Br**, there were disordered anions (seven triflate anions) and solvents which could not be restrained properly. Therefore, SQUEEZE algorithm was used to omit them.

[c]$R_1 = \Sigma||F_o|-|F_c||/\Sigma|F_o|$, $wR_2 = [\Sigma w(F_o^2-F_c^2)^2/\Sigma w(F_o^2)^2]^{1/2}$

Table S7. Crystal data and structure refinement for the figure-eight knots **Rh-5** and **Ir-5**.

Complex	**Rh-5**	**Ir-5**
Empirical formula	$C_{96}H_{104}N_8O_{18}S_2F_6Rh_4$[a]	$C_{189}H_{208}N_{16}O_{27}SF_3Ir_8$[b]
Formula weight	2247.63	6126.27
Temperature, K	150(2)	150(2)
Crystal system	tetragonal	monoclinic
Space group	$P4_2/n$	$C2/c$
a, Å	26.0592(3)	50.6669(13)
b, Å	26.0592(3)	34.0325(7)
c, Å	40.2522(7)	34.8690(7)
α, °	90	90
β, °	90	126.331(2)
γ, °	90	90
Volume/Å^3	27334.5(8)	48437(2)
Z	8	8
ρ_{calc}g, cm^3	1.092	1.680
μ, mm^{-1}	4.615	9.744
F(000)	9168.0	24160.0
Crystal size, mm^3	0.325 × 0.274 × 0.150	0.326 × 0.180 × 0.113
Radiation	CuK$_\alpha$ (λ = 1.54178)	CuK$_\alpha$ (λ = 1.54178)
2θ range for data collection, °	4.39 to 133.164	4.33 to 133.19
Index ranges	$-30 \le h \le 31, -30 \le k \le 31, -46 \le l \le 47$	$-48 \le h \le 60, -40 \le k \le 40, -41 \le l \le 27$
Reflections collected	284234	177103
Independent reflections	24012 [R_{int}= 0.1363, R_{sigma}= 0.0375]	42608 [R_{int}= 0.1071, R_{sigma}= 0.0687]
Data/restraints/parameters	24012/1251/1159	42608/7494/1869
Goodness-of-fit on F^2	1.235	1.093
R_1/wR_2[c] ($I > 2\sigma(I)$)	0.1252/0.3119	0.1139/0.2509
R_1/wR_2[c] (all data)	0.1491/ 0.3282	0.1839/0.2827
Largest diff. peak and hole, e/Å^3	0.91/-1.33	1.74/-2.15

[a]In the asymmetric unit of **Rh-5**, there were disordered anions (fourteen triflate anions) and solvents which could not be restrained properly. Therefore, SQUEEZE algorithm was used to omit them.

[b]In the asymmetric unit of **Ir-5**, there were disordered anions (seven triflate anions) and solvents which could not be restrained properly. Therefore, SQUEEZE algorithm was used to omit them.

[c]$R_1 = \Sigma||F_o|-|F_c||/\Sigma|F_o|$, $wR_2 = [\Sigma w(F_o^2-F_c^2)^2/\Sigma w(F_o^2)^2]^{1/2}$

Table S8. Crystal data and structure refinement for the tetranuclear metallomacrocycles **Rh-5′** and **Ir-5′**.

Complex	**Rh-5′**	**Ir-5′**
Empirical formula	$C_{48}H_{52}N_4O_9SF_3Rh_2$[a]	$C_{96}H_{104}N_8O_{18}S_2F_6Ir_4$[b]
Formula weight	1259.85	2875.82
Temperature, K	150(2)	150(2)
Crystal system	monoclinic	triclinic
Space group	*C*2/c	*P*-1
a, Å	30.3234(14)	17.0309(4)
b, Å	18.2486(7)	17.4321(4)
c, Å	27.8080(14)	25.1937(7)
α, °	90	72.331(2)
β, °	115.963(6)	79.995(2)
γ, °	90	78.665(2)
Volume/Å^3	13834.8(13)	6935.5(3)
Z	8	2
ρ_{calc}g, cm^3	1.210	1.377
μ, mm^{-1}	4.666	7.977
F(000)	5116.0	2812.0
Crystal size, mm^3	0.304 × 0.251 × 0.218	0.225 × 0.136 × 0.128
Radiation	CuK$_\alpha$ (λ= 1.54184)	CuK$_\alpha$ (λ= 1.54178)
2θ range for data collection, °	5.828 to 154.186	5.332 to 133.192
Index ranges	$-38 \leq h \leq 35, -21 \leq k \leq 22, -35 \leq l \leq 34$	$-20 \leq h \leq 20, -20 \leq k \leq 16, -29 \leq l \leq 29$
Reflections collected	54828	88236
Independent reflections	13729 [R_{int}= 0.0995, R_{sigma}= 0.0540]	24279 [R_{int}= 0.0765, R_{sigma}= 0.0514]
Data/restraints/parameters	13729/14/604	24276/1256/1207
Goodness-of-fit on F^2	1.582	1.052
R_1/wR_2[c] ($I > 2\sigma(I)$)	0.0797/0.2209	0.0813/0.2014
R_1/wR_2[c] (all data)	0.0904/0.2281	0.1056/0.2216
Largest diff. peak and hole, e/Å^3	1.30/-1.31	2.89/-1.16

[a]In the asymmetric unit of **Rh-5′**, there were disordered anions (three triflate anions) and solvents which could not be restrained properly. Therefore, SQUEEZE algorithm was used to omit them.

[b]In the asymmetric unit of **Ir-5′**, there were disordered anions (two triflate anions) and solvents which could not be restrained properly. Therefore, SQUEEZE algorithm was used to omit them.

[c]$R_1 = \Sigma||F_o|-|F_c||/\Sigma|F_o|$, $wR_2 = [\Sigma w(F_o^2-F_c^2)^2/\Sigma w(F_o^2)^2]^{1/2}$

Table S9. Crystal data and structure refinement for the binuclear building blocks **B1**(OTf)$_2$, **B2**(OTf)$_2$, and **B3**(OTf)$_2$.

Complex	**B1**(OTf)$_2$	**B2**(OTf)$_2$	**B3**(OTf)$_2$
Empirical formula	$C_{14}H_{16}O_5SF_3Rh$	$C_{14}H_{15}O_5SF_3BrRh$	$C_{15}H_{20}O_6SF_3Ir$[a]
Formula weight	912.47	1070.28	1091.30
Temperature, K	150(2)	150(2)	150(2)
Crystal system	triclinic	monoclinic	monoclinic
Space group	P-1	I2/m	$P2_1$/c
a, Å	7.0562(3)	8.5103(3)	15.5357(8)
b, Å	8.6603(4)	12.9037(6)	8.8274(3)
c, Å	14.4146(6)	16.0715(7)	15.6937(8)
α, °	80.038(4)	90	90
β, °	82.926(4)	94.686(4)	117.097(6)
γ, °	67.054(4)	90	90
Volume/Å^3	797.42(6)	1758.98(13)	1915.99(18)
Z	2	4	4
ρ_{calc}g, cm^3	1.900	2.021	1.892
μ, mm^{-1}	10.422	12.133	15.020
F(000)	456.0	1048.0	1009.0
Crystal size, mm^3	0.273 × 0.151 × 0.105	0.224 × 0.073 × 0.056	0.224 × 0.073 × 0.056
Radiation	CuK$_\alpha$ (λ = 1.54178)	CuK$_\alpha$ (λ = 1.54184)	CuK$_\alpha$ (λ = 1.54184)
2θ range for data collection, °	6.236 to 153.232	8.8 to 152.978	6.39 to 133.194
Index ranges	$-8 \le h \le 8, -10 \le k \le 10, -18 \le l \le 18$	$-7 \le h \le 10, -15 \le k \le 16, -19 \le l \le 20$	$-18 \le h \le 17, -10 \le k \le 7, -18 \le l \le 18$
Reflections collected	8110	6864	12286
Independent reflections	3115 [R_{int} = 0.0410, R_{sigma} = 0.0391]	1839 [R_{int} = 0.0367, R_{sigma} = 0.0296]	3386 [R_{int} = 0.0633, R_{sigma} = 0.0465]
Data/restraints/parameters	3115/0/217	1839/398/194	3386/13/255
Goodness-of-fit on F^2	1.300	1.067	1.132
R_1/wR_2[b] ($I > 2\sigma(I)$)	0.0418/0.1493	0.0622/0.1655	0.0596/0.1600
R_1/wR_2[b] (all data)	0.0444/0.1515	0.0646/0.1675	0.0710/0.1678
Largest diff. peak and hole, e/Å^3	0.79/-0.88	2.11/-2.07	3.00/-0.99

[a]In the asymmetric unit of **B3**(OTf)$_2$, there were disordered anions (one triflate anions) and solvents which could not be restrained properly. Therefore, SQUEEZE algorithm was used to omit them.

[b]$R_1 = \Sigma||F_o|-|F_c||/\Sigma|F_o|$, $wR_2 = [\Sigma w(F_o^2-F_c^2)^2/\Sigma w(F_o^2)^2]^{1/2}$

Table S10. Crystallographic characterization of individual crystals obtained from self-assembly of racemic (*S*)-**L1**/(*R*)-**L1** with **B1**$(OTf)_2$.

Crystal identifier	Ligand configuration	Knot assignment	Space group	Flack x	CCDC number
1	*R*	Δ-**Rh-1**	$P2_13$	0.004(3)	2592960
2	*S*	Λ-**Rh-1**	$P2_13$	0.009(4)	2592961
3	*R*	Δ-**Rh-1**	$P2_13$	-0.002(5)	2592962
4	*R*	Δ-**Rh-1**	$P2_13$	0.005(6)	2592963
5	*S*	Λ-**Rh-1**	$P2_13$	0.010(3)	2592964

10. References

1 W.-L. Shan, W.-X. Gao, Y.-J. Lin, G.-X. Jin, *Dalton Trans.*, 2018, **47**, 2769-2777.

2 Y. Lu, Y.-X. Deng, Y.-J. Lin, Y.-F. Han, L.-H. Weng, Z.-H. Li, G.-X. Jin, *Chem*, 2017, **3,** 110-121.

3 Z. Cui, Y. Lu, X. Gao, H.-J. Feng, G.-X. Jin, *J. Am. Chem. Soc.*, 2020, **142**, 13667-13671.

4 N. Giuseppone, J. L. Schmitt, L. Allouche, J. M. Lehn, *Angew. Chem. Int. Ed.*, 2008, **47**, 2235-2239.

5 Z. Zhang, H. Wang, X. Wang, Y. Li, B. Song, O. Bolarinwa, R. A. Reese, T. Zhang, X.-Q. Wang, J. Cai, B. Xu, M. Wang, C. Liu, H.-B. Yang, X. Li, *J. Am. Chem. Soc.*, 2017, **139**, 8174-8185.

6 G. M. Sheldrick, *SHELXS-97: Program for X-Ray Crystal Structure Solution*, University of Göttingen, Göttingen, 1997.

7 G. M. Sheldrick, *Acta Cryst.*, 2008, **A64**, 112-122.

8 A. L. Spek, *Acta Cryst.*, 2009, **D65**, 148-155.
